# The UK Coronavirus Job Retention Scheme and diet, physical activity, and sleep during the COVID-19 pandemic: evidence from eight longitudinal population surveys

**DOI:** 10.1186/s12916-022-02343-y

**Published:** 2022-04-06

**Authors:** Bożena Wielgoszewska, Jane Maddock, Michael J. Green, Giorgio Di Gessa, Sam Parsons, Gareth J. Griffith, Jazz Croft, Anna J. Stevenson, Charlotte Booth, Richard J. Silverwood, David Bann, Praveetha Patalay, Alun D. Hughes, Nishi Chaturvedi, Laura D. Howe, Emla Fitzsimons, Srinivasa Vittal Katikireddi, George B. Ploubidis

**Affiliations:** 1 Centre for Longitudinal Studies, UCL Social Research Institute, University College, London, UK; 2 MRC Unit for Lifelong Health and Ageing, University College London, London, UK; 3 MRC/CSO Social & Public Health Sciences Unit, University of Glasgow, Glasgow, UK; 4 Institute of Epidemiology and Health Care, University College London, London, UK; 5 MRC Integrative Epidemiology Unit, University of Bristol, Bristol, UK; 6 Centre for Genomic and Experimental Medicine, University of Edinburgh, Edinburgh, UK

**Keywords:** Health behaviours, Exercise, Fruit and vegetable consumption, Sleeping, Employment, Furlough

## Abstract

**Background:**

In March 2020, the UK implemented the Coronavirus Job Retention Scheme (furlough) to minimise job losses. Our aim was to investigate associations between furlough and diet, physical activity, and sleep during the early stages of the COVID-19 pandemic.

**Methods:**

We analysed data on 25,092 participants aged 16–66 years from eight UK longitudinal studies. Changes in employment, including being furloughed, were based on employment status before and during the first lockdown. Health behaviours included fruit and vegetable consumption, physical activity, and sleep. Study-specific estimates obtained using modified Poisson regression, adjusting for socio-demographic characteristics and pre-pandemic health and health behaviours, were statistically pooled using random effects meta-analysis. Associations were also stratified by sex, age, and education.

**Results:**

Across studies, between 8 and 25% of participants were furloughed. Compared to those who remained working, furloughed workers were slightly less likely to be physically inactive (*RR* = 0.85; [95% *CI* 0.75–0.97]; *I* ^2^ = 59%) and did not differ overall with respect to low fruit and vegetable consumption or atypical sleep, although findings for sleep were heterogenous (*I* ^2^ = 85%). In stratified analyses, furlough was associated with lower fruit and vegetable consumption among males (*RR* = 1.11; [1.01–1.22]; *I* ^2^ = 0%) but not females (*RR* = 0.84; [0.68–1.04]; *I* ^2^ = 65%). Considering changes in quantity, furloughed workers were more likely than those who remained working to report increases in fruit and vegetable consumption, exercise, and hours of sleep.

**Conclusions:**

Those furloughed exhibited similar health behaviours to those who remained in employment during the initial stages of the pandemic. There was little evidence to suggest that adoption of such social protection policies in the post-pandemic recovery period and during future economic crises had adverse effects on population health behaviours.

**Supplementary Information:**

The online version contains supplementary material available at 10.1186/s12916-022-02343-y.

## Background

Employment can be related to behaviours that are important for health such as diet, physical activity, and sleep [1–4]. The COVID-19 pandemic, social distancing measures, and a series of lockdowns have affected the economy and employment rates in the United Kingdom (UK) and worldwide [5, 6], so this could have short- and long-term effects on population health and health-related behaviours. However, the pandemic has also resulted in health care disruptions and closures of some sectors of the economy, including exercise facilities. In this unique situation, it is difficult to predict how health behaviours might be affected.

Moreover, social protection policies introduced during the pandemic may have moderated any health consequences of the COVID-19-related economic downturn. The UK Government launched its Coronavirus Job Retention Scheme (CJRS) in March 2020. The CJRS, widely referred to as ‘furlough’, provided employees unable to work due to the pandemic with 80% of pay (capped at £2,500 per month) [7]. By March 2021, 11.4 million employees had been furloughed and the number of people claiming unemployment-related benefits had increased by 1.4 million over the preceding year [8]. The number of people on furlough was at its peak between April and July 2020 and participation in this scheme receded in the following months [9]. These economic changes did not affect all groups equally. Younger workers, low earners, and women were more likely to work in disrupted sectors and, therefore, become unemployed or be furloughed [8, 10]. Thus, while impacts of furlough on health behaviours are unknown, they could have important implications for health inequalities.

We need to better understand how government intervention can affect health behaviours, especially schemes such as the CJRS that aim to mitigate the impact of lockdown and economic downturns via subsidised employment. With a particular focus on furlough, and using data on over 25,000 participants in eight longitudinal studies, we investigate associations between changes in employment status during the early stages of the pandemic and a range of health behaviours, namely fruit and vegetable consumption (as an indicator of diet), physical activity, and sleep. We also examined associations stratified by sex, education, and age, as we hypothesised that associations could differ by these characteristics. We have explored associations of furlough with smoking and alcohol consumption elsewhere [11].

## Methods

### Participants

The UK National Core Studies Longitudinal Health and Wellbeing initiative draws together data from multiple UK population-based longitudinal studies and analyses these data to answer priority pandemic-related questions. By conducting similar analyses within each study and pooling results in a meta-analysis, we can provide robust evidence to understand how the pandemic has impacted population health, and support efforts to mitigate effects going forward. Data were obtained from eight long-running UK population-based longitudinal studies, each of which had conducted surveys during the pandemic (which we refer to as COVID surveys). Details of the design, sample frames, current age range, timing of the most recent pre-pandemic and COVID surveys, response rates, and sample size are in Additional file 1: Table S1 [12–25].

Five studies were age homogenous birth cohorts (where all individuals within each study were of similar age): the Millennium Cohort Study (MCS), the index children from the Avon Longitudinal Study of Parents and Children (ALSPAC-G1), Next Steps (NS, formerly the Longitudinal Study of Young People in England), the 1970 British Cohort Study (BCS70), and the 1958 National Child Development Study (NCDS). Three age heterogeneous studies (each covering a range of age groups) were included: Understanding Society (USOC), the English Longitudinal Study of Ageing (ELSA), and Generation Scotland: the Scottish Family Health Study (GS). Finally, the parents of the ALSPAC-G1 cohort were treated as a fourth age heterogeneous study population (ALSPAC-G0).

Analytical samples were restricted to participants of working age, defined as those aged 16–66 years based on the current state pension age in the UK [26], and to those who had recorded at least one health behaviour outcome in a COVID-19 survey and had valid data on all covariates. Most studies were weighted to restore representativeness to their target populations, accounting for sampling design where appropriate, and differential non-response to pre-pandemic and COVID surveys [27]. Weights were not available for GS. Details of the weighting applied within each study are in Additional file 1: Table S1.

### Measures

In this section, we describe all variables in the analysis. Full details of the questions and coding used for each cohort are in Additional file 2.

#### Exposure: employment status change

Employment status change (or stability) was coded in six categories based on the status both prior to the pandemic and at their first COVID-19 survey: stable employed (reference group), furloughed (i.e. from work to furlough), no longer employed (i.e. from employed to non-employed), became employed (i.e. from non-employed to employed), stable unemployed (i.e. unemployed at both points), and stable non-employed (i.e. not available for employment at either point, including in education, early retirement, caring responsibilities, sick, or disabled).

#### Outcomes: health behaviours

We examined diet, physical activity, and sleep. Participants self-reported fruit and vegetable consumption (≤2 portions per day vs more portions [28]), time spent exercising (<3 days a week for 30 min or more vs more frequent exercise within recommended levels [29]), and hours of sleep (outside the typical range of 6–9 h vs within that range [30]) both during and pre-pandemic. However, this information, used for our main analyses, was only available in some studies (MCS, NS, BCS, NCDS, USOC), whereas others (ALSPAC, GS, ELSA) only had information on change since the start of the pandemic (see Additional file 2). Based on these levels *or* on the information on changes in health behaviours since the start of the pandemic, we additionally created dichotomous outcomes indicating change from before to during the pandemic (in comparison to no change or change in the other direction): more portions of fruit/vegetables, fewer portions of fruit/vegetables, more time spent exercising, less time spent exercising, more hours of sleep, fewer hours of sleep, a shift from outside to within the typical sleep range of 6–9 h, and a shift from within to outside the typical sleep range of 6–9 h. All information on behaviours during the pandemic was from surveys conducted between April and July 2020 (inclusive).

#### Confounders and moderators

Potential confounders included sex, ethnicity (non-white ethnic minority vs white, including white ethnic minorities), age, education (degree vs no degree), UK nation (i.e. England, Wales, Scotland, Northern Ireland or other), household composition (based on presence of a spouse/partner and presence of children), pre-pandemic psychological distress, pre-pandemic self-rated health (excellent-good vs fair-poor), and pre-pandemic health behaviour measures, where available.

We examined modification of the associations by sex, education (degree vs no degree holders), and age in three categories: 16–29, 30–49, and 50 years or more (with age-homogeneous cohorts included in the relevant band).

### Analysis

Within each study, each outcome was regressed on employment status change, using a modified Poisson model with robust standard errors that returns risk ratios for ease of interpretation and avoids issues related to non-collapsibility of odds ratios [31, 32]. After estimating unadjusted associations, confounder adjustment was performed in two steps. First, a “basic” adjustment including socio-demographic characteristics: age (only in age heterogeneous studies), sex, ethnicity (except the BCS70 and NCDS cohorts who were almost entirely white), education, UK nation (except ALSPAC, GS, and ELSA which only had participants from a single country), and household composition. Second, a “full” adjustment additionally including pre-pandemic measures of psychological distress, self-rated health, and health behaviours. Moderation by sex, age, and education was assessed with stratified regressions using “full” adjustment.

Both stages of adjustment are relevant because our exposure, employment change, incorporates pre-pandemic employment status, which may have influenced other pre-pandemic characteristics such as mental health, self-rated health, and health behaviours (see Additional file 1: Fig. S8). By not controlling for these pre-pandemic characteristics, the basic adjusted risk ratios may represent both newly acquired behaviour and/or continuation of established (pre-pandemic) behaviour. In contrast, the full adjustment risk ratios block effects via these pre-pandemic characteristics and can therefore be interpreted as representing the differential change in health behaviour between exposure groups which is independent of these pre-pandemic characteristics. For the outcomes that directly capture changes in health behaviour, the full adjustment did not include pre-pandemic levels of the behaviour in question, as pre-pandemic levels of that behaviour are incorporated within the change outcome. This means that even full adjustment risk ratios estimated for these outcomes may partially reflect associations with pre-pandemic behaviour.

The overall and stratified results from each study were pooled using a random effects meta-analysis with restricted maximum likelihood in Stata. To explore the role of potential moderators, a subgroup analysis was performed which meta-analysed findings separately within each category of each moderator and performed a test of between-group differences. For stratified results, a test of group differences was performed using the subgroup meta-analysis command. Some studies could not contribute estimates for every comparison due to differences in the ages sampled, measures used, and sparsity of data. For a small number of exposure-outcome comparisons, reliable estimates could not be computed because the outcome prevalence was low (≤2). While such selective exclusion could potentially lead to bias, the low numbers of events mean that the corresponding within-study estimates would be so imprecise that their exclusion is unlikely to lead to considerable bias (see Additional files 3 and 4 for more details and sensitivity analyses showing that results were robust to different low cell count exclusion thresholds). We report heterogeneity using the *I* ^2^ statistic: 0% indicates estimates were similar across studies, while values closer to 100% represent greater heterogeneity. While we could have undertaken a multivariate meta-analysis of all exposure categories simultaneously, for ease of interpretation, we instead conducted a series of univariate meta-analyses, bearing in mind the consistency of results from these approaches generally observed elsewhere [33, 34]. We performed a multivariate meta-analysis with one outcome in a subset of the studies as a sensitivity analysis, and differences from the individual univariate meta-analyses were negligible (results not shown).

## Results

**Fig. 1:**
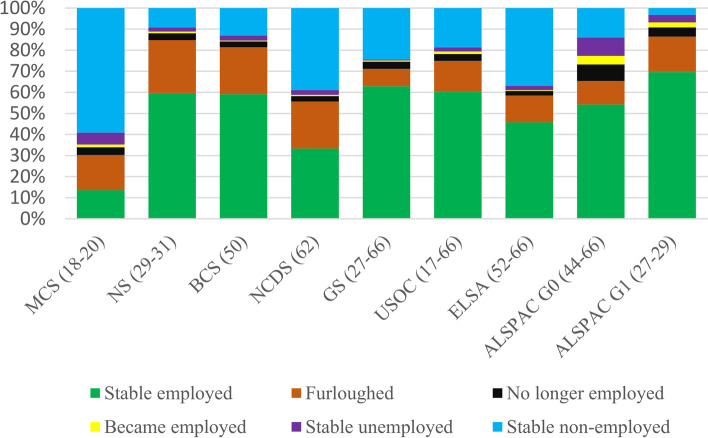
Percent distribution of change in employment status during the pandemic by study. Additional file 1: Table 1 has details of each study’s sample design and weighting applied. Analysis for GS, USOC, and ELSA restricted to participants aged 66 and younger. For more information about the questions asked in each dataset to derive changes in economic activity, please see Additional file 2

Analyses included 25,092 individuals from eight studies (see Additional file 1: Table S3 for demographic characteristics). Figure 1 shows employment status change during the first lockdown of the pandemic. Around six in 10 participants in NS, BCS70, GS, USOC, and ALSPAC were employed prior to and during the initial stages of the pandemic, with the younger (MCS) and older studies (ELSA and NCDS) showing lower levels of stable employment. The prevalence of furlough ranged between 8% (GS) and 25% (NS). Across most studies, approximately 3% of participants were no longer employed during the pandemic (8% in ALSPAC G0). Stable unemployment ranged in prevalence between 1% (GS) and 9% (ALSPAC G0). Additional file 1: Table S4 shows how the economic activity was patterned by education, sex, and age groups, with furlough generally more common among younger, female, and less educated participants and stable employment especially common among male, higher educated, and middle-aged participants. There were no clear patterns across studies regarding who was no longer employed during the pandemic.

Table 1 shows the prevalence of health behaviours and changes in behaviour by study. Proportions reporting eating no more than 2 portions of fruit or vegetables per day and reporting three or less days a week with at least 30 min of exercise were similar both pre- and during the pandemic, whereas sleep outside of the typical range of 6–9 h was more common during the pandemic in most studies (USOC was an exception). Nevertheless, changes in all three behaviours were common in both directions. In the four national birth cohorts (which used identical questions), more participants reported increasing their fruit and vegetable consumption and exercise than those who reported decreases, while reporting more hours of sleep and shifts to sleep outside the typical range were more common than reporting fewer hours of sleep or shifts from outside to within the typical range.

**Table 1:** Percent (and *N*) distribution of health behaviours and changes during the pandemic by study

	MCS	NS	BCS70	NCDS	GS	USOC	ELSA	ALSPAC G0	ALSPAC G1
**Age range of participants**	**18–20**	**29–31**	**50**	**62**	**27–66**	**17–66**	**52–66**	**44–66**	**27–29**
***N*** **participants**	**1924**	**1493**	**3050**	**4195**	**2618**	**6051**	**2417**	**2071**	**1273**
**Diet**									
Pre-pandemic: ≤ 2 portions of fruit and vegetables, % (*n*)	39 (657)	27.8 (384)	24.9 (673)	22.4 (853)	NA	26.5 (1070)	NA	NA	NA
During pandemic: ≤ 2 portions of fruit and vegetables, % (*n*)	33.3 (564)	26.1 (393)	25.3 (676)	21.9 (808)	NA	29.3 (1271)	NA	NA	NA
Eating fewer portions of fruit and vegetables, % (*n*)	17.1 (365)	15.2 (282)	14.9 (450)	10.9 (419)	NA	48.2 (2823)	NA	NA	NA
Eating more portions of fruit and vegetables, % (*n*)	32 (651)	21.5 (344)	17.2 (528)	14.6 (655)	NA	42.2 (2596)	NA	NA	NA
**Physical activity (PA)**									
Pre-pandemic: ≤ 3 days a week of at least 30m exercise, % (*n*)	43.4 (885)	46.9 (704)	43.5 (1319)	41.5 (1744)	22.7 (594)	21.2 (1181)	NA	NA	NA
During pandemic: ≤ 3 days a week of at least 30m exercise, % (*n*)	42.9 (803)	44.5 (677)	38.4 (1099)	39.9 (1588)	26.3 (608)	20.3 (994)	NA	NA	NA
Less PA/fewer days of +30m exercise, % (*n*)	33.6 (622)	29.7 (445)	20.5 (624)	18.2 (787)	31.7 (804)	49.3 (2824)	36.0 (869)	33.5 (693)	38.5 (491)
More PA/days of at least 30m exercise, % (*n*)	37.8 (750)	35.4 (544)	31.6 (1074)	26.8 (1232)	23.4 (658)	47.4 (3056)	23.3 (563)	44.4 (919)	42.5 (542)
**Sleep**									
Pre-pandemic: # hours/day, mean [95% *CI*]	7.48 [7.38–7.58]	7.13 [7.04–7.21]	6.88 [6.82–6.95]	6.93 [6.87–6.99]	7.09 [7.04–7.13]	6.82 [6.77–6.88]	NA	NA	NA
During pandemic: # hours/day, mean [95% *CI*]	8.12 [7.99–8.25]	7.41 [7.29-7.54]	6.98 [6.90–7.06]	6.99 [6.92–7.07]	7.10 [7.05–7.15]	7.01 [6.95–7.07]	NA	NA	NA
Pre-pandemic: <6 or 9+ h a night, % (*n*)	12.0 (223)	6.8 (107)	10.3 (229)	10.2 (323)	9.3 (243)	14.6 (740)	NA	NA	NA
During pandemic: <6 or 9+ h a night, % (*n*)	29.9 (569)	15.9 (231)	17.1 (430)	16.3 (540)	13.5 (347)	12.2 (673)	NA	NA	NA
From 6/9h a night to outside typical range, % (*n*)	24.6 (465)	12.0 (171)	9.6 (276)	7.8 (287)	9.0 (235)	5.4 (321)	NA	NA	NA
From outside typical range to 6/9h a night, % (*n*)	6.6 (118)	2.8 (47)	2.7 (74)	1.5 (68)	5.9 (171)	7.6 (370)	NA	NA	NA
Sleeps less than before, % (*n*)	23 (403)	22 (335)	19.6 (614)	16.7 (623)	21.4 (532)	30.8 (1903)	25.6 (618)	20.9 (432)	22.0 (280)
Sleeps more than before, % (*n*)	54.1 (1093)	36.3 (543)	27.6 (879)	21.7 (937)	22.1 (599)	44.6 (2482)	10.3 (249)	21.2 (439)	36.3 (462)

Additional file 1: Table 1 has details of each study’s sample design and weighting applied. Percentages and means are weighted (where weighting was applied), but *N* are unweighted. Analysis for GS, USOC, and ELSA restricted to participants aged 66 and younger. For more information about the questions asked in each dataset, please see Supplementary file 2

### Pooled analysis

**Fig. 2:**
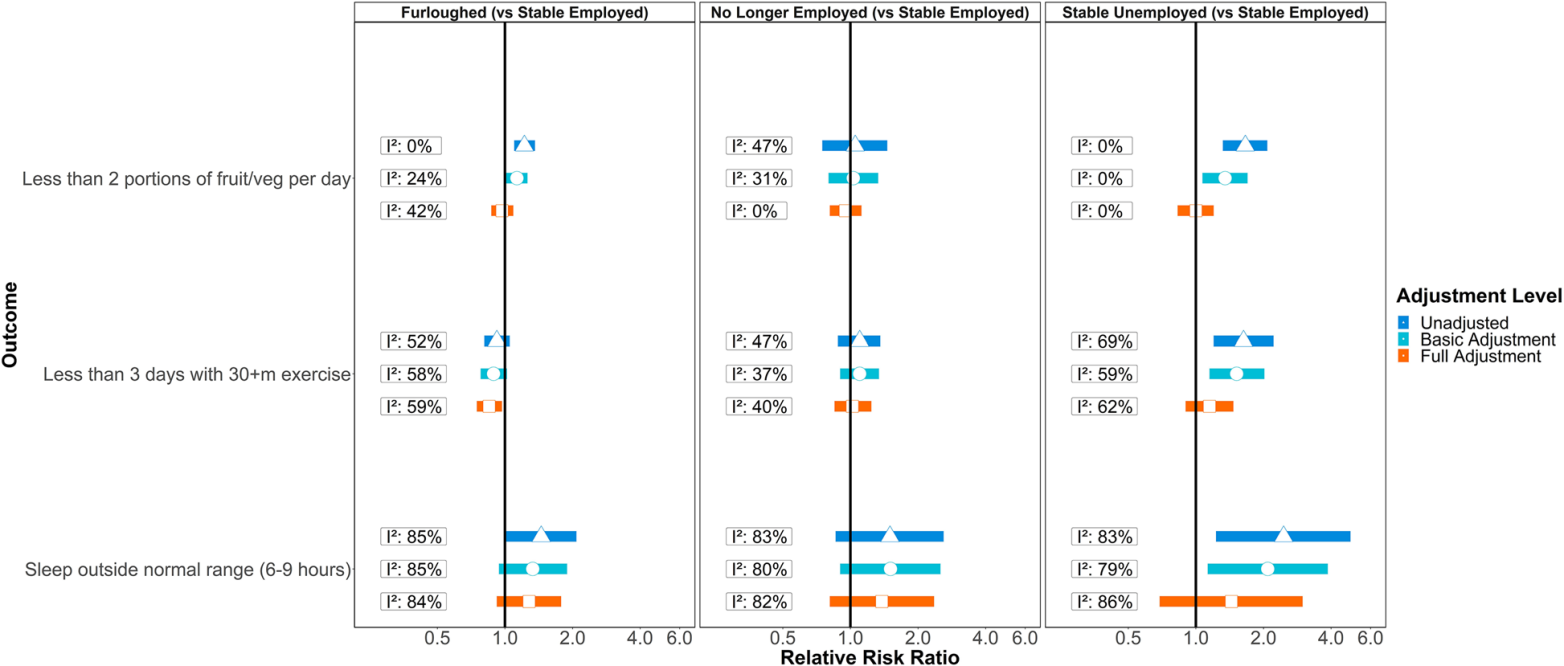
Associations between economic activity and health behaviours in pooled analyses across eight UK longitudinal studies. ‘Basic’ adjustment includes age, sex, ethnicity, education, UK nation, and household composition. ‘Full’ adjustment additionally includes pre-pandemic measures of mental health, self-rated health, diet, exercise, and sleep

**Fig. 3:**
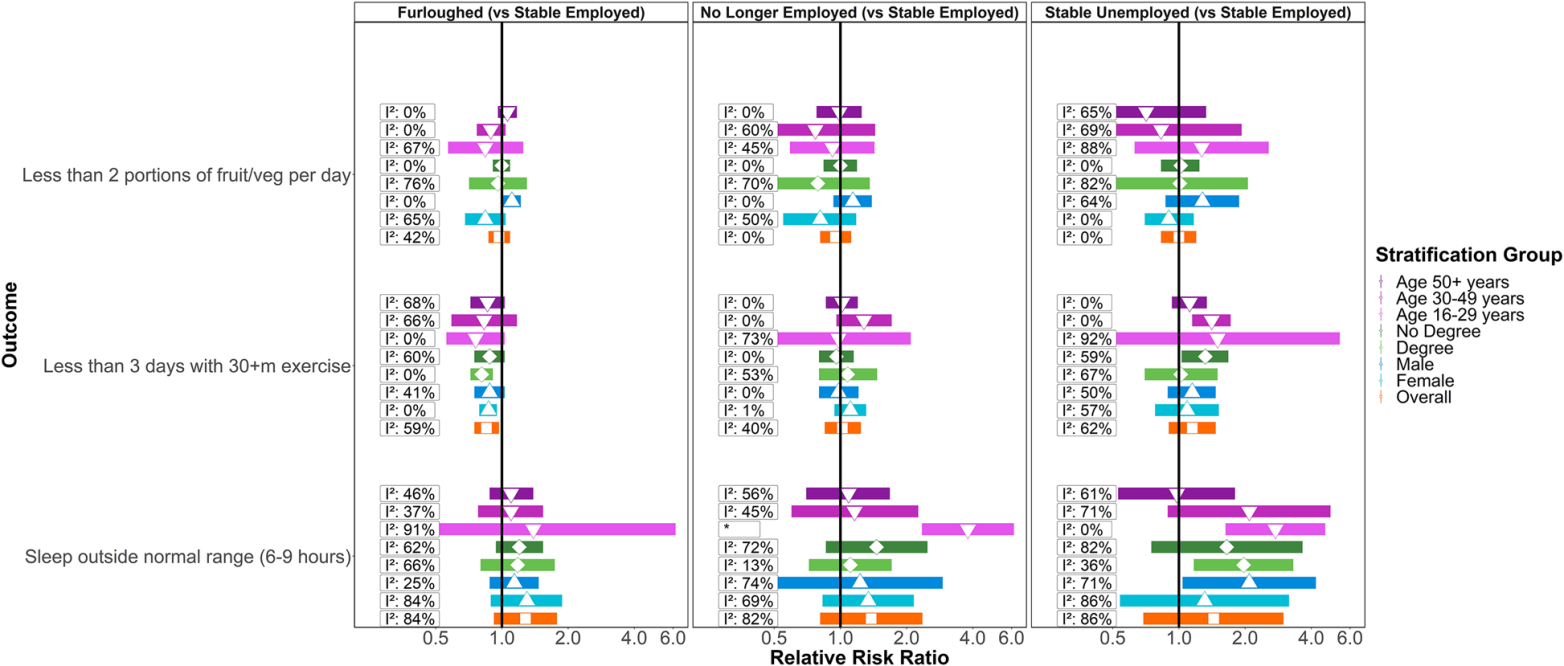
Associations between economic activity and health behaviours stratified by age, sex, and educational attainment. *No *I* ^*2*^ value as only one study was able to provide an estimate

Figure 2 shows meta-analysis estimates from unadjusted, basic adjusted, and fully adjusted models for levels of fruit and vegetable consumption, physical activity, and sleep during the pandemic. Given our primary interest is in investigating health behaviours of those furloughed, no longer employed, and stable unemployed compared to those in stable employment, we only present results for these groups (omitting those who became employed or were in stable non-employment). Figure 3 shows pooled estimates from fully adjusted models stratified by sex, education, and age. Stratified estimates were largely consistent with the main results, though we highlight some differences below. Full details of the meta-analysis including overall and stratified estimates from each study are available in Additional files 3 and 4.

#### Fruit and vegetable consumption

Unadjusted estimates suggest lower fruit and vegetable consumption during the pandemic among those furloughed or in stable unemployment compared to those who remained employed. These differences were robust to the basic adjustment, but were attenuated with full adjustment for pre-pandemic characteristics, suggesting that these associations are attributable to differences in dietary habits established prior to the pandemic. We observed moderate heterogeneity in the fully adjusted furlough model (*I* ^2^ = 42%). When looking at individual studies, only associations in MCS, where participants were 18–19 years old, remained after full adjustment (*RR* = 0.68; [95% *CI* 0.49 to 0.69]; 9% of the overall estimate) (see Additional file 3). There were no clear overall differences in fruit and vegetable consumption between those in stable employment and those who were no longer employed during the pandemic.

The association between furlough and fruit and vegetable consumption differed by gender (*p* = 0.02). Males who were furloughed were more likely to consume less fruit and vegetables during the pandemic than males who remained employed (*RR* = 1.11; [1.01–1.22]; *I* ^2^ = 0%). This association was not observed among furloughed females (*RR* = 0.84; [0.68–1.04]), although there was heterogeneity (*I* ^2^ = 65%) and furloughed females from MCS (again, the clearest outlier) were less likely to have low fruit and vegetable consumption than MCS females remaining employed (*RR* = 0.51; [0.34–0.77]). We did not observe differences by education or age (see Supplementary file 4).

#### Physical activity

Compared to stable employment, furlough was associated with lower risk of infrequent physical activity in fully adjusted models. In contrast, estimates for those no longer employed or in stable unemployment were in the opposite direction, although confidence intervals included the null. There was little evidence of subgroup differences in these associations.

#### Sleep

All three groups, furloughed, no longer employed, and stable unemployment, were more likely than those in stable employment to have atypical sleep. These associations were partly attenuated in the basic adjustment and further attenuated in the full adjustment models, so were at least partially accounted for by pre-pandemic characteristics and behaviours. Estimates for sleep exhibited high heterogeneity with *I* ^2^ values largely over 80%, perhaps partly due to age differences between the samples (see below).

The heightened risk of atypical sleep for those not in stable employment appeared to be largely concentrated at younger ages. For example, stable unemployment was associated with an *RR* of 2.75 ([95% *CI*: 1.63–4.63]; *I* ^2^ = 0%) in the 16–29 year age group, compared with 0.98 ([0.53–1.80]; *I* ^2^ = 61%) in the 50+ age group (*p* = 0.04). Age patterning was similar for those no longer employed (*p* < 0.01), but considerably less pronounced for furlough (*p* = 0.96). Thus, in this youngest age group, even after adjusting for pre-pandemic characteristics, there was evidence that atypical sleep was associated with stable unemployment (see above for RR) or being no longer employed (*RR* = 3.80; [2.35–6.15]; single estimate from MCS), but there was not a clear association with furlough (*RR* = 1.39; [0.31–6.16]; *I* ^2^ = 91%). However, the two studies that had provided estimates for furlough in this age group showed very different findings (raised risk in MCS but lower risk in USOC).

#### Changes in behaviour

Pooled estimates for the outcomes indicating change in behaviour are presented in Additional file 1: Tables S5, S6 and S7. These analyses indicated that furlough was associated with increases in fruit and vegetable consumption (*RR* = 1.22; [1.04–1.43]; *I* ^2^ = 52%), time spent exercising (*RR* = 1.18; [1.04–1.35]; *I* ^2^ = 75%), and hours of sleep (*RR* = 1.62; [1.39–1.90]; *I* ^2^ = 80%) relative to stable employment. Furlough was also associated with a higher likelihood of shifts both into and out of the typical 6–9 h sleep range, which is probably due to the strong association with increased hours of sleep (which was present in all stratified analyses). These associations were robust to adjustment for other pre-pandemic characteristics, though, by the nature of change outcomes, may still partially represent pre-pandemic differences in each behaviour. Largely similar patterns were seen for sleep among those no longer employed or in stable employment and for physical activity among those no longer employed.

## Discussion

We find little evidence that furlough was associated with worse health behaviours. Those who were furloughed did not differ with respect to risk of low fruit and vegetable consumption or atypical sleep and had a lower likelihood of infrequent exercise compared to those who remained employed. Stratified analyses showed that furloughed men, but not women, had a higher likelihood of low fruit and vegetable consumption than those who remained employed. Those who remained unemployed had worse health behaviours relative to the stable employed, although these differences were largely due to pre-pandemic behaviours. Among 16–29-year-olds who were no longer employed or remained unemployed, there was a higher risk of atypical asleep.

Previous studies on subsidised employment policies have shown beneficial effects [35]. Evidence from Sweden [36] shows that individuals in subsidised employment occupied an intermediate position in terms of subjective well-being; they were better-off than unemployed individuals, but worse-off than those in regular employment. Here, we only observed minor differences between those furloughed and those in stable employment, which may be due to the nature of the CJRS scheme, the timing of the surveys, and/or differences in the outcomes studied. Studies conducted since the COVID-19 pandemic have shown that health behaviours improved for some people while declining for others [37, 38], but our findings offer little evidence of furlough contributing to declines in healthy behaviour.

Unemployment has been shown to have detrimental effects on population health through various pathways including health-related behaviours [39–41]. These health effects may be modified by the type of welfare state regime in place and related social protection policies [42]. Employment is generally associated with good health [43], while job loss or unemployment is associated with deleterious health outcomes [44], especially among men and those in their early and middle careers [43]. While we observed similar findings for those unemployed prior and during the pandemic, we did not replicate detrimental impacts of job loss. However, participation in the furlough scheme was common, while participants who were no longer working during the initial stages of the pandemic were rare (~3%) leading to low precision in estimates for this group. The lack of strong evidence for the detrimental impacts on health behaviours that are normally associated with job loss may suggest these impacts are lessened or non-existent in the unique context of a pandemic.

While research combining results from several UK prospective studies — in their totality representative of the UK population — makes a clear contribution to understanding the impact of the furlough scheme, there are limitations that should be taken into account while interpreting our findings. Firstly, we were not able to achieve full harmonisation of measures across studies. By focusing on available comparable measures, we also limited our ability to explore other aspects of diet, physical activity, or sleep (such as frequency of snacking, specific kinds of physical activity, sleep quality, or daytime napping). For example, it remains unclear how intake of high-energy processed foods may have been associated with furlough during the pandemic. Furthermore, outcomes were only analysed during the initial stages of the pandemic (April–July 2020) and relationships may have altered with subsequent changes to restrictions, growing economic uncertainty, or in different seasons where UK weather is less conducive to outdoor exercise and leisure. However, exposure groups were observed over the same period, so seasonality would not introduce bias, only potentially affect generalisability. Further research is needed to examine this as well as heterogeneity in the stable employed and furloughed groups in greater detail. The MCS cohort particularly, who were the youngest cohort studied, often had considerably different estimates from the older aged respondents in other cohorts, so there may be different mechanisms affecting this age group.

Despite being embedded in long-standing studies, surveys during the pandemic were selective. We corrected most studies to being representative of their target population using weights derived for each study based on pre-pandemic information (and the GS study which did not have weights available exhibited similar estimates to the more nationally representative studies). Nevertheless, bias due to selective non-response cannot be excluded [45], especially as most studies (USOC being the exception) were weighted for non-response to COVID surveys but not for any residual non-response to the outcomes in question (among those who did participate in the overall COVID surveys). Similarly, bias due to unmeasured confounding cannot be ruled out and could be influential considering the small magnitude of the risk ratios observed. For example, there may be unobserved differences between participants who retained their jobs compared with those who experienced furlough or job loss. Our fully adjusted models account for differences in some key pre-pandemic characteristics among employment groups. However, it is possible that our results reflect other traits of these employment groups, for example, how workers in different industries or occupational classes responded to the pandemic, rather than being effects of furlough specifically. Adjustment for pre-pandemic characteristics may also have induced bias if there were unobserved determinants of both pre-pandemic characteristics and behaviour during the pandemic. However, we observed only minor differences between those furloughed and those who remained employed; therefore, any bias due to unmeasured confounding is unlikely to change the interpretation of our findings.

Our analyses on outcomes of change in behaviour during the pandemic showed some differences from the main analyses. Specifically, they indicated that being furloughed was associated with increases in fruit and vegetable consumption, hours of sleep, and time spent exercising relative to those maintaining stable employment. There may be several reasons for this: the change analyses included more studies, which implies a greater variability in measurement; these outcomes could have been picking up relatively minor changes in behaviour above or below the thresholds used in the main analyses; or they could be reflecting effects of initial employment status on the pre-pandemic diet, physical activity, or sleep.

## Conclusions

Despite the economic disruption of the pandemic and lockdown, participants who were no longer working during the initial stages of the pandemic were rare, while much higher proportions participated in the UK CJR furlough scheme. We found that those who were furloughed exhibited broadly similar levels of health behaviours to those who remained in employment and there was some evidence of more frequent exercise. Continuation of the UK furlough scheme has the potential to mitigate some of the adverse consequences of the pandemic and there was little evidence for detrimental impacts on population health behaviours. Our findings suggest that the UK furlough scheme may be an important component of policies aiming to mitigate the detrimental effects of economic downturns and prevent exacerbation of inequalities.

## Supplementary Information

**Additional file 1: Table S1.** Description of Studies. **Table S2.** Ethics and data access statements for each study. **Table S3.** Sample characteristics by study. **Table S4.** Employment status change by gender, education, and age-group. **Table S5.** Meta-analysed risk ratios and heterogeneity estimates for associations between changes in employment status and fruit and vegetable consumption: unadjusted, basic & full adjustment results. **Table S6.** Meta-analysed risk ratios and heterogeneity estimates for associations between changes in employment status and physical activity: unadjusted, basic & full adjustment results. **Table S7.** Meta-analysed risk ratios and heterogeneity estimates for associations between changes in employment status and sleep: unadjusted, basic & full adjustment results. **Figure S8.** Causal pathways blocked under differing levels of adjustment.

**Additional file 2.** Variable Coding. Contents: Full details of the questions and coding used for each variable and each cohort.

**Additional file 3.** Meta-Analysis; Table 1-6; Figure set 1-set 11. Contents: Table 1. Main analysis excluding studies with ≤5 cell counts for exposure-outcome. Table 2. Main analysis excluding studies with ≤2 cell counts for exposure-outcome. Table 3. Main analysis excluding studies with zero cell counts for exposure-outcome. Table 4. Analysis of change excluding studies with ≤ 5 cell counts. Table 5. Analysis of change excluding studies with ≤ 2 cell counts. Table 6. Analysis of change excluding studies with zero cell counts. Figure set 1: Currently eats 2 or fewer fruit & veg. Figure set 2: Fewer fruit and vegetables. Figure set 3: More fruit and vegetables. Figure set 4: Less than 3 days a week of at least 30 min exercise. Figure set 5: Less time/fewer days of physical exercise. Figure set 6: More time/days of physical exercise. Figure set 7: Sleeps outside ‘Normal Range’ (i.e. <6 or 9+ hours). Figure set 8: Sleeps less than before. Figure set 9: Sleeps more than before. Figure set 10: From 6/9 hours a night to outside ‘normal range’. Figure set 11: From outside ‘normal range’ to 6/9h a night.

**Additional file 4.** Stratified Meta-Analysis; Results stratified by Age (Figure set 1 - set 11); Education (set 12 - set 22); Sex (set 23 - set 33). Contents: Figure set 1; 11; 23: Currently eats 2 or fewer fruit & veg. Figure set 2; 12; 24: Fewer fruit & veg. Figure set 3; 13; 25: More fruit & veg. Figure set 4; 14; 26: Less than 3 days a week of at least 30min exercise. Figure set 5; 15; 27: Less time/ fewer days of physical exercise. Figure set 6; 16; 28: More time/ days of physical exercise. Figure set 7, 17; 29: Sleeps outside ‘Normal Range’ (i.e. <6 or 9+ hours). Figure set 8, 18, 30: Sleeps less than before. Figure set 9,19, 31: Sleeps less than before. Figure set 10, 20, 32: From 6/9h a night to outside ‘normal range’. Figure set 11, 12, 33: From outside ‘normal range’ to 6/9h a night.

## Data Availability

All datasets included in this analysis have established data sharing processes, and for most included studies, the anonymised datasets with corresponding documentation can be downloaded for use by researchers from the UK Data Service. We have detailed the processes for each dataset in Additional file 1: Table S2.

## Abbreviations

ALSPAC-G0: Parents of ALSPAC-G1
ALSPAC-G1: Avon Longitudinal Study of Parents and Children
BCS70: 1970 British Cohort Study
CI: Confidence interval
CJRS: Coronavirus Job Retention Scheme
ELSA: English Longitudinal Study of Ageing
GS: Generation Scotland: the Scottish Family Health Study
MCS: Millennium Cohort Study
NCDS: 1958 National Child Development Study
NS: Next Steps (formerly the Longitudinal Study of Young People in England)
RR: Risk ratio
UK: United Kingdom
USOC: Understanding Society
