# Supplementary material for: The UK Coronavirus Job Retention Scheme and diet, physical activity, and sleep during the COVID-19 pandemic: evidence from eight longitudinal population surveys
Source: BMC Med. 2022 Apr 6;20:147. doi: 10.1186/s12916-022-02343-y (PMC8984671; doi:10.1186/s12916-022-02343-y)
Supplement: Supplementary file 2 — Additional file 2. Variable Coding. Contents: Full details of the questions and coding used for each variable and each cohort. [file 12916_2022_2343_MOESM2_ESM.pdf]

Study	Question (exact wording)	Possible Answers	Recoding
*** CHANGE in EMPLOYMENT STATUS ***			
	**1='Stable employed'; 2='Furloughed'; 3='Became employed'; 4='No longer employed'; 5='Stable unemployed'; 6='Stable in other category'**		
**MCS**<br>**NS**<br>**BCS 70**<br>**NCDS**	Q1: Which of these best describes what you were doing just before the Coronavirus outbreak in March? If you were doing more than one activity, please choose the activity that you spent most time doing.<br>Q2: Which of these would you say best describes your situation now?	Q1. 1=Employed; 2=Self-employed; 3=In unpaid/ voluntary work; 4=Apprenticeship; 5=Unemployed; 6=Permanently sick or disabled; 7=Looking after home or family; 8=In education at school/college/university; 9=Retired; 10=Doing something else.<br>Q2. 1=Employed and currently working (or on annual leave/holiday); 2=Employed but on paid leave (including furlough); 3=Employed and on unpaid leave; 4=Apprenticeship; 5=In unpaid/voluntary work; 6=Self-employed and currently working (or on holiday); 7=Self-employed but not currently working; 8=Unemployed; 9=Permanently sick or disabled; 10=Looking after home or family; 11=In education at school/college/university; 12=Retired; 13=Doing something else.<br>Q3. 1=There was no interruption to learning activities 2. I took a break from learning activities 3. I was studying at home with online resources provided by my learning establishment 4. I was studying at home with no online resources provided by my learning establishment 5. My course finished earlier than planned 6. I dropped out from learning activities	1= if (Q1 = 1) & (Q2 = 1 OR 6)<br>2= if (Q1 = 1) & (Q2 = 2 OR 7)<br>3= if (Q1 != 1 OR 5) & (Q2 = 1 OR 6)<br>4= if (Q1 = 1) & (Q2 != 1 OR 6)<br>5= if (Q1 = 5) & (Q2 = 8)<br>6= if (Q1 = != 1 OR 5) & (Q2 != 1 OR 2 OR 6 OR 7 OR 8)"
**ALSPAC**	Q1. Just before the lockdown on the 23rd March 2020, were you?<br>Q2. Which of these would you say best describes your current situation now?	Q1. 1=In full time paid work (30 or more hours a week); 2=In part-time paid work (less than 30 hours a week); 3=In irregular or occasional work; 4=Doing a modern apprenticeship or other government supported training/work-experience scheme; 5=Unemployed and looking for work; 6=Unable to work through sickness/disability; 7=In full-time education; 8=In part-time education; 9=Doing voluntary work; 10=Self-employed; 11=A full/part time carer; 12=Retired; 13=Other.<br>Q2. 1=Employed and working same number of hours as pre-lockdown; 2=Employed and working reduced number of hours; 3=Employed and working more hours than before; 4=Employed but on paid leave (including furlough); 5=Employed and on unpaid leave; 6=Apprenticeship; 7=In unpaid/voluntary work; 8=Self-employed and currently working; 9=self-employed but not currently working; 10=Unemployed; 11=Permanently Sick/Disabled; 12=looking after home or family;13=In education at school/college/university	1=if(Q1=1/3) & (Q2= 1/3 OR 8)<br>2=if(Q1=1/3) & (Q2=4)<br>3=if(Q1!=1/3) & (Q2 = 1/3 OR 8)<br>4=if(Q1=1/3) & (Q2 = 5/7 OR 9/13)<br>5=if(Q1=5) & (Q2 = 10)<br>6=if(Q1!=1/3) & (Q2 = 5/7 OR 9/13)
**USOC**	Q1: Thinking back to earlier this year, before the outbreak of the coronavirus pandemic. Were you in paid work or self-employment at any time in January or February 2020?<br>Q2: Thinking about your situation now. Even if you did not do any paid work last week, are you currently employed or self-employed?<br>Q3: Have you received a written letter or email from your employer to confirm that you have been furloughed under the Coronavirus Job Retention Scheme?<br>Q4: (asked pre-pandemic) Which of these best describes your current employment situation?	Q1: 1. Yes, employed; 2. Yes, self-employed; 3. Yes, both employed and self-employed; 4. No.<br>Q2: 1. Yes, employed; 2. Yes, self-employed; 3. Yes, both employed and self-employed; 4. No.<br>Q3: 1. Yes; 2. No.<br>Q4: 1. Self employed; 2. Paid employment; 3. Unemployed; 4. Retired; 5. On maternity leave; 6. Family care or home; 7. Full-time student; 8. LT sick or disabled; 9. Govt training scheme; 10. Unpaid, family business; 11. On apprenticeship. 97. Doing something else.	1=if (Q1<=3) & (Q2<=3)<br>2=if (Q1<=3) & Q3=1<br>3=if (Q1=4) & (Q2<=3)<br>4=if (Q1<=3) & (Q2=4)<br>5=if (Q1=4) & (Q2=4) & (Q4=3)<br>6=if (Q1=4) & (Q2=4) & (Q4!=3)
**ELSA**	Q1: Which of these best describes what you were doing just before the coronavirus outbreak?<br>Q2: And which of these would you say best describes your current situation?	Q1. 1=Retired; 2=Employed; 3=Self-employed; 4=Unemployed; 5=Permanently sick or disabled; 6=Looking after home or family<br>Q2. 1=Retired; 2=Employed; 3=Paid/unpaid leave from employment (including furlough); 4=Self-employed and currently working; 5=Self-employed but not currently working; 6=Unemployed; 7=Permanently sick or disabled; 8=Looking after home or family.	1= if (Q1=2 OR 3) & (Q2=2 OR 4)<br>2= if (Q1=1 OR 2 OR 3) & (Q2=3 OR 5)<br>3= if (Q1=1 OR >=4) & (Q2=2 OR =4)<br>4= if (Q1=2 OR 3) & (Q2>=6 OR Q2==1)<br>5= if Q1=4 & Q2=6<br>6= if (Q1=1 & Q2=1) OR (Q1>=4 & (Q2=1 OR 3 OR 5 OR 7 OR 8)) OR (Q1=1 & Q2>=7)
**GS**	Q1.What was your employment status just before the COVID-19 measures were introduced (i.e. January 2020)?<br>Q2. What is your employment status now?<br>Q3. Have any of the following happened to you due to COVID-19 measures?	Q1 and Q2. 1=Self-employed employing others; 2=Self-employed not employing others; 3=Paid employee supervising others; 4=Paid employee not supervising others; 5=In unpaid employment; 6=Homemaker; 7=Looking after children; 8=Looking after other dependents; 9=Retired; 10=Still in school/studying full-time; 11=Unemployed as sick or disabled; 12=Unemployed; 13=Other<br>Q3. 1=Yes; 0=NA	1= if Q1<=4 & Q2<=4<br>2= if Q3=1<br>3= if Q1>=5 & Q2<=4<br>4= if Q1<=4 & Q2 >= 5<br>5= if Q1=12 & Q2=12<br>6= if (Q1>=5 & Q1<=11|Q1==13) & ((Q2>=5 & Q2<=11)|Q2==13)

Study	Question (exact wording)	Possible Answers	Recoding
*** CURRENT DIET * -- 1= Currently eats 2 or fewer portions of fruit and vegetables**			
**MCS**<br>**NS**<br>**BCS 70**<br>**NCDS**	Since the start of the Coronavirus outbreak, how many portions of fresh fruit and vegetables have you eaten in a typical day?	0 to 150	<=2
**ALSPAC**	Not Available		
**USOC**	[Asked in July]<br>Q1: Thinking about last week, including tinned, frozen, dried and fresh fruit, on how many days did you eat fruit? Please do not include fruit juice.<br>Q2: On the days when you eat fruit, how many portions (e.g. an apple, an orange, some grapes) do you eat?<br>Q3: Thinking about last week, including tinned, frozen and fresh vegetables, on how many days did you eat vegetables? Do not include potatoes, crisps or chips.<br>Q4: On the days when you eat vegetables, how many portions (i.e. 3 heaped tablespoons) do you eat? Please do not include potatoes.	Q1: 1. Never; 2. 1-3 Days; 3. 4-6 Days; 4. Every day.<br>Q2: value>=0<br>Q3: 1. Never; 2. 1-3 Days; 3. 4-6 Days; 4. Every day.<br>Q4: value>=0	Average daily portions of fruit/veg calculated from Q1-4 is <2
**ELSA**	Not Available		
**GS**	Not Available		
*** CHANGES IN DIET * -- 1= Eats more portions of fruit and vegetables**		**u**	
**MCS**<br>**NS**<br>**BCS 70**<br>**NCDS**	Q1: In the month before the start of the Coronavirus outbreak, how many portions of fresh fruit and vegetables did you eat in a typical day?<br>Q2: Since the start of the Coronavirus outbreak, how many portions of fresh fruit and vegetables have you eaten in a typical day?	Q1 & Q2: 0-150	Q2>Q1
**ALSPAC**	Not Available		
**USOC**	[Asked pre-pandemic]<br>Q1: Including tinned, frozen, dried and fresh fruit, on how many days in a usual week do you eat fruit?<br>Q2: On the days when you eat fruit, how many portions (e.g. an apple, an orange, some grapes) do you eat?<br>Q3: Including tinned, frozen and fresh vegetables, on how many days in a usual week do you eat vegetables? Do not include potatoes, crisps or chips.<br>Q4: On the days when you eat vegetables, how many portions (i.e. 3 heaped tablespoons) do you eat? Please do not include potatoes.	Q1: 1. Never; 2. 1-3 Days; 3. 4-6 Days; 4. Every day.<br>Q2: value>=0<br>Q3: 1. Never; 2. 1-3 Days; 3. 4-6 Days; 4. Every day.<br>Q4: value>=0	Average daily portions of fruit/vegetables calculated from Q1-4 is < Average daily portions calculated for current diet above.
**ELSA**	Not Available		
**GS**	Not Available		

Study	Question (exact wording)	Possible Answers	Recoding
*** CHANGES IN DIET * -- 1= Eats fewer portions of fruit and vegetables**			
**MCS**<br>**NS**<br>**BCS 70**<br>**NCDS**	Q1:In the month before the start of the Coronavirus outbreak, how many portions of fresh fruit and vegetables did you eat in a typical day?<br>Q2: Since the start of the Coronavirus outbreak, how many portions of fresh fruit and vegetables have you eaten in a typical day?	Q1 & Q2: 0-150	Q2<Q1
**ALSPAC**	Not Available		
**USOC**	[Asked pre-pandemic]<br>Q1: Including tinned, frozen, dried and fresh fruit, on how many days in a usual week do you eat fruit?<br>Q2: On the days when you eat fruit, how many portions (e.g. an apple, an orange, some grapes) do you eat?<br>Q3: Including tinned, frozen and fresh vegetables, on how many days in a usual week do you eat vegetables? Do not include potatoes, crisps or chips.<br>Q4: On the days when you eat vegetables, how many portions (i.e. 3 heaped tablespoons) do you eat? Please do not include potatoes.	Q1: 1. Never; 2. 1-3 Days; 3. 4-6 Days; 4. Every day.<br>Q2: value>=0<br>Q3: 1. Never; 2. 1-3 Days; 3. 4-6 Days; 4. Every day.<br>Q4: value>=0	Average daily portions of fruit/vegetables calculated from Q1-4 is > Average daily portions calculated for current diet above.
**ELSA**	Not Available		
**GS**	Not Available		

Study	Question (exact wording)	Possible Answers	Recoding
*** CURRENT PHYSICAL ACTIVITY * -- 1= Less than 3 days a week of at least 30min exercise**			
**MCS**<br>**NS**<br>**BCS 70**<br>**NCDS**	Since the start of the Coronavirus outbreak, on how many days in a typical week did you do 30 minutes or more of exercise where you are working hard enough to raise your heart rate and break into a sweat?	0 to 7	<=2
**ALSPAC**	Not Available		
**USOC**	Q1: Think about all the vigorous activities that you did in the last 7 days. Vigorous activities refer to activities that take hard physical effort and make you breathe much harder than normal. Think only about those physical activities that you did for at least 10 minutes at a time. During the last 7 days, on how many days did you do vigorous physical activities like heavy lifting, digging, aerobics, or fast bicycling?<br>Q2: How much time did you usually spend doing vigorous physical activities on one of those days?<br>Q3: Think about all the moderate physical activities that you did in the last 7 days. Moderate activities refer to activities that take moderate physical effort and make you breathe somewhat harder than normal. Think only about those activities that you did for at least 10 minutes at a time. During the last 7 days, on how many days did you do moderate physical activities like carrying light loads, bicycling at a regular pace, or doubles tennis? Do not include walking<br>Q4: How much time did you usually spend doing moderate physical activities on one of those days?<br>Q5: Now think about the time you spent walking in the last 7 days. This includes at work and at home, walking to travel from place to place, and any other walking that you might do solely for recreation, sport, exercise, or leisure. During the last 7 days, on how many days did you walk for at least 10 minutes at a time?<br>Q6: How much time did you usually spend walking on 1 of those days?	Q1,Q3,Q5: 0-7 days.<br>Q2,Q4,Q6: Time spent in hours/mins.	The total days per week with >=30 mins of either vigorous, moderate or walking activity was <3.
**ELSA**	Not Available		
**GS**	Not Available		

Study	Question (exact wording)	Possible Answers	Recoding
*** CHANGES IN PHYSICAL ACTIVITY * -- 1= More time/days of physical exercise**			
**MCS<br>NS<br>BCS 70<br>NCDS**	Q1: In the month before the start of the Coronavirus outbreak, on how many days in a typical week did you do 30 minutes or more of exercise where you are working hard enough to raise your heart rate and break into a sweat?<br>Q2: Since the start of the Coronavirus outbreak, on how many days in a typical week did you do 30 minutes or more of exercise where you are working hard enough to raise your heart rate and break into a sweat?	Q1 & Q2: 0-7	Q2>Q1
**ALSPAC**	Since lockdown, have any of the following aspects of your life changed? "Amount of physical activity/exercise you do"	1=Decreased a lot; 2=Decreased a little; 3=Stayed the same; 4=Increased a little; 5=Increased a lot; 6=Not applicable	4/5=1, else =0
**USOC**	Q1: Think about all the vigorous activities that you did in the last 7 days. Vigorous activities refer to activities that take hard physical effort and make you breathe much harder than normal. Think only about those physical activities that you did for at least 10 minutes at a time. During the last 7 days, on how many days did you do vigorous physical activities like heavy lifting, digging, aerobics, or fast bicycling?<br>Q2: How much time did you usually spend doing vigorous physical activities on one of those days?<br>Q3: Think about all the moderate activities that you did in the last 7 days. Moderate activities refer to activities that take moderate physical effort and make you breathe somewhat harder than normal. Think only about those physical activities that you did for at least 10 minutes at a time. During the last 7 days, on how many days did you do moderate physical activities like carrying light loads, bicycling at a regular pace, or doubles tennis? Do not include walking<br>Q4: How much time did you usually spend doing moderate physical activities on one of those days?<br>Q5: Now think about the time you spent walking in the last 7 days. This includes at work and at home, walking to travel from place to place, and any other walking that you might do solely for recreation, sport, exercise, or leisure. During the last 7 days, on how many days did you walk for at least 10 minutes at a time?<br>Q6: How much time did you usually spend walking on one of those days?	Q1,Q3,Q5: 0-7 days.<br>Q2,Q4,Q6: Time spent in hours/mins.	Total time reported doing either vigorous, moderate or walking activity in Q1-6 is less than total time reported during pandemic (see above).
**ELSA**	Since the coronavirus outbreak began in February, please say whether you have been doing physical activity less than usual, about the same, or more than usual	1=Less than usual; 2=About the same; 3=More than usual	=3
**GS**	Q1. How many days did you do moderate activity for at least 10 minutes at a time: (a) in the last 7 days?; (b) in a typical week before COVID-19 measures were introduced (i.e., January 2020)?<br>Q2. How many days did you do vigorous activity for at least 10 minutes at a time: (a) in the last 7 days?; (b) in a typical week before COVID-19 measures were introduced (i.e., January 2020)?	Q1(a and b) & Q2(a and b): 0-7	MaxValue(Q1b OR Q2b) - MaxValue(Q1a OR Q2a) < 0

Study	Question (exact wording)	Possible Answers	Recoding
*** CHANGES IN PHYSICAL ACTIVITY * -- 1= Less time/Fewer days of physical exercise**			
**MCS<br>NS<br>BCS 70<br>NCDS**	Q1: In the month before the start of the Coronavirus outbreak, on how many days in a typical week did you do 30 minutes or more of exercise where you are working hard enough to raise your heart rate and break into a sweat?<br>Q2: Since the start of the Coronavirus outbreak, on how many days in a typical week did you do 30 minutes or more of exercise where you are working hard enough to raise your heart rate and break into a sweat?	Q1 & Q2: 0-7	Q2<Q1
**ALSPAC**	Since lockdown, have any of the following aspects of your life changed? "Amount of physical activity/exercise you do"	1=Decreased a lot; 2=Decreased a little; 3=Stayed the same; 4=Increased a little; 5=Increased a lot; 6=Not applicable	1/2=1, else =0
**USOC**	Q1: Think about all the vigorous activities that you did in the last 7 days. Vigorous activities refer to activities that take hard physical effort and make you breathe much harder than normal. Think only about those physical activities that you did for at least 10 minutes at a time. During the last 7 days, on how many days did you do vigorous physical activities like heavy lifting, digging, aerobics, or fast bicycling?<br>Q2: How much time did you usually spend doing vigorous physical activities on one of those days?<br>Q3: Think about all the moderate activities that you did in the last 7 days. Moderate activities refer to activities that take moderate physical effort and make you breathe somewhat harder than normal. Think only about those physical activities that you did for at least 10 minutes at a time. During the last 7 days, on how many days did you do moderate physical activities like carrying light loads, bicycling at a regular pace, or doubles tennis? Do not include walking<br>Q4: How much time did you usually spend doing moderate physical activities on one of those days?<br>Q5: Now think about the time you spent walking in the last 7 days. This includes at work and at home, walking to travel from place to place, and any other walking that you might do solely for recreation, sport, exercise, or leisure. During the last 7 days, on how many days did you walk for at least 10 minutes at a time?<br>Q6: How much time did you usually spend walking on one of those days?	Q1,Q3,Q5: 0-7 days.<br>Q2,Q4,Q6: Time spent in hours/mins.	Total time reported doing either vigorous, moderate or walking activity in Q1-6 is more than total time reported during pandemic (see above).
**ELSA**	Since the coronavirus outbreak began in February, please say whether you have been doing physical activity less than usual, about the same, or more than usual	1=Less than usual; 2=About the same; 3=More than usual	=1
**GS**	Q1. How many days did you do moderate activity for at least 10 minutes at a time: (a) in the last 7 days?; (b) in a typical week before COVID-19 measures were introduced (i.e., January 2020)?<br>Q2. How many days did you do vigorous activity for at least 10 minutes at a time: (a) in the last 7 days?; (b) in a typical week before COVID-19 measures were introduced (i.e., January 2020)?	Q1(a and b) & Q2(a and b): 0-7	MaxValue(Q1b OR Q2b) - MaxValue(Q1a OR Q2a) > 0

Study	Question (exact wording)	Possible Answers	Recoding
*** SLEEPING TIME * -- 1= Sleeps outside 'Normal Range' (i.e. <6 or 9+ hours)**			
**MCS**<br>**NS**<br>**BCS 70**<br>**NCDS**	Since the start of the Coronavirus outbreak, how many hours did you sleep each night on average?	=1-24	<6 OR >9
**ALSPAC**	Not Available		
**USOC**	(asked in July) How many hours of actual sleep did you usually get per night during the last month? This may be different than the number of hours you spent in bed.	=0-24	<6 OR >9
**ELSA**	Not Available		
**GS**	What was the average number of hours you slept per day in the last 7 days?	=0-24	<6 OR >9
*** CHANGE IN SLEEPING TIME * -- 1= From 6/9h a night to outside 'normal range'**			
**MCS**<br>**NS**<br>**BCS 70**<br>**NCDS**	Q1: In the month before the Coronavirus outbreak, how many hours did you sleep each night on average?<br>Q2: Since the start of the Coronavirus outbreak, how many hours did you sleep each night on average?	Q1 & Q2. 1-24	Q1=(6 to 9) & Q2(<6 OR >9)
**ALSPAC**	Not Available		
**USOC**	(Asked pre-pandemic) How many hours of actual sleep did you usually get per night during the last month? This may be different than the number of hours you spent in bed.	=0-24	>6 OR <9 and above variable indicating sleep outside normal range during the pandemic=1
**ELSA**	Not Available		
**GS**	Q1: What was the average number of hours you slept per day in the last 7 days?<br>Q2: What was the average number of hours you slept per day in a typical week before COVID-19 measures were introduced (i.e., January 2020?)	Q1 & Q2. 0-24	Q1=(6 to 9) & Q2(<6 OR >9)
*** CHANGE IN SLEEPING TIME * -- 1= From outside 'normal range' to 6/9h a night**			
**MCS**<br>**NS**<br>**BCS 70**<br>**NCDS**	Q1: In the month before the Coronavirus outbreak, how many hours did you sleep each night on average?<br>Q2: Since the start of the Coronavirus outbreak, how many hours did you sleep each night on average?	Q1 & Q2. 1-24	Q1=(<6 OR >9) & Q2(6 to 9)
**ALSPAC**	Not Available		
**USOC**	(asked pre-pandemic) How many hours of actual sleep did you usually get per night during the last month? This may be different than the number of hours you spent in bed.	=0-24	<6 OR >9 and above variable indicating sleep outside normal range during the pandemic=0
**ELSA**	Not Available		
**GS**	Q1: What was the average number of hours you slept per day in the last 7 days?<br>Q2: What was the average number of hours you slept per day in a typical week before COVID-19 measures were introduced (i.e., January 2020?)	Q1 & Q2. 0-24	Q1=(<6 OR >9) & Q2(6 to 9)

Study	Question (exact wording)	Possible Answers	Recoding
*** LESS SLEEP * -- 1= Sleep less than before**			
**MCS NS BCS 70 NCDS**	Q1: In the month before the Coronavirus outbreak, how many hours did you sleep each night on average? Q2: Since the start of the Coronavirus outbreak, how many hours did you sleep each night on average?	Q1 & Q2. 1-24	Q2<Q1
**ALSPAC**	Since lockdown, have any of the following aspects of your life changed? "Amount you sleep"	1=Decreased a lot; 2=Decreased a little; 3=Stayed the same; 4=Increased a little; 5=Increased a lot; 6=Not applicable	1/2=1, else =0
**USOC**	(asked pre-pandemic) How many hours of actual sleep did you usually get per night during the last month? This may be different than the number of hours you spent in bed.	=0-24	Total hours reported pre-pandemic is < total hours reported during the pandemic.
**ELSA**	Since the coronavirus outbreak began in February, please say whether you have been sleeping less than usual, about the same, or more than usual	1=Less than usual; 2=About the same; 3=More than usual	=1
**GS**	Q1: What was the average number of hours you slept per day in the last 7 days? Q2: What was the average number of hours you slept per day in a typical week before COVID-19 measures were introduced (i.e., January 2020?)	Q1 & Q2. 0-24	Q1<Q2
*** MORE SLEEP * -- 1= Sleep more than before**			
**MCS NS BCS 70 NCDS**	Q1: In the month before the Coronavirus outbreak, how many hours did you sleep each night on average? Q2: Since the start of the Coronavirus outbreak, how many hours did you sleep each night on average?	Q1 & Q2. 1-24	Q2>Q1
**ALSPAC**	Since lockdown, have any of the following aspects of your life changed? "Amount you sleep"	1=Decreased a lot; 2=Decreased a little; 3=Stayed the same; 4=Increased a little; 5=Increased a lot; 6=Not applicable	4/5=1, else =0
**USOC**	(asked pre-pandemic) How many hours of actual sleep did you usually get per night during the last month? This may be different than the number of hours you spent in bed.	=0-24	Total hours reported pre-pandemic is < total hours reported during the pandemic.
**ELSA**	Since the coronavirus outbreak began in February, please say whether you have been sleeping less than usual, about the same, or more than usual	1=Less than usual; 2=About the same; 3=More than usual	=3
**GS**	Q1: What was the average number of hours you slept per day in the last 7 days? Q2: What was the average number of hours you slept per day in a typical week before COVID-19 measures were introduced (i.e., January 2020?)	Q1 & Q2. 0-24	Q1>Q2

Study	Possible Answers	Recoding
*** Sex * 0=Male; 1=Female**		
**All**	0=Male; 1=Female	
*** Ethnicity * 0=White; 1=Ethnic Minority**		
**MCS**	1=White; 2=Mixed; 3=Indian; 4=Pakistani; 5=Bangladeshi; 6=Other Asian; 7=Black Caribbean; 8=Black African; 9=Other Black; 10=Chinese; 11=Other ethnic group	1=0; 2/11=1
**NS**	1=White; 2=Mixed; 3=Indian; 4=Pakistani; 5=Bangladeshi; 6=Black Caribbean; 7=Black African; 8=Other	1=0; 2/8=1
**BCS70**	Not Available	
**NCDS**	Not Available	
**ALSPAC**	G0 (Parents) 1=White; 2=Black carribean; 3=Black african; 4=Other black; 5=Indian; 6=Pakistani; 7=Bangladeshi; 8=Chinese; 9=Other<br>G1 (Children) 1=White; 2=Mixed/Multiple Ethnic group; 3=Asian; 4=Black/African/Caribbean/Black British; 5=Arab or Other	1=0; 2/9=1
**USOC**	1=White British; 2=Irish (White); 3=Gypsy or Irish Traveller (white); 4=Any other white background; 5=White and black caribbean (mixed); 6=White and black african (mixed); 7=White and Asian (mixed); 8=Any other mixed background; 9=Indian (Asian or Asian British); 10=Pakistani (Asian or Asian British); 11=Bangladeshi (Asian or Asian British); 12=Chinese (Asian or Asian British); 13=Any other Asian background (Asian or Asian British); 14=Caribbean (Black or Black British); 15=African (Black or Black British); 16=Any other Black background (Black or Black British); 17=Arab (other Ethnic group); 97=Any other ethnic group	1-4=0; 5-97=1
**ELSA**	1.White; 2=Mixed ethnic group; 3=Black; 4=Black British; 5=Asian; 6=Asian British	1=0; 2/6=1
**GS**	1=White Scottish; 2=White English; 3=White Welsh; 4=White N. Irish; 5=White Irish; 6=White Gypsy/Irish traveller; 7=White Polish; 8=Any other white; 9=Asian/British Asian - Indian; 10=Asian/British Asian - Pakistani; 11=Asian/British Asian - Bangladeshi; 12=Asian/British Asian - Chinese; 13=Any other Asian background; 14=Black or Black British - African; 15=Black or Black British - Carribean; 16=Any other Black/African/Caribbean background; 17=Arab or Arab British; 18=Mixed - White and Black Caribbean; 19=Mixed - White and Black African; 20=Mixed - White and Asian; 21=Any other Mixed/Multiple ethnic background; 22=Any other ethnic group	1-8=0; 9-22=1
*** Education * 0=No Degree; 1=Degree**		
**MCS**<br>**NS**<br>**BCS 70**<br>**NCDS**	0=None; 1=Nvq1; 2=Nvq2; 3=Nvq3; 4=Nvq4; 5=Nvq5<br>*parent's education for MCS	1/4=0; 5=1
**ALSPAC**	1=Degree; 2=A levels/AS levels or equivalent; 3=O levels; 4=Vocational; 5=CSE<br>*parent's education for G1 (Children)	2/5=0
**USOC**	1.Higher degree 2. 1st degree or equivalent 3. Diploma in Higher Education 4. Teaching qualification (not PGCE) 5. Nursing or other medical qualification 6. Other higher degree 7. A-Level 8. Welsh baccalaureate 9. International baccalaureate 10. AS Level 11. Scottish Highers 12. Certificate of 6th year studies 13. GCSE/O-Level 14. Certificate of secondary education 15. Standard or lower 16. Other school certificate 96. No qualifications	1/6=1; 7-96=0
**ELSA**	1=Nvq4/nvq5/degree or equivalent; 2=Higher Education below degree; 3=Nvq3/GCE A level equivalent; 4=Nvq2/GCE O level equivalent; 5=Nvq1/CSE other grade equivalent; 6=Foreign/other; 7=No qualification	2/7=0
**GS**	1=No qualifications; 2=Other (please specify); 3=School leavers certificate; 4=CSEs or equivalent; 5=Standard grade, National 4 or 5, O levels, GCSEs or equivalent; 6=Higher grade, A levels, AS levels or equivalent; 7=NVQ or HND or HNC or equivalent; 8=Other professional or technical qualification; 9=Undergraduate degree; 10=Postgraduate degree	1/8=0; 9 OR 10=1
*** Living Arrangements * 1=Alone; 2=With partner/spouse only; 3=With partner/spouse and child(ren); 4=With child(ren), without partner/spouse; 5=Any other living arrangement OR * Partnership Status * 1=Married/Partnered; 0=Not married/partnered**		
*** Pre-Pandemic Self-Assessed Health * 1=Good/Very Good/Excellent; 0=Fair/Poor**		
**MCS**<br>**NS**<br>**BCS 70**<br>**NCDS**	In general, in the 3 months before the Coronavirus outbreak would you say your health was … 1=Excellent; 2=Very Good; 3=Good; 4=Fair; 5=Poor	1/3=1; 4/5=0
**ALSPAC**	Derived: Prepandemic Asthma (0: No, 1: Yes) + Prepandemic Obesity (0: No, 1:Yes) + Prepandemic Diabetes (0: No, 1:Yes)	0=1; >1=0
**USOC**	In general, would you say your health is… 1=Excellent; 2=Very Good; 3=Good; 4=Fair; 5=Poor	1/3=1; 4/5=0
**ELSA**	Would you say your health is… 1=Excellent; 2=Very Good; 3=Good; 4=Fair; 5=Poor	1/3=1; 4/5=0
**GS**	Not available	

Study	Possible Answers	Recoding
*** Pre-Pandemic Mental Health * 1=High Psychological Distress; 0=No Psychological Distress**		
**MCS**	Kessler K6 measure of psychological distress [Kessler, R. C., Andrews, G., Colpe, L. J., Hiripi, E., Mroczek, D. K., Normand, S. L., ... & Zaslavsky, A. M. (2002). Short screening scales to monitor population prevalences and trends in non-specific psychological distress. Psychological medicine, 32(6), 959-976.]	=1 if 13+
**NS**	General Health Questionnaire GHQ-12 [Goldberg DP, Gater R, Sartorius N,et al. The validity of two versions of the GHQ in the WHO study of mental illness in general health care. Psychol Med 1997;27:191–7.]	=1 if 4+
**BCS70**	Malaise Inventory. [Rodgers, B., Pickles, A., Power, C., Collishaw, S., & Maughan, B. (1999). Validity of the Malaise Inventory in general population samples. Social psychiatry and psychiatric epidemiology, 34(6), 333-341.]	=1 if 4+
**NCDS**	Malaise Inventory. [Rodgers, B., Pickles, A., Power, C., Collishaw, S., & Maughan, B. (1999). Validity of the Malaise Inventory in general population samples. Social psychiatry and psychiatric epidemiology, 34(6), 333-341.]	=1 if 4+
**ALSPAC G1**	Short Mood and Feelings Questionnaire (SMFQ) [Angold, A., Costello, E.J., Messer, S.C., & Pickles, A. (1995). Development of a short questionnaire for use in epidemiological studies of depression in children and adolescents. International Journal of Methods in Psychiatric Research, 5, 237–249.]	=1 if 12+
**ALSPAC G0**	Edinburgh Postnatal Depression Scale EPDS [Matthey S, Barnett B, Kavanagh DJ, et al. Validation of the Edinburgh Postnatal Depression Scale for men, and comparison of item endorsement with their partners. Journal of Affective Disorders 2001;64(2-3):175-84.]	=1 if 11+
**USOC**	General Health Questionnaire GHQ-12 [Goldberg DP, Gater R, Sartorius N,et al. The validity of two versions of the GHQ in the WHO study of mental illness in general health care. Psychol Med 1997;27:191–7.]	=1 if 4+
**ELSA**	Eight-item version of the original CES-D (felt depressed; felt everything was an effort; restless sleep; not happy; felt lonely; not enjoyed life; felt sad; could not get going) [Radloff LS. The CES-D Scale: A Self-Report Depression Scale for Research in the General Population. Applied Psychological Measurement. 1977;1(3):385-401]	=1 if 4 or more symptoms reported
**GS**	General Health Questionnaire GHQ-28 [Goldberg DP, Gater R, Sartorius N,et al.The validity of two versions of the GHQ in the WHO study of mental illness in general health care. Psychol Med 1997;27:191–7]	=1 if 24 or more
