# Supplementary material for: The UK Coronavirus Job Retention Scheme and diet, physical activity, and sleep during the COVID-19 pandemic: evidence from eight longitudinal population surveys
Source: BMC Med. 2022 Apr 6;20:147. doi: 10.1186/s12916-022-02343-y (PMC8984671; doi:10.1186/s12916-022-02343-y)
Supplement: Supplementary file 3 — Additional file 3. Meta-Analysis; Table 1-6; Figure set 1-set 11. Contents: Table 1. Main analysis excluding studies with ≤5 cell counts for exposure-outcome. Table 2. Main analysis excluding studies with ≤2 cell counts for exposure-outcome. Table 3. Main analysis excluding studies with zero cell counts for exposure-outcome. Table 4. Analysis of change excluding studies with ≤ 5 cell counts. Table 5. Analysis of change excluding studies with ≤ 2 cell counts. Table 6. Analysis of change excluding studies with zero cell counts. Figure set 1: Currently eats 2 or fewer fruit & veg. Figure set 2: Fewer fruit and vegetables. Figure set 3: More fruit and vegetables. Figure set 4: Less than 3 days a week of at least 30 min exercise. Figure set 5: Less time/fewer days of physical exercise. Figure set 6: More time/days of physical exercise. Figure set 7: Sleeps outside ‘Normal Range’ (i.e. <6 or 9+ hours). Figure set 8: Sleeps less than before. Figure set 9: Sleeps more than before. Figure set 10: From 6/9 hours a night to outside ‘normal range’. Figure set 11: From outside ‘normal range’ to 6/9h a night. [file 12916_2022_2343_MOESM3_ESM.pdf]

Additional File 3: Meta-analysis results

Summary Tables

Table 1. Main analysis excluding studies with ≤5 cell counts for exposure-outcome 2
Table 2. Main analysis excluding studies with ≤2 cell counts for exposure-outcome 3
Table 3. Main analysis excluding studies with zero cell counts for exposure-outcome 4
Table 4. Analysis of change excluding studies with ≤ 5 cell counts 5
Table 5. Analysis of change excluding studies with ≤ 2 cell counts 8
Table 6. Analysis of change excluding studies with zero cell counts 11

Forest Plots (*Unadjusted, Basic, and Fully adjusted*)

Figure set 1: Currently eats 2 or fewer fruit & veg 14
Figure set 2: Fewer fruit and vegetables 19
Figure set 3: More fruit and vegetables 24
Figure set 4: Less than 3 days a week of at least 30 min exercise 29
Figure set 5: Less time/fewer days of physical exercise 34
Figure set 6: More time/days of physical exercise 39
Figure set 7: Sleeps outside 'Normal Range' (i.e. <6 or 9+ hours) 44
Figure set 8: Sleeps less than before 49
Figure set 9: Sleeps more than before 54
Figure set 10: From 6/9 hours a night to outside 'normal range' 59
Figure set 11: From outside 'normal range' to 6/9h a night 64

Notes: Basic adjustment includes socio-demographic characteristics: age (only in age-heterogeneous studies), sex, ethnicity (except the BCS70 and NCDS cohorts which were nearly are almost entirely white), education, UK nation (except ALSPAC, GS and ELSA which only had participants from a single country), and household composition. Full adjustment additionally includes pre-pandemic measures of psychological distress, self-rated health, and health behaviours.

Table 1. Main analysis excluding studies with ≤5 cell counts for exposure-outcome

		Currently eats 2 or fewer fruit & veg				Less than 3 days a week of at least 30min exercise				Sleeps outside 'Normal Range' (i.e. <6 or 9+ hours)			
		RR	Lower CI	Upper CI	I2%	RR	Lower CI	Upper CI	I2%	RR	Lower CI	Upper CI	I2%
	Overall	0.97	0.87	1.09	41.83	0.85	0.75	0.97	58.73	1.28	0.92	1.78	84.34
	female	0.84	0.68	1.04	65.28	0.87	0.79	0.95	0	1.30	0.89	1.88	83.76
	male	1.11	1.01	1.22	0	0.88	0.75	1.03	41.13	1.14	0.88	1.47	24.55
Furloughed	Degree	0.96	0.71	1.30	76.09	0.81	0.72	0.91	0.00	1.28	0.81	2.01	73.72
	No degree	1.00	0.91	1.09	0.00	0.88	0.75	1.03	60.15	1.20	0.94	1.54	61.57
	16-29y	0.84	0.57	1.25	66.75	0.76	0.56	1.03	0.00	1.39	0.31	6.16	90.51
	30-49y	0.89	0.77	1.04	0.00	0.83	0.59	1.17	65.57	1.10	0.78	1.54	36.92
	50+y	1.06	0.96	1.17	0.00	0.86	0.72	1.03	68.17	1.10	0.88	1.39	46.33
	Overall	0.95	0.81	1.12	0	1.02	0.85	1.24	40.11	1.38	0.81	2.36	82.29
	female	0.81	0.55	1.18	49.84	1.11	0.94	1.31	1.06	1.34	0.83	2.16	68.97
	male	1.14	0.93	1.39	0	0.98	0.80	1.21	0	1.28	0.31	5.20	85.62
No longer employed	Degree	0.92	0.62	1.36	0.00	1.08	0.80	1.47	53.38	1.58	0.41	6.00	74.04
	No degree	1.08	0.89	1.30	0.00	0.96	0.80	1.15	0.00	1.46	0.86	2.49	71.78
	16-29y	0.92	0.59	1.43	45.37	0.98	0.46	2.09	73.41	3.80	2.35	6.15	
	30-49y	0.77	0.41	1.44	59.88	1.28	0.96	1.71	0.00	1.36	0.58	3.20	57.29
	50+y	0.99	0.78	1.25	0.00	1.02	0.86	1.20	0.03	1.09	0.70	1.68	56.48
	Overall	1.00	0.83	1.20	0	1.21	0.91	1.61	74.71	1.76	0.85	3.64	85.29
	female	0.94	0.72	1.23	0	1.20	0.90	1.59	42.82	2.34	1.43	3.81	51.22
	male	1.07	0.84	1.36	0	1.17	0.74	1.84	76.87	1.66	0.85	3.23	63.24
Stable unemployed	Degree	1.04	0.63	1.73	*	0.83	0.69	0.99	*	no information			
	No degree	1.03	0.84	1.26	0.00	1.33	0.96	1.82	72.69	2.19	1.48	3.25	28.66
	16-29y	1.27	0.63	2.56	87.75	1.50	0.41	5.39	91.84	2.92	1.69	5.05	*
	30-49y	0.83	0.36	1.93	69.28	1.41	1.15	1.72	0.00	2.09	0.89	4.89	70.99
	50+y	0.71	0.38	1.33	64.94	1.17	0.90	1.53	26.48	0.96	0.39	2.39	78.41

* indicates only one study included

Table 2. Main analysis excluding studies with ≤2 cell counts for exposure-outcome

		Currently eats 2 or fewer fruit & veg				Less than 3 days a week of at least 30min exercise				Sleeps outside 'Normal Range' (i.e. <6 or 9+ hours)			
		RR	Lower CI	Upper CI	I2%	RR	Lower CI	Upper CI	I2%	RR	Lower CI	Upper CI	I2%
Furloughed	Overall	0.97	0.87	1.09	41.83	0.85	0.75	0.97	58.73	1.28	0.92	1.78	84.34
	female	0.84	0.68	1.04	65.28	0.87	0.79	0.95	0	1.30	0.89	1.88	83.76
	male	1.11	1.01	1.22	0	0.88	0.75	1.03	41.13	1.14	0.88	1.47	24.55
	Degree	0.96	0.71	1.30	76.09	0.81	0.72	0.91	0.00	1.18	0.80	1.74	65.59
	No degree	1.00	0.91	1.09	0.00	0.88	0.75	1.03	60.15	1.20	0.94	1.54	61.57
	16-29y	0.84	0.57	1.25	66.75	0.76	0.56	1.03	0.00	1.39	0.31	6.16	90.51
	30-49y	0.89	0.77	1.04	0.00	0.83	0.59	1.17	65.57	1.10	0.78	1.54	36.92
	50+y	1.06	0.96	1.17	0.00	0.86	0.72	1.03	68.17	1.10	0.88	1.39	46.33
No longer employed	Overall	0.95	0.81	1.12	0	1.02	0.85	1.24	40.11	1.38	0.81	2.36	82.29
	female	0.81	0.55	1.18	49.84	1.11	0.94	1.31	1.06	1.34	0.83	2.16	68.97
	male	1.14	0.93	1.39	0	0.98	0.80	1.21	0	1.23	0.52	2.92	74.05
	Degree	0.79	0.46	1.36	69.70	1.08	0.80	1.47	53.38	1.11	0.72	1.71	12.58
	No degree	1.00	0.84	1.19	0.00	0.96	0.80	1.15	0.00	1.46	0.86	2.49	71.78
	16-29y	0.92	0.59	1.43	45.37	0.98	0.46	2.09	73.41	3.80	2.35	6.15	*
	30-49y	0.77	0.41	1.44	59.88	1.28	0.96	1.71	0.00	1.16	0.60	2.26	45.34
	50+y	0.99	0.78	1.25	0.00	1.02	0.86	1.20	0.03	1.09	0.70	1.68	56.48
Stable unemployed	Overall	1.00	0.83	1.20	0	1.15	0.90	1.47	62.25	1.44	0.69	2.99	85.86
	female	0.90	0.70	1.17	0	1.09	0.78	1.52	56.95	1.31	0.54	3.17	85.51
	male	1.28	0.87	1.88	63.67	1.15	0.89	1.47	50.01	2.09	1.04	4.20	71.15
	Degree	1.01	0.50	2.06	82.44	1.02	0.70	1.50	66.83	1.97	1.17	3.31	35.72
	No degree	1.02	0.83	1.24	0.00	1.32	1.03	1.68	58.58	1.65	0.75	3.65	82.22
	16-29y	1.27	0.63	2.56	87.75	1.50	0.41	5.39	91.84	2.75	1.63	4.63	0.00
	30-49y	0.83	0.36	1.93	69.28	1.41	1.15	1.72	0.00	2.09	0.89	4.89	70.99
	50+y	0.71	0.38	1.33	64.94	1.12	0.93	1.34	0.00	0.98	0.53	1.80	60.57

* indicates only one study included

Table 3. Main analysis excluding studies with zero cell counts for exposure-outcome

		Currently eats 2 or fewer fruit & veg				Less than 3 days a week of at least 30min exercise				Sleeps outside 'Normal Range' (i.e. <6 or 9+ hours)			
		RR	Lower CI	Upper CI	I2%	RR	Lower CI	Upper CI	I2%	RR	Lower CI	Upper CI	I2%
Furloughed	Overall	0.97	0.87	1.09	41.83	0.85	0.75	0.97	58.73	1.28	0.92	1.78	84.34
	female	0.84	0.68	1.04	65.28	0.87	0.79	0.95	0	1.30	0.89	1.88	83.76
	male	1.11	1.01	1.22	0	0.88	0.75	1.03	41.13	1.14	0.88	1.47	24.55
	Degree	0.96	0.71	1.30	76.09	0.81	0.72	0.91	0.00	1.18	0.80	1.74	65.59
	No degree	1.00	0.91	1.09	0.00	0.88	0.75	1.03	60.15	1.20	0.94	1.54	61.57
	16-29y	0.84	0.57	1.25	66.75	0.76	0.56	1.03	0.00	1.39	0.31	6.16	90.51
	30-49y	0.89	0.77	1.04	0.00	0.83	0.59	1.17	65.57	1.10	0.78	1.54	36.92
	50+y	1.06	0.96	1.17	0.00	0.86	0.72	1.03	68.17	1.10	0.88	1.39	46.33
No longer employed	Overall	0.95	0.81	1.12	0	1.02	0.85	1.24	40.11	1.38	0.81	2.36	82.29
	female	0.80	0.56	1.13	40.08	1.11	0.94	1.31	1.06	1.34	0.83	2.16	68.97
	male	1.14	0.93	1.39	0	0.98	0.80	1.21	0	1.23	0.52	2.92	74.05
	Degree	0.79	0.46	1.36	69.70	1.08	0.80	1.47	53.38	1.11	0.72	1.71	12.58
	No degree	1.00	0.84	1.19	0.00	0.96	0.80	1.15	0.00	1.46	0.86	2.49	71.78
	16-29y	0.92	0.59	1.43	45.37	0.98	0.46	2.09	73.41	3.80	2.35	6.15	*
	30-49y	0.77	0.41	1.44	59.88	1.28	0.96	1.71	0.00	1.16	0.60	2.26	45.34
	50+y	0.99	0.78	1.25	0.00	1.02	0.86	1.20	0.03	1.09	0.70	1.68	56.48
Stable unemployed	Overall	1.00	0.83	1.20	0	1.15	0.90	1.47	62.25	1.48	0.80	2.75	80.5
	female	0.90	0.70	1.17	0	1.09	0.78	1.52	56.95	1.29	0.58	2.85	80.81
	male	1.22	0.83	1.77	57.81	1.20	0.95	1.53	51.36	1.96	1.14	3.39	58.6
	Degree	1.01	0.50	2.06	82.44	0.98	0.68	1.40	55.89	1.75	1.22	2.50	0.00
	No degree	1.02	0.83	1.24	0.00	1.32	1.03	1.68	58.58	1.37	0.70	2.70	76.04
	16-29y	1.27	0.63	2.56	87.75	1.50	0.41	5.39	91.84	2.75	1.63	4.63	0.00
	30-49y	0.83	0.36	1.93	69.28	1.39	1.14	1.70	0.00	2.09	0.89	4.89	70.99
	50+y	0.71	0.38	1.33	64.94	1.12	0.93	1.34	0.00	0.98	0.53	1.80	60.57

* indicates only one study included

Table 4. Analysis of change excluding studies with ≤ 5 cell counts

		Fewer fruit and veg				More fruit and veg			
		RR	Lower CI	Upper CI	I2%	RR	Lower CI	Upper CI	I2%
Furloughed	Overall	0.96	0.87	1.08	8.96	1.22	1.04	1.43	52.46
	female	0.91	0.81	1.02	0	1.16	1.03	1.31	5.34
	male	1.03	0.90	1.19	1.11	1.21	0.98	1.50	40.11
	Degree	0.95	0.70	1.29	63.37	1.17	1.04	1.33	0
	No degree	0.97	0.87	1.09	0	1.26	0.99	1.61	62.7
	16-29y	0.75	0.38	1.47	75.61	1.18	0.91	1.52	0
	30-49y	0.88	0.76	1.03	0	1.16	0.90	1.50	42.19
	50+y	1.05	0.93	1.18	0	1.16	0.90	1.51	78.74
No longer employed	Overall	0.86	0.54	1.35	55.22	1.02	0.83	1.25	0
	female	0.88	0.68	1.14	0	1.23	1.00	1.51	0
	male	1.21	0.94	1.55		0.90	0.47	1.71	60.88
	Degree	0.72	0.30	1.75	66.85	1.17	0.91	1.51	0
	No degree	1.11	0.87	1.41	0	1.07	0.68	1.68	48.26
	16-29y	0.82	0.50	1.36	18.7	1.12	0.76	1.66	0
	30-49y	0.80	0.28	2.25	80.55	0.94	0.38	2.34	73.5
	50+y	1.00	0.79	1.27	0	1.00	0.80	1.24	0
Stable unemployed	Overall	0.86	0.34	2.20	86.92	0.98	0.56	1.70	56.16
	female	0.92	0.65	1.31	0	0.95	0.50	1.80	45.59
	male	0.81	0.42	1.55		1.22	0.63	2.38	49.82
	Degree	0.87	0.45	1.71		1.15	0.65	2.04	
	No degree	0.93	0.33	2.59	86.33	0.68	0.17	2.82	84.44
	16-29y	0.63	0.18	2.19	77.06	0.47	0.20	1.09	
	30-49y	0.47	0.23	0.96		1.88	1.32	2.70	
	50+y	1.22	0.54	2.74	80.15	1.17	0.84	1.63	0

		Less time/Fewer days of physical exercise				More time/days of physical exercise			
		RR	Lower CI	Upper CI	I2%	RR	Lower CI	Upper CI	I2%
Furloughed	Overall	1.06	0.96	1.17	45.87	1.18	1.04	1.35	75.47
	female	1.00	0.89	1.12	36.75	1.20	1.07	1.33	46.22
	male	1.16	1.04	1.29	5.21	1.18	0.98	1.42	63.43
	Degree	1.02	0.92	1.13	0	1.11	1.02	1.22	6.55
	No degree	1.08	0.96	1.23	47.21	1.21	1.03	1.43	73.17
	16-29y	1.08	0.93	1.26	0	1.15	0.93	1.44	31.15
	30-49y	1.11	0.89	1.38	59.5	1.03	0.91	1.16	0
	50+y	1.05	0.91	1.21	62.44	1.15	0.92	1.44	88.35
No longer employed	Overall	1.08	0.93	1.25	24.38	1.15	1.02	1.29	0
	female	1.14	0.91	1.44	57.53	1.01	0.87	1.18	8.32
	male	1.02	0.74	1.40	49.2	1.38	1.16	1.64	0
	Degree	0.93	0.77	1.13	0	1.08	0.93	1.26	0
	No degree	1.32	1.06	1.64	48.6	1.20	1.02	1.40	0
	16-29y	0.98	0.72	1.32	26.23	1.22	0.92	1.63	0
	30-49y	1.12	0.77	1.64	18.69	1.12	0.79	1.59	35.14
	50+y	1.09	0.94	1.27	14.01	1.06	0.92	1.22	0
Stable unemployed	Overall	0.84	0.67	1.05	17.28	1.07	0.91	1.25	0
	female	0.86	0.69	1.07	0	1.07	0.88	1.29	0
	male	0.80	0.56	1.15	0	1.24	0.93	1.66	0
	Degree	0.72	0.45	1.15	26.74	1.10	0.88	1.36	0
	No degree	0.88	0.58	1.34	67.86	1.15	0.93	1.42	0
	16-29y	0.83	0.55	1.26	0.69	1.22	0.89	1.68	0
	30-49y	1.27	0.87	1.86		0.60	0.34	1.08	4.48
	50+y	0.90	0.63	1.27	59.44	1.04	0.87	1.25	0

		Sleep less than before				Sleep more than before				From 6/9h a night to outside 'normal range				From outside 'normal range' to 6/9h a night			
		RR	Lower CI	Upper CI	I2%	RR	Lower CI	Upper CI	I2%	RR	Lower CI	Upper CI	I2%	RR	Lower CI	Upper CI	I2%
Furloughed	Overall	0.89	0.75	1.07	71.51	1.62	1.39	1.90	80.15	1.46	1.04	2.07	75.13	1.78	1.03	3.07	75.7
	female	0.90	0.80	1.02	21.96	1.54	1.32	1.80	68.28	1.36	0.89	2.09	77.96	1.63	1.07	2.50	43.73
	male	0.89	0.66	1.22	69.92	1.70	1.41	2.05	64.92	1.41	1.07	1.86	0	2.29	0.58	9.09	84.99
	Degree	0.98	0.80	1.22	50.81	1.47	1.27	1.70	50.49	1.86	0.60	5.77	93.42	1.28	0.83	1.97	0
	No degree	0.87	0.72	1.05	61.35	1.69	1.39	2.06	76.78	1.40	1.15	1.72	1.2	2.21	1.05	4.65	74.68
	16-29y	0.90	0.64	1.28	55.26	1.37	1.17	1.61	0	3.35	2.13	5.27	*	1.46	0.48	4.43	*
	30-49y	0.80	0.66	0.96	0	1.41	1.15	1.72	58.96	1.20	0.78	1.84	33.3	1.60	0.65	3.93	60.62
	50+y	0.91	0.71	1.17	79.01	1.65	1.25	2.16	88.98	1.31	1.05	1.63	0	1.62	0.68	3.87	87.1
No longer employed	Overall	0.97	0.83	1.14	0	1.46	1.24	1.72	40.22	1.35	0.68	2.66	72.61	1.14	0.63	2.05	0
	female	1.02	0.86	1.21	0	1.33	1.10	1.60	23.95	2.18	1.46	3.26	0	2.03	0.94	4.37	*
	male	1.12	0.79	1.58	0	1.62	1.26	2.07	37.91	6.38	1.89	21.51	*		no information		
	Degree	1.16	0.83	1.62	50.56	1.32	1.09	1.59	16.79	21.63	4.94	94.68	*		no information		
	No degree	0.92	0.74	1.15	0	1.56	1.27	1.92	30.74	1.90	1.13	3.18	0	1.49	0.59	3.80	
	16-29y	1.12	0.72	1.75	42.98	1.16	0.89	1.50	0	3.64	1.92	6.92	*		no information		
	30-49y	0.81	0.51	1.30	0	1.46	1.17	1.81	0	1.83	0.73	4.56	*		no information		
	50+y	0.97	0.80	1.17	0	1.52	1.24	1.86	41.46	1.44	0.73	2.84	67.03	1.59	0.79	3.22	
Stable unemployed	Overall	1.11	0.88	1.40	22.06	1.29	1.06	1.56	0	1.54	0.87	2.73	39.19	0.35	0.13	0.96	*
	female	1.09	0.82	1.44	21.38	1.34	1.10	1.63	0	1.74	0.82	3.67	*	0.57	0.19	1.67	*
	male	1.35	0.89	2.07	42.06	1.12	0.66	1.92	55.81	3.97	1.17	13.44	*		no information		
	Degree	1.22	0.79	1.88	0	1.34	0.97	1.86	0		no information				no information		
	No degree	1.21	0.96	1.52	0	1.39	1.08	1.79	12.06	2.02	1.14	3.56	0	0.35	0.12	1.02	
	16-29y	0.98	0.64	1.52	0	1.27	0.93	1.75	0	2.91	1.40	6.06	*		no information		
	30-49y	1.12	0.70	1.82	0	1.17	0.78	1.74	0	1.41	0.49	4.07	*		no information		
	50+y	1.00	0.62	1.62	69.84	1.25	0.90	1.74	26.11	1.00	0.51	1.94	0		no information		

* indicates only one study included

Table 5. Analysis of change excluding studies with ≤ 2 cell counts

		Fewer fruit and veg				More fruit and veg			
		RR	Lower CI	Upper CI	I2%	RR	Lower CI	Upper CI	I2%
Furloughed	Overall	0.96	0.87	1.08	8.96	1.22	1.04	1.43	52.46
	female	0.91	0.81	1.02	0	1.16	1.03	1.31	5.34
	male	1.03	0.90	1.19	1.11	1.21	0.98	1.50	40.11
	Degree	0.95	0.70	1.29	63.37	1.17	1.04	1.33	0
	No degree	0.97	0.87	1.09	0	1.26	0.99	1.61	62.7
	16-29y	0.75	0.38	1.47	75.61	1.18	0.91	1.52	0
	30-49y	0.88	0.76	1.03	0	1.16	0.90	1.50	42.19
	50+y	1.05	0.93	1.18	0	1.16	0.90	1.51	78.74
No longer employed	Overall	0.74	0.44	1.22	64.1	1.02	0.83	1.25	0
	female	0.84	0.65	1.08	0	1.23	1.00	1.51	0
	male	1.19	0.93	1.50	0	0.86	0.56	1.30	23.15
	Degree	0.78	0.52	1.18	25.58	1.14	0.89	1.45	0
	No degree	1.11	0.87	1.41	0	1.07	0.68	1.68	48.26
	16-29y	0.82	0.50	1.36	18.7	1.12	0.76	1.66	0
	30-49y	0.80	0.28	2.25	80.55	0.94	0.38	2.34	73.5
	50+y	0.96	0.76	1.21	0	1.00	0.80	1.24	0
Stable unemployed	Overall	0.92	0.47	1.81	76.02	1.16	0.82	1.63	21.37
	female	1.20	0.68	2.12	60.19	1.31	0.86	2.00	35.61
	male	0.91	0.53	1.57	0	1.22	0.63	2.38	49.82
	Degree	0.63	0.26	1.50	43.9	1.78	0.93	3.40	63.88
	No degree	0.93	0.33	2.59	86.33	1.07	0.53	2.16	70.2
	16-29y	0.63	0.18	2.19	77.06	0.62	0.28	1.36	14.56
	30-49y	0.65	0.29	1.44	40.92	1.84	1.34	2.54	0
	50+y	1.22	0.54	2.74	80.15	1.18	0.86	1.63	0

		Less time/Fewer days of physical exercise				More time/days of physical exercise			
		RR	Lower CI	Upper CI	I2%	RR	Lower CI	Upper CI	I2%
Furloughed	Overall	1.06	0.96	1.17	45.87	1.18	1.04	1.35	75.47
	female	1.00	0.89	1.12	36.75	1.20	1.07	1.33	46.22
	male	1.16	1.04	1.29	5.21	1.18	0.98	1.42	63.43
	Degree	1.02	0.92	1.13	0	1.11	1.02	1.22	6.55
	No degree	1.08	0.96	1.23	47.21	1.21	1.03	1.43	73.17
	16-29y	1.08	0.93	1.26	0	1.15	0.93	1.44	31.15
	30-49y	1.11	0.89	1.38	59.5	1.03	0.91	1.16	0
	50+y	1.05	0.91	1.21	62.44	1.15	0.92	1.44	88.35
No longer employed	Overall	1.08	0.93	1.25	24.38	1.15	1.02	1.29	0
	female	1.14	0.91	1.44	57.53	1.01	0.87	1.18	8.32
	male	0.97	0.70	1.34	50.83	1.36	1.15	1.61	0
	Degree	0.95	0.79	1.13	0	1.07	0.92	1.25	0
	No degree	1.27	1.01	1.59	50.35	1.20	1.02	1.40	0
	16-29y	0.98	0.72	1.32	26.23	1.22	0.92	1.63	0
	30-49y	1.12	0.77	1.64	18.69	1.12	0.79	1.59	35.14
	50+y	1.09	0.94	1.27	14.01	1.06	0.92	1.22	0
Stable unemployed	Overall	0.85	0.69	1.04	10.43	1.06	0.90	1.24	0
	female	0.91	0.73	1.14	10.95	1.03	0.86	1.24	0
	male	0.77	0.55	1.08	0	1.15	0.88	1.51	0
	Degree	0.92	0.58	1.46	51.71	1.10	0.89	1.36	0
	No degree	0.91	0.64	1.29	56.5	1.10	0.90	1.35	0
	16-29y	0.83	0.55	1.26	0.69	1.22	0.89	1.68	0
	30-49y	1.13	0.70	1.81	20.72	0.60	0.34	1.08	4.48
	50+y	0.92	0.67	1.26	50.47	1.04	0.87	1.25	0

		Sleep less than before				Sleep more than before				From 6/9h a night to outside 'normal range				From outside 'normal range' to 6/9h a night			
		RR	Lower CI	Upper CI	I2%	RR	Lower CI	Upper CI	I2%	RR	Lower CI	Upper CI	I2%	RR	Lower CI	Upper CI	I2%
Furloughed	Overall	0.89	0.75	1.07	71.51	1.62	1.39	1.90	80.15	1.46	1.04	2.07	75.13	1.78	1.03	3.07	75.7
	female	0.90	0.80	1.02	21.96	1.54	1.32	1.80	68.28	1.36	0.89	2.09	77.96	1.63	1.07	2.50	43.73
	male	0.89	0.66	1.22	69.92	1.70	1.41	2.05	64.92	1.41	1.07	1.86	0	1.60	0.64	4.01	73.78
	Degree	0.98	0.80	1.22	50.81	1.47	1.27	1.70	50.49	1.62	0.64	4.12	91.03	1.61	0.93	2.77	45.16
	No degree	0.87	0.72	1.05	61.35	1.69	1.39	2.06	76.78	1.40	1.15	1.72	1.2	2.21	1.05	4.65	74.68
	16-29y	0.90	0.64	1.28	55.26	1.37	1.17	1.61	0	1.67	0.36	7.75	84.08	1.02	0.45	2.32	0
	30-49y	0.80	0.66	0.96	0	1.41	1.15	1.72	58.96	1.20	0.78	1.84	33.3	1.35	0.79	2.29	22.42
	50+y	0.91	0.71	1.17	79.01	1.65	1.25	2.16	88.98	1.31	1.05	1.63	0	1.62	0.68	3.87	87.1
No longer employed	Overall	0.97	0.83	1.14	0	1.46	1.24	1.72	40.22	1.44	0.84	2.49	70.63	1.53	0.93	2.50	4.52
	female	1.02	0.86	1.21	0	1.33	1.10	1.60	23.95	1.32	0.76	2.30	59.54	2.24	1.20	4.20	0
	male	1.08	0.79	1.47	0	1.61	1.29	2.03	30.87	1.71	0.77	3.79	53.91	1.32	0.58	3.01	0
	Degree	1.17	0.86	1.58	42.7	1.32	1.09	1.59	16.79	1.68	0.51	5.51	79.65	1.17	0.40	3.40	0
	No degree	0.93	0.75	1.14	0	1.56	1.27	1.92	30.74	1.57	0.89	2.76	54.68	1.22	0.59	2.51	0
	16-29y	1.12	0.72	1.75	42.98	1.16	0.89	1.50	0	3.64	1.92	6.92	*	2.25	0.52	9.75	*
	30-49y	0.75	0.49	1.15	0	1.46	1.17	1.81	0	1.40	0.67	2.95	0	1.23	0.38	3.95	0
	50+y	0.97	0.80	1.17	0	1.52	1.24	1.86	41.46	1.28	0.69	2.39	61.19	1.56	0.70	3.43	47.9
Stable unemployed	Overall	1.10	0.88	1.37	16.56	1.28	1.06	1.54	0	1.45	0.84	2.51	33.89	1.13	0.10	12.58	85.65
	female	1.09	0.82	1.44	21.38	1.53	1.24	1.91	24.43	1.58	0.92	2.71	0	0.82	0.27	2.49	20.78
	male	1.35	0.89	2.07	42.06	1.12	0.66	1.92	55.81	1.71	0.79	3.69	23.67	no information			
	Degree	1.16	0.81	1.67	0	1.49	0.90	2.45	79.12	2.19	1.02	4.70	0	no information			
	No degree	1.17	0.94	1.46	0	1.39	1.08	1.79	12.06	1.66	1.02	2.69	0	1.80	0.34	9.66	83.06
	16-29y	1.12	0.76	1.65	0	1.27	0.93	1.75	0	2.91	1.40	6.06	*	4.15	0.90	19.23	
	30-49y	1.12	0.70	1.82	0	1.17	0.78	1.74	0	1.41	0.49	4.07	*	1.06	0.36	3.13	
	50+y	1.00	0.62	1.62	69.84	1.26	0.99	1.62	1.52	1.00	0.51	1.94	0	no information			

* indicates only one study included

Table 6. Analysis of change excluding studies with zero cell counts

		Fewer fruit and veg				More fruit and veg			
		RR	Lower CI	Upper CI	I2%	RR	Lower CI	Upper CI	I2%
Furloughed	Overall	0.96	0.87	1.08	8.96	1.22	1.04	1.43	52.46
	female	0.91	0.81	1.02	0	1.16	1.03	1.31	5.34
	male	1.03	0.90	1.19	1.11	1.21	0.98	1.50	40.11
	Degree	0.95	0.70	1.29	63.37	1.17	1.04	1.33	0
	No degree	0.97	0.87	1.09	0	1.26	0.99	1.61	62.7
	16-29y	0.75	0.38	1.47	75.61	1.18	0.91	1.52	0
	30-49y	0.88	0.76	1.03	0	1.16	0.90	1.50	42.19
	50+y	1.05	0.93	1.18	0	1.16	0.90	1.51	78.74
No longer employed	Overall	0.74	0.44	1.22	64.1	1.02	0.83	1.25	0
	female	0.78	0.56	1.10	14.94	1.23	1.00	1.51	0
	male	1.16	0.91	1.47	0	0.85	0.58	1.24	13.66
	Degree	0.78	0.52	1.18	25.58	1.14	0.89	1.45	0
	No degree	1.05	0.83	1.34	0	1.07	0.68	1.68	48.26
	16-29y	0.82	0.50	1.36	18.7	1.12	0.76	1.66	0
	30-49y	0.80	0.28	2.25	80.55	0.94	0.38	2.34	73.5
	50+y	0.96	0.76	1.21	0	1.00	0.80	1.24	0
Stable unemployed	Overall	0.85	0.45	1.59	71.11	1.16	0.82	1.63	21.37
	female	1.06	0.66	1.70	42.23	1.31	0.86	2.00	35.61
	male	0.87	0.38	1.99	57.38	1.19	0.69	2.05	24.75
	Degree	0.72	0.44	1.19	0.08	1.78	0.93	3.40	63.88
	No degree	0.87	0.42	1.82	70.36	1.07	0.53	2.16	70.2
	16-29y	0.63	0.18	2.19	77.06	0.62	0.28	1.36	14.56
	30-49y	0.65	0.29	1.44	40.92	1.84	1.34	2.54	0
	50+y	1.02	0.48	2.19	71.06	1.18	0.86	1.63	0

		Less time/Fewer days of physical exercise				More time/days of physical exercise			
		RR	Lower CI	Upper CI	I2%	RR	Lower CI	Upper CI	I2%
Furloughed	Overall	1.06	0.96	1.17	45.87	1.18	1.04	1.35	75.47
	female	1.00	0.89	1.12	36.75	1.20	1.07	1.33	46.22
	male	1.16	1.04	1.29	5.21	1.18	0.98	1.42	63.43
	Degree	1.02	0.92	1.13	0	1.11	1.02	1.22	6.55
	No degree	1.08	0.96	1.23	47.21	1.21	1.03	1.43	73.17
	16-29y	1.08	0.93	1.26	0	1.15	0.93	1.44	31.15
	30-49y	1.11	0.89	1.38	59.5	1.03	0.91	1.16	0
	50+y	1.05	0.91	1.21	62.44	1.15	0.92	1.44	88.35
No longer employed	Overall	1.08	0.93	1.25	24.38	1.15	1.02	1.29	0
	female	1.14	0.91	1.44	57.53	1.01	0.87	1.18	8.32
	male	0.97	0.70	1.34	50.83	1.36	1.15	1.61	0
	Degree	0.93	0.78	1.12	0	1.07	0.92	1.25	0
	No degree	1.27	1.01	1.59	50.35	1.20	1.02	1.40	0
	16-29y	0.98	0.72	1.32	26.23	1.22	0.92	1.63	0
	30-49y	1.12	0.77	1.64	18.69	1.12	0.79	1.59	35.14
	50+y	1.09	0.94	1.27	14.01	1.09	0.96	1.25	0
Stable unemployed	Overall	0.83	0.69	1.02	7.65	1.05	0.90	1.23	0
	female	0.92	0.73	1.14	9.22	1.03	0.86	1.24	0
	male	0.79	0.58	1.09	0	1.11	0.86	1.45	0
	Degree	0.86	0.58	1.28	34.21	1.10	0.90	1.35	0
	No degree	0.91	0.64	1.29	56.5	1.09	0.89	1.33	0
	16-29y	0.83	0.55	1.26	0.69	1.22	0.89	1.68	0
	30-49y	1.13	0.70	1.81	20.72	0.60	0.34	1.08	4.48
	50+y	0.89	0.65	1.22	45	1.03	0.86	1.24	0

		Sleep less than before				Sleep more than before				From 6/9h a night to outside 'normal range				From outside 'normal range' to 6/9h a night			
		RR	Lower CI	Upper CI	I2%	RR	Lower CI	Upper CI	I2%	RR	Lower CI	Upper CI	I2%	RR	Lower CI	Upper CI	I2%
Furloughed	Overall	0.89	0.75	1.07	71.51	1.62	1.39	1.90	80.15	1.46	1.04	2.07	75.13	1.78	1.03	3.07	75.7
	female	0.90	0.80	1.02	21.96	1.54	1.32	1.80	68.28	1.36	0.89	2.09	77.96	1.63	1.07	2.50	43.73
	male	0.89	0.66	1.22	69.92	1.70	1.41	2.05	64.92	1.41	1.07	1.86	0	1.57	0.70	3.55	66.38
	Degree	0.98	0.80	1.22	50.81	1.47	1.27	1.70	50.49	1.62	0.64	4.12	91.03	1.36	0.79	2.33	39.8
	No degree	0.87	0.72	1.05	61.35	1.69	1.39	2.06	76.78	1.40	1.15	1.72	1.2	2.21	1.05	4.65	74.68
	16-29y	0.90	0.64	1.28	55.26	1.37	1.17	1.61	0	1.67	0.36	7.75	84.08	1.02	0.45	2.32	0
	30-49y	0.80	0.66	0.96	0	1.41	1.15	1.72	58.96	1.20	0.78	1.84	33.3	1.35	0.79	2.29	22.42
	50+y	0.91	0.71	1.17	79.01	1.65	1.25	2.16	88.98	1.31	1.05	1.63	0	1.62	0.68	3.87	87.1
No longer employed	Overall	0.97	0.83	1.14	0	1.46	1.24	1.72	40.22	1.44	0.84	2.49	70.63	1.49	0.93	2.37	0
	female	1.02	0.86	1.21	0	1.33	1.10	1.60	23.95	1.32	0.76	2.30	59.54	1.93	1.14	3.26	0
	male	1.02	0.75	1.37	0	1.61	1.29	2.03	30.87	1.71	0.77	3.79	53.91	1.51	0.74	3.07	0
	Degree	1.17	0.86	1.58	42.7	1.30	1.08	1.57	16.03	1.54	0.59	4.00	74.01	1.25	0.61	2.58	0
	No degree	0.93	0.75	1.14	0	1.56	1.27	1.92	30.74	1.57	0.89	2.76	54.68	2.45	1.08	5.57	41.89
	16-29y	1.12	0.72	1.75	42.98	1.16	0.89	1.50	0	3.64	1.92	6.92	*	1.78	0.59	5.39	0
	30-49y	0.75	0.49	1.15	0	1.46	1.17	1.81	0	1.16	0.58	2.30	8.35	1.58	0.65	3.82	0
	50+y	0.97	0.80	1.17	0	1.52	1.24	1.86	41.46	1.28	0.69	2.39	61.19	1.50	0.78	2.89	27.27
Stable unemployed	Overall	1.04	0.81	1.34	29.37	1.28	1.06	1.54	0	1.23	0.68	2.25	43.72	2.58	0.82	8.11	77.94
	female	0.92	0.65	1.31	43.35	1.50	1.23	1.84	14.11	1.26	0.77	2.09	0	2.85	0.87	9.37	72.45
	male	1.28	0.84	1.95	41.61	1.06	0.71	1.60	34.02	1.65	0.99	2.76	0	4.03	1.27	12.83	0
	Degree	1.00	0.66	1.50	17.22	1.47	0.94	2.30	72.75	1.69	0.70	4.08	48.4	2.18	0.26	18.02	*
	No degree	1.17	0.94	1.46	0	1.33	1.03	1.70	11.59	1.66	1.02	2.69	0	3.41	0.84	13.83	78.46
	16-29y	1.12	0.76	1.65	0	1.27	0.93	1.75	0	2.84	1.42	5.67	0	4.15	0.90	19.23	*
	30-49y	1.53	0.80	2.90	68.07	1.17	0.78	1.74	0	1.34	0.60	2.98	0	3.77	0.81	17.48	74.69
	50+y	0.84	0.51	1.39	69.74	1.26	0.99	1.62	1.52	0.87	0.48	1.58	0	1.79	0.29	11.03	82.46

* indicates only one study included

Figure set 1: Currently eats 2 or fewer fruit & veg

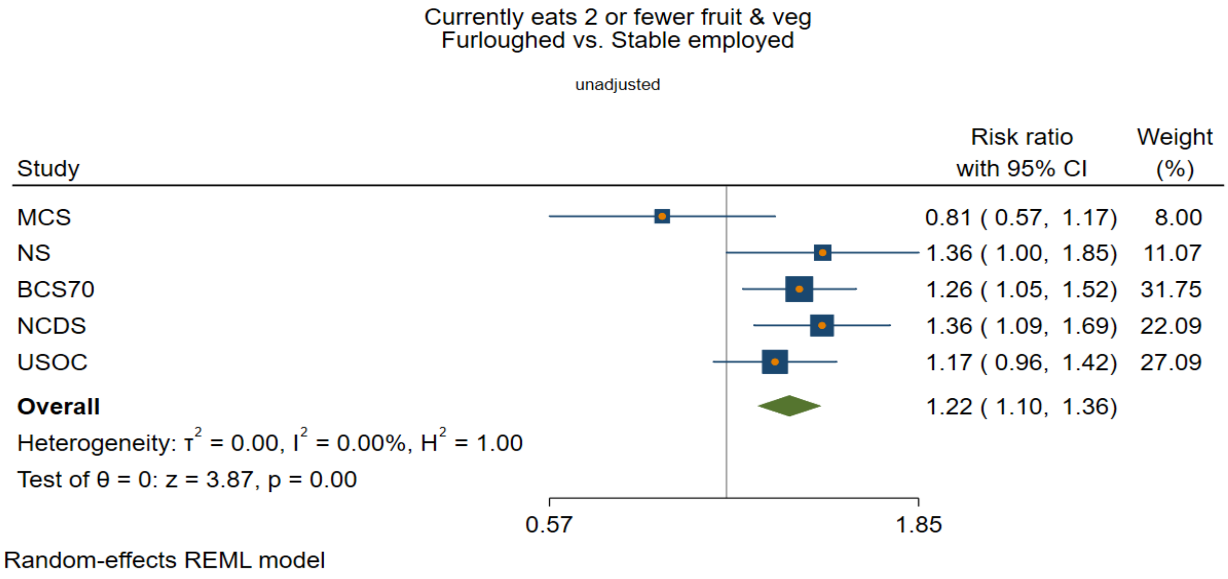

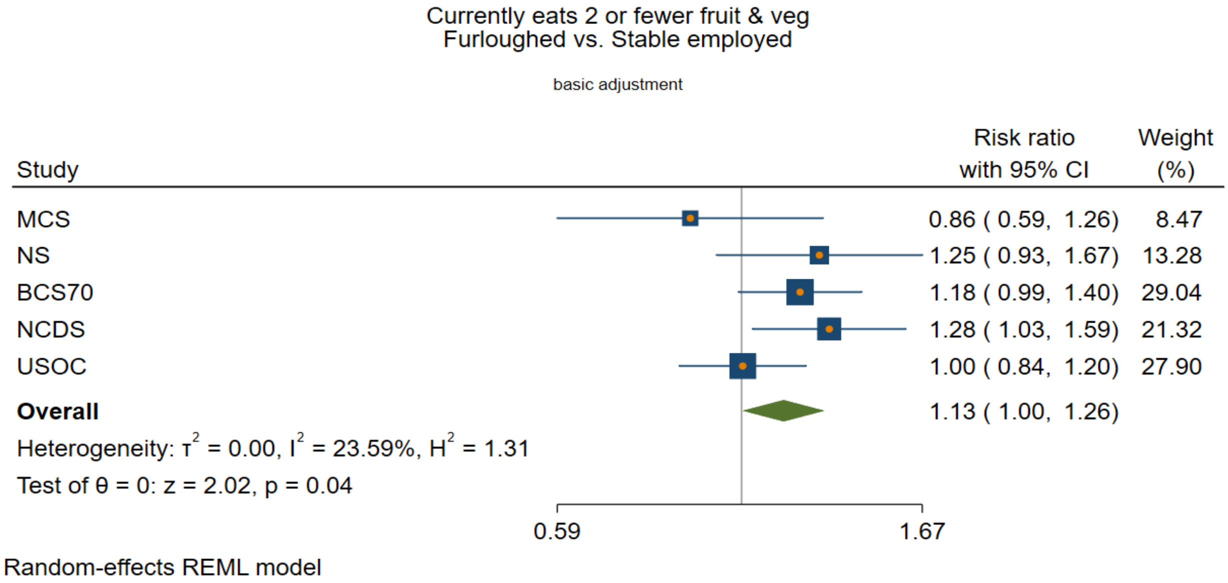

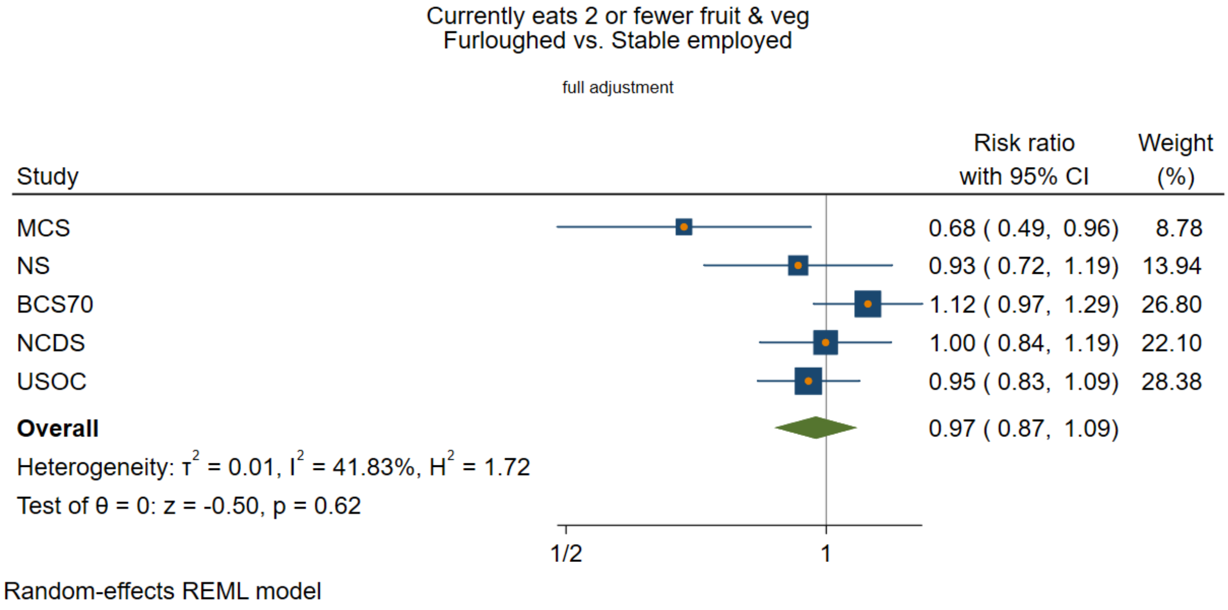

Currently eats 2 or fewer fruit & veg
No longer employed vs. Stable employed

unadjusted

Study	Risk ratio with 95% CI	Weight (%)
MCS	0.83 (0.49, 1.40)	20.50
NS	0.52 (0.22, 1.19)	11.51
BCS70	0.91 (0.46, 1.80)	15.13
NCDS	1.33 (0.83, 2.12)	23.26
USOC	1.46 (1.03, 2.06)	29.60
**Overall**	1.05 (0.75, 1.46)	

Heterogeneity: $\tau^2 = 0.06$, $I^2 = 47.44\%$, $H^2 = 1.90$

Test of $\theta = 0$: z = 0.28, p = 0.78

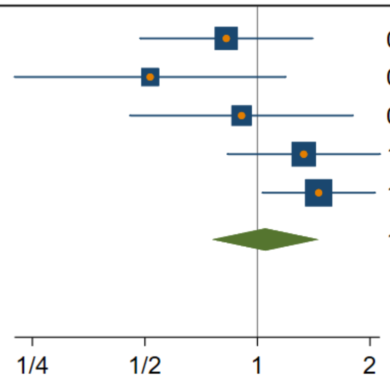

Random-effects REML model

Currently eats 2 or fewer fruit & veg
No longer employed vs. Stable employed

basic adjustment

Study	Risk ratio with 95% CI	Weight (%)
MCS	0.82 (0.49, 1.38)	17.74
NS	0.53 (0.24, 1.18)	8.88
BCS70	0.86 (0.43, 1.72)	11.44
NCDS	1.29 (0.85, 1.96)	23.98
USOC	1.22 (0.94, 1.60)	37.97
**Overall**	1.03 (0.80, 1.33)	

Heterogeneity: $\tau^2 = 0.03$, $I^2 = 31.02\%$, $H^2 = 1.45$

Test of $\theta = 0$: z = 0.22, p = 0.82

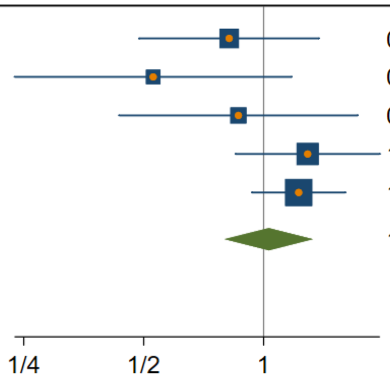

Random-effects REML model

Currently eats 2 or fewer fruit & veg
No longer employed vs. Stable employed

full adjustment

Study	Risk ratio with 95% CI	Weight (%)
MCS	0.74 (0.47, 1.16)	12.49
NS	0.51 (0.24, 1.06)	4.76
BCS70	0.94 (0.59, 1.48)	12.24
NCDS	1.05 (0.78, 1.42)	28.48
USOC	1.04 (0.81, 1.34)	42.03
**Overall**	0.95 (0.81, 1.12)	

Heterogeneity: $\tau^2 = 0.00$, $I^2 = 0.00\%$, $H^2 = 1.00$

Test of $\theta = 0$: z = -0.57, p = 0.57

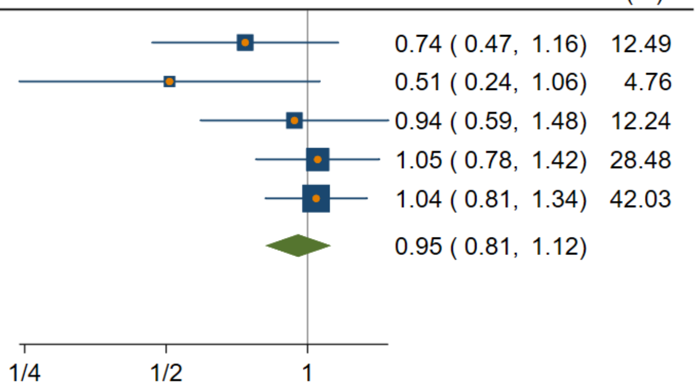

Random-effects REML model

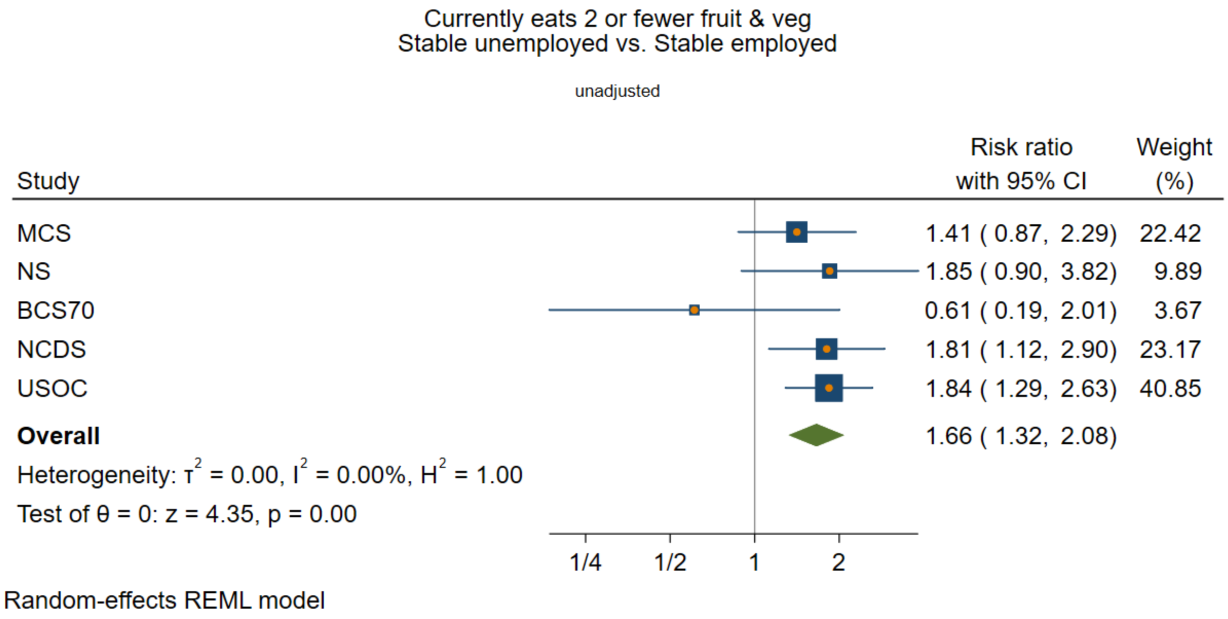
Currently eats 2 or fewer fruit & veg
Stable unemployed vs. Stable employed
unadjusted
Study
Risk ratio with 95% CI
Weight (%)
MCS 1.41 (0.87, 2.29) 22.42
NS 1.85 (0.90, 3.82) 9.89
BCS70 0.61 (0.19, 2.01) 3.67
NCDS 1.81 (1.12, 2.90) 23.17
USOC 1.84 (1.29, 2.63) 40.85
Overall 1.66 (1.32, 2.08)
Heterogeneity: τ² = 0.00, I² = 0.00%, H² = 1.00
Test of θ = 0: z = 4.35, p = 0.00
1/4
1/2
1
2
Random-effects REML model

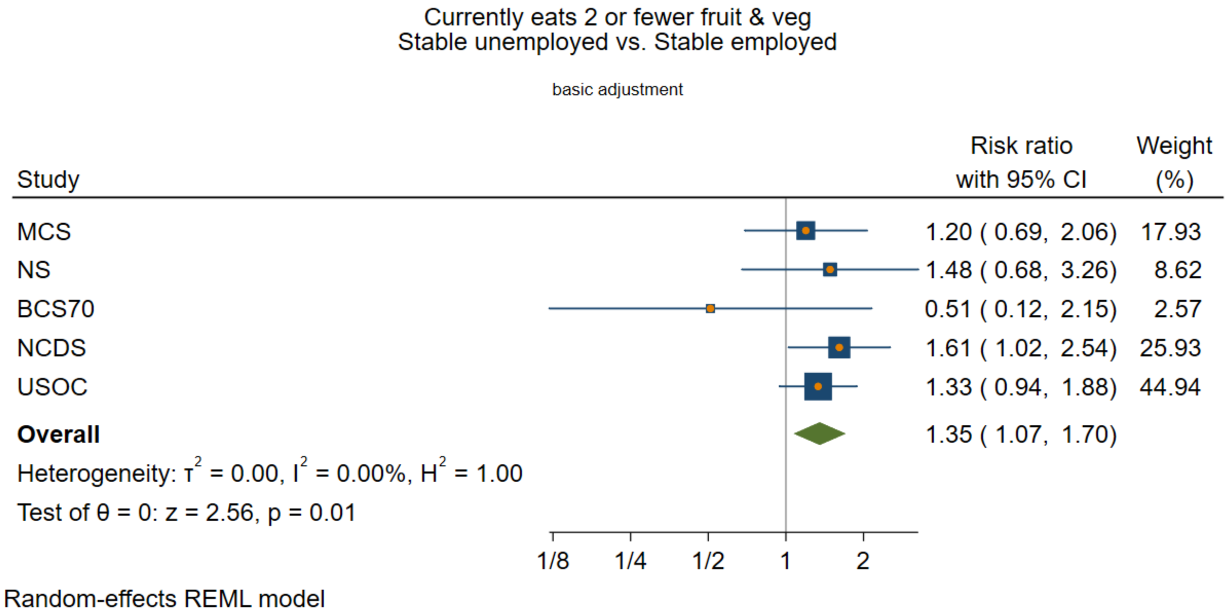
Currently eats 2 or fewer fruit & veg
Stable unemployed vs. Stable employed
basic adjustment
Study
Risk ratio with 95% CI
Weight (%)
MCS 1.20 (0.69, 2.06) 17.93
NS 1.48 (0.68, 3.26) 8.62
BCS70 0.51 (0.12, 2.15) 2.57
NCDS 1.61 (1.02, 2.54) 25.93
USOC 1.33 (0.94, 1.88) 44.94
Overall 1.35 (1.07, 1.70)
Heterogeneity: τ² = 0.00, I² = 0.00%, H² = 1.00
Test of θ = 0: z = 2.56, p = 0.01
1/8
1/4
1/2
1
2
Random-effects REML model

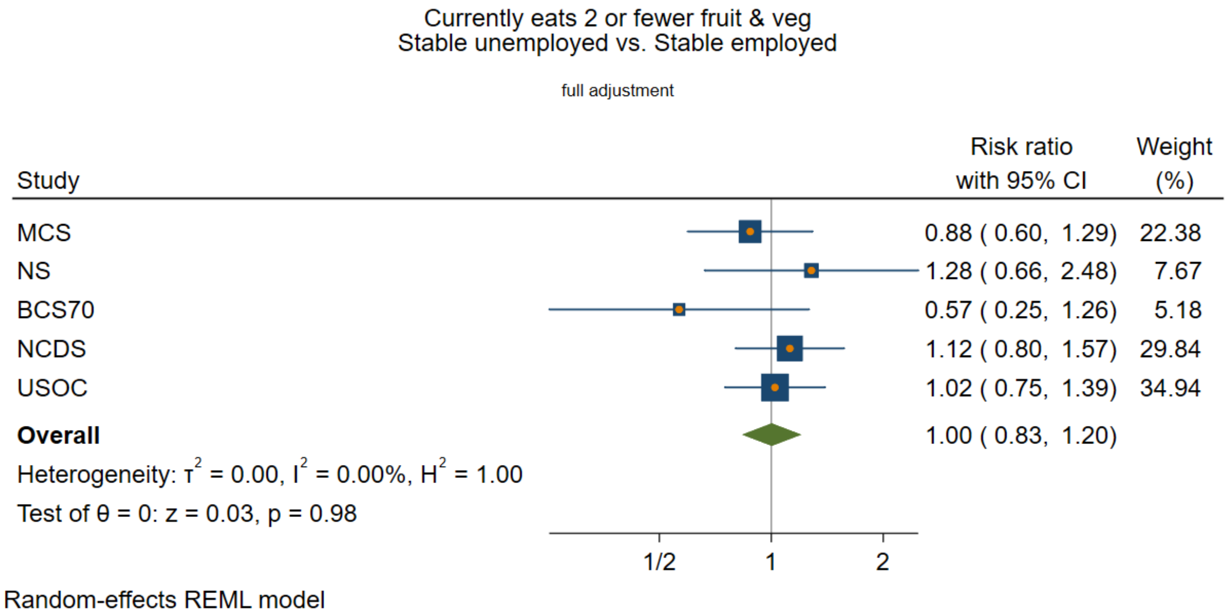
Currently eats 2 or fewer fruit & veg
Stable unemployed vs. Stable employed
full adjustment
Study
Risk ratio with 95% CI
Weight (%)
MCS 0.88 (0.60, 1.29) 22.38
NS 1.28 (0.66, 2.48) 7.67
BCS70 0.57 (0.25, 1.26) 5.18
NCDS 1.12 (0.80, 1.57) 29.84
USOC 1.02 (0.75, 1.39) 34.94
Overall 1.00 (0.83, 1.20)
Heterogeneity: τ² = 0.00, I² = 0.00%, H² = 1.00
Test of θ = 0: z = 0.03, p = 0.98
1/2
1
2
Random-effects REML model

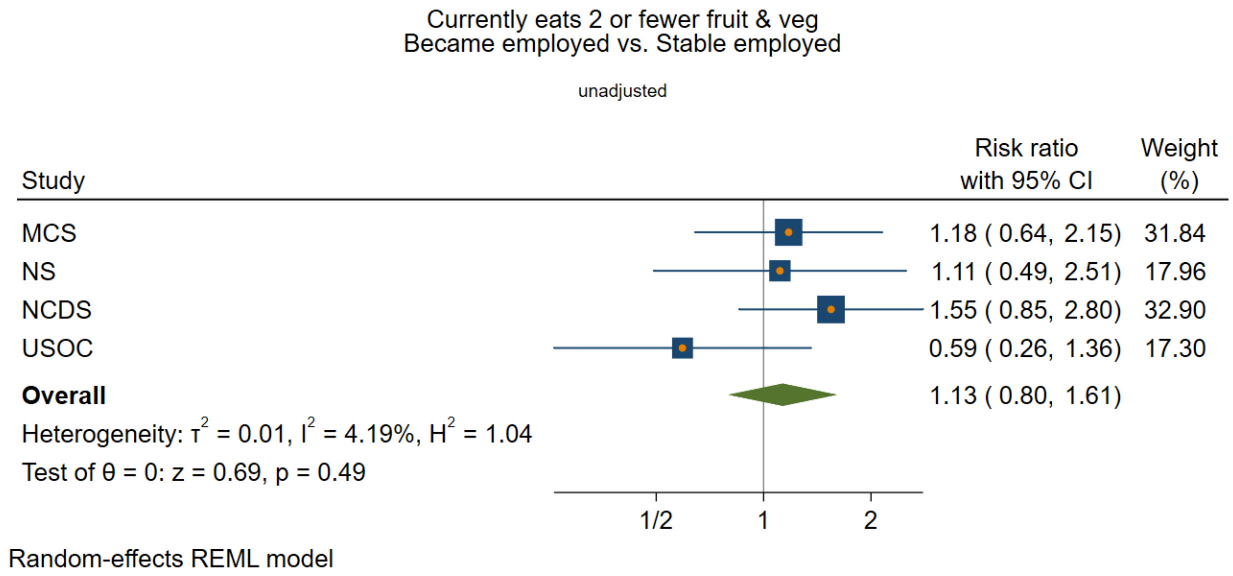
Currently eats 2 or fewer fruit & veg
Became employed vs. Stable employed
unadjusted
Study
Risk ratio with 95% CI
Weight (%)
MCS
1.18 (0.64, 2.15)
31.84
NS
1.11 (0.49, 2.51)
17.96
NCDS
1.55 (0.85, 2.80)
32.90
USOC
0.59 (0.26, 1.36)
17.30
Overall
1.13 (0.80, 1.61)
Heterogeneity: τ^2 = 0.01, I^2 = 4.19%, H^2 = 1.04
Test of θ = 0: z = 0.69, p = 0.49
1/2
1
2
Random-effects REML model

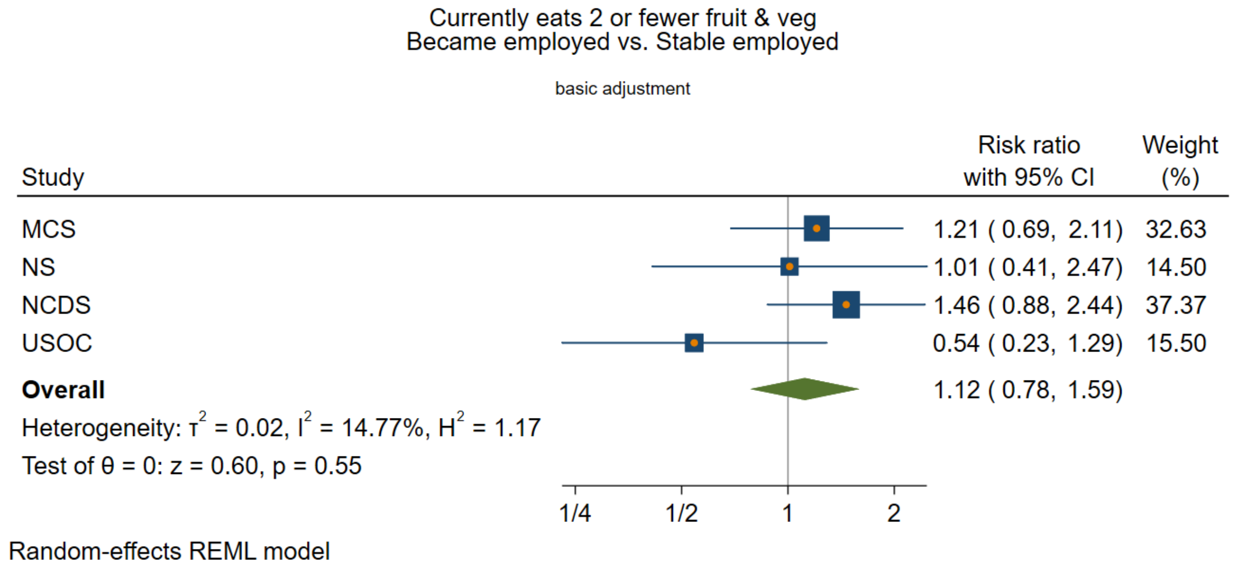
Currently eats 2 or fewer fruit & veg
Became employed vs. Stable employed
basic adjustment
Study
Risk ratio with 95% CI
Weight (%)
MCS
1.21 (0.69, 2.11)
32.63
NS
1.01 (0.41, 2.47)
14.50
NCDS
1.46 (0.88, 2.44)
37.37
USOC
0.54 (0.23, 1.29)
15.50
Overall
1.12 (0.78, 1.59)
Heterogeneity: τ^2 = 0.02, I^2 = 14.77%, H^2 = 1.17
Test of θ = 0: z = 0.60, p = 0.55
1/4
1/2
1
2
Random-effects REML model

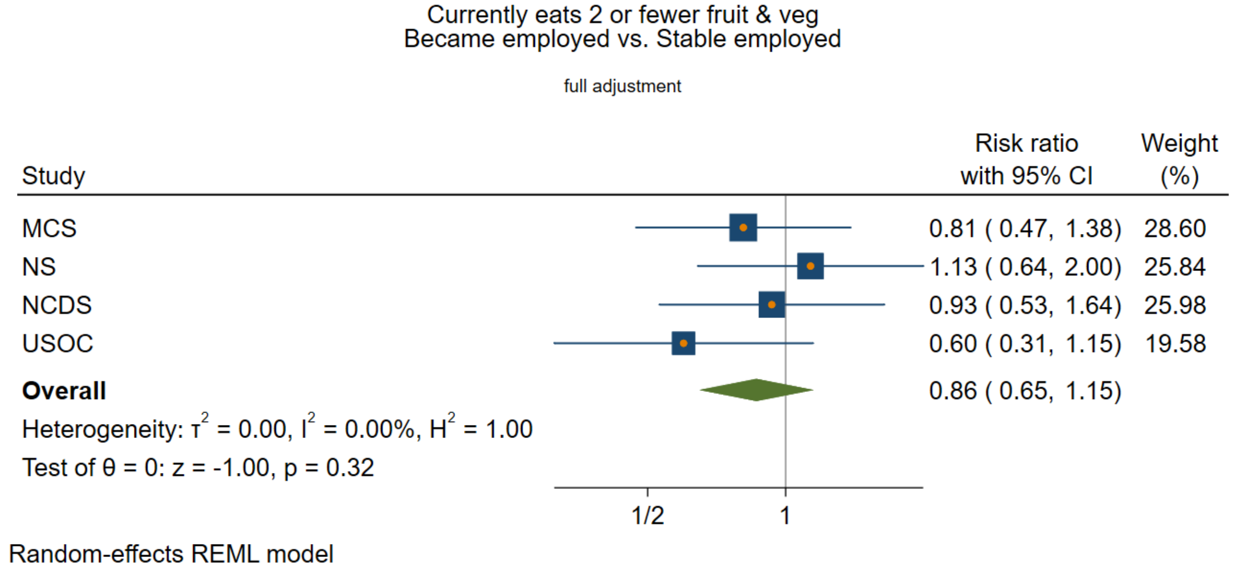
Currently eats 2 or fewer fruit & veg
Became employed vs. Stable employed
full adjustment
Study
Risk ratio with 95% CI
Weight (%)
MCS
0.81 (0.47, 1.38)
28.60
NS
1.13 (0.64, 2.00)
25.84
NCDS
0.93 (0.53, 1.64)
25.98
USOC
0.60 (0.31, 1.15)
19.58
Overall
0.86 (0.65, 1.15)
Heterogeneity: τ^2 = 0.00, I^2 = 0.00%, H^2 = 1.00
Test of θ = 0: z = -1.00, p = 0.32
1/2
1
Random-effects REML model

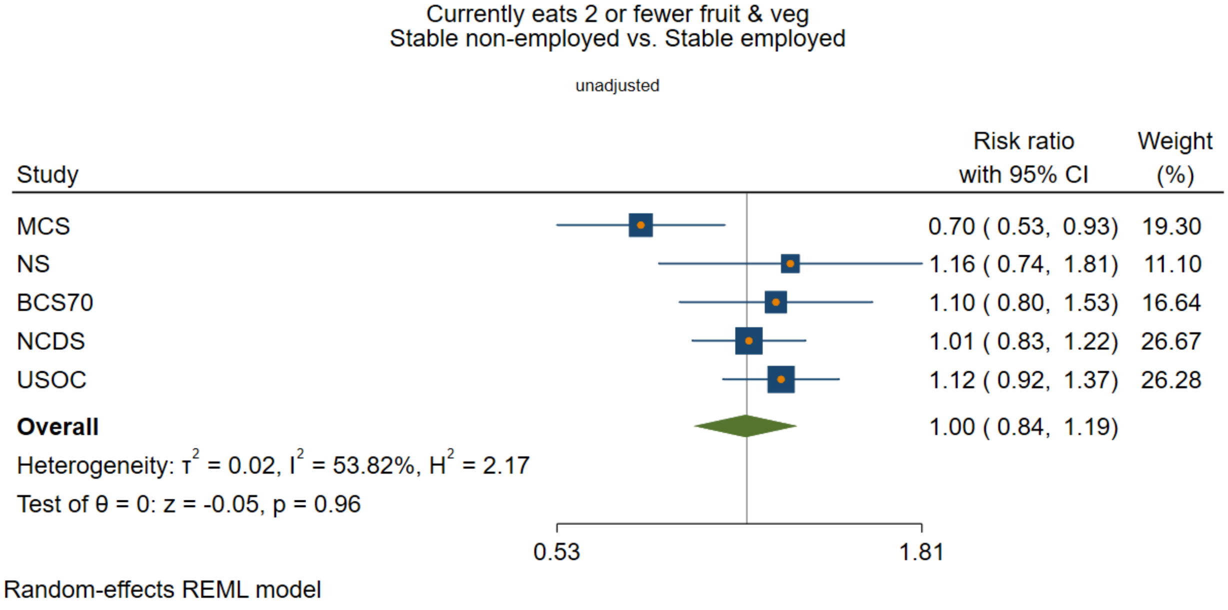
Currently eats 2 or fewer fruit & veg
Stable non-employed vs. Stable employed
unadjusted
Study
Risk ratio with 95% CI
Weight (%)
MCS 0.70 (0.53, 0.93) 19.30
NS 1.16 (0.74, 1.81) 11.10
BCS70 1.10 (0.80, 1.53) 16.64
NCDS 1.01 (0.83, 1.22) 26.67
USOC 1.12 (0.92, 1.37) 26.28
Overall 1.00 (0.84, 1.19)
Heterogeneity: τ^2 = 0.02, I^2 = 53.82%, H^2 = 2.17
Test of θ = 0: z = -0.05, p = 0.96
0.53
1.81
Random-effects REML model

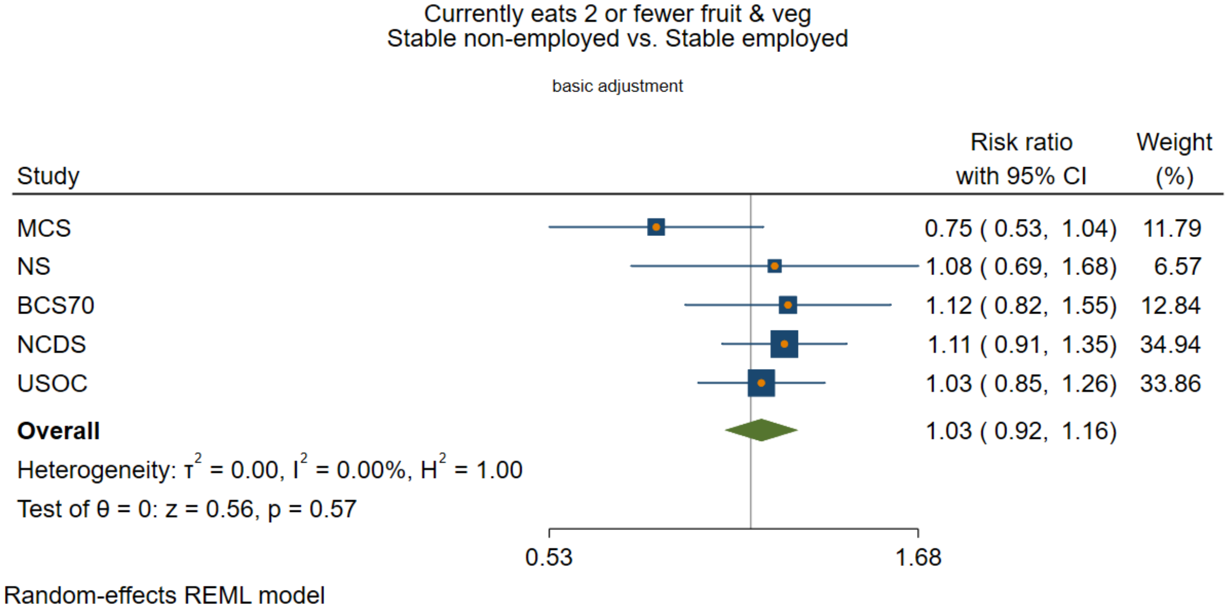
Currently eats 2 or fewer fruit & veg
Stable non-employed vs. Stable employed
basic adjustment
Study
Risk ratio with 95% CI
Weight (%)
MCS 0.75 (0.53, 1.04) 11.79
NS 1.08 (0.69, 1.68) 6.57
BCS70 1.12 (0.82, 1.55) 12.84
NCDS 1.11 (0.91, 1.35) 34.94
USOC 1.03 (0.85, 1.26) 33.86
Overall 1.03 (0.92, 1.16)
Heterogeneity: τ^2 = 0.00, I^2 = 0.00%, H^2 = 1.00
Test of θ = 0: z = 0.56, p = 0.57
0.53
1.68
Random-effects REML model

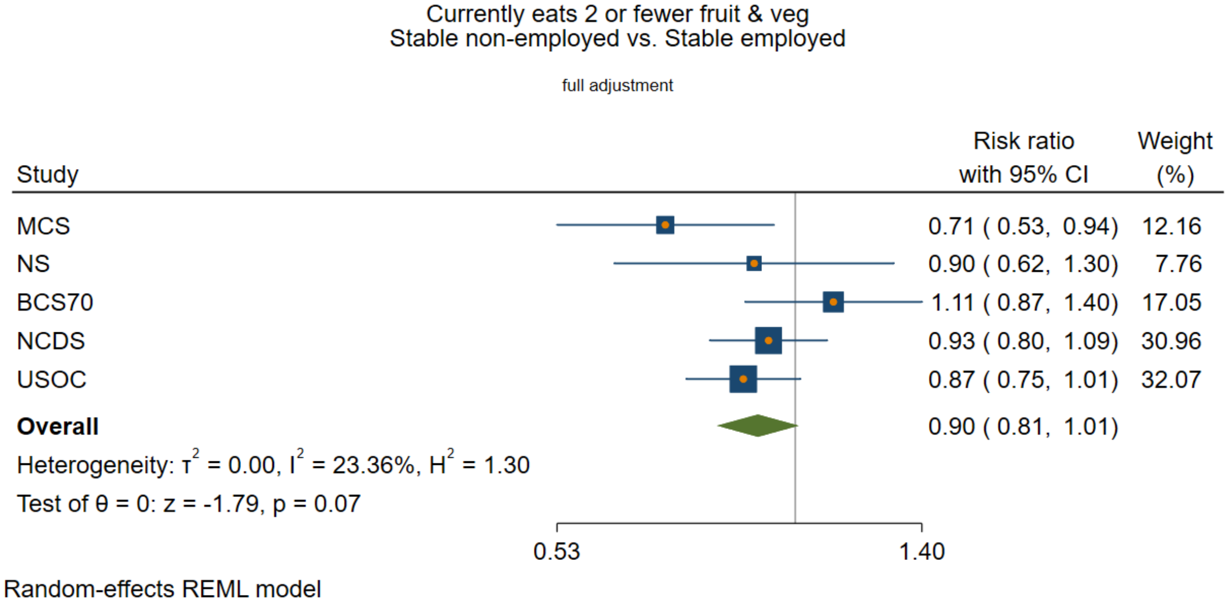
Currently eats 2 or fewer fruit & veg
Stable non-employed vs. Stable employed
full adjustment
Study
Risk ratio with 95% CI
Weight (%)
MCS 0.71 (0.53, 0.94) 12.16
NS 0.90 (0.62, 1.30) 7.76
BCS70 1.11 (0.87, 1.40) 17.05
NCDS 0.93 (0.80, 1.09) 30.96
USOC 0.87 (0.75, 1.01) 32.07
Overall 0.90 (0.81, 1.01)
Heterogeneity: τ^2 = 0.00, I^2 = 23.36%, H^2 = 1.30
Test of θ = 0: z = -1.79, p = 0.07
0.53
1.40
Random-effects REML model

Figure set 2: Fewer fruit and vegetables

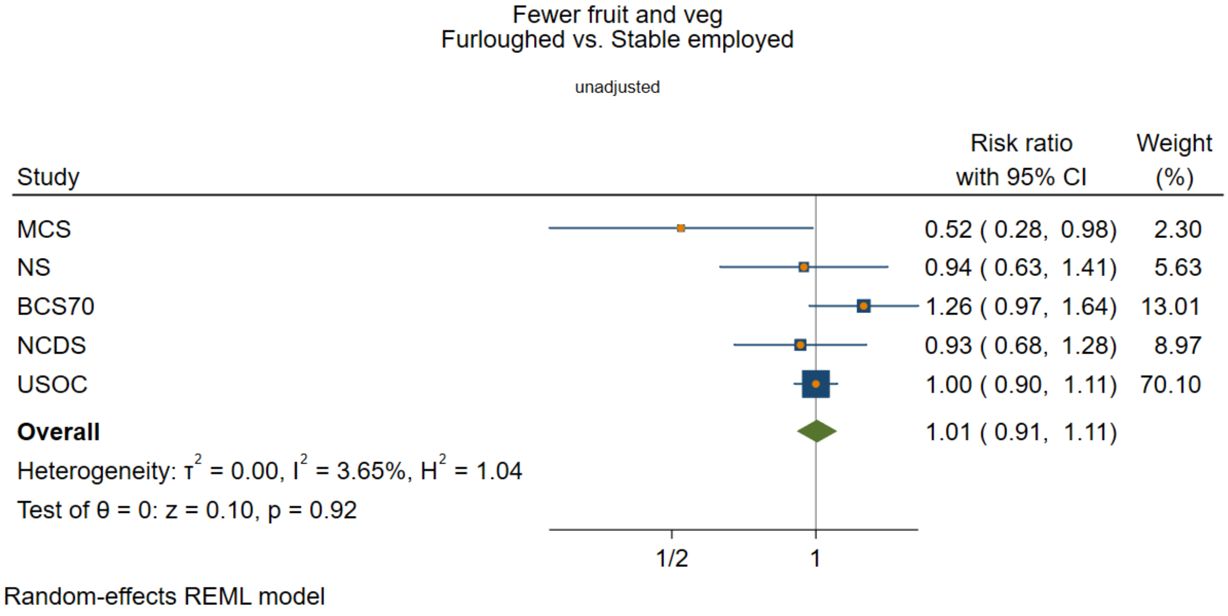

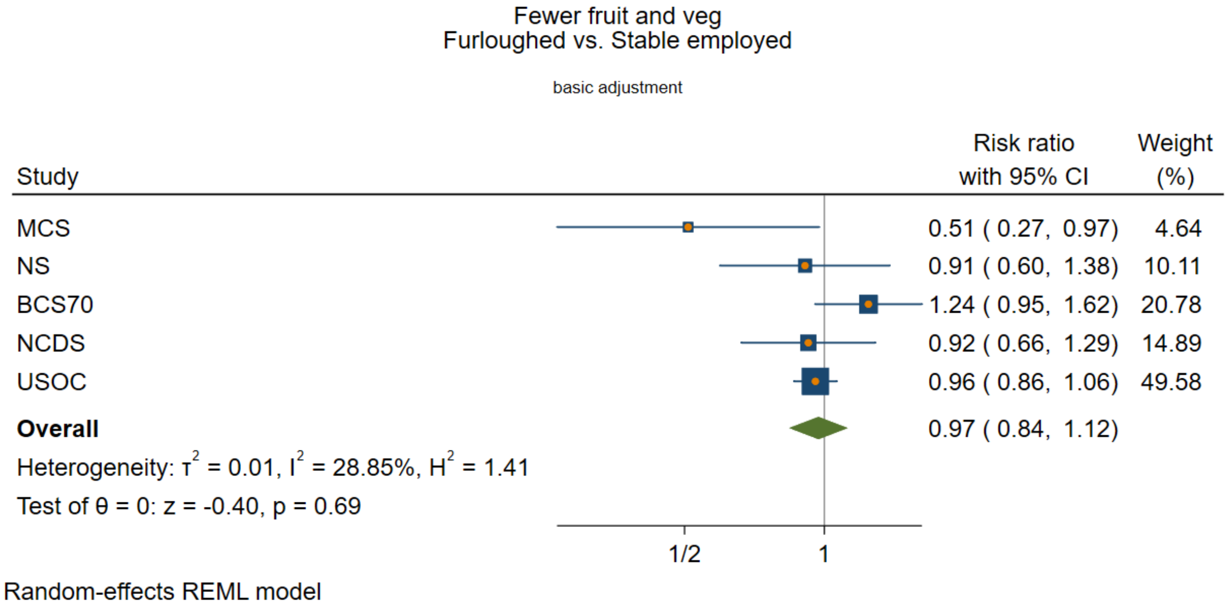

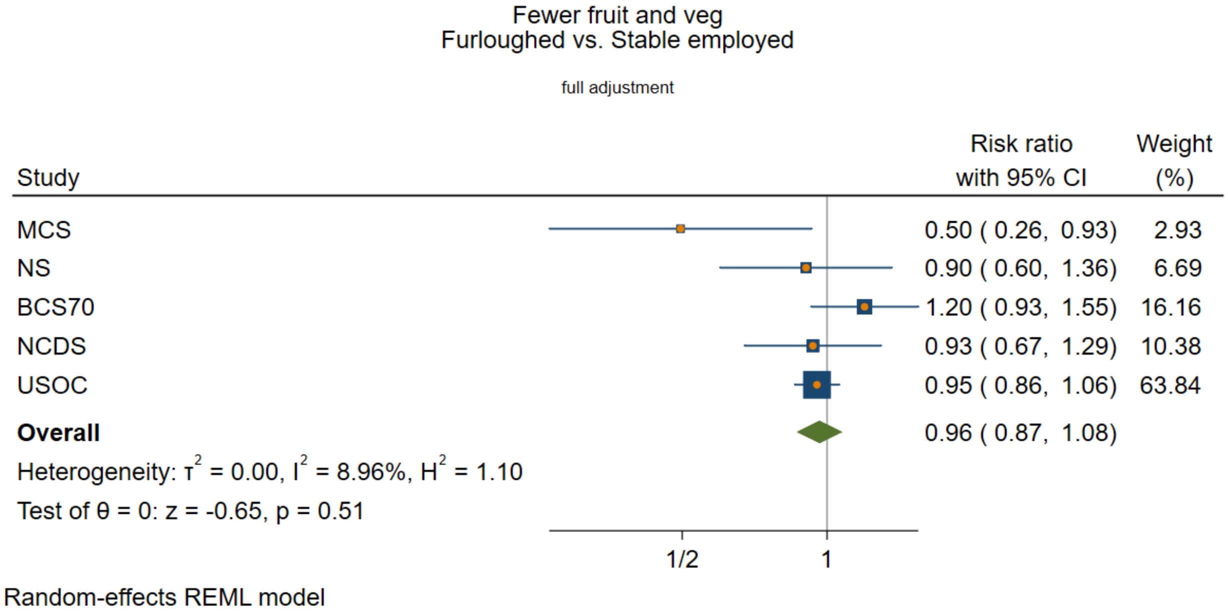

Fewer fruit and veg
No longer employed vs. Stable employed

unadjusted

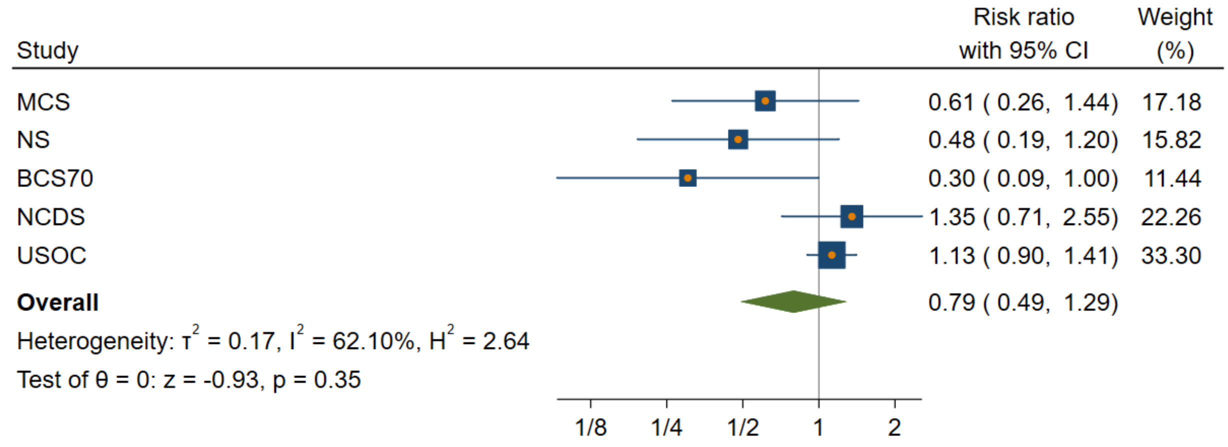

Random-effects REML model

Fewer fruit and veg
No longer employed vs. Stable employed

basic adjustment

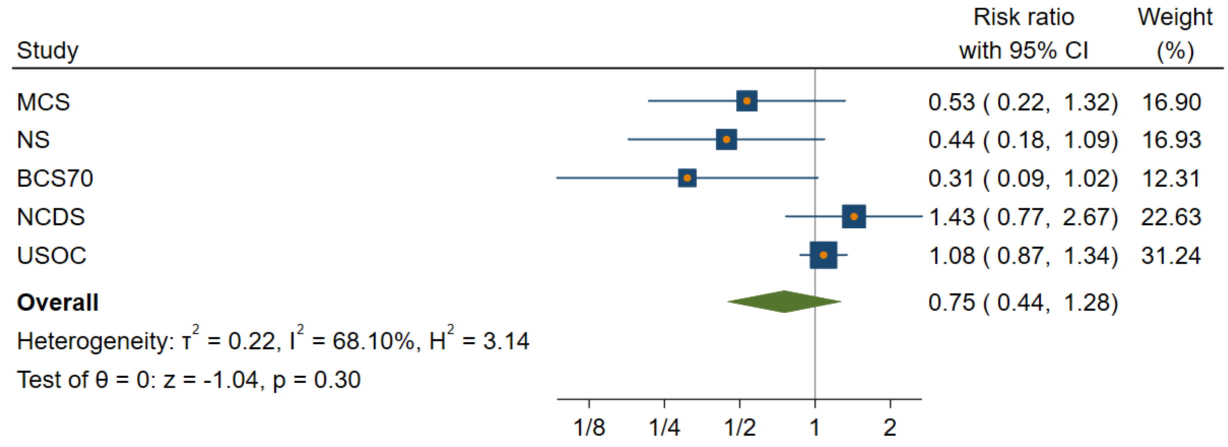

Random-effects REML model

Fewer fruit and veg
No longer employed vs. Stable employed

full adjustment

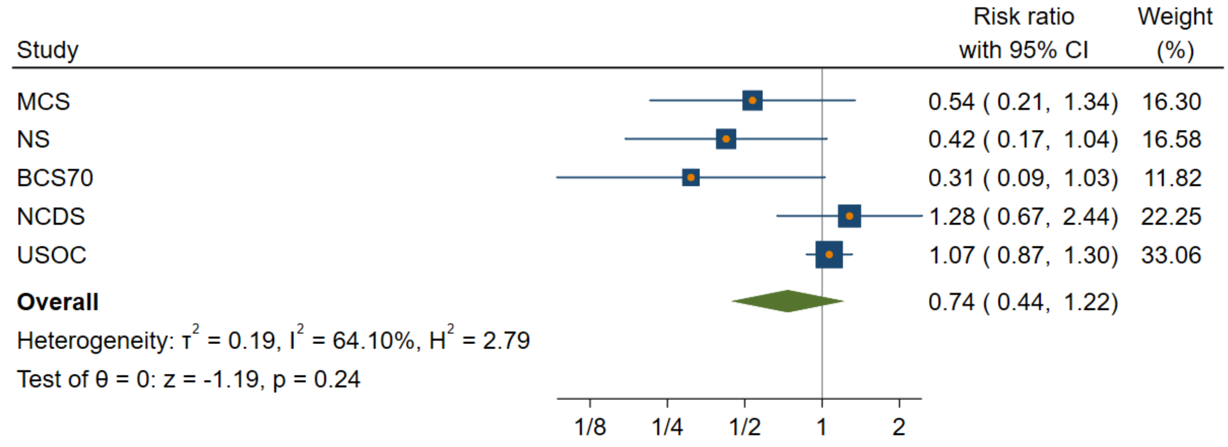

Random-effects REML model

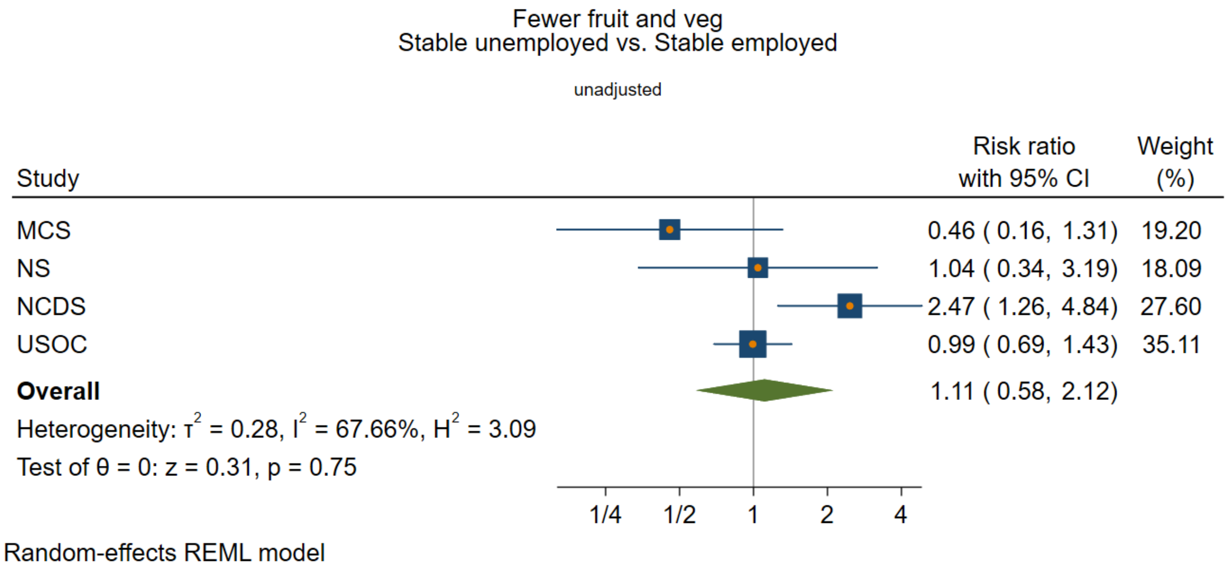
Fewer fruit and veg
Stable unemployed vs. Stable employed
unadjusted
Study
Risk ratio with 95% CI
Weight (%)
MCS 0.46 (0.16, 1.31) 19.20
NS 1.04 (0.34, 3.19) 18.09
NCDS 2.47 (1.26, 4.84) 27.60
USOC 0.99 (0.69, 1.43) 35.11
Overall 1.11 (0.58, 2.12)
Heterogeneity: τ^2 = 0.28, I^2 = 67.66%, H^2 = 3.09
Test of θ = 0: z = 0.31, p = 0.75
1/4
1/2
1
2
4
Random-effects REML model

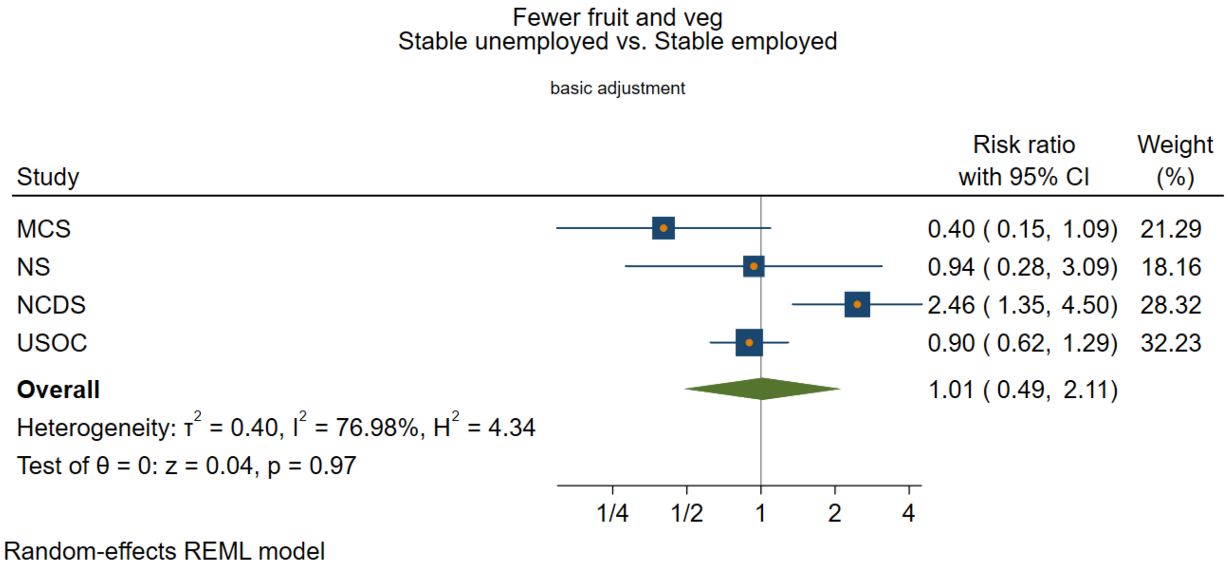
Fewer fruit and veg
Stable unemployed vs. Stable employed
basic adjustment
Study
Risk ratio with 95% CI
Weight (%)
MCS 0.40 (0.15, 1.09) 21.29
NS 0.94 (0.28, 3.09) 18.16
NCDS 2.46 (1.35, 4.50) 28.32
USOC 0.90 (0.62, 1.29) 32.23
Overall 1.01 (0.49, 2.11)
Heterogeneity: τ^2 = 0.40, I^2 = 76.98%, H^2 = 4.34
Test of θ = 0: z = 0.04, p = 0.97
1/4
1/2
1
2
4
Random-effects REML model

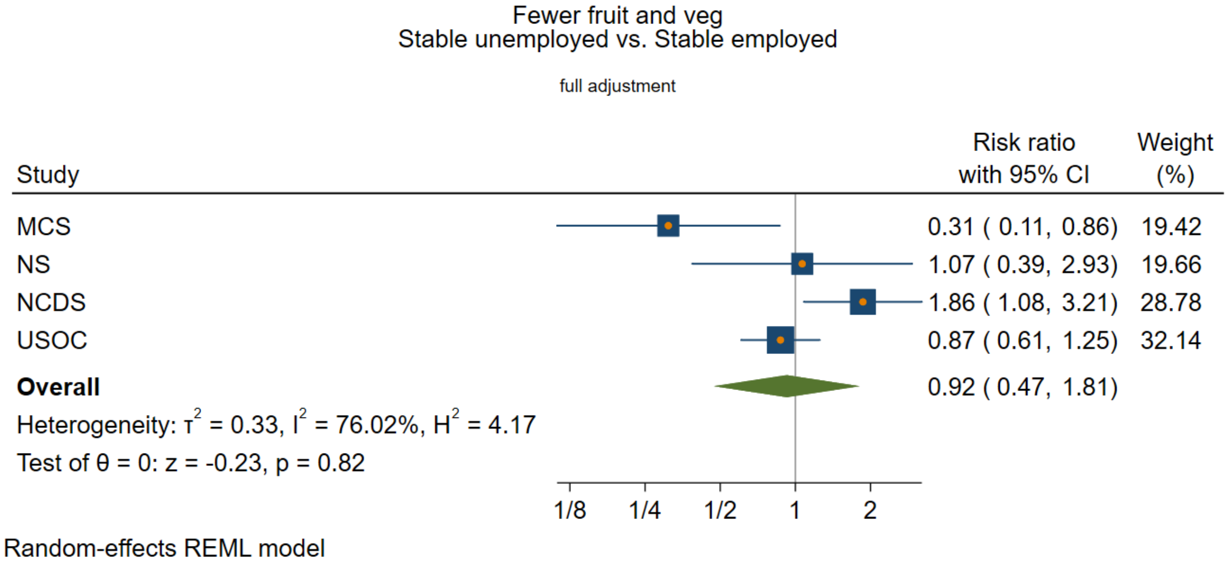
Fewer fruit and veg
Stable unemployed vs. Stable employed
full adjustment
Study
Risk ratio with 95% CI
Weight (%)
MCS 0.31 (0.11, 0.86) 19.42
NS 1.07 (0.39, 2.93) 19.66
NCDS 1.86 (1.08, 3.21) 28.78
USOC 0.87 (0.61, 1.25) 32.14
Overall 0.92 (0.47, 1.81)
Heterogeneity: τ^2 = 0.33, I^2 = 76.02%, H^2 = 4.17
Test of θ = 0: z = -0.23, p = 0.82
1/8
1/4
1/2
1
2
Random-effects REML model

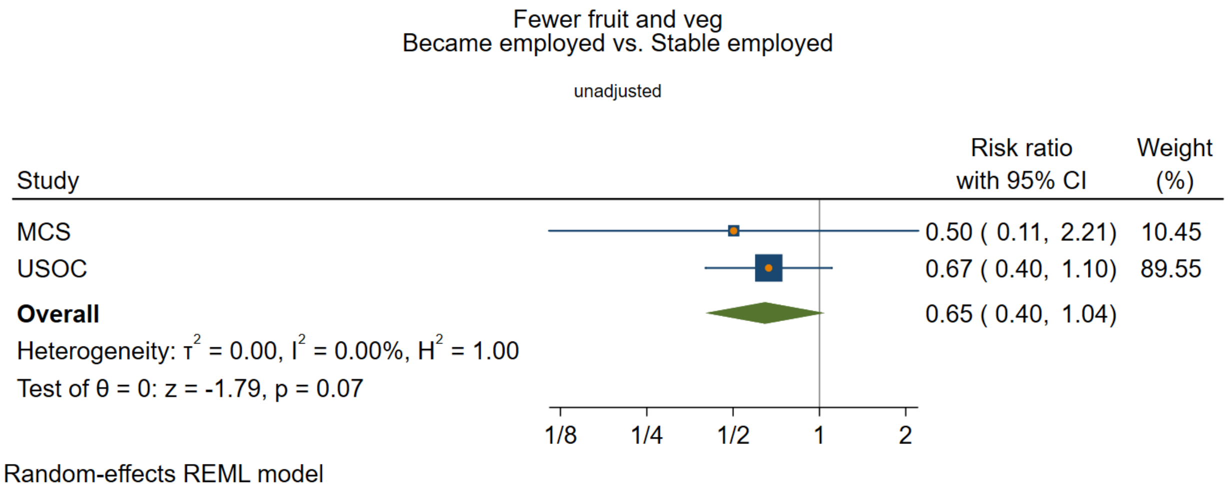
Fewer fruit and veg
Became employed vs. Stable employed
unadjusted
Study
Risk ratio with 95% CI
Weight (%)
MCS
0.50 (0.11, 2.21)
10.45
USOC
0.67 (0.40, 1.10)
89.55
Overall
0.65 (0.40, 1.04)
Heterogeneity: τ^2 = 0.00, I^2 = 0.00%, H^2 = 1.00
Test of θ = 0: z = -1.79, p = 0.07
1/8
1/4
1/2
1
2
Random-effects REML model

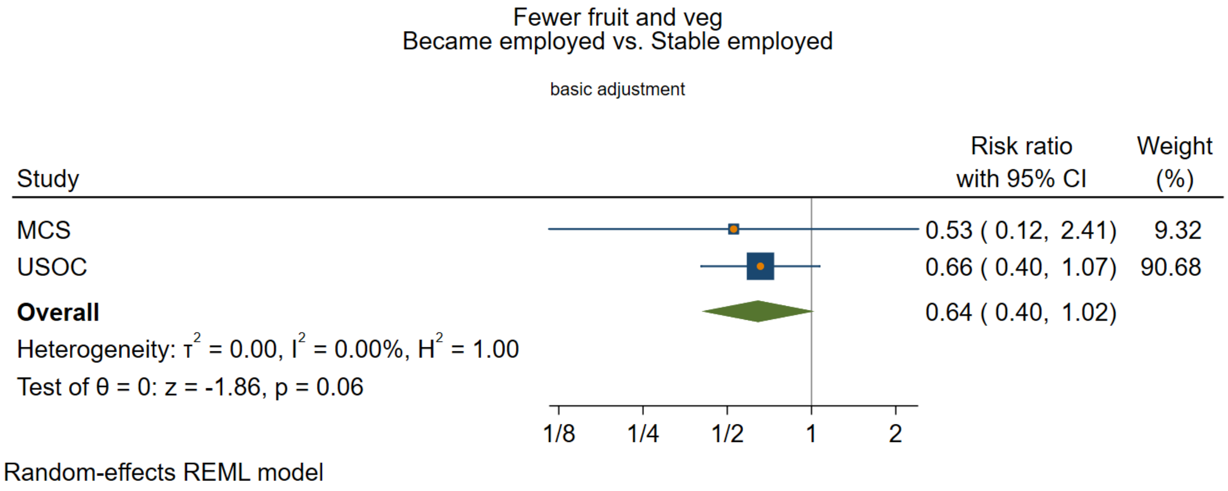
Fewer fruit and veg
Became employed vs. Stable employed
basic adjustment
Study
Risk ratio with 95% CI
Weight (%)
MCS
0.53 (0.12, 2.41)
9.32
USOC
0.66 (0.40, 1.07)
90.68
Overall
0.64 (0.40, 1.02)
Heterogeneity: τ^2 = 0.00, I^2 = 0.00%, H^2 = 1.00
Test of θ = 0: z = -1.86, p = 0.06
1/8
1/4
1/2
1
2
Random-effects REML model

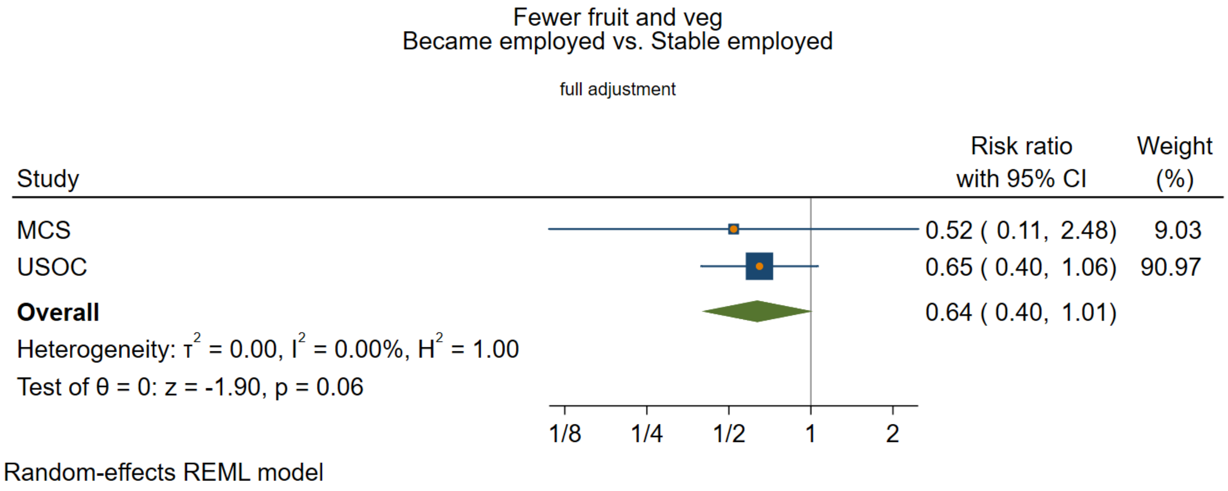
Fewer fruit and veg
Became employed vs. Stable employed
full adjustment
Study
Risk ratio with 95% CI
Weight (%)
MCS
0.52 (0.11, 2.48)
9.03
USOC
0.65 (0.40, 1.06)
90.97
Overall
0.64 (0.40, 1.01)
Heterogeneity: τ^2 = 0.00, I^2 = 0.00%, H^2 = 1.00
Test of θ = 0: z = -1.90, p = 0.06
1/8
1/4
1/2
1
2
Random-effects REML model

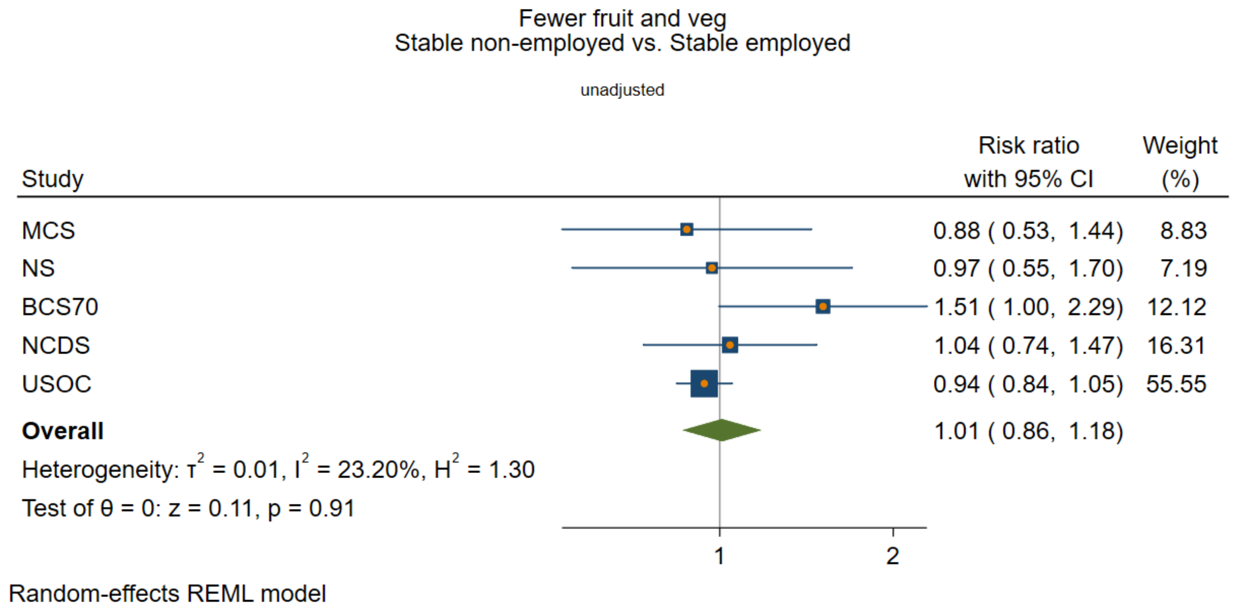
Fewer fruit and veg
Stable non-employed vs. Stable employed
unadjusted
Study
Risk ratio with 95% CI
Weight (%)
MCS 0.88 (0.53, 1.44) 8.83
NS 0.97 (0.55, 1.70) 7.19
BCS70 1.51 (1.00, 2.29) 12.12
NCDS 1.04 (0.74, 1.47) 16.31
USOC 0.94 (0.84, 1.05) 55.55
Overall 1.01 (0.86, 1.18)
Heterogeneity: τ² = 0.01, I² = 23.20%, H² = 1.30
Test of θ = 0: z = 0.11, p = 0.91
1
2
Random-effects REML model

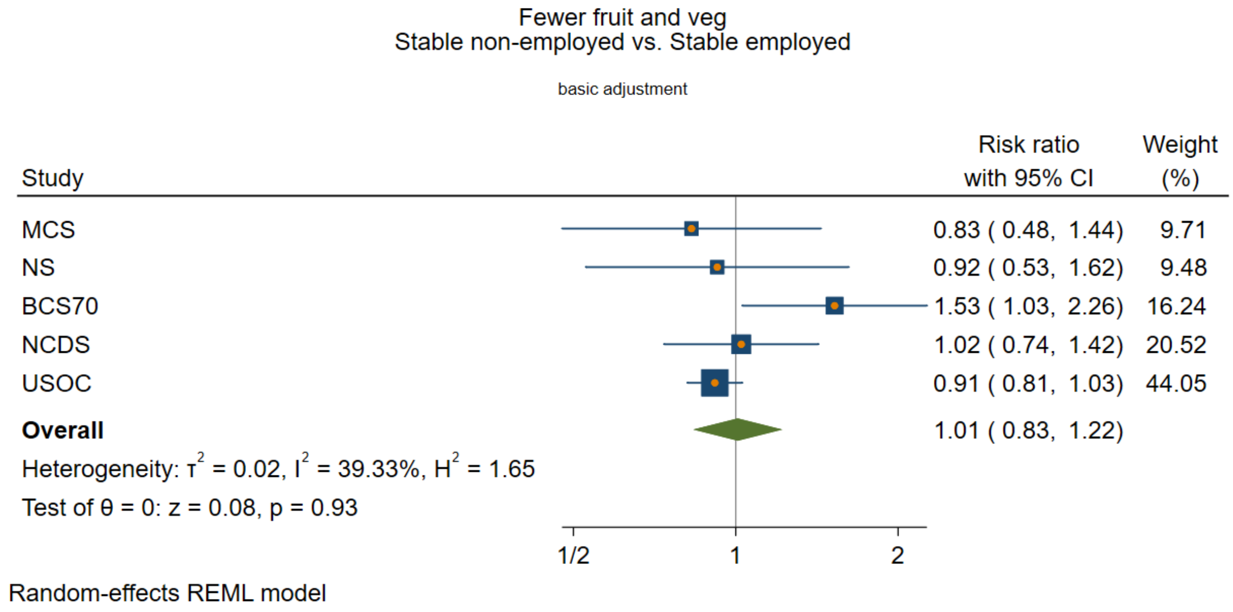
Fewer fruit and veg
Stable non-employed vs. Stable employed
basic adjustment
Study
Risk ratio with 95% CI
Weight (%)
MCS 0.83 (0.48, 1.44) 9.71
NS 0.92 (0.53, 1.62) 9.48
BCS70 1.53 (1.03, 2.26) 16.24
NCDS 1.02 (0.74, 1.42) 20.52
USOC 0.91 (0.81, 1.03) 44.05
Overall 1.01 (0.83, 1.22)
Heterogeneity: τ² = 0.02, I² = 39.33%, H² = 1.65
Test of θ = 0: z = 0.08, p = 0.93
1/2
1
2
Random-effects REML model

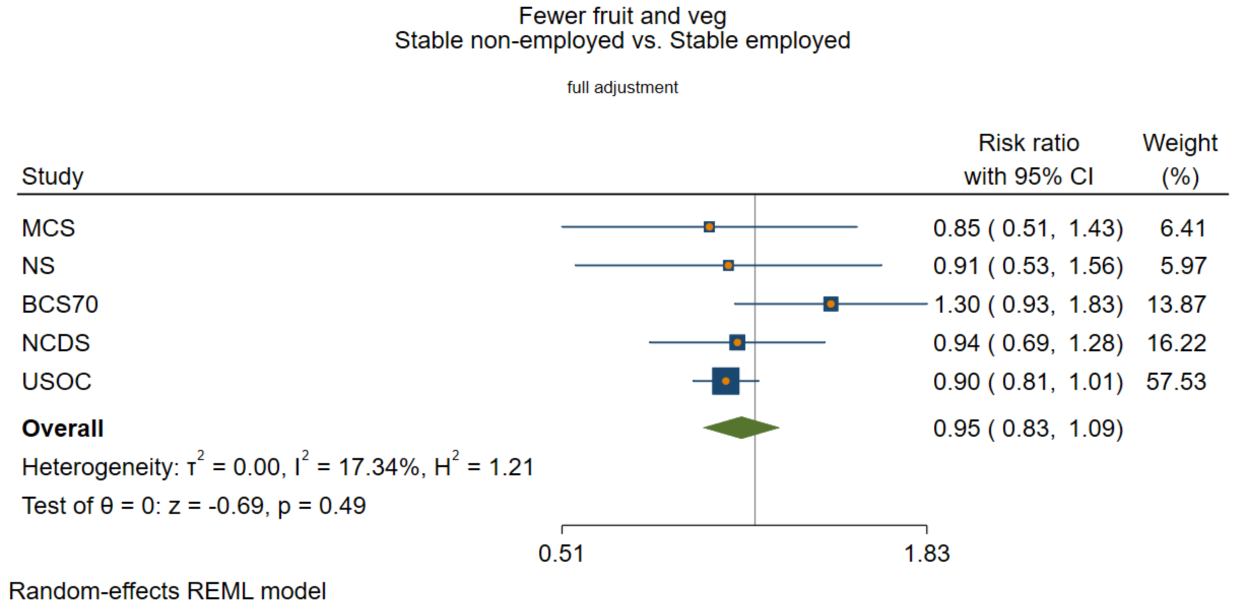
Fewer fruit and veg
Stable non-employed vs. Stable employed
full adjustment
Study
Risk ratio with 95% CI
Weight (%)
MCS 0.85 (0.51, 1.43) 6.41
NS 0.91 (0.53, 1.56) 5.97
BCS70 1.30 (0.93, 1.83) 13.87
NCDS 0.94 (0.69, 1.28) 16.22
USOC 0.90 (0.81, 1.01) 57.53
Overall 0.95 (0.83, 1.09)
Heterogeneity: τ² = 0.00, I² = 17.34%, H² = 1.21
Test of θ = 0: z = -0.69, p = 0.49
0.51
1.83
Random-effects REML model

Figure set 3: More fruit and vegetables

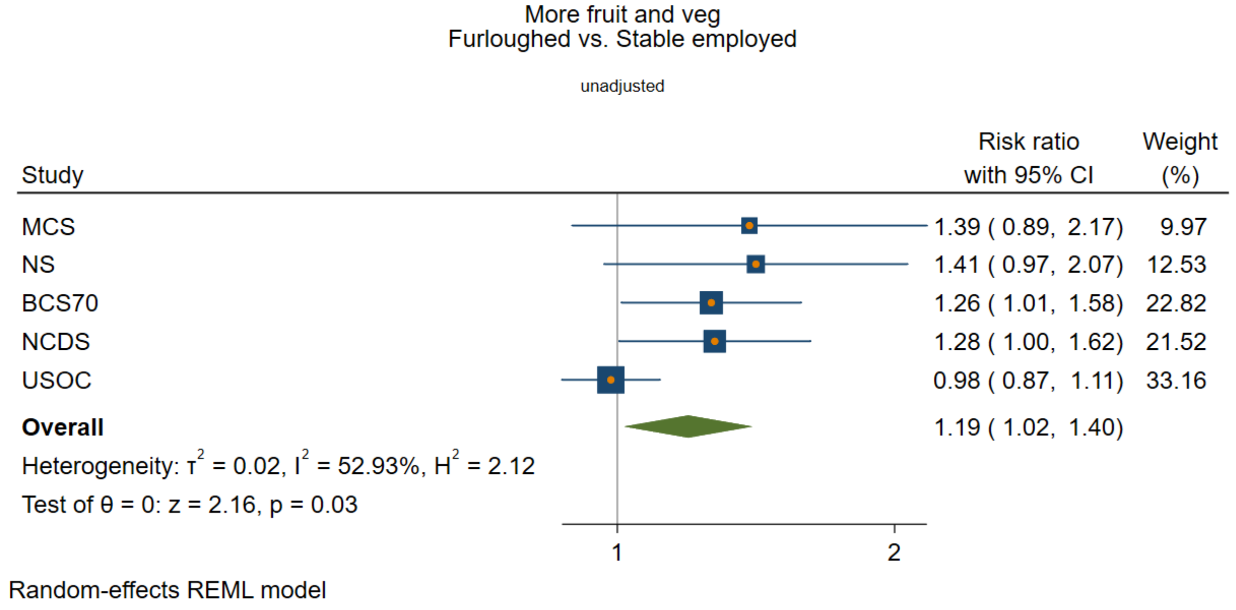

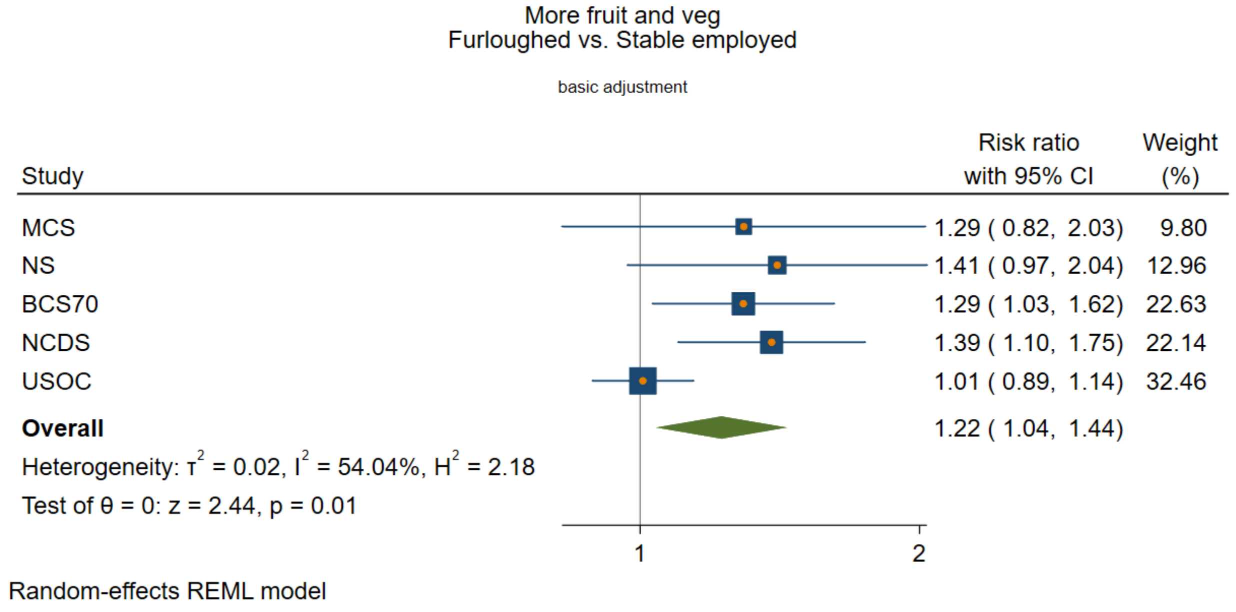

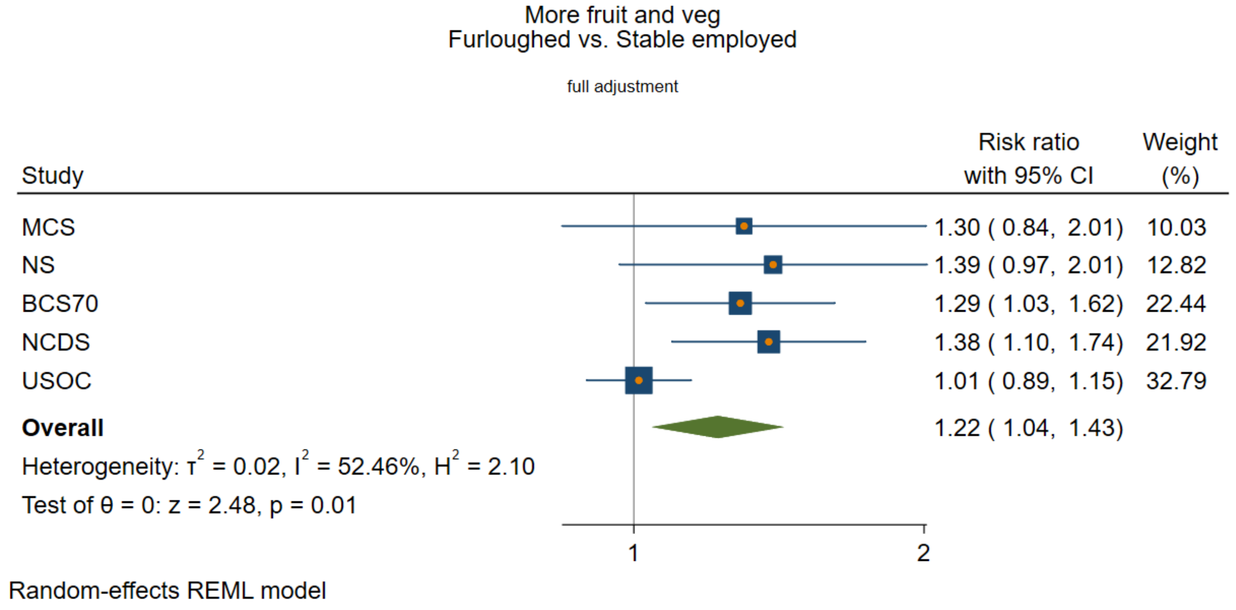

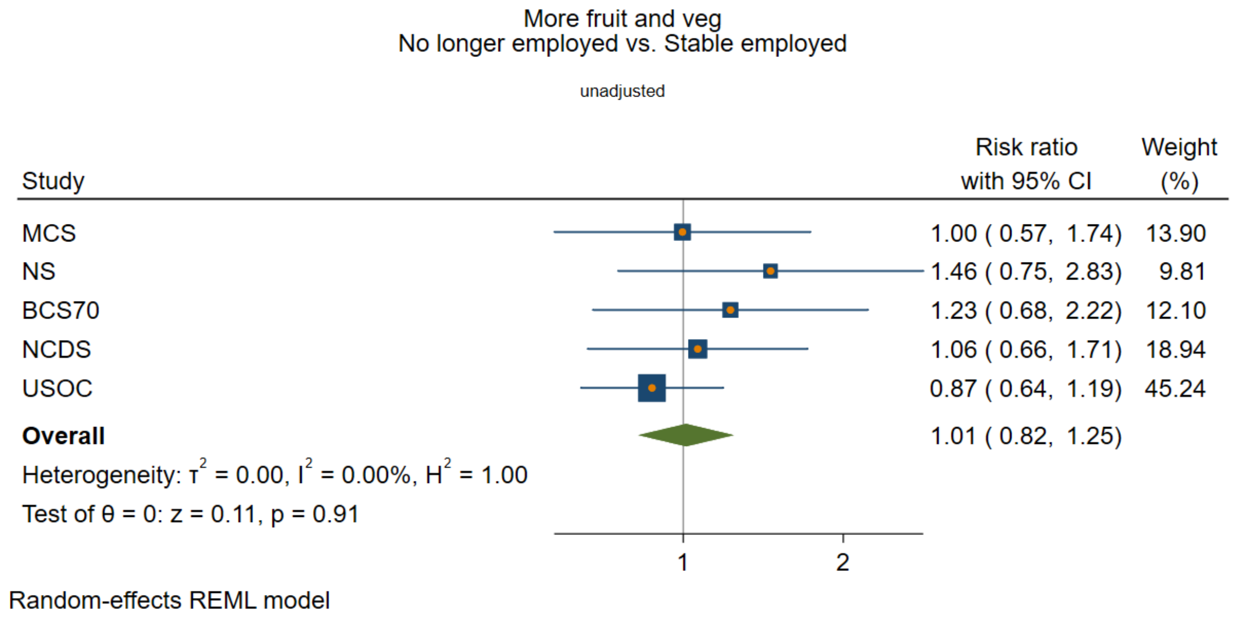
More fruit and veg
No longer employed vs. Stable employed
unadjusted
Study
Risk ratio with 95% CI
Weight (%)
MCS 1.00 (0.57, 1.74) 13.90
NS 1.46 (0.75, 2.83) 9.81
BCS70 1.23 (0.68, 2.22) 12.10
NCDS 1.06 (0.66, 1.71) 18.94
USOC 0.87 (0.64, 1.19) 45.24
Overall 1.01 (0.82, 1.25)
Heterogeneity: τ2 = 0.00, I2 = 0.00%, H2 = 1.00
Test of θ = 0: z = 0.11, p = 0.91
1
2
Random-effects REML model

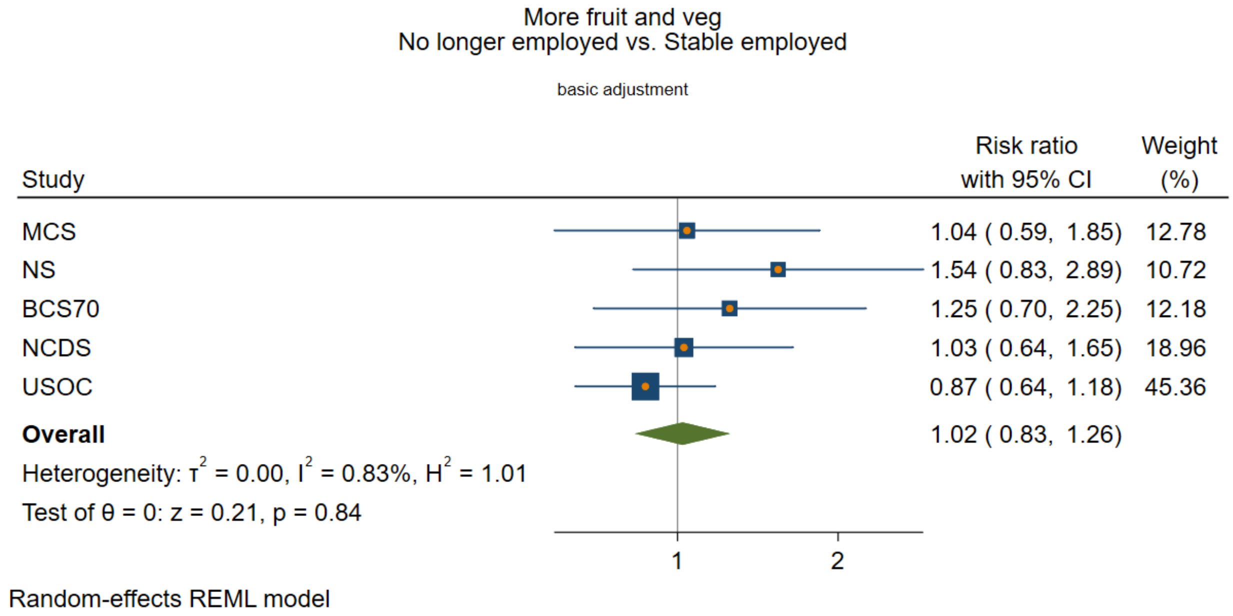
More fruit and veg
No longer employed vs. Stable employed
basic adjustment
Study
Risk ratio with 95% CI
Weight (%)
MCS 1.04 (0.59, 1.85) 12.78
NS 1.54 (0.83, 2.89) 10.72
BCS70 1.25 (0.70, 2.25) 12.18
NCDS 1.03 (0.64, 1.65) 18.96
USOC 0.87 (0.64, 1.18) 45.36
Overall 1.02 (0.83, 1.26)
Heterogeneity: τ2 = 0.00, I2 = 0.83%, H2 = 1.01
Test of θ = 0: z = 0.21, p = 0.84
1
2
Random-effects REML model

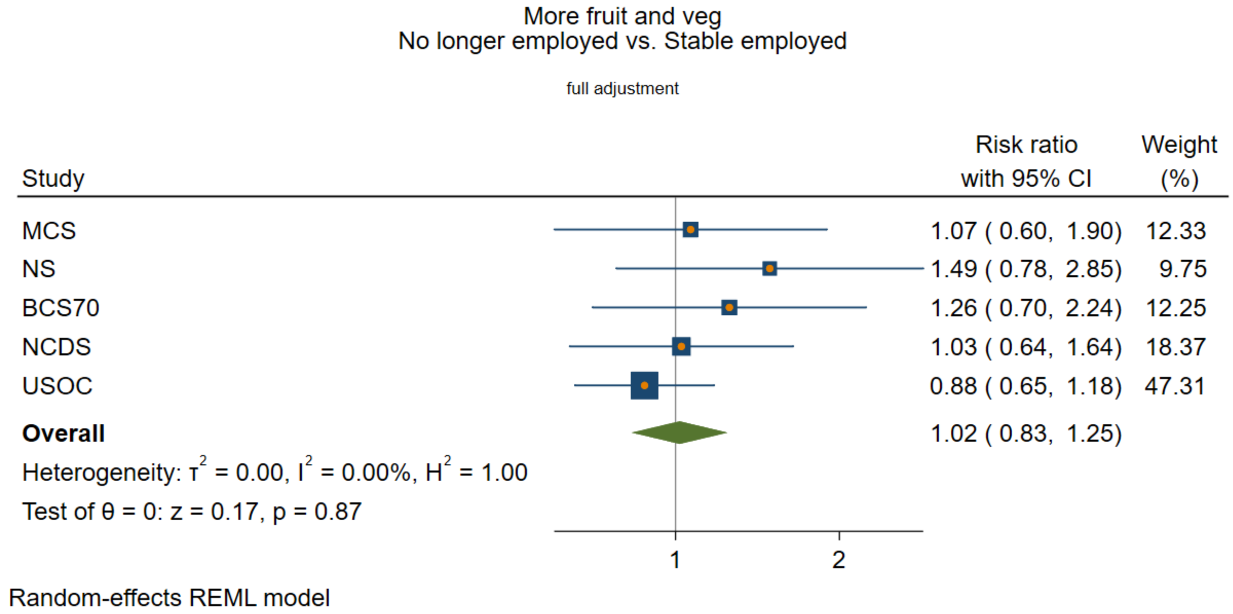
More fruit and veg
No longer employed vs. Stable employed
full adjustment
Study
Risk ratio with 95% CI
Weight (%)
MCS 1.07 (0.60, 1.90) 12.33
NS 1.49 (0.78, 2.85) 9.75
BCS70 1.26 (0.70, 2.24) 12.25
NCDS 1.03 (0.64, 1.64) 18.37
USOC 0.88 (0.65, 1.18) 47.31
Overall 1.02 (0.83, 1.25)
Heterogeneity: τ2 = 0.00, I2 = 0.00%, H2 = 1.00
Test of θ = 0: z = 0.17, p = 0.87
1
2
Random-effects REML model

More fruit and veg
Stable unemployed vs. Stable employed

unadjusted

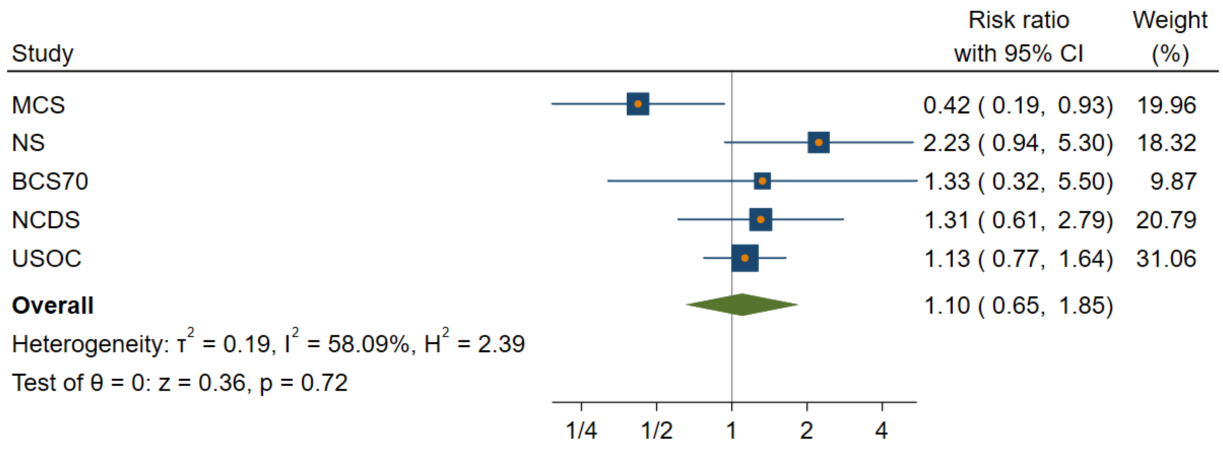

Random-effects REML model

More fruit and veg
Stable unemployed vs. Stable employed

basic adjustment

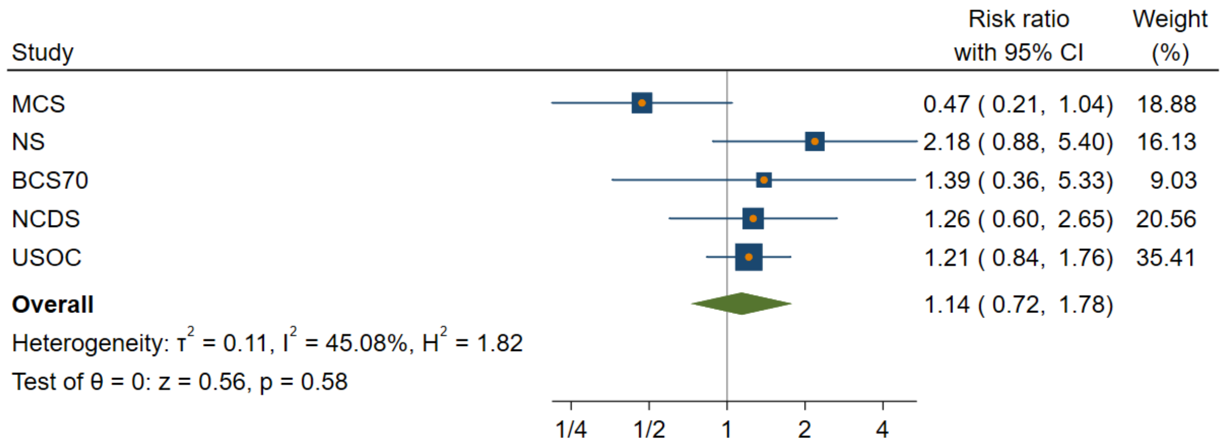

Random-effects REML model

More fruit and veg
Stable unemployed vs. Stable employed

full adjustment

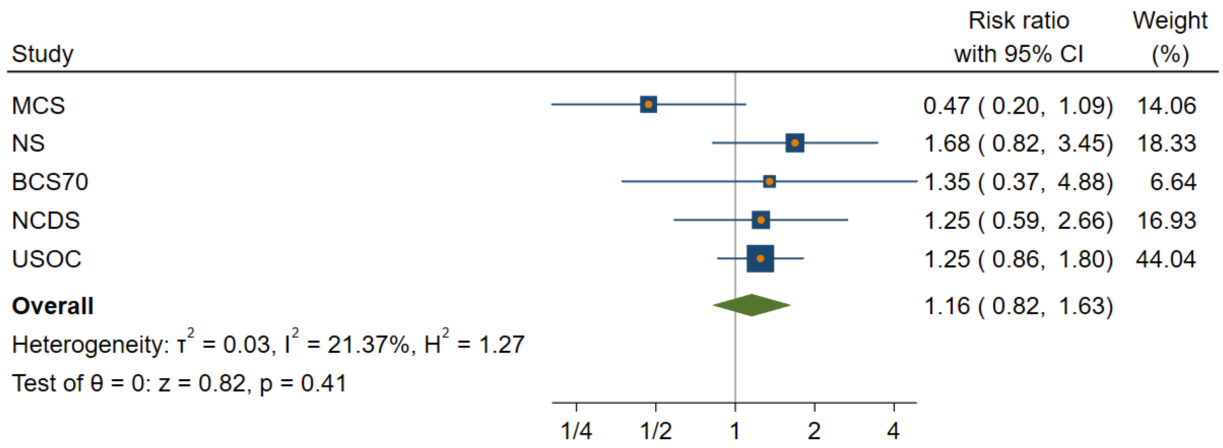

Random-effects REML model

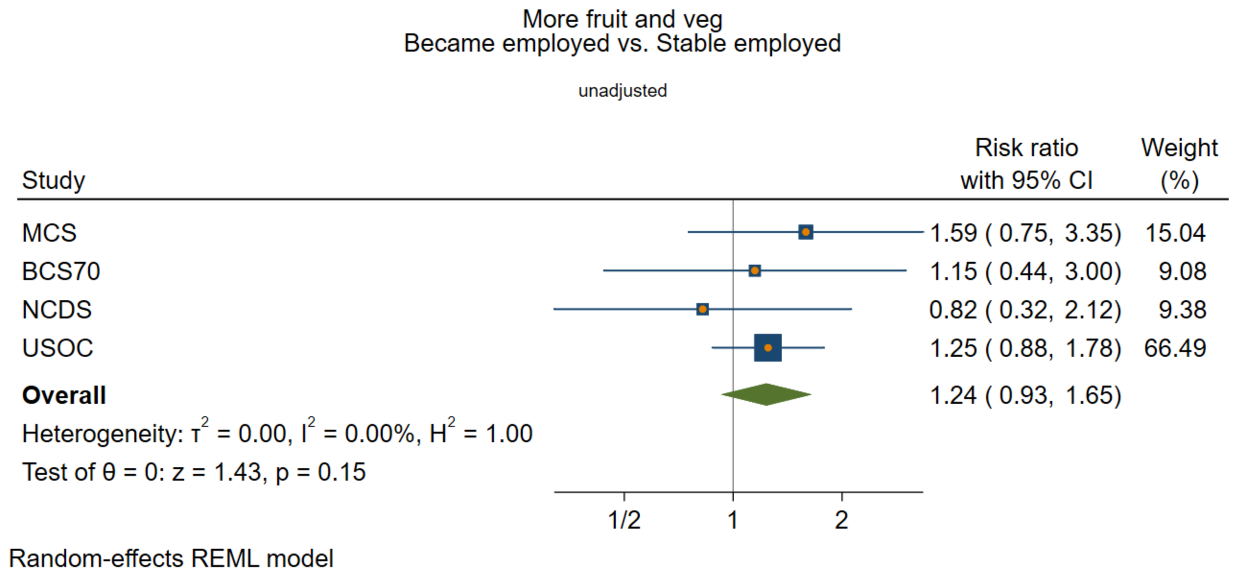
More fruit and veg
Became employed vs. Stable employed
unadjusted
Study
Risk ratio with 95% CI
Weight (%)
MCS 1.59 (0.75, 3.35) 15.04
BCS70 1.15 (0.44, 3.00) 9.08
NCDS 0.82 (0.32, 2.12) 9.38
USOC 1.25 (0.88, 1.78) 66.49
Overall 1.24 (0.93, 1.65)
Heterogeneity: τ² = 0.00, I² = 0.00%, H² = 1.00
Test of θ = 0: z = 1.43, p = 0.15
1/2
1
2
Random-effects REML model

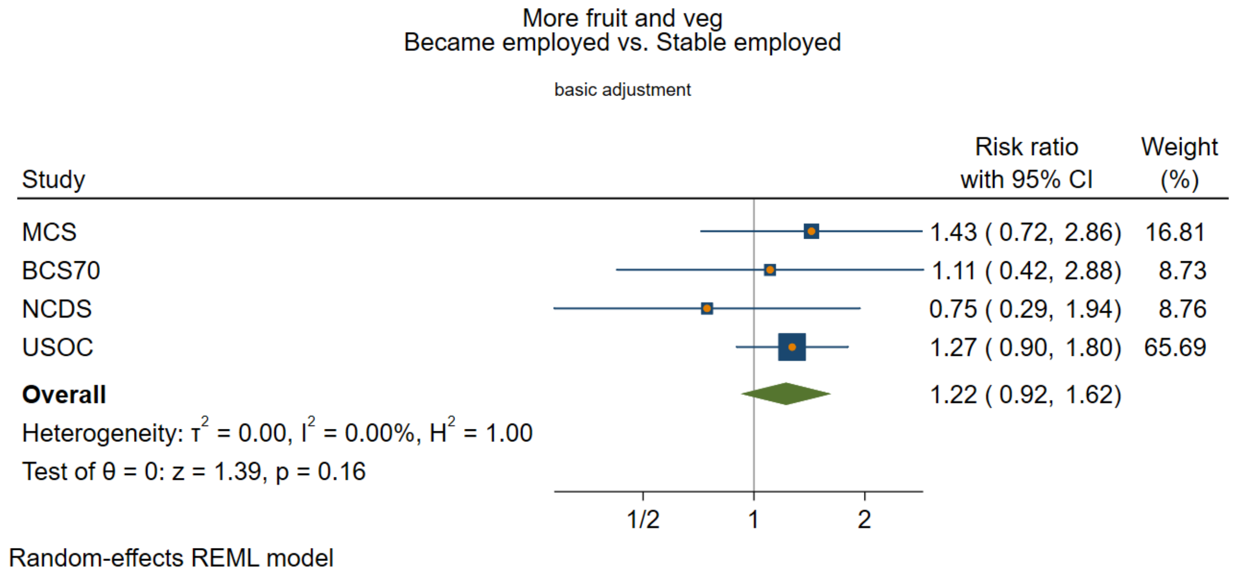
More fruit and veg
Became employed vs. Stable employed
basic adjustment
Study
Risk ratio with 95% CI
Weight (%)
MCS 1.43 (0.72, 2.86) 16.81
BCS70 1.11 (0.42, 2.88) 8.73
NCDS 0.75 (0.29, 1.94) 8.76
USOC 1.27 (0.90, 1.80) 65.69
Overall 1.22 (0.92, 1.62)
Heterogeneity: τ² = 0.00, I² = 0.00%, H² = 1.00
Test of θ = 0: z = 1.39, p = 0.16
1/2
1
2
Random-effects REML model

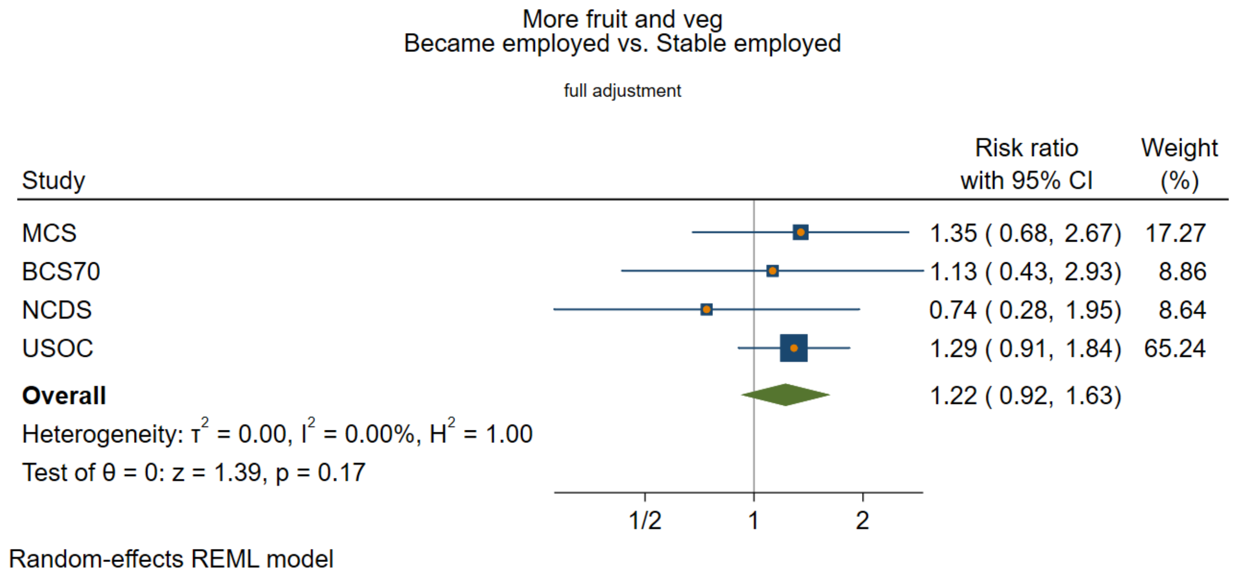
More fruit and veg
Became employed vs. Stable employed
full adjustment
Study
Risk ratio with 95% CI
Weight (%)
MCS 1.35 (0.68, 2.67) 17.27
BCS70 1.13 (0.43, 2.93) 8.86
NCDS 0.74 (0.28, 1.95) 8.64
USOC 1.29 (0.91, 1.84) 65.24
Overall 1.22 (0.92, 1.63)
Heterogeneity: τ² = 0.00, I² = 0.00%, H² = 1.00
Test of θ = 0: z = 1.39, p = 0.17
1/2
1
2
Random-effects REML model

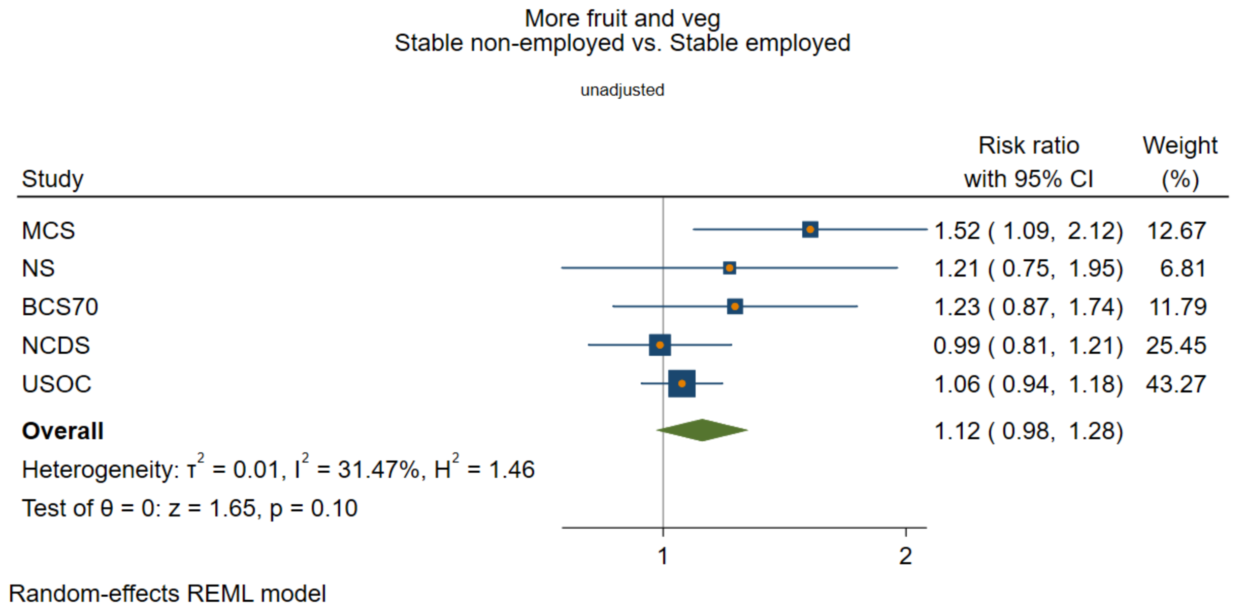
More fruit and veg
Stable non-employed vs. Stable employed
unadjusted
Study
Risk ratio with 95% CI
Weight (%)
MCS 1.52 (1.09, 2.12) 12.67
NS 1.21 (0.75, 1.95) 6.81
BCS70 1.23 (0.87, 1.74) 11.79
NCDS 0.99 (0.81, 1.21) 25.45
USOC 1.06 (0.94, 1.18) 43.27
Overall 1.12 (0.98, 1.28)
Heterogeneity: τ^2 = 0.01, I^2 = 31.47%, H^2 = 1.46
Test of θ = 0: z = 1.65, p = 0.10
1
2
Random-effects REML model

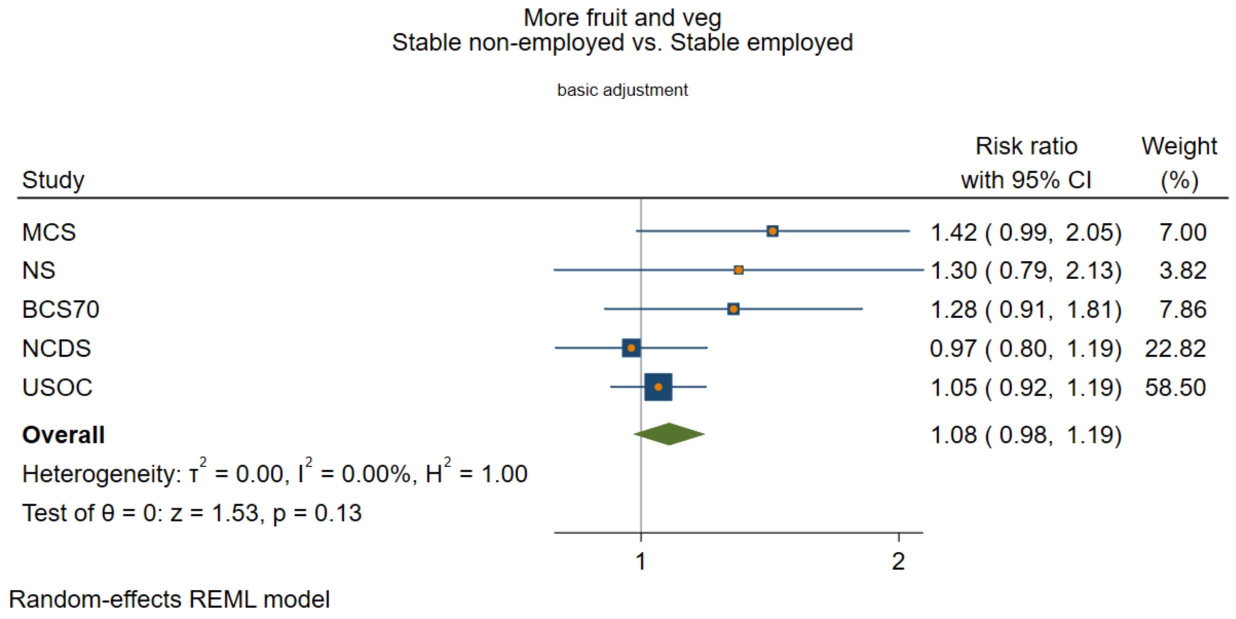
More fruit and veg
Stable non-employed vs. Stable employed
basic adjustment
Study
Risk ratio with 95% CI
Weight (%)
MCS 1.42 (0.99, 2.05) 7.00
NS 1.30 (0.79, 2.13) 3.82
BCS70 1.28 (0.91, 1.81) 7.86
NCDS 0.97 (0.80, 1.19) 22.82
USOC 1.05 (0.92, 1.19) 58.50
Overall 1.08 (0.98, 1.19)
Heterogeneity: τ^2 = 0.00, I^2 = 0.00%, H^2 = 1.00
Test of θ = 0: z = 1.53, p = 0.13
1
2
Random-effects REML model

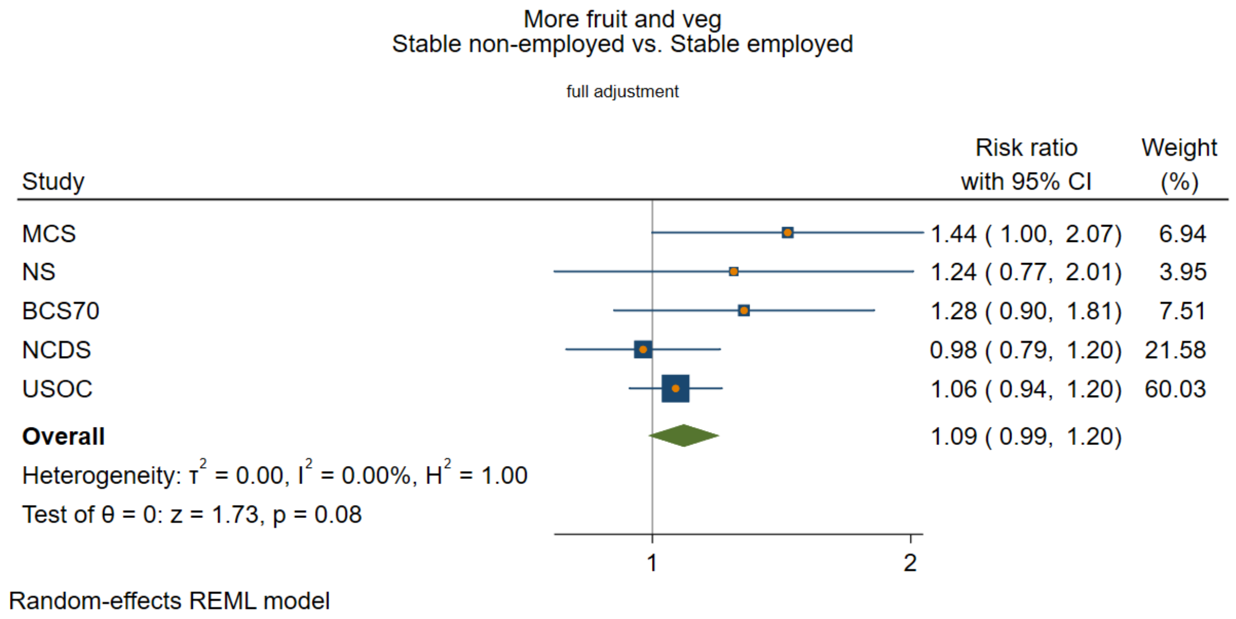
More fruit and veg
Stable non-employed vs. Stable employed
full adjustment
Study
Risk ratio with 95% CI
Weight (%)
MCS 1.44 (1.00, 2.07) 6.94
NS 1.24 (0.77, 2.01) 3.95
BCS70 1.28 (0.90, 1.81) 7.51
NCDS 0.98 (0.79, 1.20) 21.58
USOC 1.06 (0.94, 1.20) 60.03
Overall 1.09 (0.99, 1.20)
Heterogeneity: τ^2 = 0.00, I^2 = 0.00%, H^2 = 1.00
Test of θ = 0: z = 1.73, p = 0.08
1
2
Random-effects REML model

Figure set 4: Less than 3 days a week of at least 30 min exercise

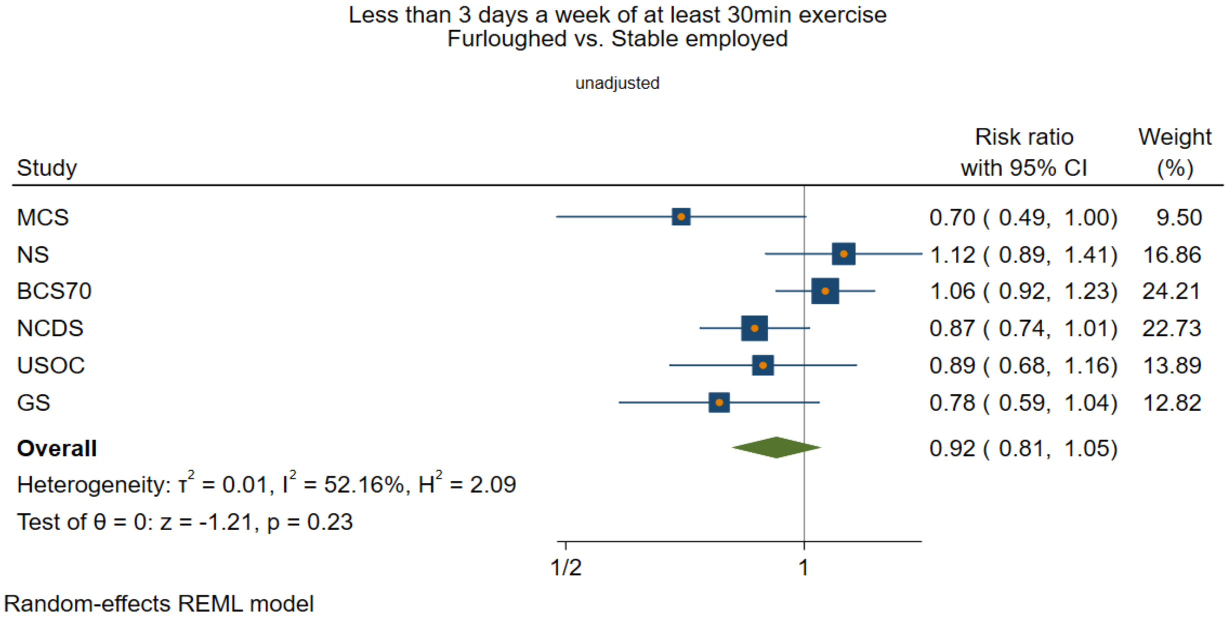

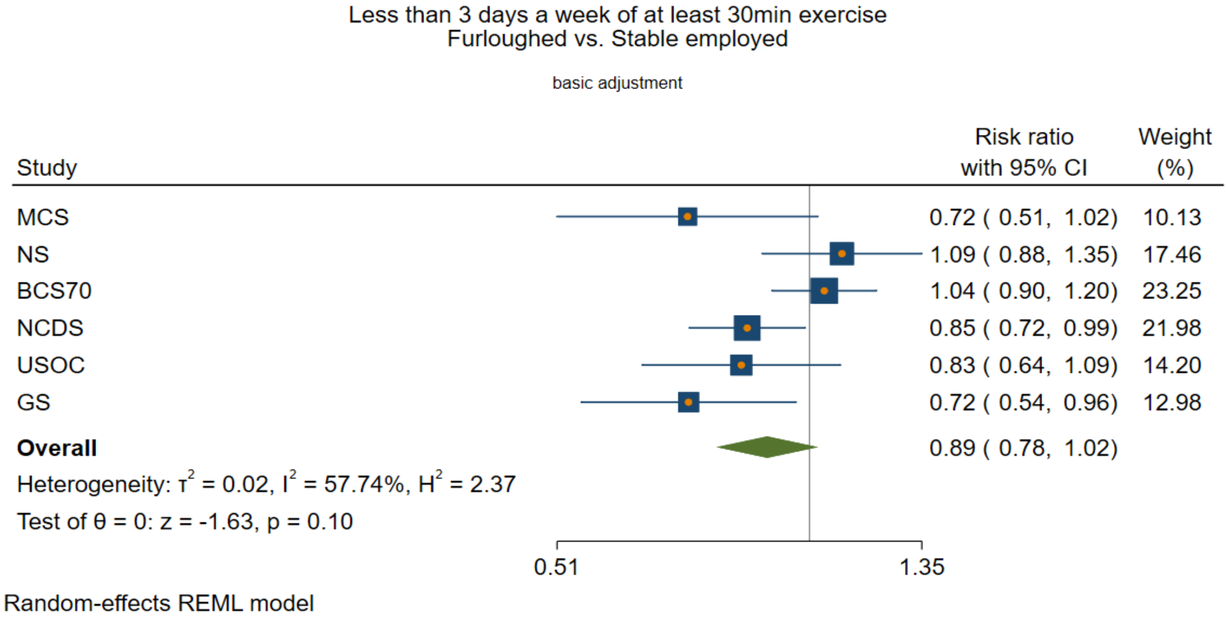

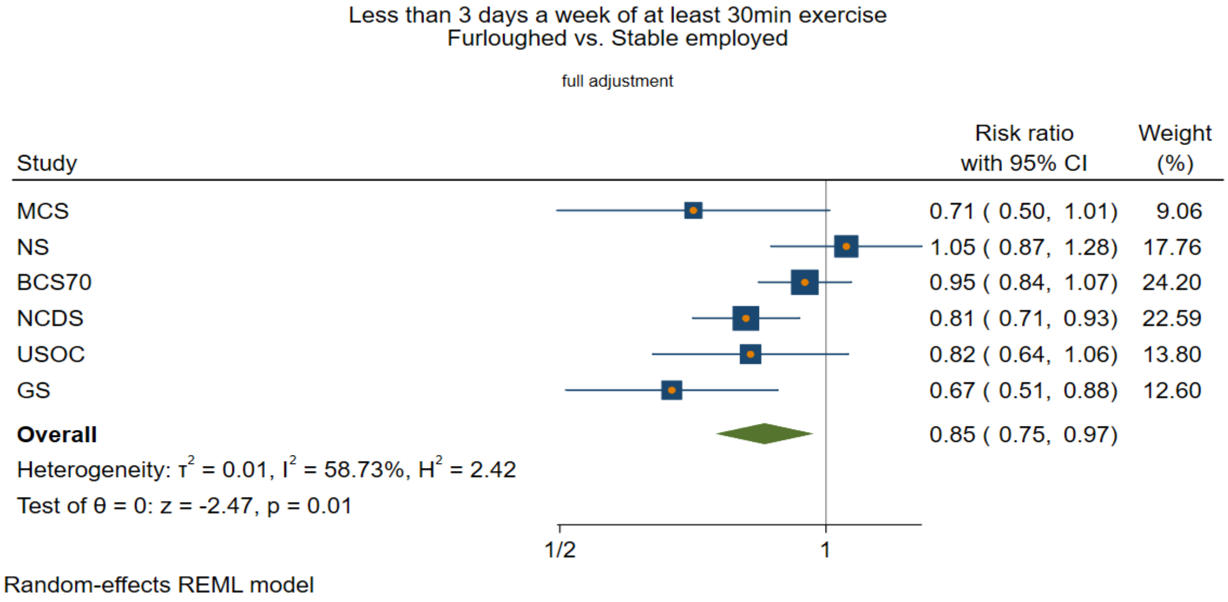

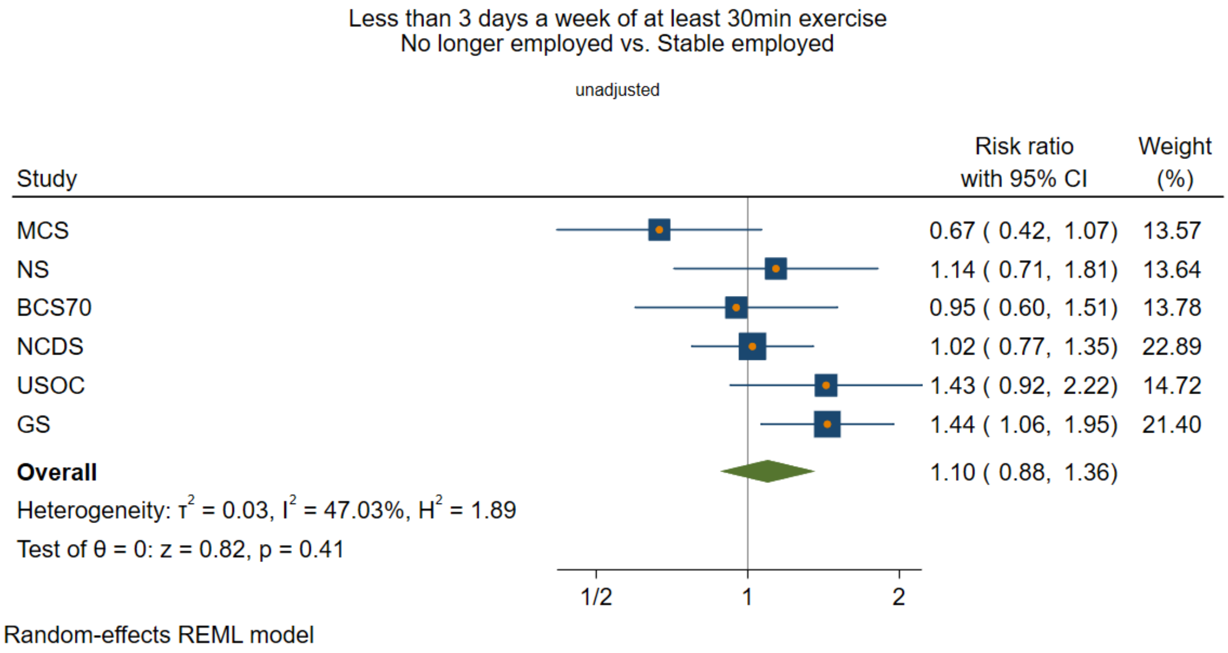
Less than 3 days a week of at least 30min exercise
No longer employed vs. Stable employed
unadjusted
Study
Risk ratio with 95% CI
Weight (%)
MCS 0.67 (0.42, 1.07) 13.57
NS 1.14 (0.71, 1.81) 13.64
BCS70 0.95 (0.60, 1.51) 13.78
NCDS 1.02 (0.77, 1.35) 22.89
USOC 1.43 (0.92, 2.22) 14.72
GS 1.44 (1.06, 1.95) 21.40
Overall 1.10 (0.88, 1.36)
Heterogeneity: τ² = 0.03, I² = 47.03%, H² = 1.89
Test of θ = 0: z = 0.82, p = 0.41
1/2
1
2
Random-effects REML model

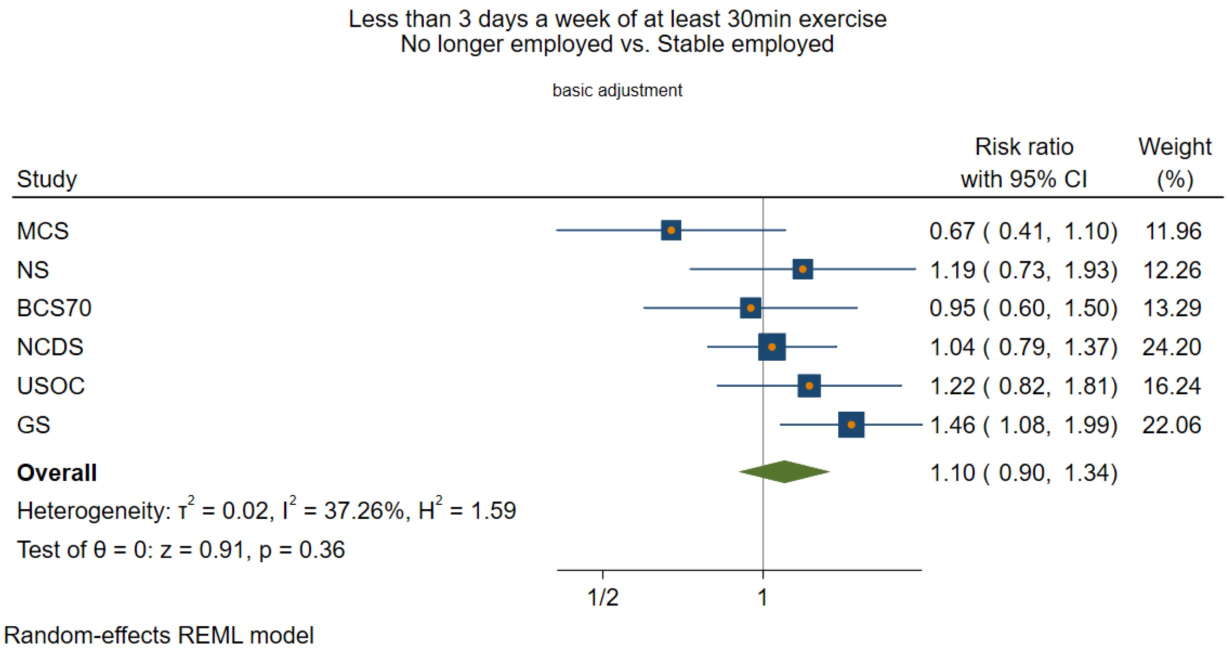
Less than 3 days a week of at least 30min exercise
No longer employed vs. Stable employed
basic adjustment
Study
Risk ratio with 95% CI
Weight (%)
MCS 0.67 (0.41, 1.10) 11.96
NS 1.19 (0.73, 1.93) 12.26
BCS70 0.95 (0.60, 1.50) 13.29
NCDS 1.04 (0.79, 1.37) 24.20
USOC 1.22 (0.82, 1.81) 16.24
GS 1.46 (1.08, 1.99) 22.06
Overall 1.10 (0.90, 1.34)
Heterogeneity: τ² = 0.02, I² = 37.26%, H² = 1.59
Test of θ = 0: z = 0.91, p = 0.36
1/2
1
Random-effects REML model

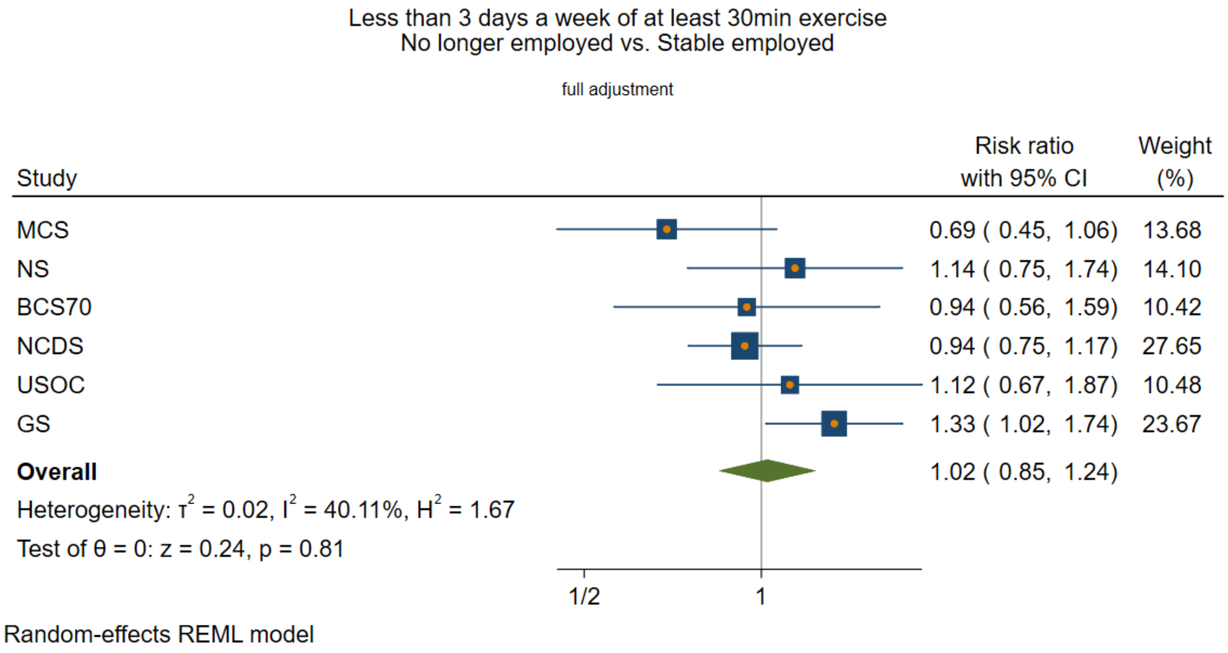
Less than 3 days a week of at least 30min exercise
No longer employed vs. Stable employed
full adjustment
Study
Risk ratio with 95% CI
Weight (%)
MCS 0.69 (0.45, 1.06) 13.68
NS 1.14 (0.75, 1.74) 14.10
BCS70 0.94 (0.56, 1.59) 10.42
NCDS 0.94 (0.75, 1.17) 27.65
USOC 1.12 (0.67, 1.87) 10.48
GS 1.33 (1.02, 1.74) 23.67
Overall 1.02 (0.85, 1.24)
Heterogeneity: τ² = 0.02, I² = 40.11%, H² = 1.67
Test of θ = 0: z = 0.24, p = 0.81
1/2
1
Random-effects REML model

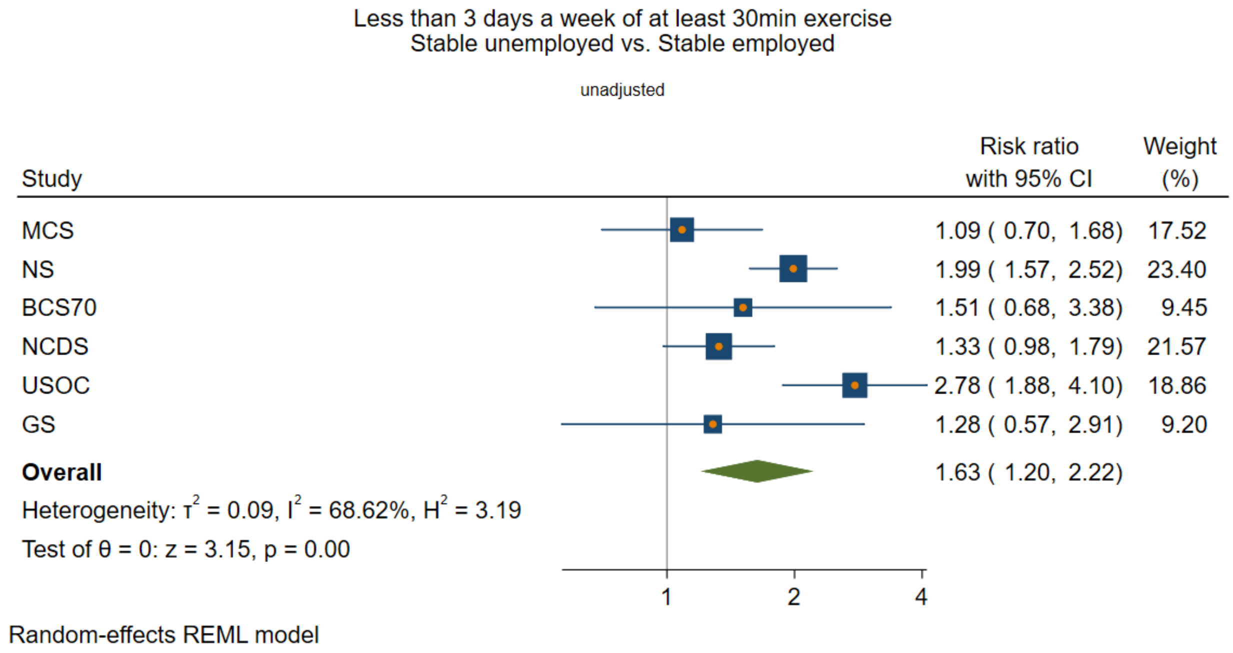
Less than 3 days a week of at least 30min exercise
Stable unemployed vs. Stable employed
unadjusted
Study
Risk ratio with 95% CI
Weight (%)
MCS 1.09 (0.70, 1.68) 17.52
NS 1.99 (1.57, 2.52) 23.40
BCS70 1.51 (0.68, 3.38) 9.45
NCDS 1.33 (0.98, 1.79) 21.57
USOC 2.78 (1.88, 4.10) 18.86
GS 1.28 (0.57, 2.91) 9.20
Overall 1.63 (1.20, 2.22)
Heterogeneity: τ² = 0.09, I² = 68.62%, H² = 3.19
Test of θ = 0: z = 3.15, p = 0.00
1
2
4
Random-effects REML model

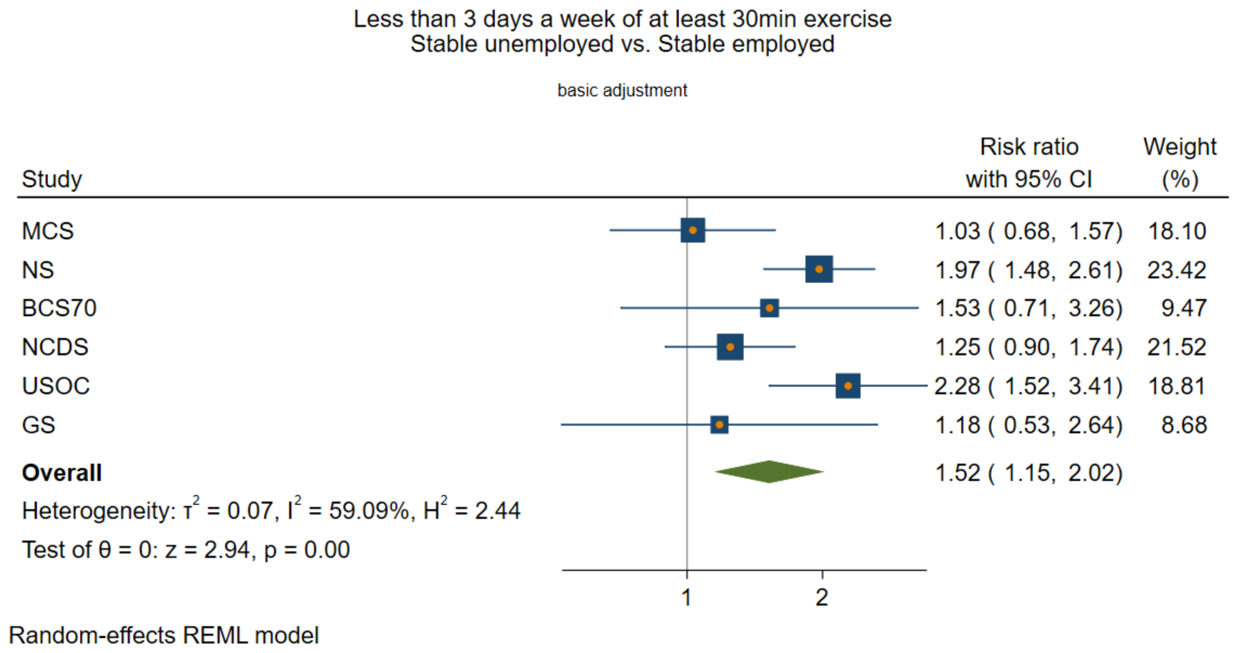
Less than 3 days a week of at least 30min exercise
Stable unemployed vs. Stable employed
basic adjustment
Study
Risk ratio with 95% CI
Weight (%)
MCS 1.03 (0.68, 1.57) 18.10
NS 1.97 (1.48, 2.61) 23.42
BCS70 1.53 (0.71, 3.26) 9.47
NCDS 1.25 (0.90, 1.74) 21.52
USOC 2.28 (1.52, 3.41) 18.81
GS 1.18 (0.53, 2.64) 8.68
Overall 1.52 (1.15, 2.02)
Heterogeneity: τ² = 0.07, I² = 59.09%, H² = 2.44
Test of θ = 0: z = 2.94, p = 0.00
1
2
Random-effects REML model

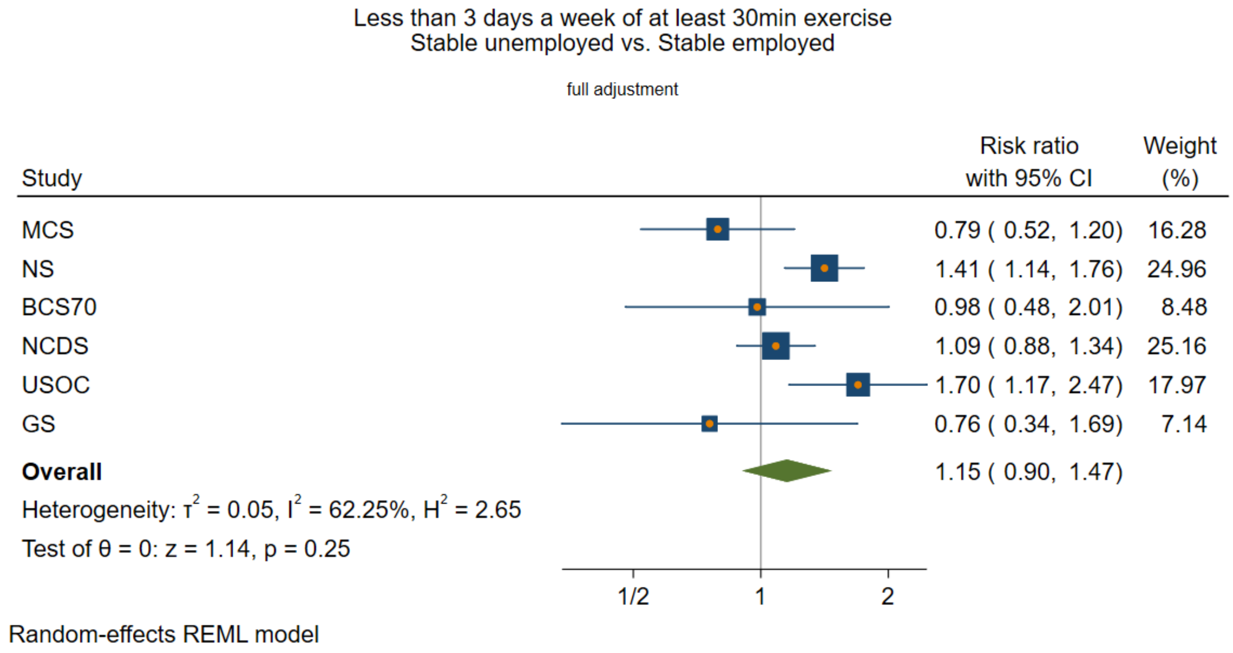
Less than 3 days a week of at least 30min exercise
Stable unemployed vs. Stable employed
full adjustment
Study
Risk ratio with 95% CI
Weight (%)
MCS 0.79 (0.52, 1.20) 16.28
NS 1.41 (1.14, 1.76) 24.96
BCS70 0.98 (0.48, 2.01) 8.48
NCDS 1.09 (0.88, 1.34) 25.16
USOC 1.70 (1.17, 2.47) 17.97
GS 0.76 (0.34, 1.69) 7.14
Overall 1.15 (0.90, 1.47)
Heterogeneity: τ² = 0.05, I² = 62.25%, H² = 2.65
Test of θ = 0: z = 1.14, p = 0.25
1/2
1
2
Random-effects REML model

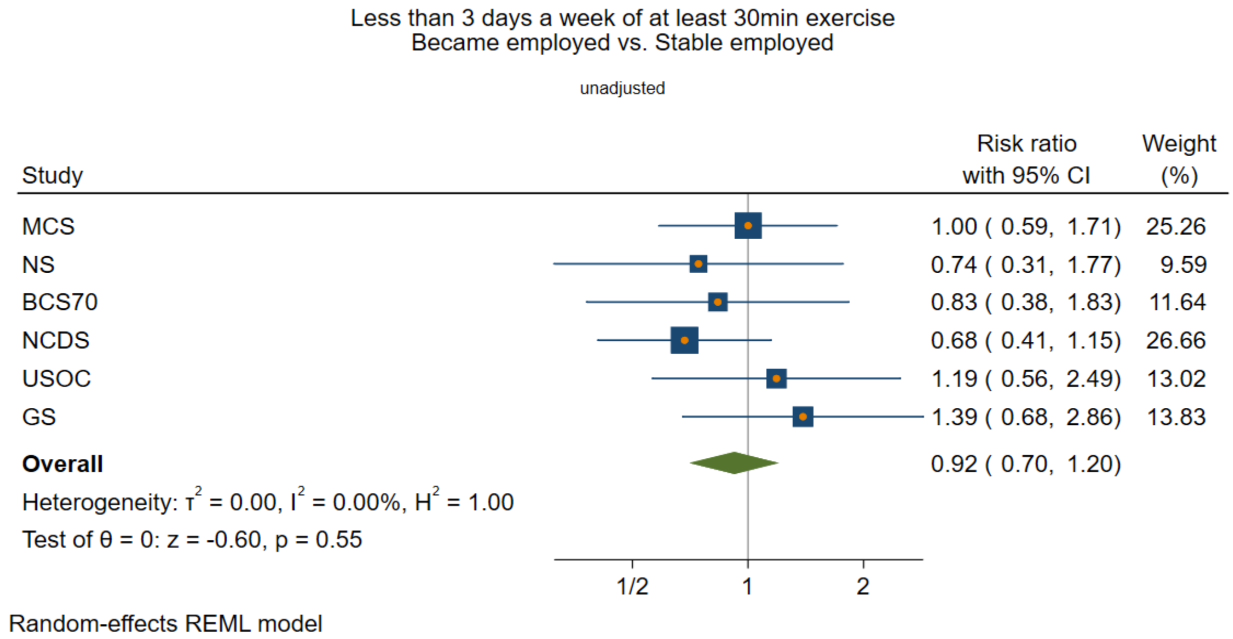

Less than 3 days a week of at least 30min exercise
Became employed vs. Stable employed
unadjusted
Study
Risk ratio with 95% CI
Weight (%)
MCS 1.00 (0.59, 1.71) 25.26
NS 0.74 (0.31, 1.77) 9.59
BCS70 0.83 (0.38, 1.83) 11.64
NCDS 0.68 (0.41, 1.15) 26.66
USOC 1.19 (0.56, 2.49) 13.02
GS 1.39 (0.68, 2.86) 13.83
Overall 0.92 (0.70, 1.20)
Heterogeneity: τ² = 0.00, I² = 0.00%, H² = 1.00
Test of θ = 0: z = -0.60, p = 0.55
1/2
1
2
Random-effects REML model

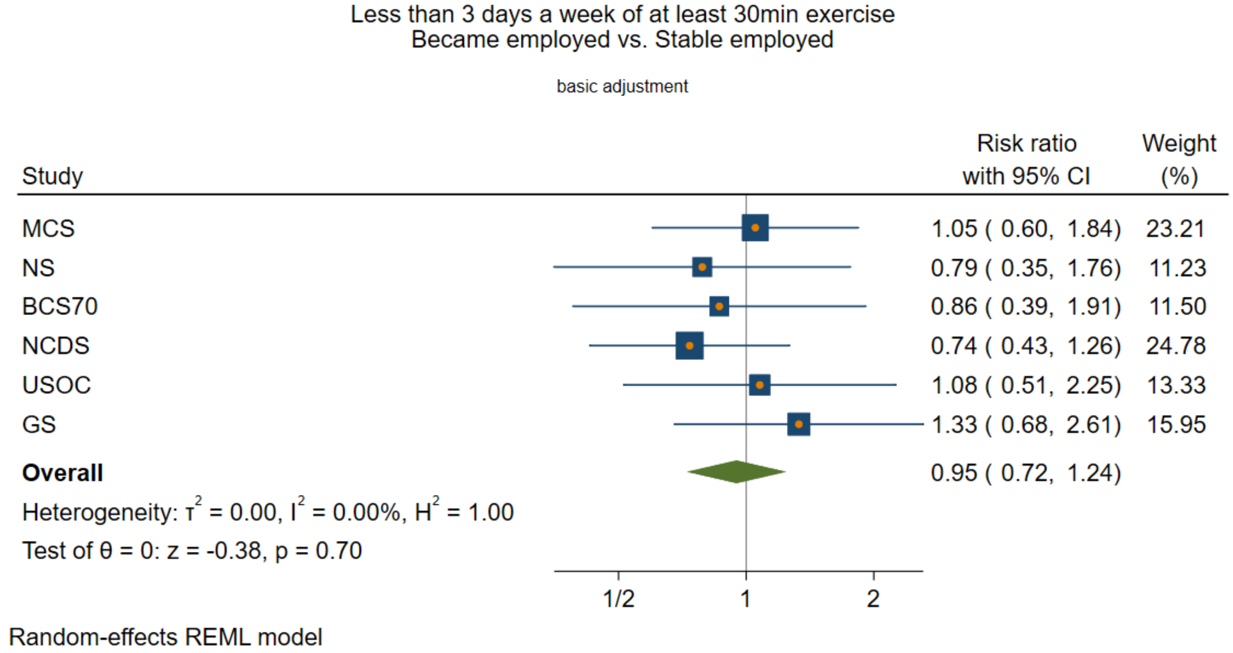

Less than 3 days a week of at least 30min exercise
Became employed vs. Stable employed
basic adjustment
Study
Risk ratio with 95% CI
Weight (%)
MCS 1.05 (0.60, 1.84) 23.21
NS 0.79 (0.35, 1.76) 11.23
BCS70 0.86 (0.39, 1.91) 11.50
NCDS 0.74 (0.43, 1.26) 24.78
USOC 1.08 (0.51, 2.25) 13.33
GS 1.33 (0.68, 2.61) 15.95
Overall 0.95 (0.72, 1.24)
Heterogeneity: τ² = 0.00, I² = 0.00%, H² = 1.00
Test of θ = 0: z = -0.38, p = 0.70
1/2
1
2
Random-effects REML model

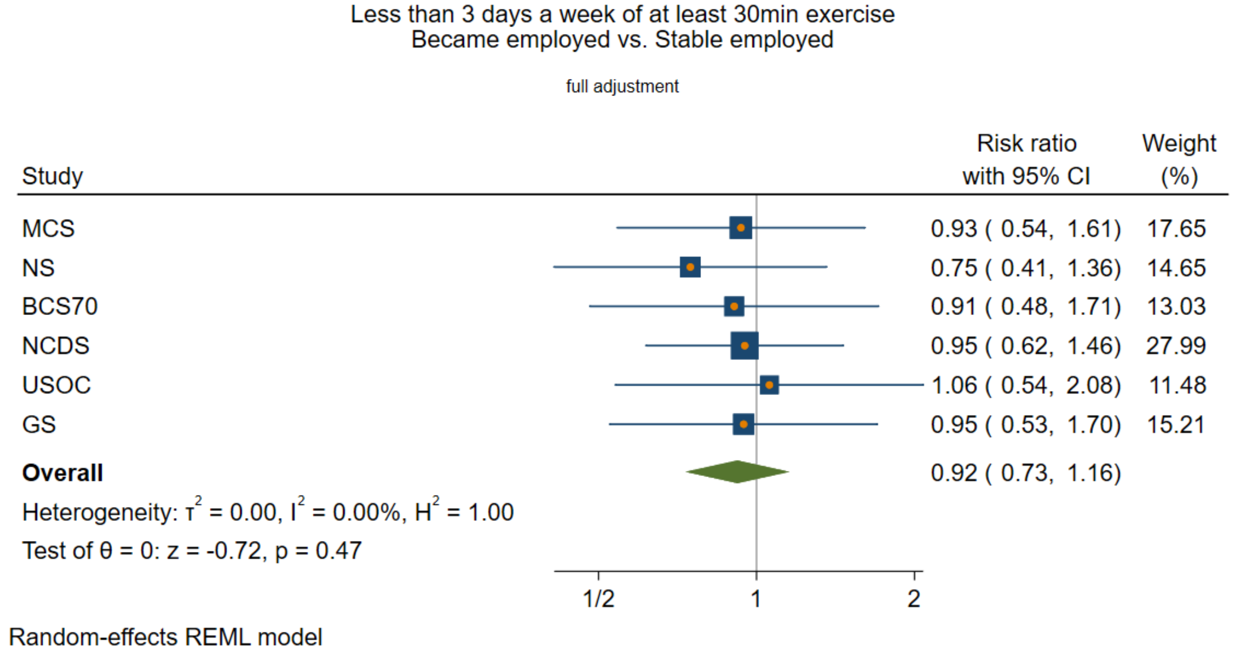

Less than 3 days a week of at least 30min exercise
Became employed vs. Stable employed
full adjustment
Study
Risk ratio with 95% CI
Weight (%)
MCS 0.93 (0.54, 1.61) 17.65
NS 0.75 (0.41, 1.36) 14.65
BCS70 0.91 (0.48, 1.71) 13.03
NCDS 0.95 (0.62, 1.46) 27.99
USOC 1.06 (0.54, 2.08) 11.48
GS 0.95 (0.53, 1.70) 15.21
Overall 0.92 (0.73, 1.16)
Heterogeneity: τ² = 0.00, I² = 0.00%, H² = 1.00
Test of θ = 0: z = -0.72, p = 0.47
1/2
1
2
Random-effects REML model

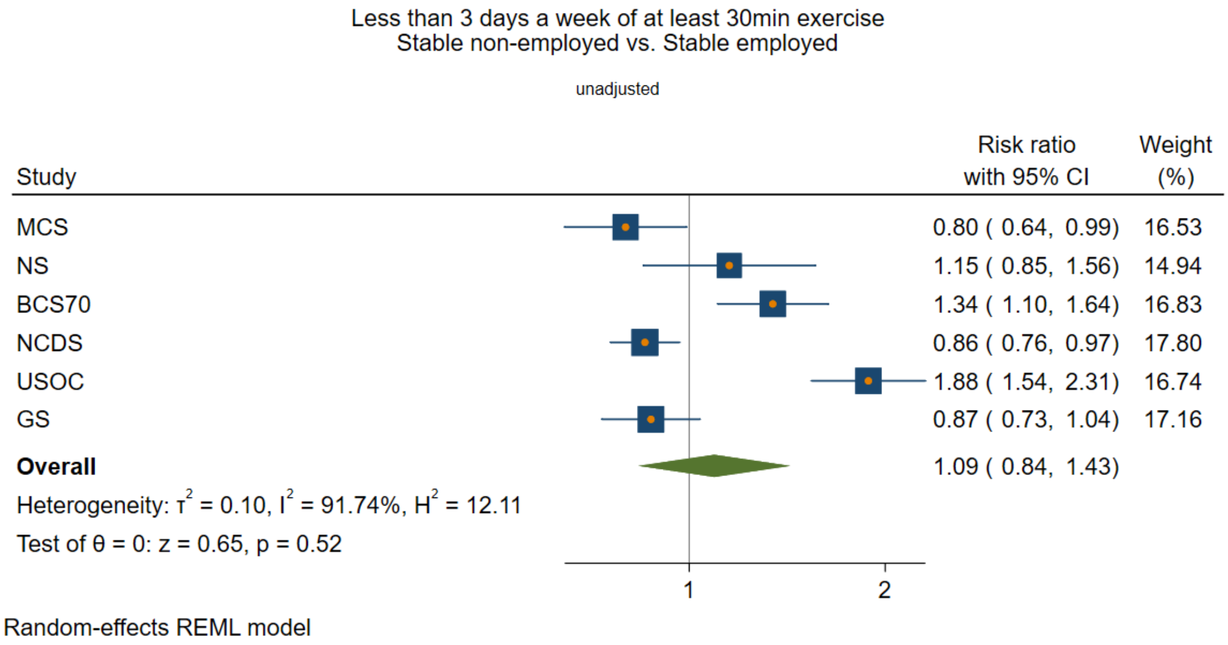
Less than 3 days a week of at least 30min exercise
Stable non-employed vs. Stable employed
unadjusted
Study
Risk ratio with 95% CI
Weight (%)
MCS 0.80 (0.64, 0.99) 16.53
NS 1.15 (0.85, 1.56) 14.94
BCS70 1.34 (1.10, 1.64) 16.83
NCDS 0.86 (0.76, 0.97) 17.80
USOC 1.88 (1.54, 2.31) 16.74
GS 0.87 (0.73, 1.04) 17.16
Overall 1.09 (0.84, 1.43)
Heterogeneity: τ² = 0.10, I² = 91.74%, H² = 12.11
Test of θ = 0: z = 0.65, p = 0.52
1
2
Random-effects REML model

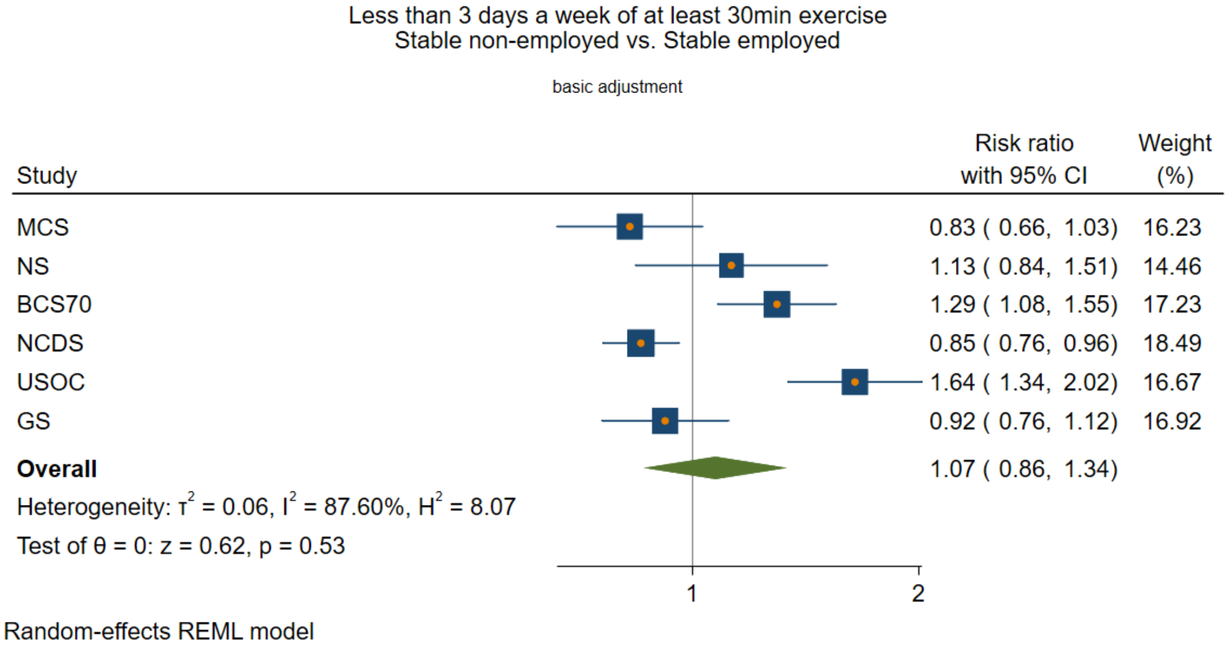
Less than 3 days a week of at least 30min exercise
Stable non-employed vs. Stable employed
basic adjustment
Study
Risk ratio with 95% CI
Weight (%)
MCS 0.83 (0.66, 1.03) 16.23
NS 1.13 (0.84, 1.51) 14.46
BCS70 1.29 (1.08, 1.55) 17.23
NCDS 0.85 (0.76, 0.96) 18.49
USOC 1.64 (1.34, 2.02) 16.67
GS 0.92 (0.76, 1.12) 16.92
Overall 1.07 (0.86, 1.34)
Heterogeneity: τ² = 0.06, I² = 87.60%, H² = 8.07
Test of θ = 0: z = 0.62, p = 0.53
1
2
Random-effects REML model

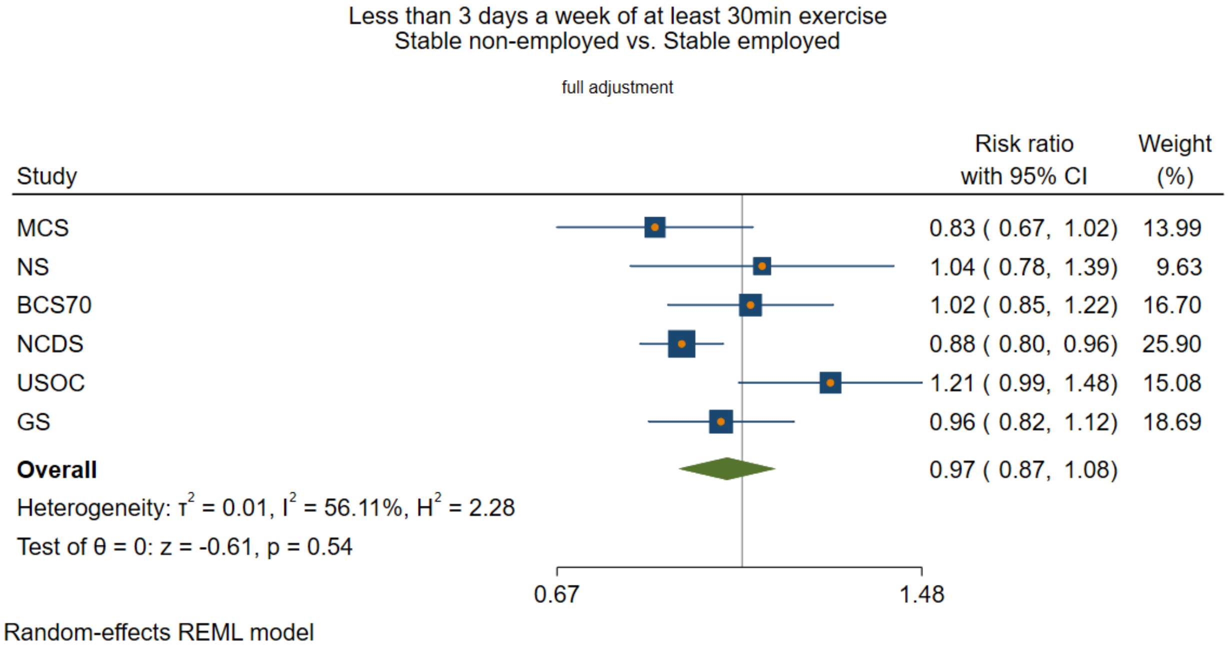
Less than 3 days a week of at least 30min exercise
Stable non-employed vs. Stable employed
full adjustment
Study
Risk ratio with 95% CI
Weight (%)
MCS 0.83 (0.67, 1.02) 13.99
NS 1.04 (0.78, 1.39) 9.63
BCS70 1.02 (0.85, 1.22) 16.70
NCDS 0.88 (0.80, 0.96) 25.90
USOC 1.21 (0.99, 1.48) 15.08
GS 0.96 (0.82, 1.12) 18.69
Overall 0.97 (0.87, 1.08)
Heterogeneity: τ² = 0.01, I² = 56.11%, H² = 2.28
Test of θ = 0: z = -0.61, p = 0.54
0.67
1.48
Random-effects REML model

Figure set 5: Less time/fewer days of physical exercise

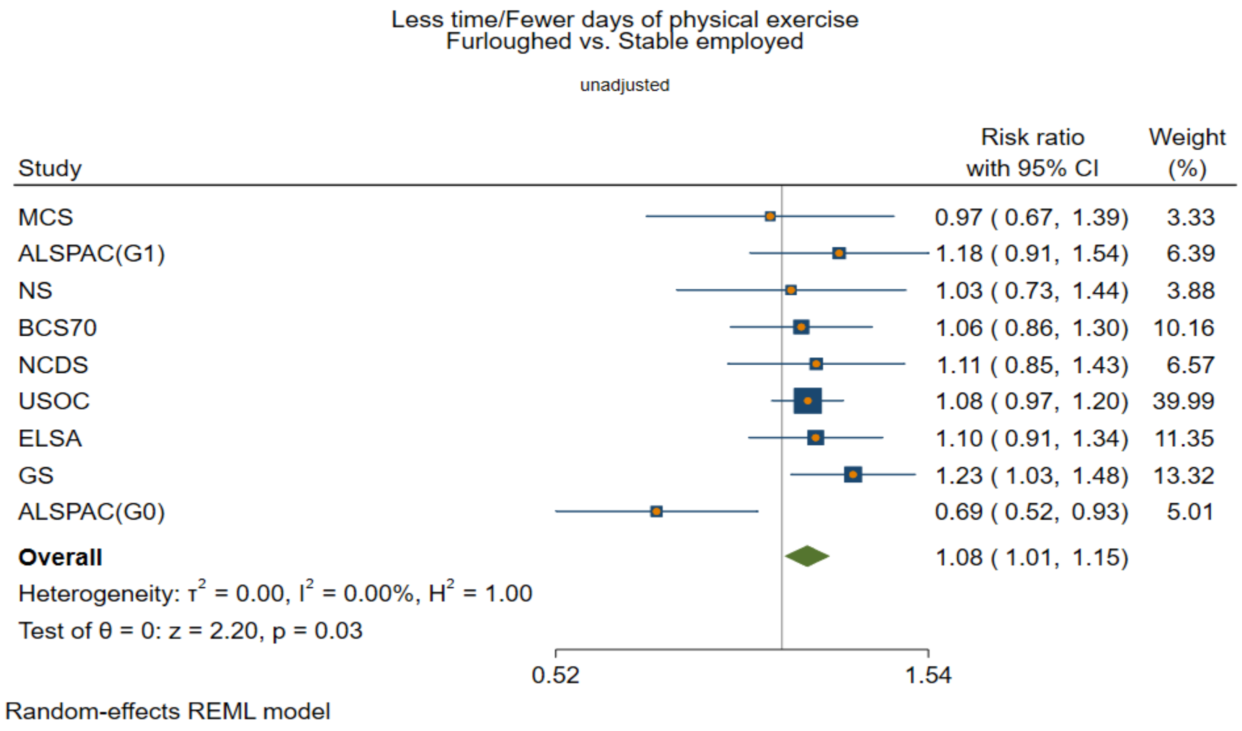

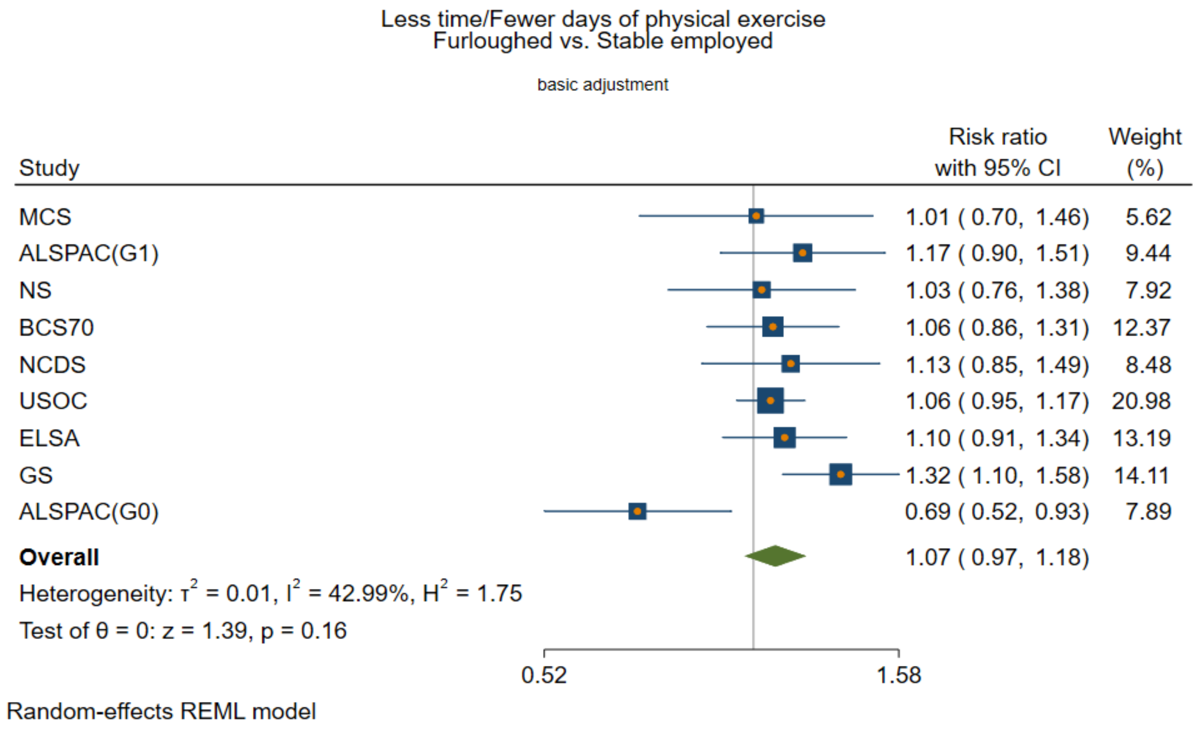

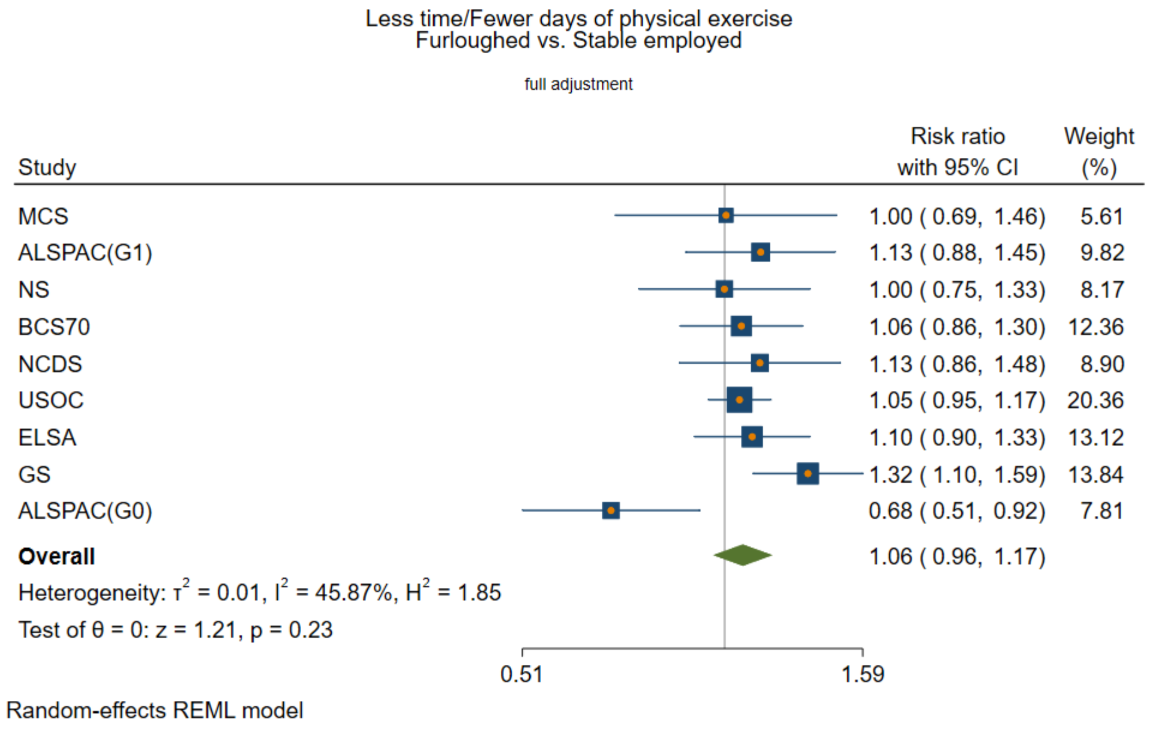

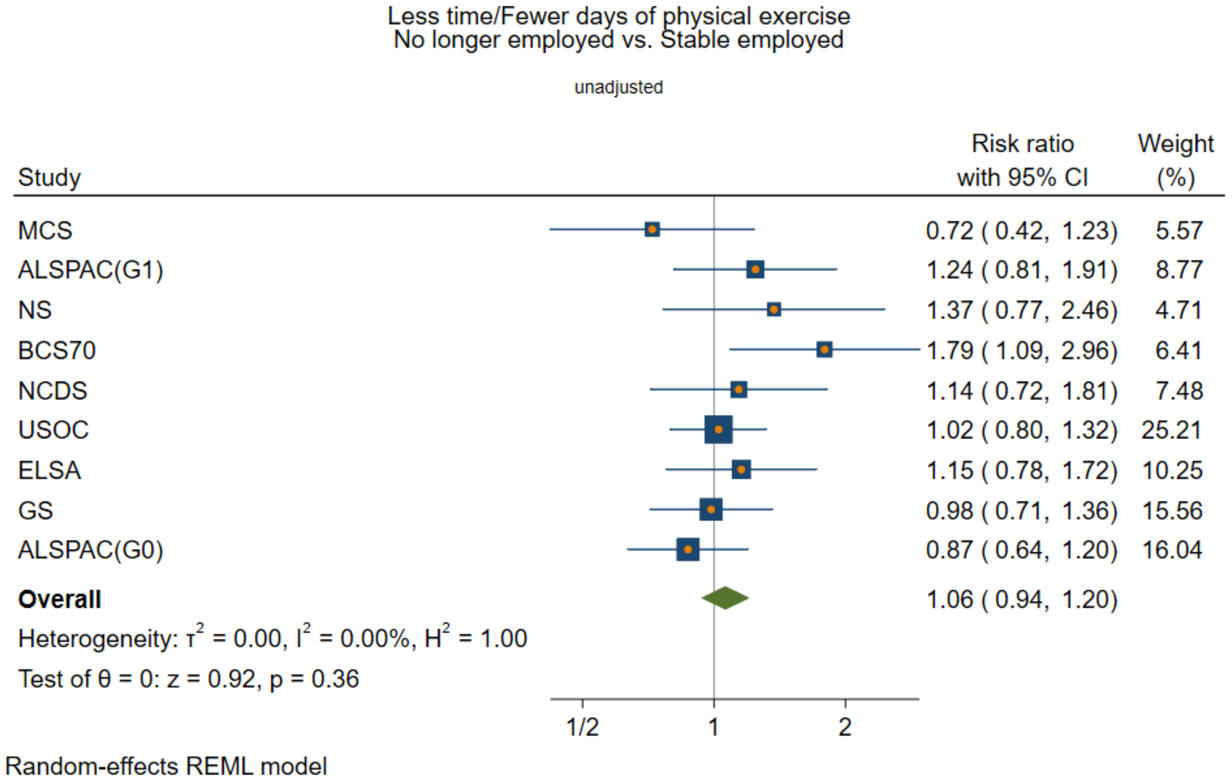
Less time/Fewer days of physical exercise
No longer employed vs. Stable employed
unadjusted
Study
Risk ratio with 95% CI
Weight (%)
MCS 0.72 (0.42, 1.23) 5.57
ALSPAC(G1) 1.24 (0.81, 1.91) 8.77
NS 1.37 (0.77, 2.46) 4.71
BCS70 1.79 (1.09, 2.96) 6.41
NCDS 1.14 (0.72, 1.81) 7.48
USOC 1.02 (0.80, 1.32) 25.21
ELSA 1.15 (0.78, 1.72) 10.25
GS 0.98 (0.71, 1.36) 15.56
ALSPAC(G0) 0.87 (0.64, 1.20) 16.04
Overall 1.06 (0.94, 1.20)
Heterogeneity: τ² = 0.00, I² = 0.00%, H² = 1.00
Test of θ = 0: z = 0.92, p = 0.36
1/2
1
2
Random-effects REML model

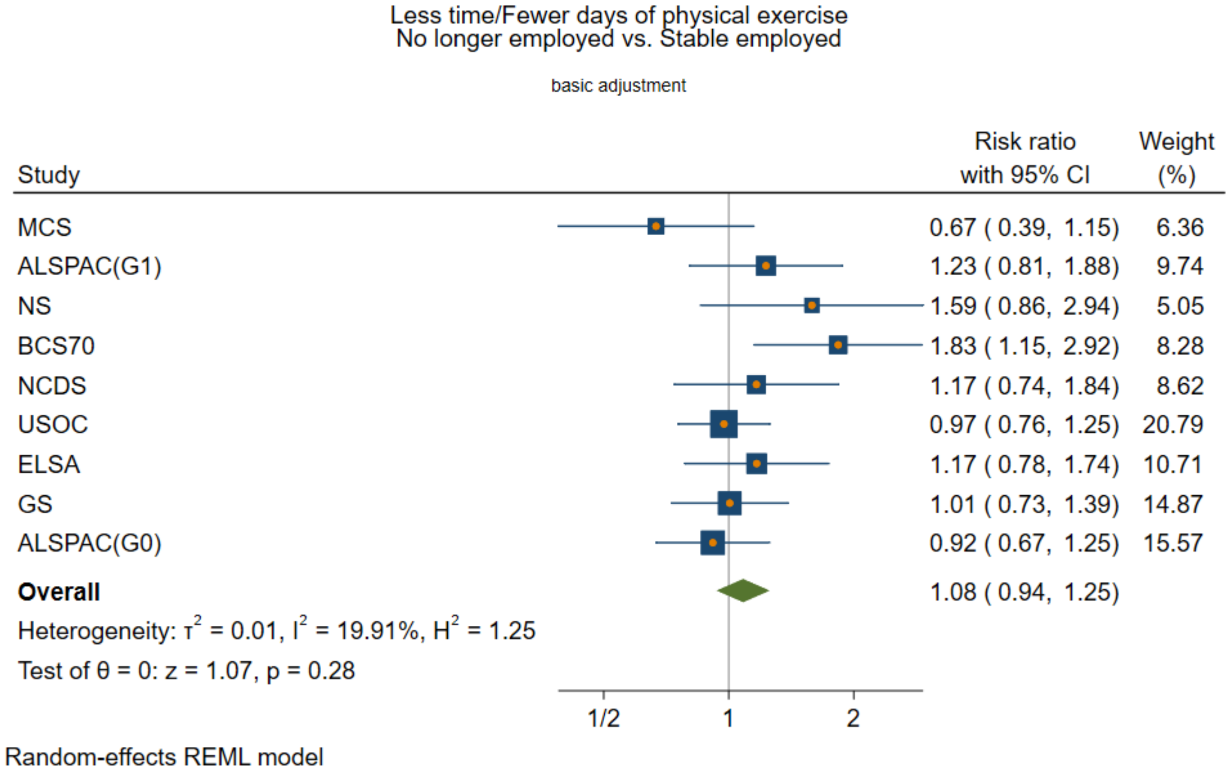
Less time/Fewer days of physical exercise
No longer employed vs. Stable employed
basic adjustment
Study
Risk ratio with 95% CI
Weight (%)
MCS 0.67 (0.39, 1.15) 6.36
ALSPAC(G1) 1.23 (0.81, 1.88) 9.74
NS 1.59 (0.86, 2.94) 5.05
BCS70 1.83 (1.15, 2.92) 8.28
NCDS 1.17 (0.74, 1.84) 8.62
USOC 0.97 (0.76, 1.25) 20.79
ELSA 1.17 (0.78, 1.74) 10.71
GS 1.01 (0.73, 1.39) 14.87
ALSPAC(G0) 0.92 (0.67, 1.25) 15.57
Overall 1.08 (0.94, 1.25)
Heterogeneity: τ² = 0.01, I² = 19.91%, H² = 1.25
Test of θ = 0: z = 1.07, p = 0.28
1/2
1
2
Random-effects REML model

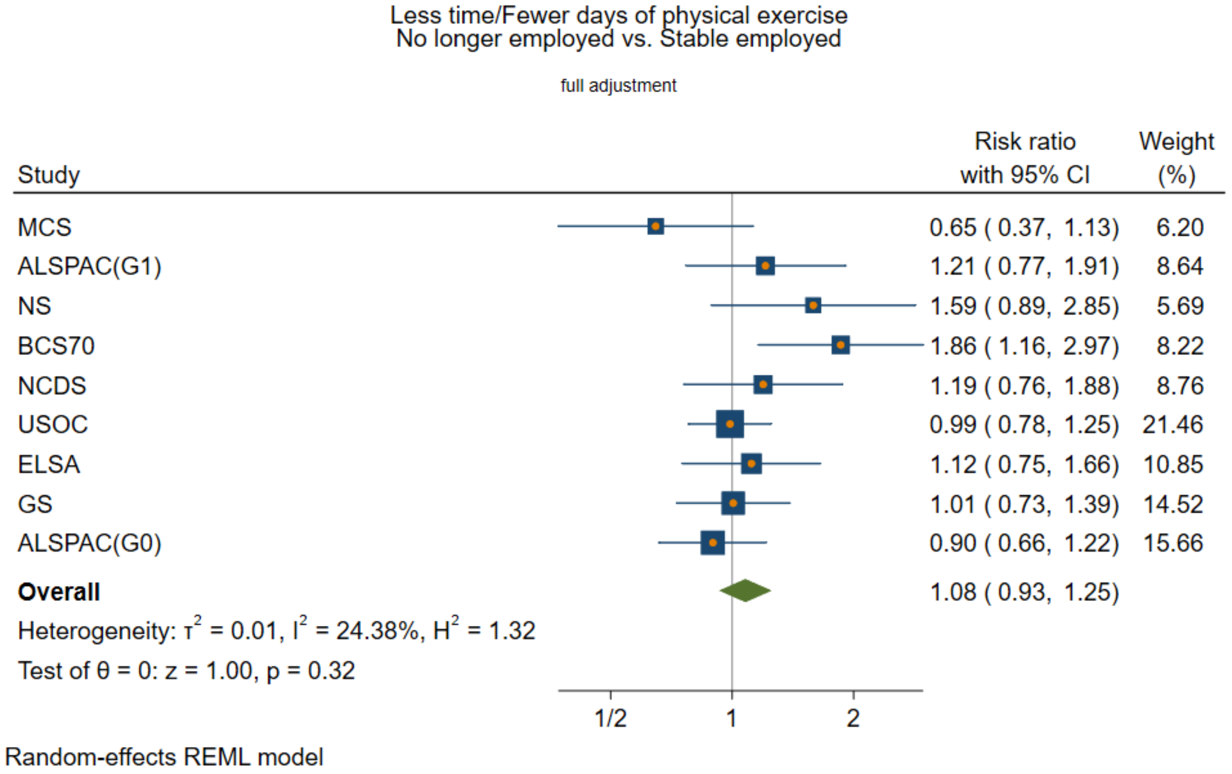
Less time/Fewer days of physical exercise
No longer employed vs. Stable employed
full adjustment
Study
Risk ratio with 95% CI
Weight (%)
MCS 0.65 (0.37, 1.13) 6.20
ALSPAC(G1) 1.21 (0.77, 1.91) 8.64
NS 1.59 (0.89, 2.85) 5.69
BCS70 1.86 (1.16, 2.97) 8.22
NCDS 1.19 (0.76, 1.88) 8.76
USOC 0.99 (0.78, 1.25) 21.46
ELSA 1.12 (0.75, 1.66) 10.85
GS 1.01 (0.73, 1.39) 14.52
ALSPAC(G0) 0.90 (0.66, 1.22) 15.66
Overall 1.08 (0.93, 1.25)
Heterogeneity: τ² = 0.01, I² = 24.38%, H² = 1.32
Test of θ = 0: z = 1.00, p = 0.32
1/2
1
2
Random-effects REML model

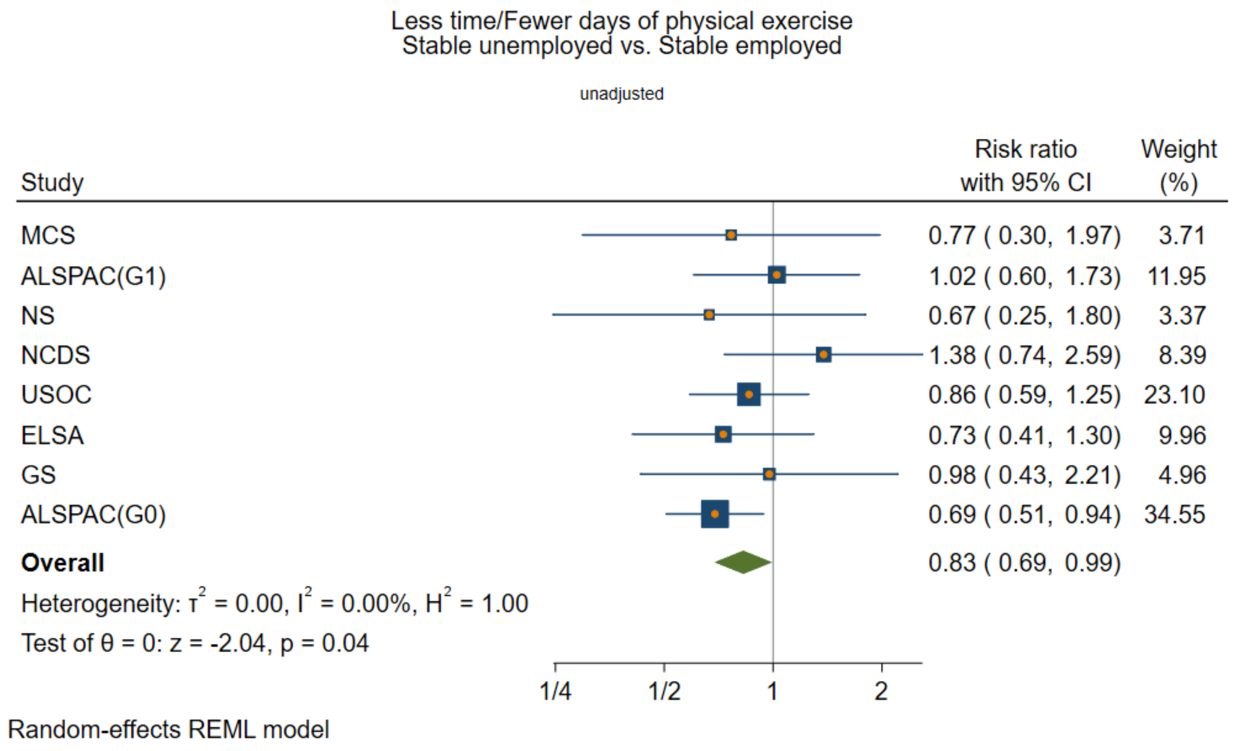
Less time/Fewer days of physical exercise
Stable unemployed vs. Stable employed
unadjusted
Study
Risk ratio with 95% CI
Weight (%)
MCS 0.77 (0.30, 1.97) 3.71
ALSPAC(G1) 1.02 (0.60, 1.73) 11.95
NS 0.67 (0.25, 1.80) 3.37
NCDS 1.38 (0.74, 2.59) 8.39
USOC 0.86 (0.59, 1.25) 23.10
ELSA 0.73 (0.41, 1.30) 9.96
GS 0.98 (0.43, 2.21) 4.96
ALSPAC(G0) 0.69 (0.51, 0.94) 34.55
Overall 0.83 (0.69, 0.99)
Heterogeneity: τ² = 0.00, I² = 0.00%, H² = 1.00
Test of θ = 0: z = -2.04, p = 0.04
1/4
1/2
1
2
Random-effects REML model

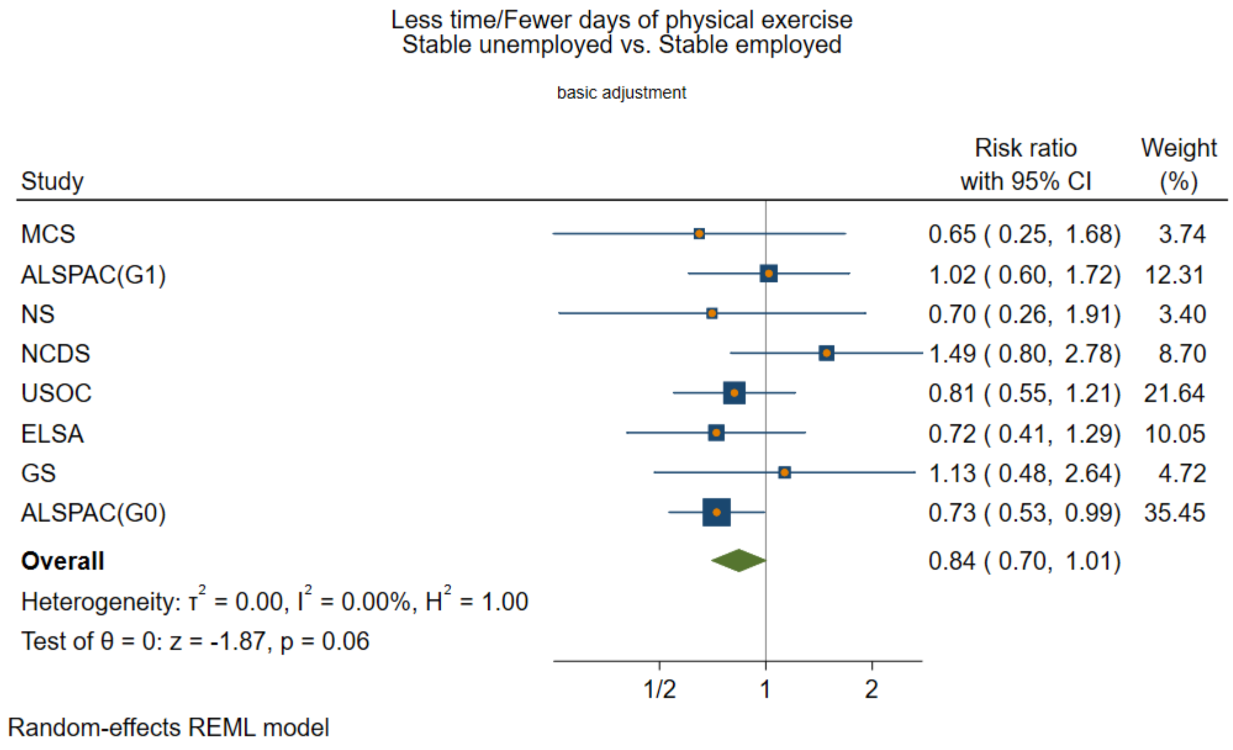
Less time/Fewer days of physical exercise
Stable unemployed vs. Stable employed
basic adjustment
Study
Risk ratio with 95% CI
Weight (%)
MCS 0.65 (0.25, 1.68) 3.74
ALSPAC(G1) 1.02 (0.60, 1.72) 12.31
NS 0.70 (0.26, 1.91) 3.40
NCDS 1.49 (0.80, 2.78) 8.70
USOC 0.81 (0.55, 1.21) 21.64
ELSA 0.72 (0.41, 1.29) 10.05
GS 1.13 (0.48, 2.64) 4.72
ALSPAC(G0) 0.73 (0.53, 0.99) 35.45
Overall 0.84 (0.70, 1.01)
Heterogeneity: τ² = 0.00, I² = 0.00%, H² = 1.00
Test of θ = 0: z = -1.87, p = 0.06
1/2
1
2
Random-effects REML model

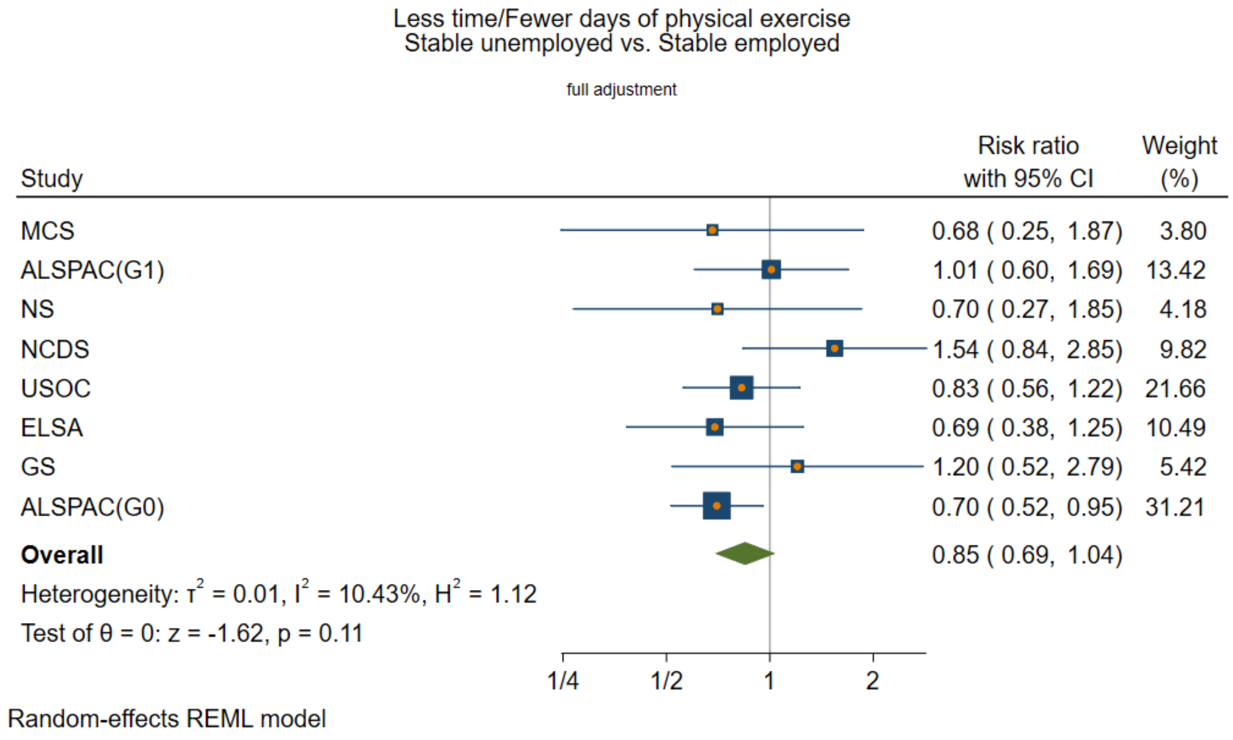
Less time/Fewer days of physical exercise
Stable unemployed vs. Stable employed
full adjustment
Study
Risk ratio with 95% CI
Weight (%)
MCS 0.68 (0.25, 1.87) 3.80
ALSPAC(G1) 1.01 (0.60, 1.69) 13.42
NS 0.70 (0.27, 1.85) 4.18
NCDS 1.54 (0.84, 2.85) 9.82
USOC 0.83 (0.56, 1.22) 21.66
ELSA 0.69 (0.38, 1.25) 10.49
GS 1.20 (0.52, 2.79) 5.42
ALSPAC(G0) 0.70 (0.52, 0.95) 31.21
Overall 0.85 (0.69, 1.04)
Heterogeneity: τ² = 0.01, I² = 10.43%, H² = 1.12
Test of θ = 0: z = -1.62, p = 0.11
1/4
1/2
1
2
Random-effects REML model

Less time/Fewer days of physical exercise
Became employed vs. Stable employed

unadjusted

Study	Risk ratio with 95% CI	Weight (%)
MCS	0.43 (0.18, 1.02)	9.46
ALSPAC(G1)	0.92 (0.47, 1.81)	12.21
NS	0.27 (0.07, 1.06)	4.92
BCS70	0.73 (0.24, 2.25)	6.53
NCDS	1.22 (0.65, 2.31)	12.95
USOC	0.93 (0.63, 1.39)	17.93
ELSA	0.98 (0.40, 2.40)	8.89
GS	1.27 (0.68, 2.37)	13.19
ALSPAC(G0)	0.37 (0.21, 0.66)	13.91
**Overall**	0.76 (0.54, 1.07)	

Heterogeneity: $\tau^2 = 0.12$, $I^2 = 50.27\%$, $H^2 = 2.01$

Test of $\theta = 0$: $z = -1.56$, $p = 0.12$

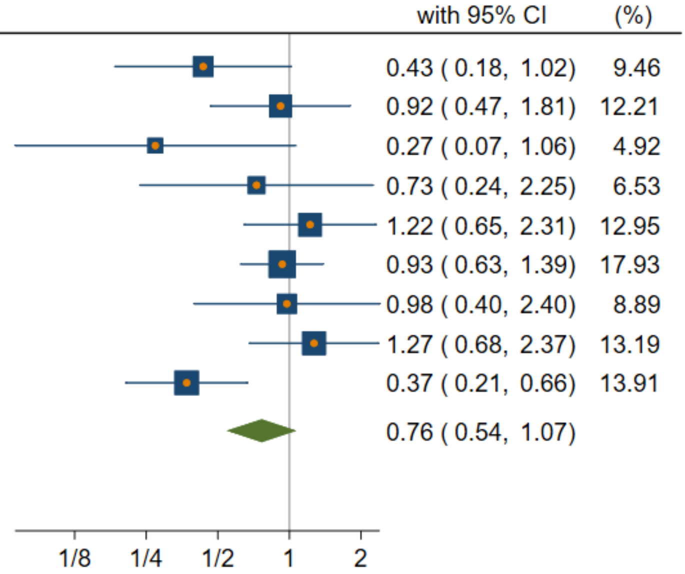

Random-effects REML model

Less time/Fewer days of physical exercise
Became employed vs. Stable employed

basic adjustment

Study	Risk ratio with 95% CI	Weight (%)
MCS	0.42 (0.17, 1.01)	8.89
ALSPAC(G1)	0.94 (0.47, 1.89)	11.97
NS	0.31 (0.08, 1.21)	4.65
BCS70	0.74 (0.24, 2.31)	6.27
NCDS	1.23 (0.66, 2.30)	13.33
USOC	0.88 (0.60, 1.30)	18.84
ELSA	0.94 (0.37, 2.40)	8.36
GS	1.26 (0.68, 2.32)	13.56
ALSPAC(G0)	0.38 (0.21, 0.69)	14.12
**Overall**	0.77 (0.55, 1.07)	

Heterogeneity: $\tau^2 = 0.11$, $I^2 = 47.47\%$, $H^2 = 1.90$

Test of $\theta = 0$: $z = -1.57$, $p = 0.12$

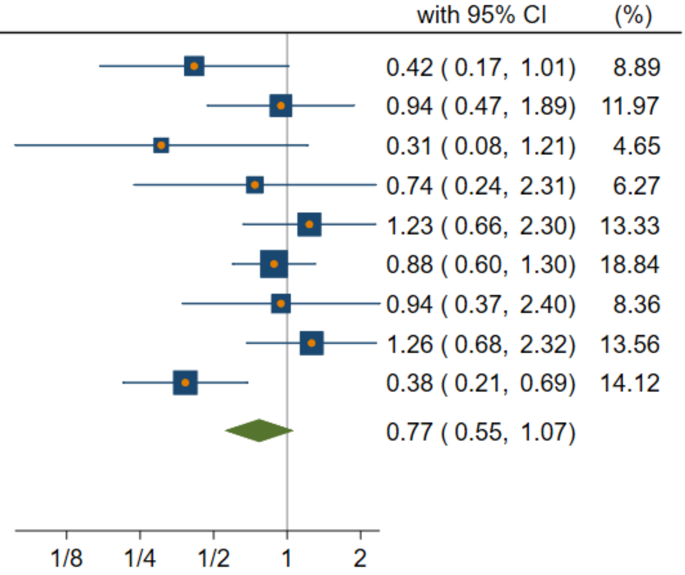

Random-effects REML model

Less time/Fewer days of physical exercise
Became employed vs. Stable employed

full adjustment

Study	Risk ratio with 95% CI	Weight (%)
MCS	0.42 (0.17, 1.05)	9.77
ALSPAC(G1)	0.97 (0.49, 1.89)	13.53
NS	0.29 (0.07, 1.15)	5.40
BCS70	0.76 (0.23, 2.49)	6.76
NCDS	1.23 (0.67, 2.29)	14.59
USOC	0.89 (0.60, 1.32)	19.66
GS	1.24 (0.68, 2.27)	14.88
ALSPAC(G0)	0.39 (0.22, 0.69)	15.41
**Overall**	0.76 (0.53, 1.09)	

Heterogeneity: $\tau^2 = 0.13$, $I^2 = 53.60\%$, $H^2 = 2.16$

Test of $\theta = 0$: $z = -1.51$, $p = 0.13$

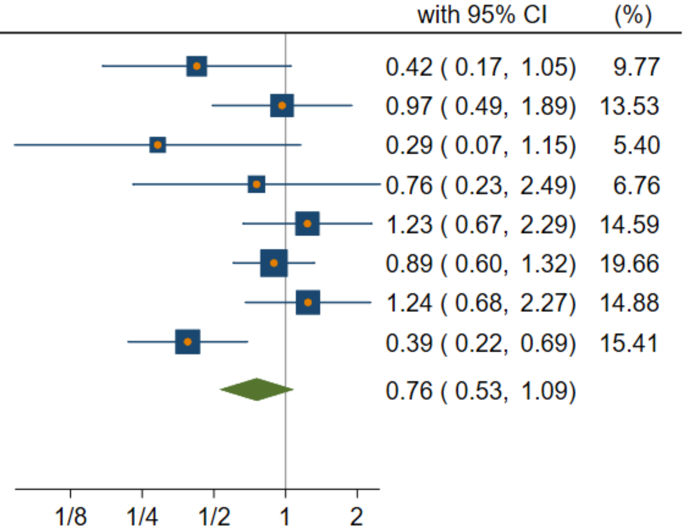

Random-effects REML model

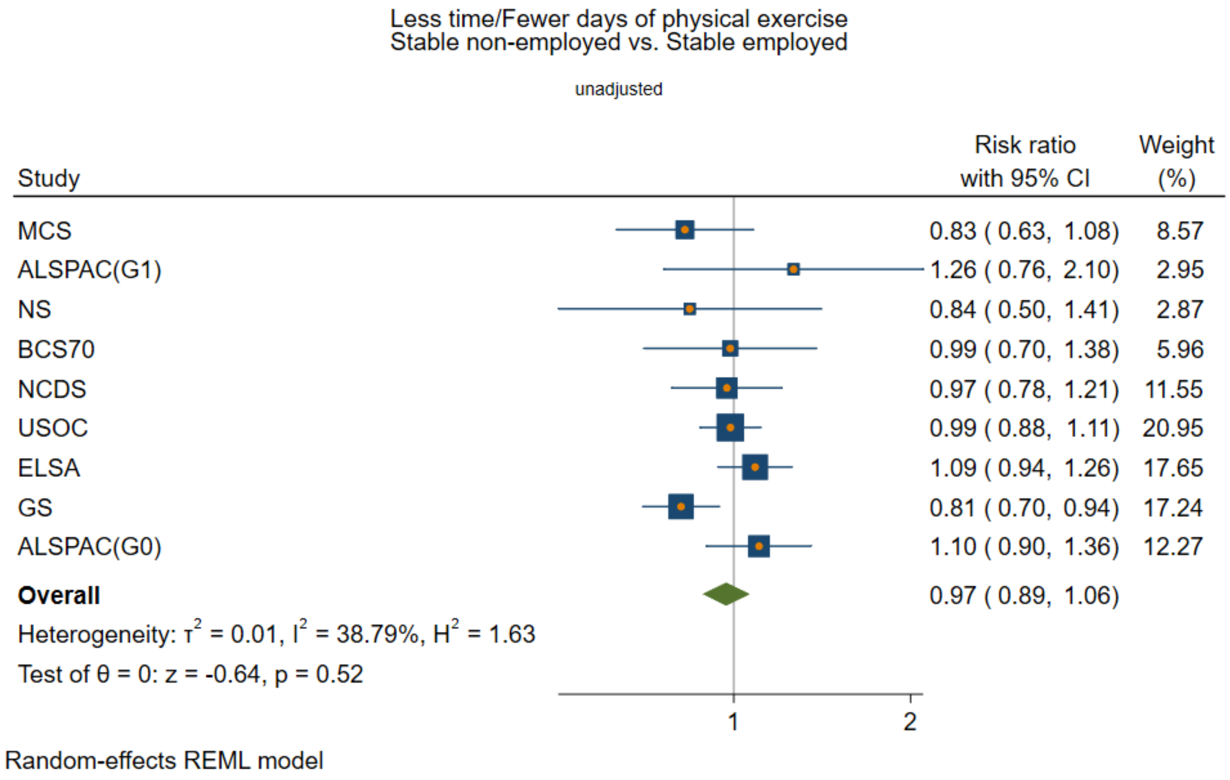
Less time/Fewer days of physical exercise
Stable non-employed vs. Stable employed
unadjusted
Study
Risk ratio with 95% CI
Weight (%)
MCS 0.83 (0.63, 1.08) 8.57
ALSPAC(G1) 1.26 (0.76, 2.10) 2.95
NS 0.84 (0.50, 1.41) 2.87
BCS70 0.99 (0.70, 1.38) 5.96
NCDS 0.97 (0.78, 1.21) 11.55
USOC 0.99 (0.88, 1.11) 20.95
ELSA 1.09 (0.94, 1.26) 17.65
GS 0.81 (0.70, 0.94) 17.24
ALSPAC(G0) 1.10 (0.90, 1.36) 12.27
Overall 0.97 (0.89, 1.06)
Heterogeneity: τ^2 = 0.01, I^2 = 38.79%, H^2 = 1.63
Test of θ = 0: z = -0.64, p = 0.52
1
2
Random-effects REML model

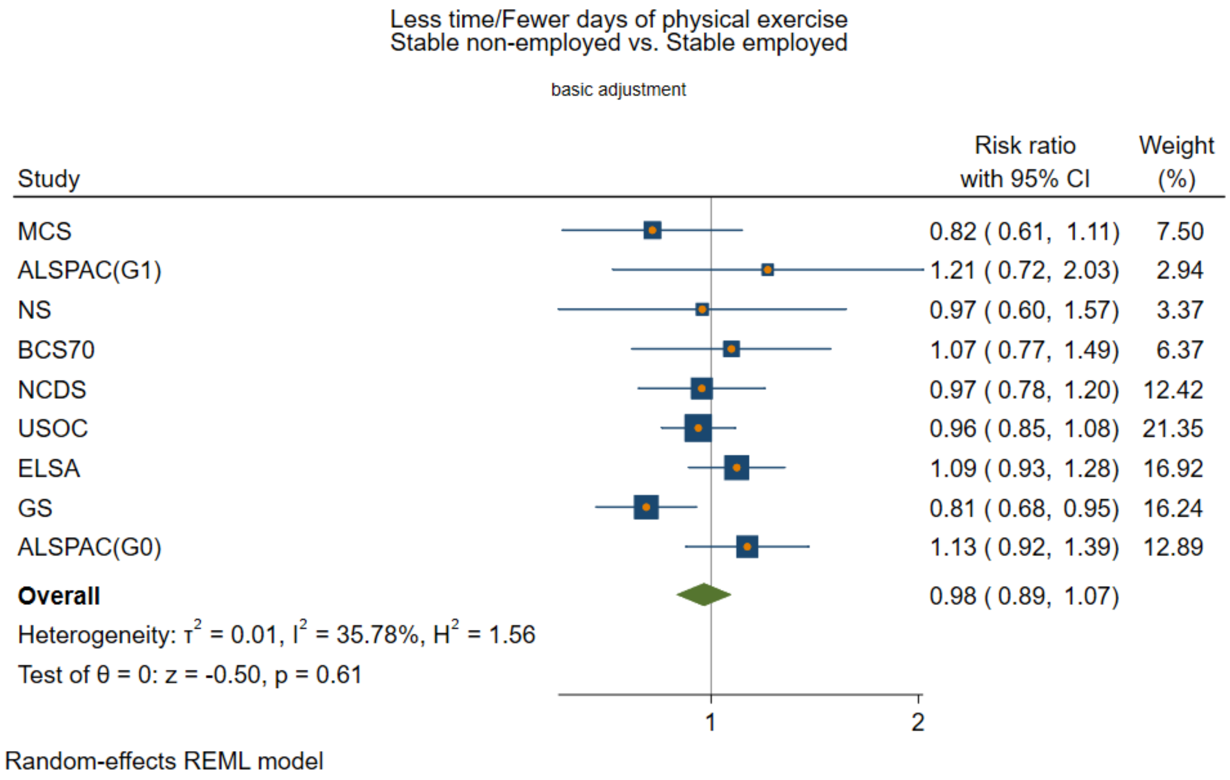
Less time/Fewer days of physical exercise
Stable non-employed vs. Stable employed
basic adjustment
Study
Risk ratio with 95% CI
Weight (%)
MCS 0.82 (0.61, 1.11) 7.50
ALSPAC(G1) 1.21 (0.72, 2.03) 2.94
NS 0.97 (0.60, 1.57) 3.37
BCS70 1.07 (0.77, 1.49) 6.37
NCDS 0.97 (0.78, 1.20) 12.42
USOC 0.96 (0.85, 1.08) 21.35
ELSA 1.09 (0.93, 1.28) 16.92
GS 0.81 (0.68, 0.95) 16.24
ALSPAC(G0) 1.13 (0.92, 1.39) 12.89
Overall 0.98 (0.89, 1.07)
Heterogeneity: τ^2 = 0.01, I^2 = 35.78%, H^2 = 1.56
Test of θ = 0: z = -0.50, p = 0.61
1
2
Random-effects REML model

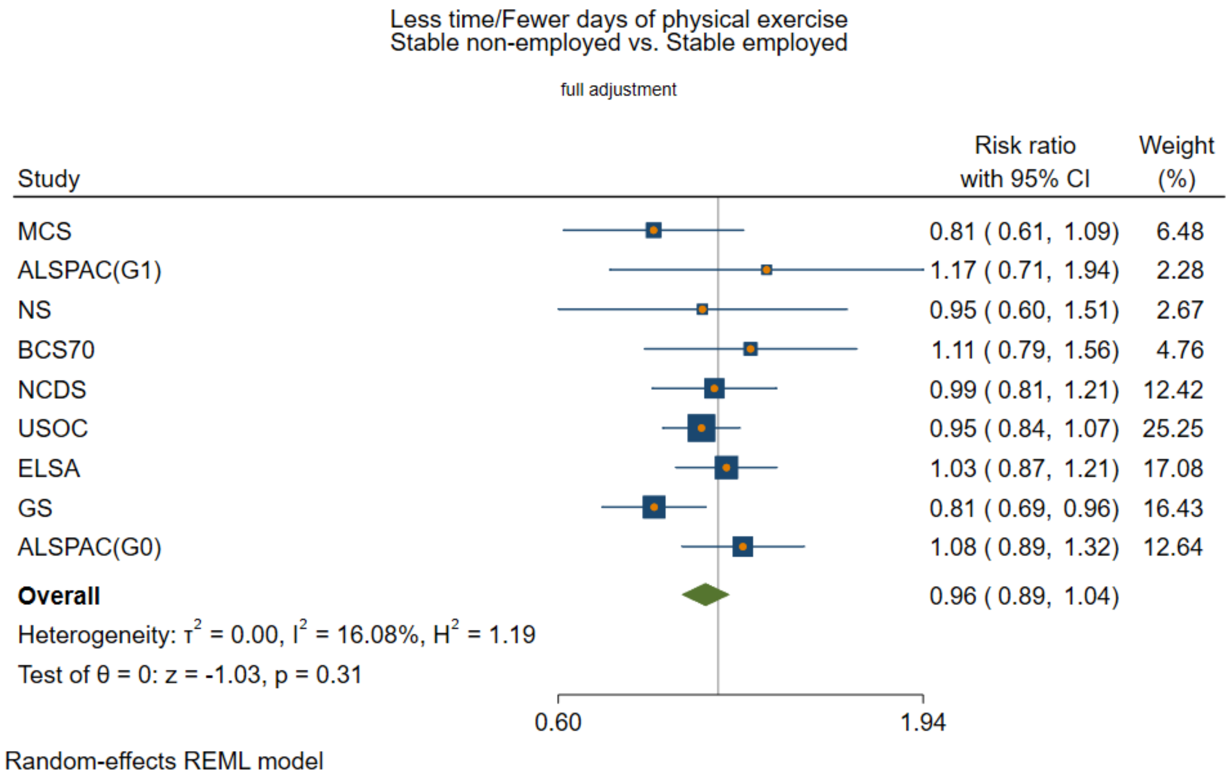
Less time/Fewer days of physical exercise
Stable non-employed vs. Stable employed
full adjustment
Study
Risk ratio with 95% CI
Weight (%)
MCS 0.81 (0.61, 1.09) 6.48
ALSPAC(G1) 1.17 (0.71, 1.94) 2.28
NS 0.95 (0.60, 1.51) 2.67
BCS70 1.11 (0.79, 1.56) 4.76
NCDS 0.99 (0.81, 1.21) 12.42
USOC 0.95 (0.84, 1.07) 25.25
ELSA 1.03 (0.87, 1.21) 17.08
GS 0.81 (0.69, 0.96) 16.43
ALSPAC(G0) 1.08 (0.89, 1.32) 12.64
Overall 0.96 (0.89, 1.04)
Heterogeneity: τ^2 = 0.00, I^2 = 16.08%, H^2 = 1.19
Test of θ = 0: z = -1.03, p = 0.31
0.60
1.94
Random-effects REML model

Figure set 6: More time/days of physical exercise

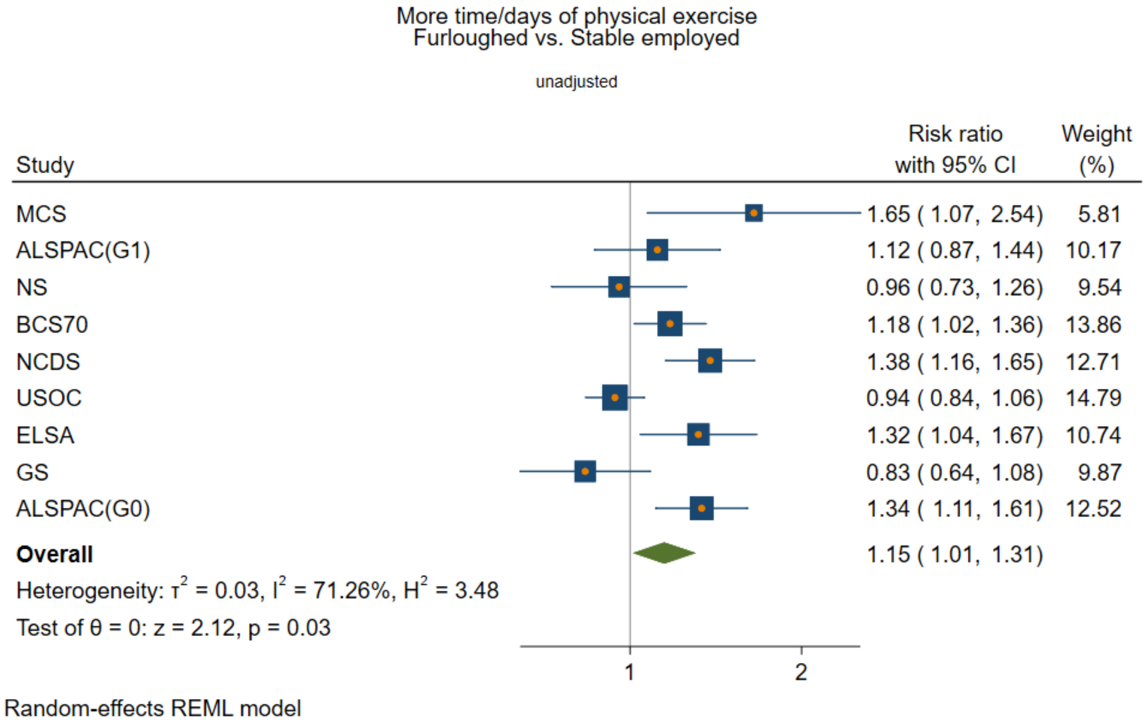

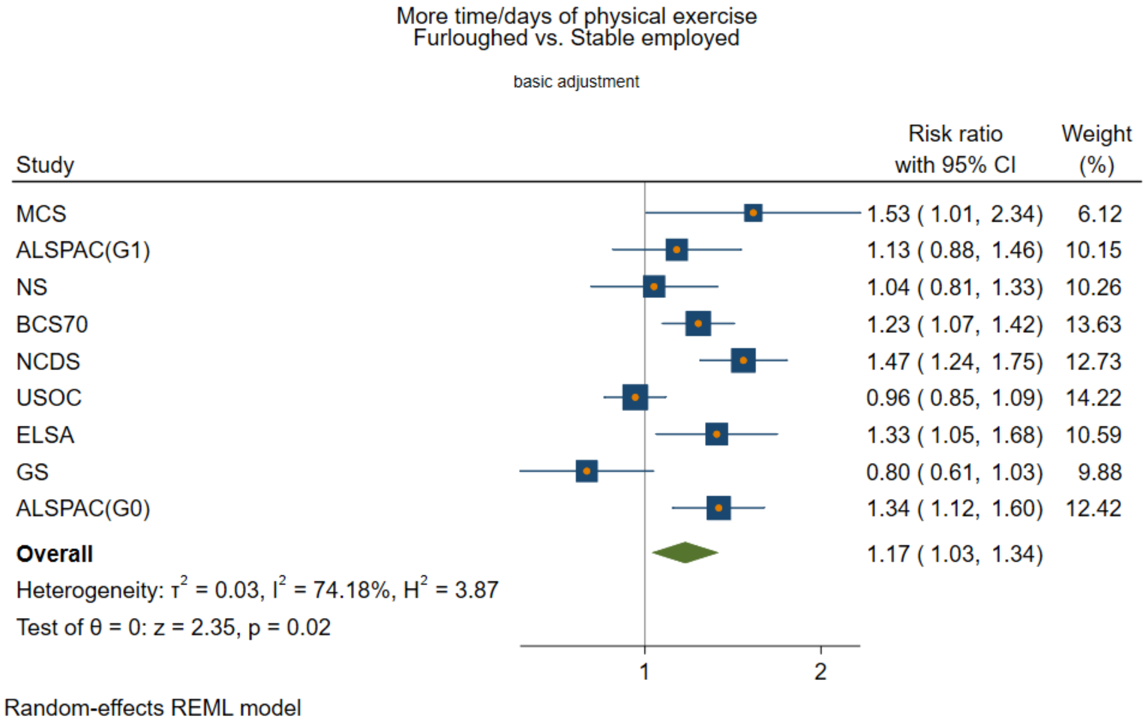

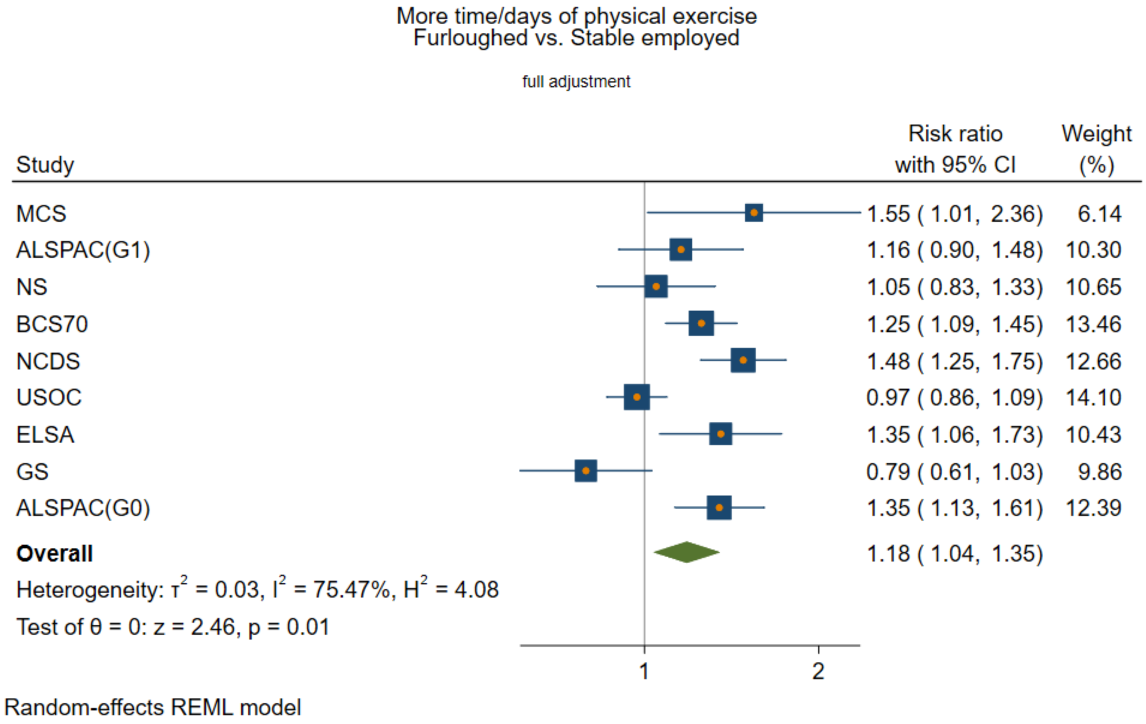

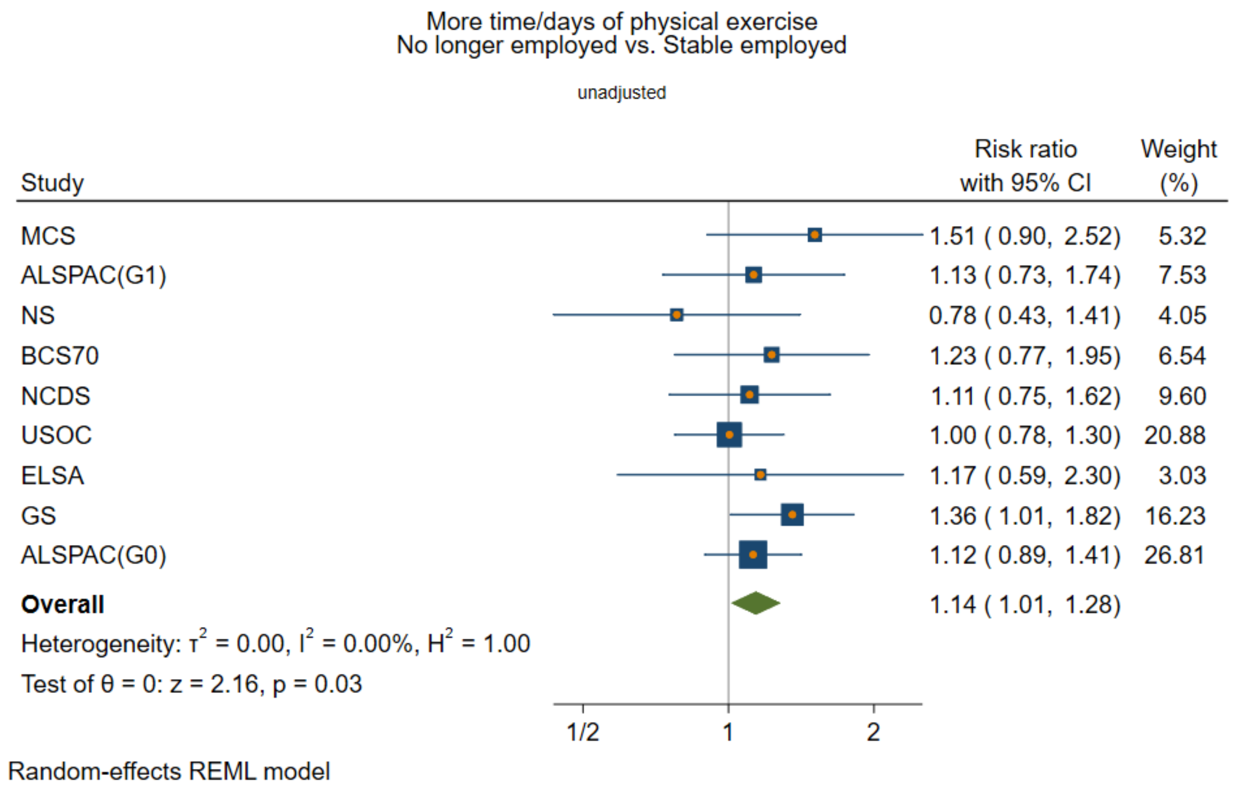
More time/days of physical exercise
No longer employed vs. Stable employed
unadjusted
Study
Risk ratio with 95% CI
Weight (%)
MCS 1.51 (0.90, 2.52) 5.32
ALSPAC(G1) 1.13 (0.73, 1.74) 7.53
NS 0.78 (0.43, 1.41) 4.05
BCS70 1.23 (0.77, 1.95) 6.54
NCDS 1.11 (0.75, 1.62) 9.60
USOC 1.00 (0.78, 1.30) 20.88
ELSA 1.17 (0.59, 2.30) 3.03
GS 1.36 (1.01, 1.82) 16.23
ALSPAC(G0) 1.12 (0.89, 1.41) 26.81
Overall 1.14 (1.01, 1.28)
Heterogeneity: τ^2 = 0.00, I^2 = 0.00%, H^2 = 1.00
Test of θ = 0: z = 2.16, p = 0.03
1/2
1
2
Random-effects REML model

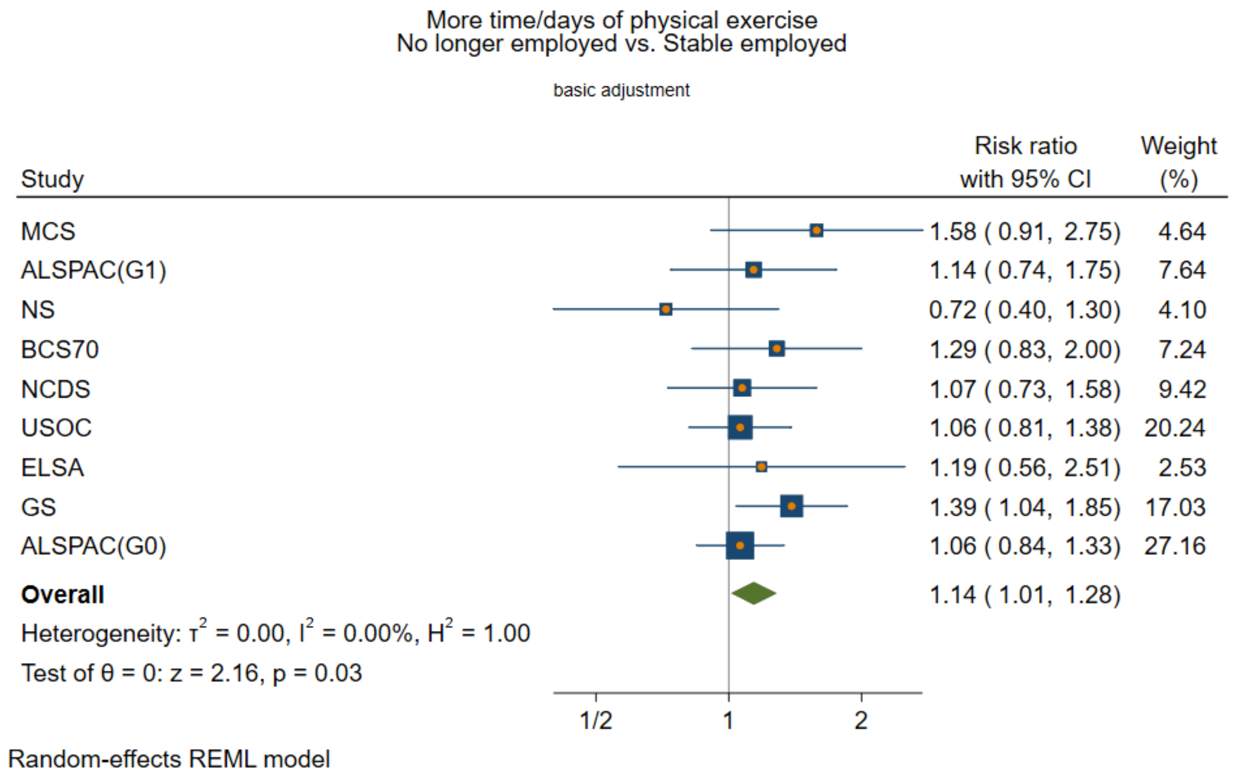
More time/days of physical exercise
No longer employed vs. Stable employed
basic adjustment
Study
Risk ratio with 95% CI
Weight (%)
MCS 1.58 (0.91, 2.75) 4.64
ALSPAC(G1) 1.14 (0.74, 1.75) 7.64
NS 0.72 (0.40, 1.30) 4.10
BCS70 1.29 (0.83, 2.00) 7.24
NCDS 1.07 (0.73, 1.58) 9.42
USOC 1.06 (0.81, 1.38) 20.24
ELSA 1.19 (0.56, 2.51) 2.53
GS 1.39 (1.04, 1.85) 17.03
ALSPAC(G0) 1.06 (0.84, 1.33) 27.16
Overall 1.14 (1.01, 1.28)
Heterogeneity: τ^2 = 0.00, I^2 = 0.00%, H^2 = 1.00
Test of θ = 0: z = 2.16, p = 0.03
1/2
1
2
Random-effects REML model

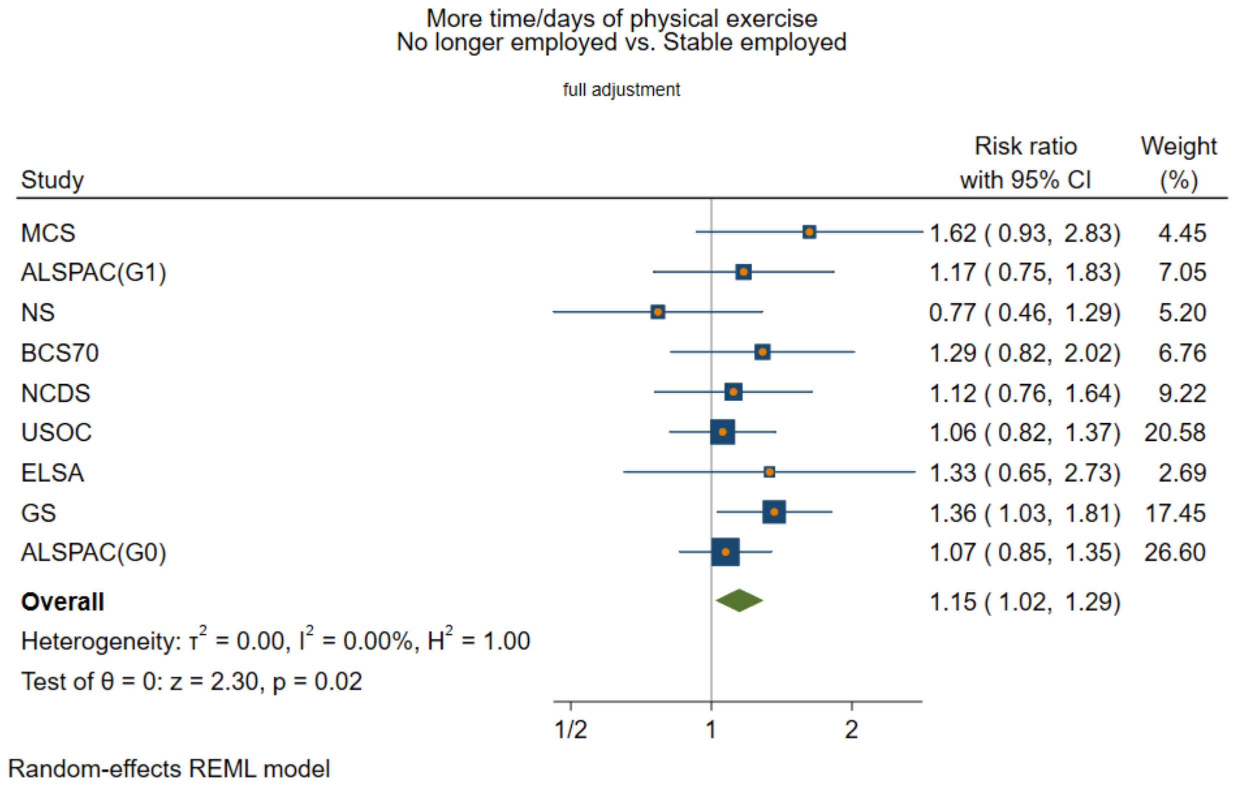
More time/days of physical exercise
No longer employed vs. Stable employed
full adjustment
Study
Risk ratio with 95% CI
Weight (%)
MCS 1.62 (0.93, 2.83) 4.45
ALSPAC(G1) 1.17 (0.75, 1.83) 7.05
NS 0.77 (0.46, 1.29) 5.20
BCS70 1.29 (0.82, 2.02) 6.76
NCDS 1.12 (0.76, 1.64) 9.22
USOC 1.06 (0.82, 1.37) 20.58
ELSA 1.33 (0.65, 2.73) 2.69
GS 1.36 (1.03, 1.81) 17.45
ALSPAC(G0) 1.07 (0.85, 1.35) 26.60
Overall 1.15 (1.02, 1.29)
Heterogeneity: τ^2 = 0.00, I^2 = 0.00%, H^2 = 1.00
Test of θ = 0: z = 2.30, p = 0.02
1/2
1
2
Random-effects REML model

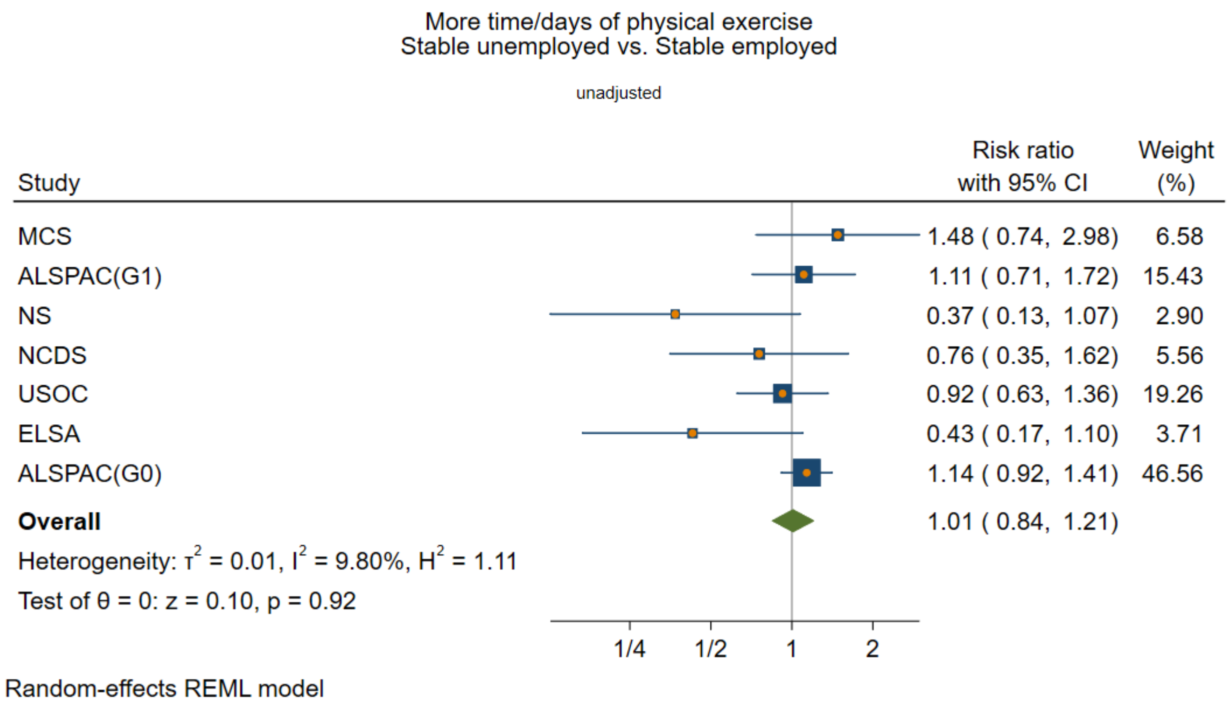
More time/days of physical exercise
Stable unemployed vs. Stable employed
unadjusted
Study
Risk ratio with 95% CI
Weight (%)
MCS 1.48 (0.74, 2.98) 6.58
ALSPAC(G1) 1.11 (0.71, 1.72) 15.43
NS 0.37 (0.13, 1.07) 2.90
NCDS 0.76 (0.35, 1.62) 5.56
USOC 0.92 (0.63, 1.36) 19.26
ELSA 0.43 (0.17, 1.10) 3.71
ALSPAC(G0) 1.14 (0.92, 1.41) 46.56
Overall 1.01 (0.84, 1.21)
Heterogeneity: τ² = 0.01, I² = 9.80%, H² = 1.11
Test of θ = 0: z = 0.10, p = 0.92
1/4
1/2
1
2
Random-effects REML model

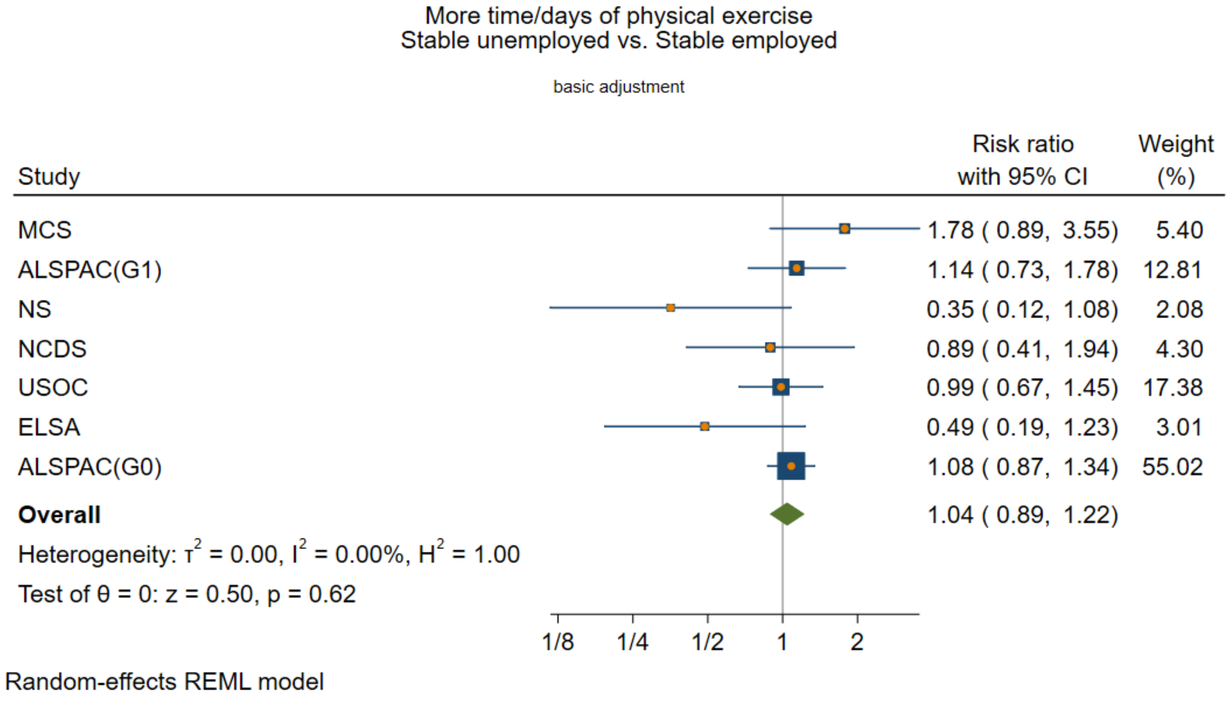
More time/days of physical exercise
Stable unemployed vs. Stable employed
basic adjustment
Study
Risk ratio with 95% CI
Weight (%)
MCS 1.78 (0.89, 3.55) 5.40
ALSPAC(G1) 1.14 (0.73, 1.78) 12.81
NS 0.35 (0.12, 1.08) 2.08
NCDS 0.89 (0.41, 1.94) 4.30
USOC 0.99 (0.67, 1.45) 17.38
ELSA 0.49 (0.19, 1.23) 3.01
ALSPAC(G0) 1.08 (0.87, 1.34) 55.02
Overall 1.04 (0.89, 1.22)
Heterogeneity: τ² = 0.00, I² = 0.00%, H² = 1.00
Test of θ = 0: z = 0.50, p = 0.62
1/8
1/4
1/2
1
2
Random-effects REML model

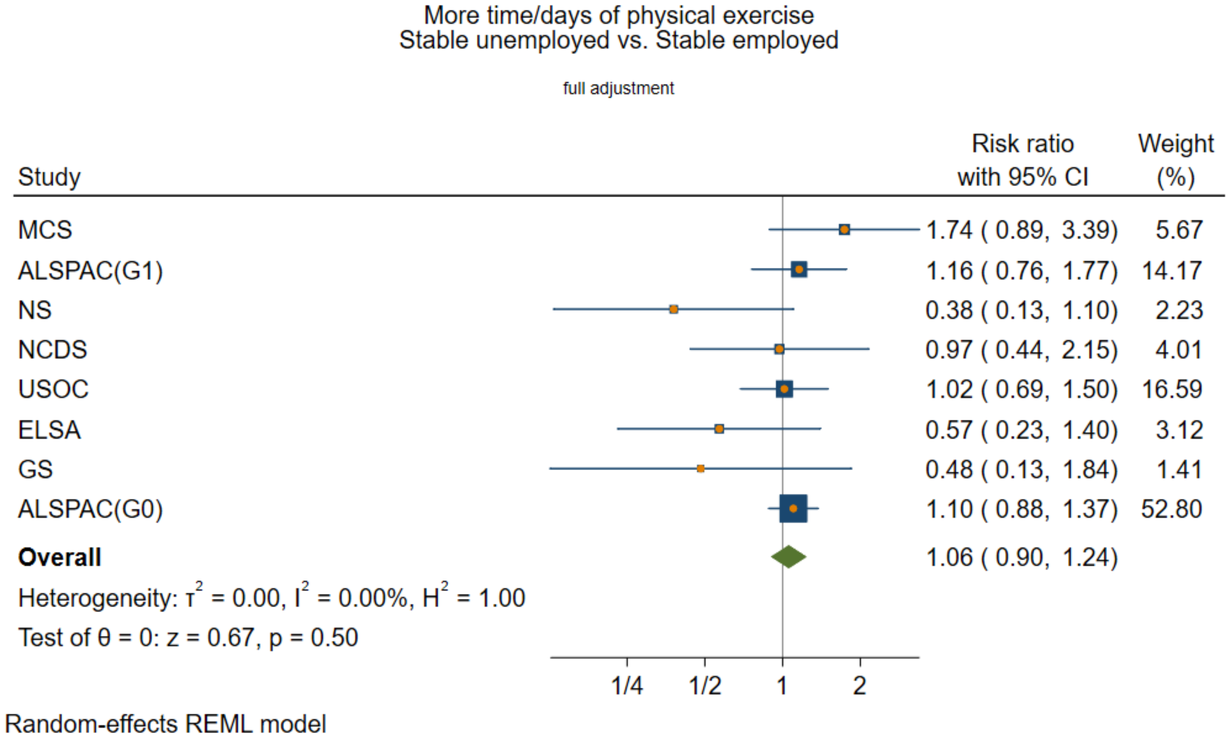
More time/days of physical exercise
Stable unemployed vs. Stable employed
full adjustment
Study
Risk ratio with 95% CI
Weight (%)
MCS 1.74 (0.89, 3.39) 5.67
ALSPAC(G1) 1.16 (0.76, 1.77) 14.17
NS 0.38 (0.13, 1.10) 2.23
NCDS 0.97 (0.44, 2.15) 4.01
USOC 1.02 (0.69, 1.50) 16.59
ELSA 0.57 (0.23, 1.40) 3.12
GS 0.48 (0.13, 1.84) 1.41
ALSPAC(G0) 1.10 (0.88, 1.37) 52.80
Overall 1.06 (0.90, 1.24)
Heterogeneity: τ² = 0.00, I² = 0.00%, H² = 1.00
Test of θ = 0: z = 0.67, p = 0.50
1/4
1/2
1
2
Random-effects REML model

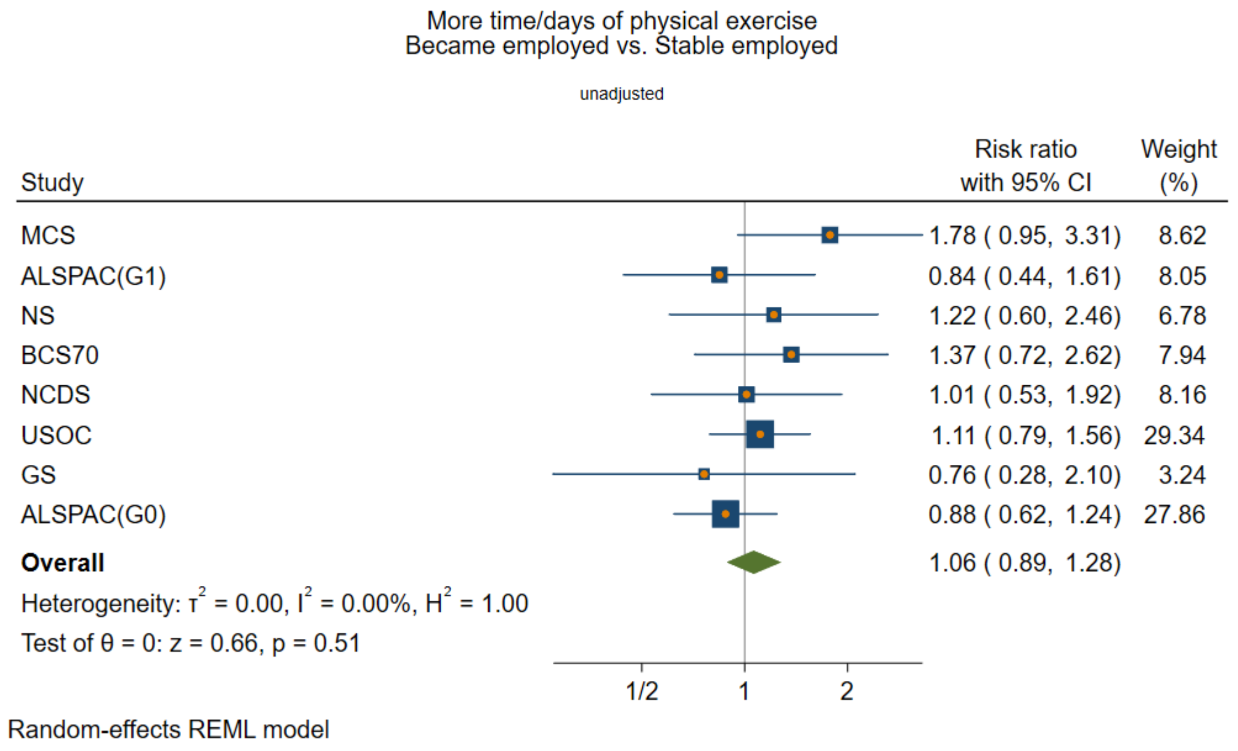
More time/days of physical exercise
Became employed vs. Stable employed
unadjusted
Study
Risk ratio with 95% CI
Weight (%)
MCS 1.78 (0.95, 3.31) 8.62
ALSPAC(G1) 0.84 (0.44, 1.61) 8.05
NS 1.22 (0.60, 2.46) 6.78
BCS70 1.37 (0.72, 2.62) 7.94
NCDS 1.01 (0.53, 1.92) 8.16
USOC 1.11 (0.79, 1.56) 29.34
GS 0.76 (0.28, 2.10) 3.24
ALSPAC(G0) 0.88 (0.62, 1.24) 27.86
Overall 1.06 (0.89, 1.28)
Heterogeneity: τ² = 0.00, I² = 0.00%, H² = 1.00
Test of θ = 0: z = 0.66, p = 0.51
1/2
1
2
Random-effects REML model

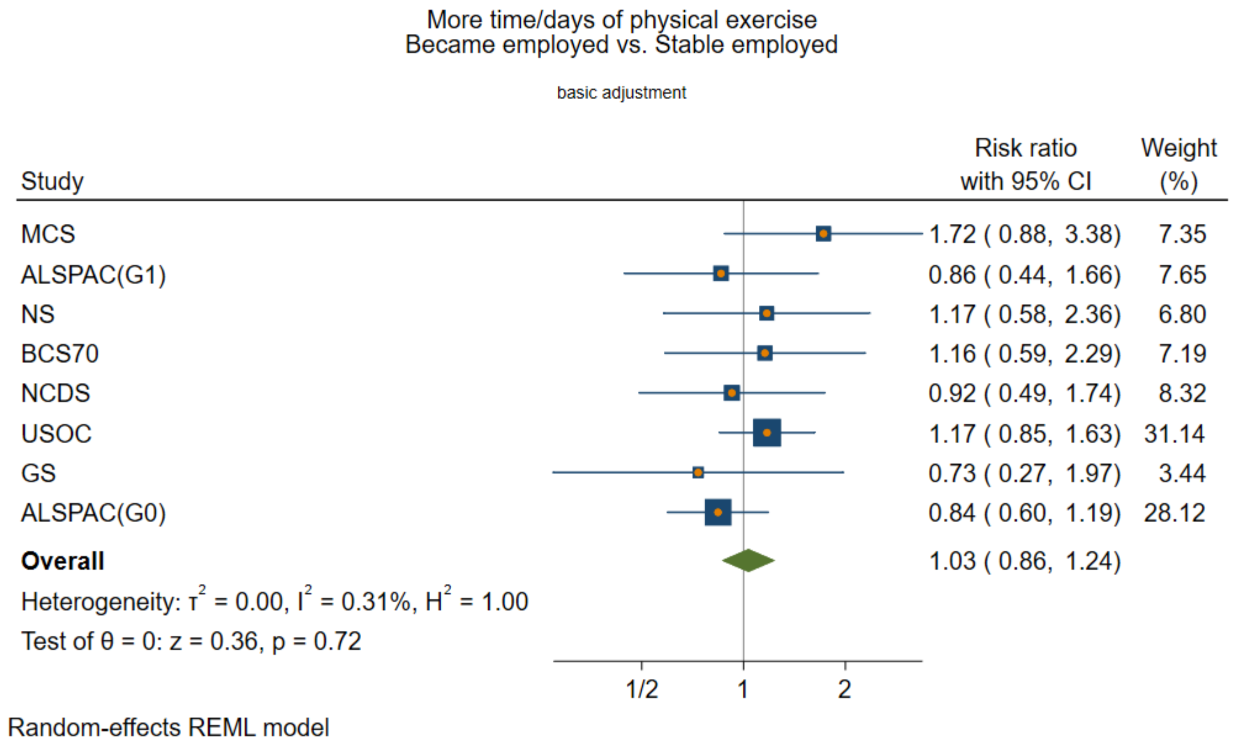
More time/days of physical exercise
Became employed vs. Stable employed
basic adjustment
Study
Risk ratio with 95% CI
Weight (%)
MCS 1.72 (0.88, 3.38) 7.35
ALSPAC(G1) 0.86 (0.44, 1.66) 7.65
NS 1.17 (0.58, 2.36) 6.80
BCS70 1.16 (0.59, 2.29) 7.19
NCDS 0.92 (0.49, 1.74) 8.32
USOC 1.17 (0.85, 1.63) 31.14
GS 0.73 (0.27, 1.97) 3.44
ALSPAC(G0) 0.84 (0.60, 1.19) 28.12
Overall 1.03 (0.86, 1.24)
Heterogeneity: τ² = 0.00, I² = 0.31%, H² = 1.00
Test of θ = 0: z = 0.36, p = 0.72
1/2
1
2
Random-effects REML model

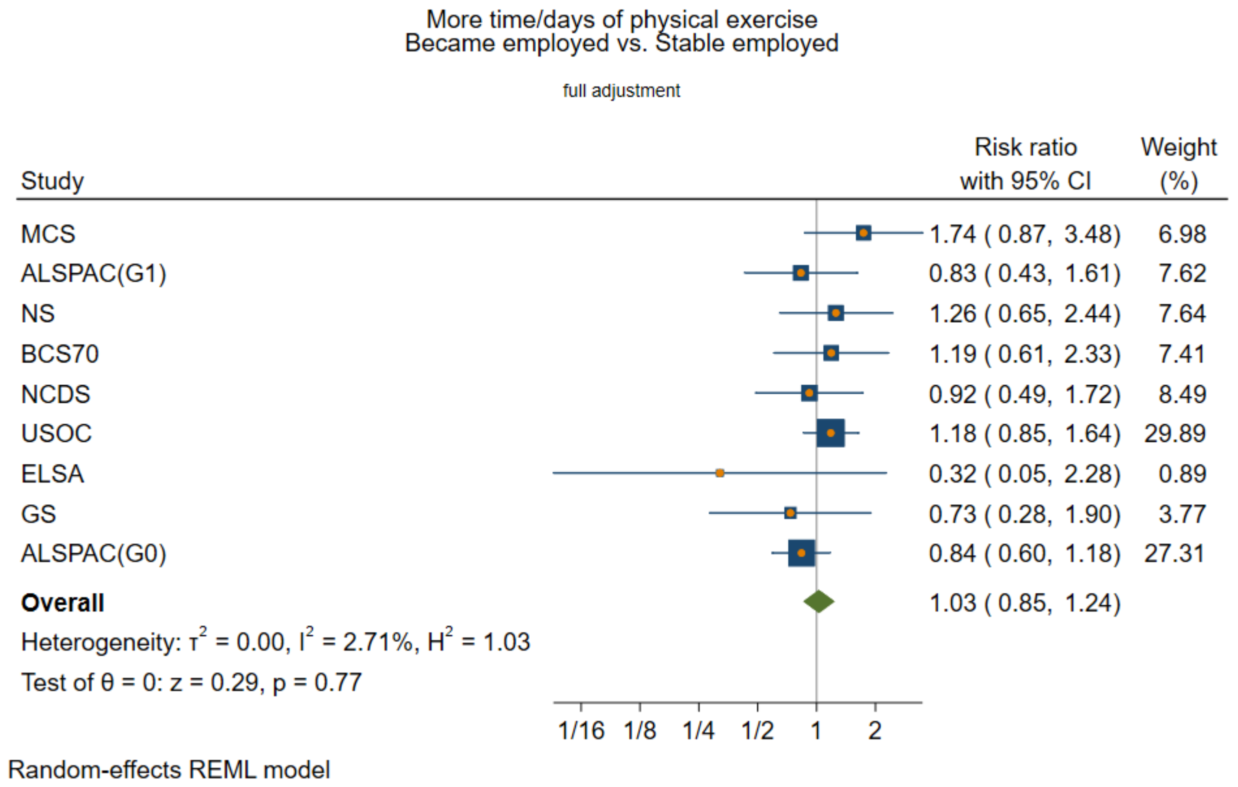
More time/days of physical exercise
Became employed vs. Stable employed
full adjustment
Study
Risk ratio with 95% CI
Weight (%)
MCS 1.74 (0.87, 3.48) 6.98
ALSPAC(G1) 0.83 (0.43, 1.61) 7.62
NS 1.26 (0.65, 2.44) 7.64
BCS70 1.19 (0.61, 2.33) 7.41
NCDS 0.92 (0.49, 1.72) 8.49
USOC 1.18 (0.85, 1.64) 29.89
ELSA 0.32 (0.05, 2.28) 0.89
GS 0.73 (0.28, 1.90) 3.77
ALSPAC(G0) 0.84 (0.60, 1.18) 27.31
Overall 1.03 (0.85, 1.24)
Heterogeneity: τ² = 0.00, I² = 2.71%, H² = 1.03
Test of θ = 0: z = 0.29, p = 0.77
1/16
1/8
1/4
1/2
1
2
Random-effects REML model

More time/days of physical exercise
Stable non-employed vs. Stable employed

unadjusted

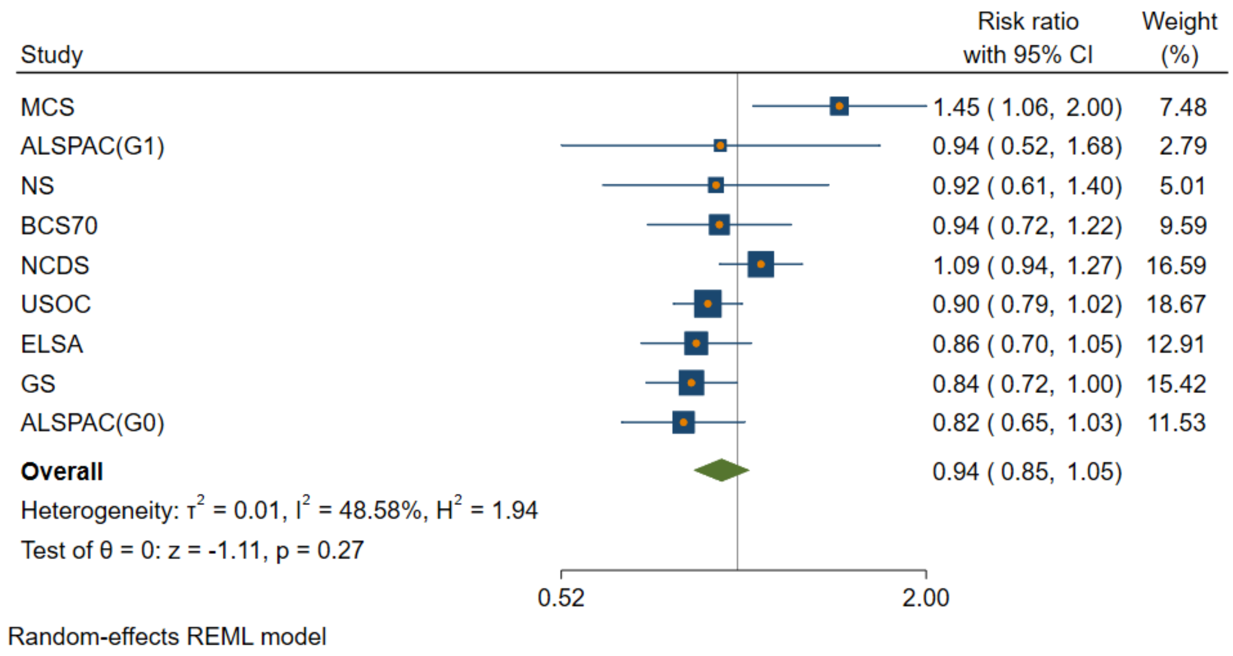

More time/days of physical exercise
Stable non-employed vs. Stable employed

basic adjustment

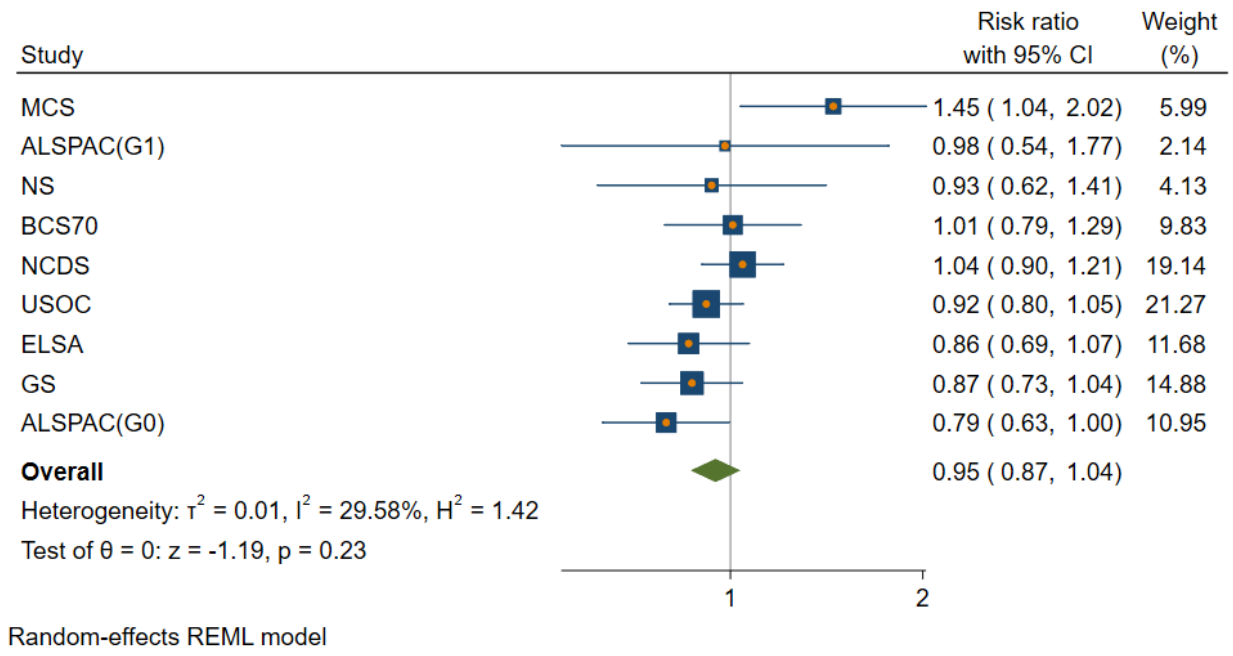

More time/days of physical exercise
Stable non-employed vs. Stable employed

full adjustment

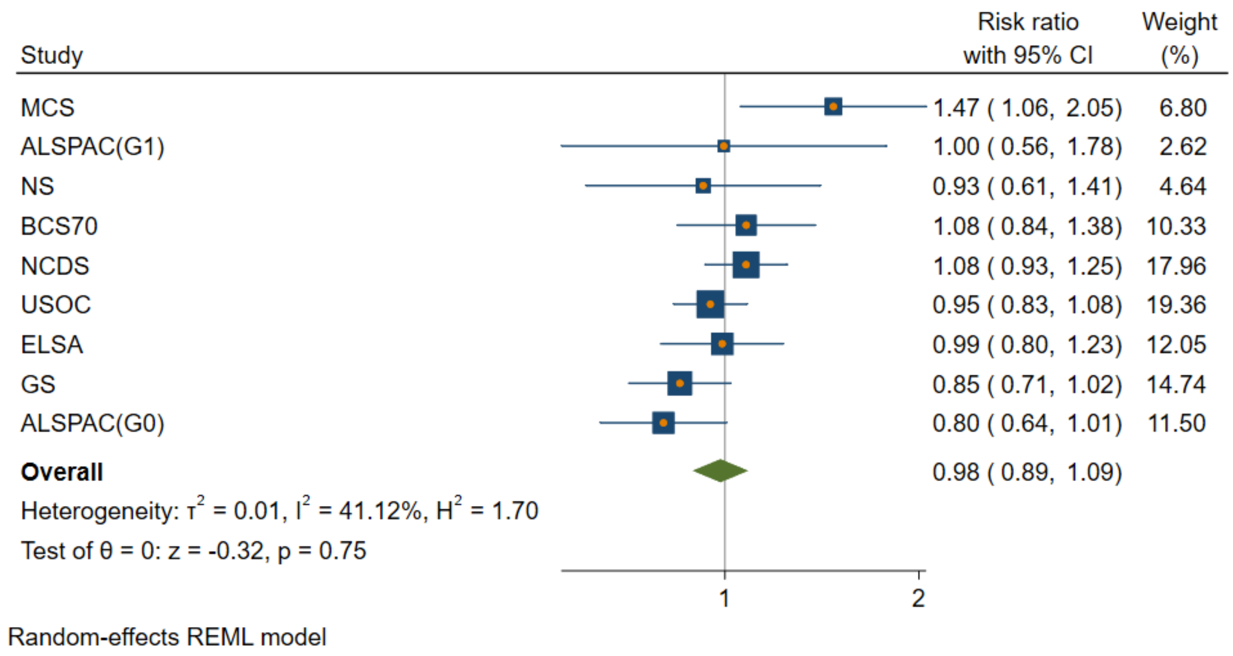

Figure set 7: Sleeps outside ‘Normal Range’ (i.e. <6 or 9+ hours)

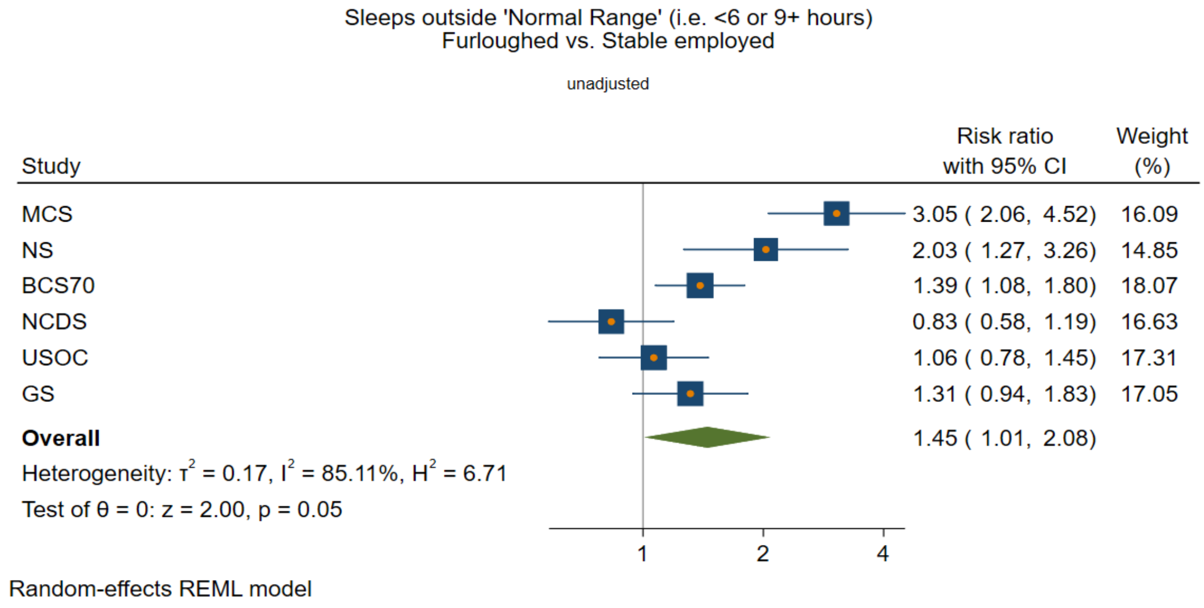

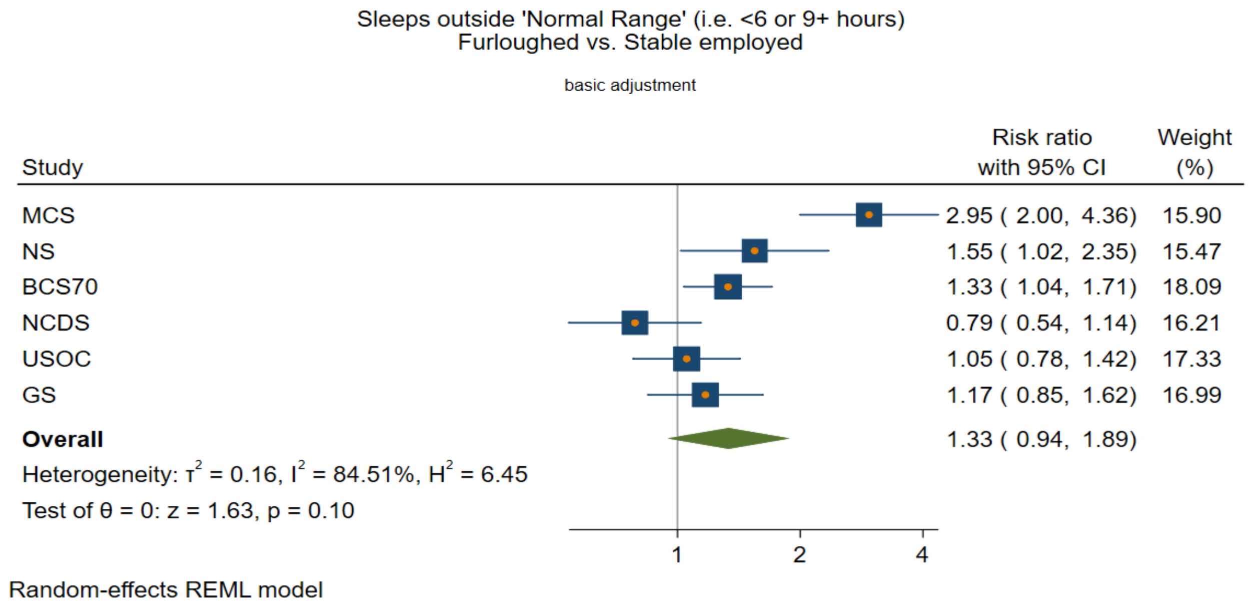

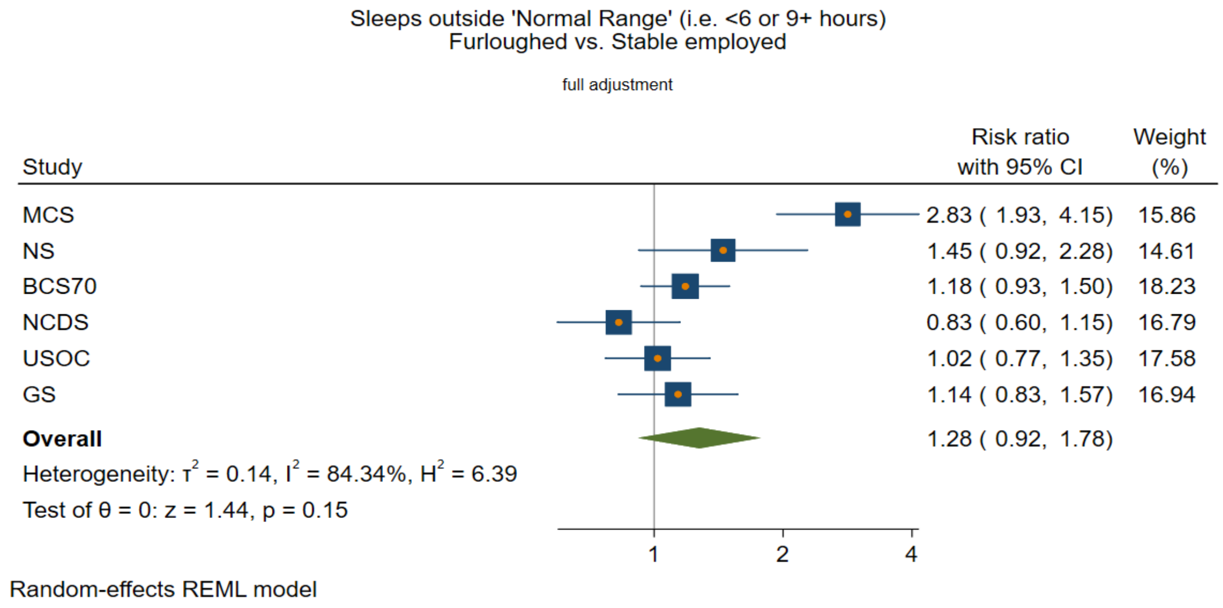

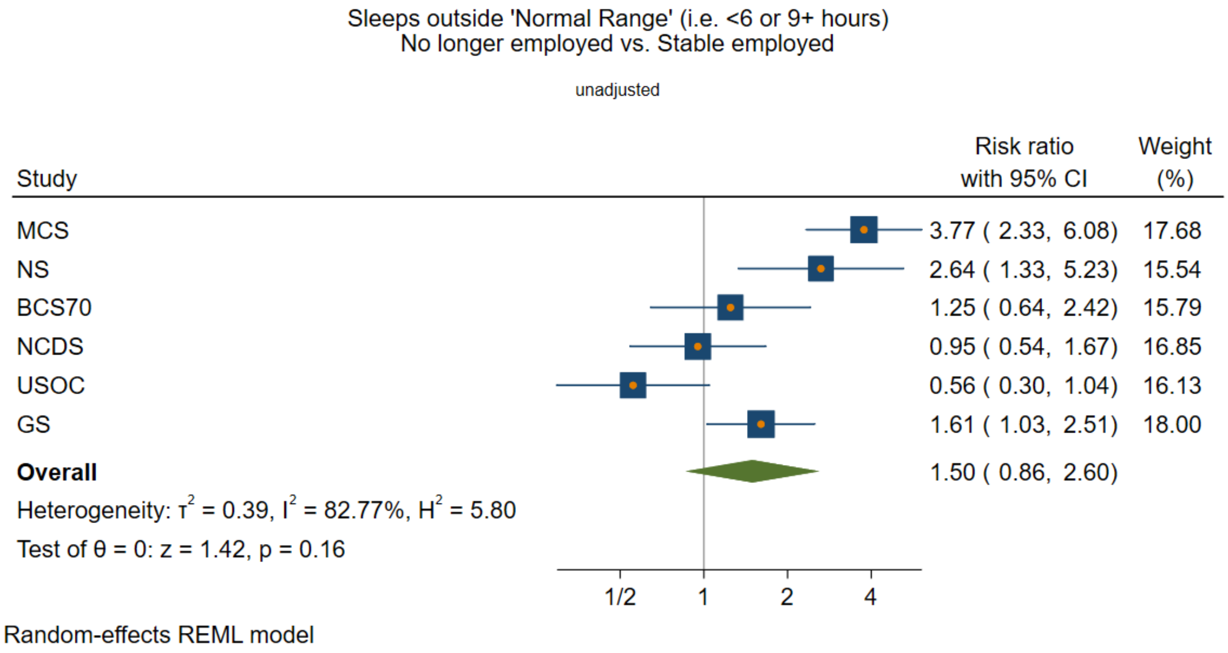
Sleeps outside 'Normal Range' (i.e. <6 or 9+ hours)
No longer employed vs. Stable employed
unadjusted
Study
Risk ratio with 95% CI
Weight (%)
MCS 3.77 (2.33, 6.08) 17.68
NS 2.64 (1.33, 5.23) 15.54
BCS70 1.25 (0.64, 2.42) 15.79
NCDS 0.95 (0.54, 1.67) 16.85
USOC 0.56 (0.30, 1.04) 16.13
GS 1.61 (1.03, 2.51) 18.00
Overall 1.50 (0.86, 2.60)
Heterogeneity: τ^2 = 0.39, I^2 = 82.77%, H^2 = 5.80
Test of θ = 0: z = 1.42, p = 0.16
1/2
1
2
4
Random-effects REML model

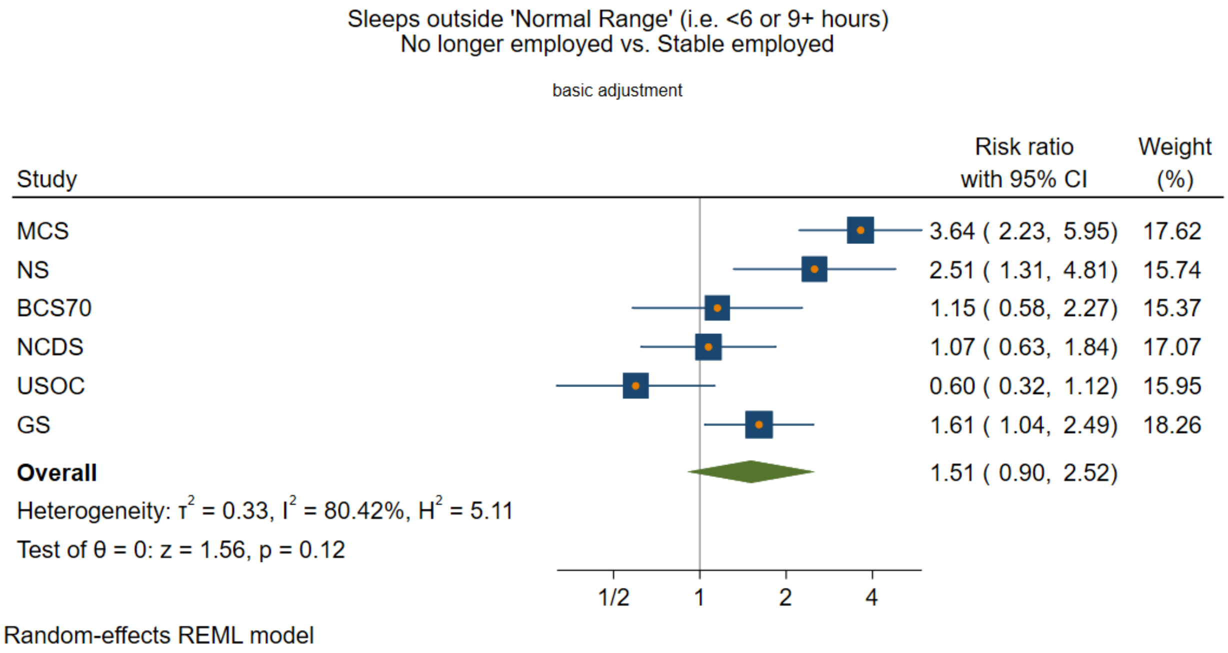
Sleeps outside 'Normal Range' (i.e. <6 or 9+ hours)
No longer employed vs. Stable employed
basic adjustment
Study
Risk ratio with 95% CI
Weight (%)
MCS 3.64 (2.23, 5.95) 17.62
NS 2.51 (1.31, 4.81) 15.74
BCS70 1.15 (0.58, 2.27) 15.37
NCDS 1.07 (0.63, 1.84) 17.07
USOC 0.60 (0.32, 1.12) 15.95
GS 1.61 (1.04, 2.49) 18.26
Overall 1.51 (0.90, 2.52)
Heterogeneity: τ^2 = 0.33, I^2 = 80.42%, H^2 = 5.11
Test of θ = 0: z = 1.56, p = 0.12
1/2
1
2
4
Random-effects REML model

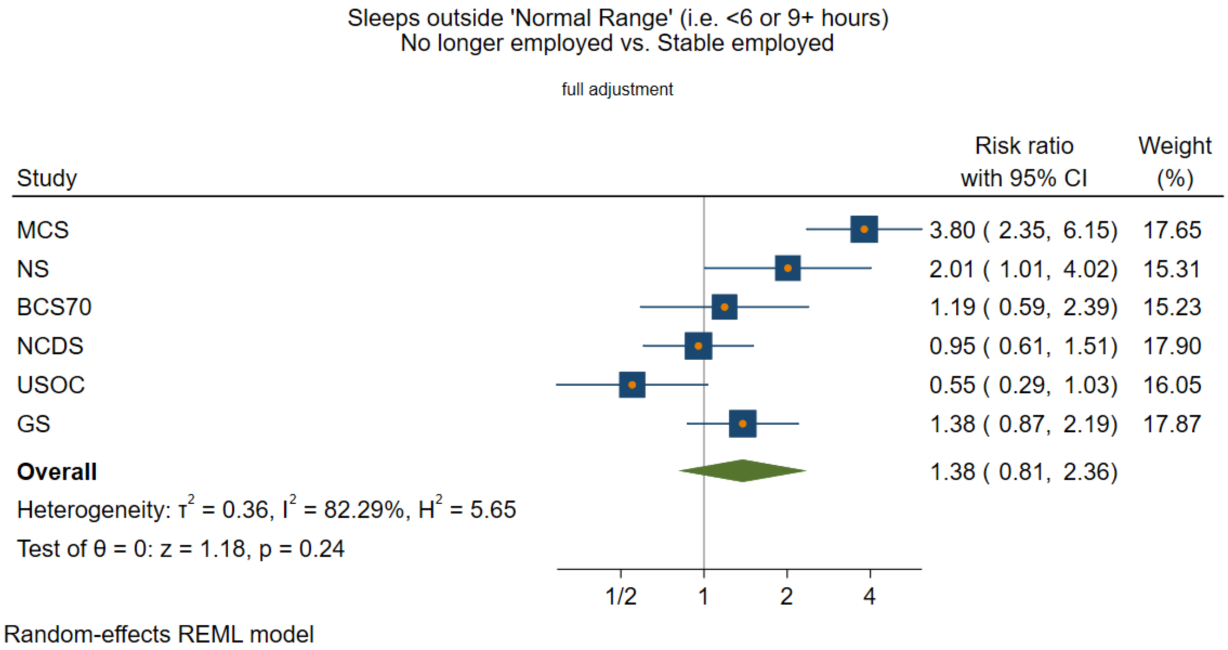
Sleeps outside 'Normal Range' (i.e. <6 or 9+ hours)
No longer employed vs. Stable employed
full adjustment
Study
Risk ratio with 95% CI
Weight (%)
MCS 3.80 (2.35, 6.15) 17.65
NS 2.01 (1.01, 4.02) 15.31
BCS70 1.19 (0.59, 2.39) 15.23
NCDS 0.95 (0.61, 1.51) 17.90
USOC 0.55 (0.29, 1.03) 16.05
GS 1.38 (0.87, 2.19) 17.87
Overall 1.38 (0.81, 2.36)
Heterogeneity: τ^2 = 0.36, I^2 = 82.29%, H^2 = 5.65
Test of θ = 0: z = 1.18, p = 0.24
1/2
1
2
4
Random-effects REML model

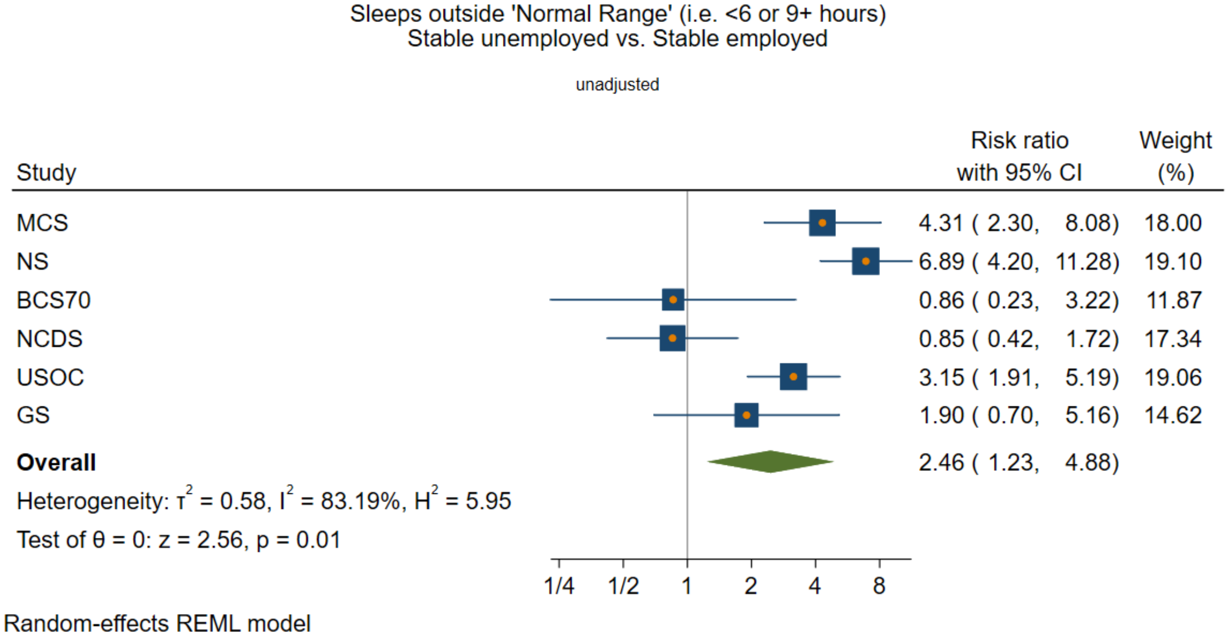
Sleeps outside 'Normal Range' (i.e. <6 or 9+ hours)
Stable unemployed vs. Stable employed
unadjusted
Study
Risk ratio with 95% CI
Weight (%)
MCS 4.31 (2.30, 8.08) 18.00
NS 6.89 (4.20, 11.28) 19.10
BCS70 0.86 (0.23, 3.22) 11.87
NCDS 0.85 (0.42, 1.72) 17.34
USOC 3.15 (1.91, 5.19) 19.06
GS 1.90 (0.70, 5.16) 14.62
Overall 2.46 (1.23, 4.88)
Heterogeneity: τ² = 0.58, I² = 83.19%, H² = 5.95
Test of θ = 0: z = 2.56, p = 0.01
1/4
1/2
1
2
4
8
Random-effects REML model

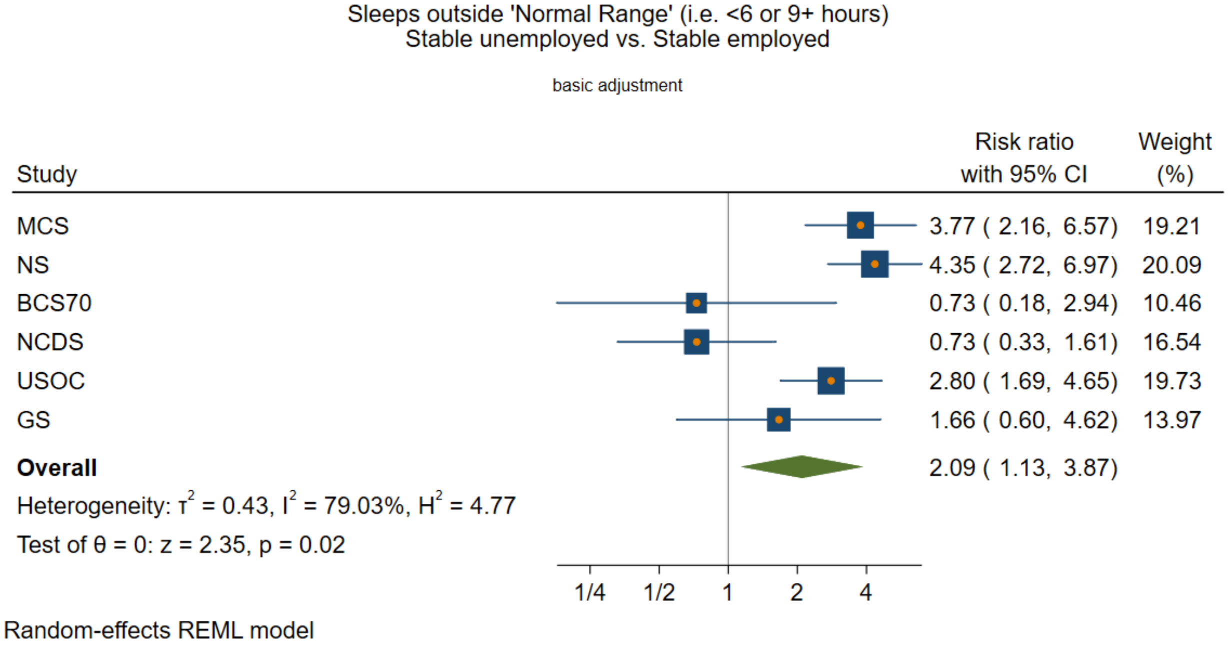
Sleeps outside 'Normal Range' (i.e. <6 or 9+ hours)
Stable unemployed vs. Stable employed
basic adjustment
Study
Risk ratio with 95% CI
Weight (%)
MCS 3.77 (2.16, 6.57) 19.21
NS 4.35 (2.72, 6.97) 20.09
BCS70 0.73 (0.18, 2.94) 10.46
NCDS 0.73 (0.33, 1.61) 16.54
USOC 2.80 (1.69, 4.65) 19.73
GS 1.66 (0.60, 4.62) 13.97
Overall 2.09 (1.13, 3.87)
Heterogeneity: τ² = 0.43, I² = 79.03%, H² = 4.77
Test of θ = 0: z = 2.35, p = 0.02
1/4
1/2
1
2
4
Random-effects REML model

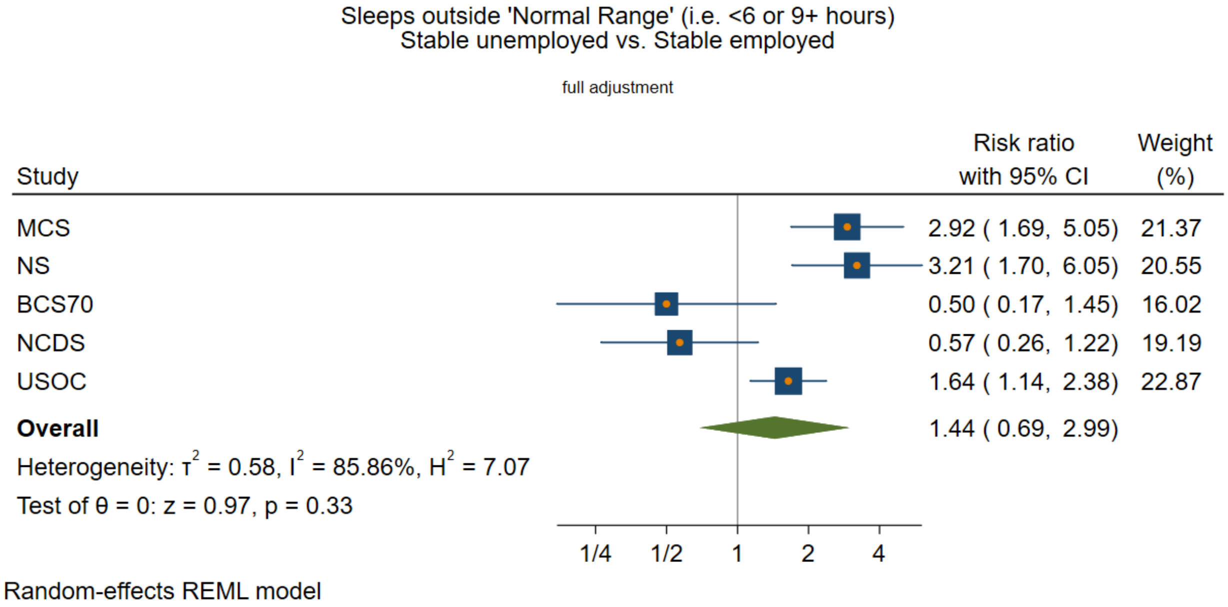
Sleeps outside 'Normal Range' (i.e. <6 or 9+ hours)
Stable unemployed vs. Stable employed
full adjustment
Study
Risk ratio with 95% CI
Weight (%)
MCS 2.92 (1.69, 5.05) 21.37
NS 3.21 (1.70, 6.05) 20.55
BCS70 0.50 (0.17, 1.45) 16.02
NCDS 0.57 (0.26, 1.22) 19.19
USOC 1.64 (1.14, 2.38) 22.87
Overall 1.44 (0.69, 2.99)
Heterogeneity: τ² = 0.58, I² = 85.86%, H² = 7.07
Test of θ = 0: z = 0.97, p = 0.33
1/4
1/2
1
2
4
Random-effects REML model

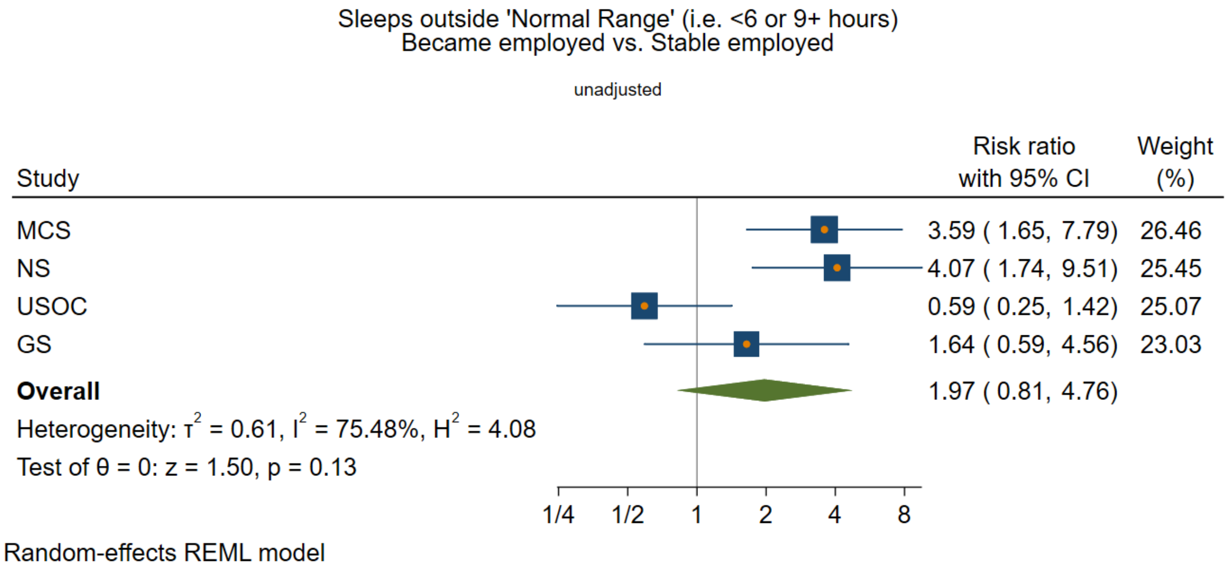
Sleeps outside 'Normal Range' (i.e. <6 or 9+ hours)
Became employed vs. Stable employed
unadjusted
Study
Risk ratio with 95% CI
Weight (%)
MCS 3.59 (1.65, 7.79) 26.46
NS 4.07 (1.74, 9.51) 25.45
USOC 0.59 (0.25, 1.42) 25.07
GS 1.64 (0.59, 4.56) 23.03
Overall 1.97 (0.81, 4.76)
Heterogeneity: τ² = 0.61, I² = 75.48%, H² = 4.08
Test of θ = 0: z = 1.50, p = 0.13
1/4
1/2
1
2
4
8
Random-effects REML model

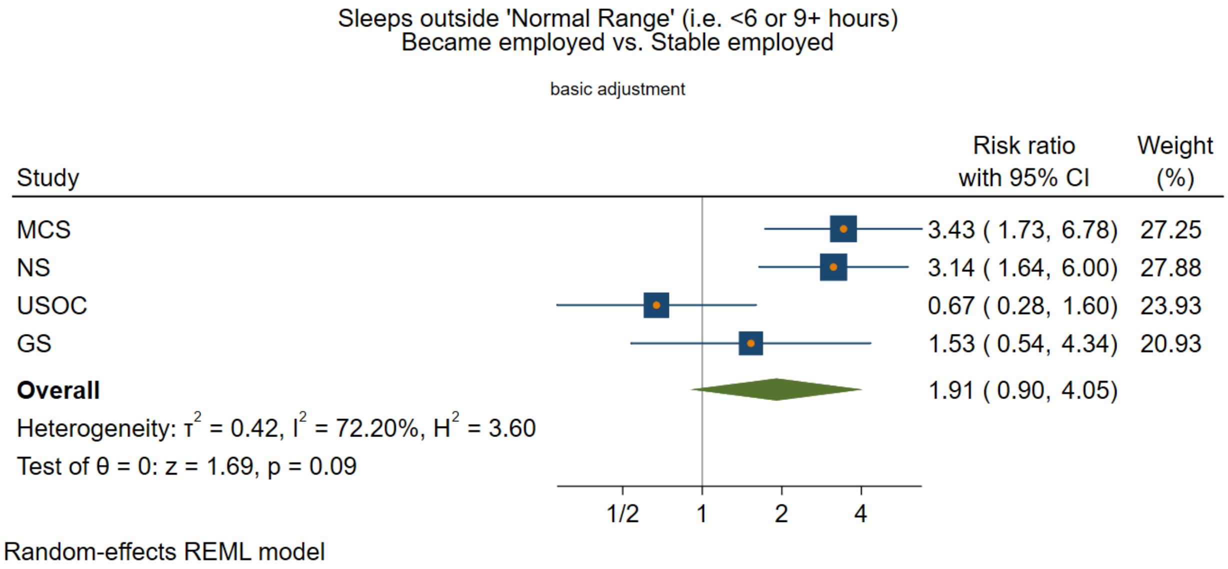
Sleeps outside 'Normal Range' (i.e. <6 or 9+ hours)
Became employed vs. Stable employed
basic adjustment
Study
Risk ratio with 95% CI
Weight (%)
MCS 3.43 (1.73, 6.78) 27.25
NS 3.14 (1.64, 6.00) 27.88
USOC 0.67 (0.28, 1.60) 23.93
GS 1.53 (0.54, 4.34) 20.93
Overall 1.91 (0.90, 4.05)
Heterogeneity: τ² = 0.42, I² = 72.20%, H² = 3.60
Test of θ = 0: z = 1.69, p = 0.09
1/2
1
2
4
Random-effects REML model

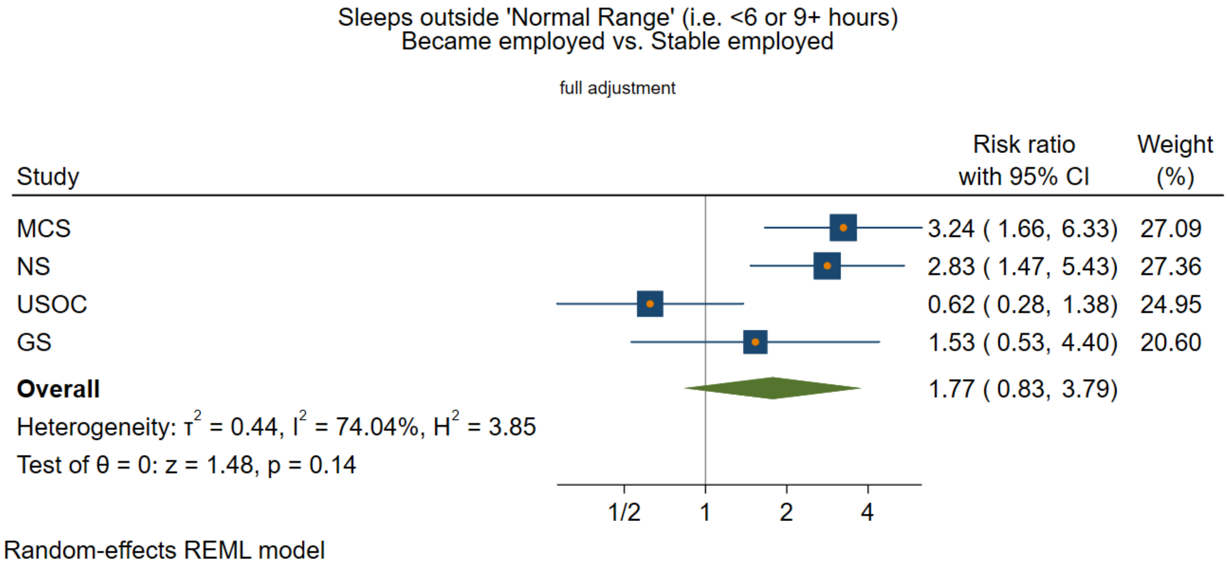
Sleeps outside 'Normal Range' (i.e. <6 or 9+ hours)
Became employed vs. Stable employed
full adjustment
Study
Risk ratio with 95% CI
Weight (%)
MCS 3.24 (1.66, 6.33) 27.09
NS 2.83 (1.47, 5.43) 27.36
USOC 0.62 (0.28, 1.38) 24.95
GS 1.53 (0.53, 4.40) 20.60
Overall 1.77 (0.83, 3.79)
Heterogeneity: τ² = 0.44, I² = 74.04%, H² = 3.85
Test of θ = 0: z = 1.48, p = 0.14
1/2
1
2
4
Random-effects REML model

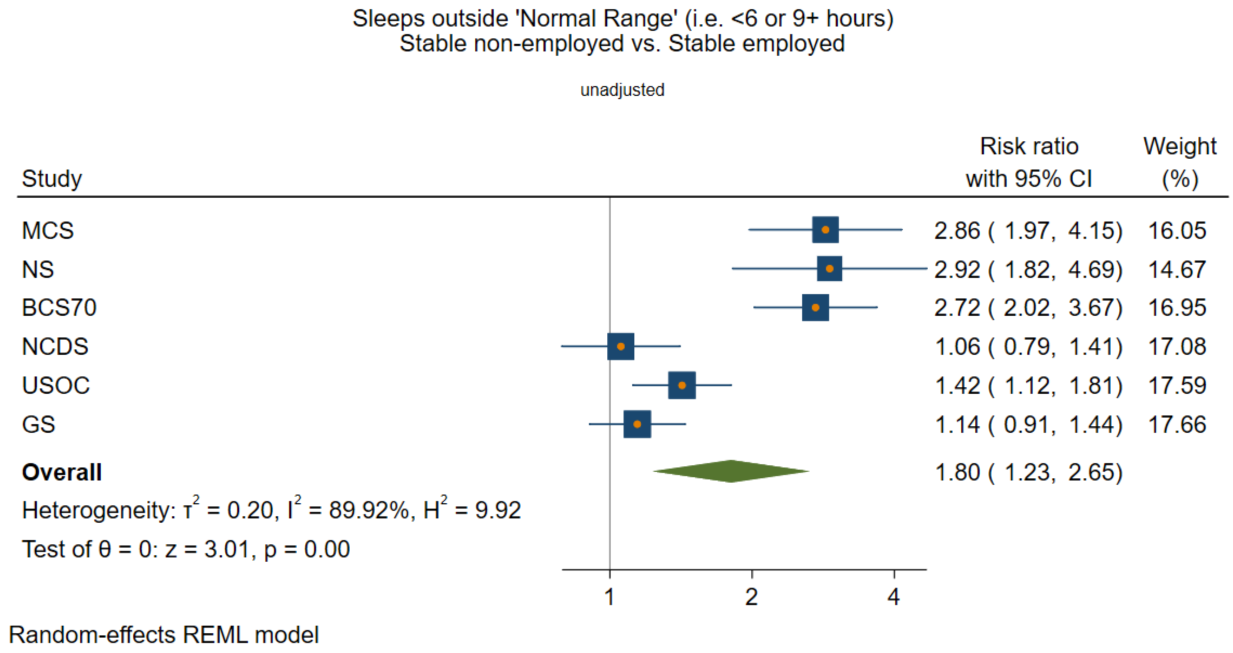
Sleeps outside 'Normal Range' (i.e. <6 or 9+ hours)
Stable non-employed vs. Stable employed
unadjusted
Study
Risk ratio with 95% CI
Weight (%)
MCS 2.86 (1.97, 4.15) 16.05
NS 2.92 (1.82, 4.69) 14.67
BCS70 2.72 (2.02, 3.67) 16.95
NCDS 1.06 (0.79, 1.41) 17.08
USOC 1.42 (1.12, 1.81) 17.59
GS 1.14 (0.91, 1.44) 17.66
Overall 1.80 (1.23, 2.65)
Heterogeneity: τ^2 = 0.20, I^2 = 89.92%, H^2 = 9.92
Test of θ = 0: z = 3.01, p = 0.00
1
2
4
Random-effects REML model

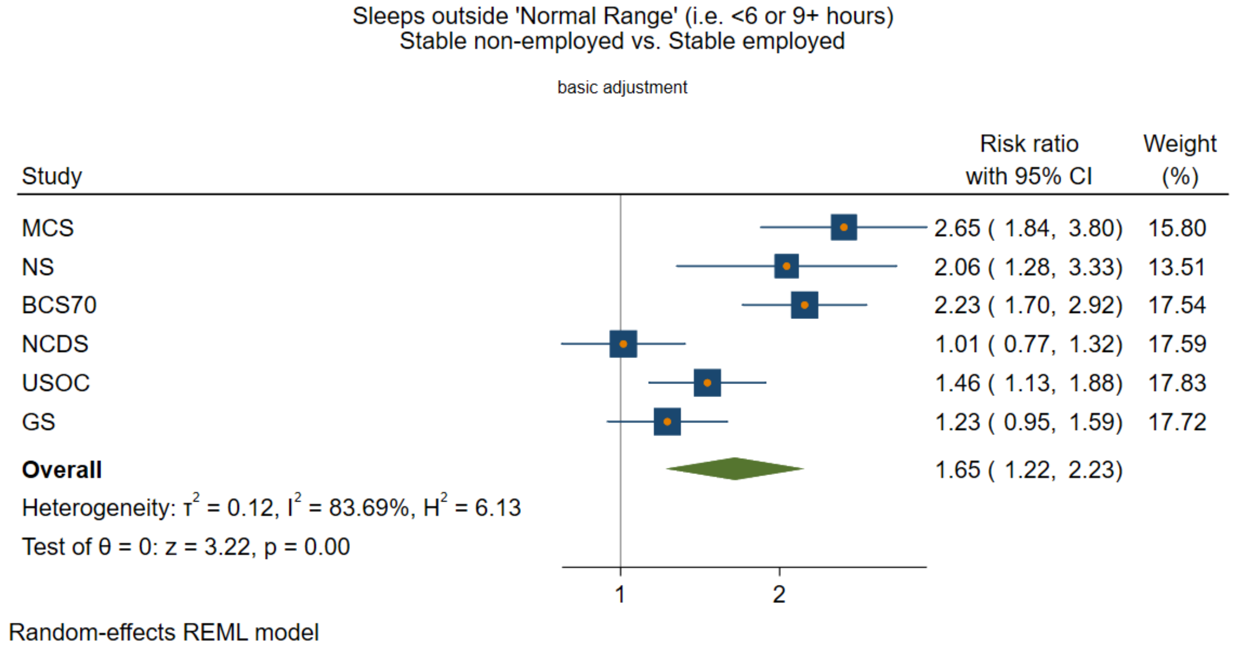
Sleeps outside 'Normal Range' (i.e. <6 or 9+ hours)
Stable non-employed vs. Stable employed
basic adjustment
Study
Risk ratio with 95% CI
Weight (%)
MCS 2.65 (1.84, 3.80) 15.80
NS 2.06 (1.28, 3.33) 13.51
BCS70 2.23 (1.70, 2.92) 17.54
NCDS 1.01 (0.77, 1.32) 17.59
USOC 1.46 (1.13, 1.88) 17.83
GS 1.23 (0.95, 1.59) 17.72
Overall 1.65 (1.22, 2.23)
Heterogeneity: τ^2 = 0.12, I^2 = 83.69%, H^2 = 6.13
Test of θ = 0: z = 3.22, p = 0.00
1
2
Random-effects REML model

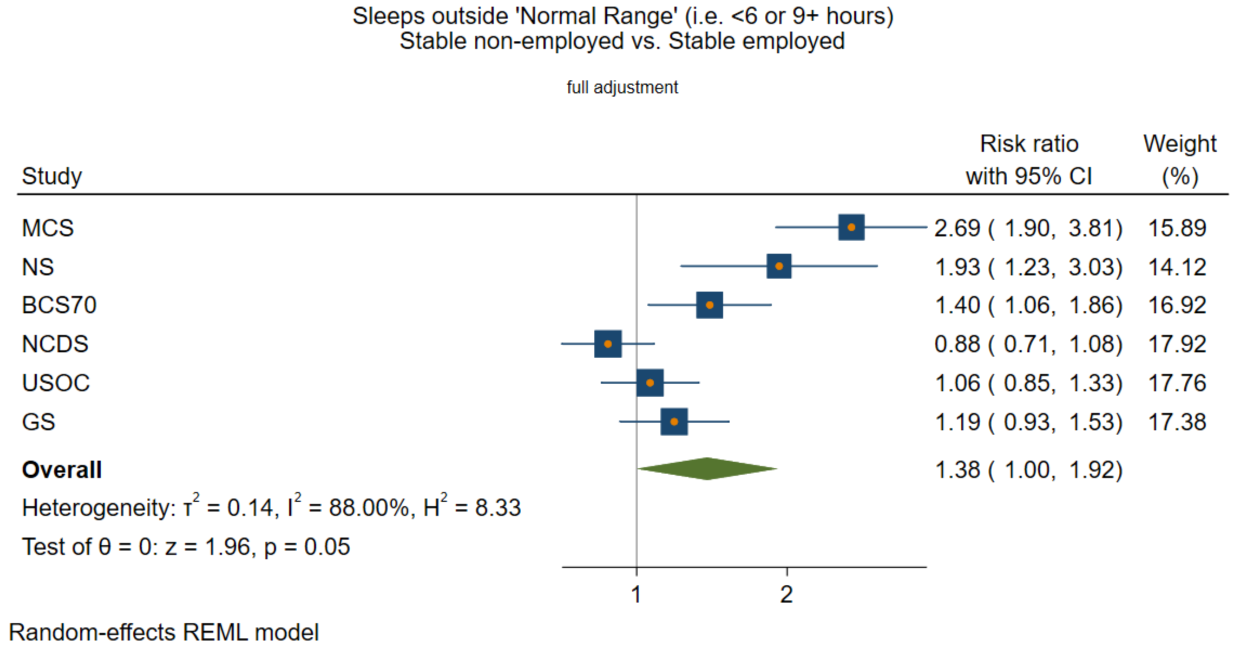
Sleeps outside 'Normal Range' (i.e. <6 or 9+ hours)
Stable non-employed vs. Stable employed
full adjustment
Study
Risk ratio with 95% CI
Weight (%)
MCS 2.69 (1.90, 3.81) 15.89
NS 1.93 (1.23, 3.03) 14.12
BCS70 1.40 (1.06, 1.86) 16.92
NCDS 0.88 (0.71, 1.08) 17.92
USOC 1.06 (0.85, 1.33) 17.76
GS 1.19 (0.93, 1.53) 17.38
Overall 1.38 (1.00, 1.92)
Heterogeneity: τ^2 = 0.14, I^2 = 88.00%, H^2 = 8.33
Test of θ = 0: z = 1.96, p = 0.05
1
2
Random-effects REML model

Figure set 8: Sleeps less than before

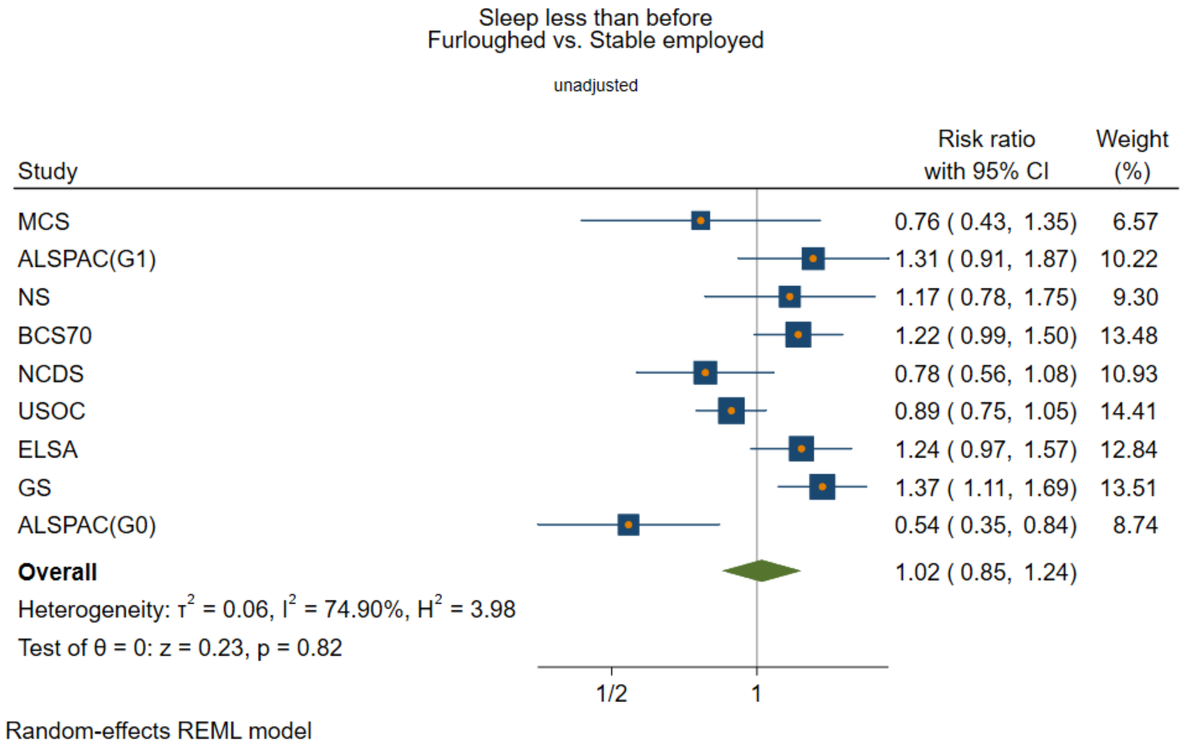

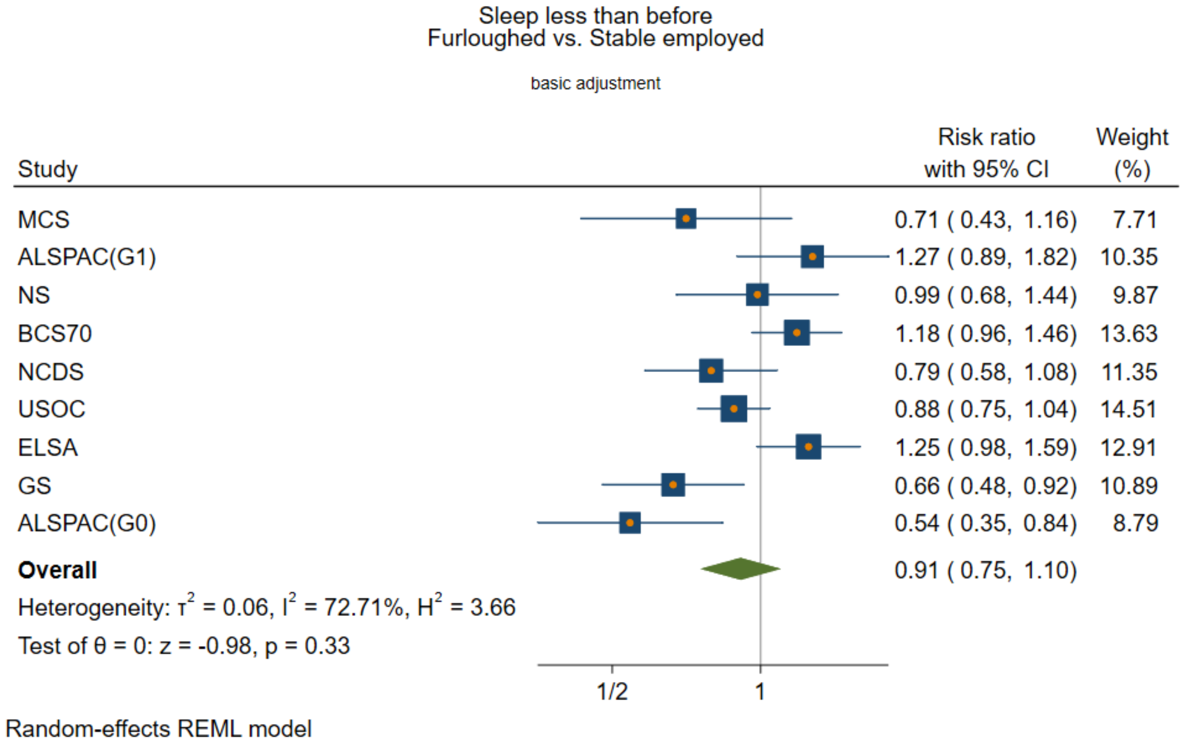

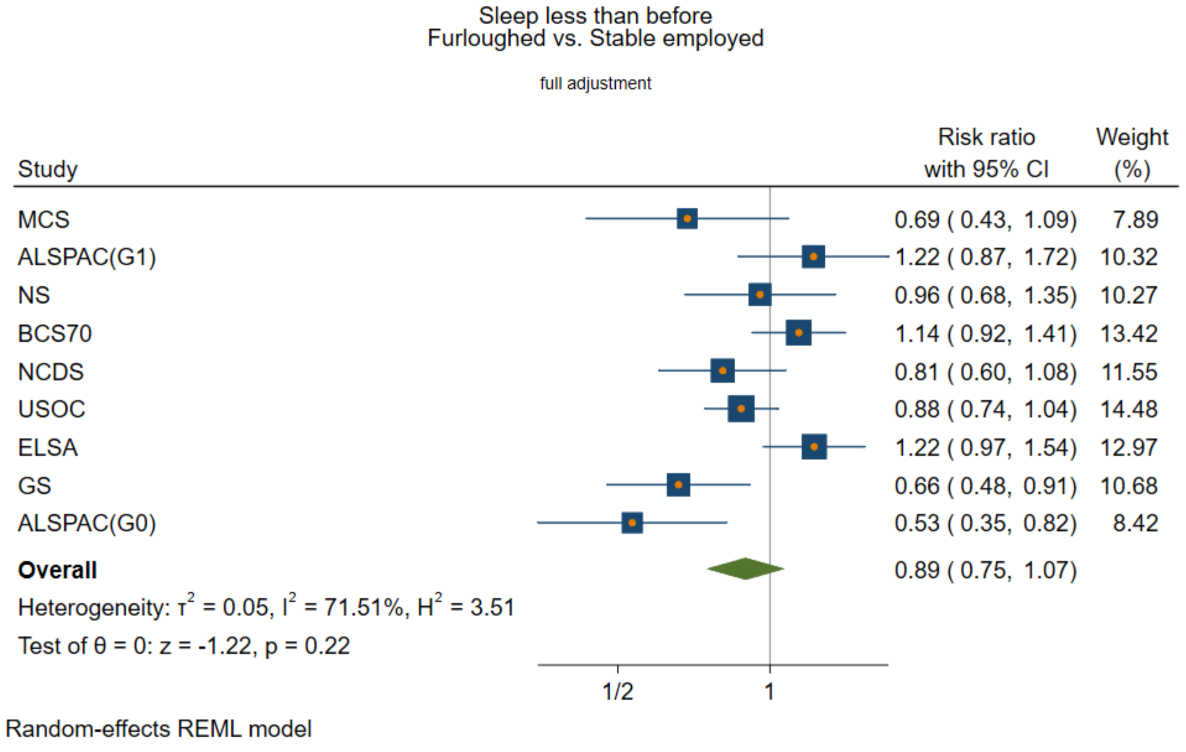

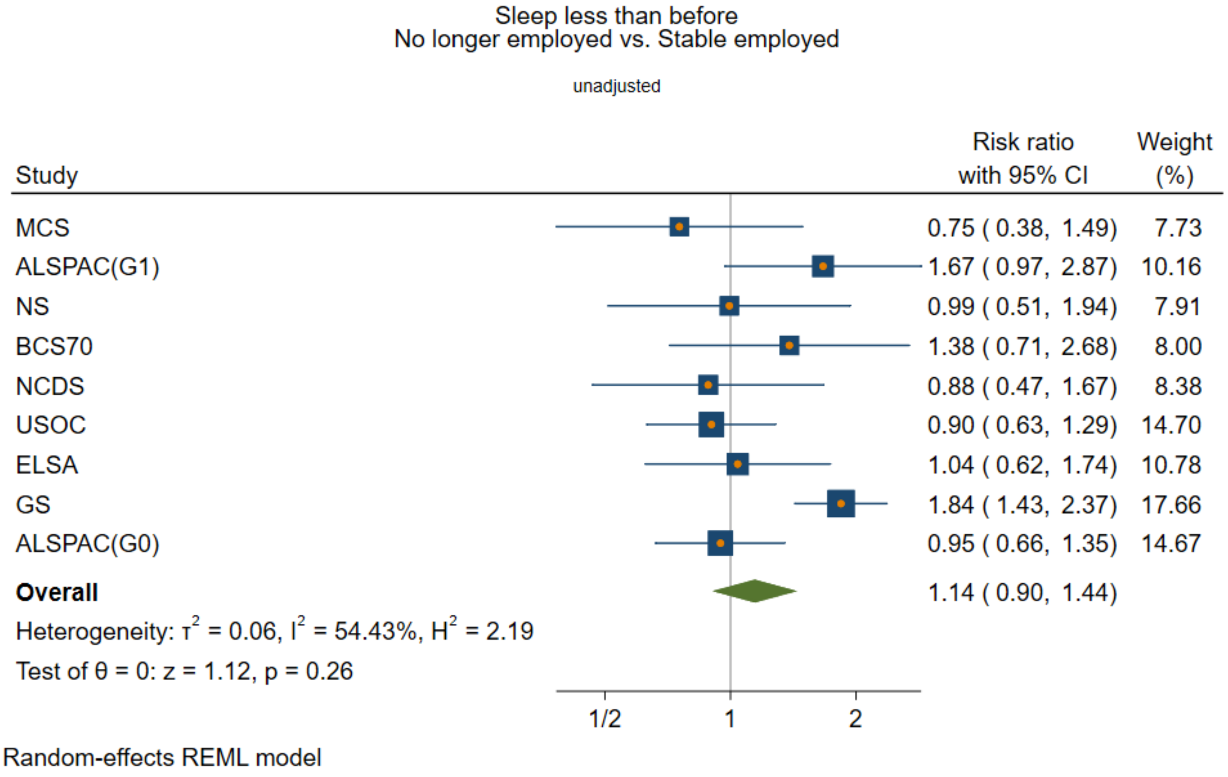
Sleep less than before
No longer employed vs. Stable employed
unadjusted
Study
Risk ratio with 95% CI
Weight (%)
MCS 0.75 (0.38, 1.49) 7.73
ALSPAC(G1) 1.67 (0.97, 2.87) 10.16
NS 0.99 (0.51, 1.94) 7.91
BCS70 1.38 (0.71, 2.68) 8.00
NCDS 0.88 (0.47, 1.67) 8.38
USOC 0.90 (0.63, 1.29) 14.70
ELSA 1.04 (0.62, 1.74) 10.78
GS 1.84 (1.43, 2.37) 17.66
ALSPAC(G0) 0.95 (0.66, 1.35) 14.67
Overall 1.14 (0.90, 1.44)
Heterogeneity: τ^2 = 0.06, I^2 = 54.43%, H^2 = 2.19
Test of θ = 0: z = 1.12, p = 0.26
1/2
1
2
Random-effects REML model

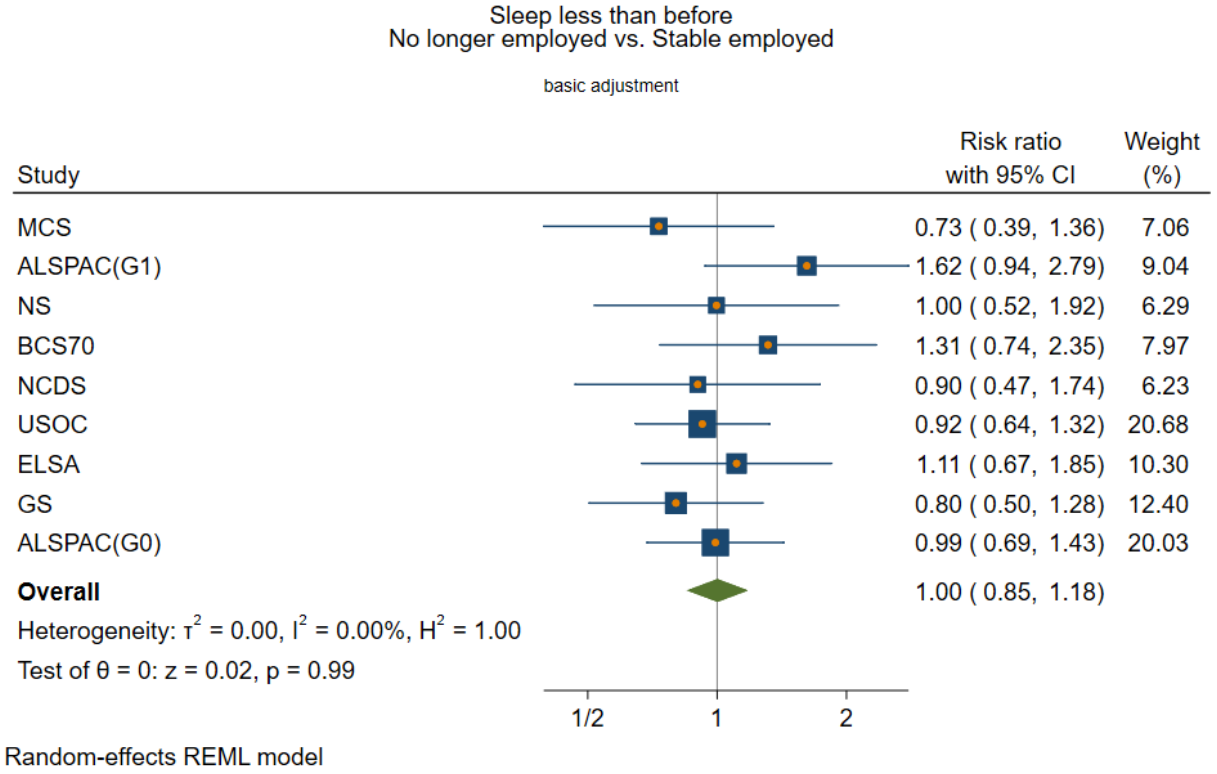
Sleep less than before
No longer employed vs. Stable employed
basic adjustment
Study
Risk ratio with 95% CI
Weight (%)
MCS 0.73 (0.39, 1.36) 7.06
ALSPAC(G1) 1.62 (0.94, 2.79) 9.04
NS 1.00 (0.52, 1.92) 6.29
BCS70 1.31 (0.74, 2.35) 7.97
NCDS 0.90 (0.47, 1.74) 6.23
USOC 0.92 (0.64, 1.32) 20.68
ELSA 1.11 (0.67, 1.85) 10.30
GS 0.80 (0.50, 1.28) 12.40
ALSPAC(G0) 0.99 (0.69, 1.43) 20.03
Overall 1.00 (0.85, 1.18)
Heterogeneity: τ^2 = 0.00, I^2 = 0.00%, H^2 = 1.00
Test of θ = 0: z = 0.02, p = 0.99
1/2
1
2
Random-effects REML model

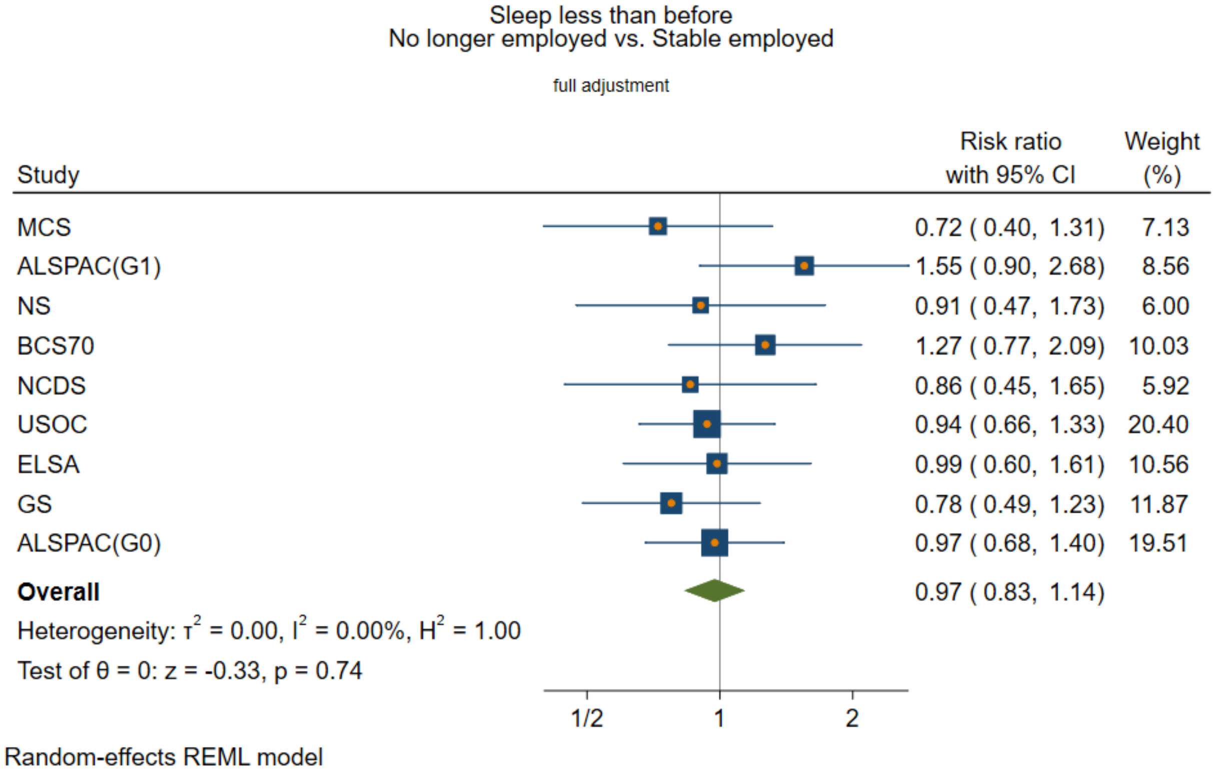
Sleep less than before
No longer employed vs. Stable employed
full adjustment
Study
Risk ratio with 95% CI
Weight (%)
MCS 0.72 (0.40, 1.31) 7.13
ALSPAC(G1) 1.55 (0.90, 2.68) 8.56
NS 0.91 (0.47, 1.73) 6.00
BCS70 1.27 (0.77, 2.09) 10.03
NCDS 0.86 (0.45, 1.65) 5.92
USOC 0.94 (0.66, 1.33) 20.40
ELSA 0.99 (0.60, 1.61) 10.56
GS 0.78 (0.49, 1.23) 11.87
ALSPAC(G0) 0.97 (0.68, 1.40) 19.51
Overall 0.97 (0.83, 1.14)
Heterogeneity: τ^2 = 0.00, I^2 = 0.00%, H^2 = 1.00
Test of θ = 0: z = -0.33, p = 0.74
1/2
1
2
Random-effects REML model

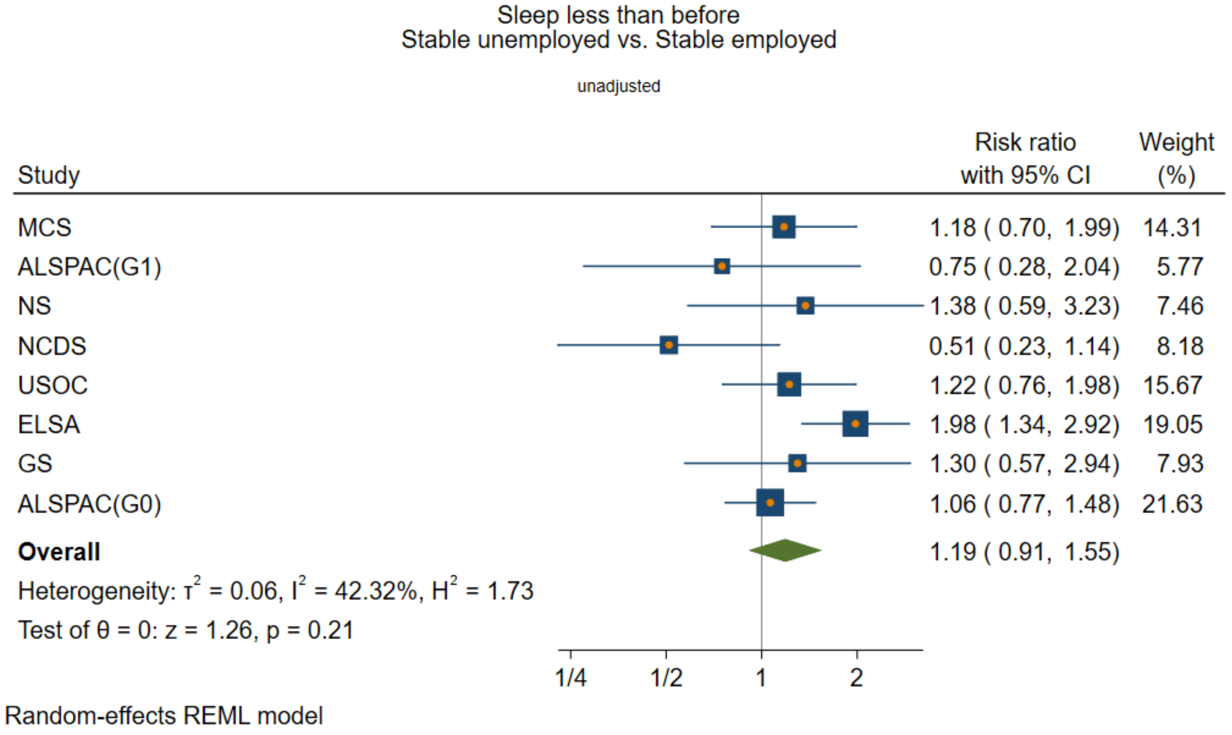
Sleep less than before
Stable unemployed vs. Stable employed
unadjusted
Study
Risk ratio with 95% CI
Weight (%)
MCS 1.18 (0.70, 1.99) 14.31
ALSPAC(G1) 0.75 (0.28, 2.04) 5.77
NS 1.38 (0.59, 3.23) 7.46
NCDS 0.51 (0.23, 1.14) 8.18
USOC 1.22 (0.76, 1.98) 15.67
ELSA 1.98 (1.34, 2.92) 19.05
GS 1.30 (0.57, 2.94) 7.93
ALSPAC(G0) 1.06 (0.77, 1.48) 21.63
Overall 1.19 (0.91, 1.55)
Heterogeneity: τ² = 0.06, I² = 42.32%, H² = 1.73
Test of θ = 0: z = 1.26, p = 0.21
1/4
1/2
1
2
Random-effects REML model

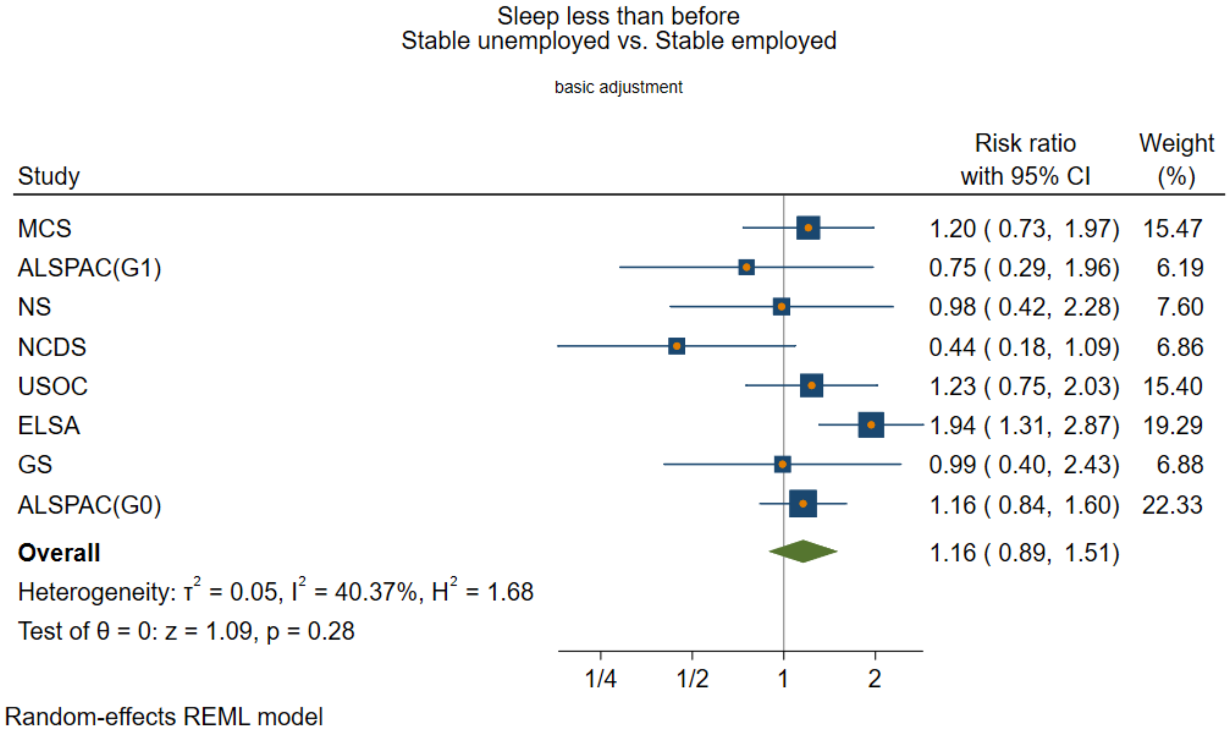
Sleep less than before
Stable unemployed vs. Stable employed
basic adjustment
Study
Risk ratio with 95% CI
Weight (%)
MCS 1.20 (0.73, 1.97) 15.47
ALSPAC(G1) 0.75 (0.29, 1.96) 6.19
NS 0.98 (0.42, 2.28) 7.60
NCDS 0.44 (0.18, 1.09) 6.86
USOC 1.23 (0.75, 2.03) 15.40
ELSA 1.94 (1.31, 2.87) 19.29
GS 0.99 (0.40, 2.43) 6.88
ALSPAC(G0) 1.16 (0.84, 1.60) 22.33
Overall 1.16 (0.89, 1.51)
Heterogeneity: τ² = 0.05, I² = 40.37%, H² = 1.68
Test of θ = 0: z = 1.09, p = 0.28
1/4
1/2
1
2
Random-effects REML model

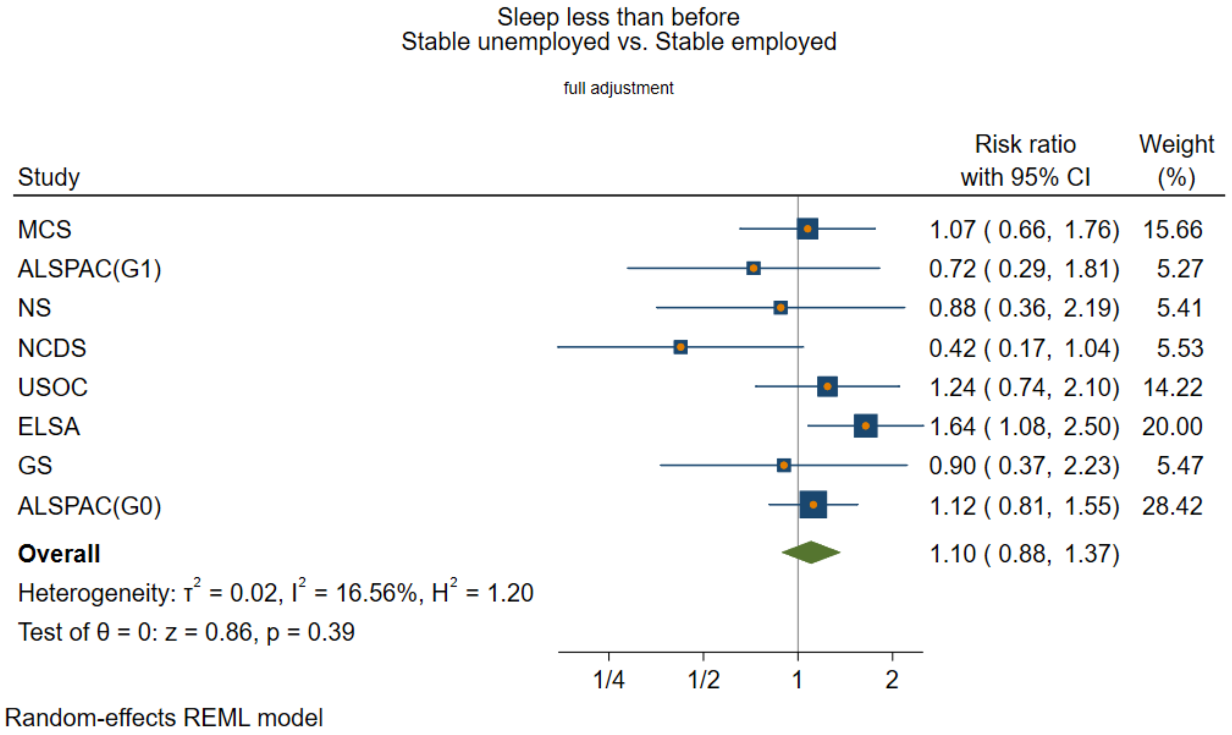
Sleep less than before
Stable unemployed vs. Stable employed
full adjustment
Study
Risk ratio with 95% CI
Weight (%)
MCS 1.07 (0.66, 1.76) 15.66
ALSPAC(G1) 0.72 (0.29, 1.81) 5.27
NS 0.88 (0.36, 2.19) 5.41
NCDS 0.42 (0.17, 1.04) 5.53
USOC 1.24 (0.74, 2.10) 14.22
ELSA 1.64 (1.08, 2.50) 20.00
GS 0.90 (0.37, 2.23) 5.47
ALSPAC(G0) 1.12 (0.81, 1.55) 28.42
Overall 1.10 (0.88, 1.37)
Heterogeneity: τ² = 0.02, I² = 16.56%, H² = 1.20
Test of θ = 0: z = 0.86, p = 0.39
1/4
1/2
1
2
Random-effects REML model

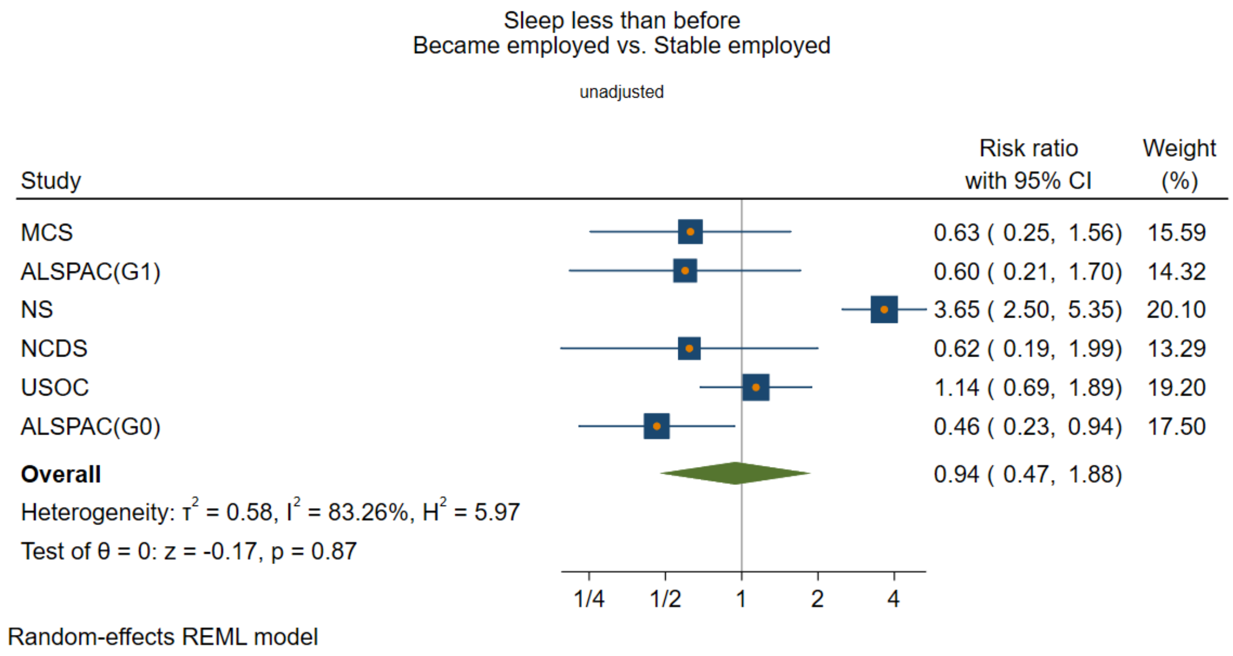
Sleep less than before
Became employed vs. Stable employed
unadjusted
Study
Risk ratio with 95% CI
Weight (%)
MCS 0.63 (0.25, 1.56) 15.59
ALSPAC(G1) 0.60 (0.21, 1.70) 14.32
NS 3.65 (2.50, 5.35) 20.10
NCDS 0.62 (0.19, 1.99) 13.29
USOC 1.14 (0.69, 1.89) 19.20
ALSPAC(G0) 0.46 (0.23, 0.94) 17.50
Overall 0.94 (0.47, 1.88)
Heterogeneity: τ² = 0.58, I² = 83.26%, H² = 5.97
Test of θ = 0: z = -0.17, p = 0.87
1/4 1/2 1 2 4
Random-effects REML model

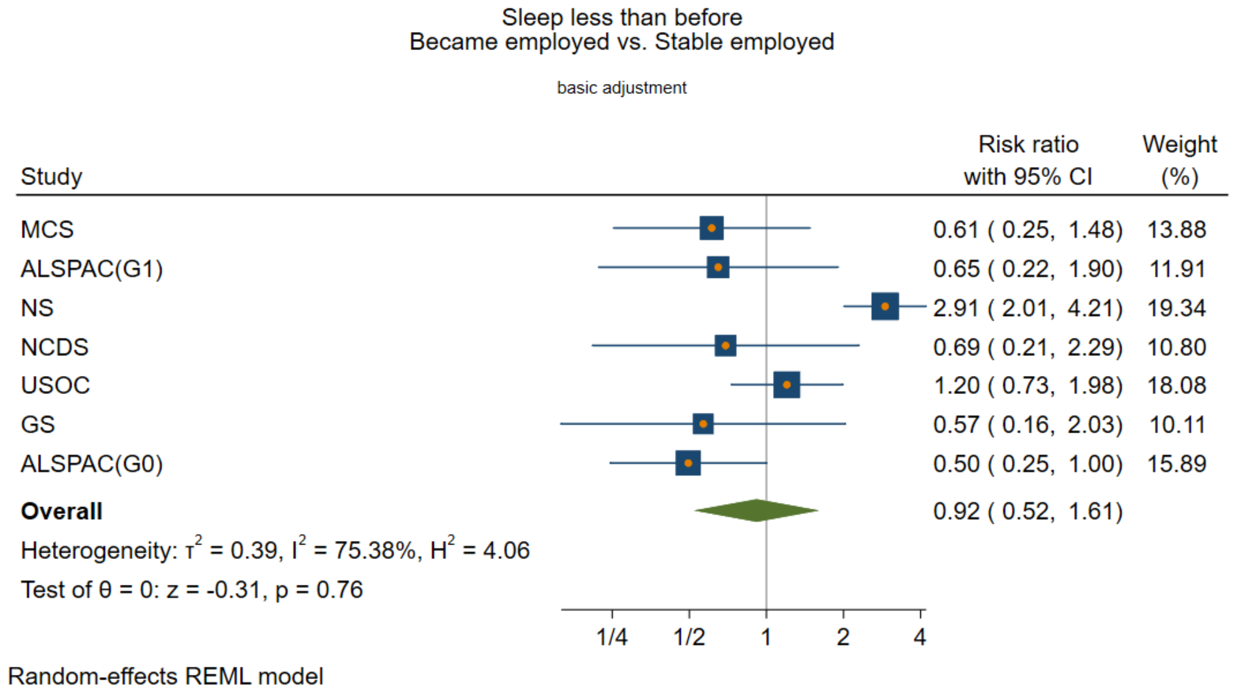
Sleep less than before
Became employed vs. Stable employed
basic adjustment
Study
Risk ratio with 95% CI
Weight (%)
MCS 0.61 (0.25, 1.48) 13.88
ALSPAC(G1) 0.65 (0.22, 1.90) 11.91
NS 2.91 (2.01, 4.21) 19.34
NCDS 0.69 (0.21, 2.29) 10.80
USOC 1.20 (0.73, 1.98) 18.08
GS 0.57 (0.16, 2.03) 10.11
ALSPAC(G0) 0.50 (0.25, 1.00) 15.89
Overall 0.92 (0.52, 1.61)
Heterogeneity: τ² = 0.39, I² = 75.38%, H² = 4.06
Test of θ = 0: z = -0.31, p = 0.76
1/4 1/2 1 2 4
Random-effects REML model

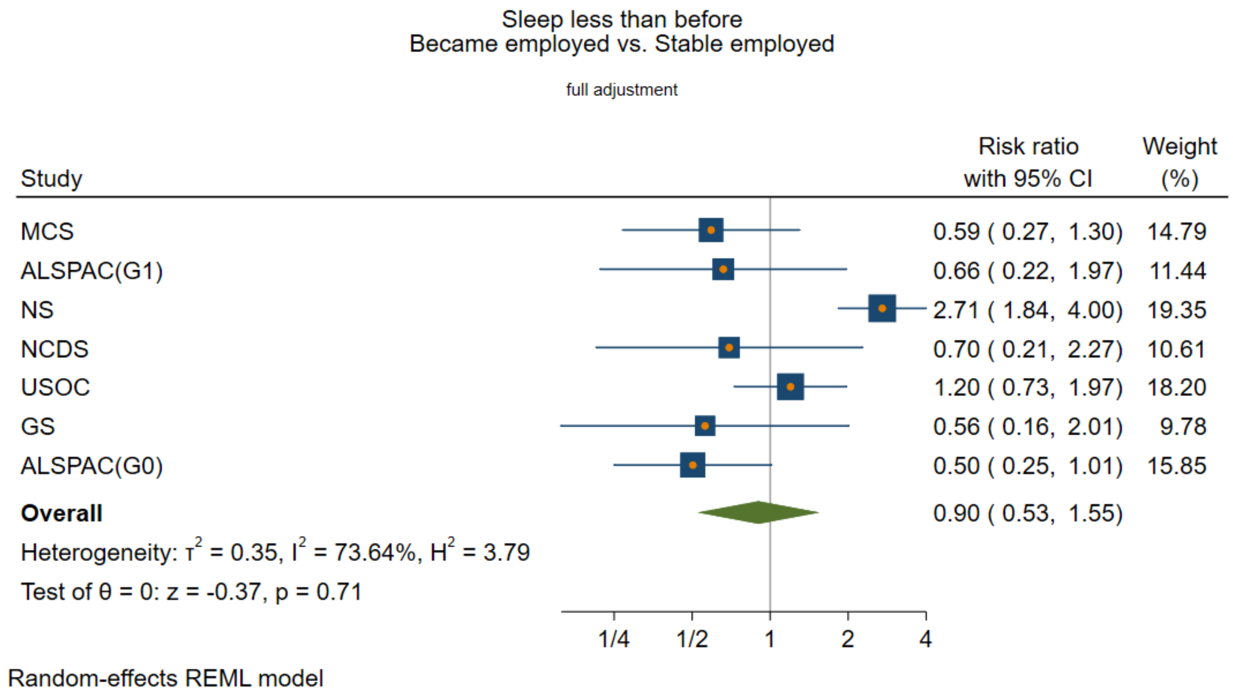
Sleep less than before
Became employed vs. Stable employed
full adjustment
Study
Risk ratio with 95% CI
Weight (%)
MCS 0.59 (0.27, 1.30) 14.79
ALSPAC(G1) 0.66 (0.22, 1.97) 11.44
NS 2.71 (1.84, 4.00) 19.35
NCDS 0.70 (0.21, 2.27) 10.61
USOC 1.20 (0.73, 1.97) 18.20
GS 0.56 (0.16, 2.01) 9.78
ALSPAC(G0) 0.50 (0.25, 1.01) 15.85
Overall 0.90 (0.53, 1.55)
Heterogeneity: τ² = 0.35, I² = 73.64%, H² = 3.79
Test of θ = 0: z = -0.37, p = 0.71
1/4 1/2 1 2 4
Random-effects REML model

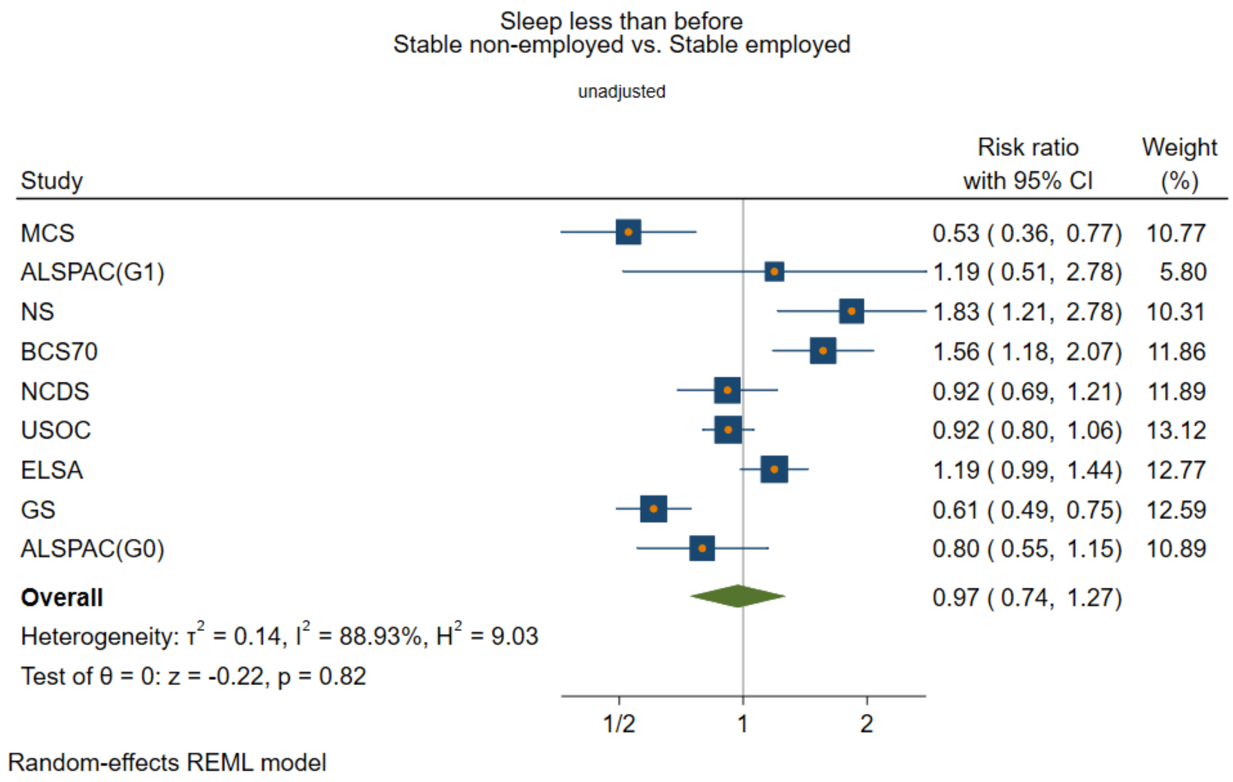

Sleep less than before
Stable non-employed vs. Stable employed
unadjusted
Study
Risk ratio with 95% CI
Weight (%)
MCS 0.53 (0.36, 0.77) 10.77
ALSPAC(G1) 1.19 (0.51, 2.78) 5.80
NS 1.83 (1.21, 2.78) 10.31
BCS70 1.56 (1.18, 2.07) 11.86
NCDS 0.92 (0.69, 1.21) 11.89
USOC 0.92 (0.80, 1.06) 13.12
ELSA 1.19 (0.99, 1.44) 12.77
GS 0.61 (0.49, 0.75) 12.59
ALSPAC(G0) 0.80 (0.55, 1.15) 10.89
Overall 0.97 (0.74, 1.27)
Heterogeneity: τ^2 = 0.14, I^2 = 88.93%, H^2 = 9.03
Test of θ = 0: z = -0.22, p = 0.82
1/2
1
2
Random-effects REML model

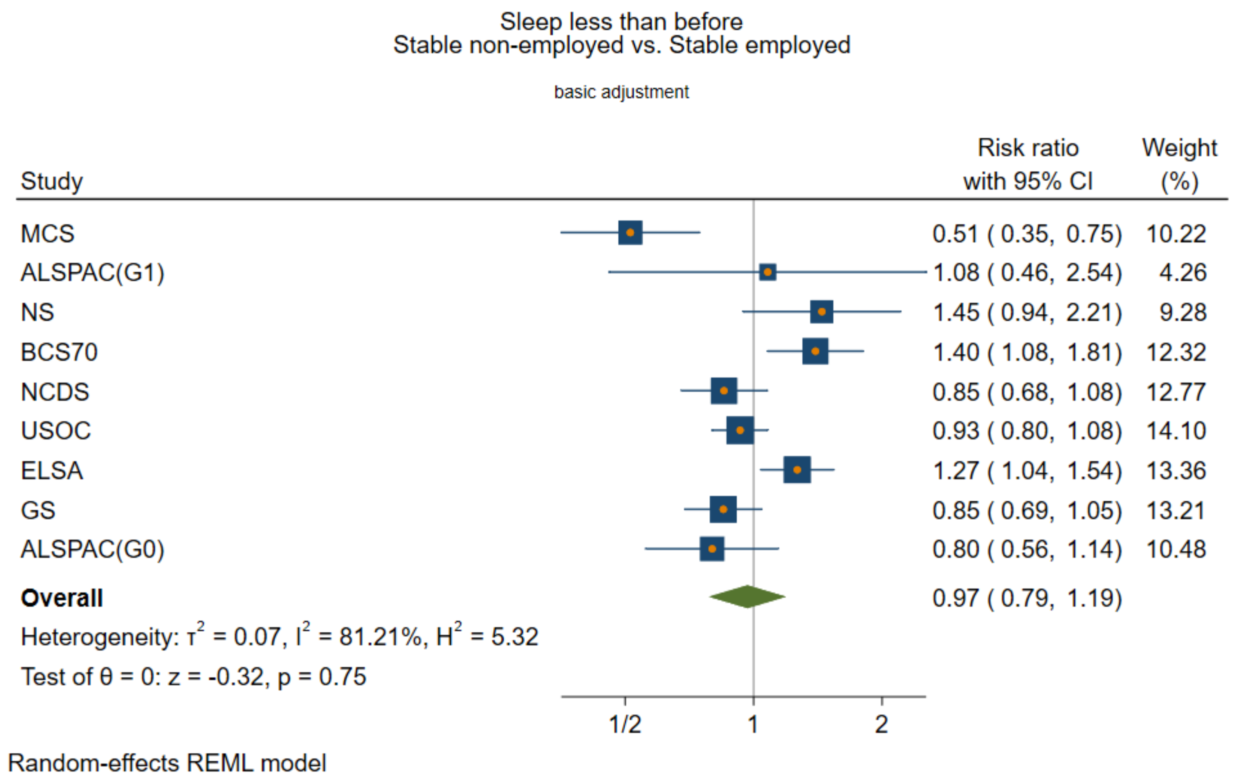

Sleep less than before
Stable non-employed vs. Stable employed
basic adjustment
Study
Risk ratio with 95% CI
Weight (%)
MCS 0.51 (0.35, 0.75) 10.22
ALSPAC(G1) 1.08 (0.46, 2.54) 4.26
NS 1.45 (0.94, 2.21) 9.28
BCS70 1.40 (1.08, 1.81) 12.32
NCDS 0.85 (0.68, 1.08) 12.77
USOC 0.93 (0.80, 1.08) 14.10
ELSA 1.27 (1.04, 1.54) 13.36
GS 0.85 (0.69, 1.05) 13.21
ALSPAC(G0) 0.80 (0.56, 1.14) 10.48
Overall 0.97 (0.79, 1.19)
Heterogeneity: τ^2 = 0.07, I^2 = 81.21%, H^2 = 5.32
Test of θ = 0: z = -0.32, p = 0.75
1/2
1
2
Random-effects REML model

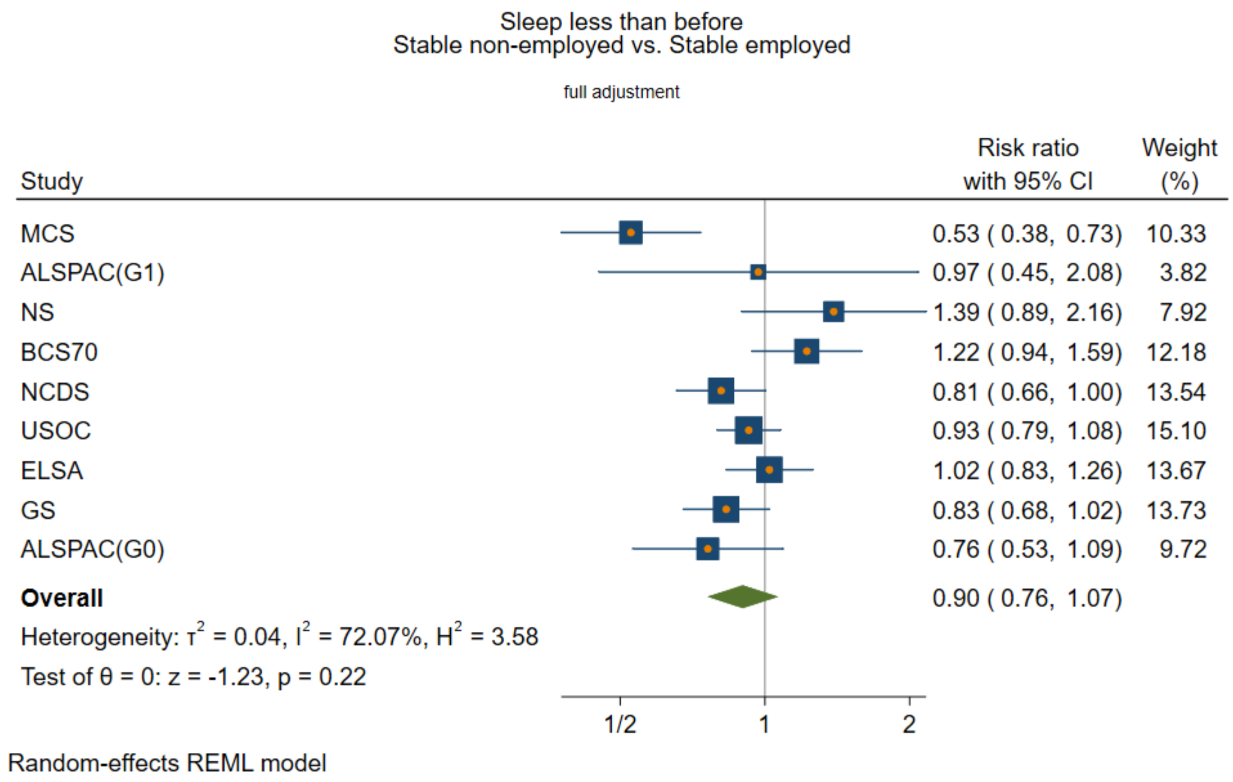

Sleep less than before
Stable non-employed vs. Stable employed
full adjustment
Study
Risk ratio with 95% CI
Weight (%)
MCS 0.53 (0.38, 0.73) 10.33
ALSPAC(G1) 0.97 (0.45, 2.08) 3.82
NS 1.39 (0.89, 2.16) 7.92
BCS70 1.22 (0.94, 1.59) 12.18
NCDS 0.81 (0.66, 1.00) 13.54
USOC 0.93 (0.79, 1.08) 15.10
ELSA 1.02 (0.83, 1.26) 13.67
GS 0.83 (0.68, 1.02) 13.73
ALSPAC(G0) 0.76 (0.53, 1.09) 9.72
Overall 0.90 (0.76, 1.07)
Heterogeneity: τ^2 = 0.04, I^2 = 72.07%, H^2 = 3.58
Test of θ = 0: z = -1.23, p = 0.22
1/2
1
2
Random-effects REML model

Figure set 9: Sleeps more than before

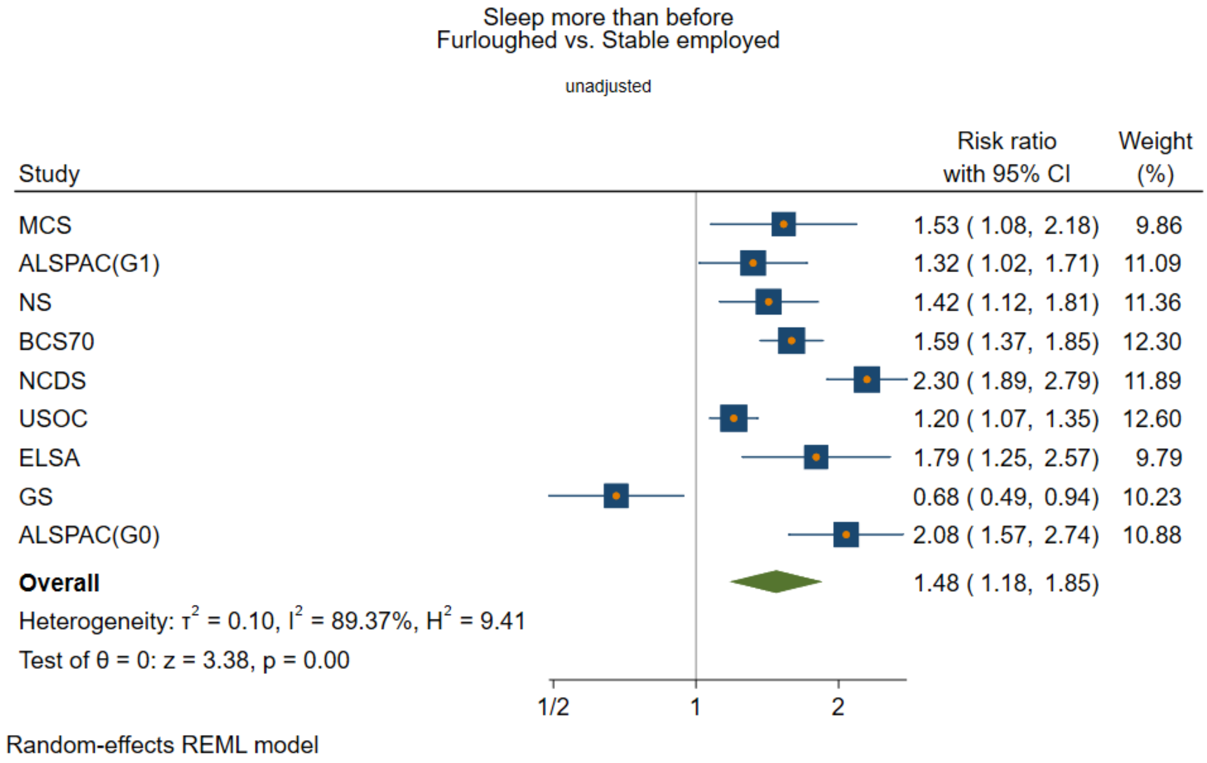

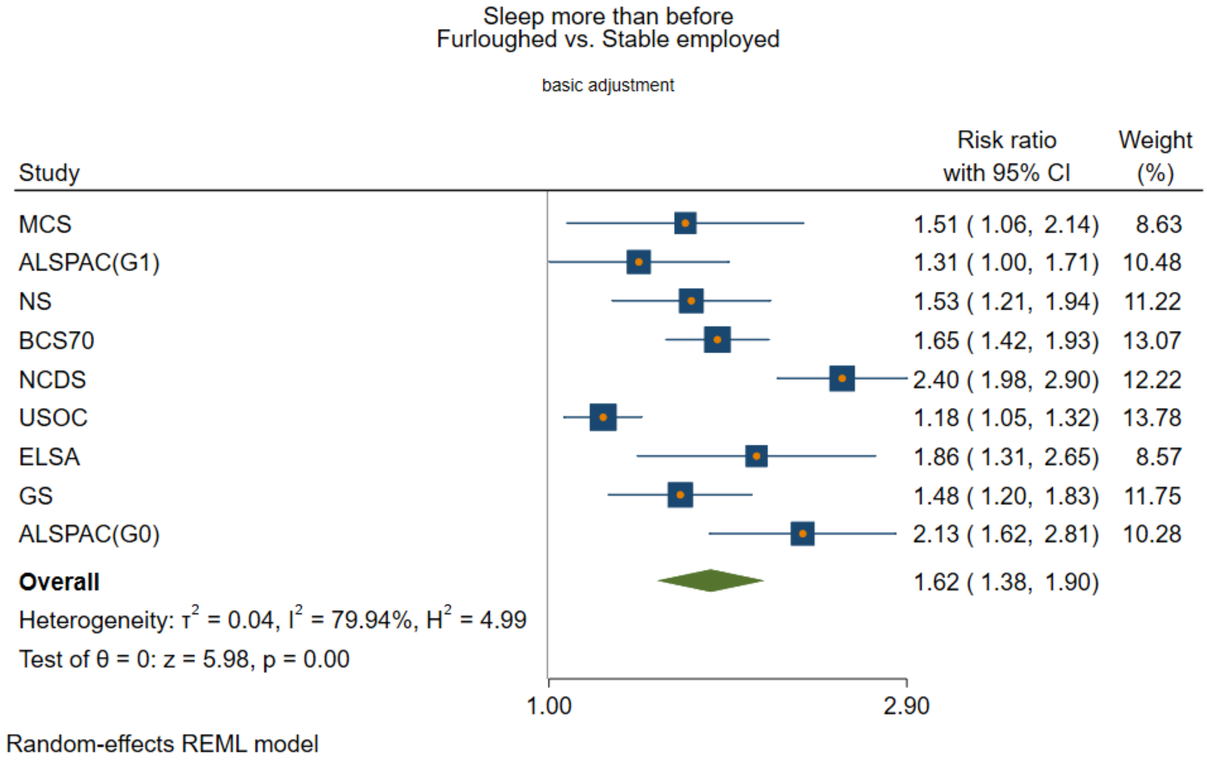

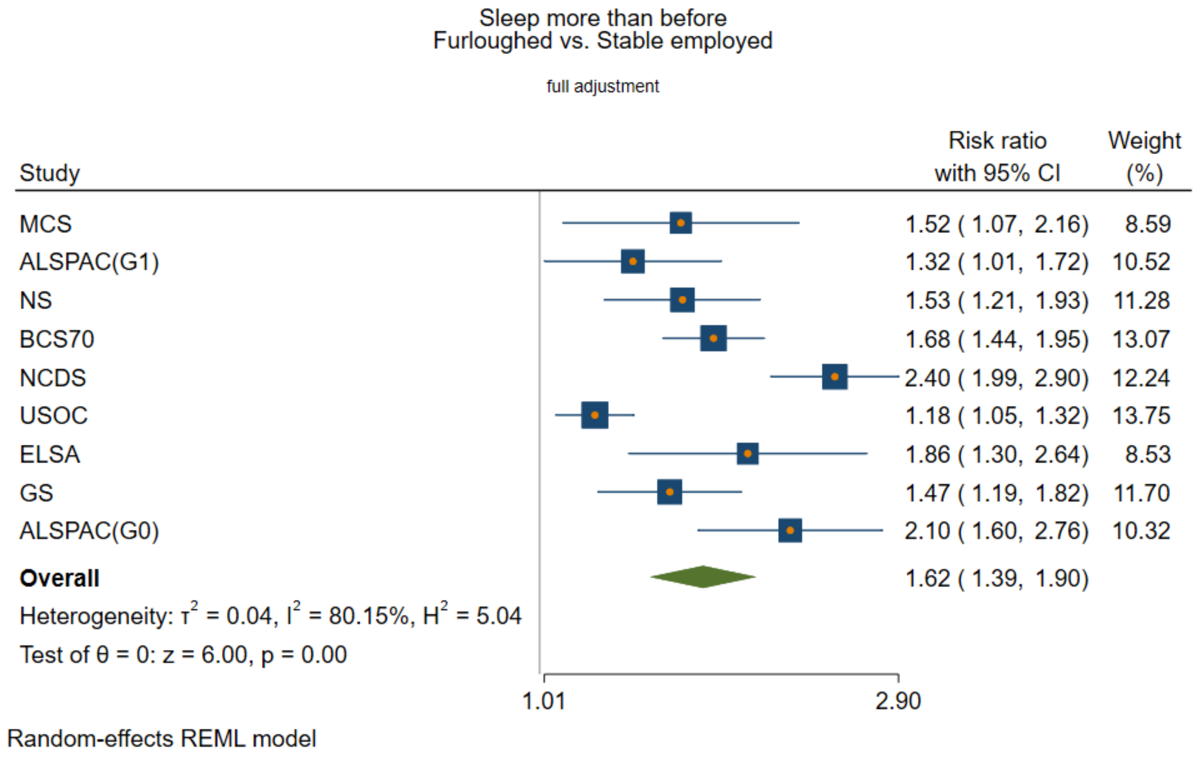

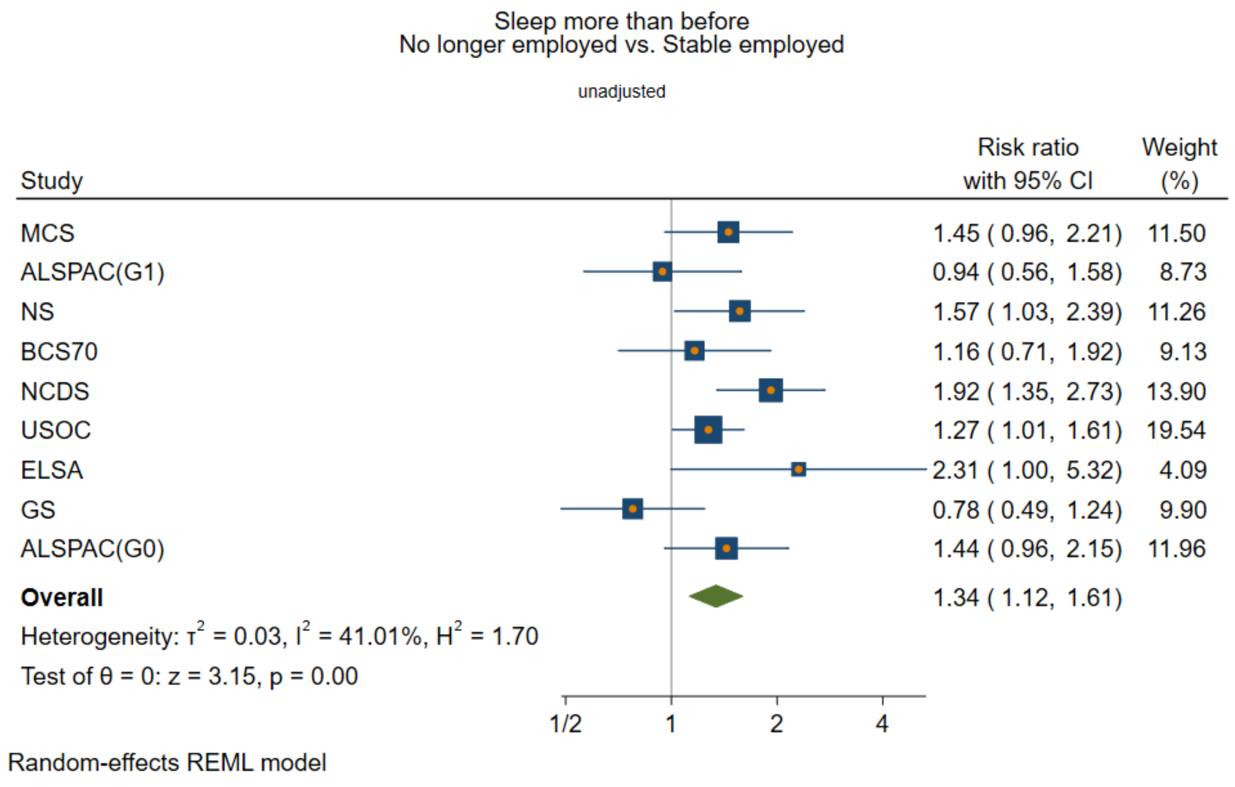
Sleep more than before
No longer employed vs. Stable employed
unadjusted
Study
Risk ratio with 95% CI
Weight (%)
MCS 1.45 (0.96, 2.21) 11.50
ALSPAC(G1) 0.94 (0.56, 1.58) 8.73
NS 1.57 (1.03, 2.39) 11.26
BCS70 1.16 (0.71, 1.92) 9.13
NCDS 1.92 (1.35, 2.73) 13.90
USOC 1.27 (1.01, 1.61) 19.54
ELSA 2.31 (1.00, 5.32) 4.09
GS 0.78 (0.49, 1.24) 9.90
ALSPAC(G0) 1.44 (0.96, 2.15) 11.96
Overall 1.34 (1.12, 1.61)
Heterogeneity: τ^2 = 0.03, I^2 = 41.01%, H^2 = 1.70
Test of θ = 0: z = 3.15, p = 0.00
1/2
1
2
4
Random-effects REML model

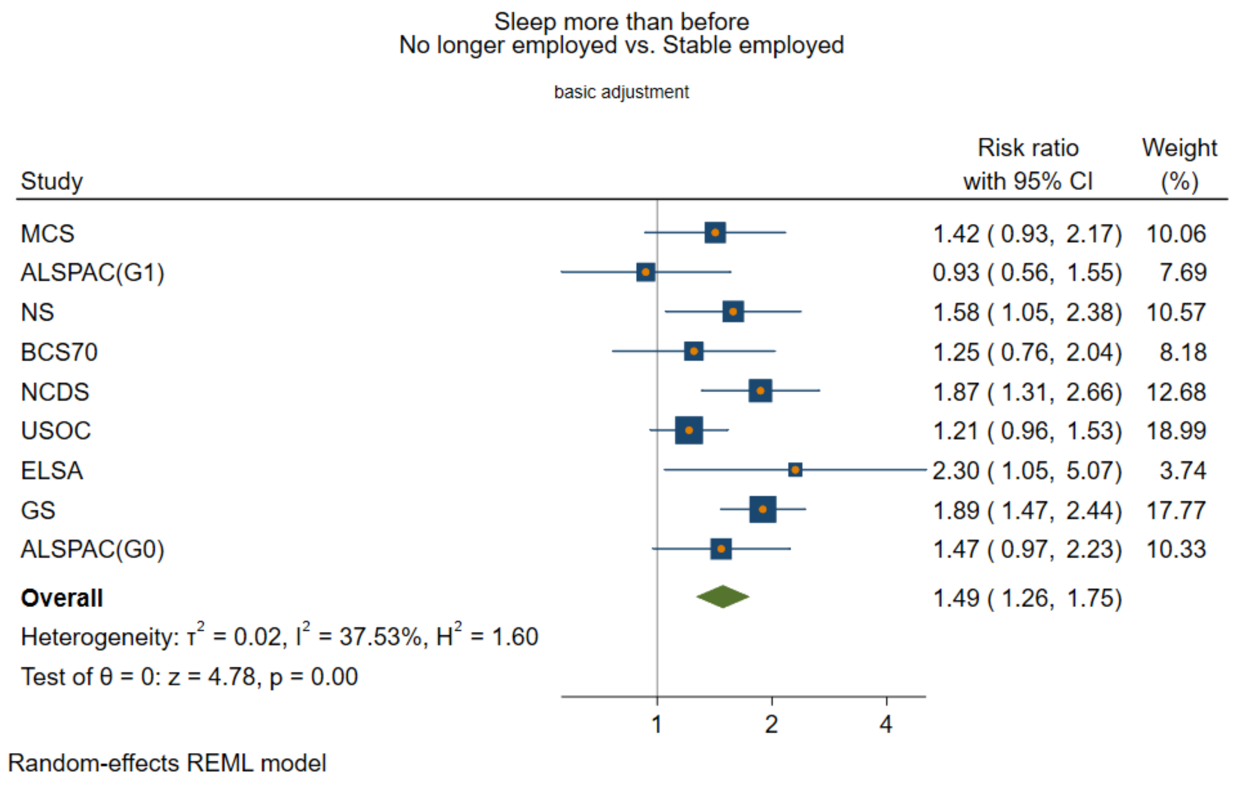
Sleep more than before
No longer employed vs. Stable employed
basic adjustment
Study
Risk ratio with 95% CI
Weight (%)
MCS 1.42 (0.93, 2.17) 10.06
ALSPAC(G1) 0.93 (0.56, 1.55) 7.69
NS 1.58 (1.05, 2.38) 10.57
BCS70 1.25 (0.76, 2.04) 8.18
NCDS 1.87 (1.31, 2.66) 12.68
USOC 1.21 (0.96, 1.53) 18.99
ELSA 2.30 (1.05, 5.07) 3.74
GS 1.89 (1.47, 2.44) 17.77
ALSPAC(G0) 1.47 (0.97, 2.23) 10.33
Overall 1.49 (1.26, 1.75)
Heterogeneity: τ^2 = 0.02, I^2 = 37.53%, H^2 = 1.60
Test of θ = 0: z = 4.78, p = 0.00
1
2
4
Random-effects REML model

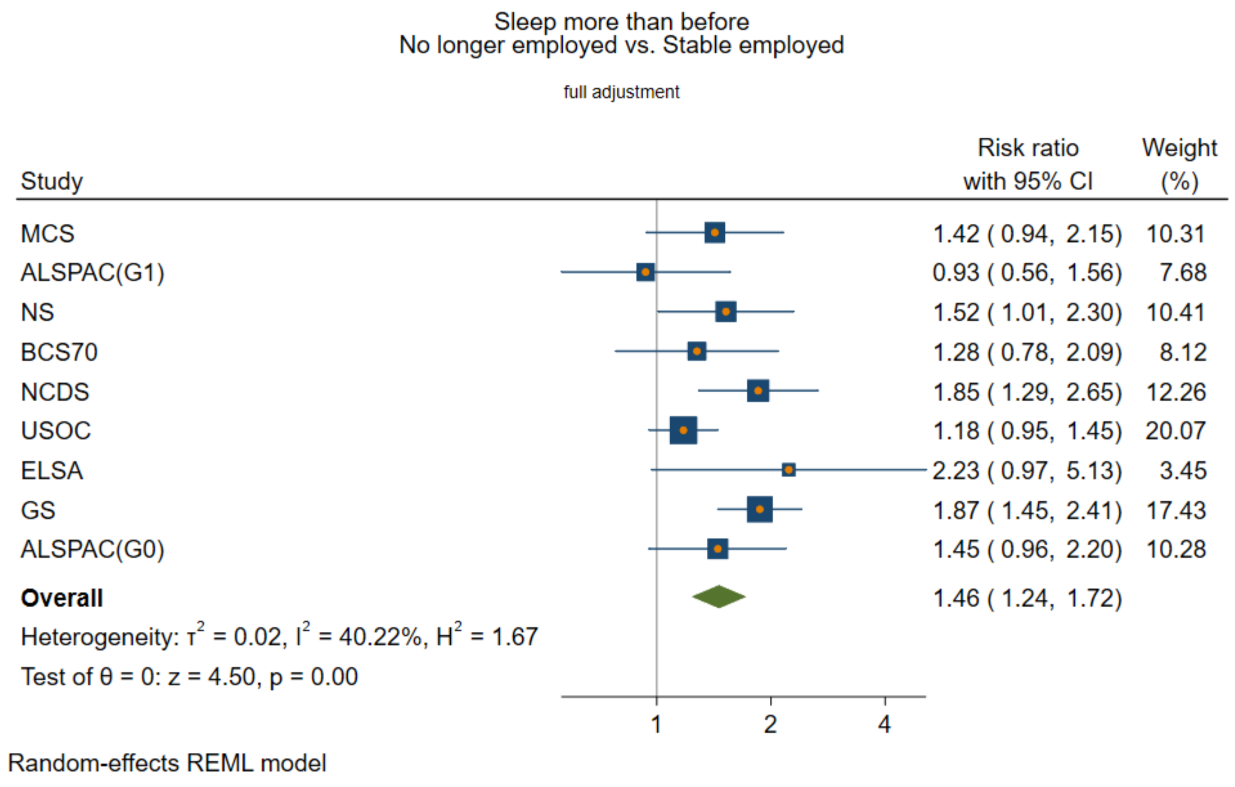
Sleep more than before
No longer employed vs. Stable employed
full adjustment
Study
Risk ratio with 95% CI
Weight (%)
MCS 1.42 (0.94, 2.15) 10.31
ALSPAC(G1) 0.93 (0.56, 1.56) 7.68
NS 1.52 (1.01, 2.30) 10.41
BCS70 1.28 (0.78, 2.09) 8.12
NCDS 1.85 (1.29, 2.65) 12.26
USOC 1.18 (0.95, 1.45) 20.07
ELSA 2.23 (0.97, 5.13) 3.45
GS 1.87 (1.45, 2.41) 17.43
ALSPAC(G0) 1.45 (0.96, 2.20) 10.28
Overall 1.46 (1.24, 1.72)
Heterogeneity: τ^2 = 0.02, I^2 = 40.22%, H^2 = 1.67
Test of θ = 0: z = 4.50, p = 0.00
1
2
4
Random-effects REML model

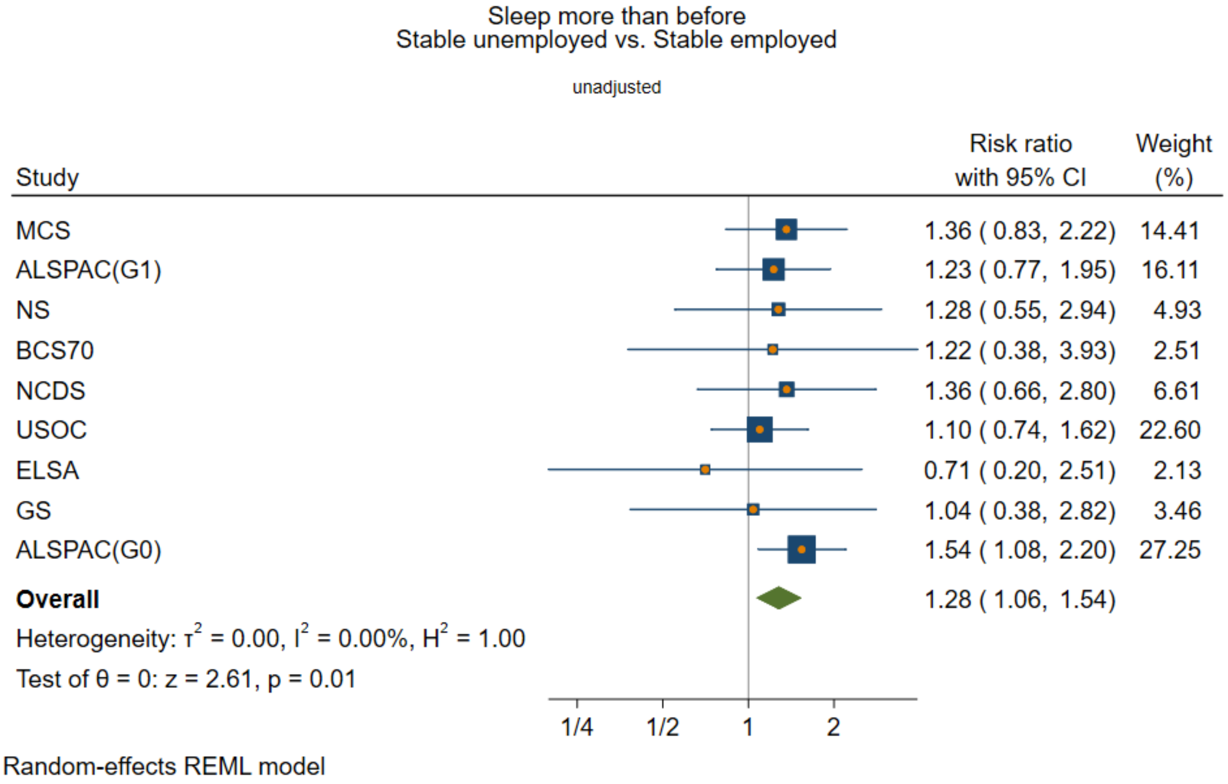
Sleep more than before
Stable unemployed vs. Stable employed
unadjusted
Study
Risk ratio with 95% CI
Weight (%)
MCS 1.36 (0.83, 2.22) 14.41
ALSPAC(G1) 1.23 (0.77, 1.95) 16.11
NS 1.28 (0.55, 2.94) 4.93
BCS70 1.22 (0.38, 3.93) 2.51
NCDS 1.36 (0.66, 2.80) 6.61
USOC 1.10 (0.74, 1.62) 22.60
ELSA 0.71 (0.20, 2.51) 2.13
GS 1.04 (0.38, 2.82) 3.46
ALSPAC(G0) 1.54 (1.08, 2.20) 27.25
Overall 1.28 (1.06, 1.54)
Heterogeneity: τ^2 = 0.00, I^2 = 0.00%, H^2 = 1.00
Test of θ = 0: z = 2.61, p = 0.01
1/4
1/2
1
2
Random-effects REML model

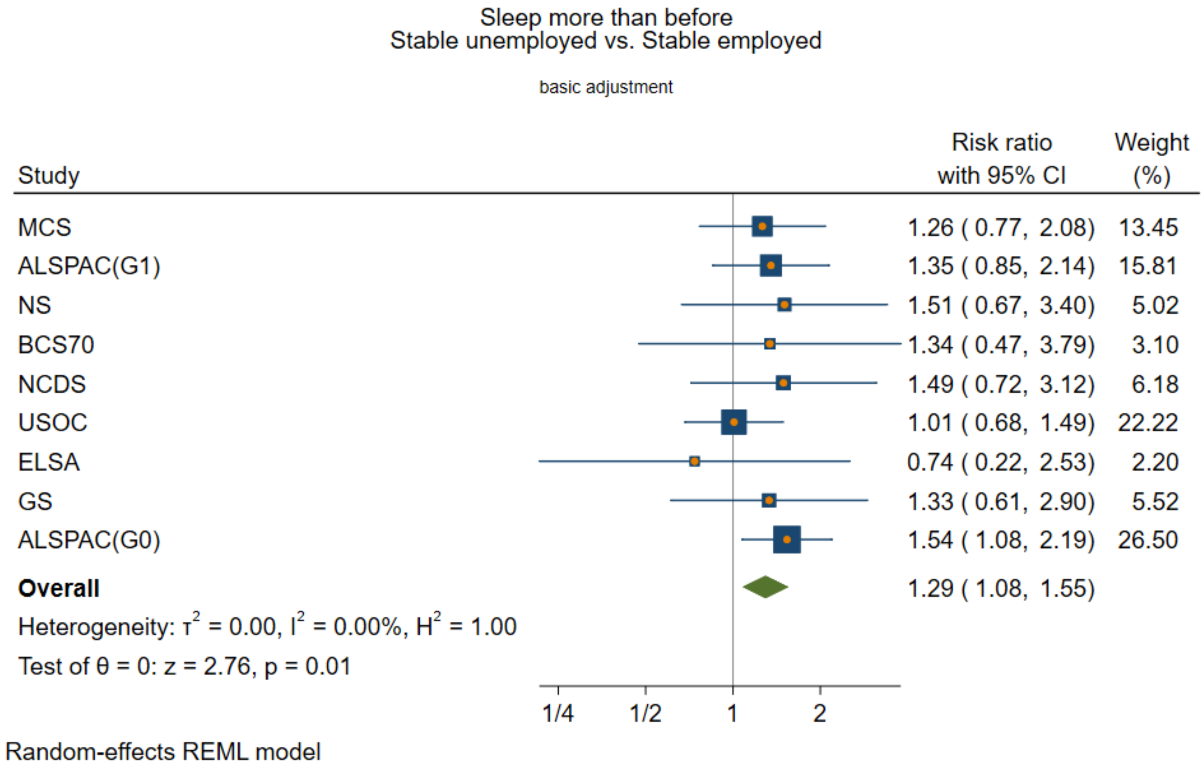
Sleep more than before
Stable unemployed vs. Stable employed
basic adjustment
Study
Risk ratio with 95% CI
Weight (%)
MCS 1.26 (0.77, 2.08) 13.45
ALSPAC(G1) 1.35 (0.85, 2.14) 15.81
NS 1.51 (0.67, 3.40) 5.02
BCS70 1.34 (0.47, 3.79) 3.10
NCDS 1.49 (0.72, 3.12) 6.18
USOC 1.01 (0.68, 1.49) 22.22
ELSA 0.74 (0.22, 2.53) 2.20
GS 1.33 (0.61, 2.90) 5.52
ALSPAC(G0) 1.54 (1.08, 2.19) 26.50
Overall 1.29 (1.08, 1.55)
Heterogeneity: τ^2 = 0.00, I^2 = 0.00%, H^2 = 1.00
Test of θ = 0: z = 2.76, p = 0.01
1/4
1/2
1
2
Random-effects REML model

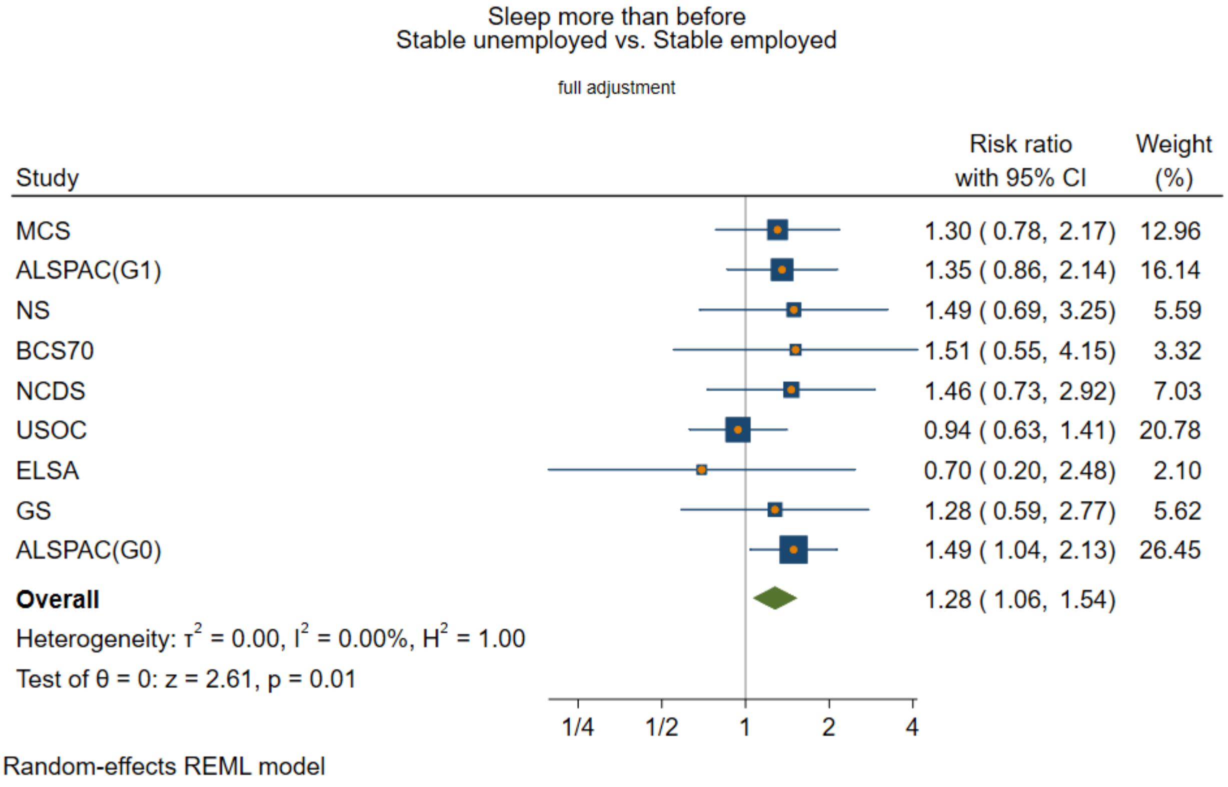
Sleep more than before
Stable unemployed vs. Stable employed
full adjustment
Study
Risk ratio with 95% CI
Weight (%)
MCS 1.30 (0.78, 2.17) 12.96
ALSPAC(G1) 1.35 (0.86, 2.14) 16.14
NS 1.49 (0.69, 3.25) 5.59
BCS70 1.51 (0.55, 4.15) 3.32
NCDS 1.46 (0.73, 2.92) 7.03
USOC 0.94 (0.63, 1.41) 20.78
ELSA 0.70 (0.20, 2.48) 2.10
GS 1.28 (0.59, 2.77) 5.62
ALSPAC(G0) 1.49 (1.04, 2.13) 26.45
Overall 1.28 (1.06, 1.54)
Heterogeneity: τ^2 = 0.00, I^2 = 0.00%, H^2 = 1.00
Test of θ = 0: z = 2.61, p = 0.01
1/4
1/2
1
2
4
Random-effects REML model

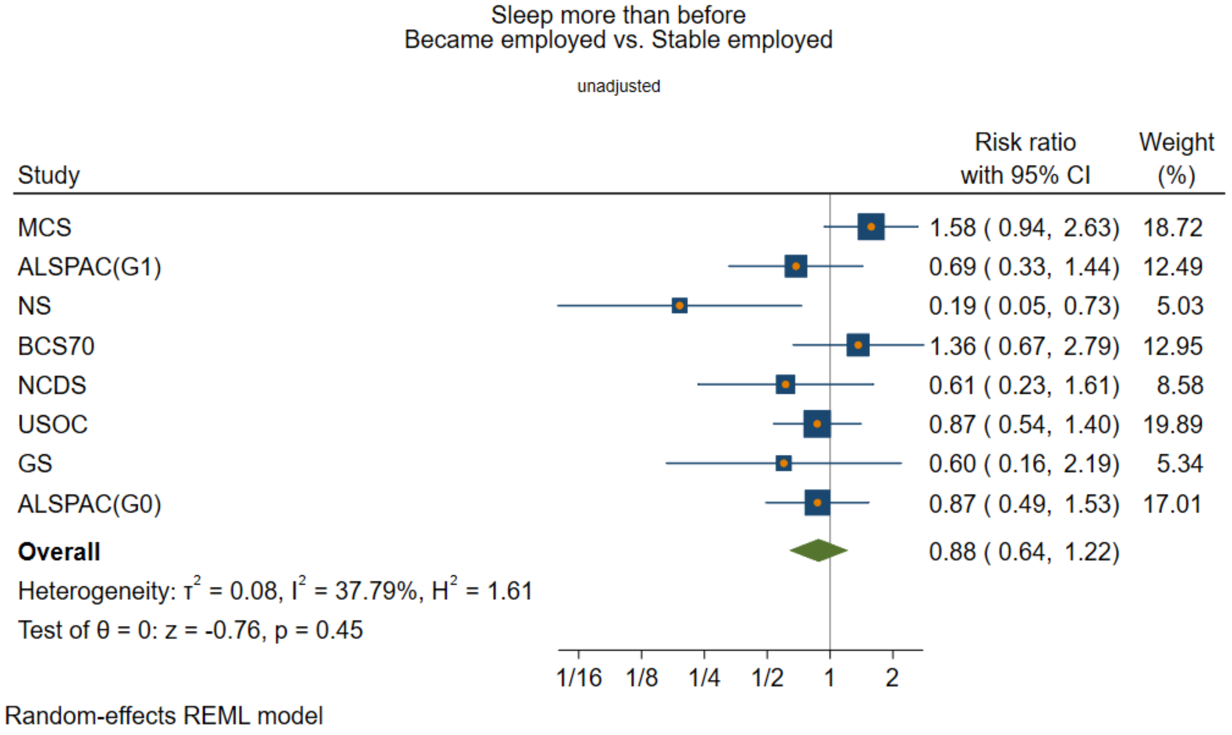
Sleep more than before
Became employed vs. Stable employed
unadjusted
Study
Risk ratio with 95% CI
Weight (%)
MCS 1.58 (0.94, 2.63) 18.72
ALSPAC(G1) 0.69 (0.33, 1.44) 12.49
NS 0.19 (0.05, 0.73) 5.03
BCS70 1.36 (0.67, 2.79) 12.95
NCDS 0.61 (0.23, 1.61) 8.58
USOC 0.87 (0.54, 1.40) 19.89
GS 0.60 (0.16, 2.19) 5.34
ALSPAC(G0) 0.87 (0.49, 1.53) 17.01
Overall 0.88 (0.64, 1.22)
Heterogeneity: τ² = 0.08, I² = 37.79%, H² = 1.61
Test of θ = 0: z = -0.76, p = 0.45
1/16 1/8 1/4 1/2 1 2
Random-effects REML model

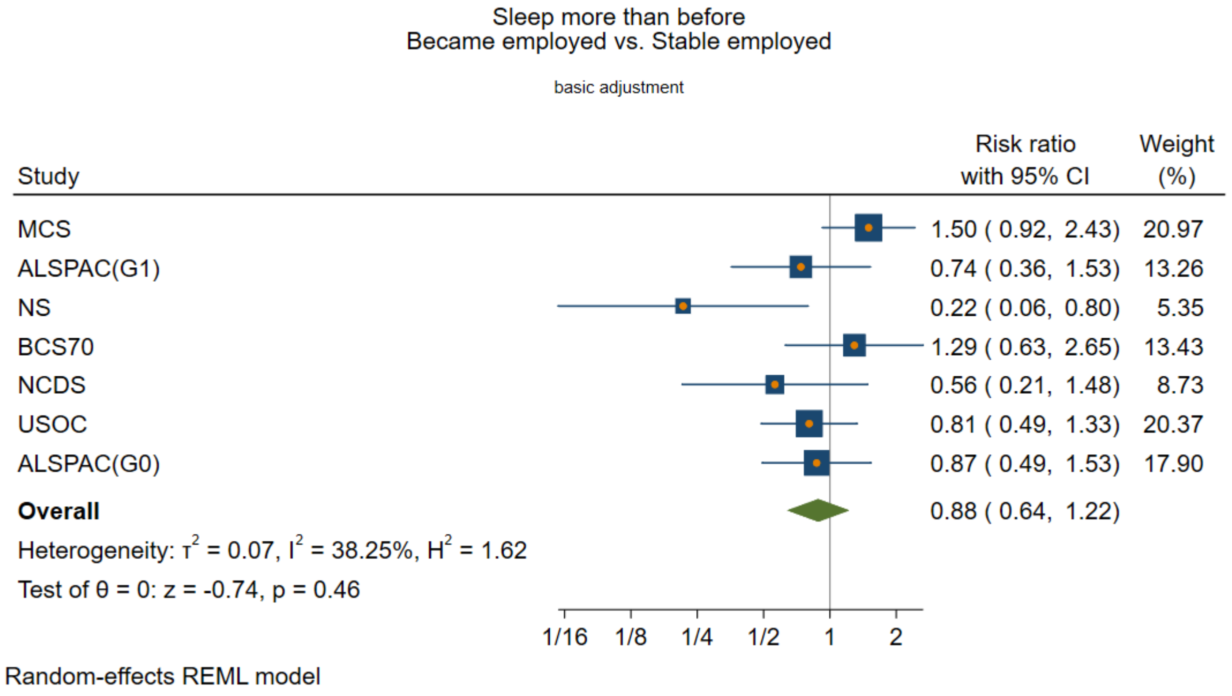
Sleep more than before
Became employed vs. Stable employed
basic adjustment
Study
Risk ratio with 95% CI
Weight (%)
MCS 1.50 (0.92, 2.43) 20.97
ALSPAC(G1) 0.74 (0.36, 1.53) 13.26
NS 0.22 (0.06, 0.80) 5.35
BCS70 1.29 (0.63, 2.65) 13.43
NCDS 0.56 (0.21, 1.48) 8.73
USOC 0.81 (0.49, 1.33) 20.37
ALSPAC(G0) 0.87 (0.49, 1.53) 17.90
Overall 0.88 (0.64, 1.22)
Heterogeneity: τ² = 0.07, I² = 38.25%, H² = 1.62
Test of θ = 0: z = -0.74, p = 0.46
1/16 1/8 1/4 1/2 1 2
Random-effects REML model

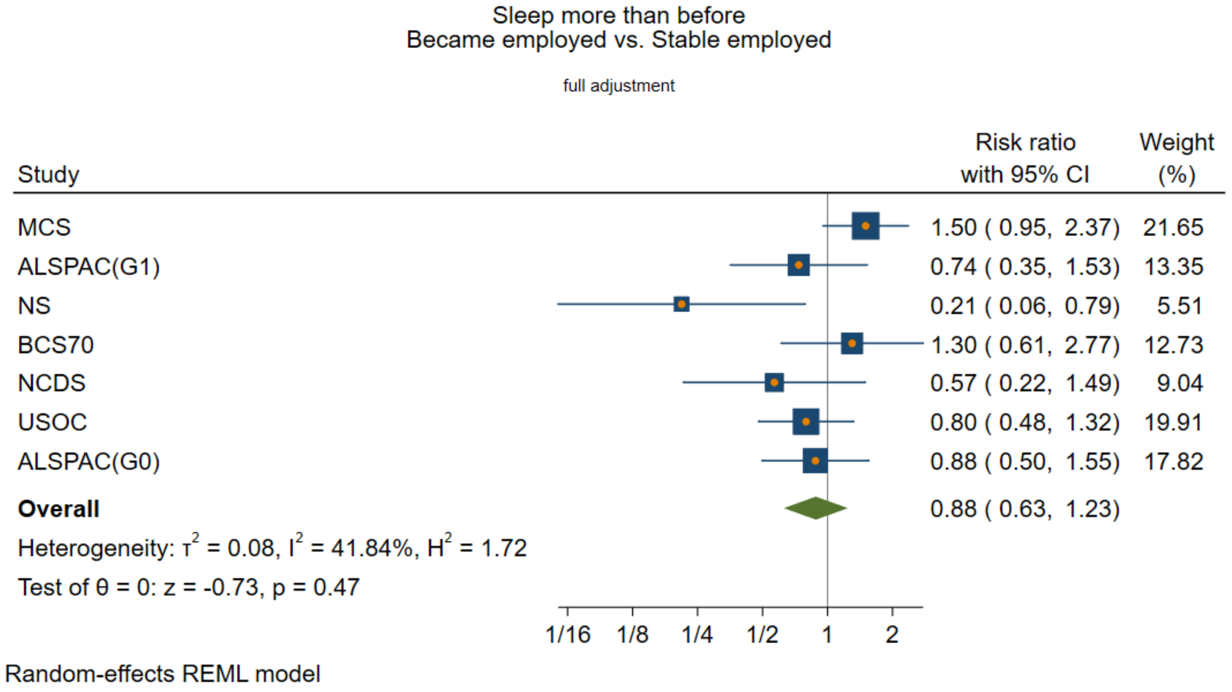
Sleep more than before
Became employed vs. Stable employed
full adjustment
Study
Risk ratio with 95% CI
Weight (%)
MCS 1.50 (0.95, 2.37) 21.65
ALSPAC(G1) 0.74 (0.35, 1.53) 13.35
NS 0.21 (0.06, 0.79) 5.51
BCS70 1.30 (0.61, 2.77) 12.73
NCDS 0.57 (0.22, 1.49) 9.04
USOC 0.80 (0.48, 1.32) 19.91
ALSPAC(G0) 0.88 (0.50, 1.55) 17.82
Overall 0.88 (0.63, 1.23)
Heterogeneity: τ² = 0.08, I² = 41.84%, H² = 1.72
Test of θ = 0: z = -0.73, p = 0.47
1/16 1/8 1/4 1/2 1 2
Random-effects REML model

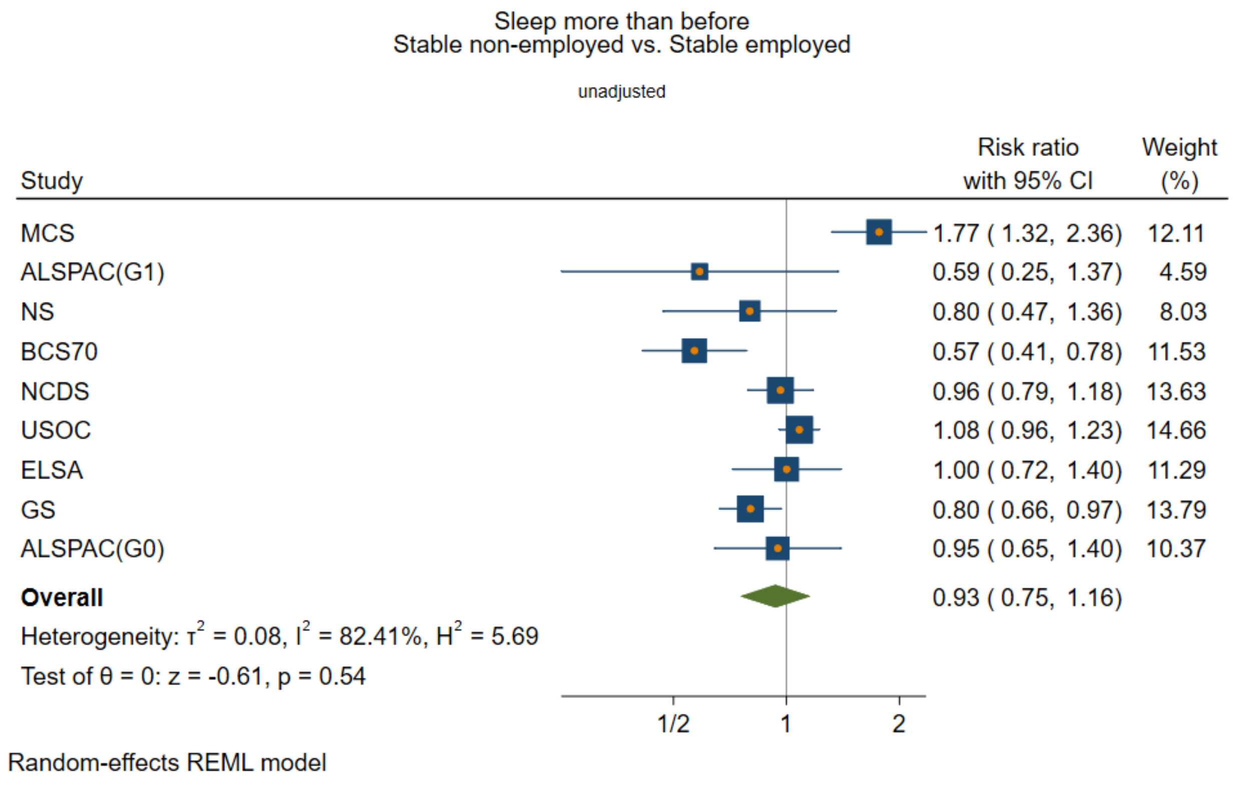
Sleep more than before
Stable non-employed vs. Stable employed
unadjusted
Study
Risk ratio with 95% CI
Weight (%)
MCS 1.77 (1.32, 2.36) 12.11
ALSPAC(G1) 0.59 (0.25, 1.37) 4.59
NS 0.80 (0.47, 1.36) 8.03
BCS70 0.57 (0.41, 0.78) 11.53
NCDS 0.96 (0.79, 1.18) 13.63
USOC 1.08 (0.96, 1.23) 14.66
ELSA 1.00 (0.72, 1.40) 11.29
GS 0.80 (0.66, 0.97) 13.79
ALSPAC(G0) 0.95 (0.65, 1.40) 10.37
Overall 0.93 (0.75, 1.16)
Heterogeneity: τ^2 = 0.08, I^2 = 82.41%, H^2 = 5.69
Test of θ = 0: z = -0.61, p = 0.54
1/2
1
2
Random-effects REML model

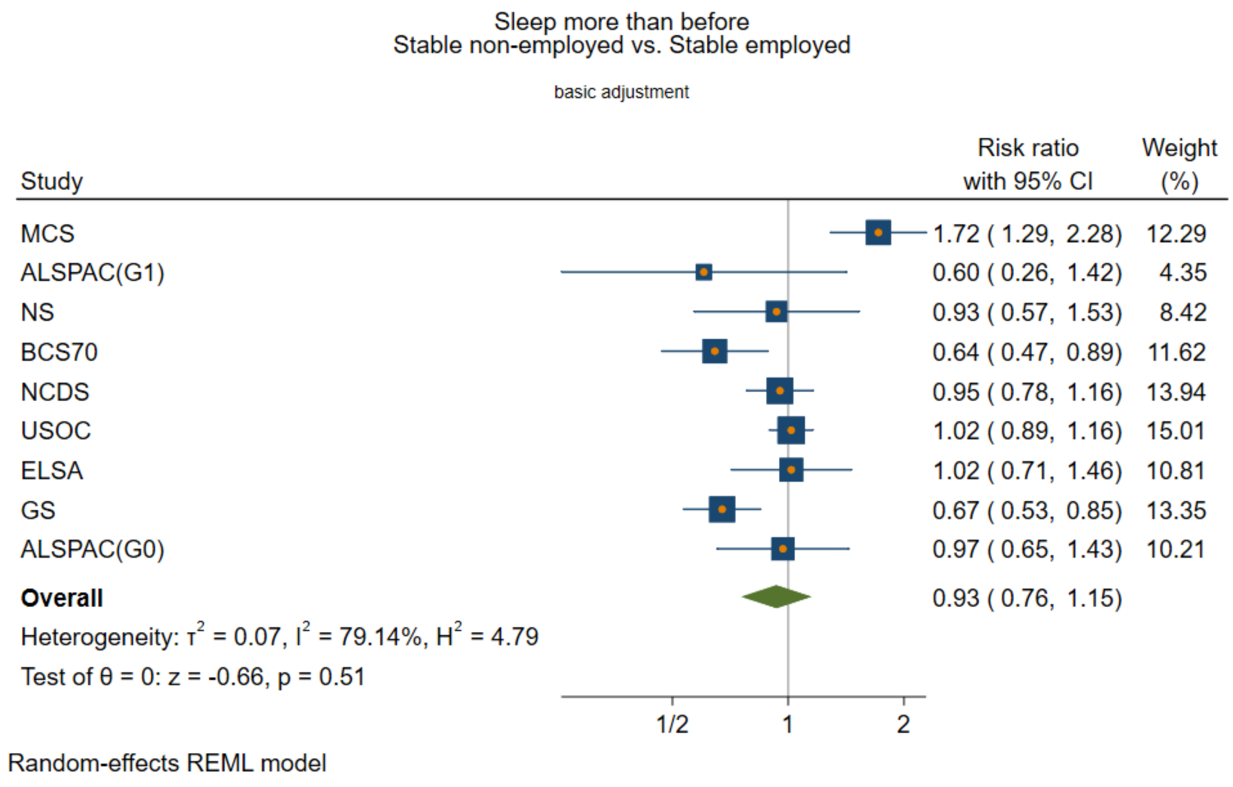
Sleep more than before
Stable non-employed vs. Stable employed
basic adjustment
Study
Risk ratio with 95% CI
Weight (%)
MCS 1.72 (1.29, 2.28) 12.29
ALSPAC(G1) 0.60 (0.26, 1.42) 4.35
NS 0.93 (0.57, 1.53) 8.42
BCS70 0.64 (0.47, 0.89) 11.62
NCDS 0.95 (0.78, 1.16) 13.94
USOC 1.02 (0.89, 1.16) 15.01
ELSA 1.02 (0.71, 1.46) 10.81
GS 0.67 (0.53, 0.85) 13.35
ALSPAC(G0) 0.97 (0.65, 1.43) 10.21
Overall 0.93 (0.76, 1.15)
Heterogeneity: τ^2 = 0.07, I^2 = 79.14%, H^2 = 4.79
Test of θ = 0: z = -0.66, p = 0.51
1/2
1
2
Random-effects REML model

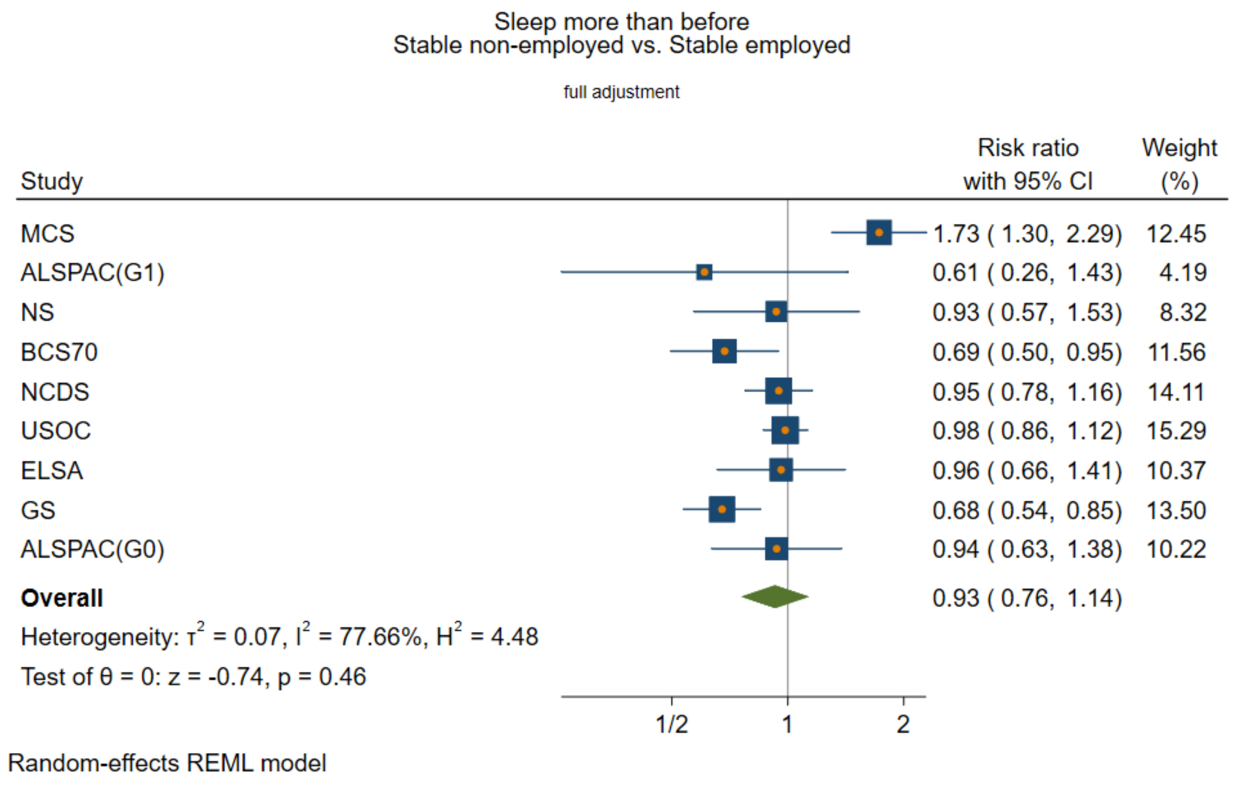
Sleep more than before
Stable non-employed vs. Stable employed
full adjustment
Study
Risk ratio with 95% CI
Weight (%)
MCS 1.73 (1.30, 2.29) 12.45
ALSPAC(G1) 0.61 (0.26, 1.43) 4.19
NS 0.93 (0.57, 1.53) 8.32
BCS70 0.69 (0.50, 0.95) 11.56
NCDS 0.95 (0.78, 1.16) 14.11
USOC 0.98 (0.86, 1.12) 15.29
ELSA 0.96 (0.66, 1.41) 10.37
GS 0.68 (0.54, 0.85) 13.50
ALSPAC(G0) 0.94 (0.63, 1.38) 10.22
Overall 0.93 (0.76, 1.14)
Heterogeneity: τ^2 = 0.07, I^2 = 77.66%, H^2 = 4.48
Test of θ = 0: z = -0.74, p = 0.46
1/2
1
2
Random-effects REML model

Figure set 10: From 6/9 hours a night to outside ‘normal range’

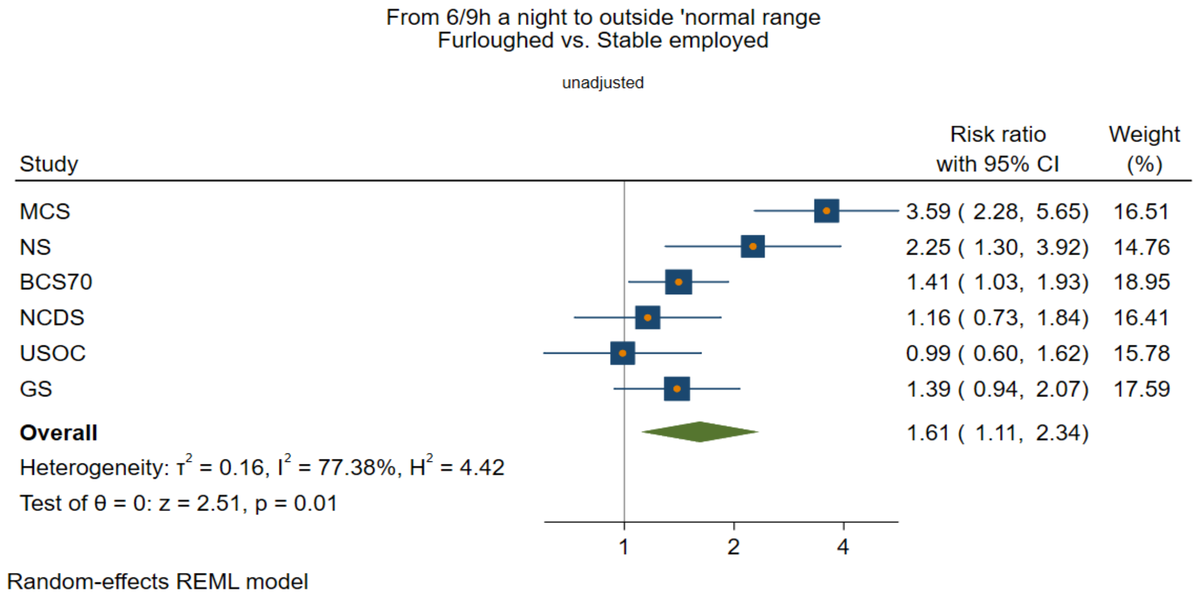

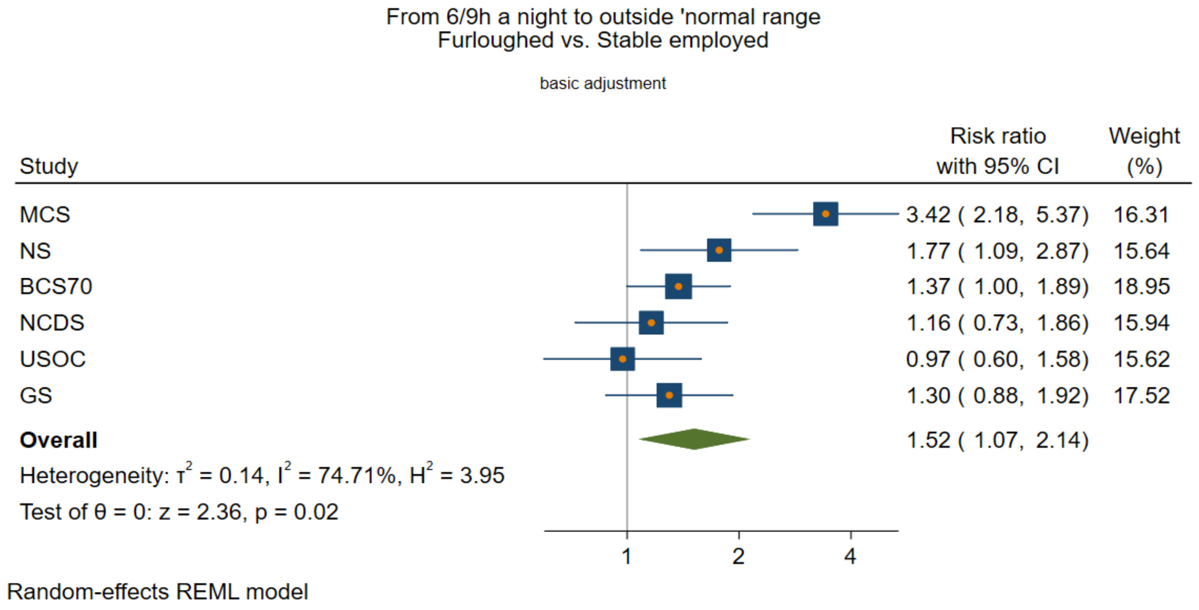

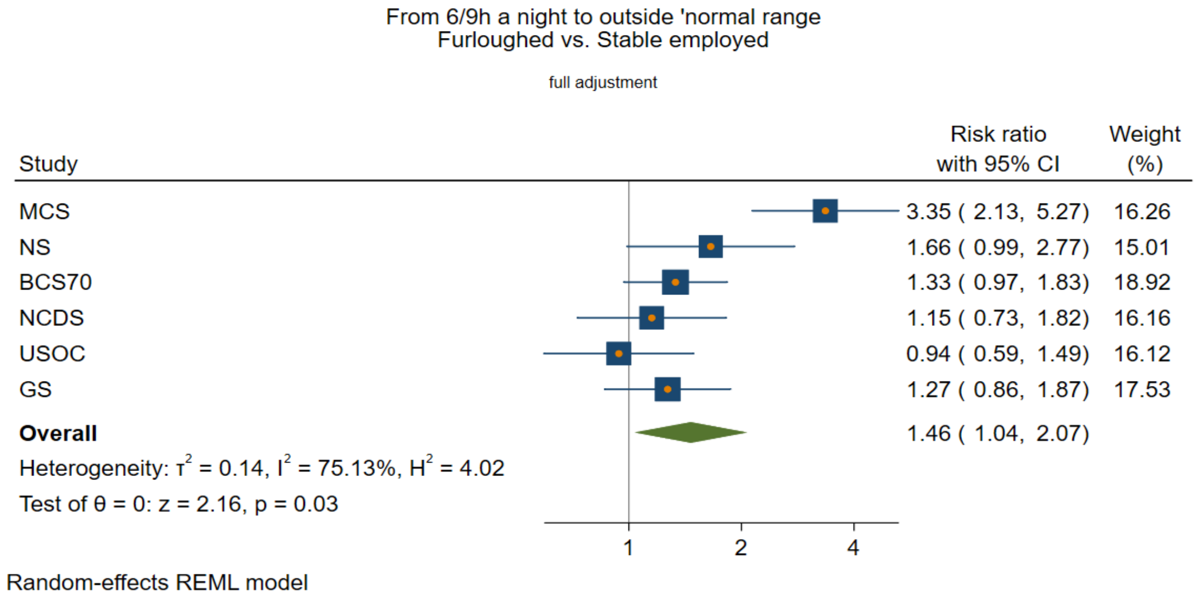

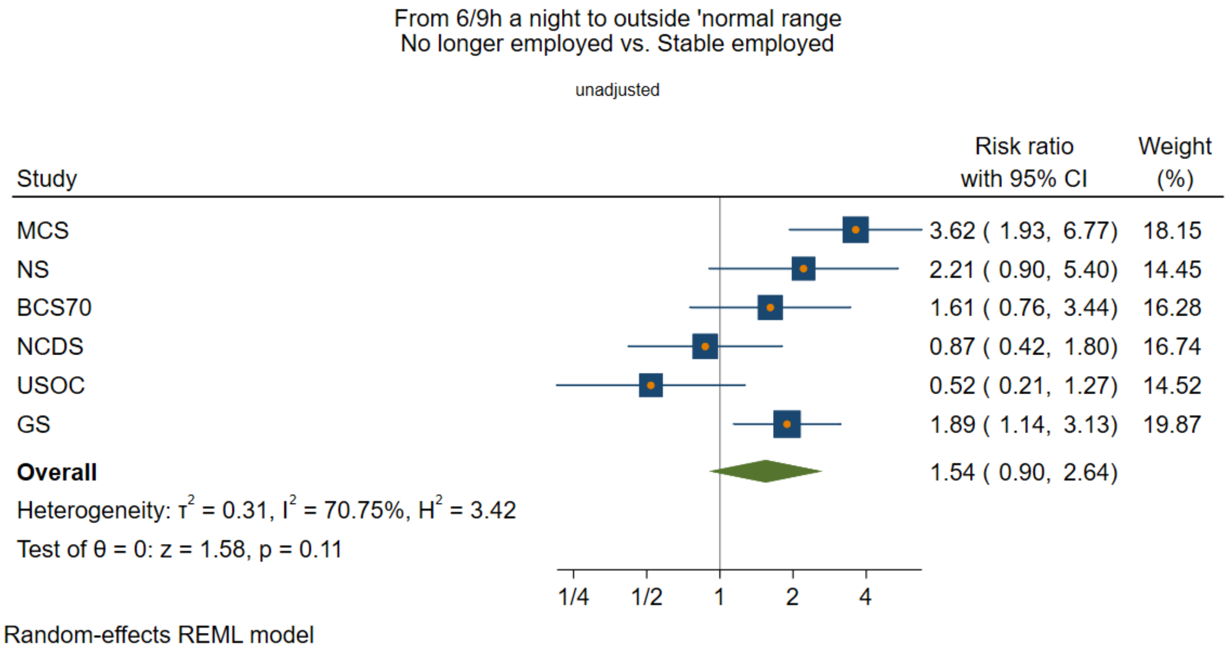
From 6/9h a night to outside 'normal range
No longer employed vs. Stable employed
unadjusted
Study
Risk ratio with 95% CI
Weight (%)
MCS
3.62 (1.93, 6.77)
18.15
NS
2.21 (0.90, 5.40)
14.45
BCS70
1.61 (0.76, 3.44)
16.28
NCDS
0.87 (0.42, 1.80)
16.74
USOC
0.52 (0.21, 1.27)
14.52
GS
1.89 (1.14, 3.13)
19.87
Overall
1.54 (0.90, 2.64)
Heterogeneity: τ² = 0.31, I² = 70.75%, H² = 3.42
Test of θ = 0: z = 1.58, p = 0.11
1/4
1/2
1
2
4
Random-effects REML model

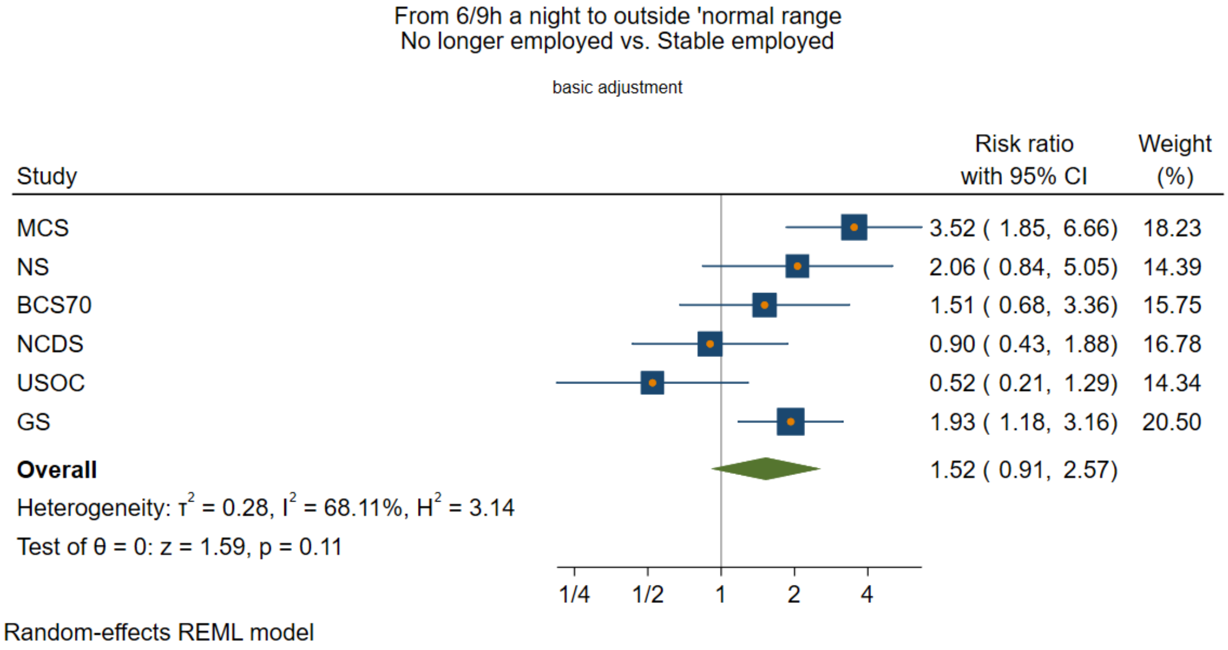
From 6/9h a night to outside 'normal range
No longer employed vs. Stable employed
basic adjustment
Study
Risk ratio with 95% CI
Weight (%)
MCS
3.52 (1.85, 6.66)
18.23
NS
2.06 (0.84, 5.05)
14.39
BCS70
1.51 (0.68, 3.36)
15.75
NCDS
0.90 (0.43, 1.88)
16.78
USOC
0.52 (0.21, 1.29)
14.34
GS
1.93 (1.18, 3.16)
20.50
Overall
1.52 (0.91, 2.57)
Heterogeneity: τ² = 0.28, I² = 68.11%, H² = 3.14
Test of θ = 0: z = 1.59, p = 0.11
1/4
1/2
1
2
4
Random-effects REML model

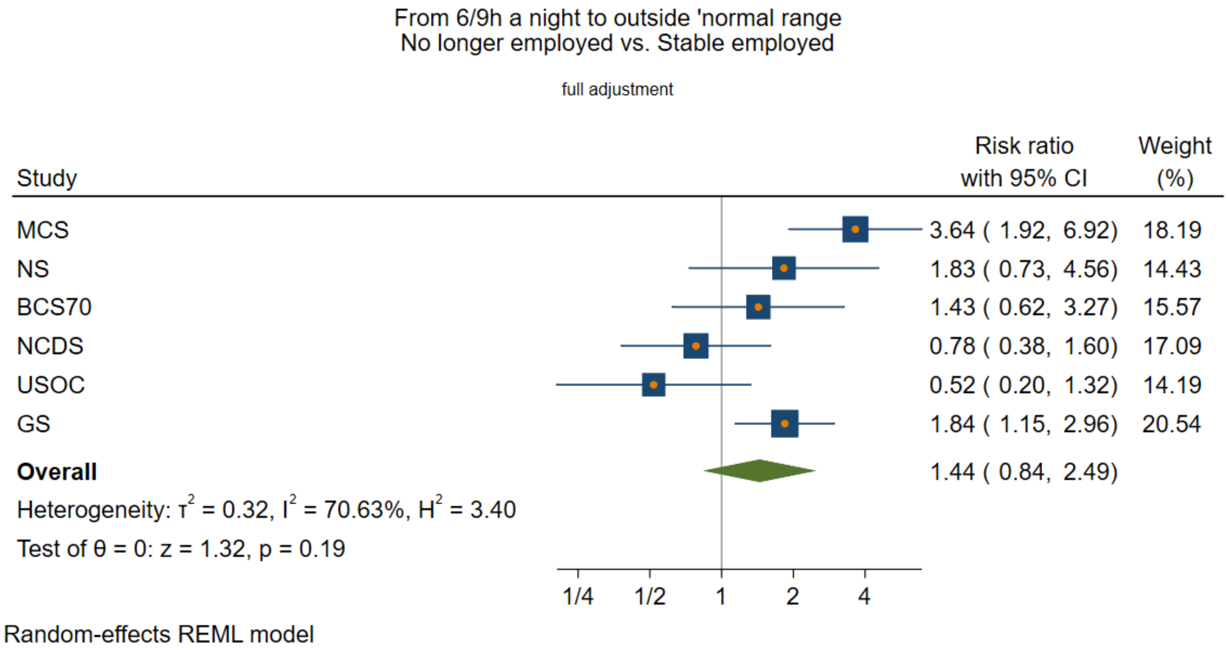
From 6/9h a night to outside 'normal range
No longer employed vs. Stable employed
full adjustment
Study
Risk ratio with 95% CI
Weight (%)
MCS
3.64 (1.92, 6.92)
18.19
NS
1.83 (0.73, 4.56)
14.43
BCS70
1.43 (0.62, 3.27)
15.57
NCDS
0.78 (0.38, 1.60)
17.09
USOC
0.52 (0.20, 1.32)
14.19
GS
1.84 (1.15, 2.96)
20.54
Overall
1.44 (0.84, 2.49)
Heterogeneity: τ² = 0.32, I² = 70.63%, H² = 3.40
Test of θ = 0: z = 1.32, p = 0.19
1/4
1/2
1
2
4
Random-effects REML model

From 6/9h a night to outside 'normal range
Stable unemployed vs. Stable employed

unadjusted

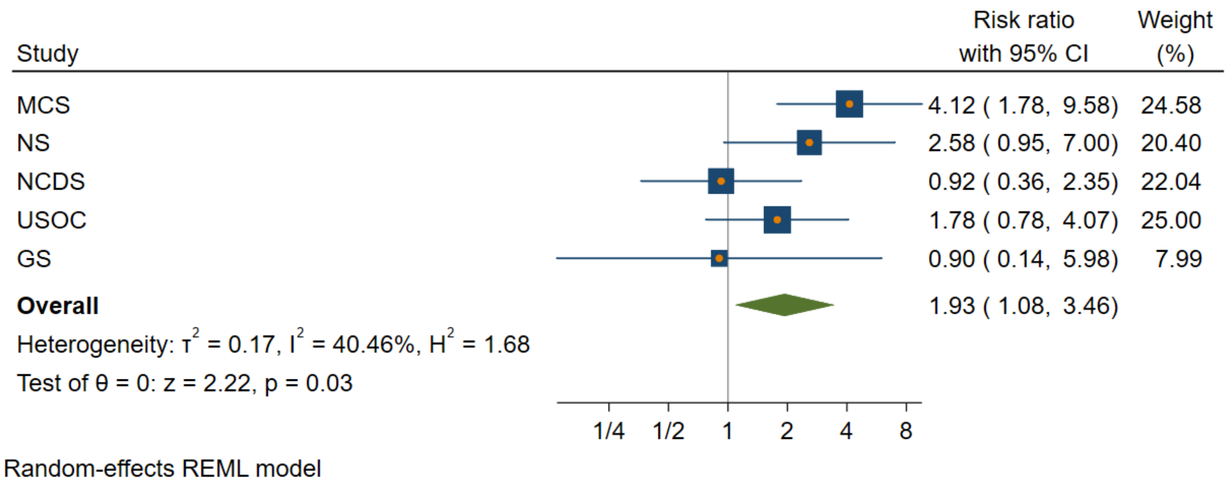

From 6/9h a night to outside 'normal range
Stable unemployed vs. Stable employed

basic adjustment

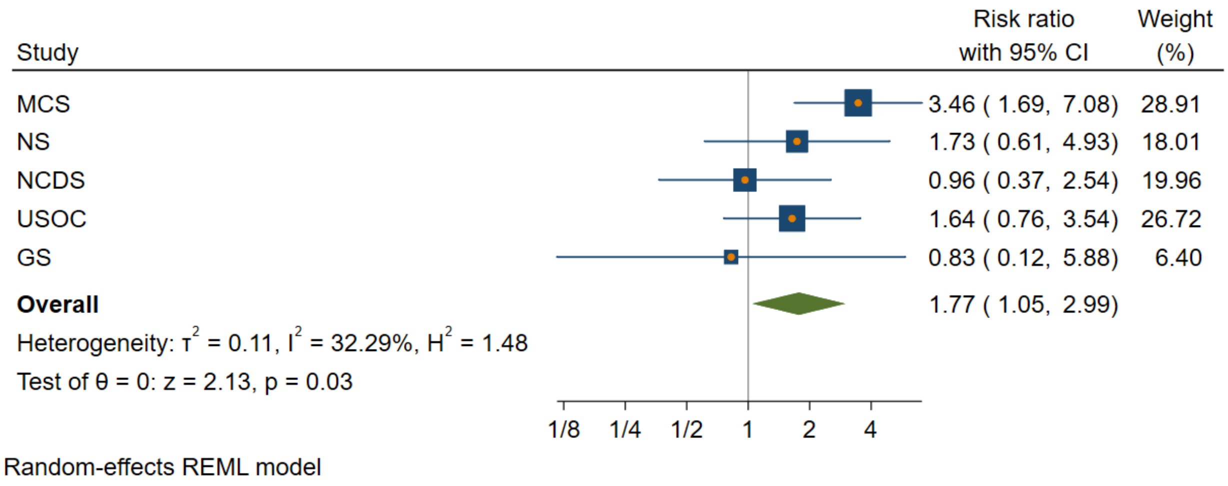

From 6/9h a night to outside 'normal range
Stable unemployed vs. Stable employed

full adjustment

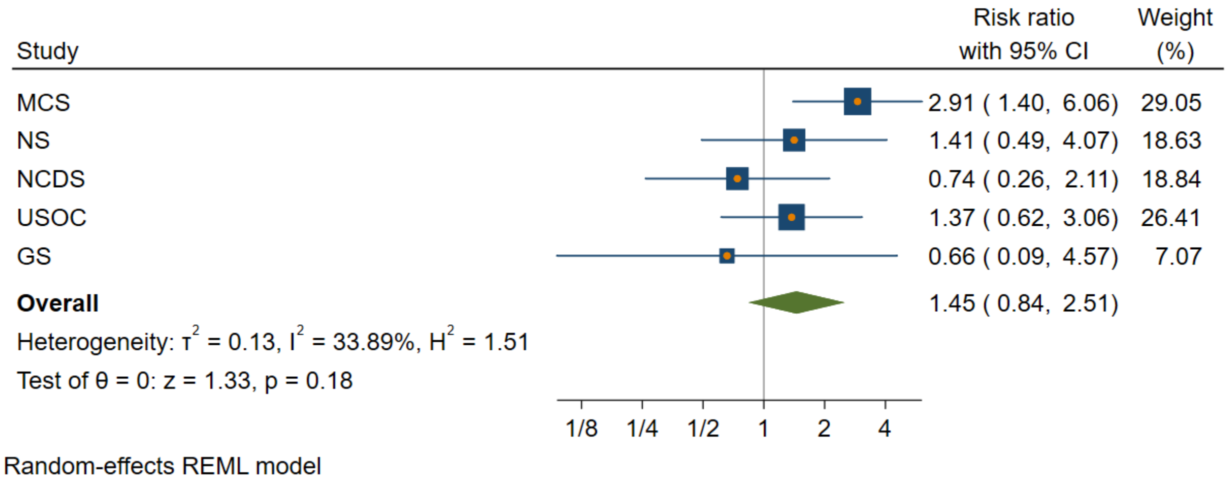

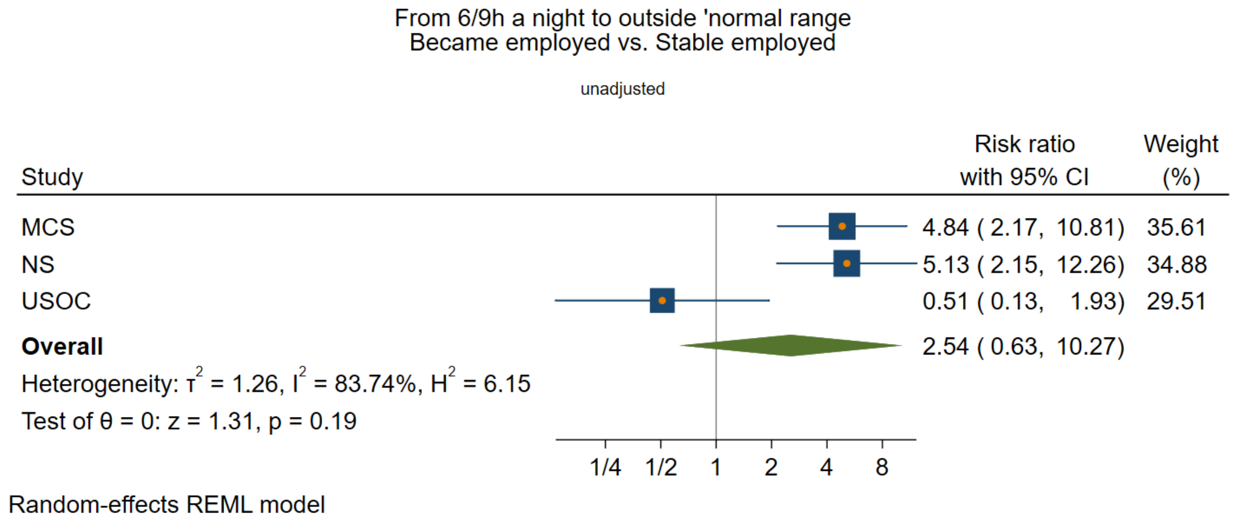
From 6/9h a night to outside 'normal range
Became employed vs. Stable employed
unadjusted
Study
Risk ratio with 95% CI
Weight (%)
MCS 4.84 (2.17, 10.81) 35.61
NS 5.13 (2.15, 12.26) 34.88
USOC 0.51 (0.13, 1.93) 29.51
Overall 2.54 (0.63, 10.27)
Heterogeneity: τ² = 1.26, I² = 83.74%, H² = 6.15
Test of θ = 0: z = 1.31, p = 0.19
1/4 1/2 1 2 4 8
Random-effects REML model

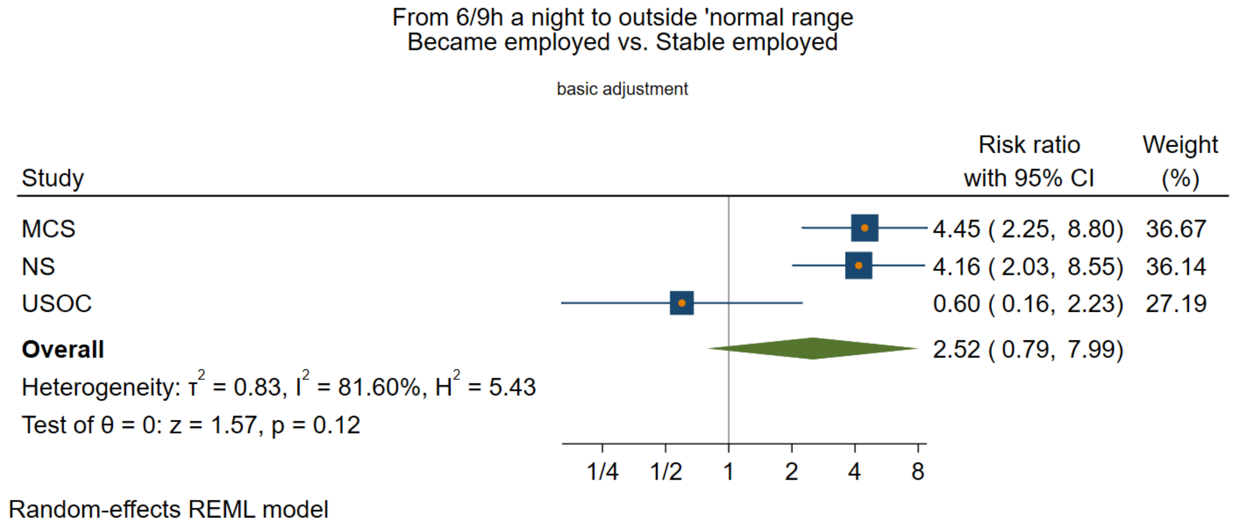
From 6/9h a night to outside 'normal range
Became employed vs. Stable employed
basic adjustment
Study
Risk ratio with 95% CI
Weight (%)
MCS 4.45 (2.25, 8.80) 36.67
NS 4.16 (2.03, 8.55) 36.14
USOC 0.60 (0.16, 2.23) 27.19
Overall 2.52 (0.79, 7.99)
Heterogeneity: τ² = 0.83, I² = 81.60%, H² = 5.43
Test of θ = 0: z = 1.57, p = 0.12
1/4 1/2 1 2 4 8
Random-effects REML model

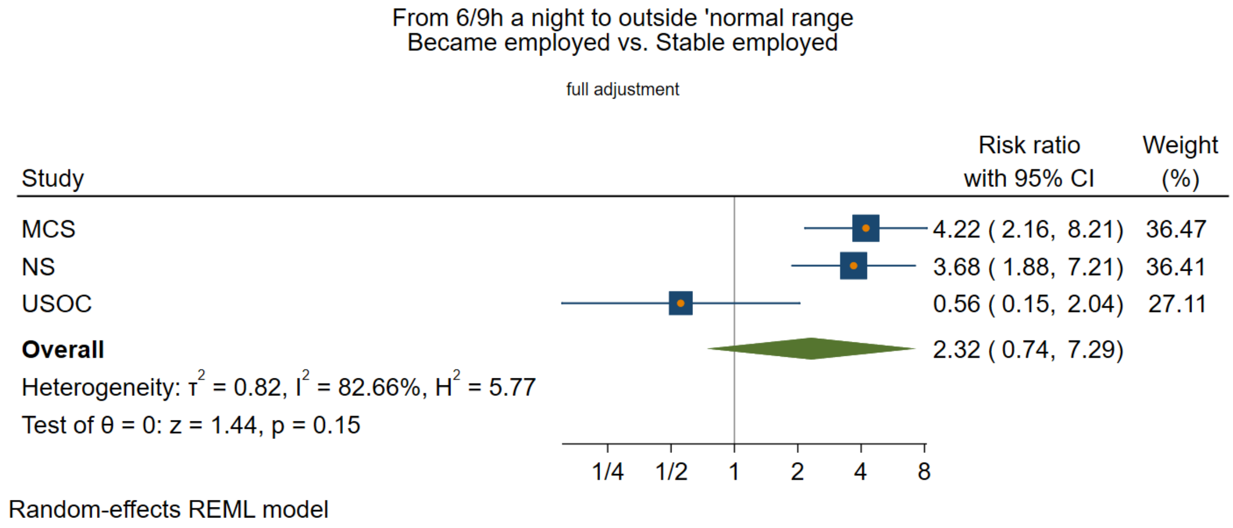
From 6/9h a night to outside 'normal range
Became employed vs. Stable employed
full adjustment
Study
Risk ratio with 95% CI
Weight (%)
MCS 4.22 (2.16, 8.21) 36.47
NS 3.68 (1.88, 7.21) 36.41
USOC 0.56 (0.15, 2.04) 27.11
Overall 2.32 (0.74, 7.29)
Heterogeneity: τ² = 0.82, I² = 82.66%, H² = 5.77
Test of θ = 0: z = 1.44, p = 0.15
1/4 1/2 1 2 4 8
Random-effects REML model

From 6/9h a night to outside 'normal range
Stable non-employed vs. Stable employed

unadjusted

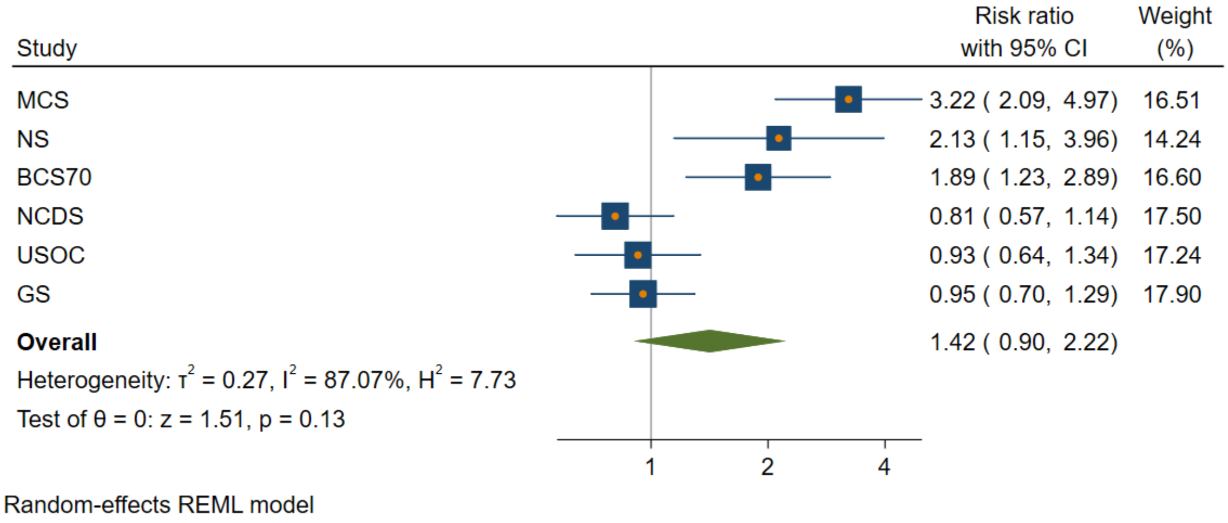

From 6/9h a night to outside 'normal range
Stable non-employed vs. Stable employed

basic adjustment

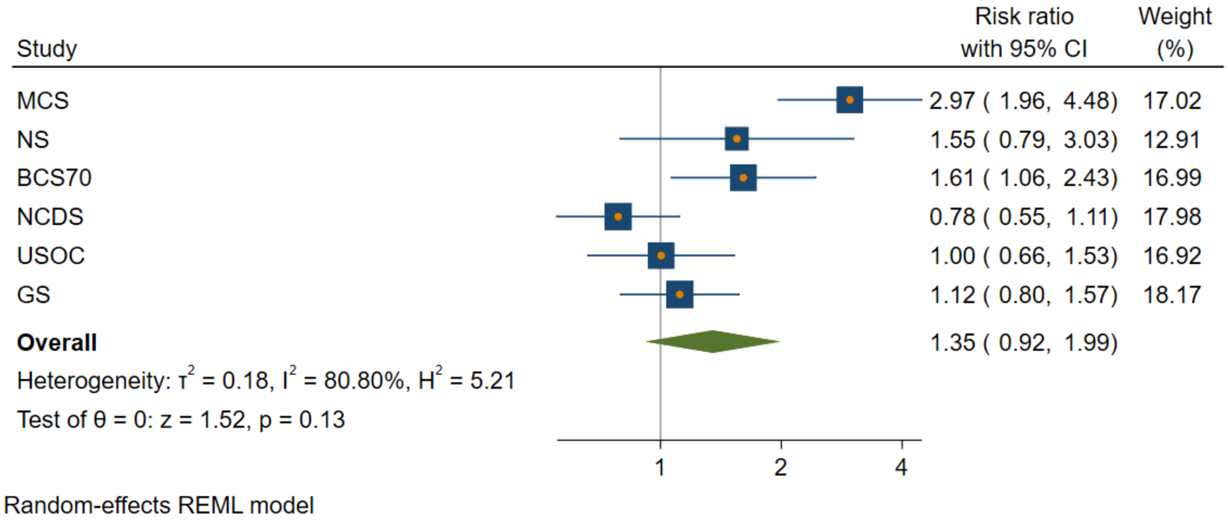

From 6/9h a night to outside 'normal range
Stable non-employed vs. Stable employed

full adjustment

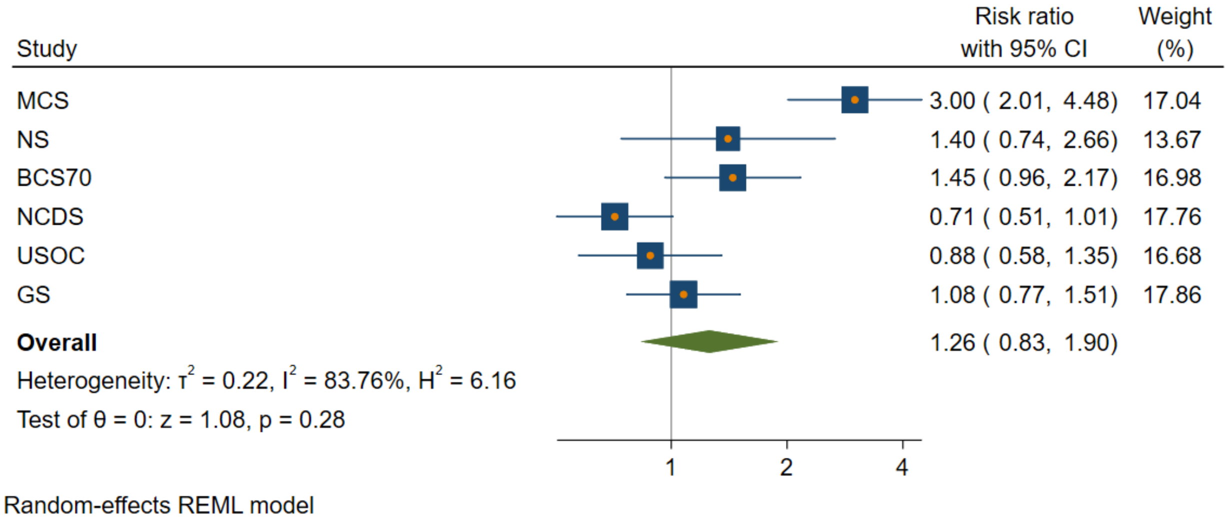

Figure set 11: From outside 'normal range' to 6/9h a night

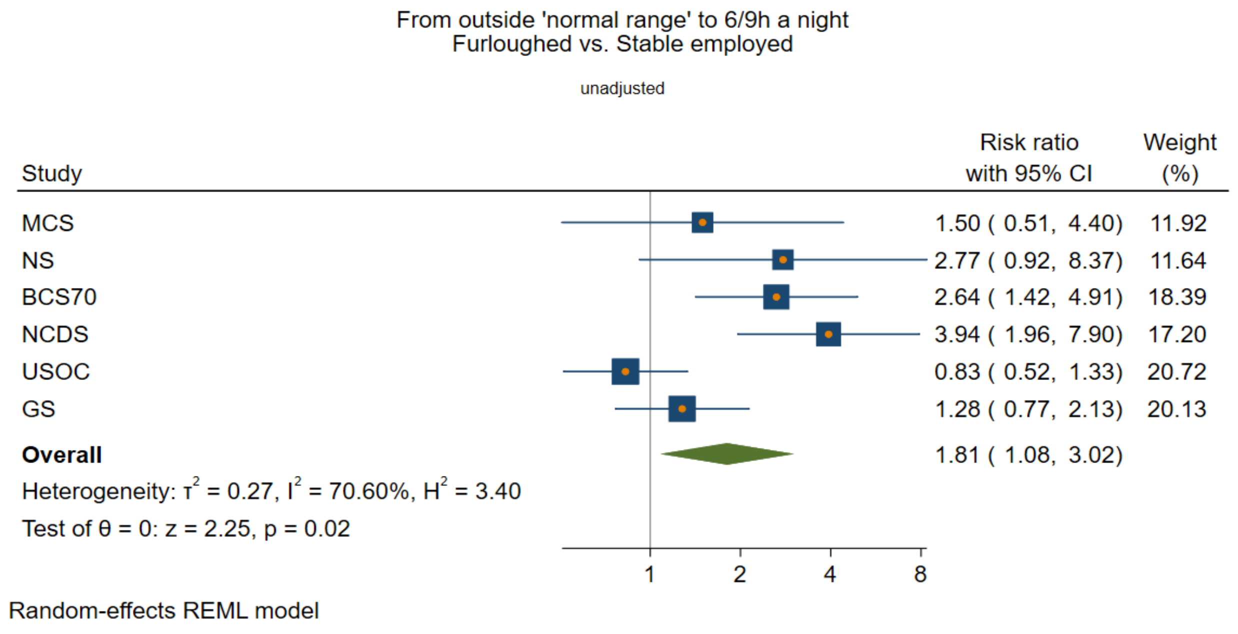

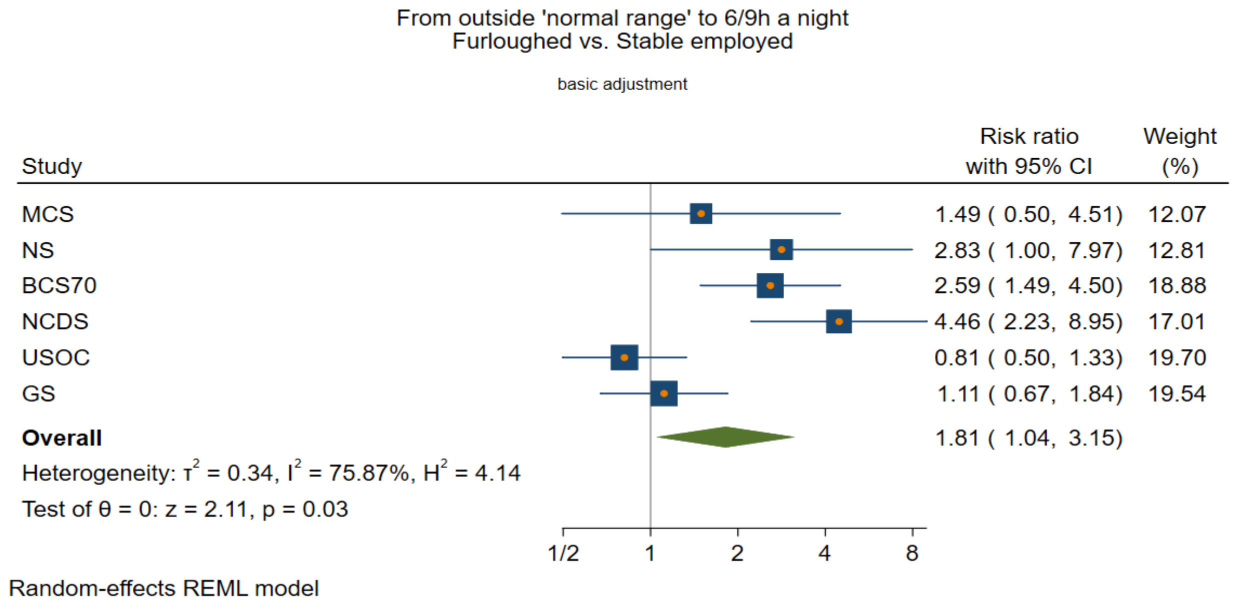

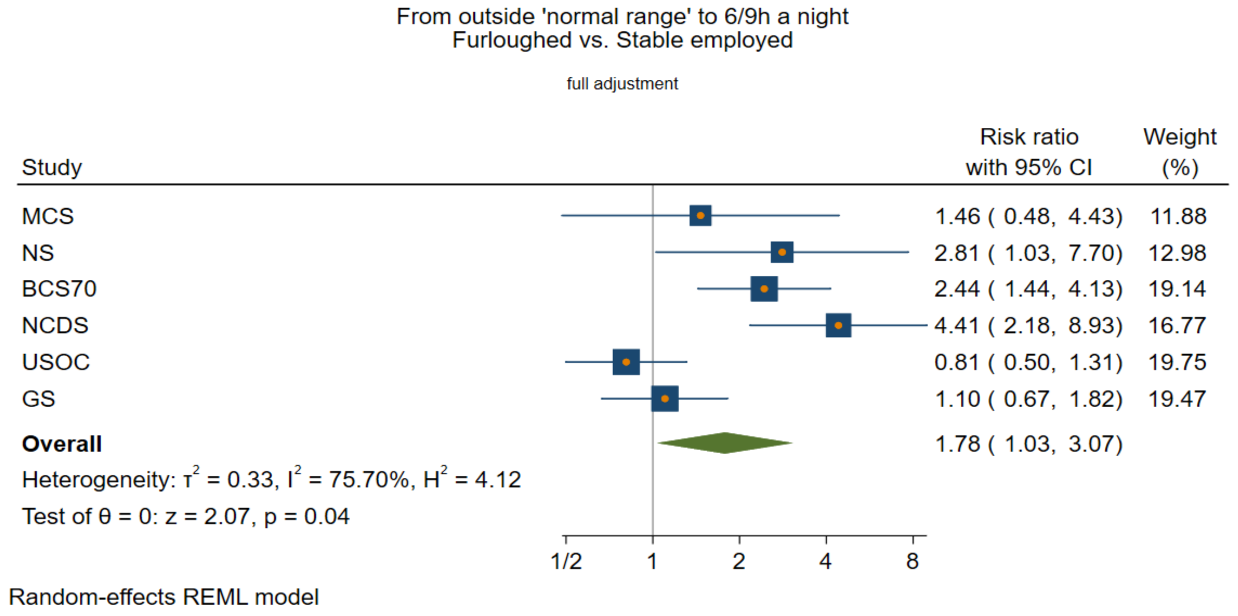

From outside 'normal range' to 6/9h a night
No longer employed vs. Stable employed

unadjusted

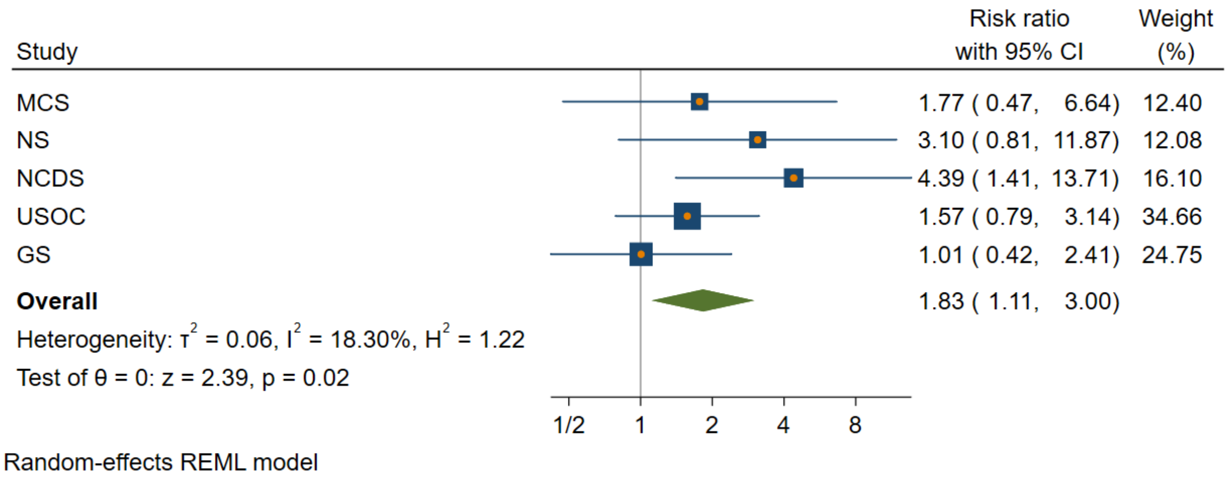

From outside 'normal range' to 6/9h a night
No longer employed vs. Stable employed

basic adjustment

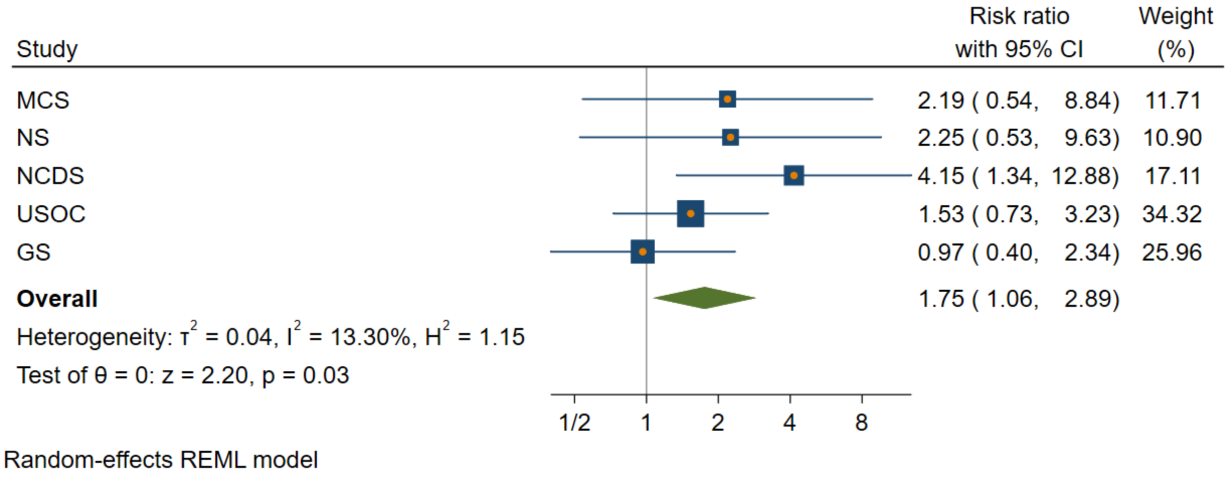

From outside 'normal range' to 6/9h a night
No longer employed vs. Stable employed

full adjustment

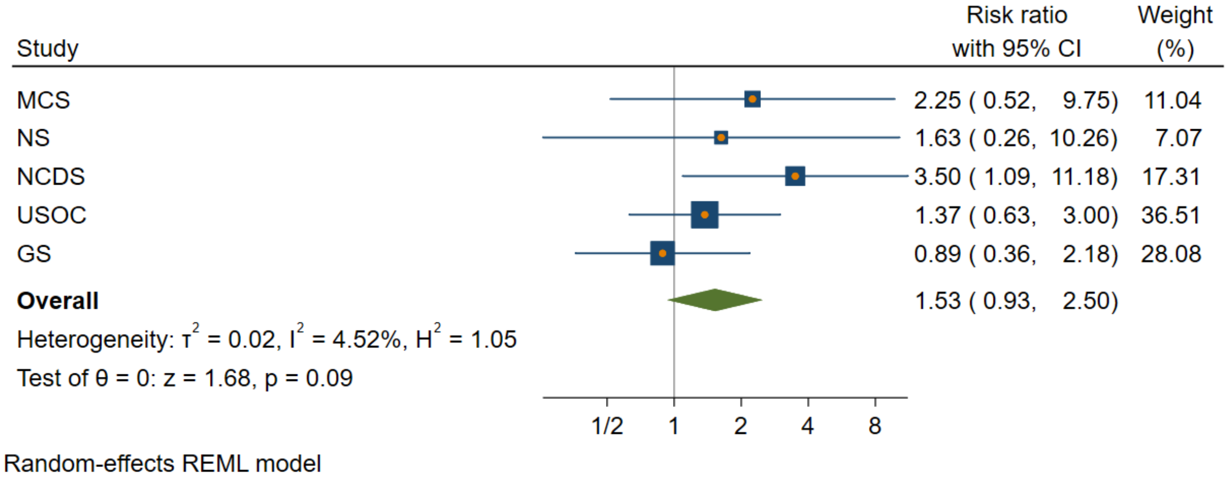

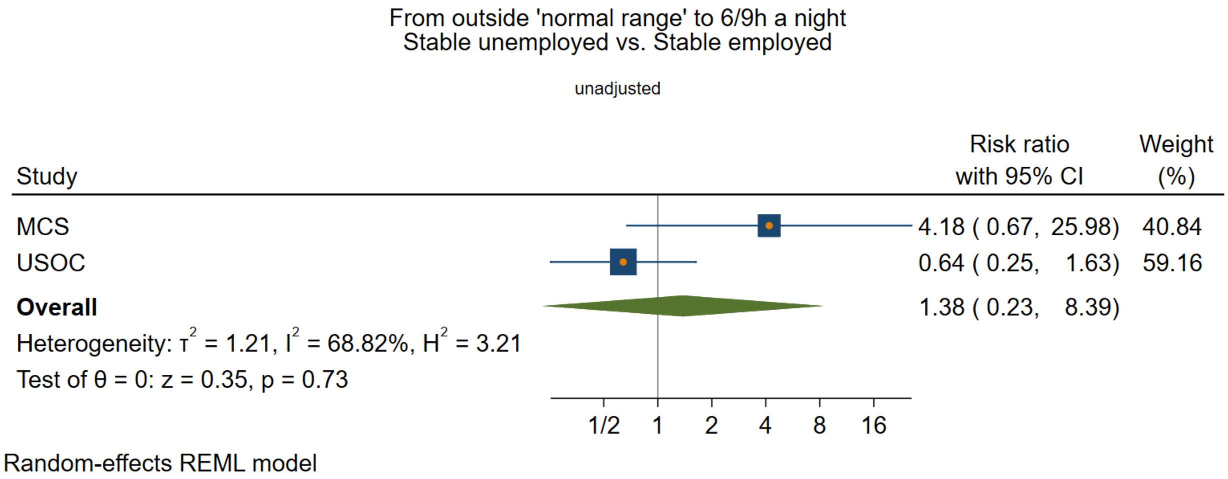
From outside 'normal range' to 6/9h a night
Stable unemployed vs. Stable employed
unadjusted
Study
Risk ratio with 95% CI
Weight (%)
MCS
4.18 (0.67, 25.98)
40.84
USOC
0.64 (0.25, 1.63)
59.16
Overall
1.38 (0.23, 8.39)
Heterogeneity: τ² = 1.21, I² = 68.82%, H² = 3.21
Test of θ = 0: z = 0.35, p = 0.73
1/2
1
2
4
8
16
Random-effects REML model

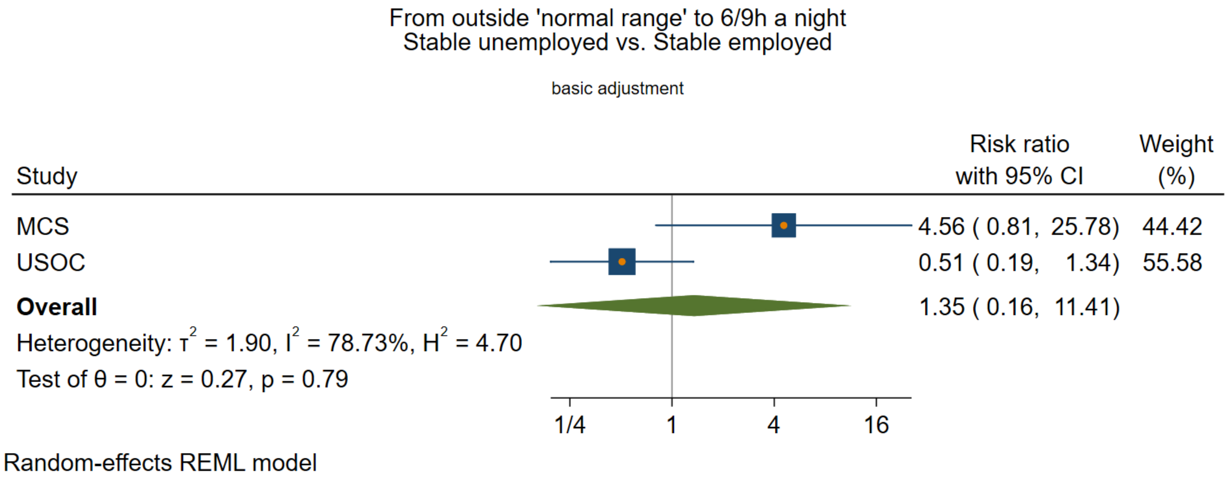
From outside 'normal range' to 6/9h a night
Stable unemployed vs. Stable employed
basic adjustment
Study
Risk ratio with 95% CI
Weight (%)
MCS
4.56 (0.81, 25.78)
44.42
USOC
0.51 (0.19, 1.34)
55.58
Overall
1.35 (0.16, 11.41)
Heterogeneity: τ² = 1.90, I² = 78.73%, H² = 4.70
Test of θ = 0: z = 0.27, p = 0.79
1/4
1
4
16
Random-effects REML model

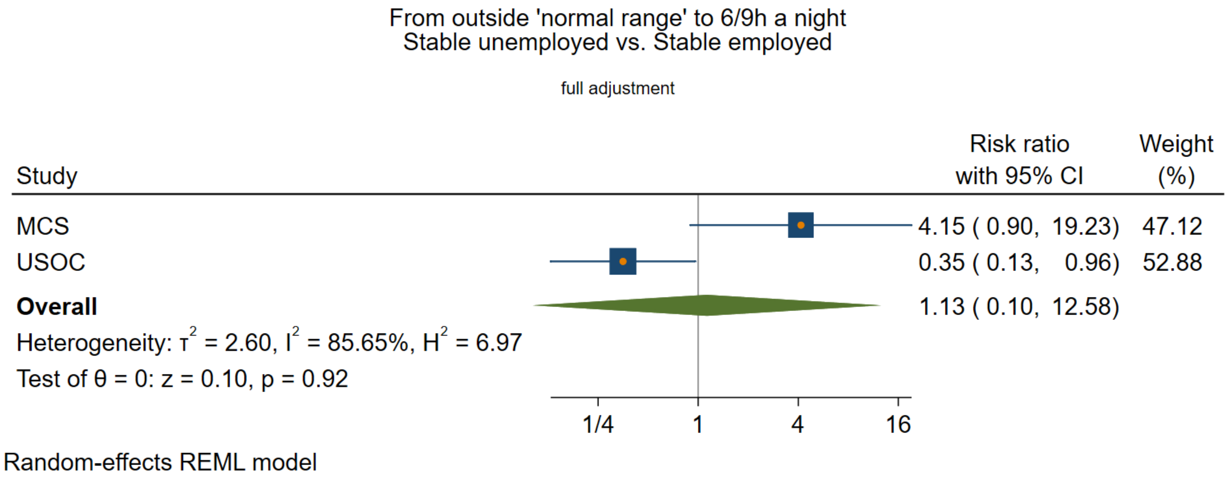
From outside 'normal range' to 6/9h a night
Stable unemployed vs. Stable employed
full adjustment
Study
Risk ratio with 95% CI
Weight (%)
MCS
4.15 (0.90, 19.23)
47.12
USOC
0.35 (0.13, 0.96)
52.88
Overall
1.13 (0.10, 12.58)
Heterogeneity: τ² = 2.60, I² = 85.65%, H² = 6.97
Test of θ = 0: z = 0.10, p = 0.92
1/4
1
4
16
Random-effects REML model

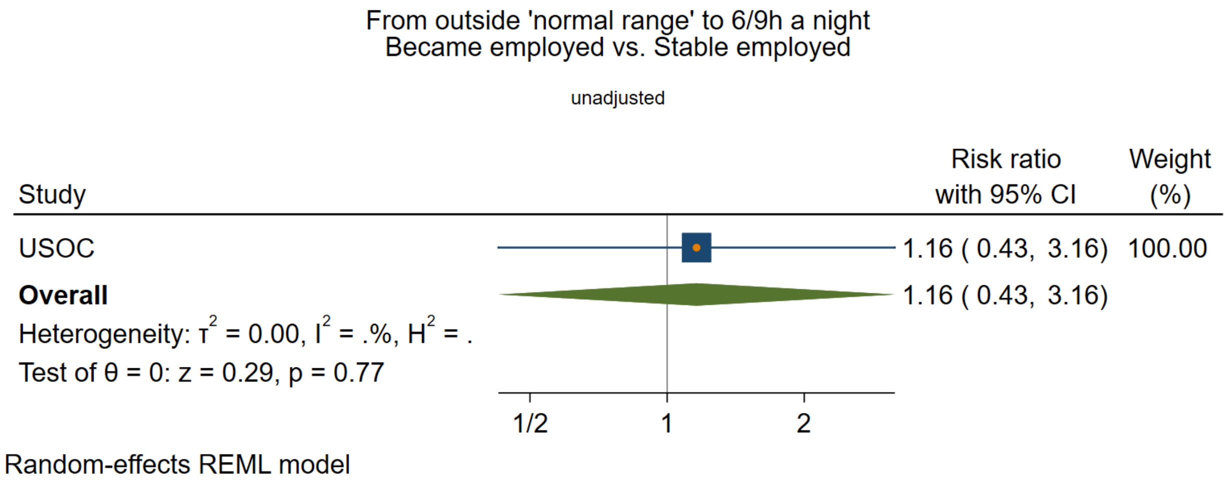
From outside 'normal range' to 6/9h a night
Became employed vs. Stable employed
unadjusted
Study
Risk ratio
with 95% CI
Weight
(%)
USOC
1.16 (0.43, 3.16)
100.00
Overall
1.16 (0.43, 3.16)
Heterogeneity: τ² = 0.00, I² = .%, H² = .
Test of θ = 0: z = 0.29, p = 0.77
1/2
1
2
Random-effects REML model

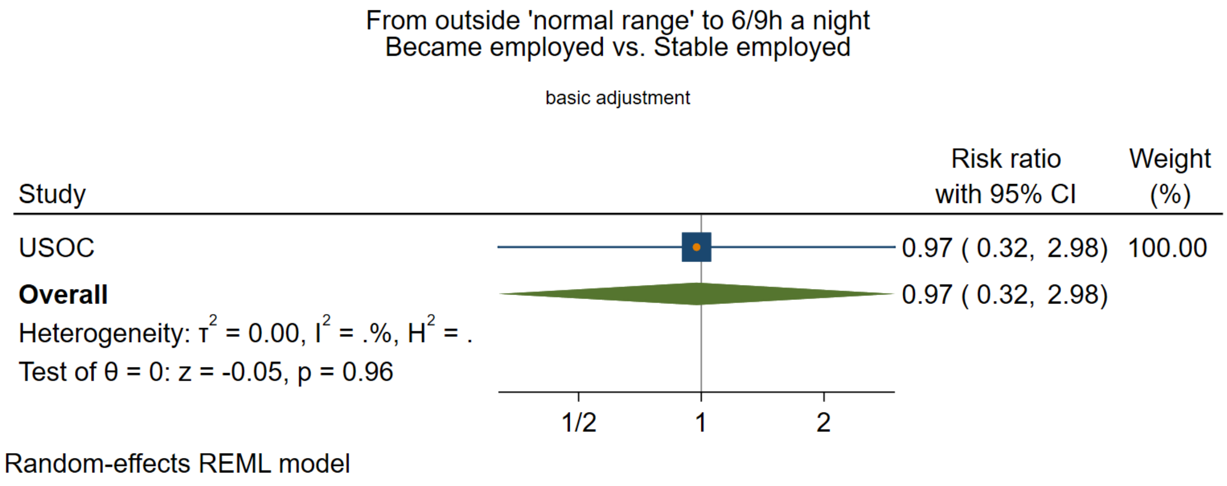
From outside 'normal range' to 6/9h a night
Became employed vs. Stable employed
basic adjustment
Study
Risk ratio
with 95% CI
Weight
(%)
USOC
0.97 (0.32, 2.98)
100.00
Overall
0.97 (0.32, 2.98)
Heterogeneity: τ² = 0.00, I² = .%, H² = .
Test of θ = 0: z = -0.05, p = 0.96
1/2
1
2
Random-effects REML model

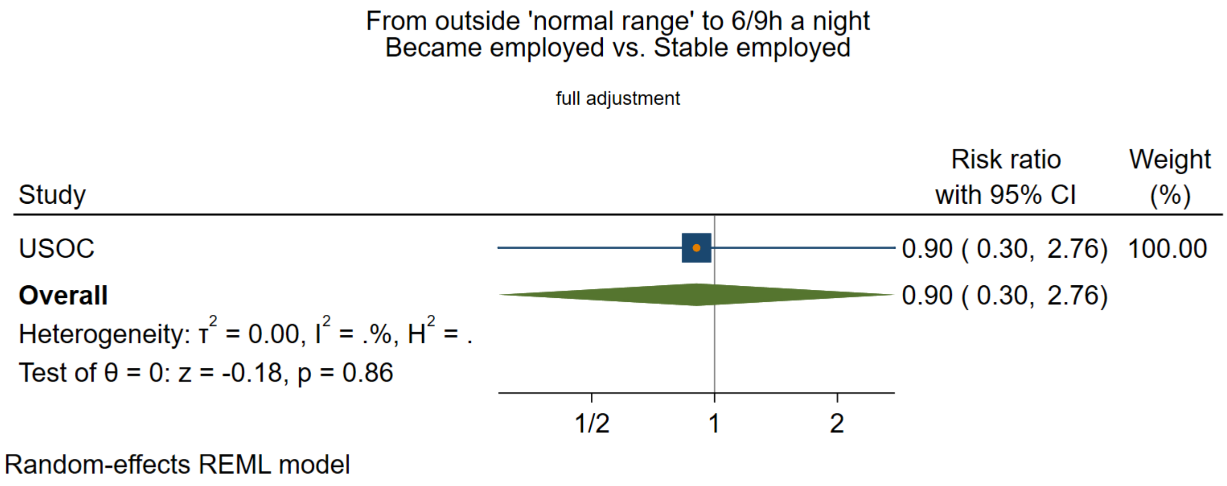
From outside 'normal range' to 6/9h a night
Became employed vs. Stable employed
full adjustment
Study
Risk ratio
with 95% CI
Weight
(%)
USOC
0.90 (0.30, 2.76)
100.00
Overall
0.90 (0.30, 2.76)
Heterogeneity: τ² = 0.00, I² = .%, H² = .
Test of θ = 0: z = -0.18, p = 0.86
1/2
1
2
Random-effects REML model

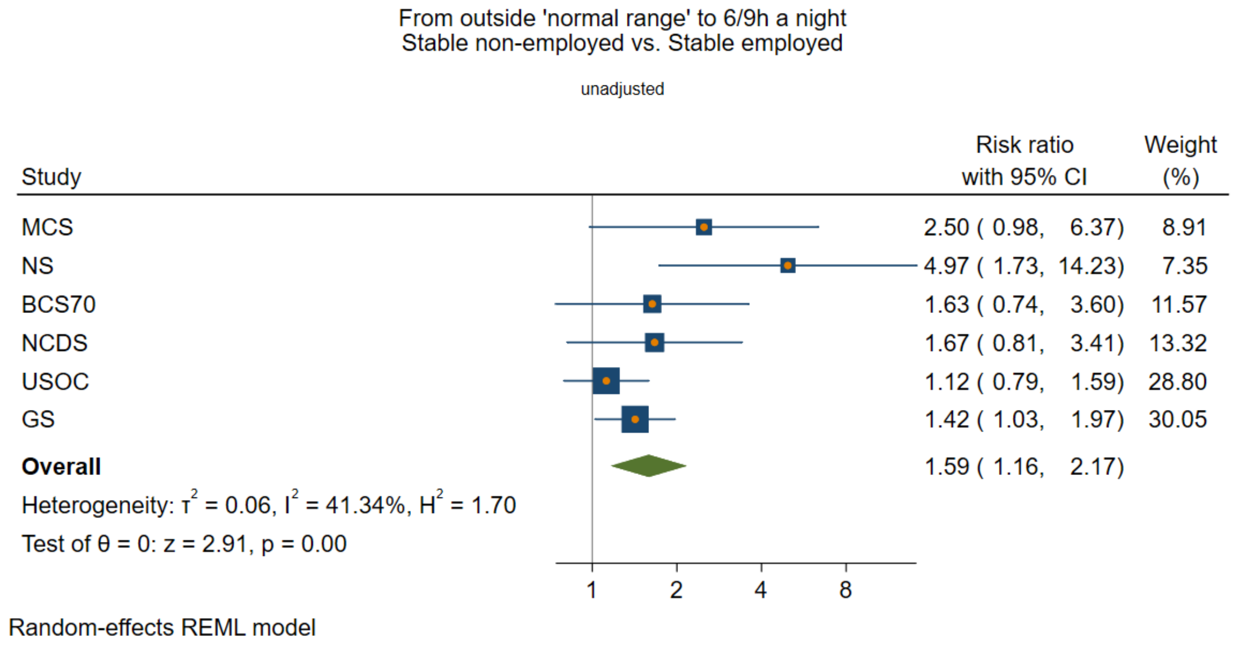
From outside 'normal range' to 6/9h a night
Stable non-employed vs. Stable employed
unadjusted
Study
Risk ratio with 95% CI
Weight (%)
MCS 2.50 (0.98, 6.37) 8.91
NS 4.97 (1.73, 14.23) 7.35
BCS70 1.63 (0.74, 3.60) 11.57
NCDS 1.67 (0.81, 3.41) 13.32
USOC 1.12 (0.79, 1.59) 28.80
GS 1.42 (1.03, 1.97) 30.05
Overall 1.59 (1.16, 2.17)
Heterogeneity: τ² = 0.06, I² = 41.34%, H² = 1.70
Test of θ = 0: z = 2.91, p = 0.00
1
2
4
8
Random-effects REML model

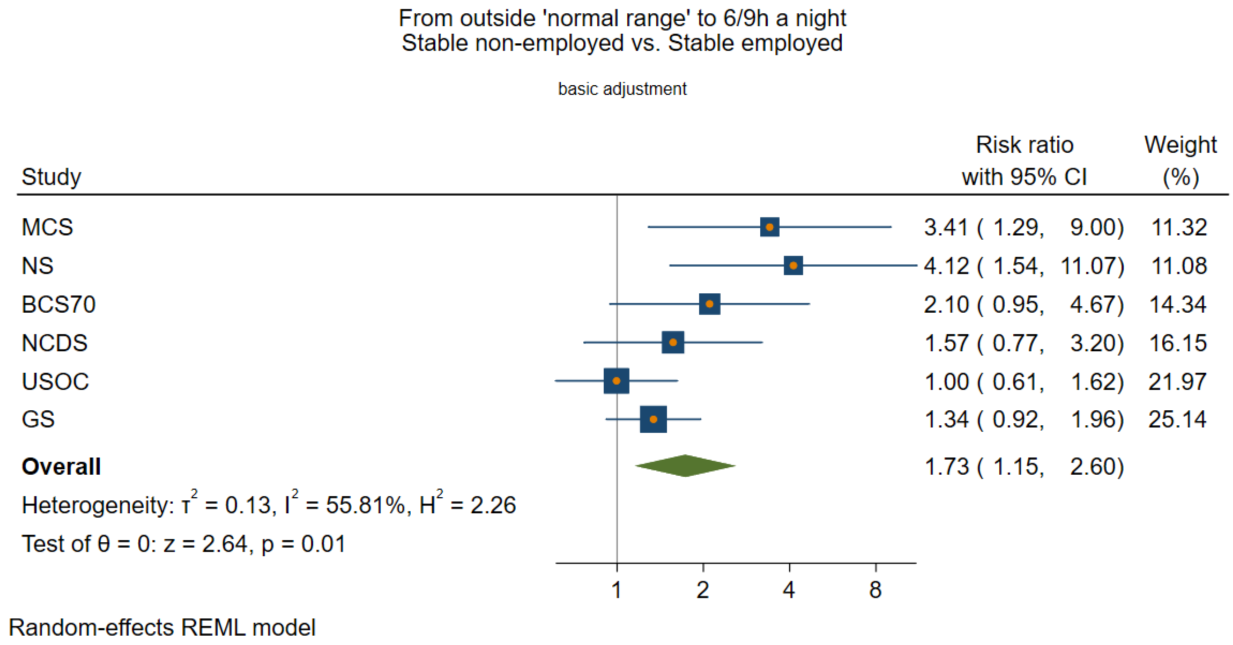
From outside 'normal range' to 6/9h a night
Stable non-employed vs. Stable employed
basic adjustment
Study
Risk ratio with 95% CI
Weight (%)
MCS 3.41 (1.29, 9.00) 11.32
NS 4.12 (1.54, 11.07) 11.08
BCS70 2.10 (0.95, 4.67) 14.34
NCDS 1.57 (0.77, 3.20) 16.15
USOC 1.00 (0.61, 1.62) 21.97
GS 1.34 (0.92, 1.96) 25.14
Overall 1.73 (1.15, 2.60)
Heterogeneity: τ² = 0.13, I² = 55.81%, H² = 2.26
Test of θ = 0: z = 2.64, p = 0.01
1
2
4
8
Random-effects REML model

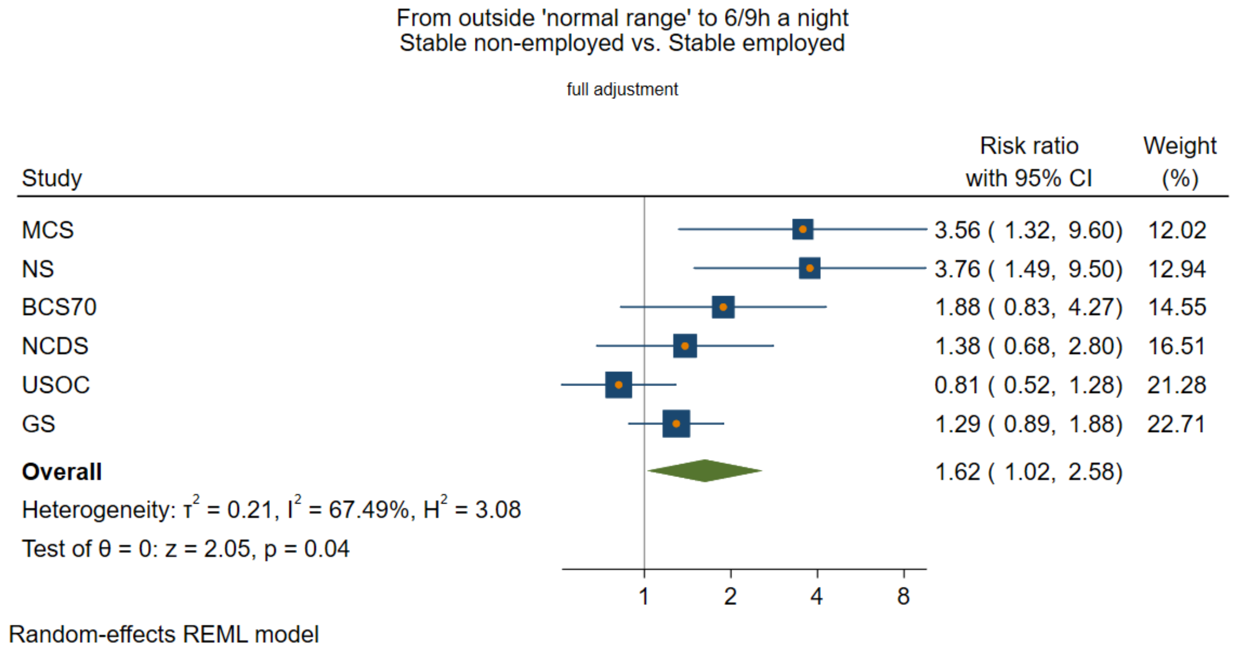
From outside 'normal range' to 6/9h a night
Stable non-employed vs. Stable employed
full adjustment
Study
Risk ratio with 95% CI
Weight (%)
MCS 3.56 (1.32, 9.60) 12.02
NS 3.76 (1.49, 9.50) 12.94
BCS70 1.88 (0.83, 4.27) 14.55
NCDS 1.38 (0.68, 2.80) 16.51
USOC 0.81 (0.52, 1.28) 21.28
GS 1.29 (0.89, 1.88) 22.71
Overall 1.62 (1.02, 2.58)
Heterogeneity: τ² = 0.21, I² = 67.49%, H² = 3.08
Test of θ = 0: z = 2.05, p = 0.04
1
2
4
8
Random-effects REML model
