# Supplementary material for: The UK Coronavirus Job Retention Scheme and diet, physical activity, and sleep during the COVID-19 pandemic: evidence from eight longitudinal population surveys
Source: BMC Med. 2022 Apr 6;20:147. doi: 10.1186/s12916-022-02343-y (PMC8984671; doi:10.1186/s12916-022-02343-y)
Supplement: Supplementary file 4 — Additional file 4. Stratified Meta-Analysis; Results stratified by Age (Figure set 1 - set 11); Education (set 12 - set 22); Sex (set 23 - set 33). Contents: Figure set 1; 11; 23: Currently eats 2 or fewer fruit & veg. Figure set 2; 12; 24: Fewer fruit & veg. Figure set 3; 13; 25: More fruit & veg. Figure set 4; 14; 26: Less than 3 days a week of at least 30min exercise. Figure set 5; 15; 27: Less time/ fewer days of physical exercise. Figure set 6; 16; 28: More time/ days of physical exercise. Figure set 7, 17; 29: Sleeps outside ‘Normal Range’ (i.e. <6 or 9+ hours). Figure set 8, 18, 30: Sleeps less than before. Figure set 9,19, 31: Sleeps less than before. Figure set 10, 20, 32: From 6/9h a night to outside ‘normal range’. Figure set 11, 12, 33: From outside ‘normal range’ to 6/9h a night. [file 12916_2022_2343_MOESM4_ESM.pdf]

Additional File 4: Stratified Results

Results stratified by Age

Figure set 1: Currently eats 2 or fewer fruit & veg ... 3

Figure set 2: Fewer fruit & veg ... 5

Figure set 3: More fruit & veg ... 7

Figure set 4: Less than 3 days a week of at least 30min exercise ... 9

Figure set 5: Less time/ fewer days of physical exercise ... 11

Figure set 6: More time/ days of physical exercise ... 13

Figure set 7: Sleeps outside ‘Normal Range’ (i.e. <6 or 9+ hours) ... 15

Figure set 8: Sleeps less than before ... 17

Figure set 9: Sleeps less than before ... 19

Figure set 10: From 6/9h a night to outside ‘normal range’ ... 22

Figure set 11: From outside ‘normal range’ to 6/9h a night ... 24

Results stratified by Education

Figure set 12: Currently eats 2 or fewer fruit and veg ... 26

Figure set 13: Fewer fruit and veg ... 28

Figure set 14: More fruit and veg ... 30

Figure set 15: Less than 3 days a week of at least 30min exercise ... 32

Figure set 16: Less time/ fewer days of physical exercise ... 34

Figure set 17: More time/ days of physical exercise ... 37

Figure set 18: Sleeps outside ‘normal range’ (i.e. <6 or 9+ hours) ... 40

Figure set 19: Sleeps less than before ... 42

Figure set 20: Sleeps more than before ... 45

Figure set 21: From 6/9h a night to ‘normal range’ ... 48

Figure set 22: From ‘normal range’ to 6/9h a night ... 50

Results stratified by Sex

Figure set 23: Currently eats 2 or fewer fruit and veg 52
Figure set 24: Fewer fruit and veg 54
Figure set 25: More fruit and veg 56
Figure set 26: Less than 3 days a week of at least 30min exercise 58
Figure set 27: Less time/fewer days of physical exercise 60
Figure set 28: More time/ days of physical exercise 63
Figure set 29: Sleeps outside ‘normal range’ (i.e. <6 or 9+ hours) 66
Figure set 30: Sleeps less than before 68
Figure set 31: Sleeps more than before 71
Figure set 32: From 6/9h a night to outside ‘normal range’ 74
Figure set 33: From outside ‘normal range’ to 6/9h a night 76

Notes: Moderation by age, education, and sex was assessed with stratified regressions using “full” adjustment that includes socio-demographic characteristics as well as pre-pandemic measures of psychological distress, self-rated health, and health behaviours

Figure set 1: Currently eats 2 or fewer fruit & veg

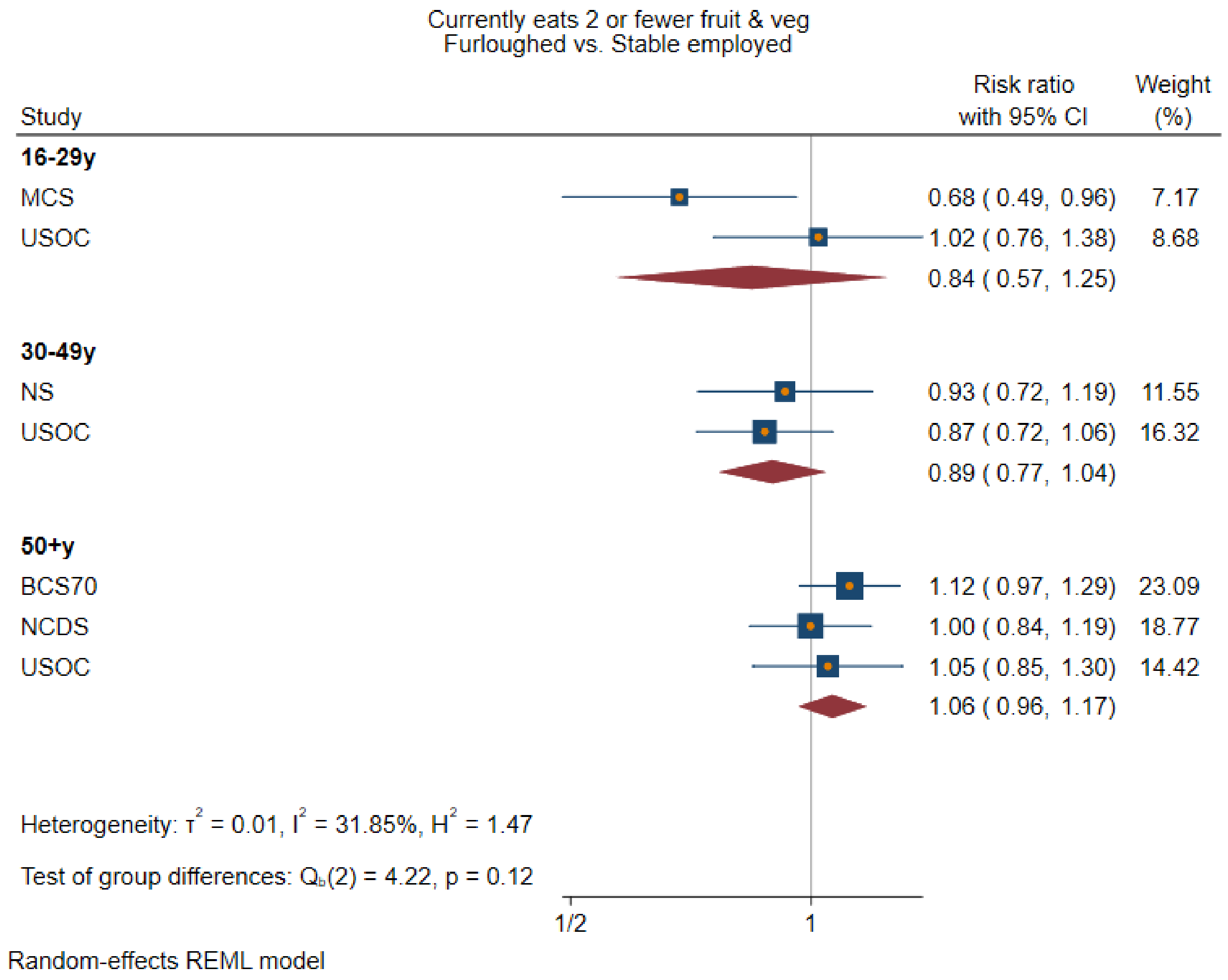

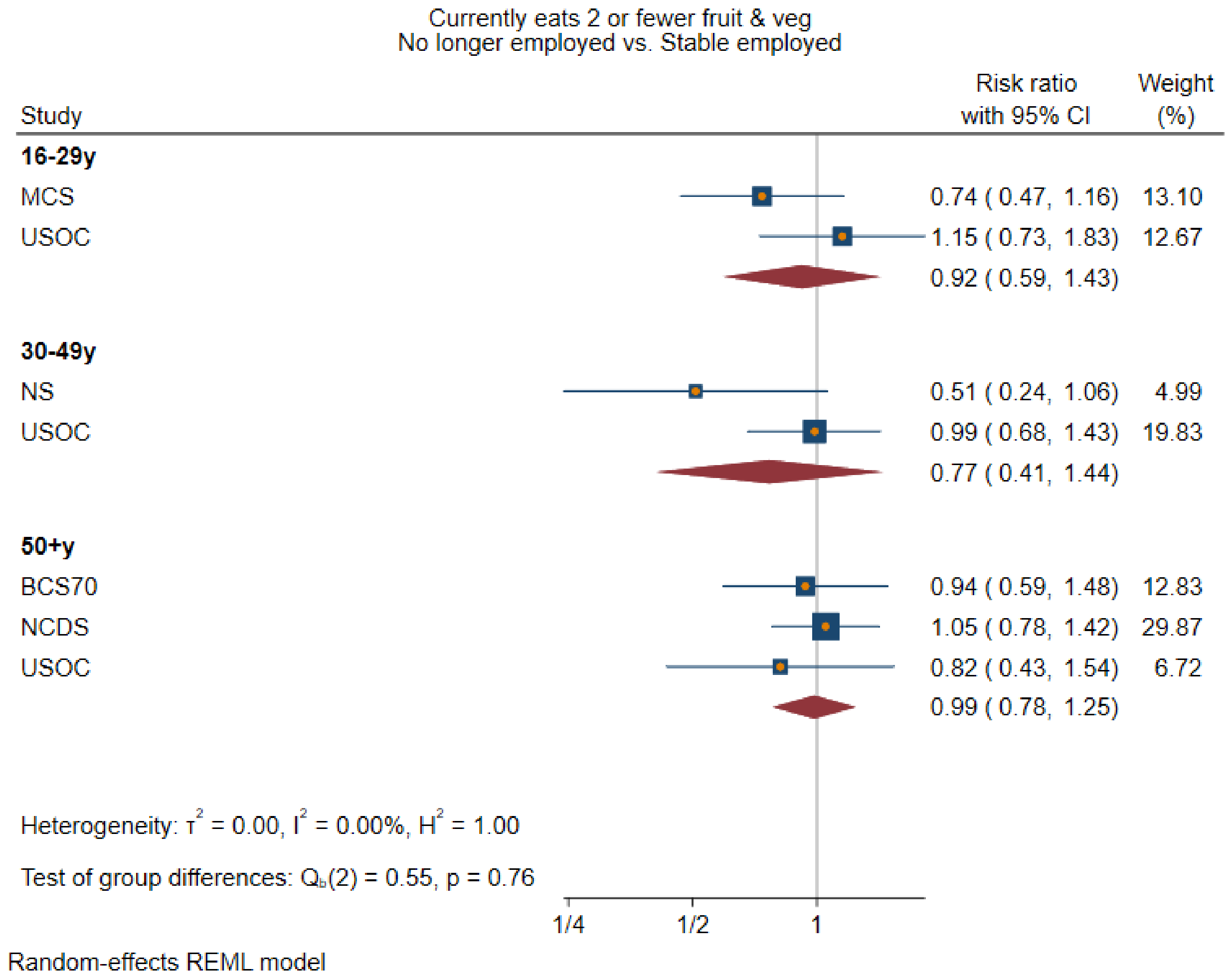

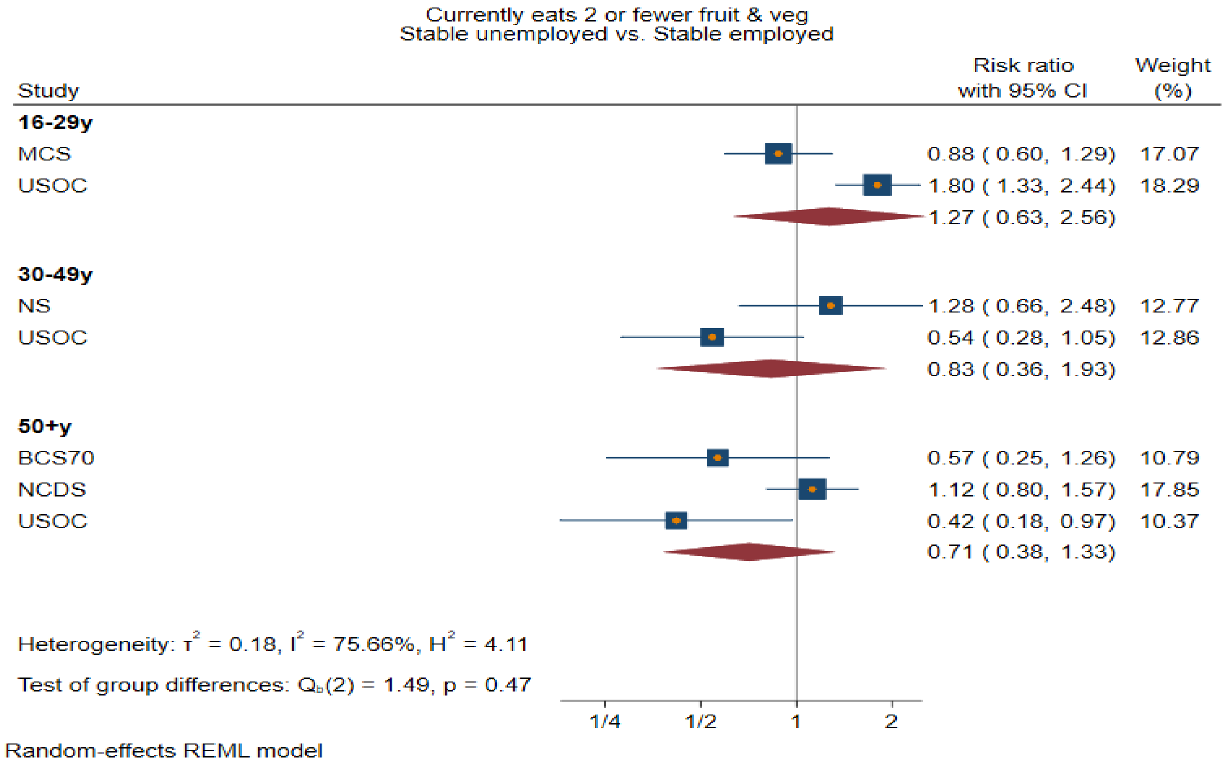
Currently eats 2 or fewer fruit & veg
Stable unemployed vs. Stable employed
Study
Risk ratio with 95% CI
Weight (%)
16-29y
MCS 0.88 (0.60, 1.29) 17.07
USOC 1.80 (1.33, 2.44) 18.29
1.27 (0.63, 2.56)
30-49y
NS 1.28 (0.66, 2.48) 12.77
USOC 0.54 (0.28, 1.05) 12.86
0.83 (0.36, 1.93)
50+y
BCS70 0.57 (0.25, 1.26) 10.79
NCDS 1.12 (0.80, 1.57) 17.85
USOC 0.42 (0.18, 0.97) 10.37
0.71 (0.38, 1.33)
Heterogeneity: τ^2 = 0.18, I^2 = 75.66%, H^2 = 4.11
Test of group differences: $Q_b(2)$ = 1.49, p = 0.47
1/4 1/2 1 2
Random-effects REML model

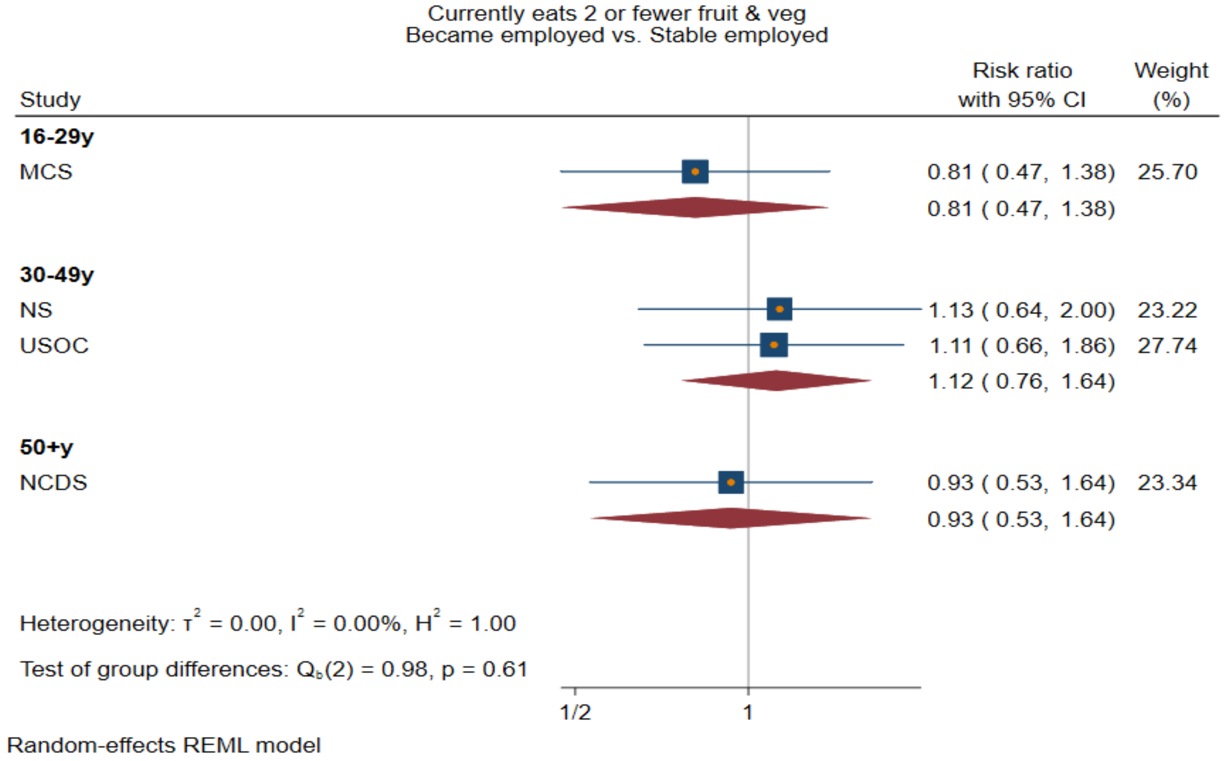
Currently eats 2 or fewer fruit & veg
Became employed vs. Stable employed
Study
Risk ratio with 95% CI
Weight (%)
16-29y
MCS 0.81 (0.47, 1.38) 25.70
0.81 (0.47, 1.38)
30-49y
NS 1.13 (0.64, 2.00) 23.22
USOC 1.11 (0.66, 1.86) 27.74
1.12 (0.76, 1.64)
50+y
NCDS 0.93 (0.53, 1.64) 23.34
0.93 (0.53, 1.64)
Heterogeneity: τ^2 = 0.00, I^2 = 0.00%, H^2 = 1.00
Test of group differences: $Q_b(2)$ = 0.98, p = 0.61
1/2 1
Random-effects REML model

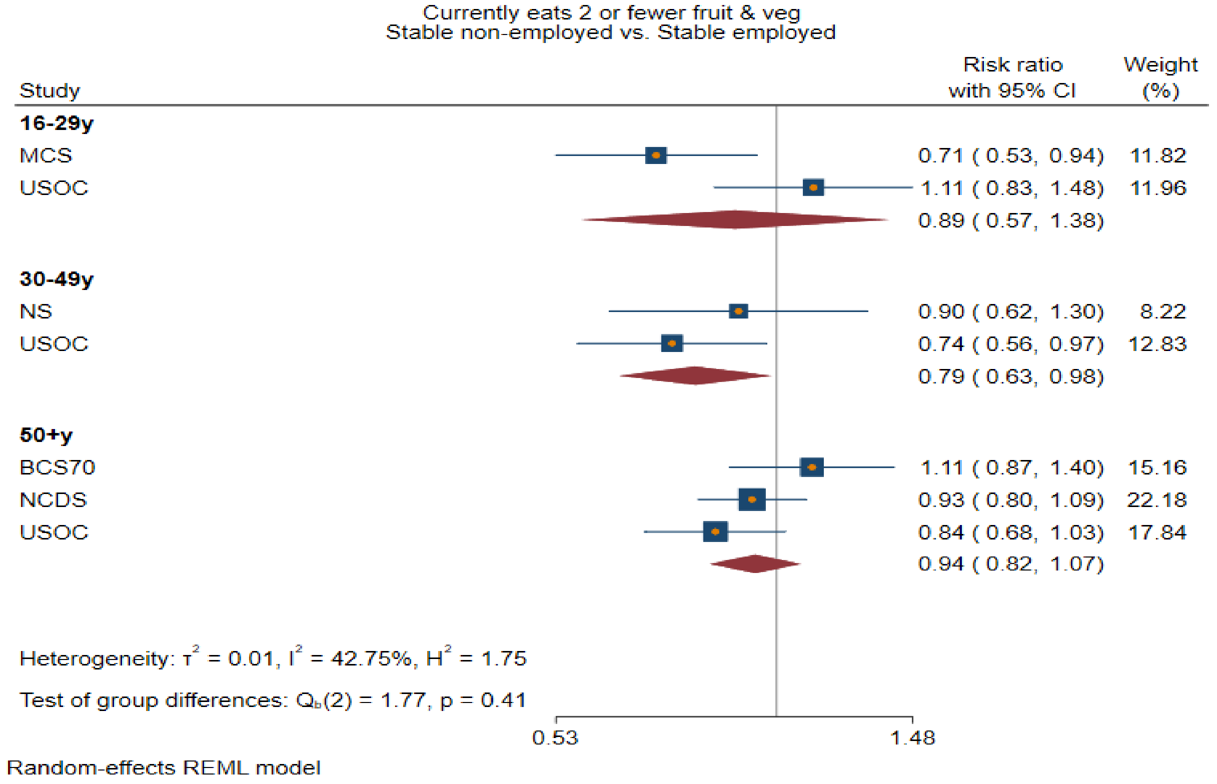
Currently eats 2 or fewer fruit & veg
Stable non-employed vs. Stable employed
Study
Risk ratio with 95% CI
Weight (%)
16-29y
MCS 0.71 (0.53, 0.94) 11.82
USOC 1.11 (0.83, 1.48) 11.96
0.89 (0.57, 1.38)
30-49y
NS 0.90 (0.62, 1.30) 8.22
USOC 0.74 (0.56, 0.97) 12.83
0.79 (0.63, 0.98)
50+y
BCS70 1.11 (0.87, 1.40) 15.16
NCDS 0.93 (0.80, 1.09) 22.18
USOC 0.84 (0.68, 1.03) 17.84
0.94 (0.82, 1.07)
Heterogeneity: τ^2 = 0.01, I^2 = 42.75%, H^2 = 1.75
Test of group differences: $Q_b(2)$ = 1.77, p = 0.41
0.53 1.48
Random-effects REML model

Figure set 2: Fewer fruit & veg

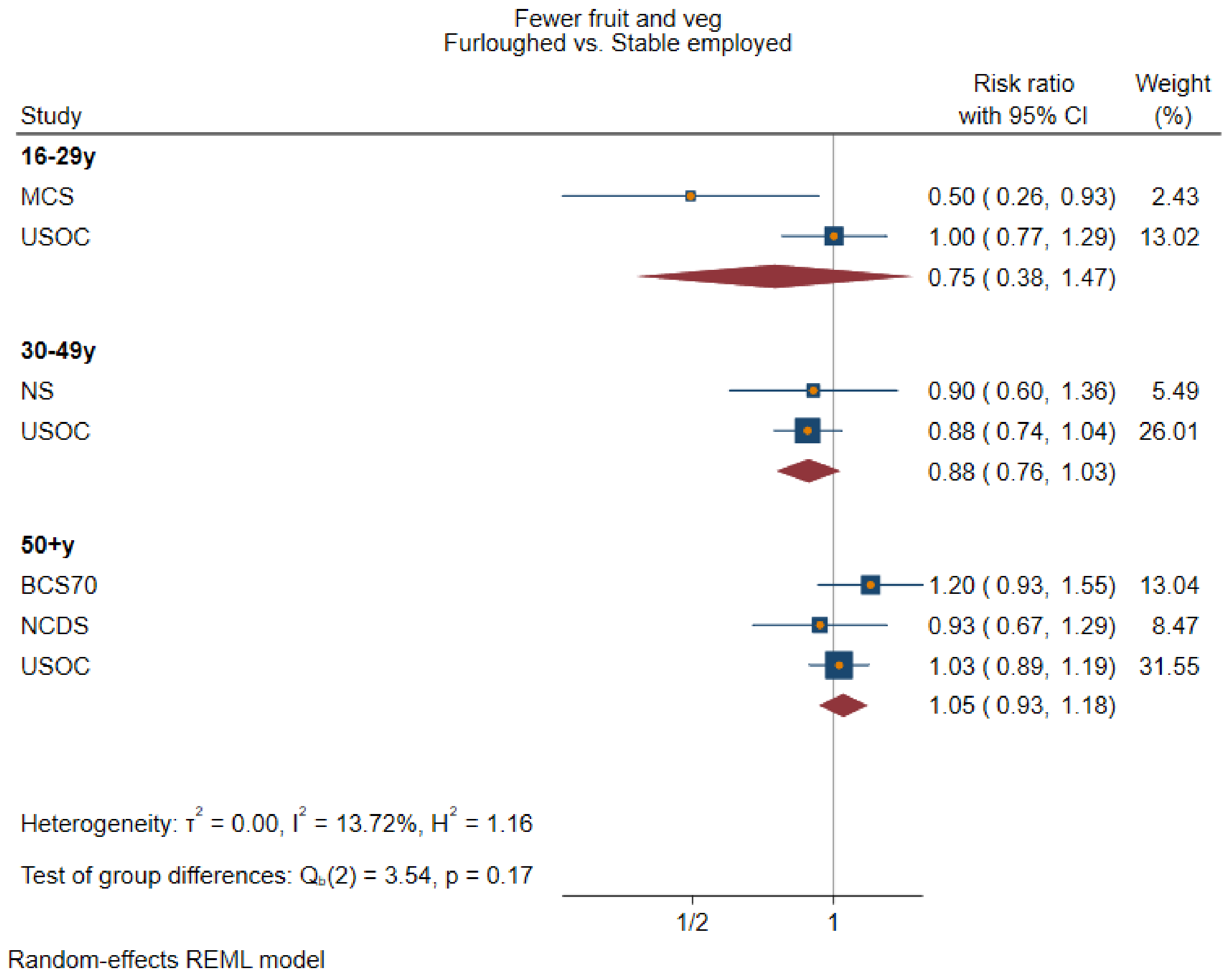

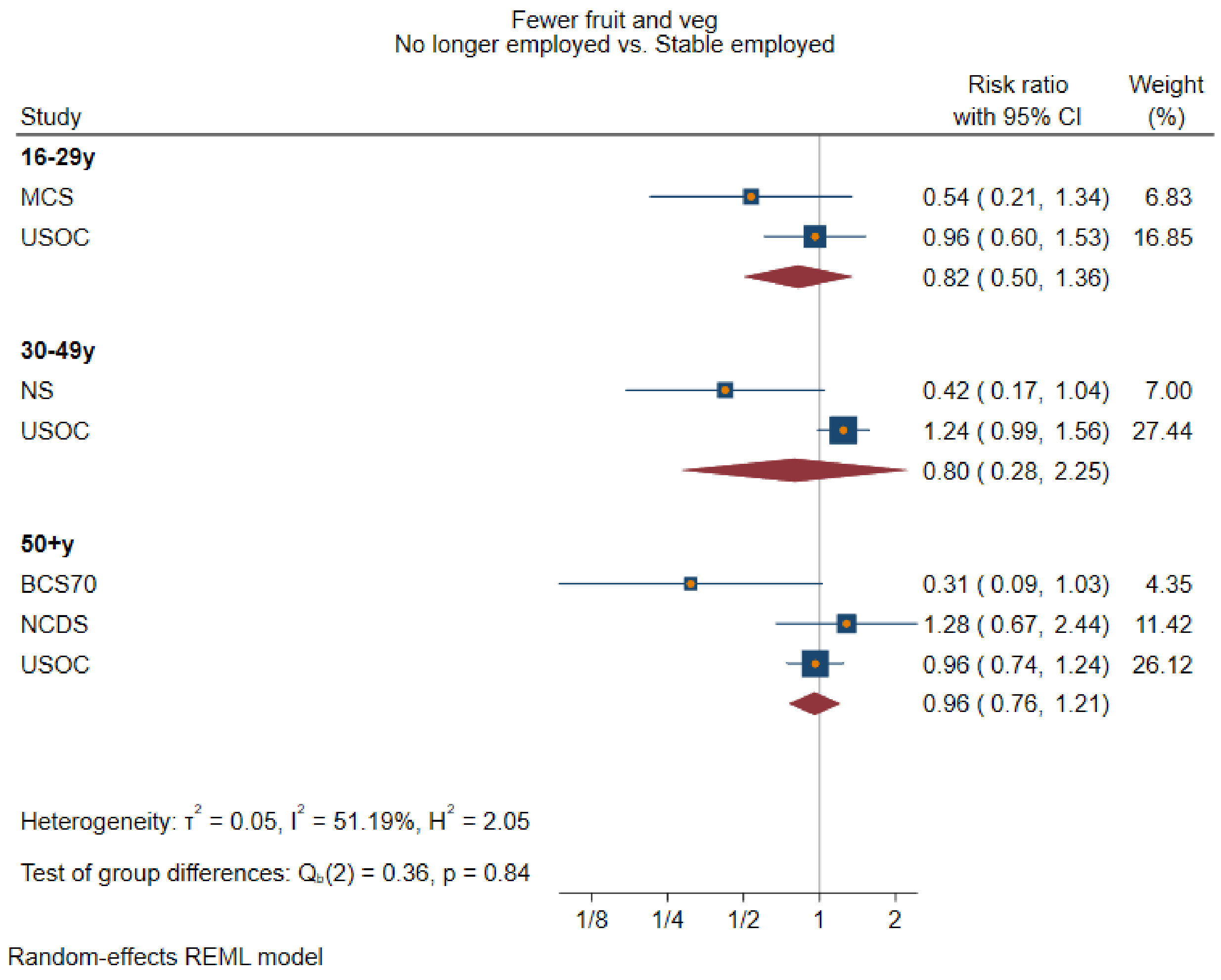

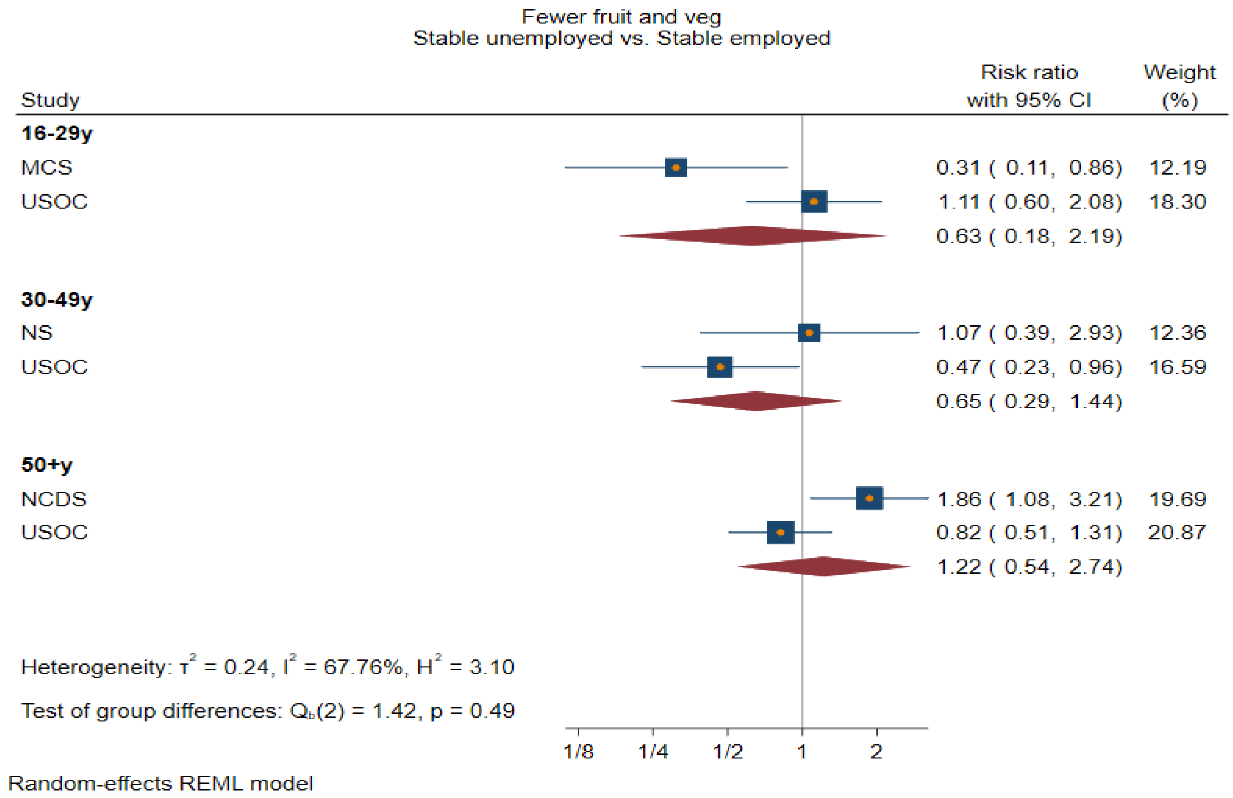
Fewer fruit and veg
Stable unemployed vs. Stable employed
Study
Risk ratio with 95% CI
Weight (%)
16-29y
MCS 0.31 (0.11, 0.86) 12.19
USOC 1.11 (0.60, 2.08) 18.30
0.63 (0.18, 2.19)
30-49y
NS 1.07 (0.39, 2.93) 12.36
USOC 0.47 (0.23, 0.96) 16.59
0.65 (0.29, 1.44)
50+y
NCDS 1.86 (1.08, 3.21) 19.69
USOC 0.82 (0.51, 1.31) 20.87
1.22 (0.54, 2.74)
Heterogeneity: τ^2 = 0.24, I^2 = 67.76%, H^2 = 3.10
Test of group differences: $Q_b(2)$ = 1.42, p = 0.49
1/8 1/4 1/2 1 2
Random-effects REML model

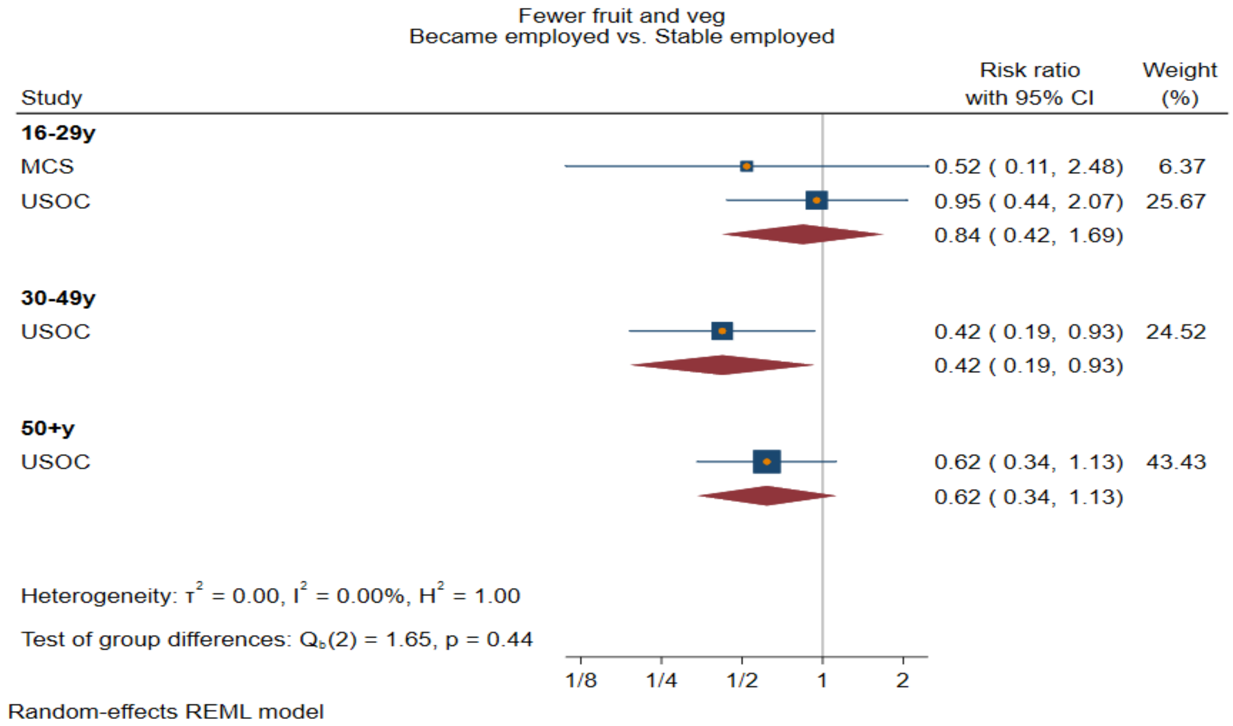
Fewer fruit and veg
Became employed vs. Stable employed
Study
Risk ratio with 95% CI
Weight (%)
16-29y
MCS 0.52 (0.11, 2.48) 6.37
USOC 0.95 (0.44, 2.07) 25.67
0.84 (0.42, 1.69)
30-49y
USOC 0.42 (0.19, 0.93) 24.52
0.42 (0.19, 0.93)
50+y
USOC 0.62 (0.34, 1.13) 43.43
0.62 (0.34, 1.13)
Heterogeneity: τ^2 = 0.00, I^2 = 0.00%, H^2 = 1.00
Test of group differences: $Q_b(2)$ = 1.65, p = 0.44
1/8 1/4 1/2 1 2
Random-effects REML model

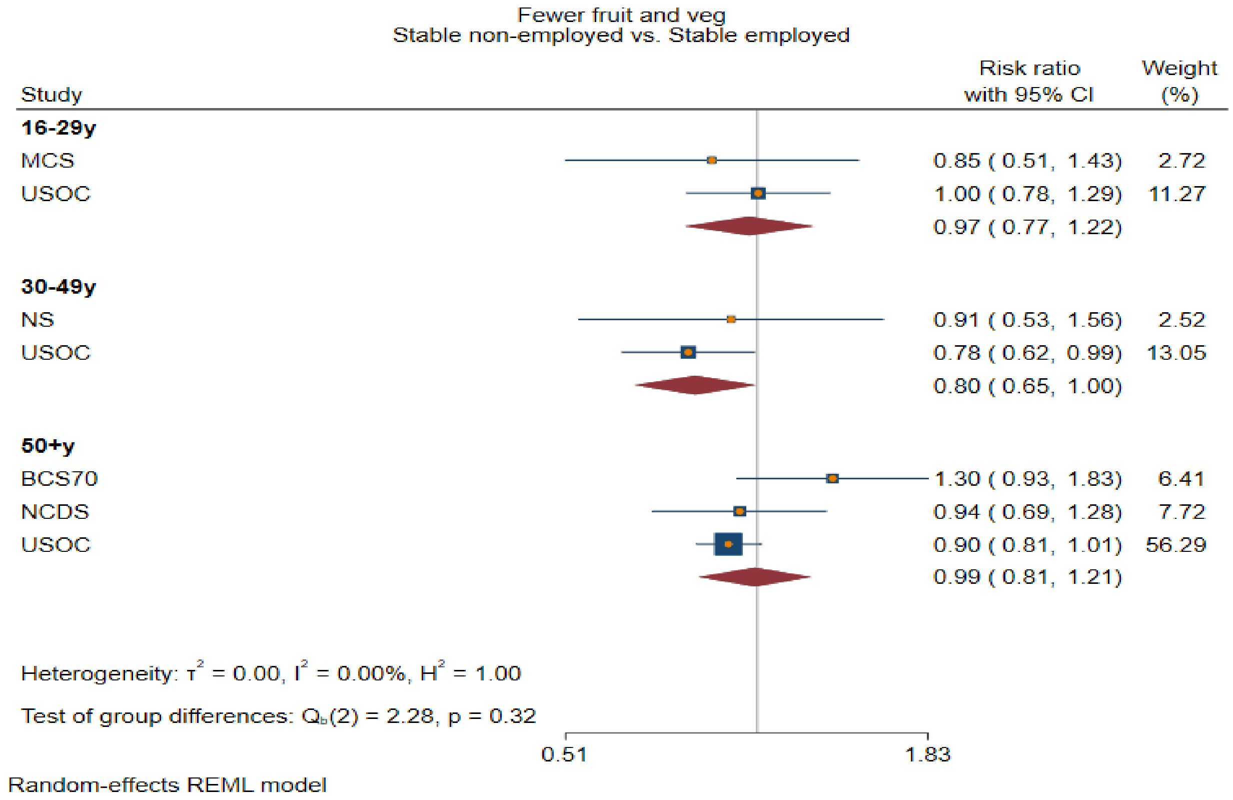
Fewer fruit and veg
Stable non-employed vs. Stable employed
Study
Risk ratio with 95% CI
Weight (%)
16-29y
MCS 0.85 (0.51, 1.43) 2.72
USOC 1.00 (0.78, 1.29) 11.27
0.97 (0.77, 1.22)
30-49y
NS 0.91 (0.53, 1.56) 2.52
USOC 0.78 (0.62, 0.99) 13.05
0.80 (0.65, 1.00)
50+y
BCS70 1.30 (0.93, 1.83) 6.41
NCDS 0.94 (0.69, 1.28) 7.72
USOC 0.90 (0.81, 1.01) 56.29
0.99 (0.81, 1.21)
Heterogeneity: τ^2 = 0.00, I^2 = 0.00%, H^2 = 1.00
Test of group differences: $Q_b(2)$ = 2.28, p = 0.32
0.51 1.83
Random-effects REML model

Figure set 3: More fruit & veg

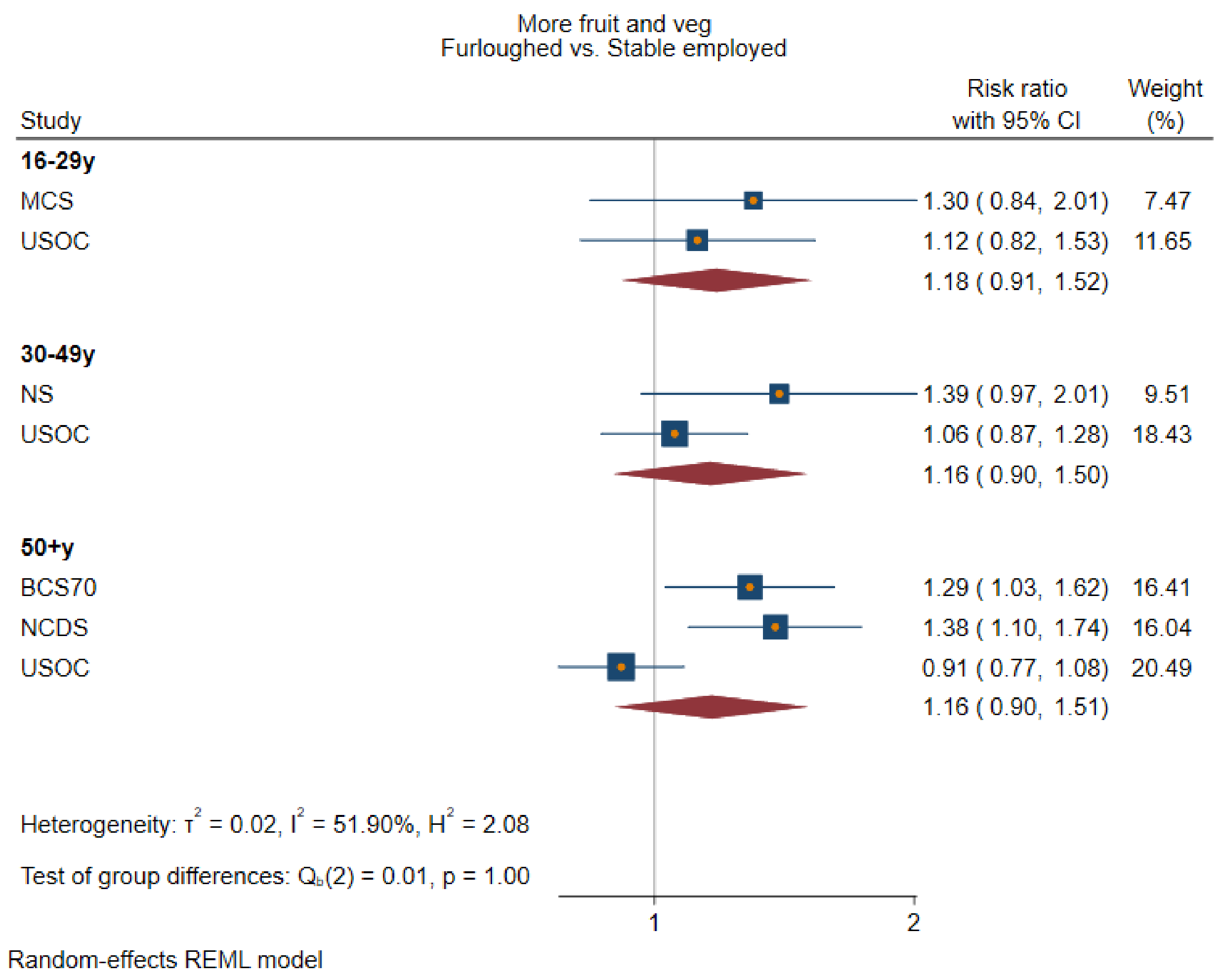

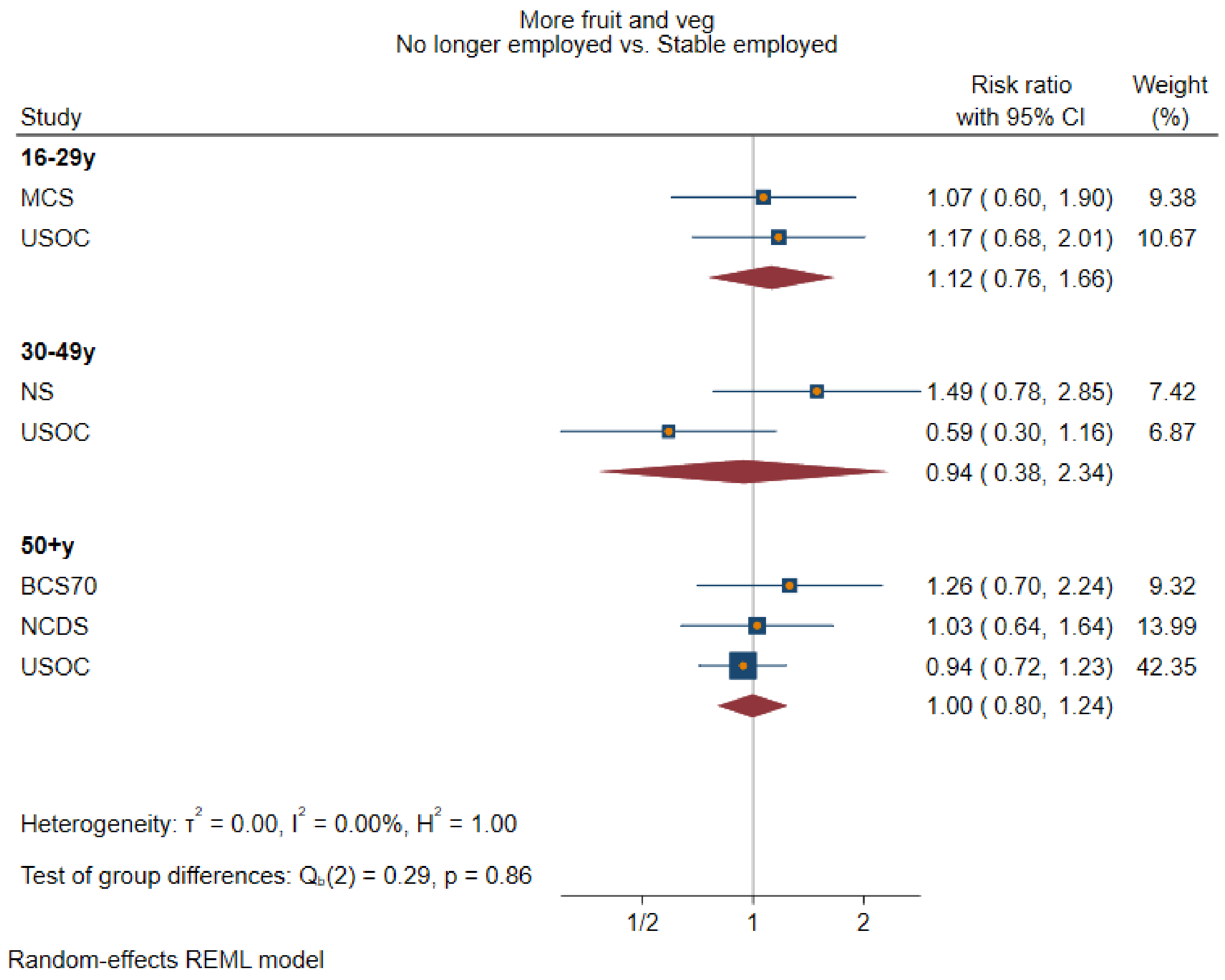

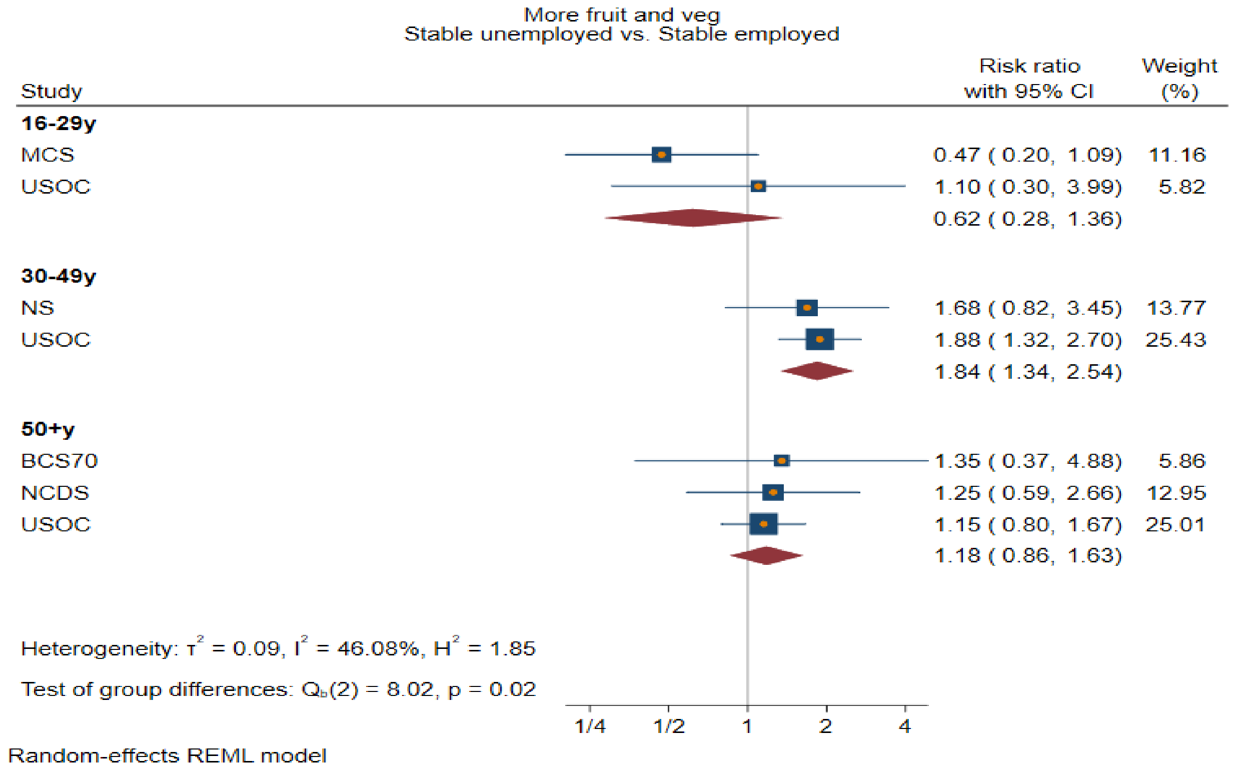
More fruit and veg
Stable unemployed vs. Stable employed
Study
Risk ratio with 95% CI
Weight (%)
16-29y
MCS 0.47 (0.20, 1.09) 11.16
USOC 1.10 (0.30, 3.99) 5.82
0.62 (0.28, 1.36)
30-49y
NS 1.68 (0.82, 3.45) 13.77
USOC 1.88 (1.32, 2.70) 25.43
1.84 (1.34, 2.54)
50+y
BCS70 1.35 (0.37, 4.88) 5.86
NCDS 1.25 (0.59, 2.66) 12.95
USOC 1.15 (0.80, 1.67) 25.01
1.18 (0.86, 1.63)
Heterogeneity: τ² = 0.09, I² = 46.08%, H² = 1.85
Test of group differences: Q_b(2) = 8.02, p = 0.02
1/4 1/2 1 2 4
Random-effects REML model

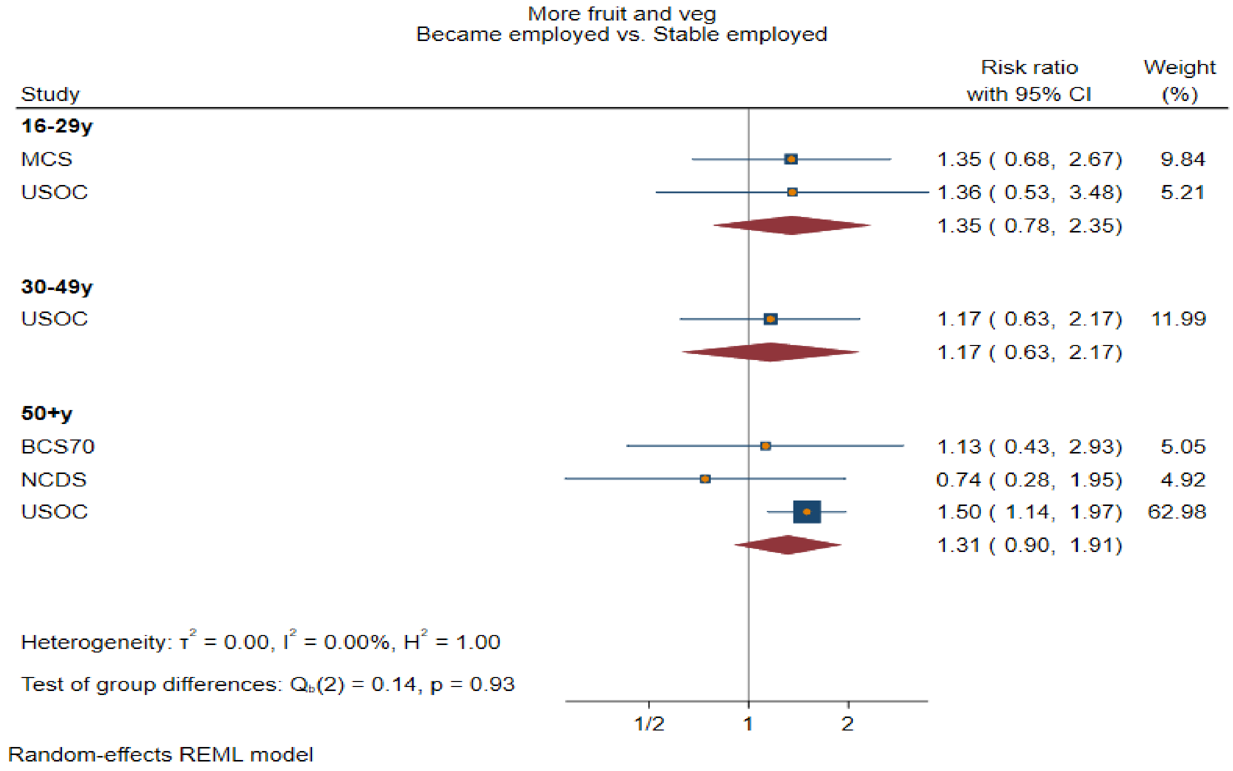
More fruit and veg
Became employed vs. Stable employed
Study
Risk ratio with 95% CI
Weight (%)
16-29y
MCS 1.35 (0.68, 2.67) 9.84
USOC 1.36 (0.53, 3.48) 5.21
1.35 (0.78, 2.35)
30-49y
USOC 1.17 (0.63, 2.17) 11.99
1.17 (0.63, 2.17)
50+y
BCS70 1.13 (0.43, 2.93) 5.05
NCDS 0.74 (0.28, 1.95) 4.92
USOC 1.50 (1.14, 1.97) 62.98
1.31 (0.90, 1.91)
Heterogeneity: τ² = 0.00, I² = 0.00%, H² = 1.00
Test of group differences: Q_b(2) = 0.14, p = 0.93
1/2 1 2
Random-effects REML model

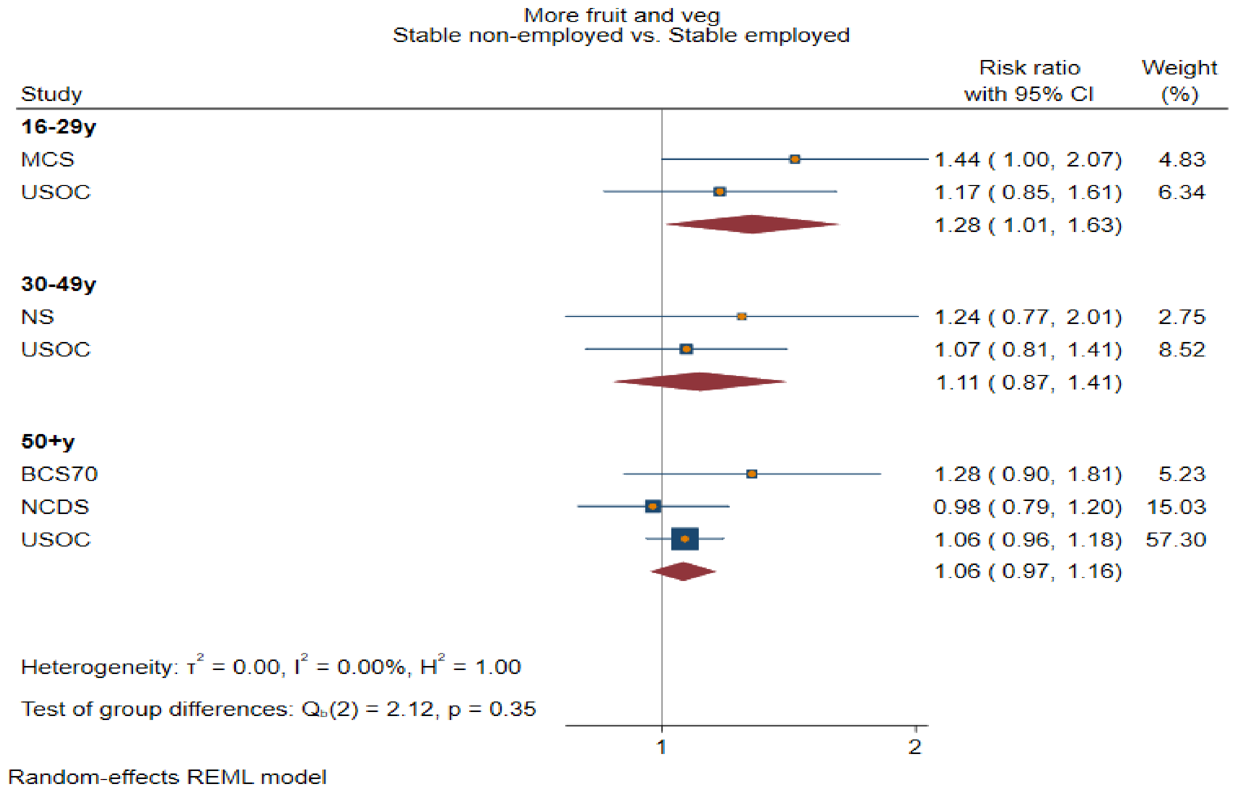
More fruit and veg
Stable non-employed vs. Stable employed
Study
Risk ratio with 95% CI
Weight (%)
16-29y
MCS 1.44 (1.00, 2.07) 4.83
USOC 1.17 (0.85, 1.61) 6.34
1.28 (1.01, 1.63)
30-49y
NS 1.24 (0.77, 2.01) 2.75
USOC 1.07 (0.81, 1.41) 8.52
1.11 (0.87, 1.41)
50+y
BCS70 1.28 (0.90, 1.81) 5.23
NCDS 0.98 (0.79, 1.20) 15.03
USOC 1.06 (0.96, 1.18) 57.30
1.06 (0.97, 1.16)
Heterogeneity: τ² = 0.00, I² = 0.00%, H² = 1.00
Test of group differences: Q_b(2) = 2.12, p = 0.35
1 2
Random-effects REML model

Figure set 4: Less than 3 days a week of at least 30min exercise

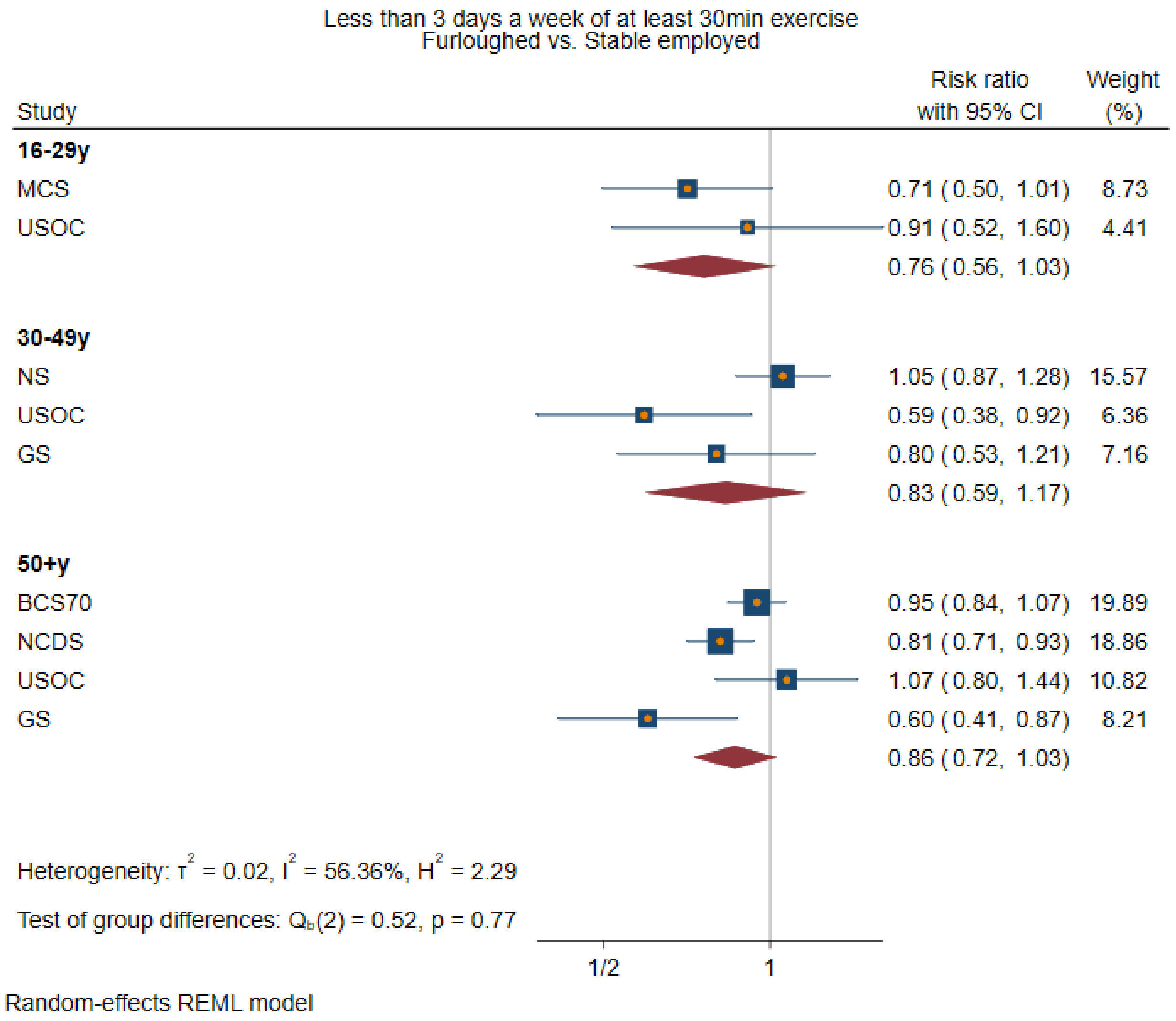

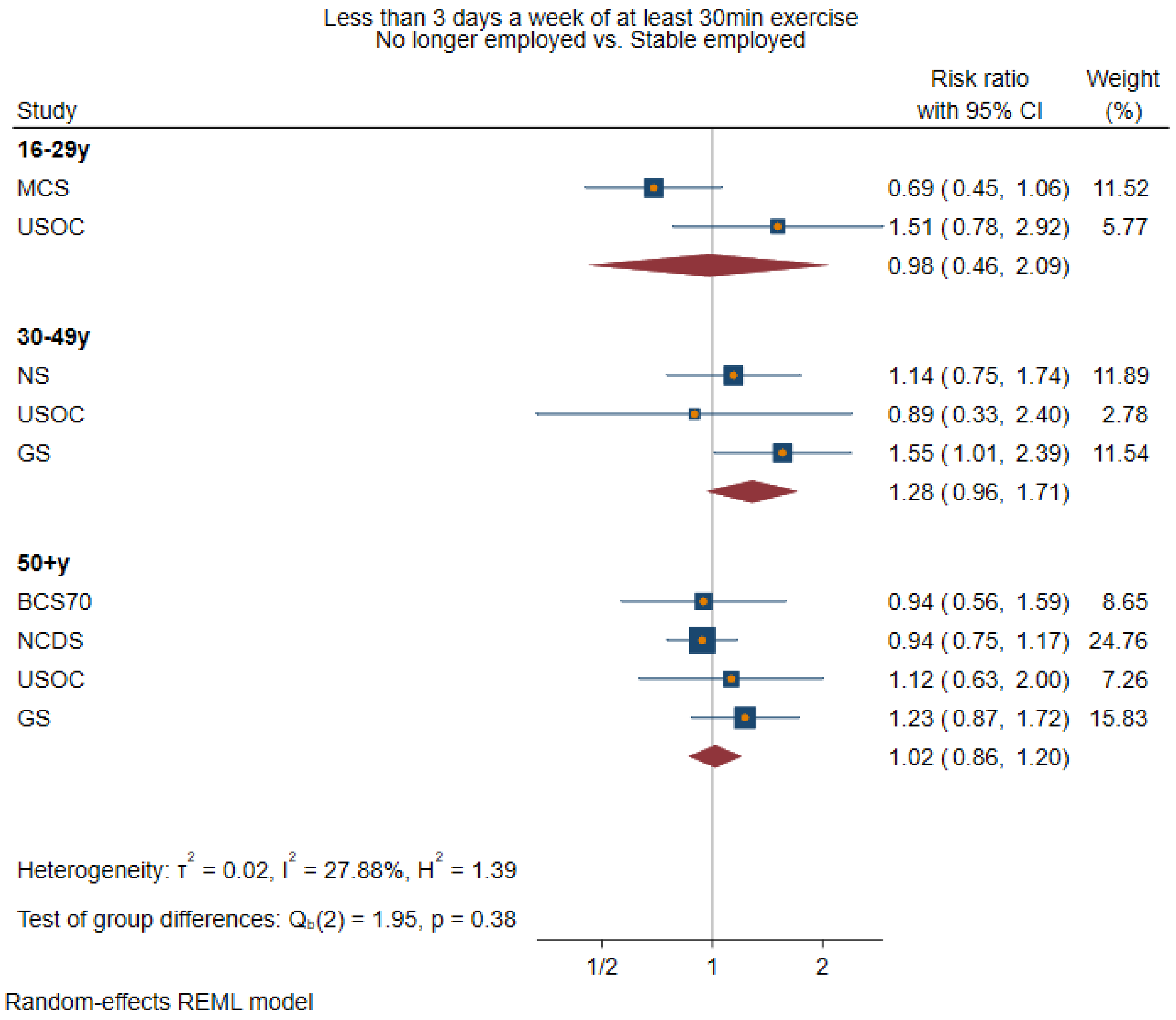

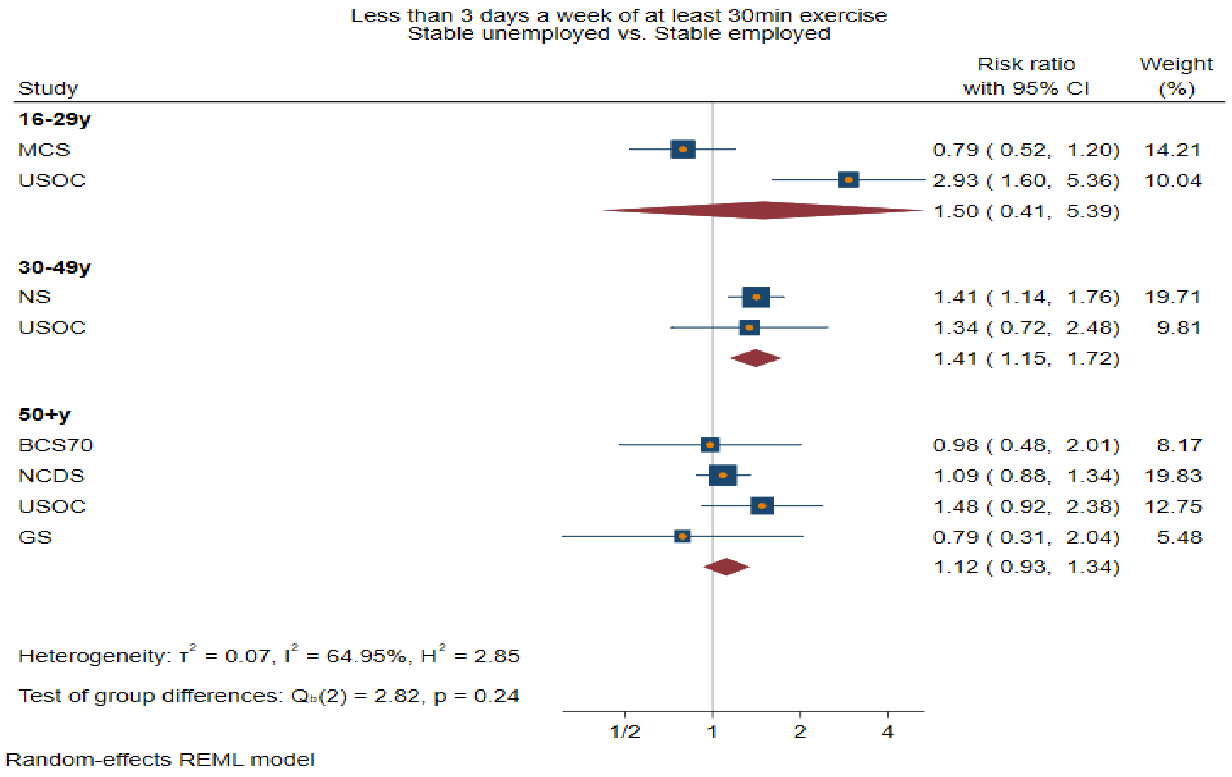
Less than 3 days a week of at least 30min exercise
Stable unemployed vs. Stable employed
Study
Risk ratio with 95% CI
Weight (%)
16-29y
MCS 0.79 (0.52, 1.20) 14.21
USOC 2.93 (1.60, 5.36) 10.04
1.50 (0.41, 5.39)
30-49y
NS 1.41 (1.14, 1.76) 19.71
USOC 1.34 (0.72, 2.48) 9.81
1.41 (1.15, 1.72)
50+y
BCS70 0.98 (0.48, 2.01) 8.17
NCDS 1.09 (0.88, 1.34) 19.83
USOC 1.48 (0.92, 2.38) 12.75
GS 0.79 (0.31, 2.04) 5.48
1.12 (0.93, 1.34)
Heterogeneity: τ² = 0.07, I² = 64.95%, H² = 2.85
Test of group differences: Qb(2) = 2.82, p = 0.24
1/2
1
2
4
Random-effects REML model

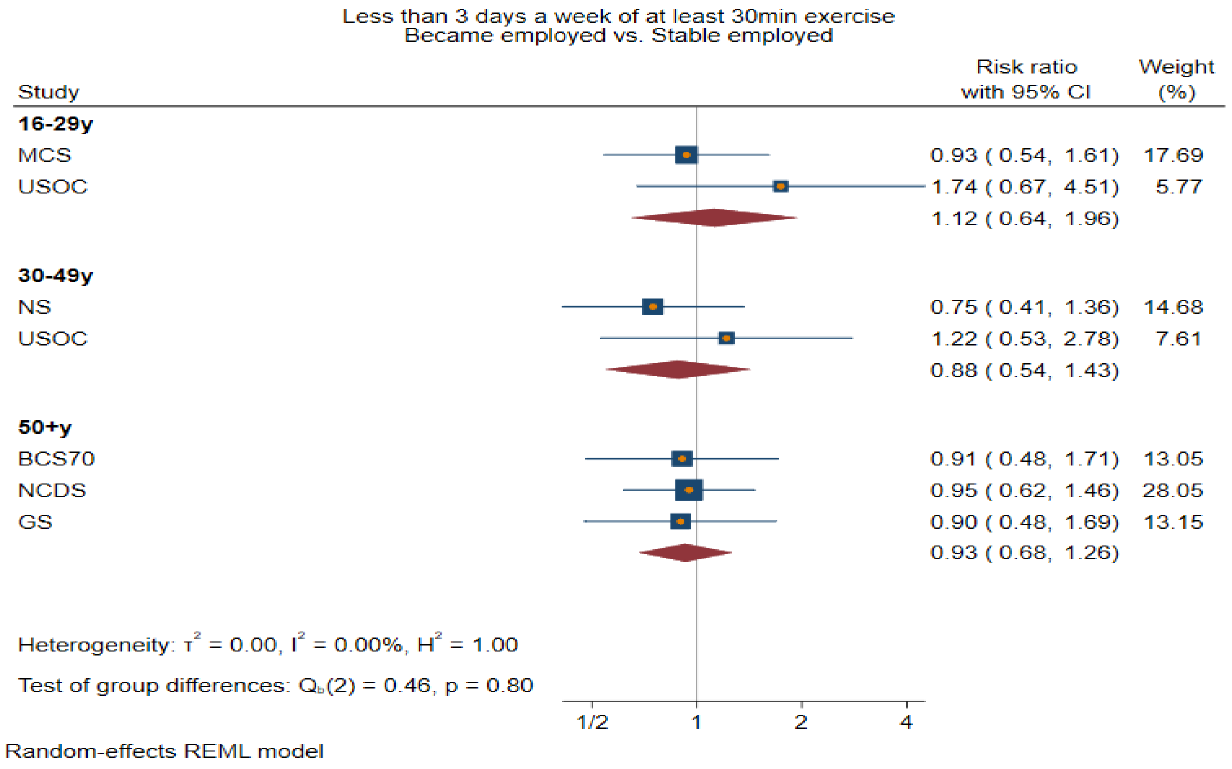
Less than 3 days a week of at least 30min exercise
Became employed vs. Stable employed
Study
Risk ratio with 95% CI
Weight (%)
16-29y
MCS 0.93 (0.54, 1.61) 17.69
USOC 1.74 (0.67, 4.51) 5.77
1.12 (0.64, 1.96)
30-49y
NS 0.75 (0.41, 1.36) 14.68
USOC 1.22 (0.53, 2.78) 7.61
0.88 (0.54, 1.43)
50+y
BCS70 0.91 (0.48, 1.71) 13.05
NCDS 0.95 (0.62, 1.46) 28.05
GS 0.90 (0.48, 1.69) 13.15
0.93 (0.68, 1.26)
Heterogeneity: τ² = 0.00, I² = 0.00%, H² = 1.00
Test of group differences: Qb(2) = 0.46, p = 0.80
1/2
1
2
4
Random-effects REML model

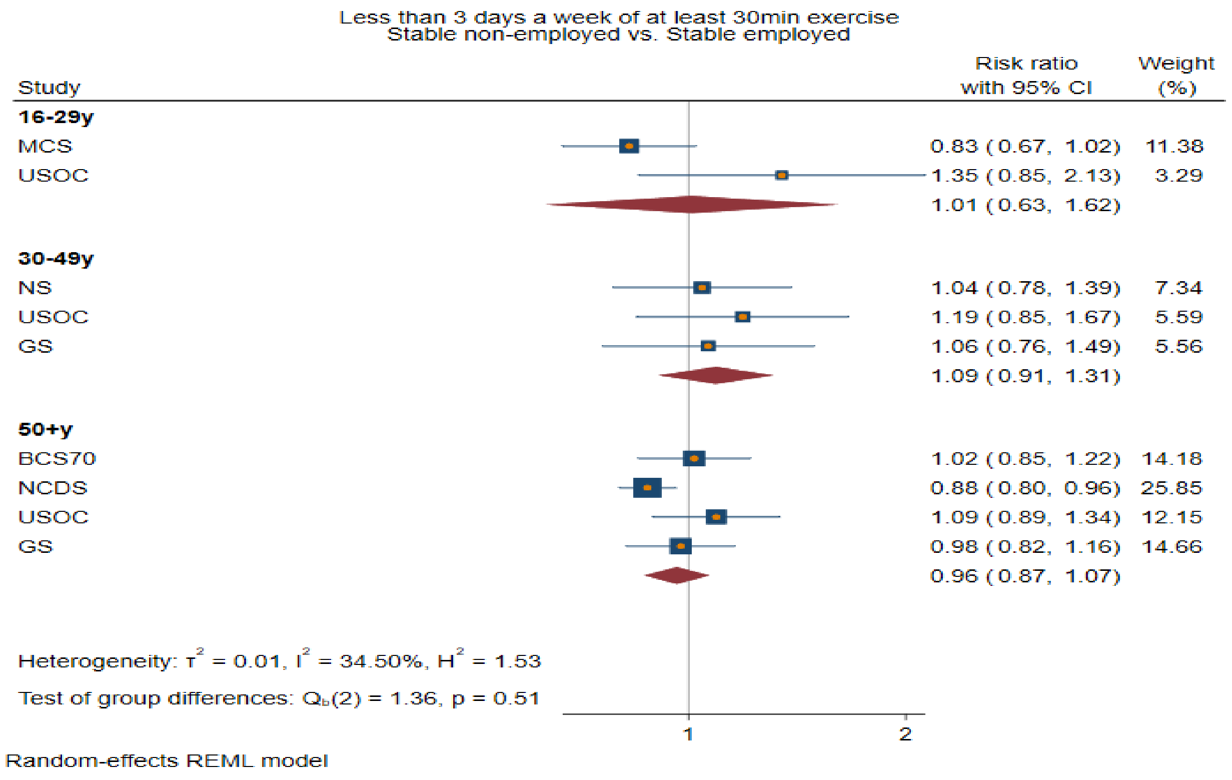
Less than 3 days a week of at least 30min exercise
Stable non-employed vs. Stable employed
Study
Risk ratio with 95% CI
Weight (%)
16-29y
MCS 0.83 (0.67, 1.02) 11.38
USOC 1.35 (0.85, 2.13) 3.29
1.01 (0.63, 1.62)
30-49y
NS 1.04 (0.78, 1.39) 7.34
USOC 1.19 (0.85, 1.67) 5.59
GS 1.06 (0.76, 1.49) 5.56
1.09 (0.91, 1.31)
50+y
BCS70 1.02 (0.85, 1.22) 14.18
NCDS 0.88 (0.80, 0.96) 25.85
USOC 1.09 (0.89, 1.34) 12.15
GS 0.98 (0.82, 1.16) 14.66
0.96 (0.87, 1.07)
Heterogeneity: τ² = 0.01, I² = 34.50%, H² = 1.53
Test of group differences: Qb(2) = 1.36, p = 0.51
1
2
Random-effects REML model

Figure set 5: Less time/ fewer days of physical exercise

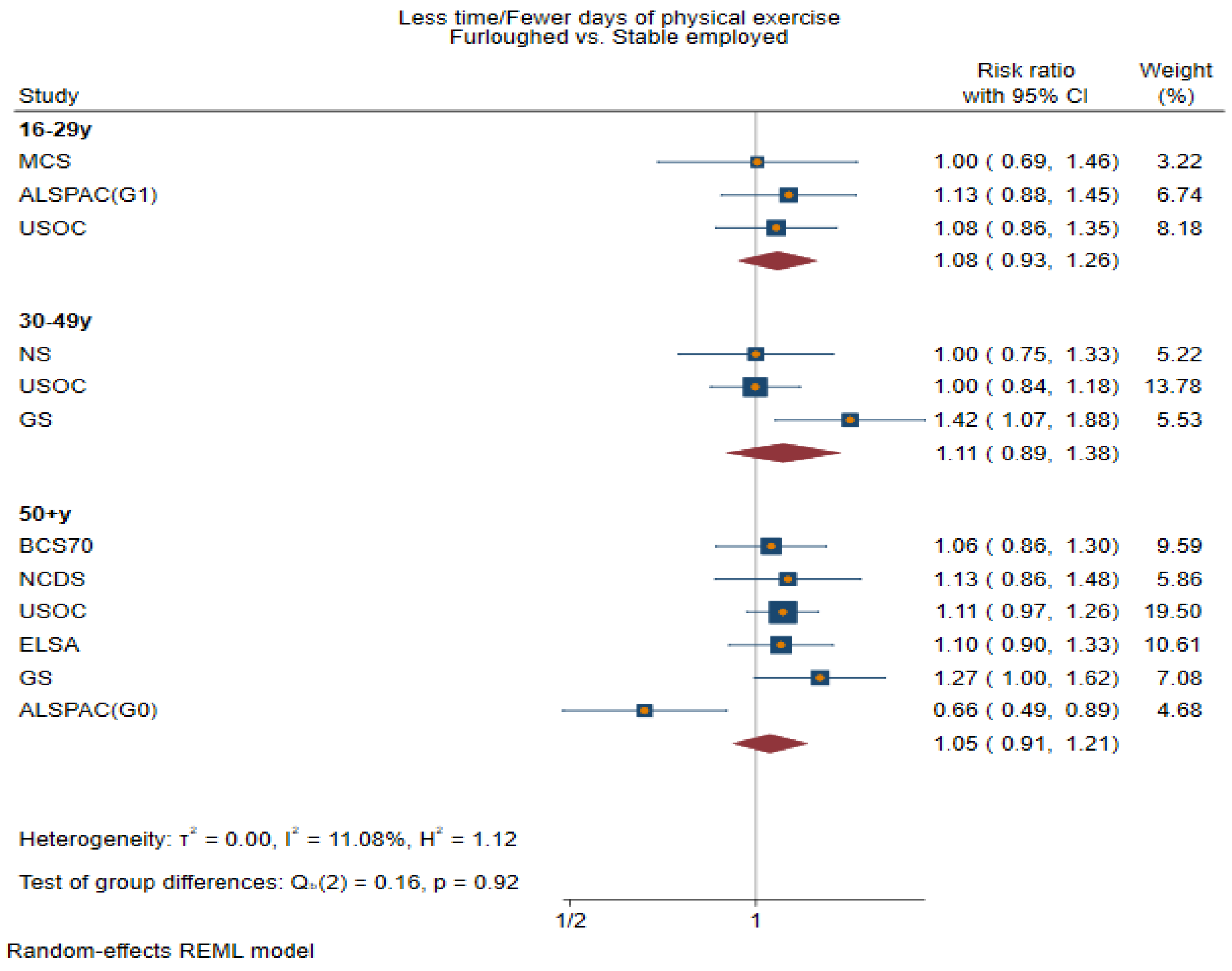

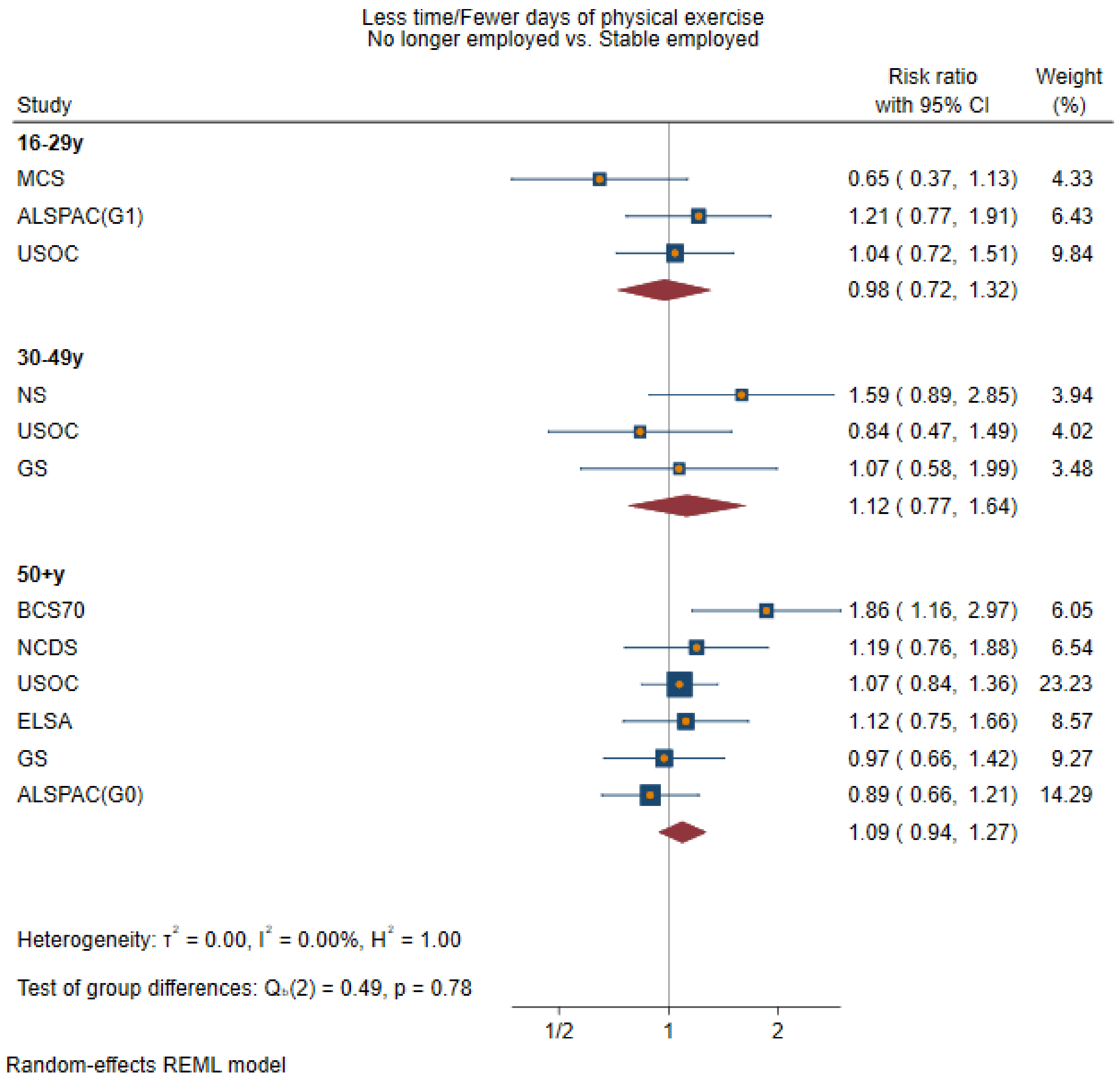

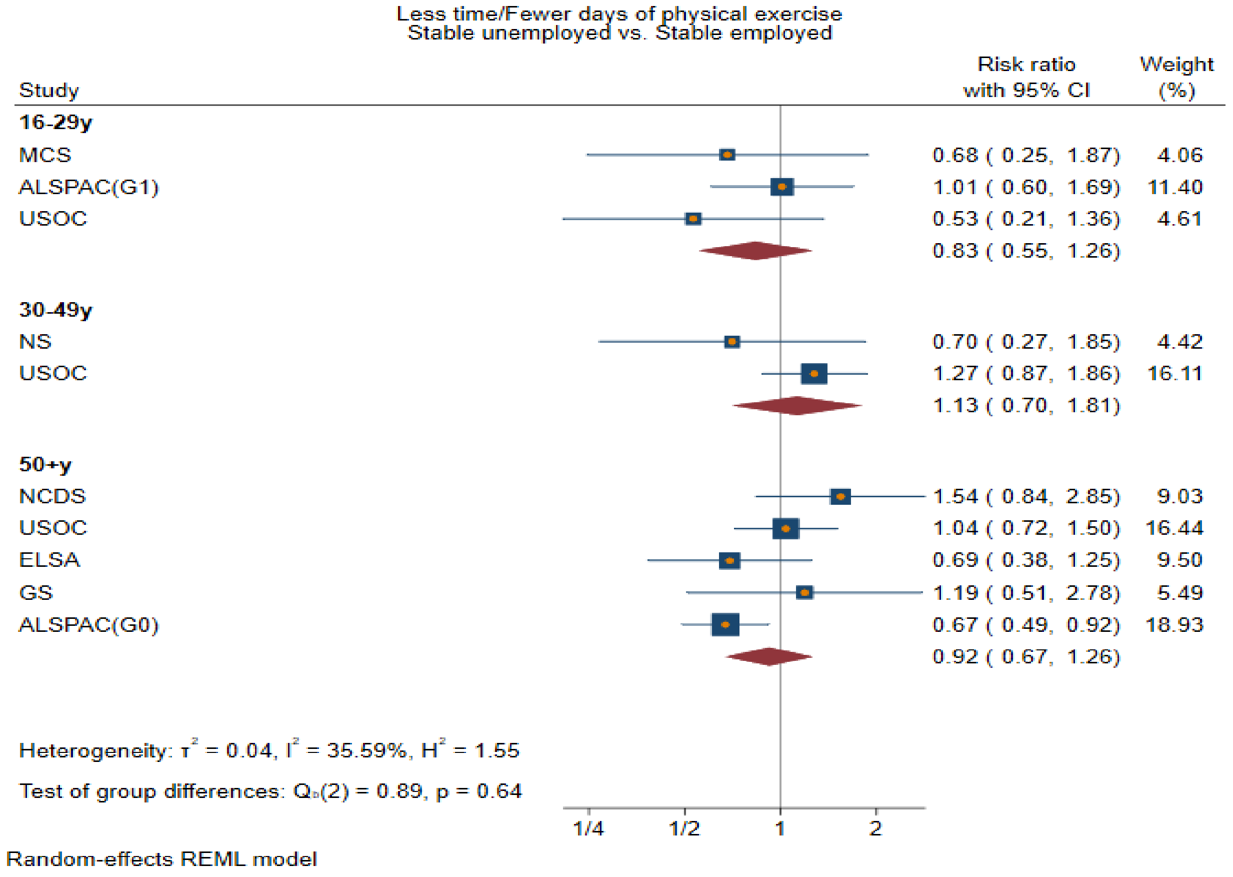
Less time/Fewer days of physical exercise
Stable unemployed vs. Stable employed
Study
Risk ratio with 95% CI
Weight (%)
16-29y
MCS 0.68 (0.25, 1.87) 4.06
ALSPAC(G1) 1.01 (0.60, 1.69) 11.40
USOC 0.53 (0.21, 1.36) 4.61
0.83 (0.55, 1.26)
30-49y
NS 0.70 (0.27, 1.85) 4.42
USOC 1.27 (0.87, 1.86) 16.11
1.13 (0.70, 1.81)
50+y
NCDS 1.54 (0.84, 2.85) 9.03
USOC 1.04 (0.72, 1.50) 16.44
ELSA 0.69 (0.38, 1.25) 9.50
GS 1.19 (0.51, 2.78) 5.49
ALSPAC(G0) 0.67 (0.49, 0.92) 18.93
0.92 (0.67, 1.26)
Heterogeneity: τ^2 = 0.04, I^2 = 35.59%, H^2 = 1.55
Test of group differences: $Q_b(2)$ = 0.89, p = 0.64
1/4
1/2
1
2
Random-effects REML model

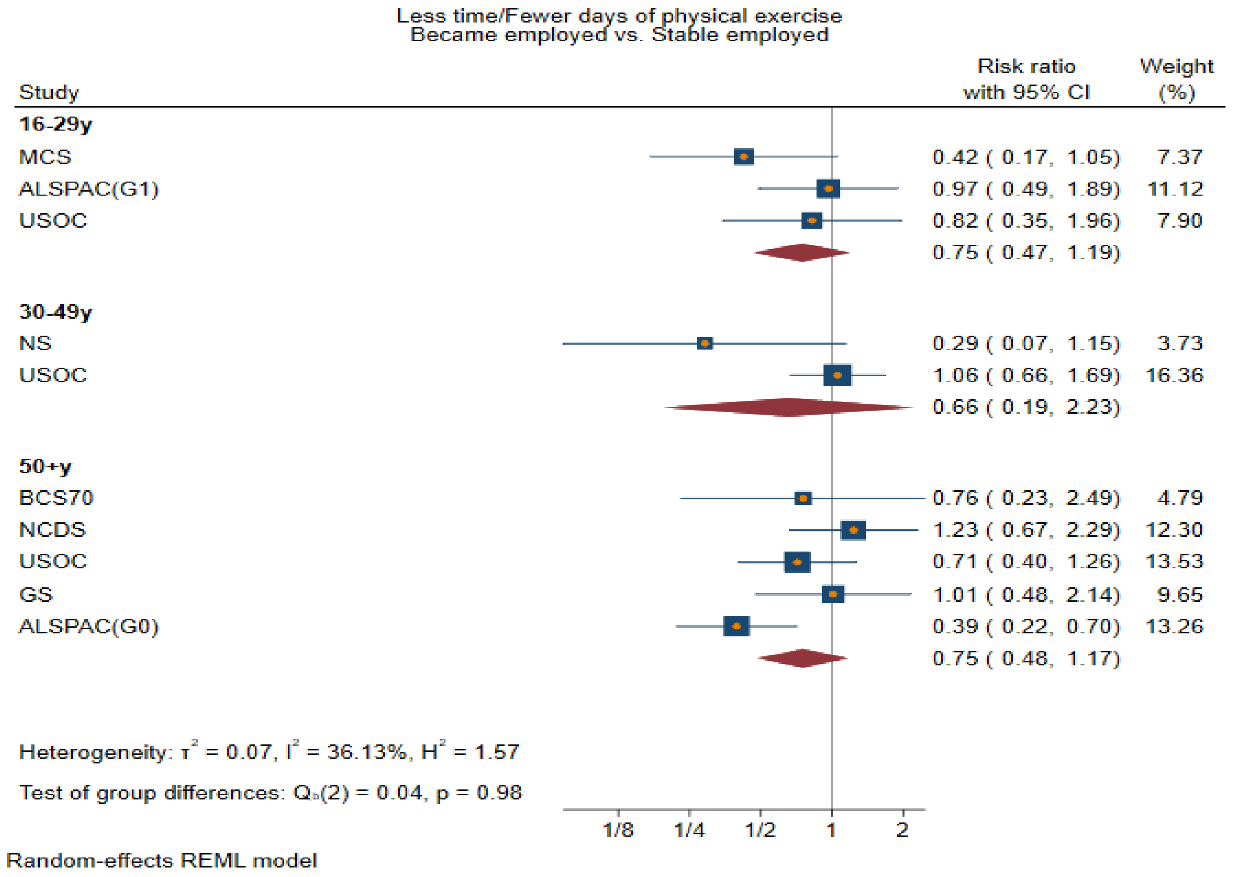
Less time/Fewer days of physical exercise
Became employed vs. Stable employed
Study
Risk ratio with 95% CI
Weight (%)
16-29y
MCS 0.42 (0.17, 1.05) 7.37
ALSPAC(G1) 0.97 (0.49, 1.89) 11.12
USOC 0.82 (0.35, 1.96) 7.90
0.75 (0.47, 1.19)
30-49y
NS 0.29 (0.07, 1.15) 3.73
USOC 1.06 (0.66, 1.69) 16.36
0.66 (0.19, 2.23)
50+y
BCS70 0.76 (0.23, 2.49) 4.79
NCDS 1.23 (0.67, 2.29) 12.30
USOC 0.71 (0.40, 1.26) 13.53
GS 1.01 (0.48, 2.14) 9.65
ALSPAC(G0) 0.39 (0.22, 0.70) 13.26
0.75 (0.48, 1.17)
Heterogeneity: τ^2 = 0.07, I^2 = 36.13%, H^2 = 1.57
Test of group differences: $Q_b(2)$ = 0.04, p = 0.98
1/8
1/4
1/2
1
2
Random-effects REML model

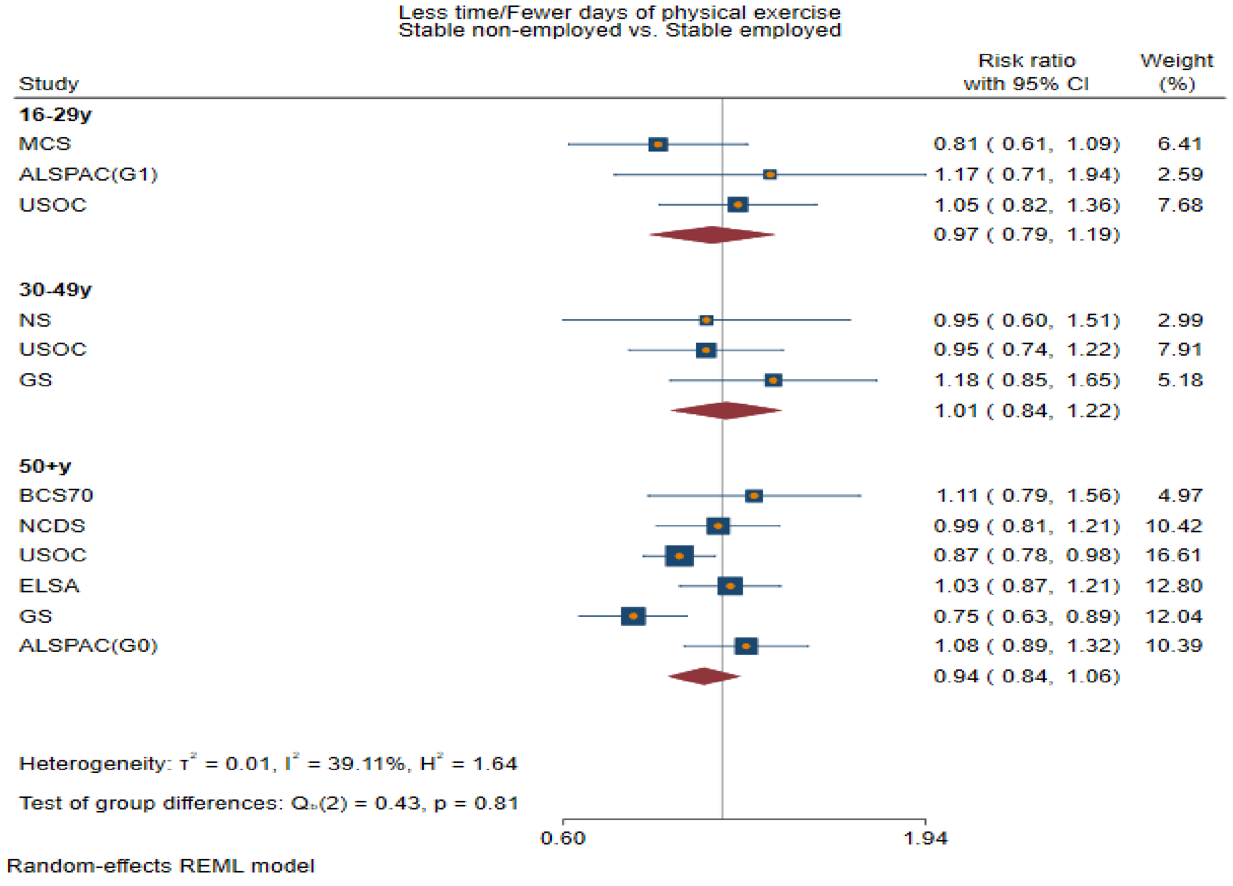
Less time/Fewer days of physical exercise
Stable non-employed vs. Stable employed
Study
Risk ratio with 95% CI
Weight (%)
16-29y
MCS 0.81 (0.61, 1.09) 6.41
ALSPAC(G1) 1.17 (0.71, 1.94) 2.59
USOC 1.05 (0.82, 1.36) 7.68
0.97 (0.79, 1.19)
30-49y
NS 0.95 (0.60, 1.51) 2.99
USOC 0.95 (0.74, 1.22) 7.91
GS 1.18 (0.85, 1.65) 5.18
1.01 (0.84, 1.22)
50+y
BCS70 1.11 (0.79, 1.56) 4.97
NCDS 0.99 (0.81, 1.21) 10.42
USOC 0.87 (0.78, 0.98) 16.61
ELSA 1.03 (0.87, 1.21) 12.80
GS 0.75 (0.63, 0.89) 12.04
ALSPAC(G0) 1.08 (0.89, 1.32) 10.39
0.94 (0.84, 1.06)
Heterogeneity: τ^2 = 0.01, I^2 = 39.11%, H^2 = 1.64
Test of group differences: $Q_b(2)$ = 0.43, p = 0.81
0.60
1.94
Random-effects REML model

Figure set 6: More time/ days of physical exercise

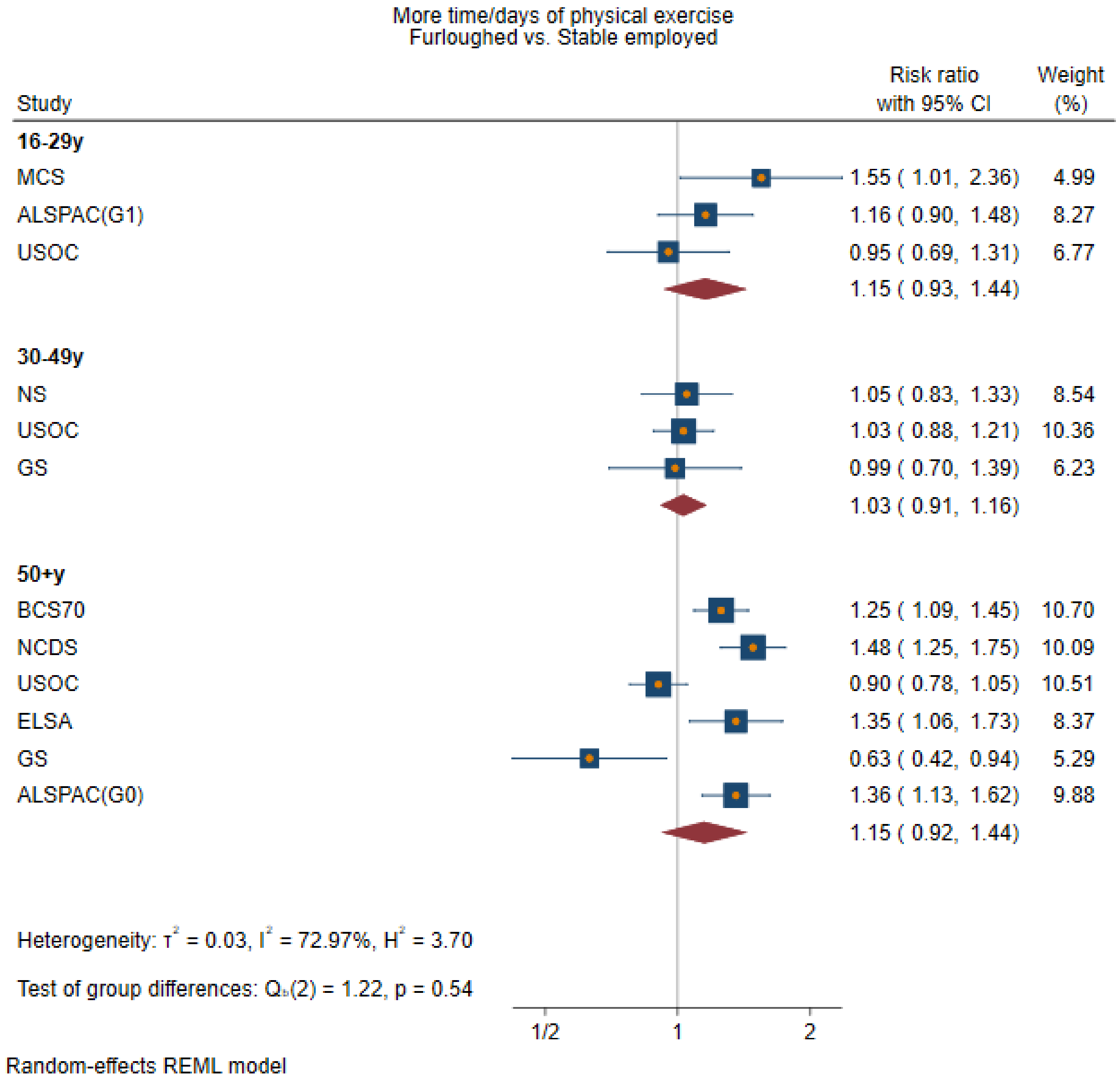

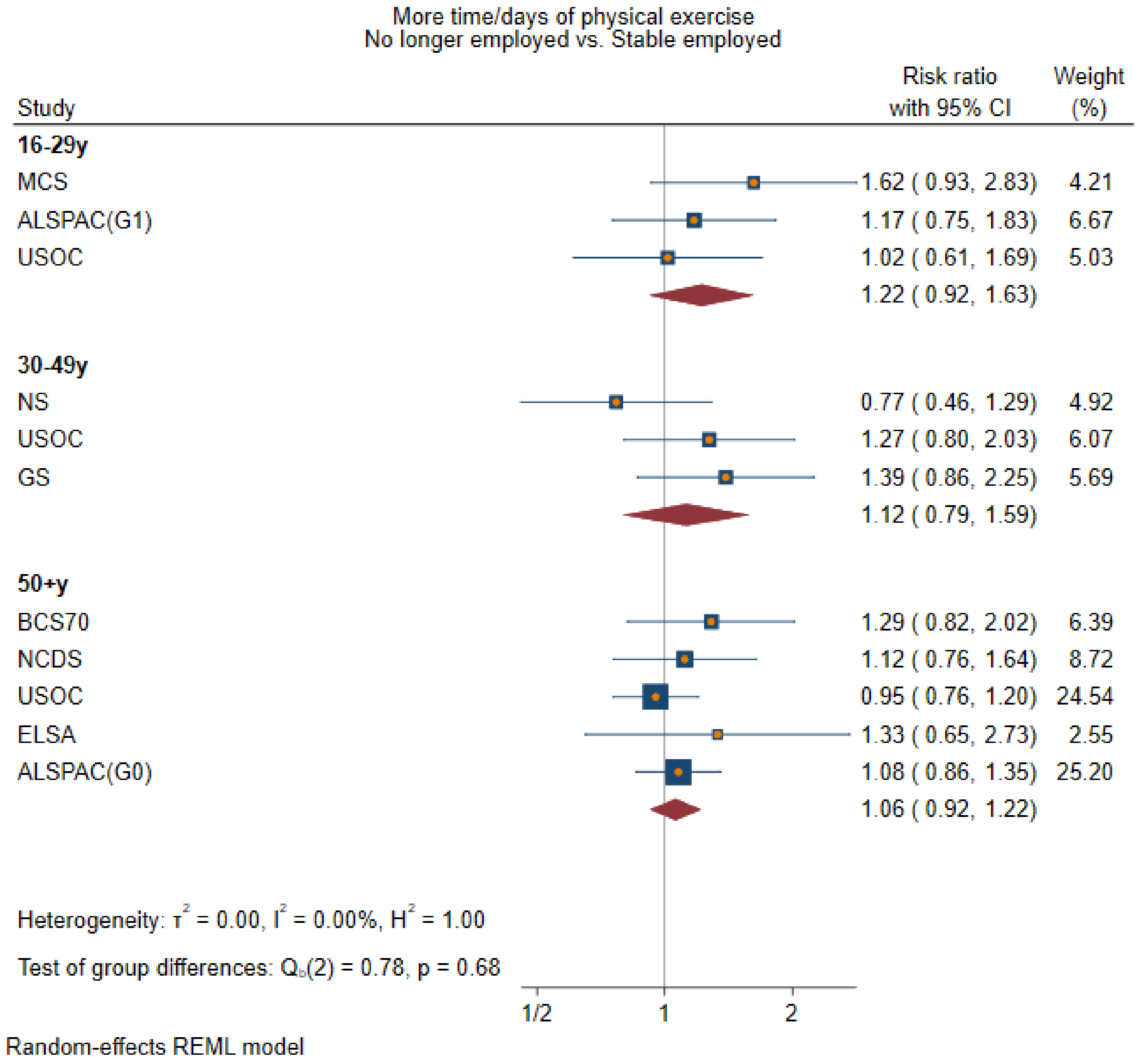

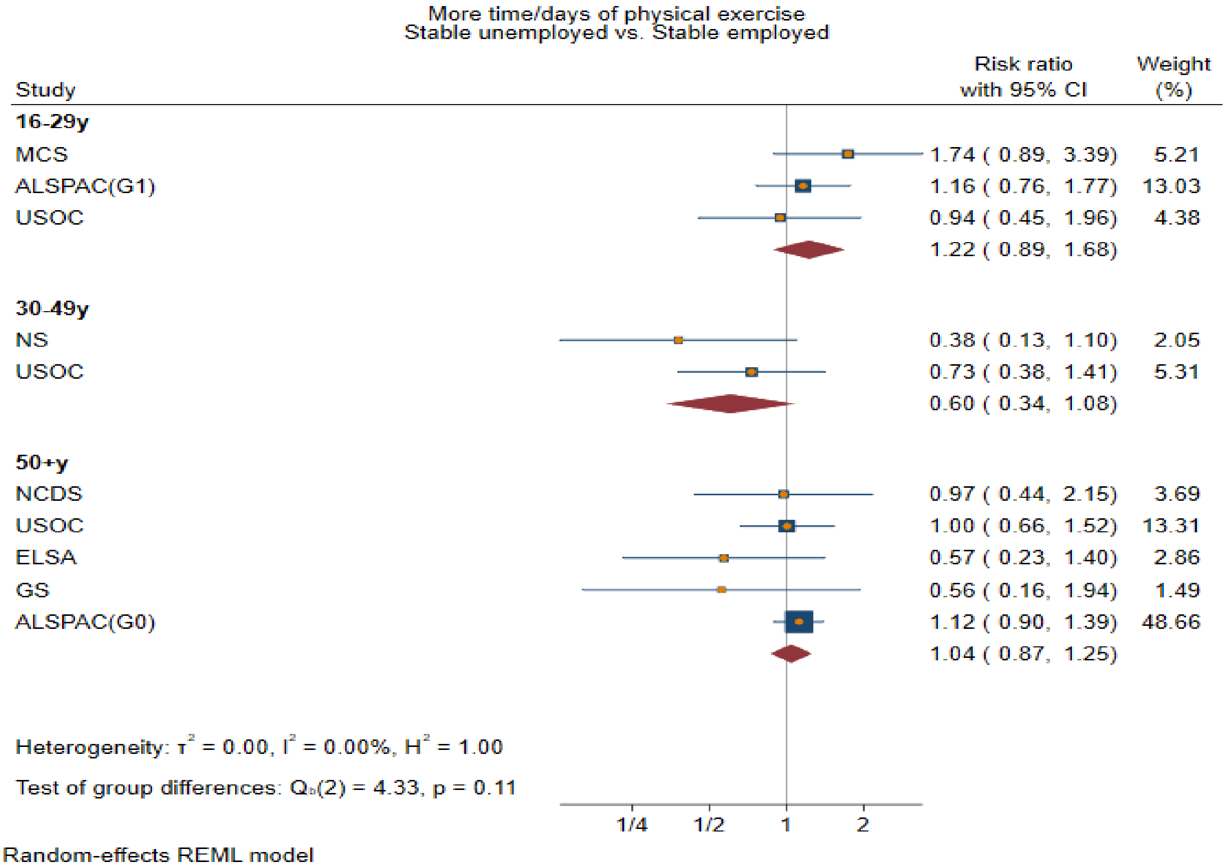
More time/days of physical exercise
Stable unemployed vs. Stable employed
Study
Risk ratio with 95% CI
Weight (%)
16-29y
MCS 1.74 (0.89, 3.39) 5.21
ALSPAC(G1) 1.16 (0.76, 1.77) 13.03
USOC 0.94 (0.45, 1.96) 4.38
1.22 (0.89, 1.68)
30-49y
NS 0.38 (0.13, 1.10) 2.05
USOC 0.73 (0.38, 1.41) 5.31
0.60 (0.34, 1.08)
50+y
NCDS 0.97 (0.44, 2.15) 3.69
USOC 1.00 (0.66, 1.52) 13.31
ELSA 0.57 (0.23, 1.40) 2.86
GS 0.56 (0.16, 1.94) 1.49
ALSPAC(G0) 1.12 (0.90, 1.39) 48.66
1.04 (0.87, 1.25)
Heterogeneity: τ² = 0.00, I² = 0.00%, H² = 1.00
Test of group differences: $Q_b(2)$ = 4.33, p = 0.11
1/4
1/2
1
2
Random-effects REML model

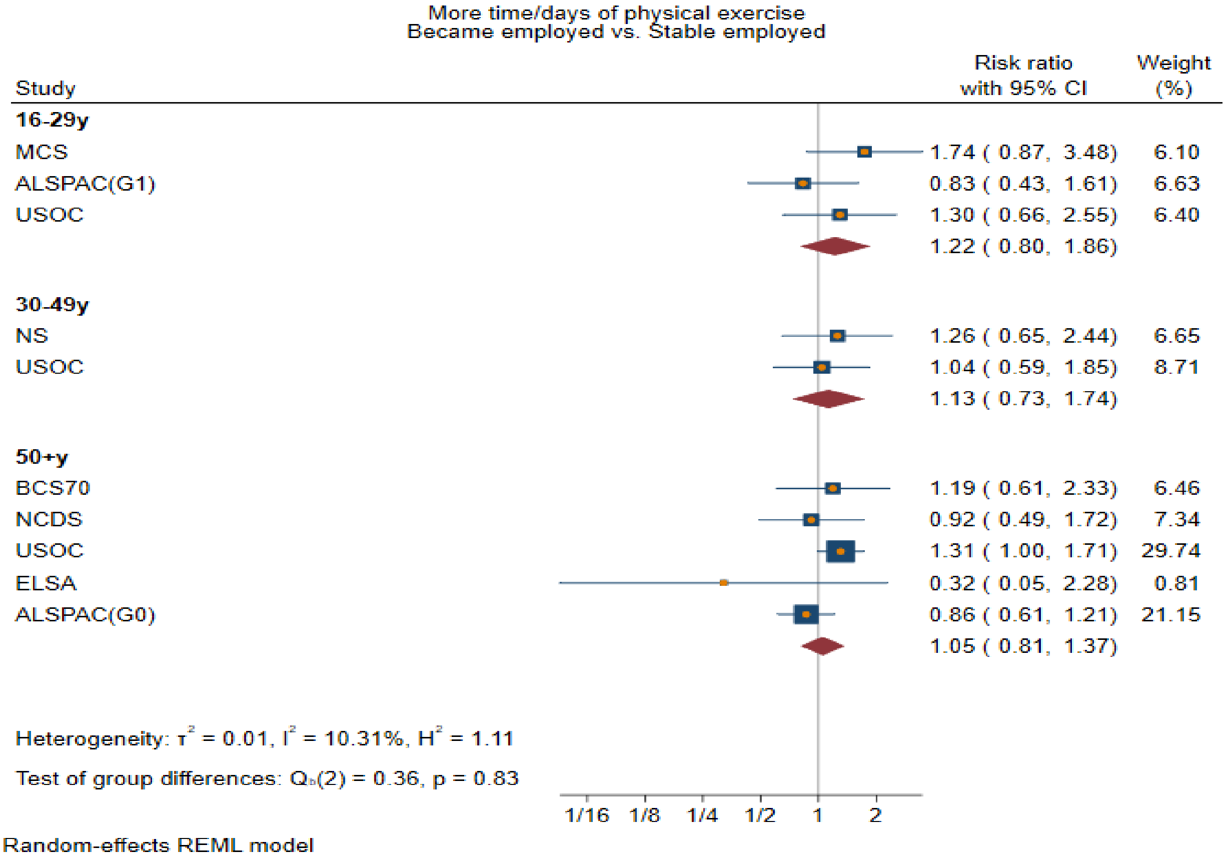
More time/days of physical exercise
Became employed vs. Stable employed
Study
Risk ratio with 95% CI
Weight (%)
16-29y
MCS 1.74 (0.87, 3.48) 6.10
ALSPAC(G1) 0.83 (0.43, 1.61) 6.63
USOC 1.30 (0.66, 2.55) 6.40
1.22 (0.80, 1.86)
30-49y
NS 1.26 (0.65, 2.44) 6.65
USOC 1.04 (0.59, 1.85) 8.71
1.13 (0.73, 1.74)
50+y
BCS70 1.19 (0.61, 2.33) 6.46
NCDS 0.92 (0.49, 1.72) 7.34
USOC 1.31 (1.00, 1.71) 29.74
ELSA 0.32 (0.05, 2.28) 0.81
ALSPAC(G0) 0.86 (0.61, 1.21) 21.15
1.05 (0.81, 1.37)
Heterogeneity: τ² = 0.01, I² = 10.31%, H² = 1.11
Test of group differences: $Q_b(2)$ = 0.36, p = 0.83
1/16
1/8
1/4
1/2
1
2
Random-effects REML model

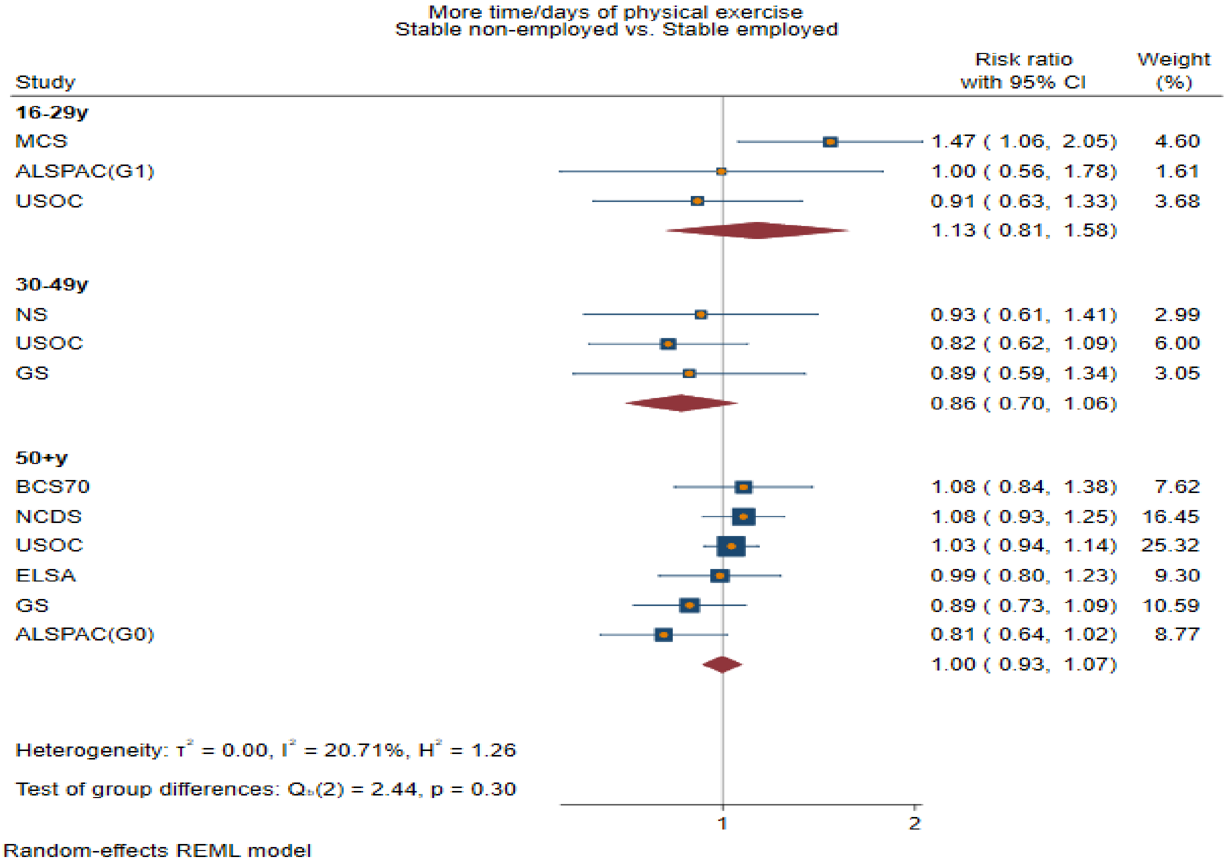
More time/days of physical exercise
Stable non-employed vs. Stable employed
Study
Risk ratio with 95% CI
Weight (%)
16-29y
MCS 1.47 (1.06, 2.05) 4.60
ALSPAC(G1) 1.00 (0.56, 1.78) 1.61
USOC 0.91 (0.63, 1.33) 3.68
1.13 (0.81, 1.58)
30-49y
NS 0.93 (0.61, 1.41) 2.99
USOC 0.82 (0.62, 1.09) 6.00
GS 0.89 (0.59, 1.34) 3.05
0.86 (0.70, 1.06)
50+y
BCS70 1.08 (0.84, 1.38) 7.62
NCDS 1.08 (0.93, 1.25) 16.45
USOC 1.03 (0.94, 1.14) 25.32
ELSA 0.99 (0.80, 1.23) 9.30
GS 0.89 (0.73, 1.09) 10.59
ALSPAC(G0) 0.81 (0.64, 1.02) 8.77
1.00 (0.93, 1.07)
Heterogeneity: τ² = 0.00, I² = 20.71%, H² = 1.26
Test of group differences: $Q_b(2)$ = 2.44, p = 0.30
1
2
Random-effects REML model

Figure set 7: Sleeps outside ‘Normal Range’ (i.e. <6 or 9+ hours)

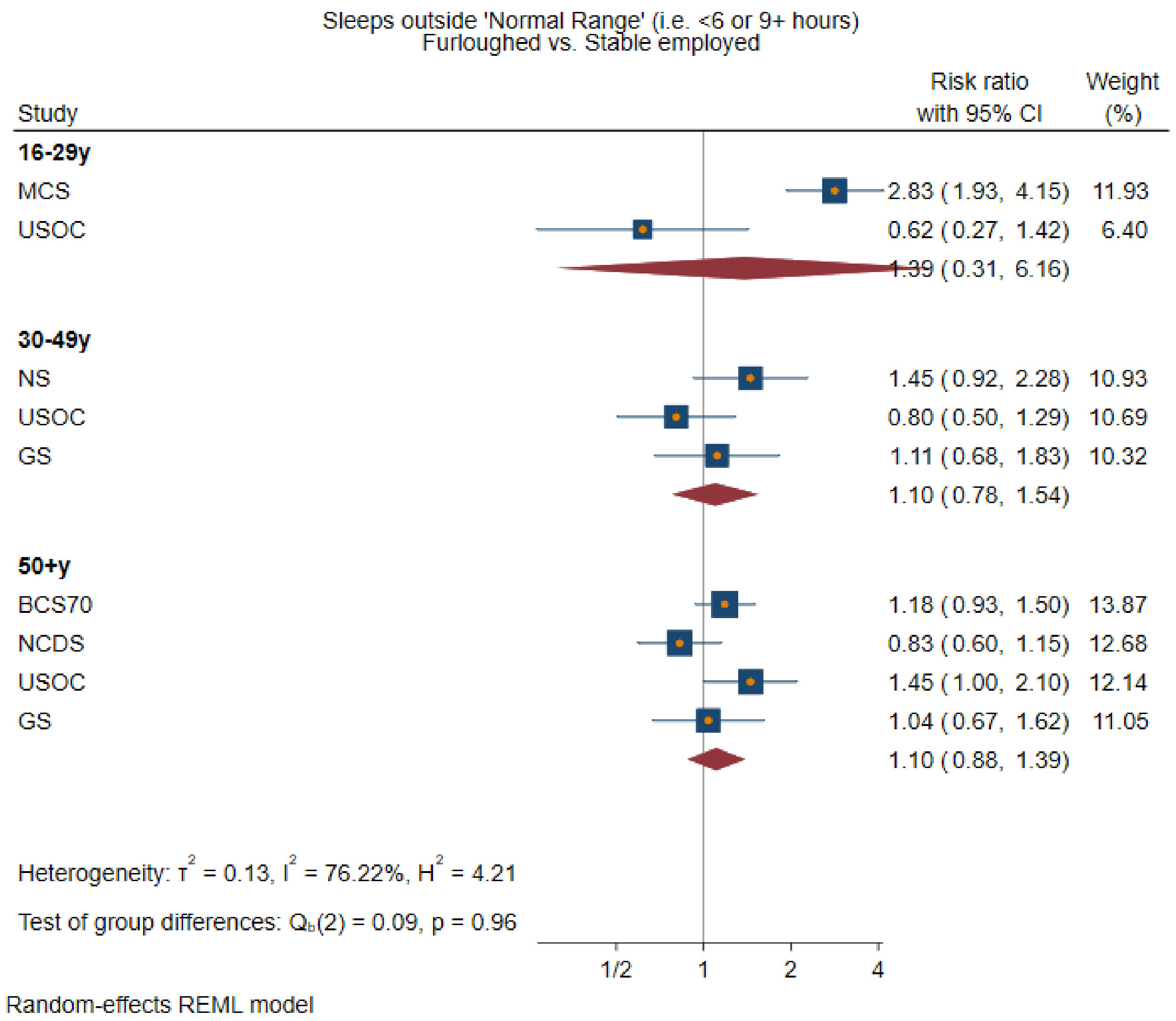

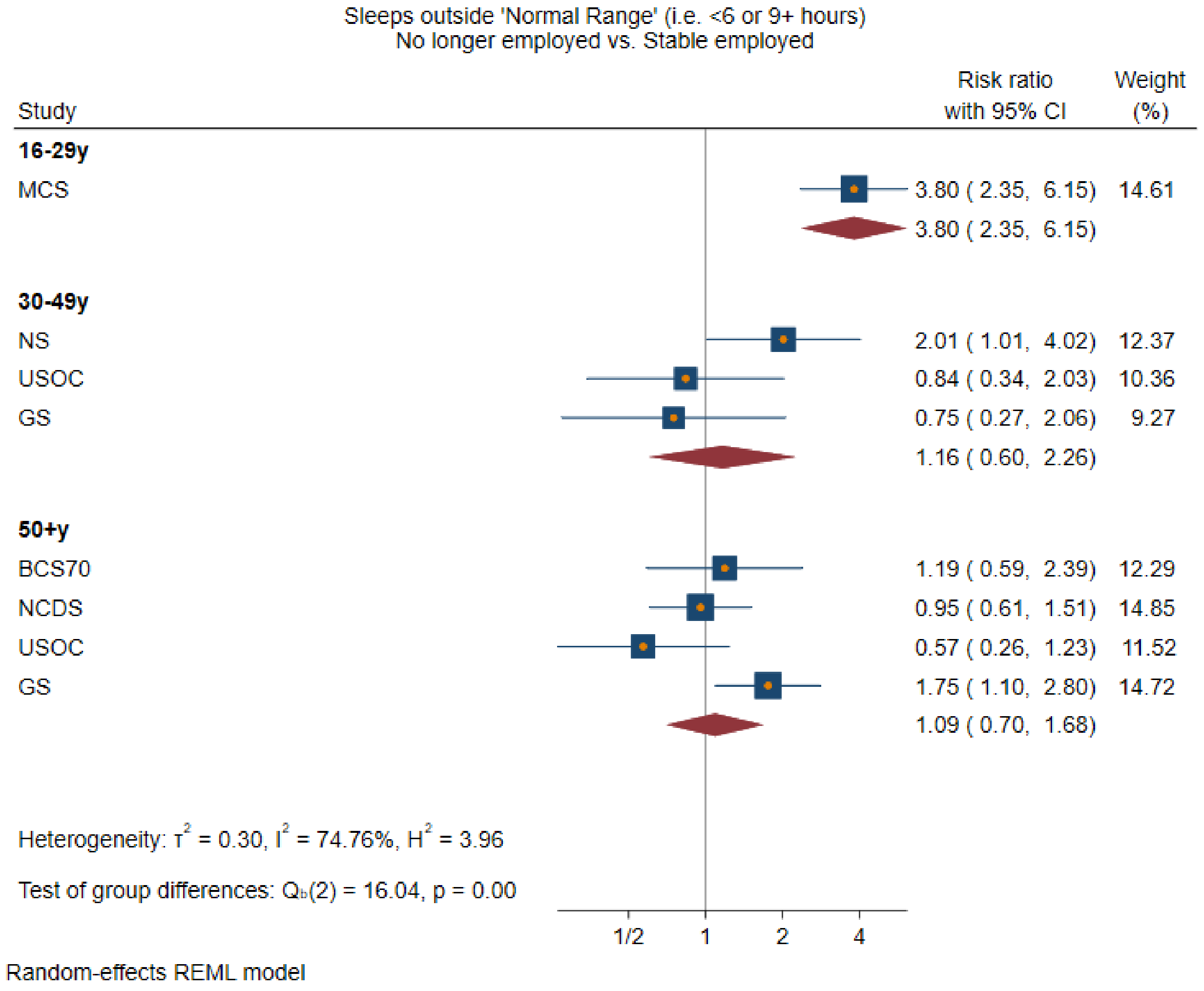

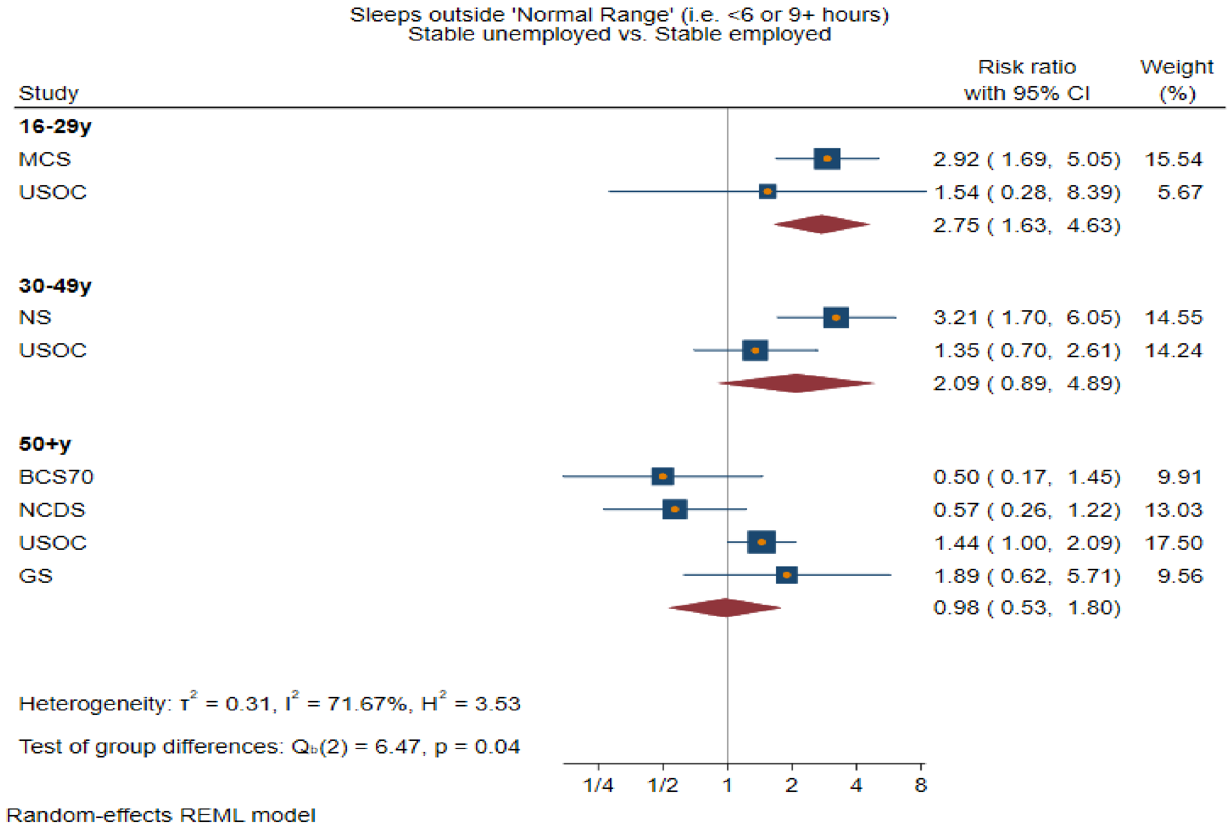
Sleeps outside 'Normal Range' (i.e. <6 or 9+ hours)
Stable unemployed vs. Stable employed
Study
Risk ratio with 95% CI
Weight (%)
16-29y
MCS 2.92 (1.69, 5.05) 15.54
USOC 1.54 (0.28, 8.39) 5.67
2.75 (1.63, 4.63)
30-49y
NS 3.21 (1.70, 6.05) 14.55
USOC 1.35 (0.70, 2.61) 14.24
2.09 (0.89, 4.89)
50+y
BCS70 0.50 (0.17, 1.45) 9.91
NCDS 0.57 (0.26, 1.22) 13.03
USOC 1.44 (1.00, 2.09) 17.50
GS 1.89 (0.62, 5.71) 9.56
0.98 (0.53, 1.80)
Heterogeneity: τ² = 0.31, I² = 71.67%, H² = 3.53
Test of group differences: Q_b(2) = 6.47, p = 0.04
1/4 1/2 1 2 4 8
Random-effects REML model

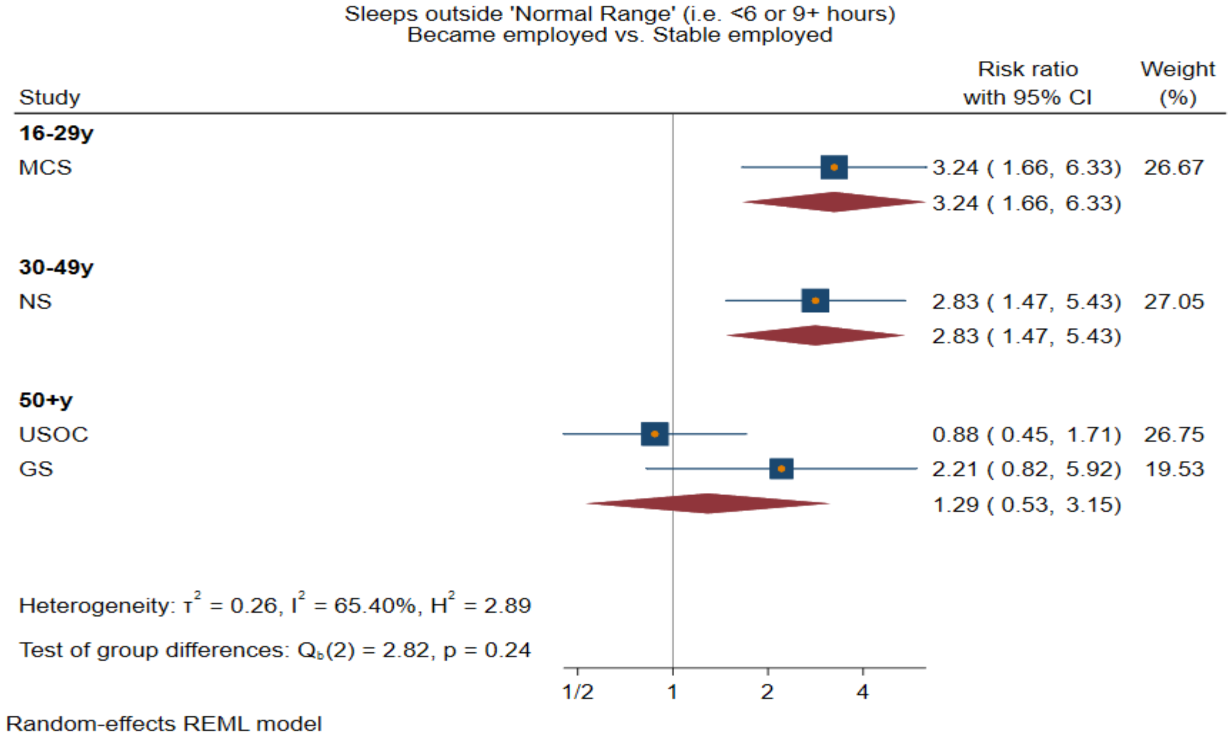
Sleeps outside 'Normal Range' (i.e. <6 or 9+ hours)
Became employed vs. Stable employed
Study
Risk ratio with 95% CI
Weight (%)
16-29y
MCS 3.24 (1.66, 6.33) 26.67
3.24 (1.66, 6.33)
30-49y
NS 2.83 (1.47, 5.43) 27.05
2.83 (1.47, 5.43)
50+y
USOC 0.88 (0.45, 1.71) 26.75
GS 2.21 (0.82, 5.92) 19.53
1.29 (0.53, 3.15)
Heterogeneity: τ² = 0.26, I² = 65.40%, H² = 2.89
Test of group differences: Q_b(2) = 2.82, p = 0.24
1/2 1 2 4
Random-effects REML model

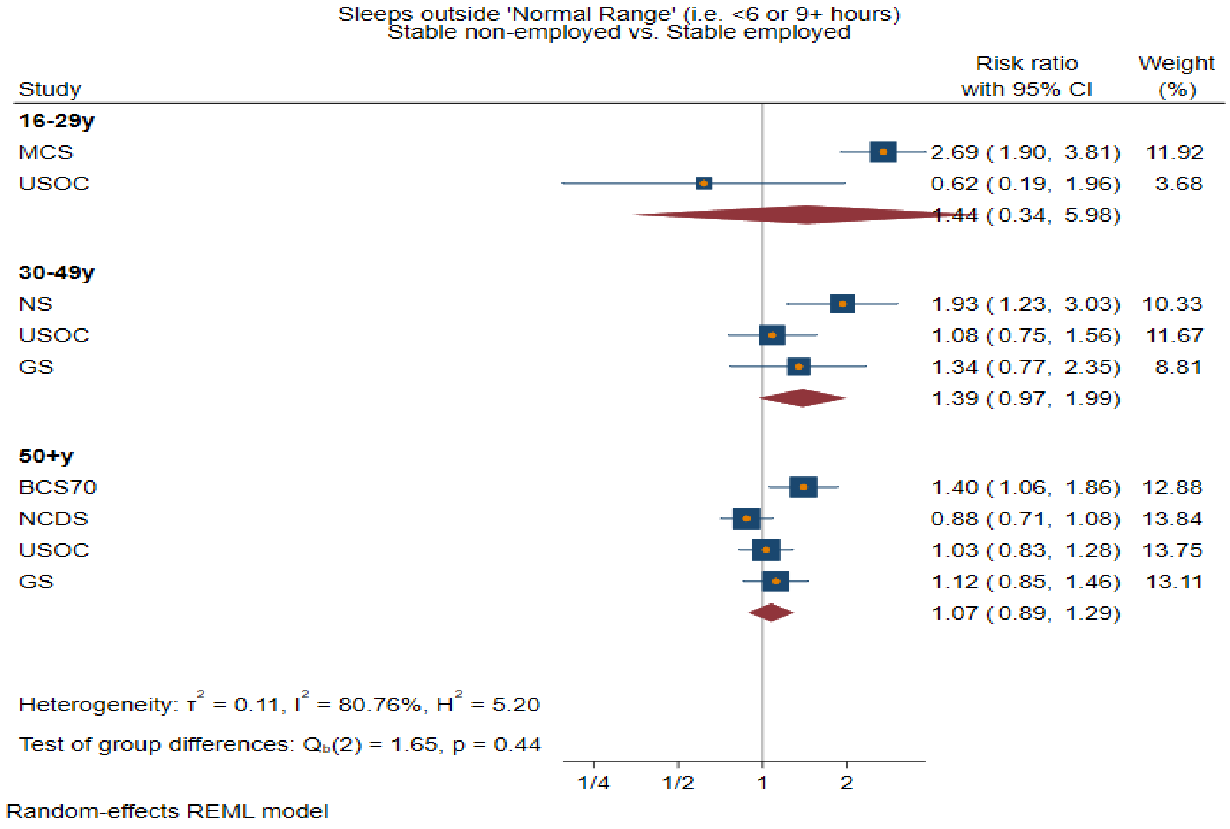
Sleeps outside 'Normal Range' (i.e. <6 or 9+ hours)
Stable non-employed vs. Stable employed
Study
Risk ratio with 95% CI
Weight (%)
16-29y
MCS 2.69 (1.90, 3.81) 11.92
USOC 0.62 (0.19, 1.96) 3.68
1.44 (0.34, 5.98)
30-49y
NS 1.93 (1.23, 3.03) 10.33
USOC 1.08 (0.75, 1.56) 11.67
GS 1.34 (0.77, 2.35) 8.81
1.39 (0.97, 1.99)
50+y
BCS70 1.40 (1.06, 1.86) 12.88
NCDS 0.88 (0.71, 1.08) 13.84
USOC 1.03 (0.83, 1.28) 13.75
GS 1.12 (0.85, 1.46) 13.11
1.07 (0.89, 1.29)
Heterogeneity: τ² = 0.11, I² = 80.76%, H² = 5.20
Test of group differences: Q_b(2) = 1.65, p = 0.44
1/4 1/2 1 2
Random-effects REML model

Figure set 8: Sleeps less than before

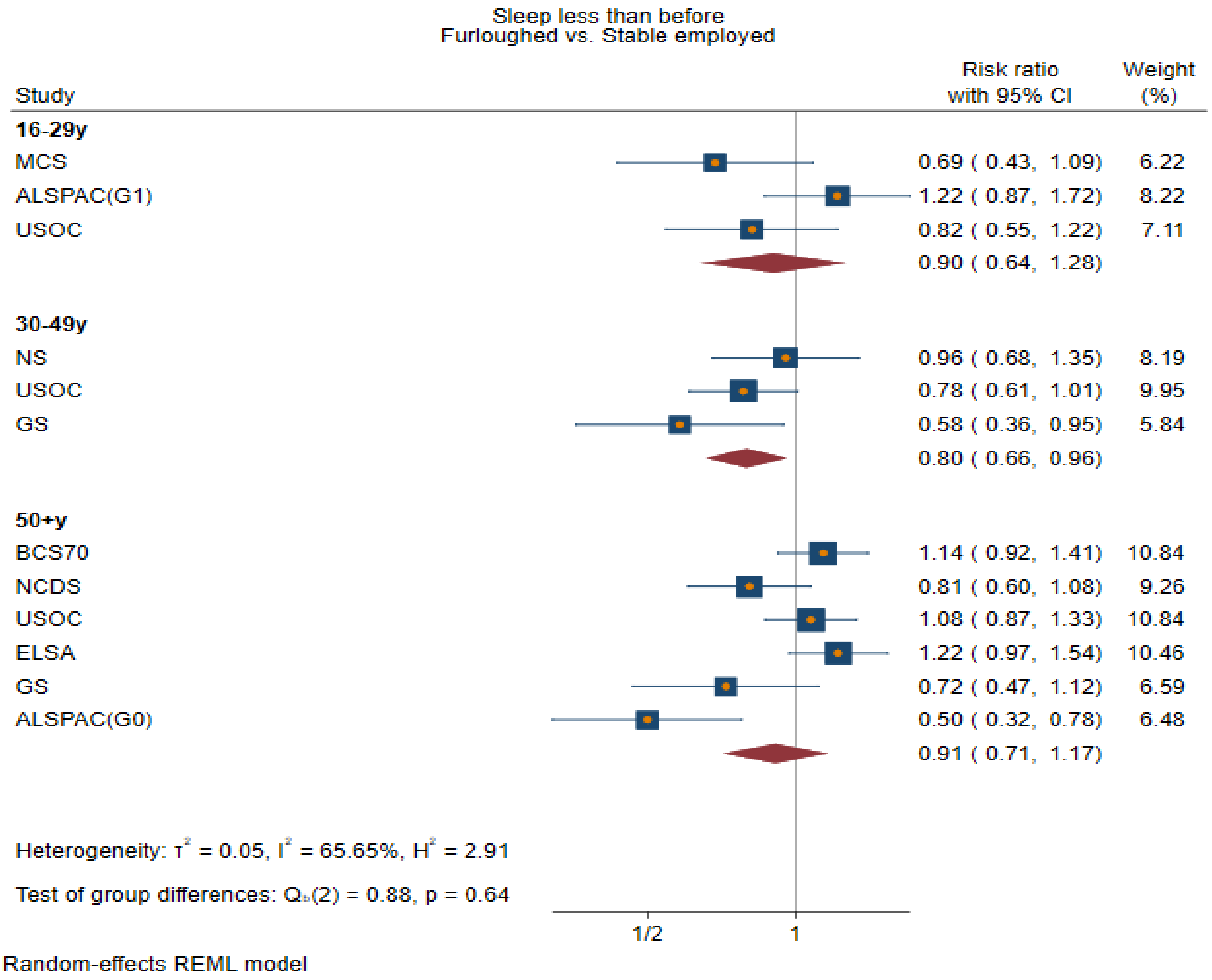

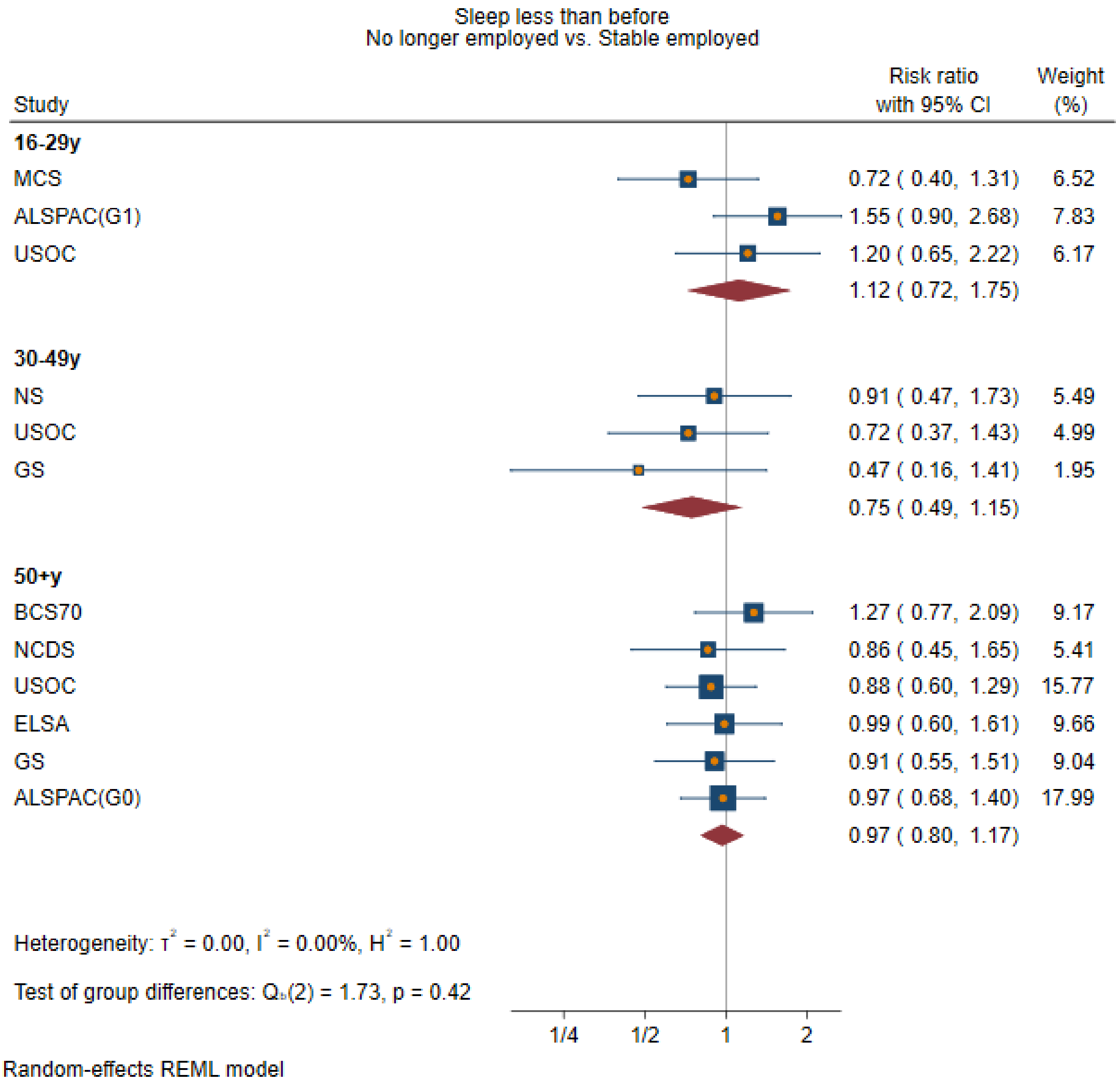

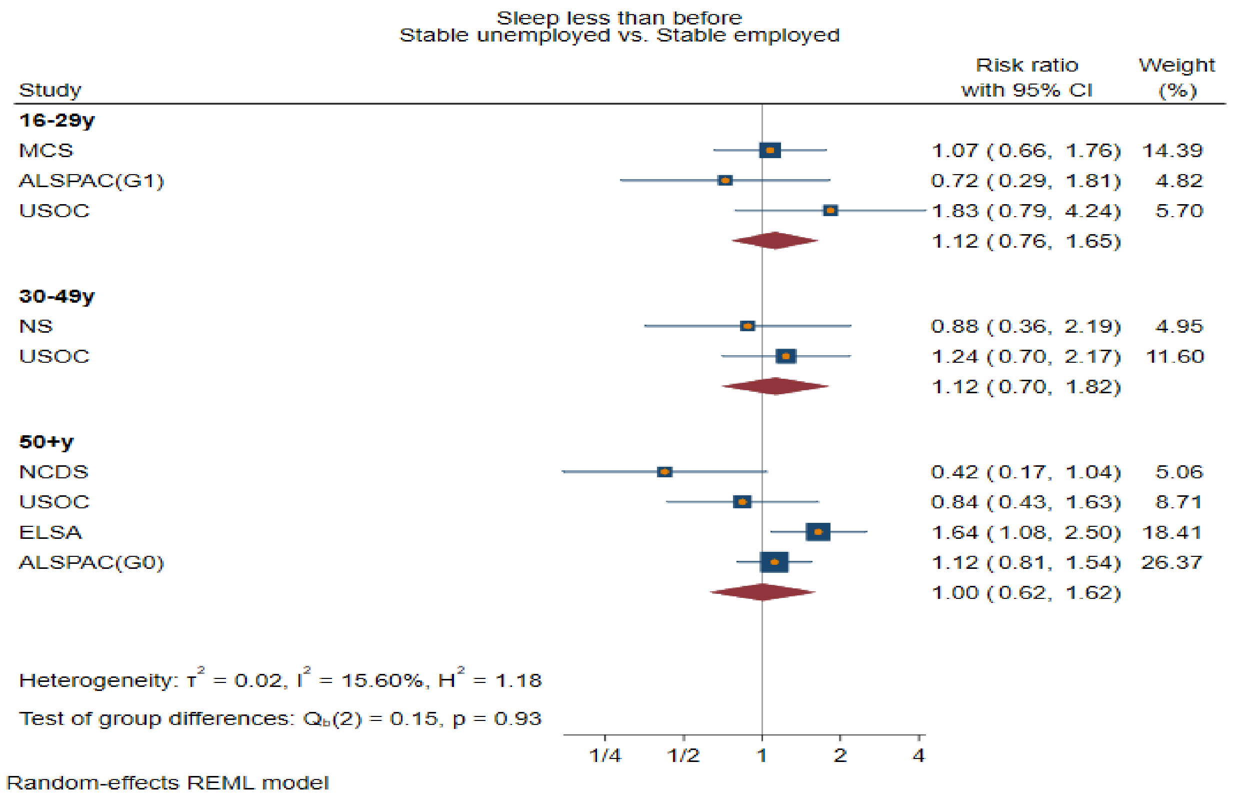
Sleep less than before
Stable unemployed vs. Stable employed
Study
Risk ratio with 95% CI
Weight (%)
16-29y
MCS 1.07 (0.66, 1.76) 14.39
ALSPAC(G1) 0.72 (0.29, 1.81) 4.82
USOC 1.83 (0.79, 4.24) 5.70
1.12 (0.76, 1.65)
30-49y
NS 0.88 (0.36, 2.19) 4.95
USOC 1.24 (0.70, 2.17) 11.60
1.12 (0.70, 1.82)
50+y
NCDS 0.42 (0.17, 1.04) 5.06
USOC 0.84 (0.43, 1.63) 8.71
ELSA 1.64 (1.08, 2.50) 18.41
ALSPAC(G0) 1.12 (0.81, 1.54) 26.37
1.00 (0.62, 1.62)
Heterogeneity: τ² = 0.02, I² = 15.60%, H² = 1.18
Test of group differences: Qb(2) = 0.15, p = 0.93
1/4 1/2 1 2 4
Random-effects REML model

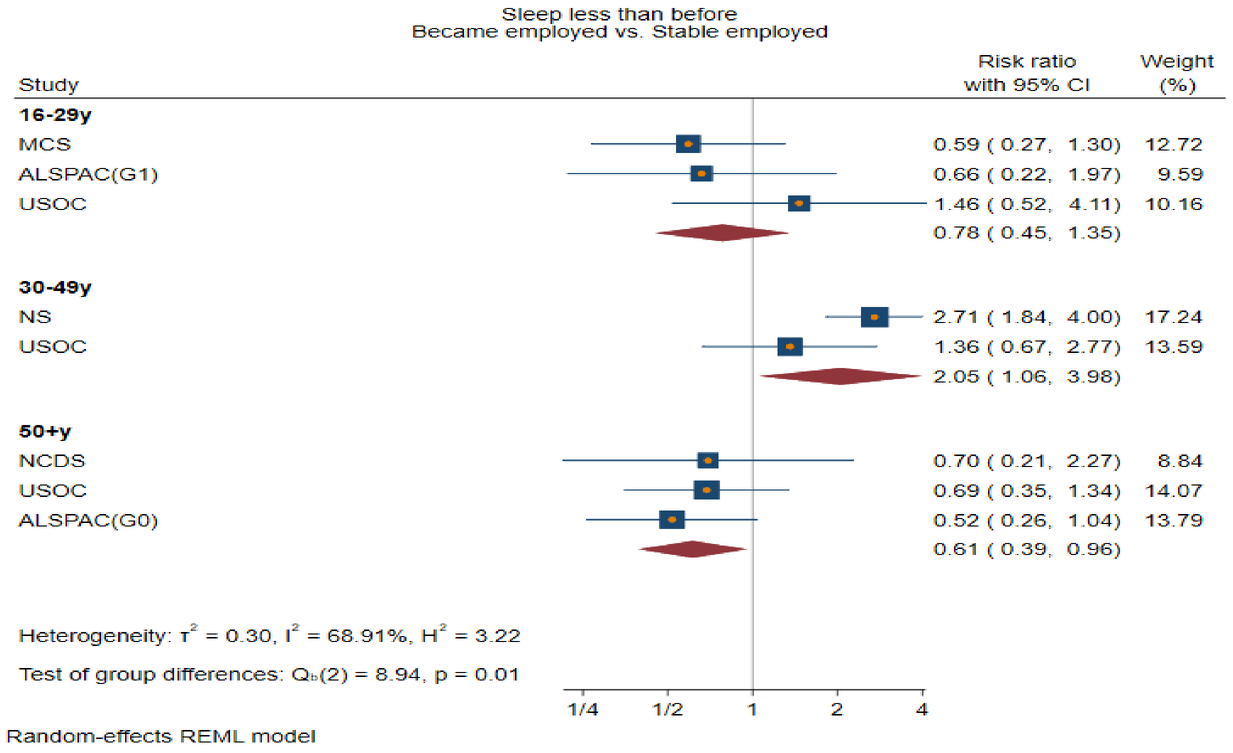
Sleep less than before
Became employed vs. Stable employed
Study
Risk ratio with 95% CI
Weight (%)
16-29y
MCS 0.59 (0.27, 1.30) 12.72
ALSPAC(G1) 0.66 (0.22, 1.97) 9.59
USOC 1.46 (0.52, 4.11) 10.16
0.78 (0.45, 1.35)
30-49y
NS 2.71 (1.84, 4.00) 17.24
USOC 1.36 (0.67, 2.77) 13.59
2.05 (1.06, 3.98)
50+y
NCDS 0.70 (0.21, 2.27) 8.84
USOC 0.69 (0.35, 1.34) 14.07
ALSPAC(G0) 0.52 (0.26, 1.04) 13.79
0.61 (0.39, 0.96)
Heterogeneity: τ² = 0.30, I² = 68.91%, H² = 3.22
Test of group differences: Qb(2) = 8.94, p = 0.01
1/4 1/2 1 2 4
Random-effects REML model

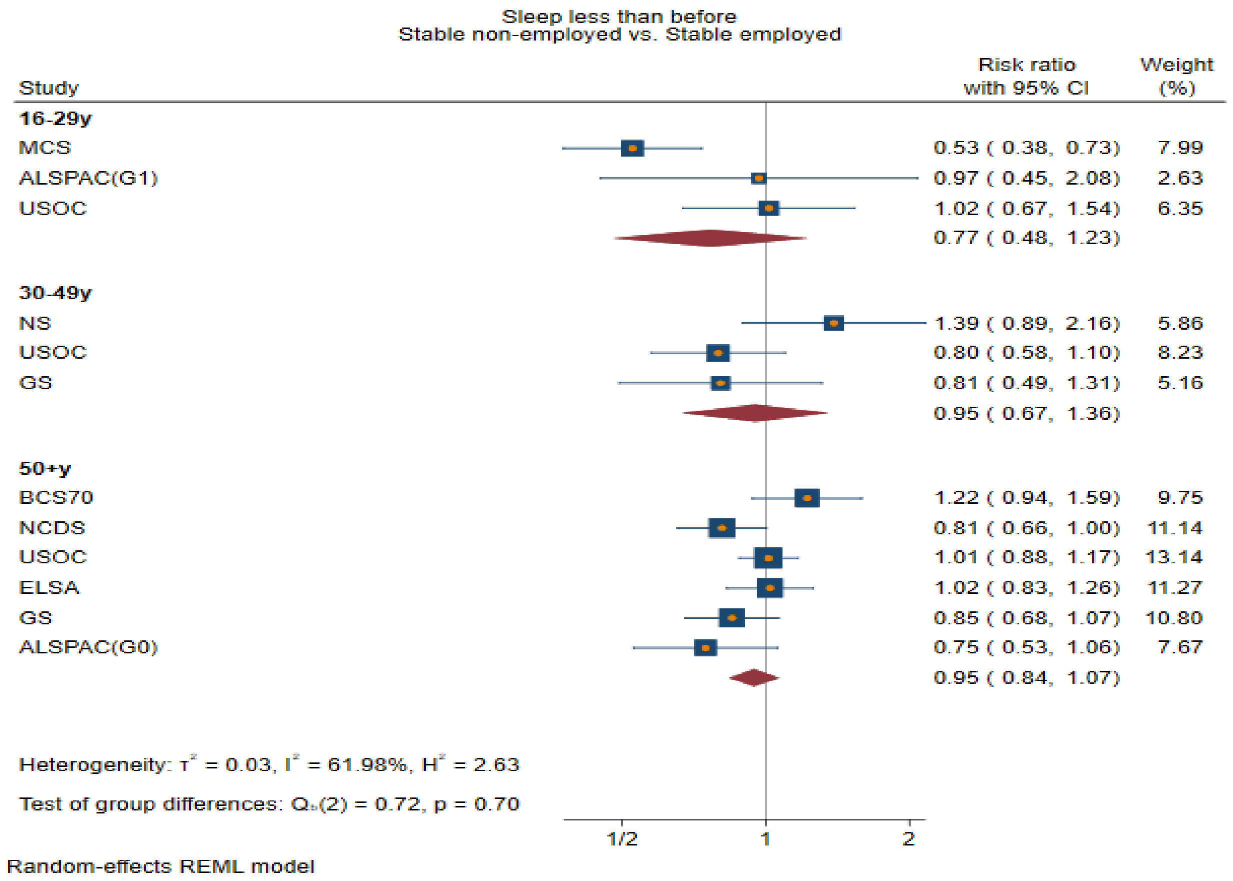
Sleep less than before
Stable non-employed vs. Stable employed
Study
Risk ratio with 95% CI
Weight (%)
16-29y
MCS 0.53 (0.38, 0.73) 7.99
ALSPAC(G1) 0.97 (0.45, 2.08) 2.63
USOC 1.02 (0.67, 1.54) 6.35
0.77 (0.48, 1.23)
30-49y
NS 1.39 (0.89, 2.16) 5.86
USOC 0.80 (0.58, 1.10) 8.23
GS 0.81 (0.49, 1.31) 5.16
0.95 (0.67, 1.36)
50+y
BCS70 1.22 (0.94, 1.59) 9.75
NCDS 0.81 (0.66, 1.00) 11.14
USOC 1.01 (0.88, 1.17) 13.14
ELSA 1.02 (0.83, 1.26) 11.27
GS 0.85 (0.68, 1.07) 10.80
ALSPAC(G0) 0.75 (0.53, 1.06) 7.67
0.95 (0.84, 1.07)
Heterogeneity: τ² = 0.03, I² = 61.98%, H² = 2.63
Test of group differences: Qb(2) = 0.72, p = 0.70
1/2 1 2
Random-effects REML model

Figure set 9: Sleeps less than before

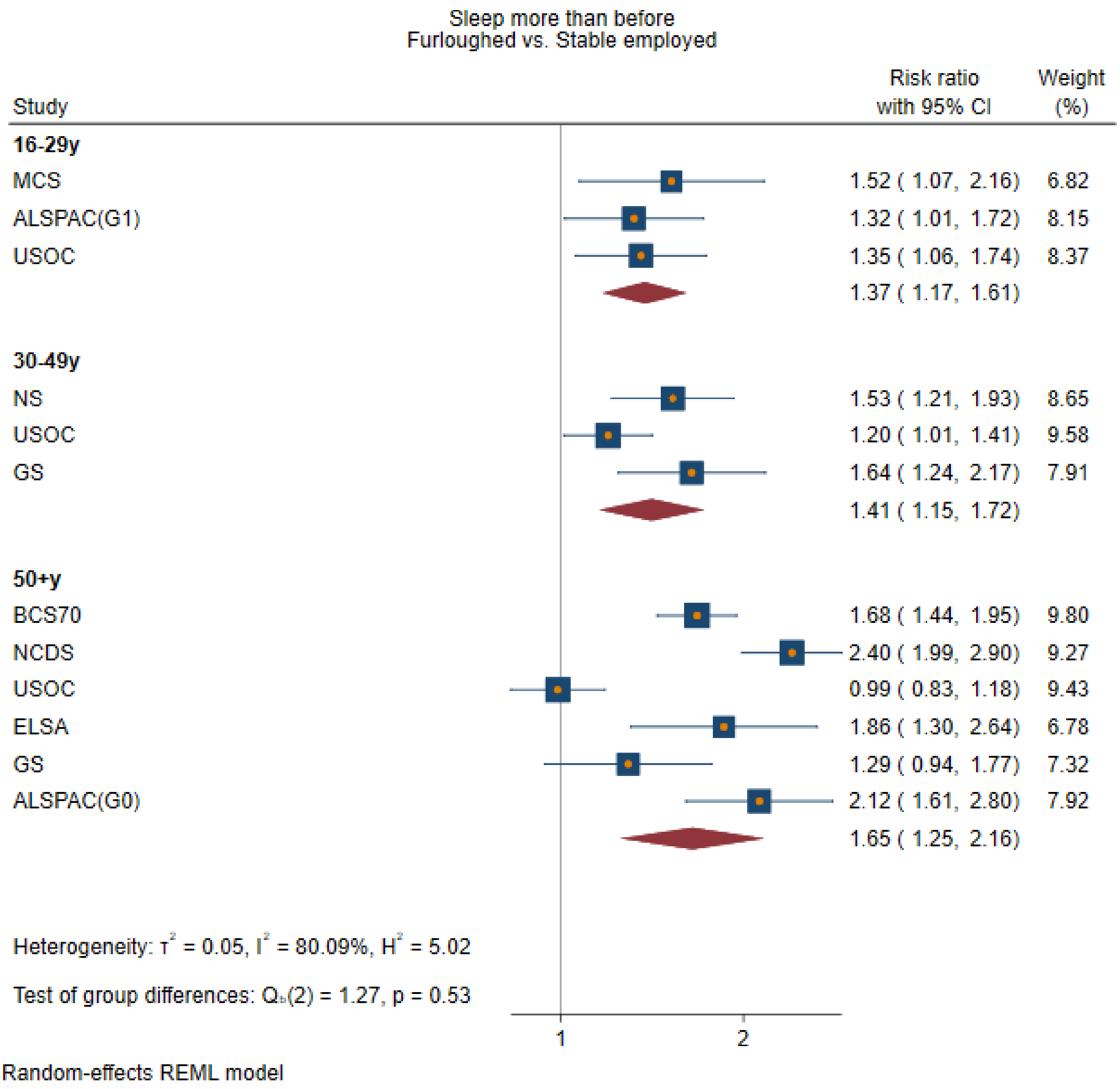

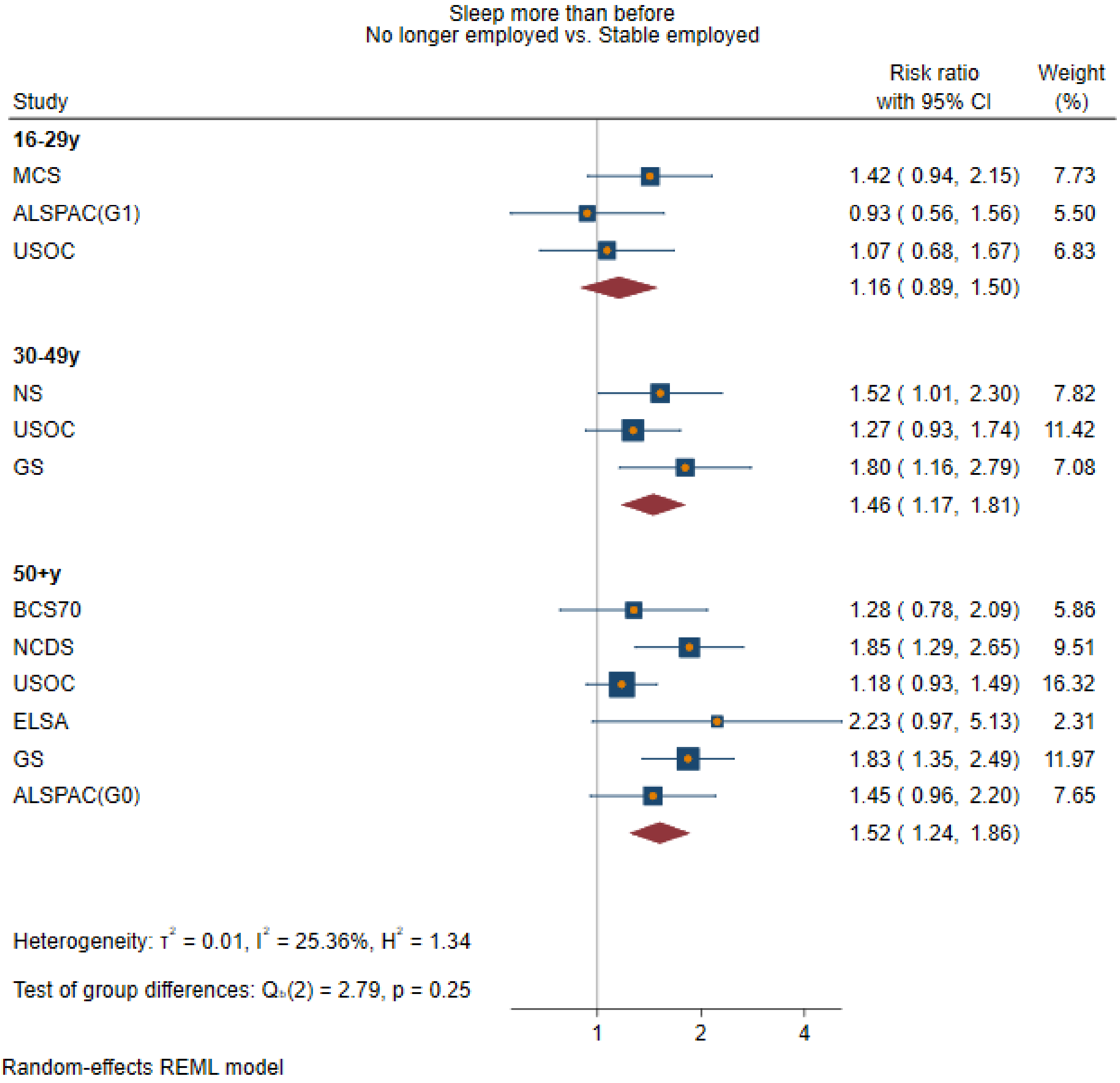

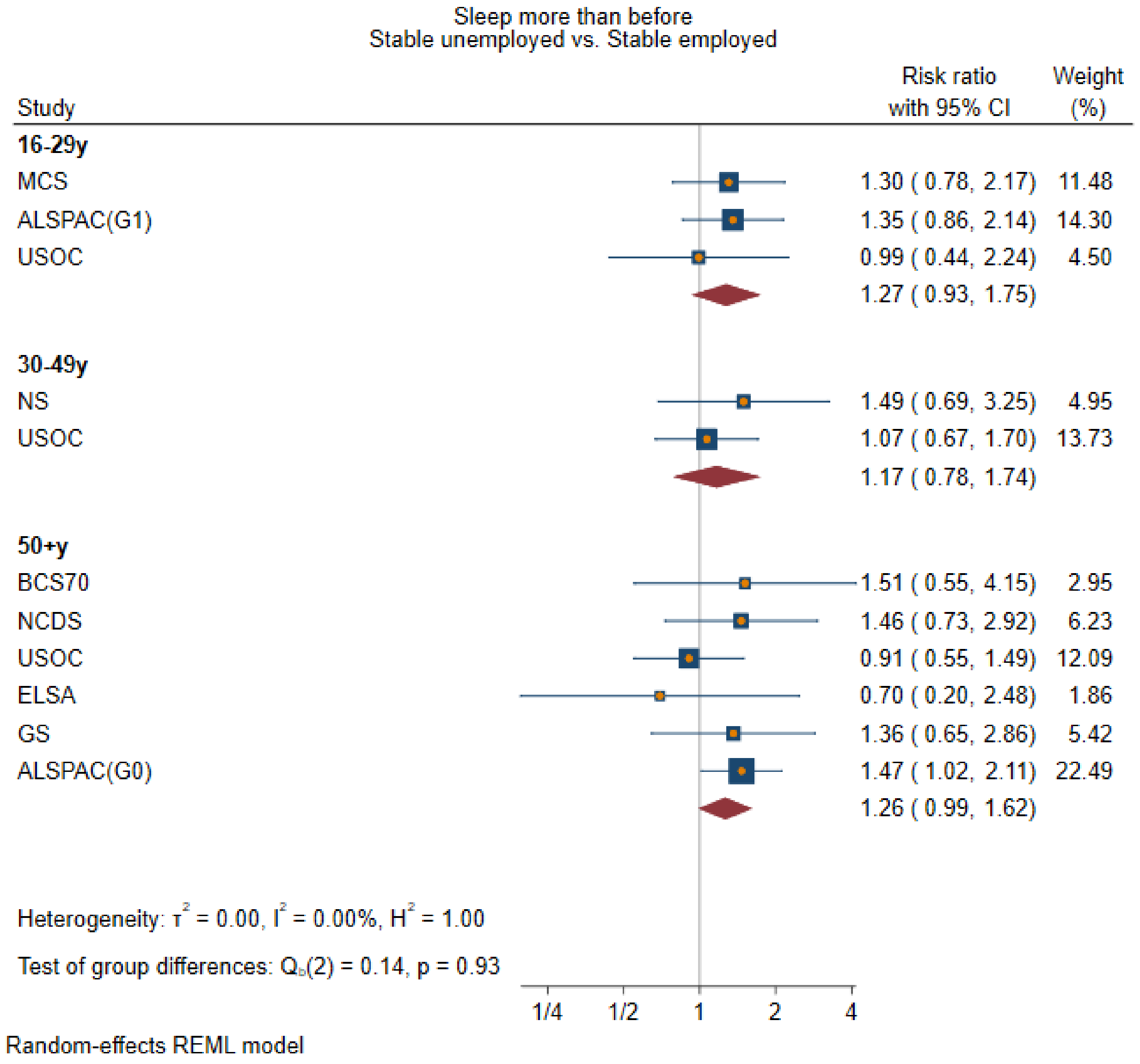
Sleep more than before
Stable unemployed vs. Stable employed
Study
Risk ratio with 95% CI
Weight (%)
16-29y
MCS 1.30 (0.78, 2.17) 11.48
ALSPAC(G1) 1.35 (0.86, 2.14) 14.30
USOC 0.99 (0.44, 2.24) 4.50
1.27 (0.93, 1.75)
30-49y
NS 1.49 (0.69, 3.25) 4.95
USOC 1.07 (0.67, 1.70) 13.73
1.17 (0.78, 1.74)
50+y
BCS70 1.51 (0.55, 4.15) 2.95
NCDS 1.46 (0.73, 2.92) 6.23
USOC 0.91 (0.55, 1.49) 12.09
ELSA 0.70 (0.20, 2.48) 1.86
GS 1.36 (0.65, 2.86) 5.42
ALSPAC(G0) 1.47 (1.02, 2.11) 22.49
1.26 (0.99, 1.62)
Heterogeneity: τ^2 = 0.00, I^2 = 0.00%, H^2 = 1.00
Test of group differences: $Q_b(2)$ = 0.14, p = 0.93
1/4 1/2 1 2 4
Random-effects REML model

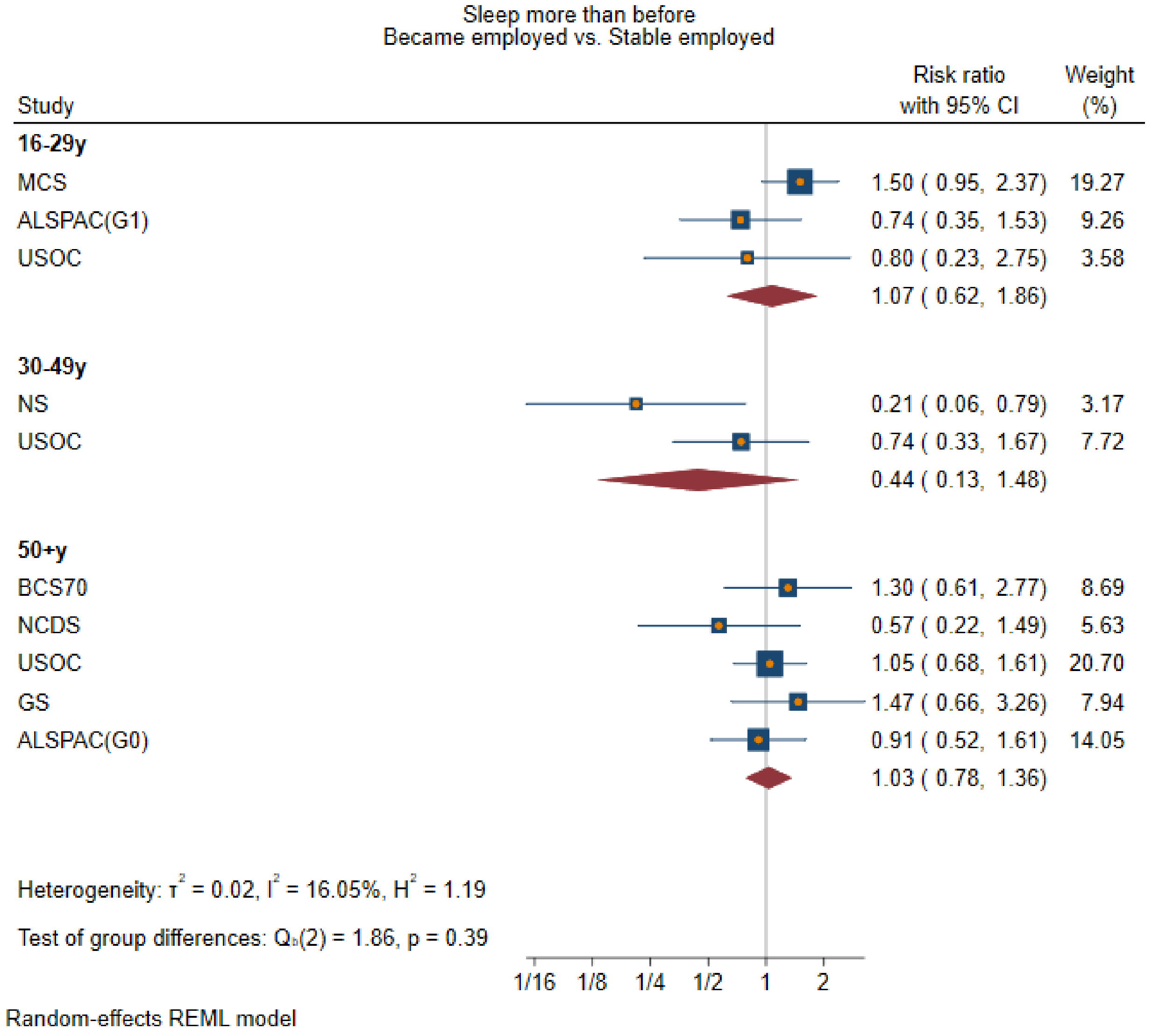
Sleep more than before
Became employed vs. Stable employed
Study
Risk ratio with 95% CI
Weight (%)
16-29y
MCS 1.50 (0.95, 2.37) 19.27
ALSPAC(G1) 0.74 (0.35, 1.53) 9.26
USOC 0.80 (0.23, 2.75) 3.58
1.07 (0.62, 1.86)
30-49y
NS 0.21 (0.06, 0.79) 3.17
USOC 0.74 (0.33, 1.67) 7.72
0.44 (0.13, 1.48)
50+y
BCS70 1.30 (0.61, 2.77) 8.69
NCDS 0.57 (0.22, 1.49) 5.63
USOC 1.05 (0.68, 1.61) 20.70
GS 1.47 (0.66, 3.26) 7.94
ALSPAC(G0) 0.91 (0.52, 1.61) 14.05
1.03 (0.78, 1.36)
Heterogeneity: τ^2 = 0.02, I^2 = 16.05%, H^2 = 1.19
Test of group differences: $Q_b(2)$ = 1.86, p = 0.39
1/16 1/8 1/4 1/2 1 2
Random-effects REML model

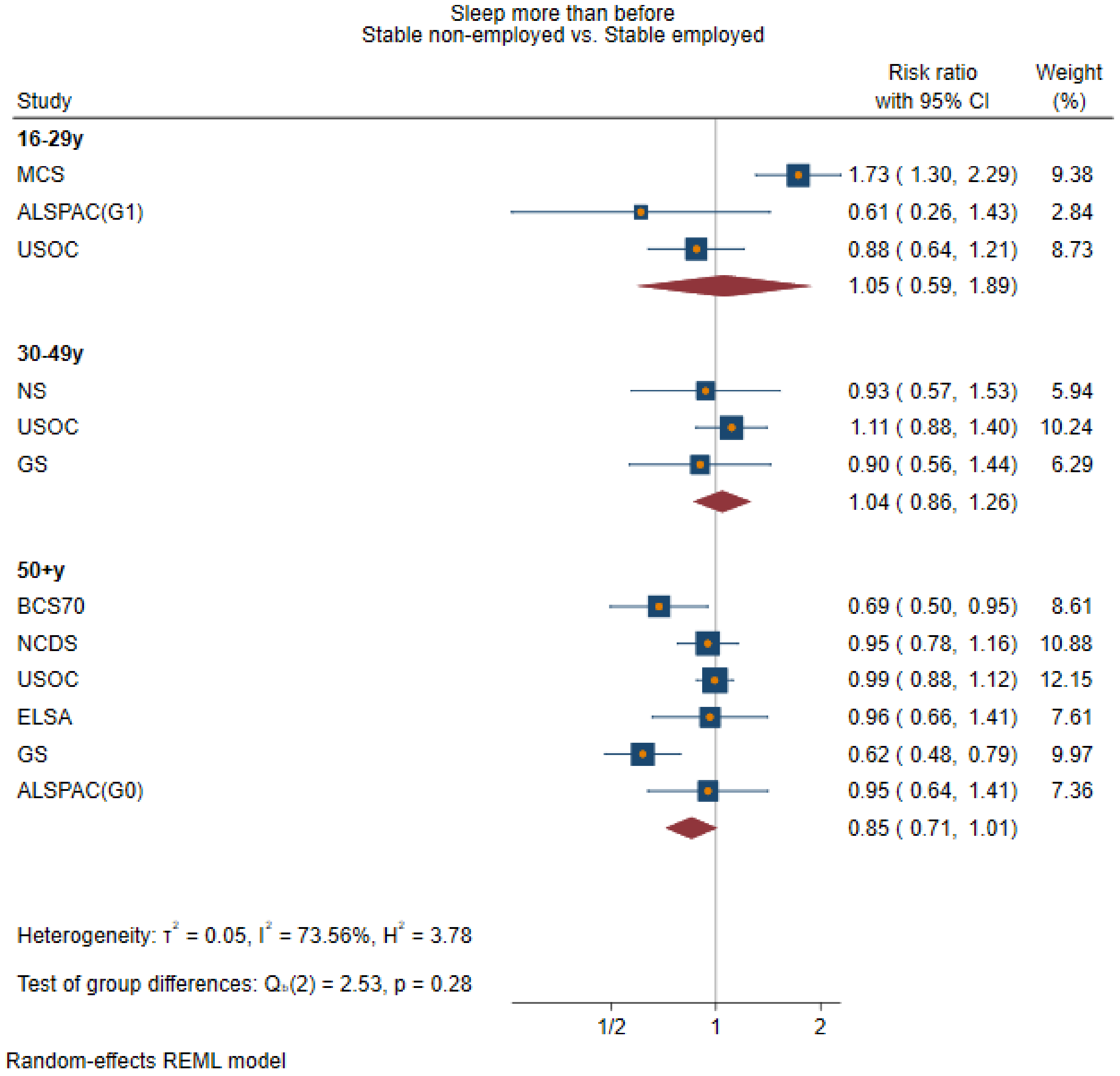
Sleep more than before
Stable non-employed vs. Stable employed
Study
Risk ratio with 95% CI
Weight (%)
16-29y
MCS 1.73 (1.30, 2.29) 9.38
ALSPAC(G1) 0.61 (0.26, 1.43) 2.84
USOC 0.88 (0.64, 1.21) 8.73
1.05 (0.59, 1.89)
30-49y
NS 0.93 (0.57, 1.53) 5.94
USOC 1.11 (0.88, 1.40) 10.24
GS 0.90 (0.56, 1.44) 6.29
1.04 (0.86, 1.26)
50+y
BCS70 0.69 (0.50, 0.95) 8.61
NCDS 0.95 (0.78, 1.16) 10.88
USOC 0.99 (0.88, 1.12) 12.15
ELSA 0.96 (0.66, 1.41) 7.61
GS 0.62 (0.48, 0.79) 9.97
ALSPAC(G0) 0.95 (0.64, 1.41) 7.36
0.85 (0.71, 1.01)
Heterogeneity: $\tau^2 = 0.05$, $I^2 = 73.56\%$, $H^2 = 3.78$
Test of group differences: $Q_b(2) = 2.53$, $p = 0.28$
1/2
1
2
Random-effects REML model

Figure set 10: From 6/9h a night to outside ‘normal range’

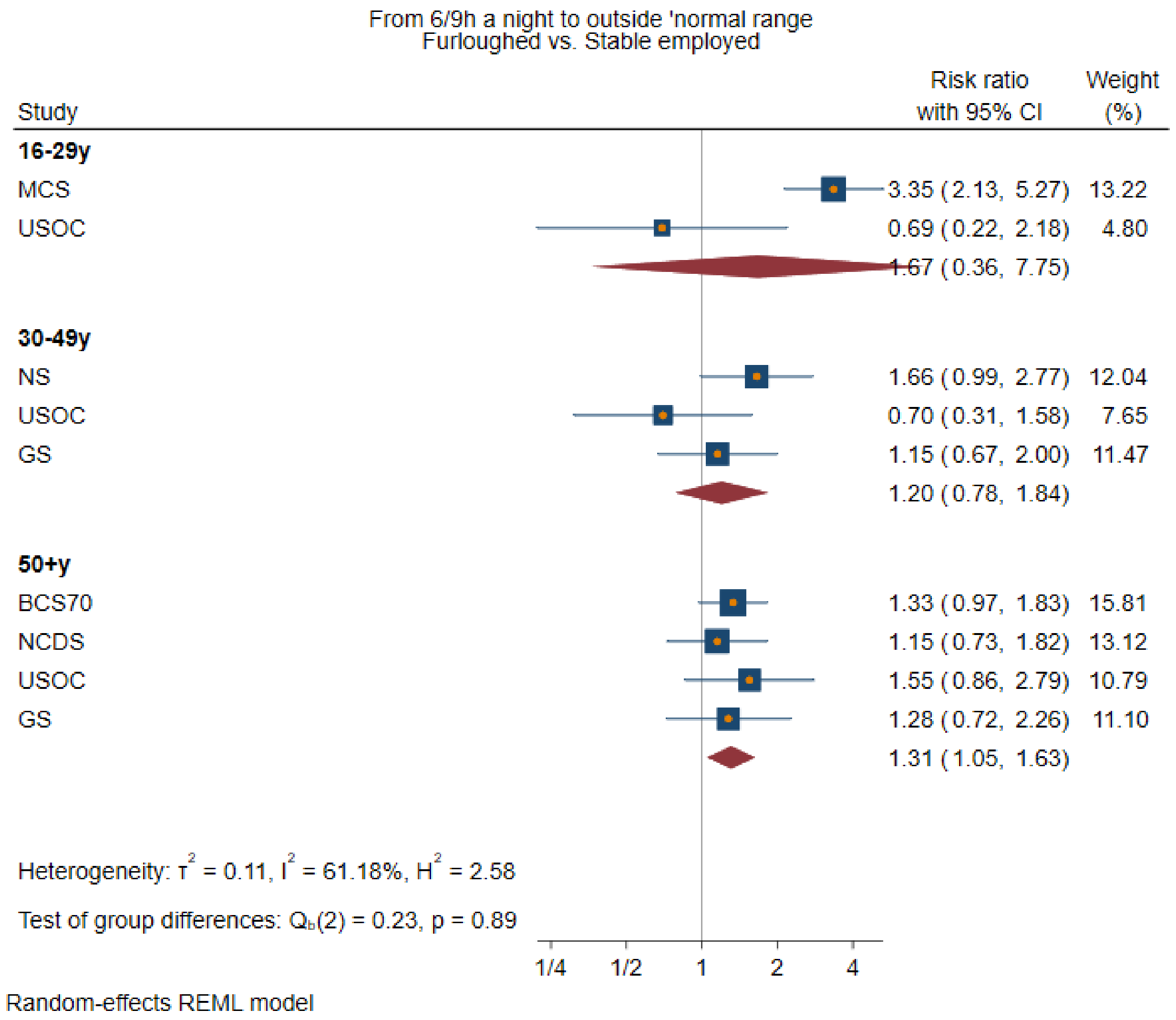

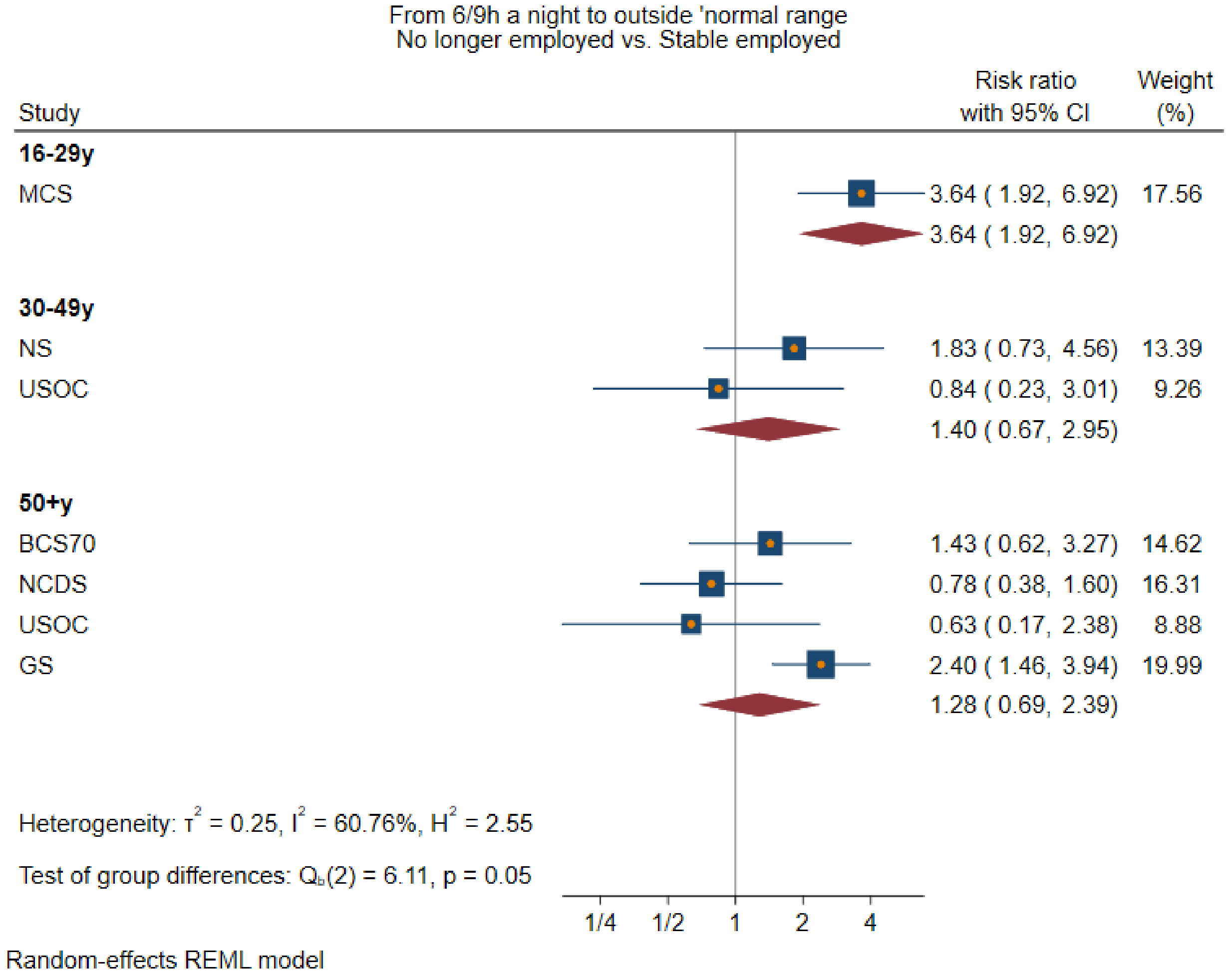

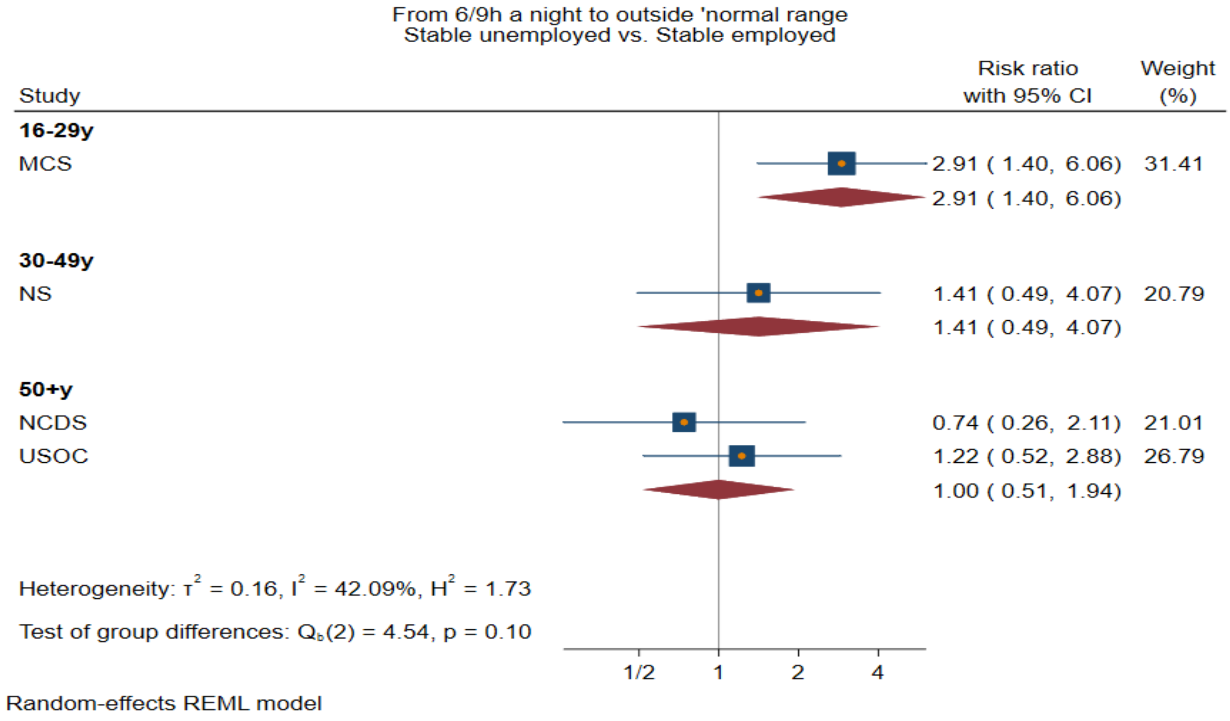
From 6/9h a night to outside 'normal range
Stable unemployed vs. Stable employed
Study
Risk ratio with 95% CI
Weight (%)
16-29y
MCS
2.91 (1.40, 6.06) 31.41
2.91 (1.40, 6.06)
30-49y
NS
1.41 (0.49, 4.07) 20.79
1.41 (0.49, 4.07)
50+y
NCDS
0.74 (0.26, 2.11) 21.01
USOC
1.22 (0.52, 2.88) 26.79
1.00 (0.51, 1.94)
Heterogeneity: τ2 = 0.16, I2 = 42.09%, H2 = 1.73
Test of group differences: Qb(2) = 4.54, p = 0.10
1/2
1
2
4
Random-effects REML model

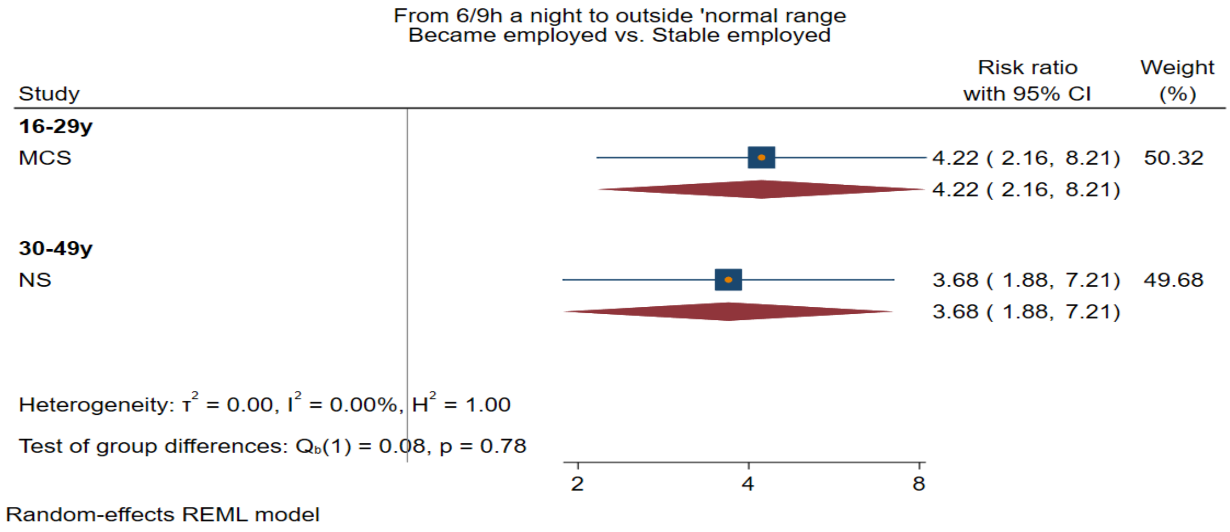
From 6/9h a night to outside 'normal range
Became employed vs. Stable employed
Study
Risk ratio with 95% CI
Weight (%)
16-29y
MCS
4.22 (2.16, 8.21) 50.32
4.22 (2.16, 8.21)
30-49y
NS
3.68 (1.88, 7.21) 49.68
3.68 (1.88, 7.21)
Heterogeneity: τ2 = 0.00, I2 = 0.00%, H2 = 1.00
Test of group differences: Qb(1) = 0.08, p = 0.78
2
4
8
Random-effects REML model

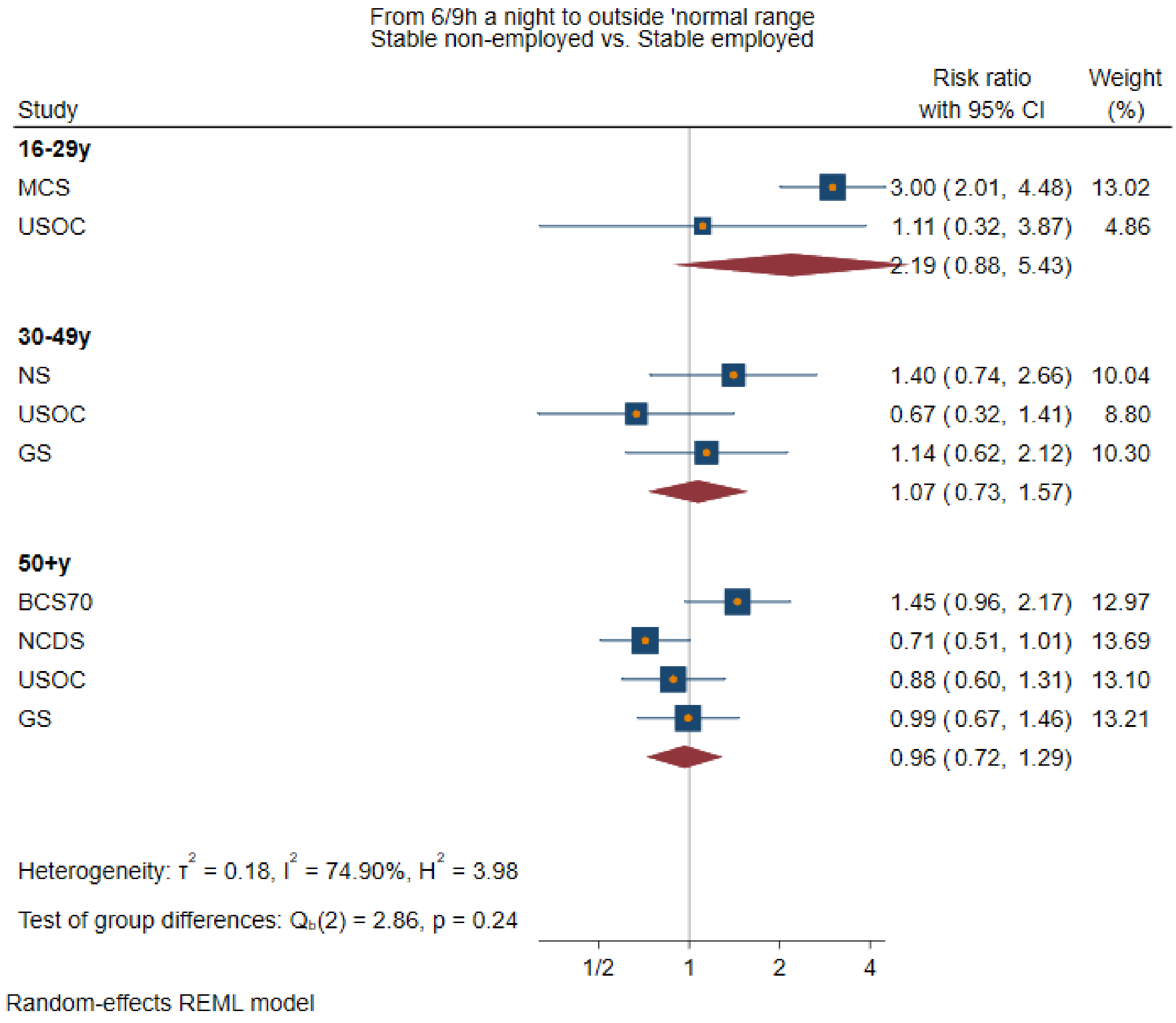
From 6/9h a night to outside 'normal range
Stable non-employed vs. Stable employed
Study
Risk ratio with 95% CI
Weight (%)
16-29y
MCS
3.00 (2.01, 4.48) 13.02
USOC
1.11 (0.32, 3.87) 4.86
2.19 (0.88, 5.43)
30-49y
NS
1.40 (0.74, 2.66) 10.04
USOC
0.67 (0.32, 1.41) 8.80
GS
1.14 (0.62, 2.12) 10.30
1.07 (0.73, 1.57)
50+y
BCS70
1.45 (0.96, 2.17) 12.97
NCDS
0.71 (0.51, 1.01) 13.69
USOC
0.88 (0.60, 1.31) 13.10
GS
0.99 (0.67, 1.46) 13.21
0.96 (0.72, 1.29)
Heterogeneity: τ2 = 0.18, I2 = 74.90%, H2 = 3.98
Test of group differences: Qb(2) = 2.86, p = 0.24
1/2
1
2
4
Random-effects REML model

Figure set 11: From outside ‘normal range’ to 6/9h a night

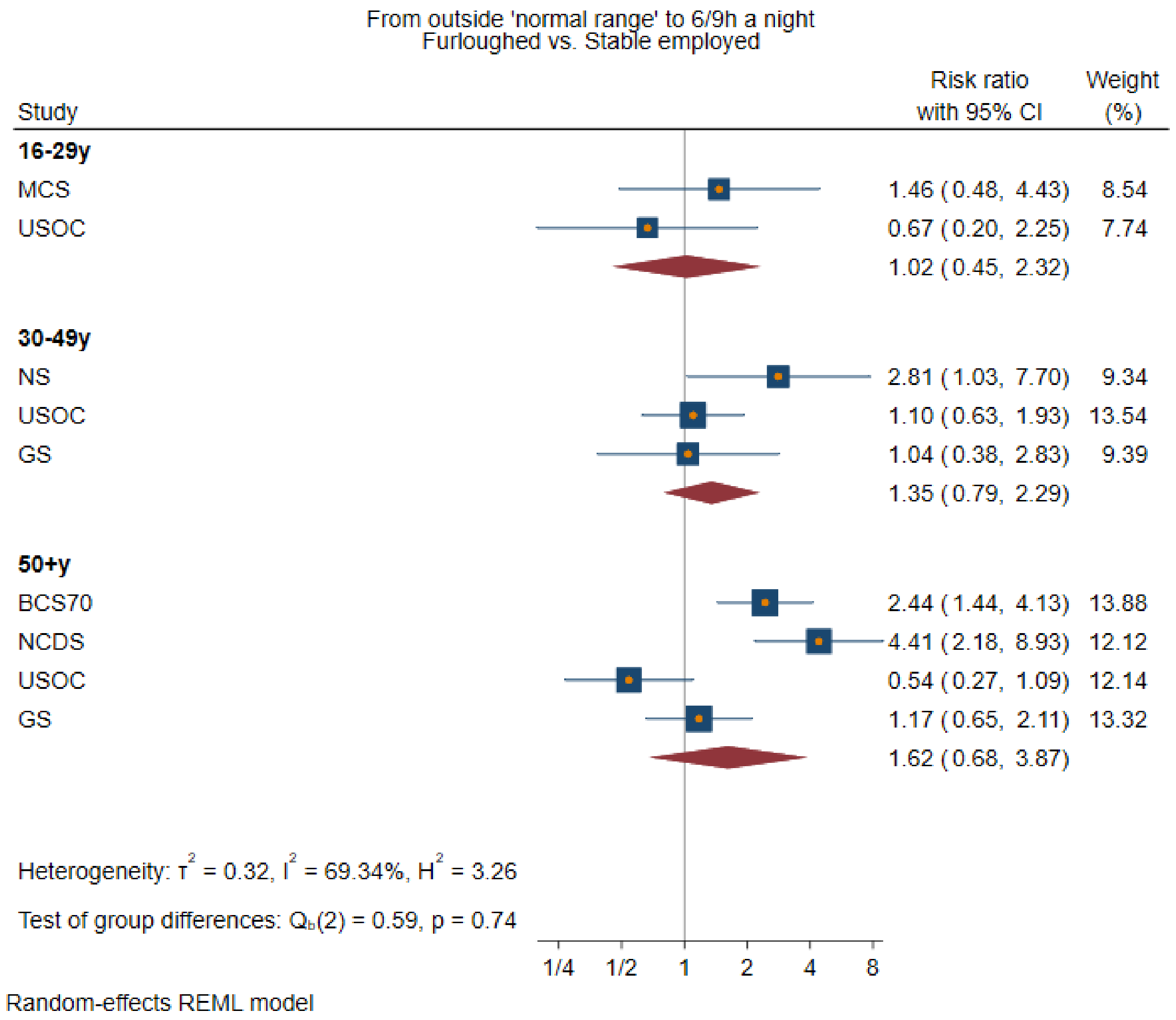

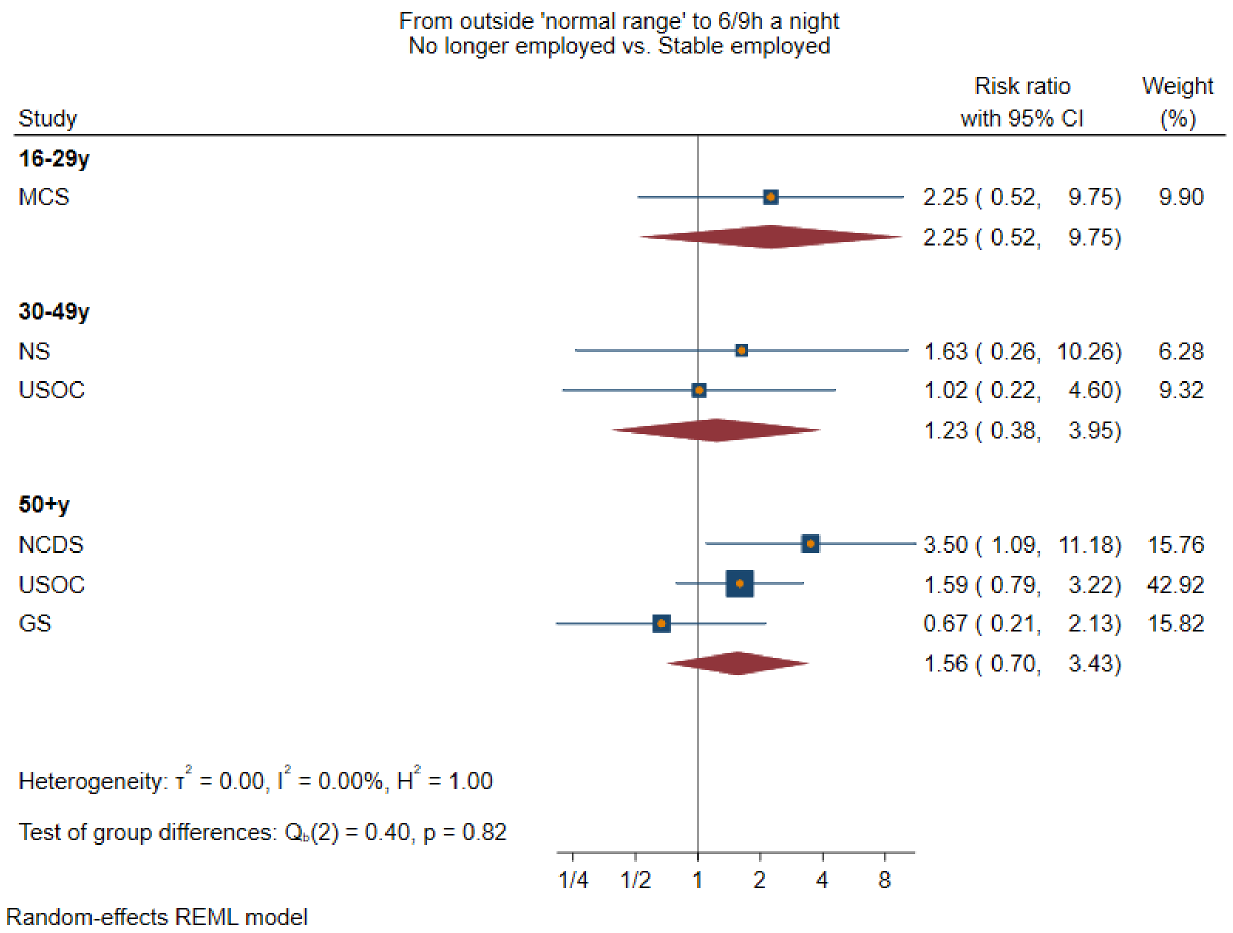

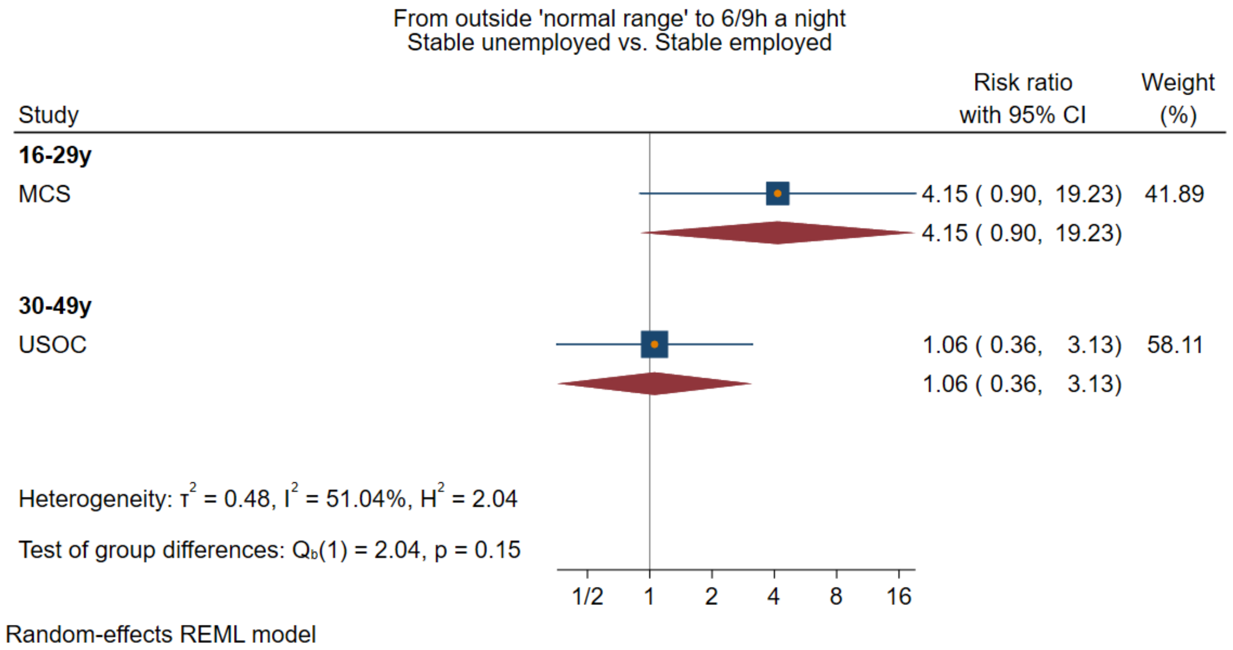
From outside 'normal range' to 6/9h a night
Stable unemployed vs. Stable employed
Study
Risk ratio with 95% CI
Weight (%)
16-29y
MCS 4.15 (0.90, 19.23) 41.89
4.15 (0.90, 19.23)
30-49y
USOC 1.06 (0.36, 3.13) 58.11
1.06 (0.36, 3.13)
Heterogeneity: τ² = 0.48, I² = 51.04%, H² = 2.04
Test of group differences: Q_b(1) = 2.04, p = 0.15
1/2 1 2 4 8 16
Random-effects REML model

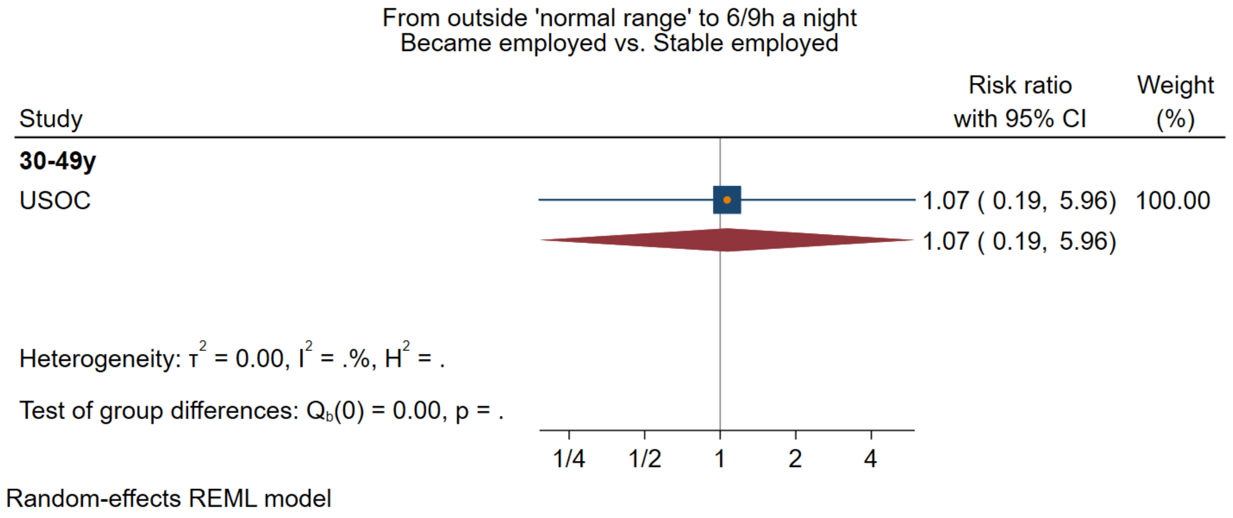
From outside 'normal range' to 6/9h a night
Became employed vs. Stable employed
Study
Risk ratio with 95% CI
Weight (%)
30-49y
USOC 1.07 (0.19, 5.96) 100.00
1.07 (0.19, 5.96)
Heterogeneity: τ² = 0.00, I² = .%, H² = .
Test of group differences: Q_b(0) = 0.00, p = .
1/4 1/2 1 2 4
Random-effects REML model

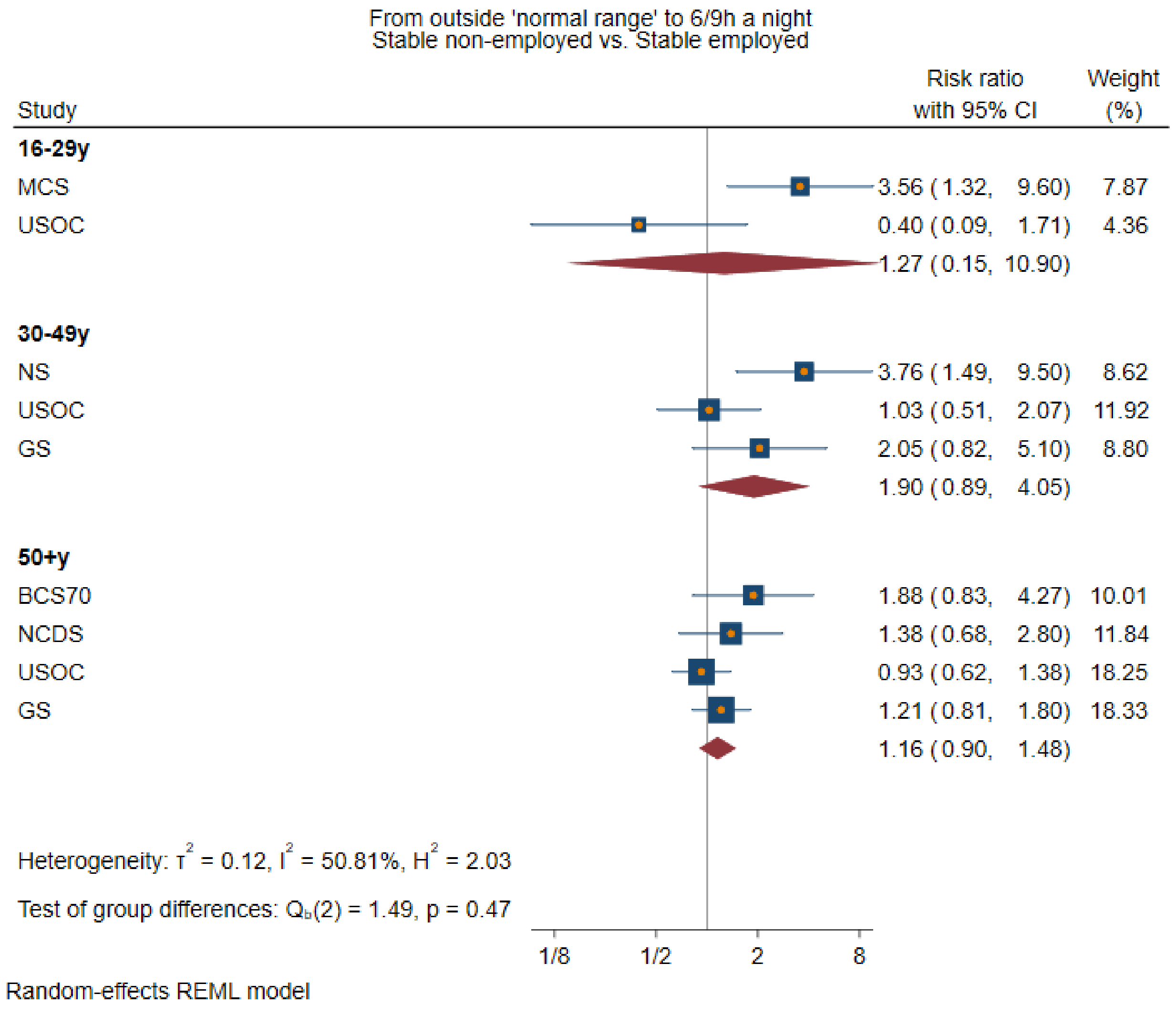
From outside 'normal range' to 6/9h a night
Stable non-employed vs. Stable employed
Study
Risk ratio with 95% CI
Weight (%)
16-29y
MCS 3.56 (1.32, 9.60) 7.87
USOC 0.40 (0.09, 1.71) 4.36
1.27 (0.15, 10.90)
30-49y
NS 3.76 (1.49, 9.50) 8.62
USOC 1.03 (0.51, 2.07) 11.92
GS 2.05 (0.82, 5.10) 8.80
1.90 (0.89, 4.05)
50+y
BCS70 1.88 (0.83, 4.27) 10.01
NCDS 1.38 (0.68, 2.80) 11.84
USOC 0.93 (0.62, 1.38) 18.25
GS 1.21 (0.81, 1.80) 18.33
1.16 (0.90, 1.48)
Heterogeneity: τ² = 0.12, I² = 50.81%, H² = 2.03
Test of group differences: Q_b(2) = 1.49, p = 0.47
1/8 1/2 2 8
Random-effects REML model

Figure set 12: Currently eats 2 or fewer fruit and veg

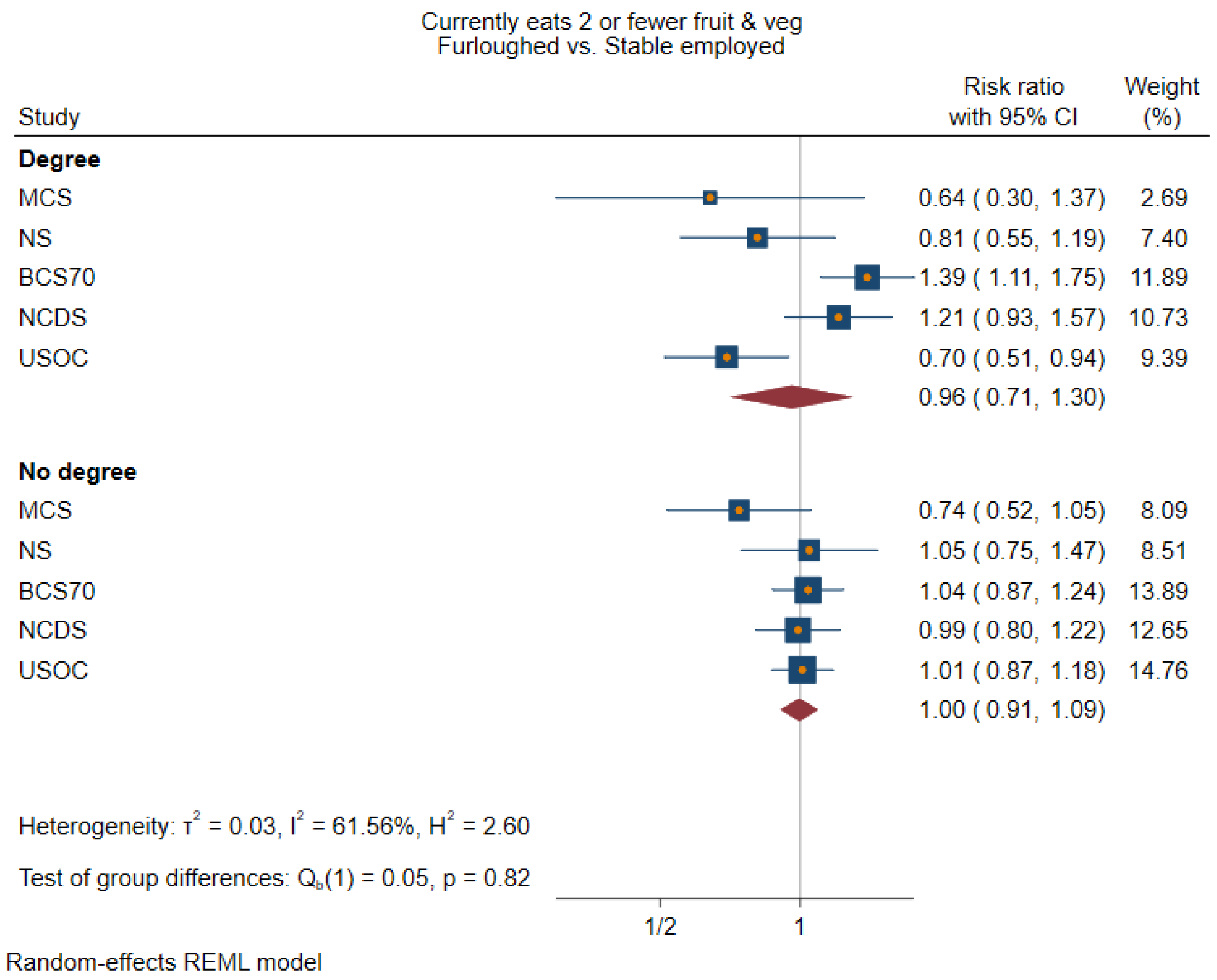

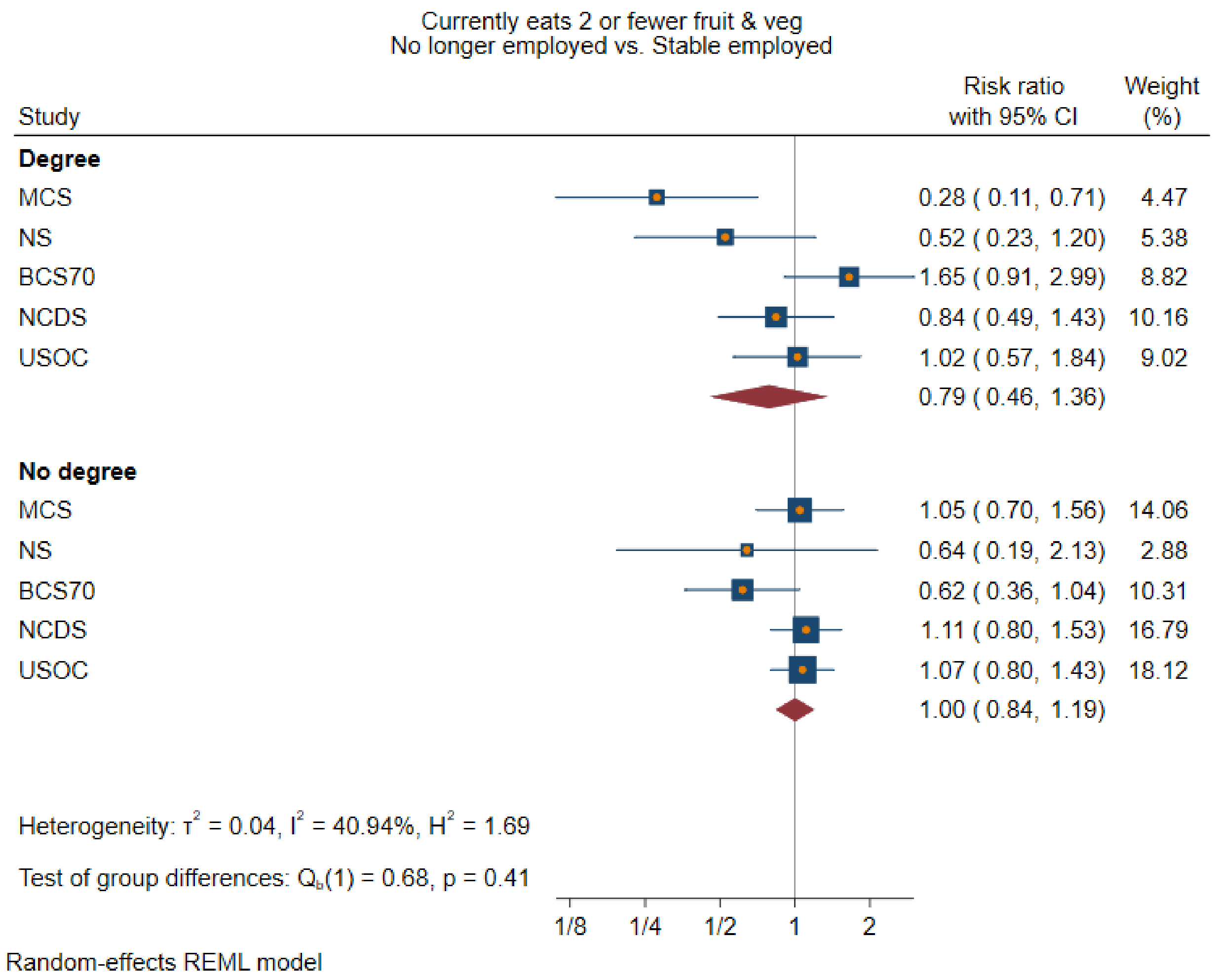

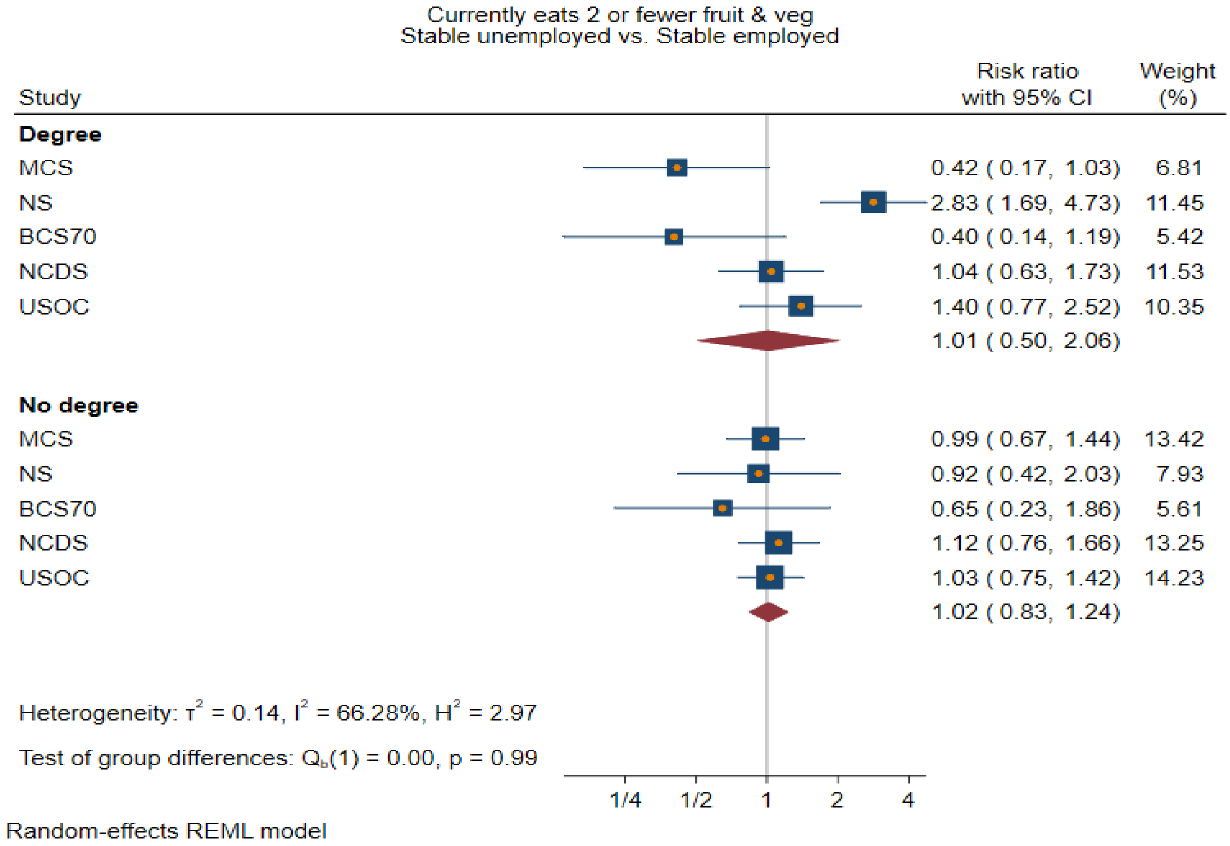
Currently eats 2 or fewer fruit & veg
Stable unemployed vs. Stable employed
Study
Risk ratio with 95% CI
Weight (%)
Degree
MCS 0.42 (0.17, 1.03) 6.81
NS 2.83 (1.69, 4.73) 11.45
BCS70 0.40 (0.14, 1.19) 5.42
NCDS 1.04 (0.63, 1.73) 11.53
USOC 1.40 (0.77, 2.52) 10.35
1.01 (0.50, 2.06)
No degree
MCS 0.99 (0.67, 1.44) 13.42
NS 0.92 (0.42, 2.03) 7.93
BCS70 0.65 (0.23, 1.86) 5.61
NCDS 1.12 (0.76, 1.66) 13.25
USOC 1.03 (0.75, 1.42) 14.23
1.02 (0.83, 1.24)
Heterogeneity: τ² = 0.14, I² = 66.28%, H² = 2.97
Test of group differences: Qb(1) = 0.00, p = 0.99
1/4
1/2
1
2
4
Random-effects REML model

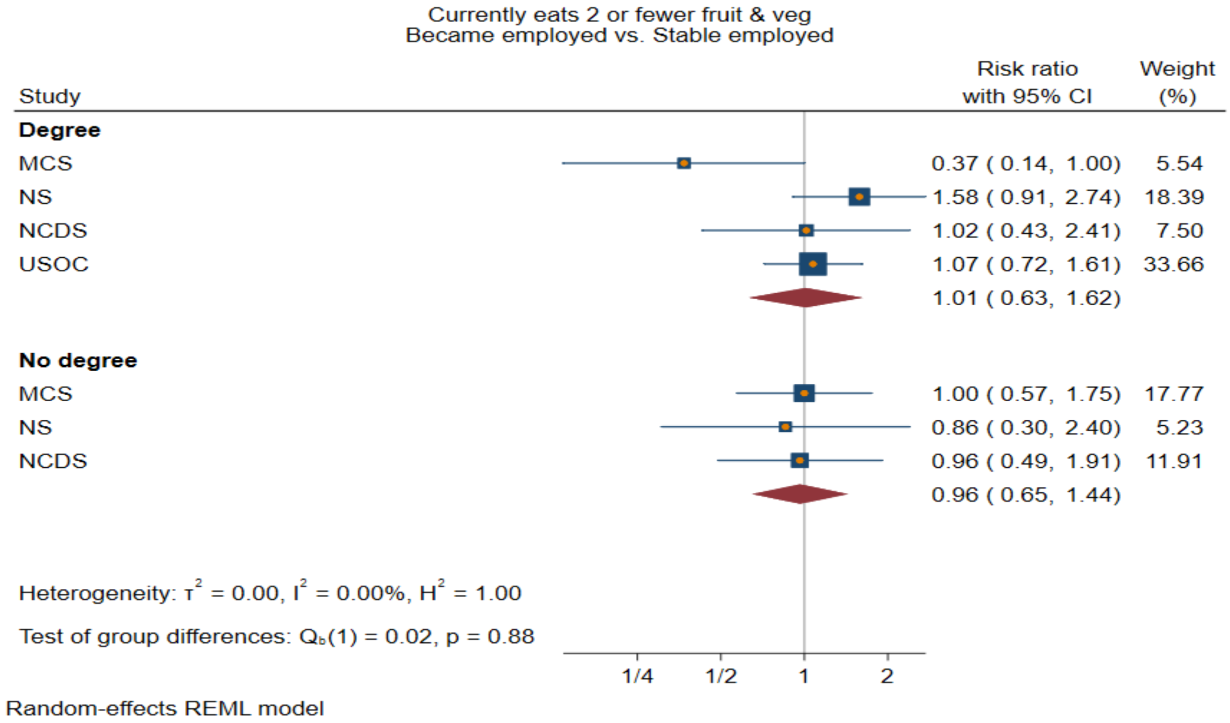
Currently eats 2 or fewer fruit & veg
Became employed vs. Stable employed
Study
Risk ratio with 95% CI
Weight (%)
Degree
MCS 0.37 (0.14, 1.00) 5.54
NS 1.58 (0.91, 2.74) 18.39
NCDS 1.02 (0.43, 2.41) 7.50
USOC 1.07 (0.72, 1.61) 33.66
1.01 (0.63, 1.62)
No degree
MCS 1.00 (0.57, 1.75) 17.77
NS 0.86 (0.30, 2.40) 5.23
NCDS 0.96 (0.49, 1.91) 11.91
0.96 (0.65, 1.44)
Heterogeneity: τ² = 0.00, I² = 0.00%, H² = 1.00
Test of group differences: Qb(1) = 0.02, p = 0.88
1/4
1/2
1
2
Random-effects REML model

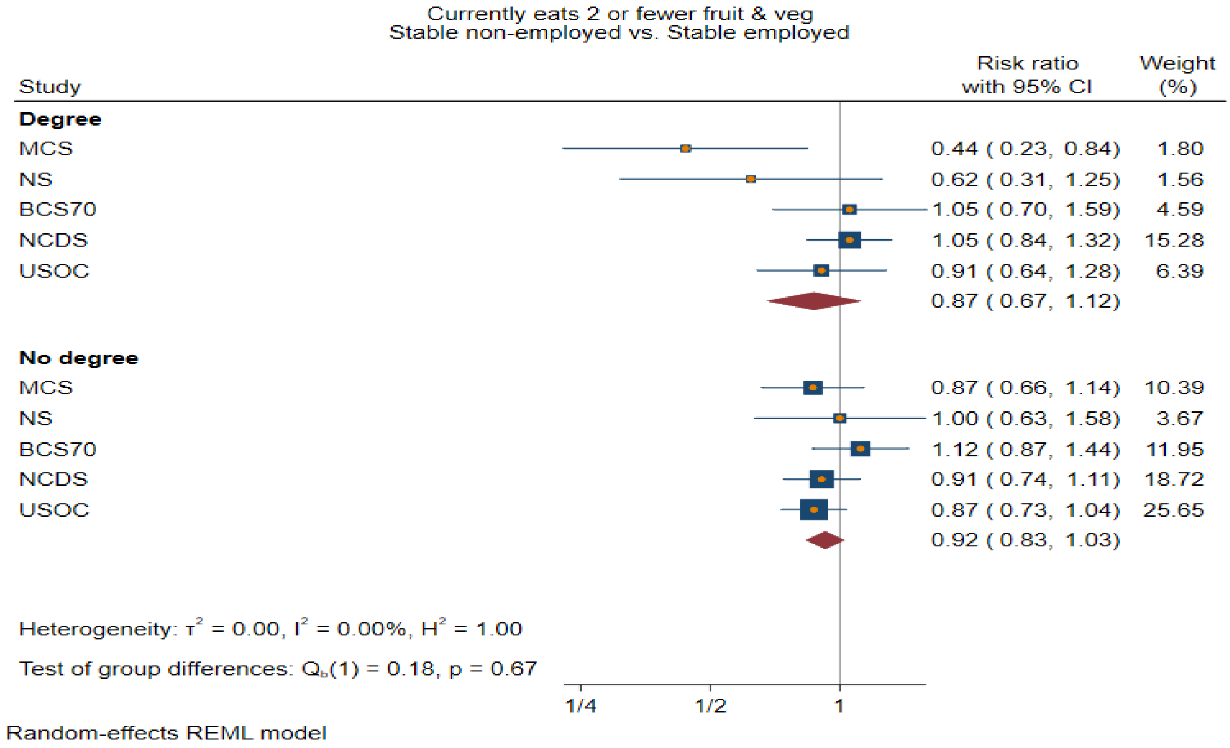
Currently eats 2 or fewer fruit & veg
Stable non-employed vs. Stable employed
Study
Risk ratio with 95% CI
Weight (%)
Degree
MCS 0.44 (0.23, 0.84) 1.80
NS 0.62 (0.31, 1.25) 1.56
BCS70 1.05 (0.70, 1.59) 4.59
NCDS 1.05 (0.84, 1.32) 15.28
USOC 0.91 (0.64, 1.28) 6.39
0.87 (0.67, 1.12)
No degree
MCS 0.87 (0.66, 1.14) 10.39
NS 1.00 (0.63, 1.58) 3.67
BCS70 1.12 (0.87, 1.44) 11.95
NCDS 0.91 (0.74, 1.11) 18.72
USOC 0.87 (0.73, 1.04) 25.65
0.92 (0.83, 1.03)
Heterogeneity: τ² = 0.00, I² = 0.00%, H² = 1.00
Test of group differences: Qb(1) = 0.18, p = 0.67
1/4
1/2
1
Random-effects REML model

Figure set 13: Fewer fruit and veg

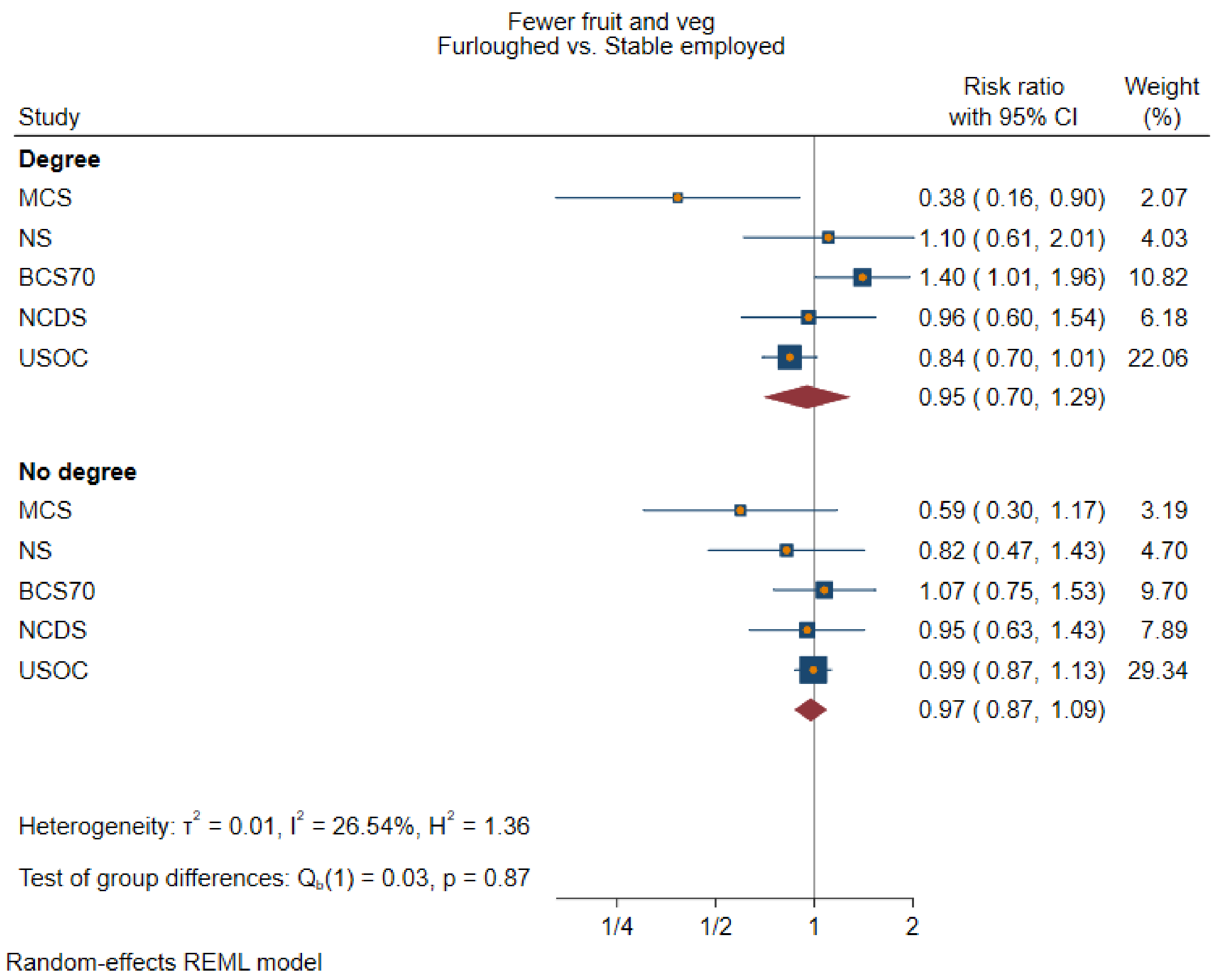

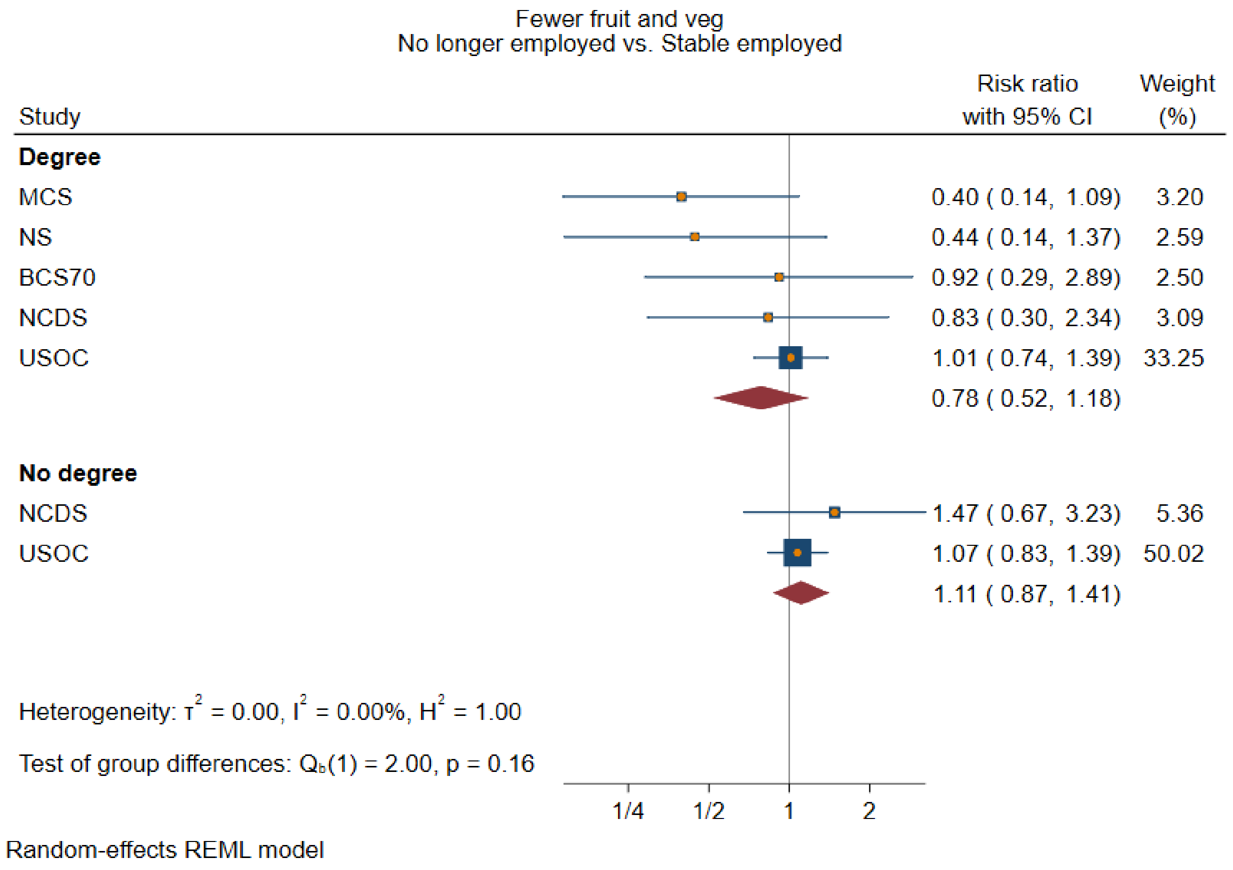

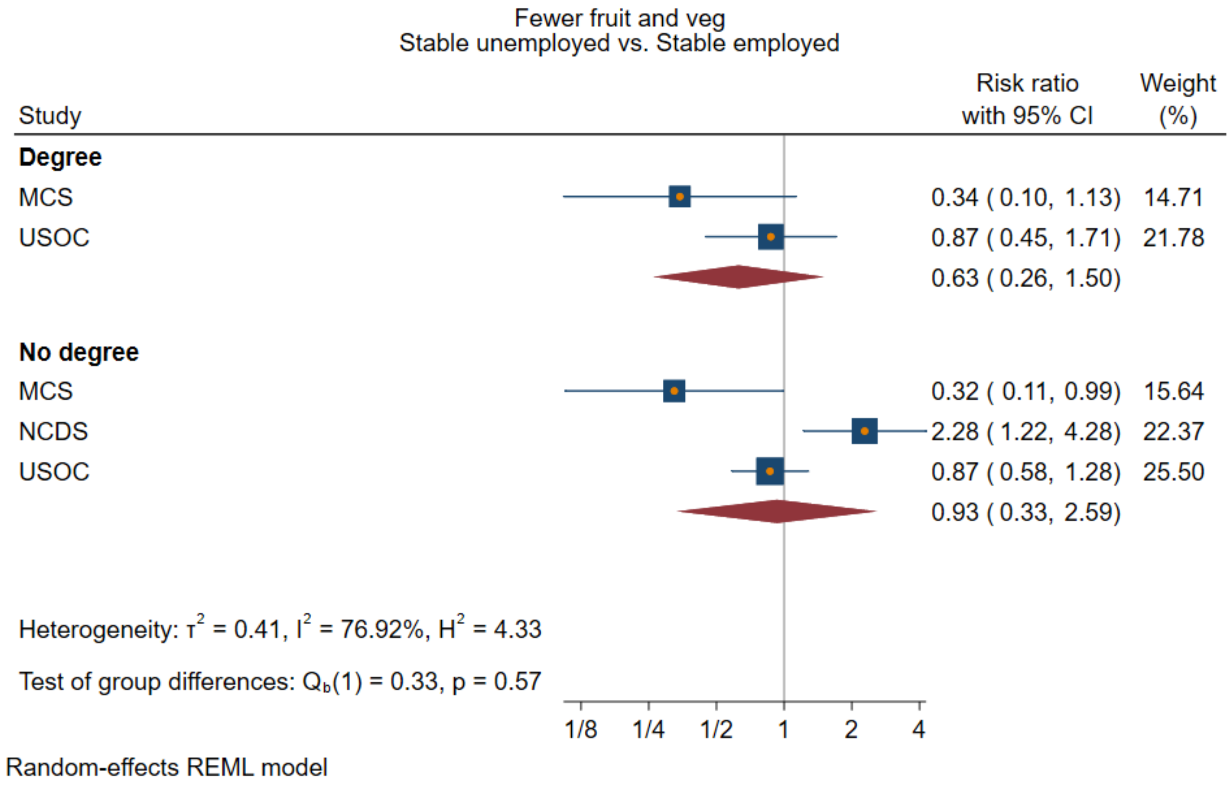
Fewer fruit and veg
Stable unemployed vs. Stable employed
Study
Risk ratio with 95% CI
Weight (%)
Degree
MCS 0.34 (0.10, 1.13) 14.71
USOC 0.87 (0.45, 1.71) 21.78
0.63 (0.26, 1.50)
No degree
MCS 0.32 (0.11, 0.99) 15.64
NCDS 2.28 (1.22, 4.28) 22.37
USOC 0.87 (0.58, 1.28) 25.50
0.93 (0.33, 2.59)
Heterogeneity: τ² = 0.41, I² = 76.92%, H² = 4.33
Test of group differences: Qb(1) = 0.33, p = 0.57
1/8 1/4 1/2 1 2 4
Random-effects REML model

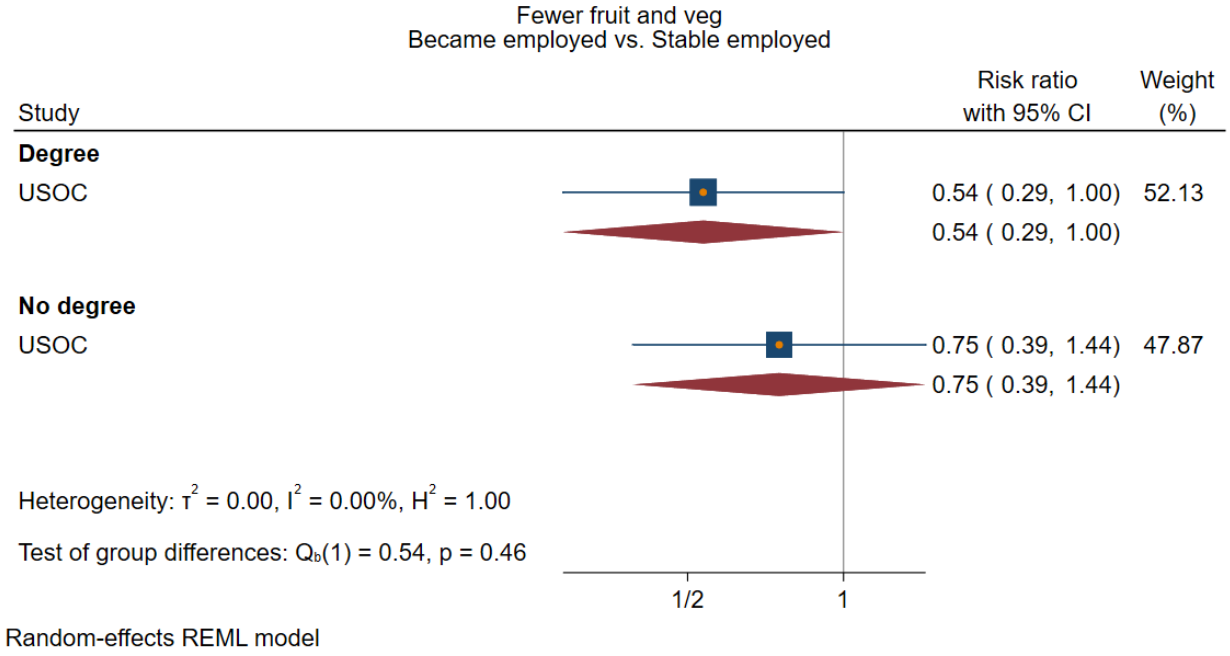
Fewer fruit and veg
Became employed vs. Stable employed
Study
Risk ratio with 95% CI
Weight (%)
Degree
USOC 0.54 (0.29, 1.00) 52.13
0.54 (0.29, 1.00)
No degree
USOC 0.75 (0.39, 1.44) 47.87
0.75 (0.39, 1.44)
Heterogeneity: τ² = 0.00, I² = 0.00%, H² = 1.00
Test of group differences: Qb(1) = 0.54, p = 0.46
1/2 1
Random-effects REML model

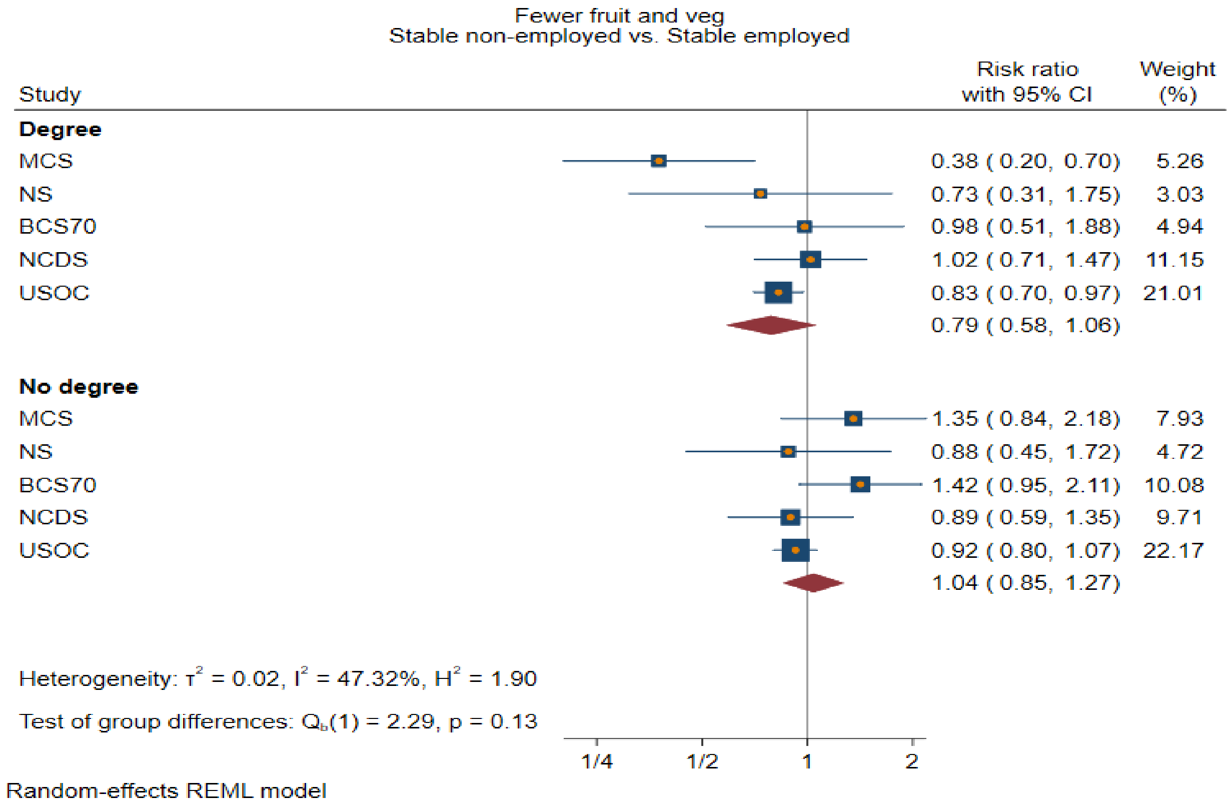
Fewer fruit and veg
Stable non-employed vs. Stable employed
Study
Risk ratio with 95% CI
Weight (%)
Degree
MCS 0.38 (0.20, 0.70) 5.26
NS 0.73 (0.31, 1.75) 3.03
BCS70 0.98 (0.51, 1.88) 4.94
NCDS 1.02 (0.71, 1.47) 11.15
USOC 0.83 (0.70, 0.97) 21.01
0.79 (0.58, 1.06)
No degree
MCS 1.35 (0.84, 2.18) 7.93
NS 0.88 (0.45, 1.72) 4.72
BCS70 1.42 (0.95, 2.11) 10.08
NCDS 0.89 (0.59, 1.35) 9.71
USOC 0.92 (0.80, 1.07) 22.17
1.04 (0.85, 1.27)
Heterogeneity: τ² = 0.02, I² = 47.32%, H² = 1.90
Test of group differences: Qb(1) = 2.29, p = 0.13
1/4 1/2 1 2
Random-effects REML model

Figure set 14: More fruit and veg

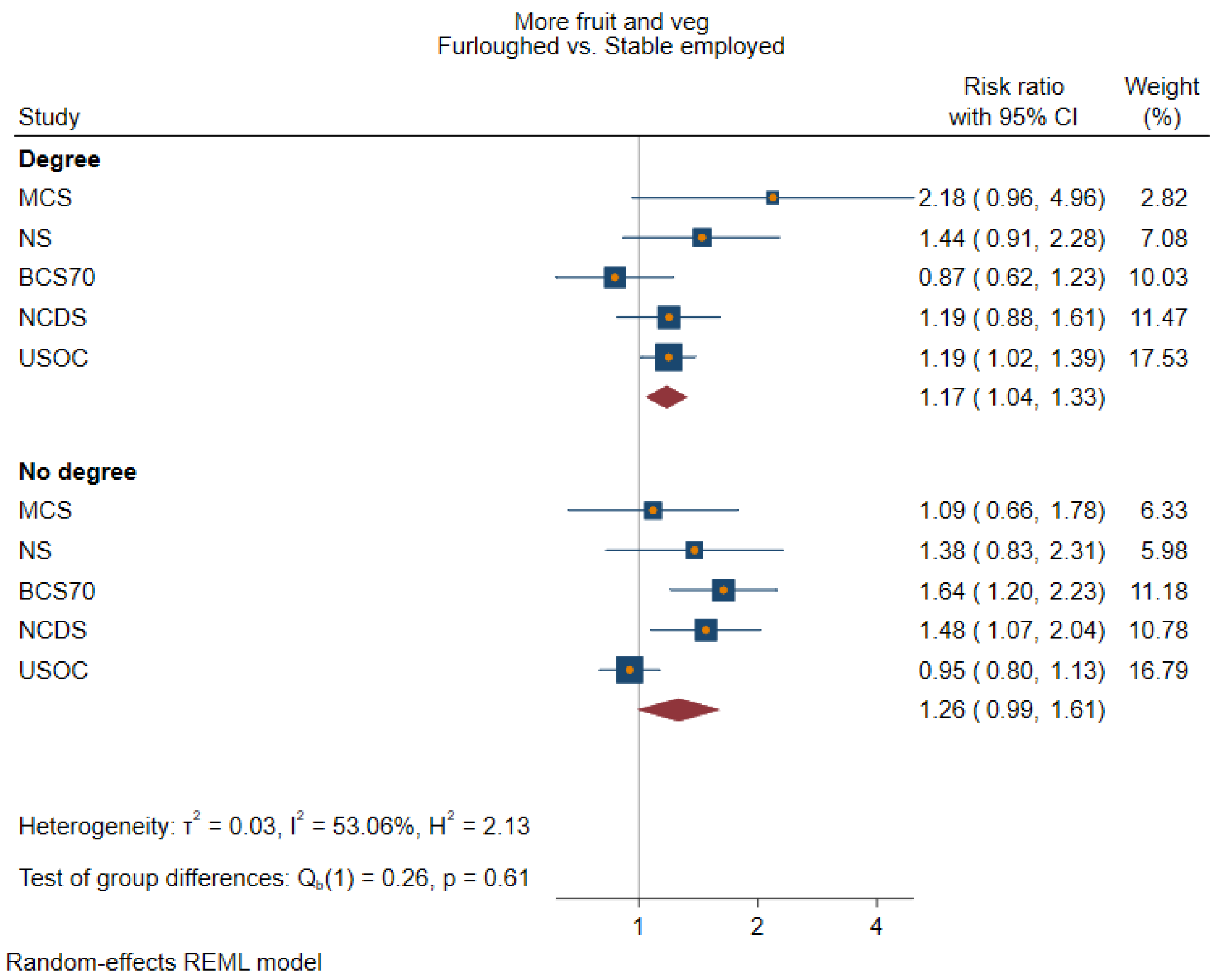

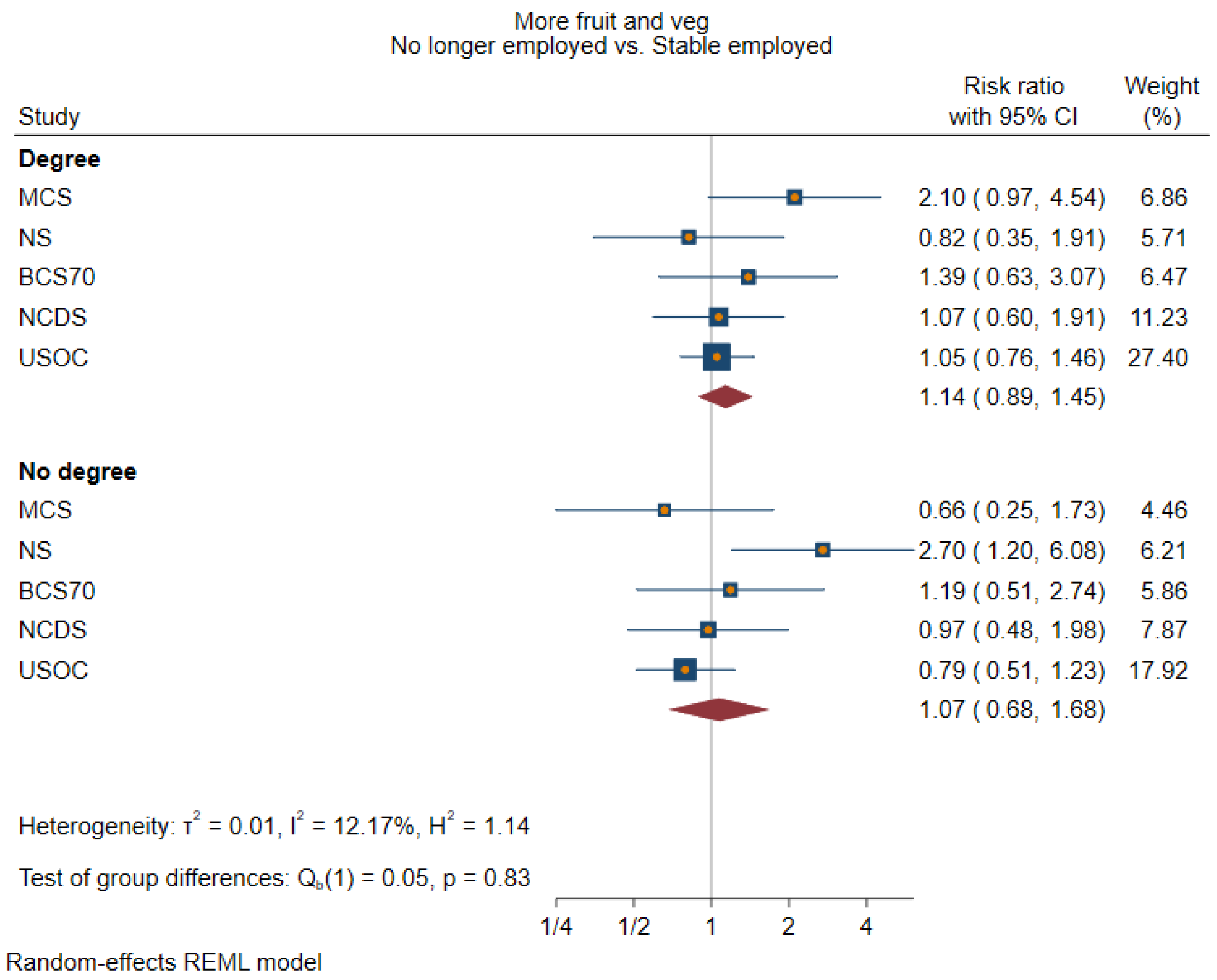

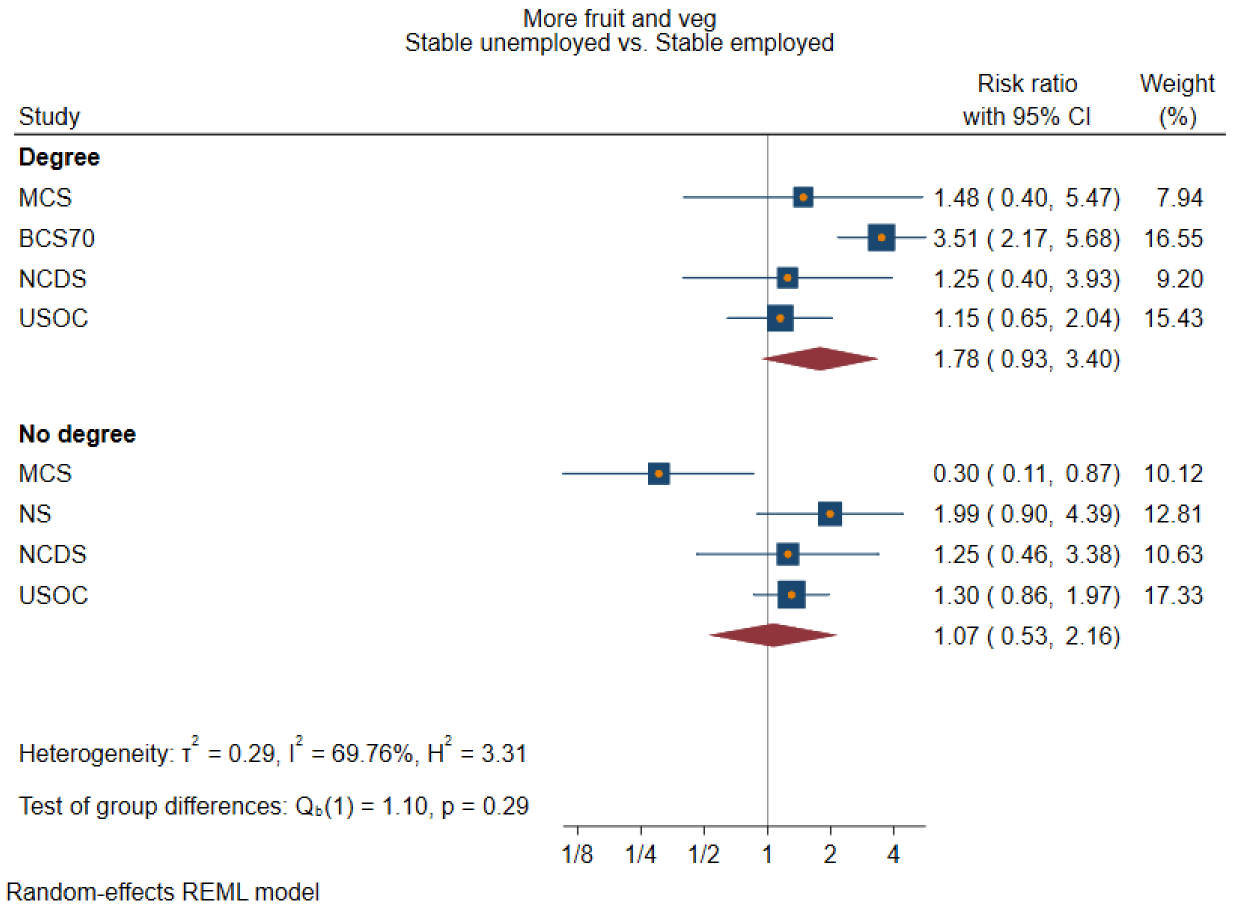
More fruit and veg
Stable unemployed vs. Stable employed
Study
Risk ratio with 95% CI
Weight (%)
Degree
MCS 1.48 (0.40, 5.47) 7.94
BCS70 3.51 (2.17, 5.68) 16.55
NCDS 1.25 (0.40, 3.93) 9.20
USOC 1.15 (0.65, 2.04) 15.43
1.78 (0.93, 3.40)
No degree
MCS 0.30 (0.11, 0.87) 10.12
NS 1.99 (0.90, 4.39) 12.81
NCDS 1.25 (0.46, 3.38) 10.63
USOC 1.30 (0.86, 1.97) 17.33
1.07 (0.53, 2.16)
Heterogeneity: τ^2 = 0.29, I^2 = 69.76%, H^2 = 3.31
Test of group differences: $Q_b(1)$ = 1.10, p = 0.29
1/8 1/4 1/2 1 2 4
Random-effects REML model

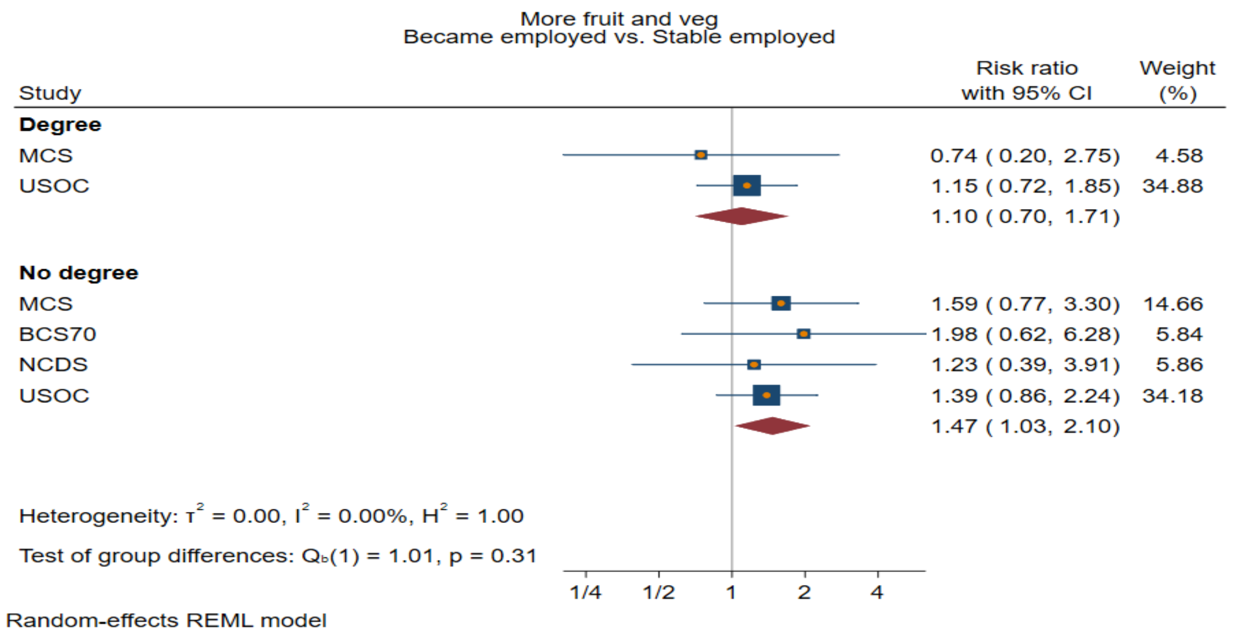
More fruit and veg
Became employed vs. Stable employed
Study
Risk ratio with 95% CI
Weight (%)
Degree
MCS 0.74 (0.20, 2.75) 4.58
USOC 1.15 (0.72, 1.85) 34.88
1.10 (0.70, 1.71)
No degree
MCS 1.59 (0.77, 3.30) 14.66
BCS70 1.98 (0.62, 6.28) 5.84
NCDS 1.23 (0.39, 3.91) 5.86
USOC 1.39 (0.86, 2.24) 34.18
1.47 (1.03, 2.10)
Heterogeneity: τ^2 = 0.00, I^2 = 0.00%, H^2 = 1.00
Test of group differences: $Q_b(1)$ = 1.01, p = 0.31
1/4 1/2 1 2 4
Random-effects REML model

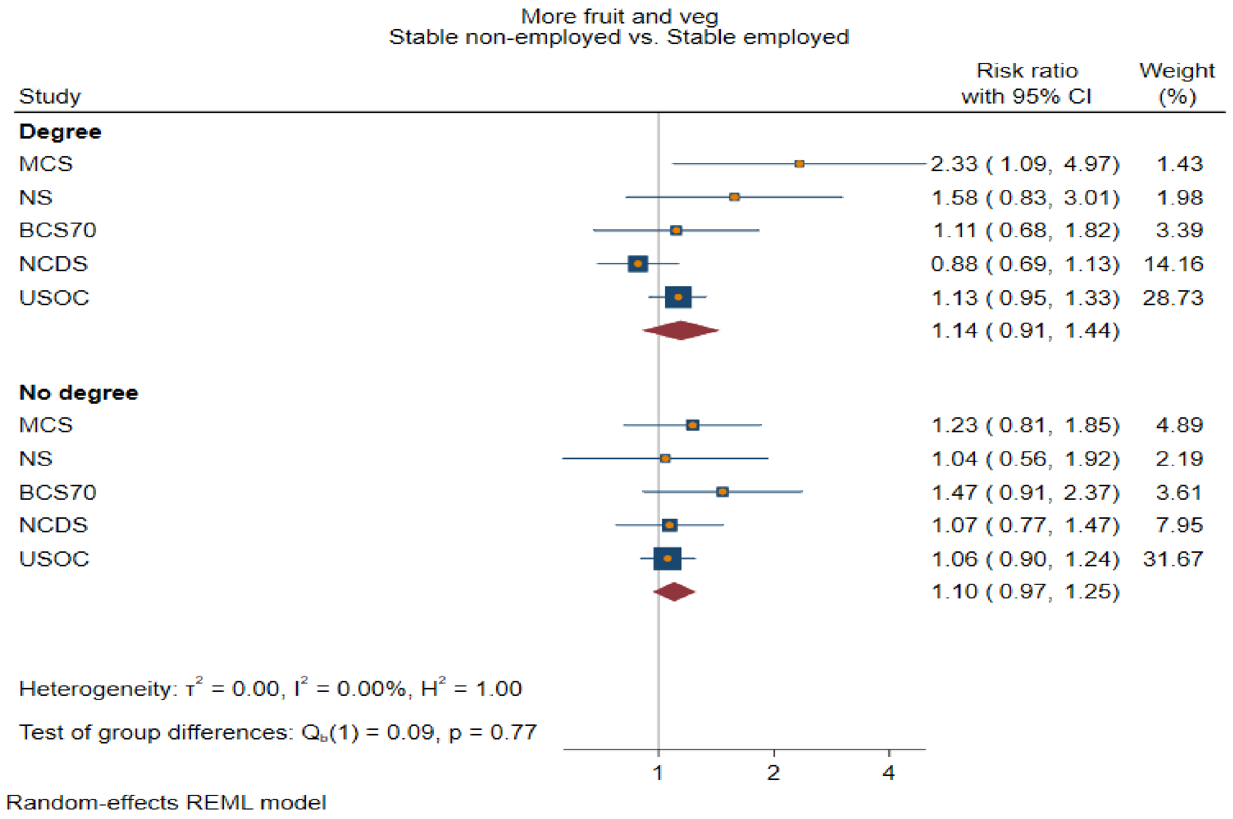
More fruit and veg
Stable non-employed vs. Stable employed
Study
Risk ratio with 95% CI
Weight (%)
Degree
MCS 2.33 (1.09, 4.97) 1.43
NS 1.58 (0.83, 3.01) 1.98
BCS70 1.11 (0.68, 1.82) 3.39
NCDS 0.88 (0.69, 1.13) 14.16
USOC 1.13 (0.95, 1.33) 28.73
1.14 (0.91, 1.44)
No degree
MCS 1.23 (0.81, 1.85) 4.89
NS 1.04 (0.56, 1.92) 2.19
BCS70 1.47 (0.91, 2.37) 3.61
NCDS 1.07 (0.77, 1.47) 7.95
USOC 1.06 (0.90, 1.24) 31.67
1.10 (0.97, 1.25)
Heterogeneity: τ^2 = 0.00, I^2 = 0.00%, H^2 = 1.00
Test of group differences: $Q_b(1)$ = 0.09, p = 0.77
1 2 4
Random-effects REML model

Figure set 15: Less than 3 days a week of at least 30min exercise

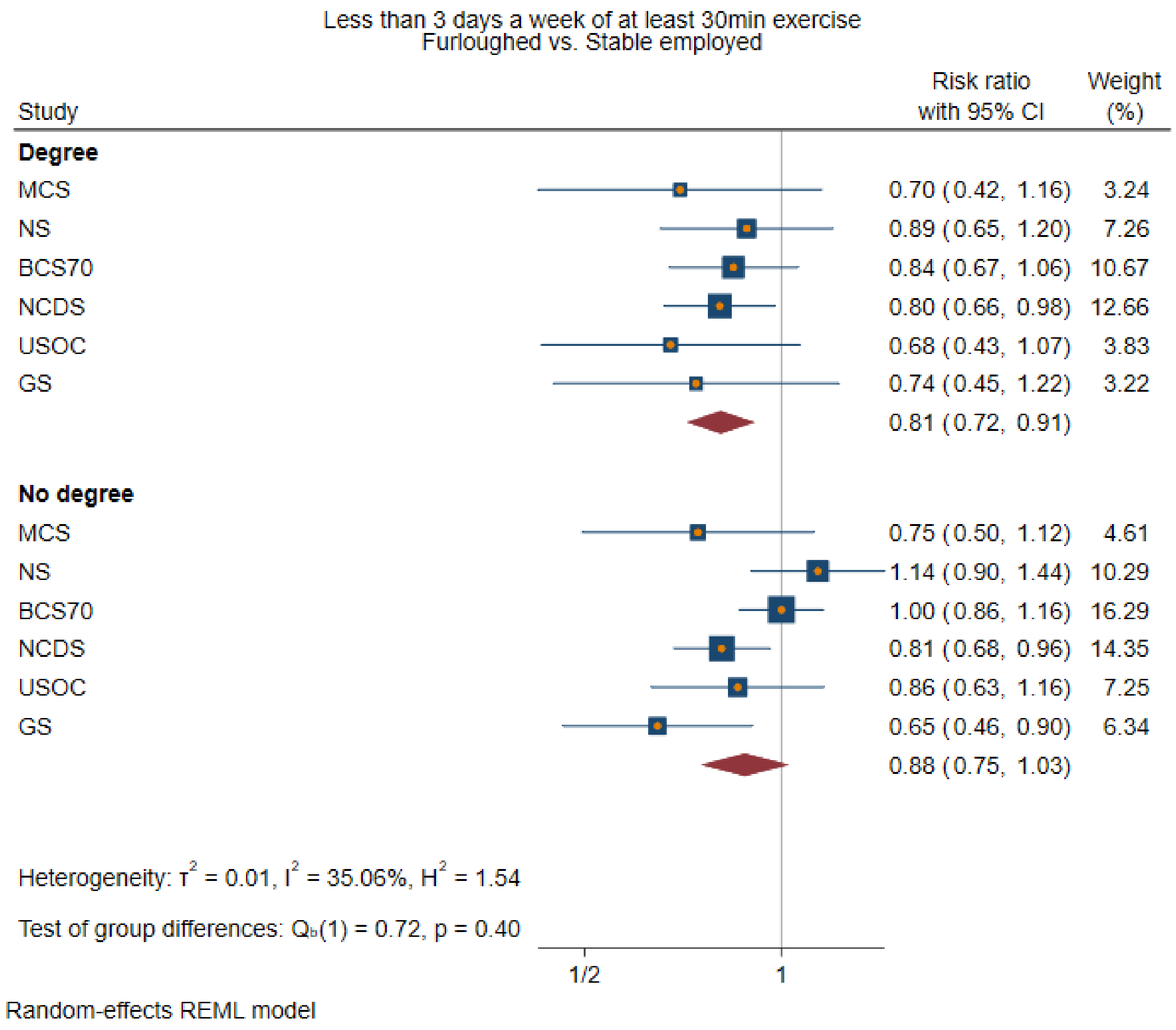

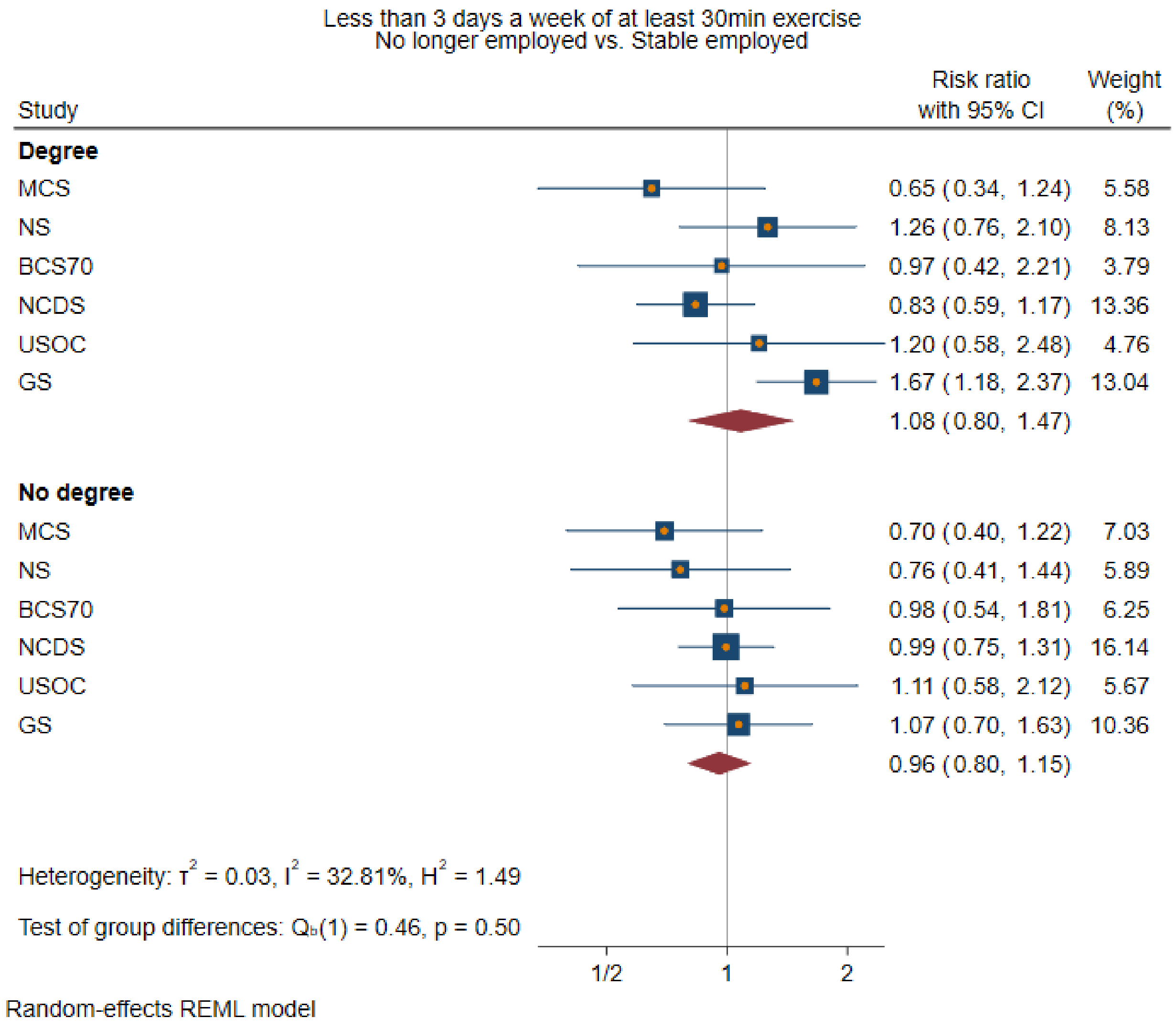

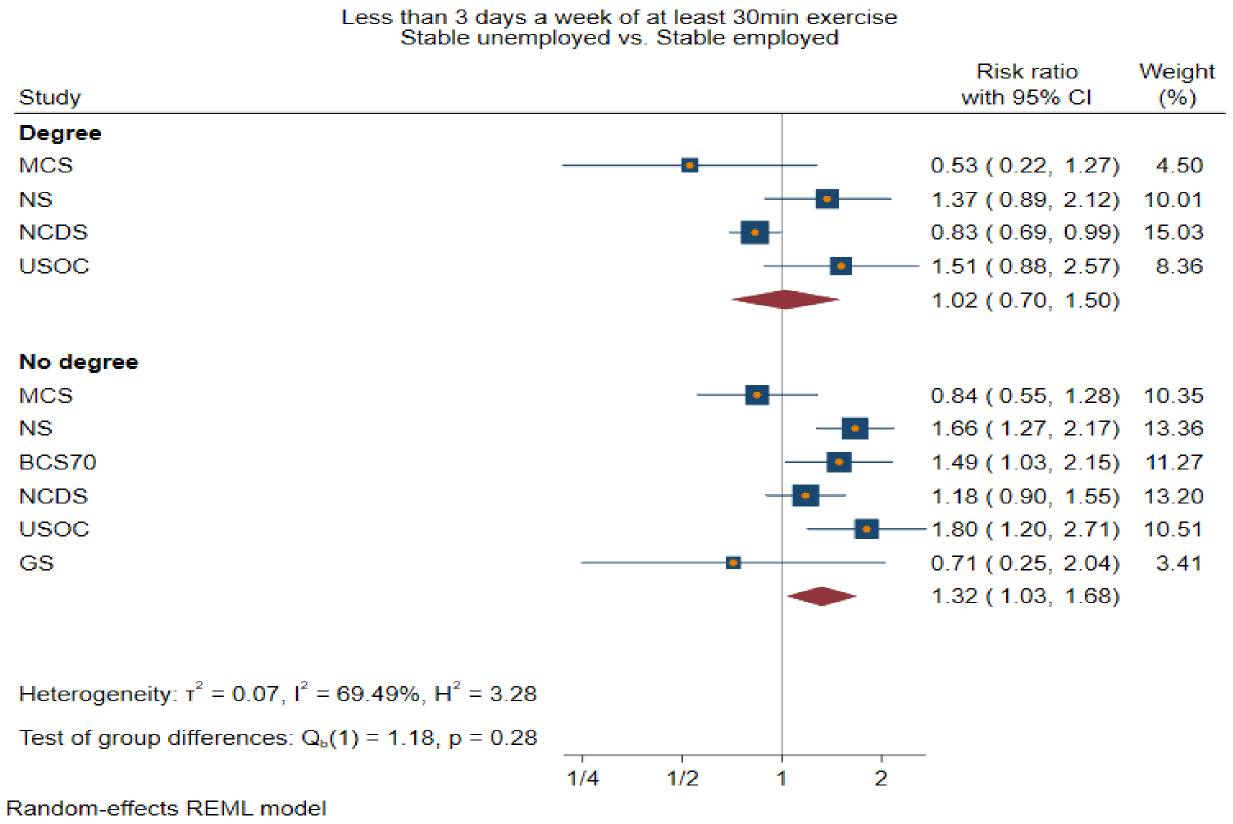
Less than 3 days a week of at least 30min exercise
Stable unemployed vs. Stable employed
Study
Risk ratio with 95% CI
Weight (%)
Degree
MCS 0.53 (0.22, 1.27) 4.50
NS 1.37 (0.89, 2.12) 10.01
NCDS 0.83 (0.69, 0.99) 15.03
USOC 1.51 (0.88, 2.57) 8.36
1.02 (0.70, 1.50)
No degree
MCS 0.84 (0.55, 1.28) 10.35
NS 1.66 (1.27, 2.17) 13.36
BCS70 1.49 (1.03, 2.15) 11.27
NCDS 1.18 (0.90, 1.55) 13.20
USOC 1.80 (1.20, 2.71) 10.51
GS 0.71 (0.25, 2.04) 3.41
1.32 (1.03, 1.68)
Heterogeneity: τ² = 0.07, I² = 69.49%, H² = 3.28
Test of group differences: Q_b(1) = 1.18, p = 0.28
1/4
1/2
1
2
Random-effects REML model

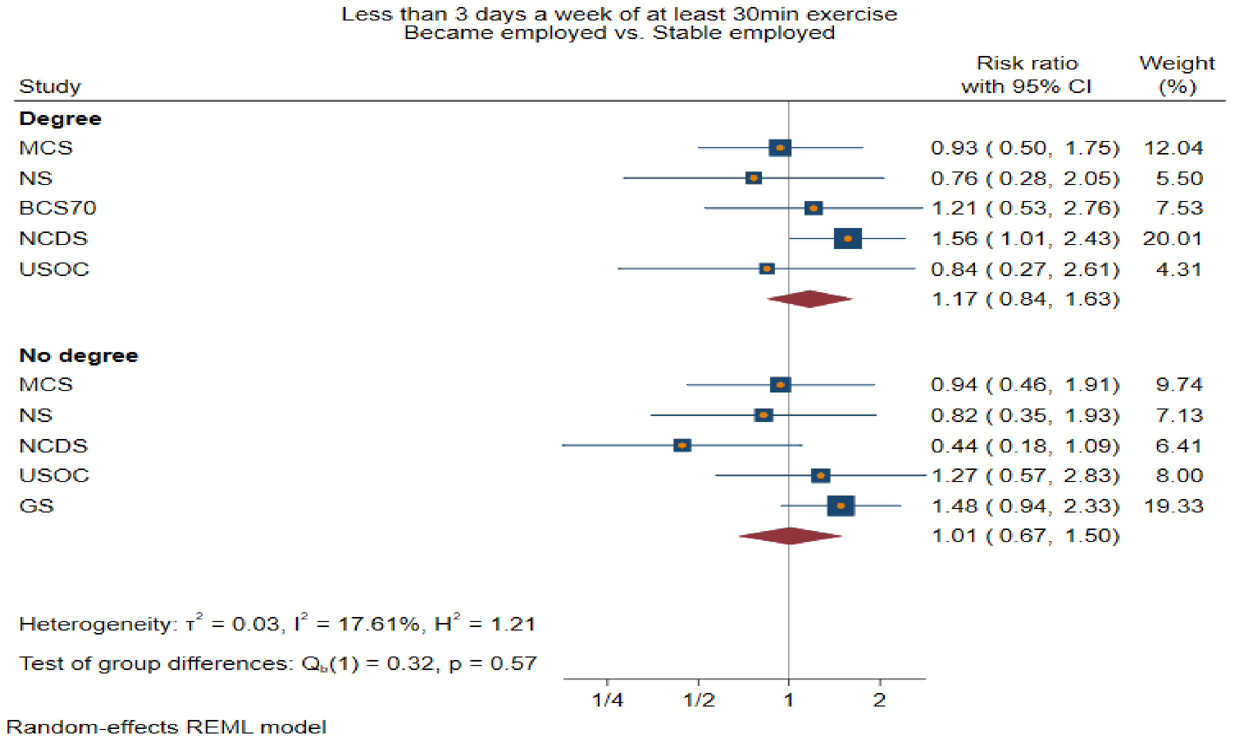
Less than 3 days a week of at least 30min exercise
Became employed vs. Stable employed
Study
Risk ratio with 95% CI
Weight (%)
Degree
MCS 0.93 (0.50, 1.75) 12.04
NS 0.76 (0.28, 2.05) 5.50
BCS70 1.21 (0.53, 2.76) 7.53
NCDS 1.56 (1.01, 2.43) 20.01
USOC 0.84 (0.27, 2.61) 4.31
1.17 (0.84, 1.63)
No degree
MCS 0.94 (0.46, 1.91) 9.74
NS 0.82 (0.35, 1.93) 7.13
NCDS 0.44 (0.18, 1.09) 6.41
USOC 1.27 (0.57, 2.83) 8.00
GS 1.48 (0.94, 2.33) 19.33
1.01 (0.67, 1.50)
Heterogeneity: τ² = 0.03, I² = 17.61%, H² = 1.21
Test of group differences: Q_b(1) = 0.32, p = 0.57
1/4
1/2
1
2
Random-effects REML model

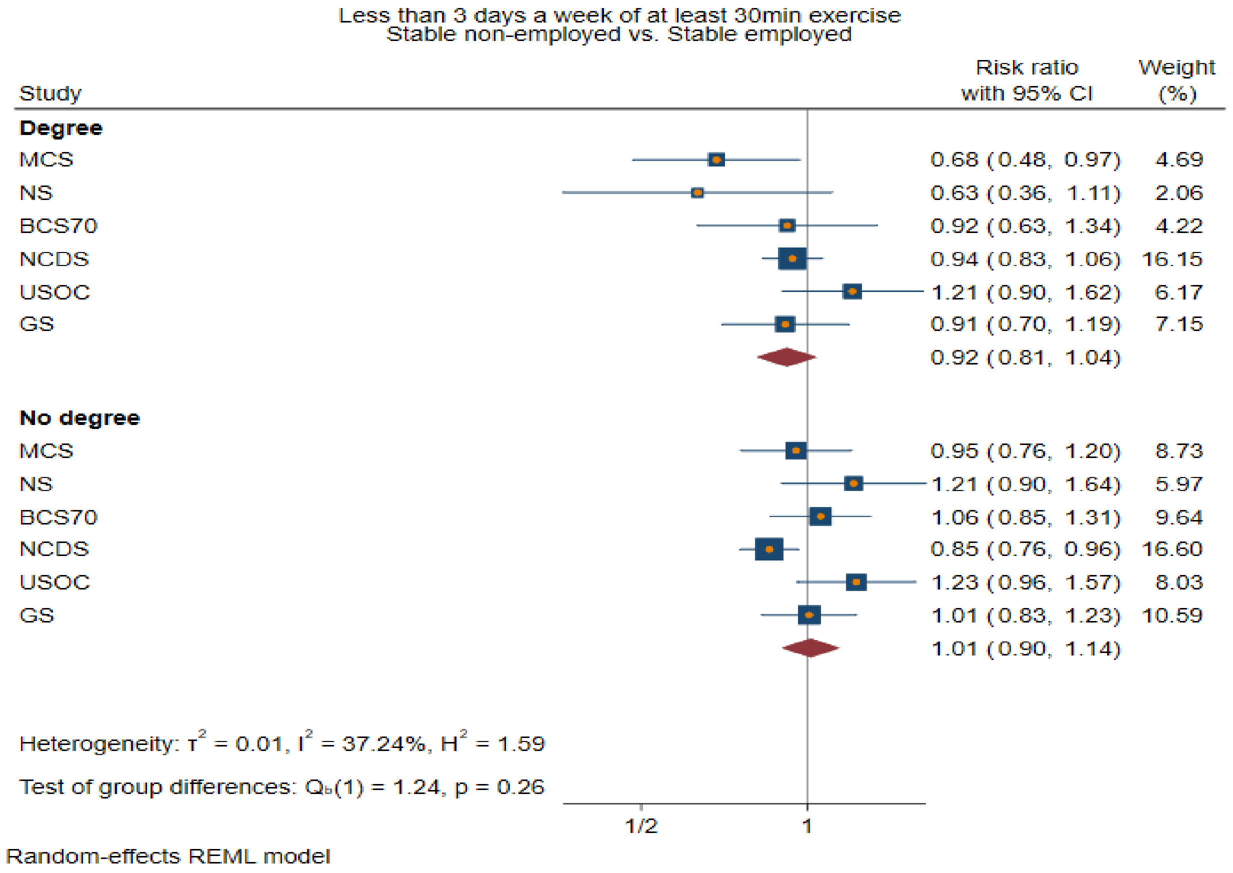
Less than 3 days a week of at least 30min exercise
Stable non-employed vs. Stable employed
Study
Risk ratio with 95% CI
Weight (%)
Degree
MCS 0.68 (0.48, 0.97) 4.69
NS 0.63 (0.36, 1.11) 2.06
BCS70 0.92 (0.63, 1.34) 4.22
NCDS 0.94 (0.83, 1.06) 16.15
USOC 1.21 (0.90, 1.62) 6.17
GS 0.91 (0.70, 1.19) 7.15
0.92 (0.81, 1.04)
No degree
MCS 0.95 (0.76, 1.20) 8.73
NS 1.21 (0.90, 1.64) 5.97
BCS70 1.06 (0.85, 1.31) 9.64
NCDS 0.85 (0.76, 0.96) 16.60
USOC 1.23 (0.96, 1.57) 8.03
GS 1.01 (0.83, 1.23) 10.59
1.01 (0.90, 1.14)
Heterogeneity: τ² = 0.01, I² = 37.24%, H² = 1.59
Test of group differences: Q_b(1) = 1.24, p = 0.26
1/2
1
Random-effects REML model

Figure set 16: Less time/ fewer days of physical exercise

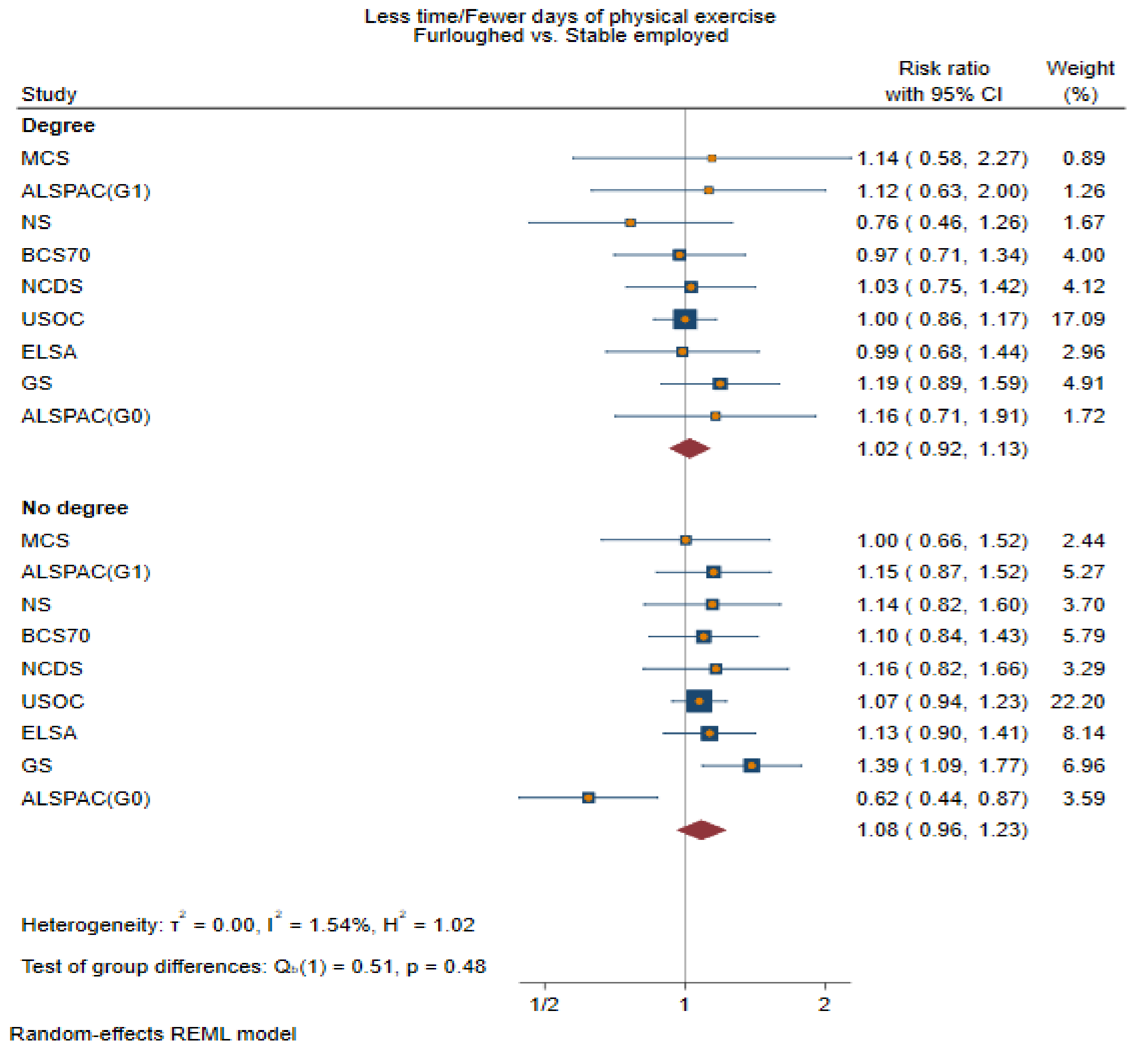

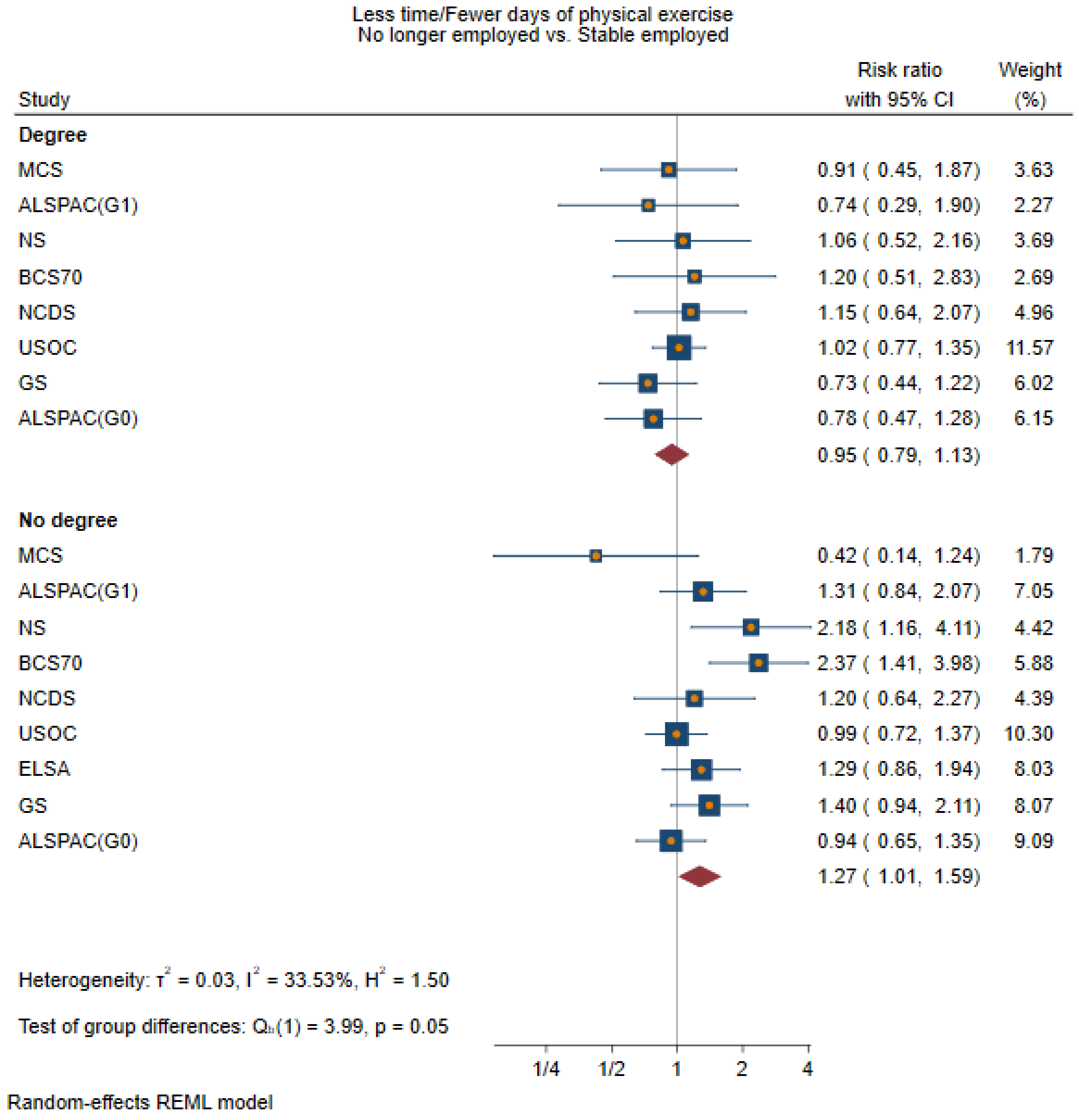

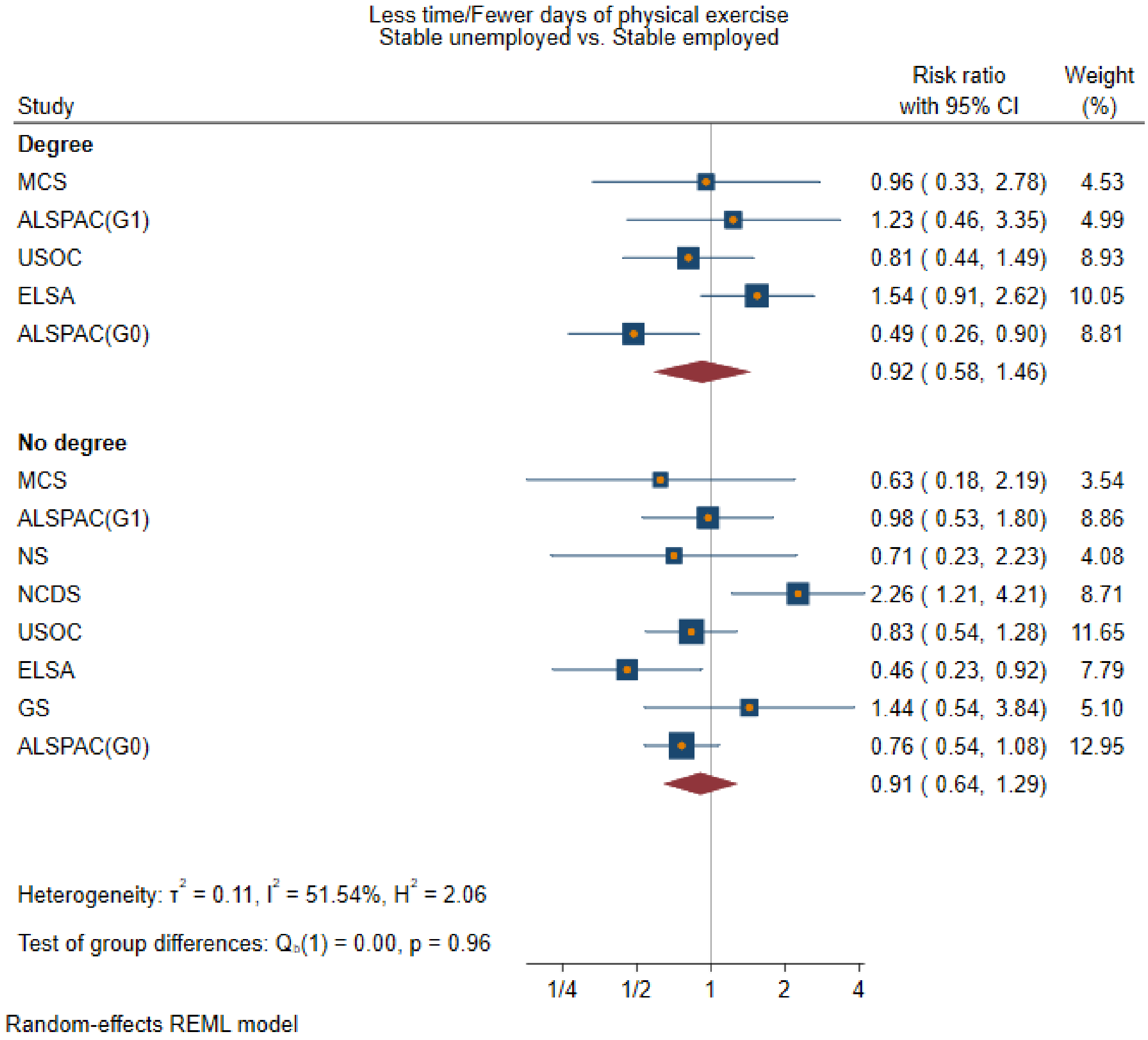
Less time/Fewer days of physical exercise
Stable unemployed vs. Stable employed
Study
Risk ratio with 95% CI
Weight (%)
Degree
MCS 0.96 (0.33, 2.78) 4.53
ALSPAC(G1) 1.23 (0.46, 3.35) 4.99
USOC 0.81 (0.44, 1.49) 8.93
ELSA 1.54 (0.91, 2.62) 10.05
ALSPAC(G0) 0.49 (0.26, 0.90) 8.81
0.92 (0.58, 1.46)
No degree
MCS 0.63 (0.18, 2.19) 3.54
ALSPAC(G1) 0.98 (0.53, 1.80) 8.86
NS 0.71 (0.23, 2.23) 4.08
NCDS 2.26 (1.21, 4.21) 8.71
USOC 0.83 (0.54, 1.28) 11.65
ELSA 0.46 (0.23, 0.92) 7.79
GS 1.44 (0.54, 3.84) 5.10
ALSPAC(G0) 0.76 (0.54, 1.08) 12.95
0.91 (0.64, 1.29)
Heterogeneity: τ² = 0.11, I² = 51.54%, H² = 2.06
Test of group differences: Q_b(1) = 0.00, p = 0.96
1/4
1/2
1
2
4
Random-effects REML model

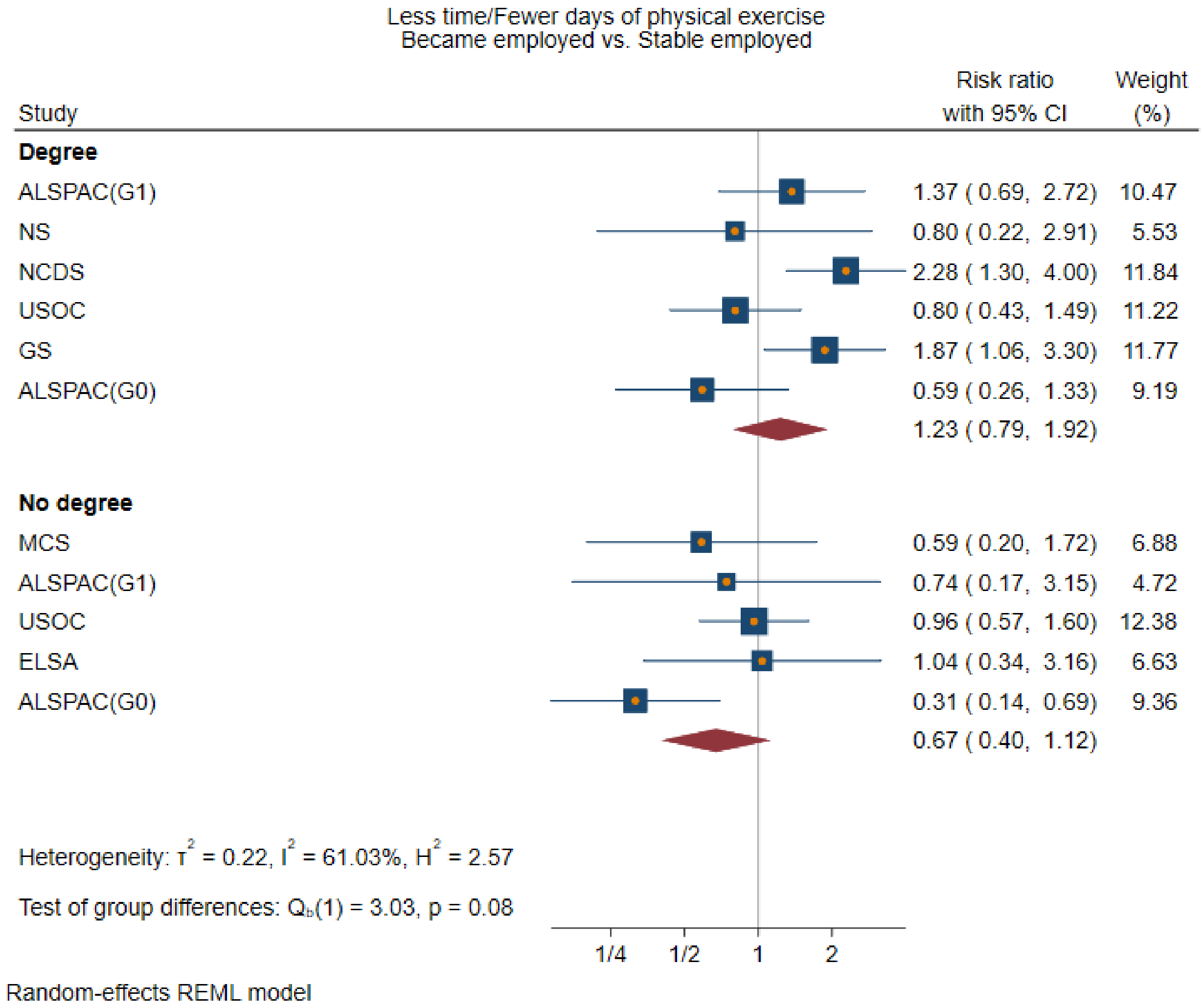
Less time/Fewer days of physical exercise
Became employed vs. Stable employed
Study
Risk ratio with 95% CI
Weight (%)
Degree
ALSPAC(G1) 1.37 (0.69, 2.72) 10.47
NS 0.80 (0.22, 2.91) 5.53
NCDS 2.28 (1.30, 4.00) 11.84
USOC 0.80 (0.43, 1.49) 11.22
GS 1.87 (1.06, 3.30) 11.77
ALSPAC(G0) 0.59 (0.26, 1.33) 9.19
1.23 (0.79, 1.92)
No degree
MCS 0.59 (0.20, 1.72) 6.88
ALSPAC(G1) 0.74 (0.17, 3.15) 4.72
USOC 0.96 (0.57, 1.60) 12.38
ELSA 1.04 (0.34, 3.16) 6.63
ALSPAC(G0) 0.31 (0.14, 0.69) 9.36
0.67 (0.40, 1.12)
Heterogeneity: τ² = 0.22, I² = 61.03%, H² = 2.57
Test of group differences: Q_b(1) = 3.03, p = 0.08
1/4
1/2
1
2
Random-effects REML model

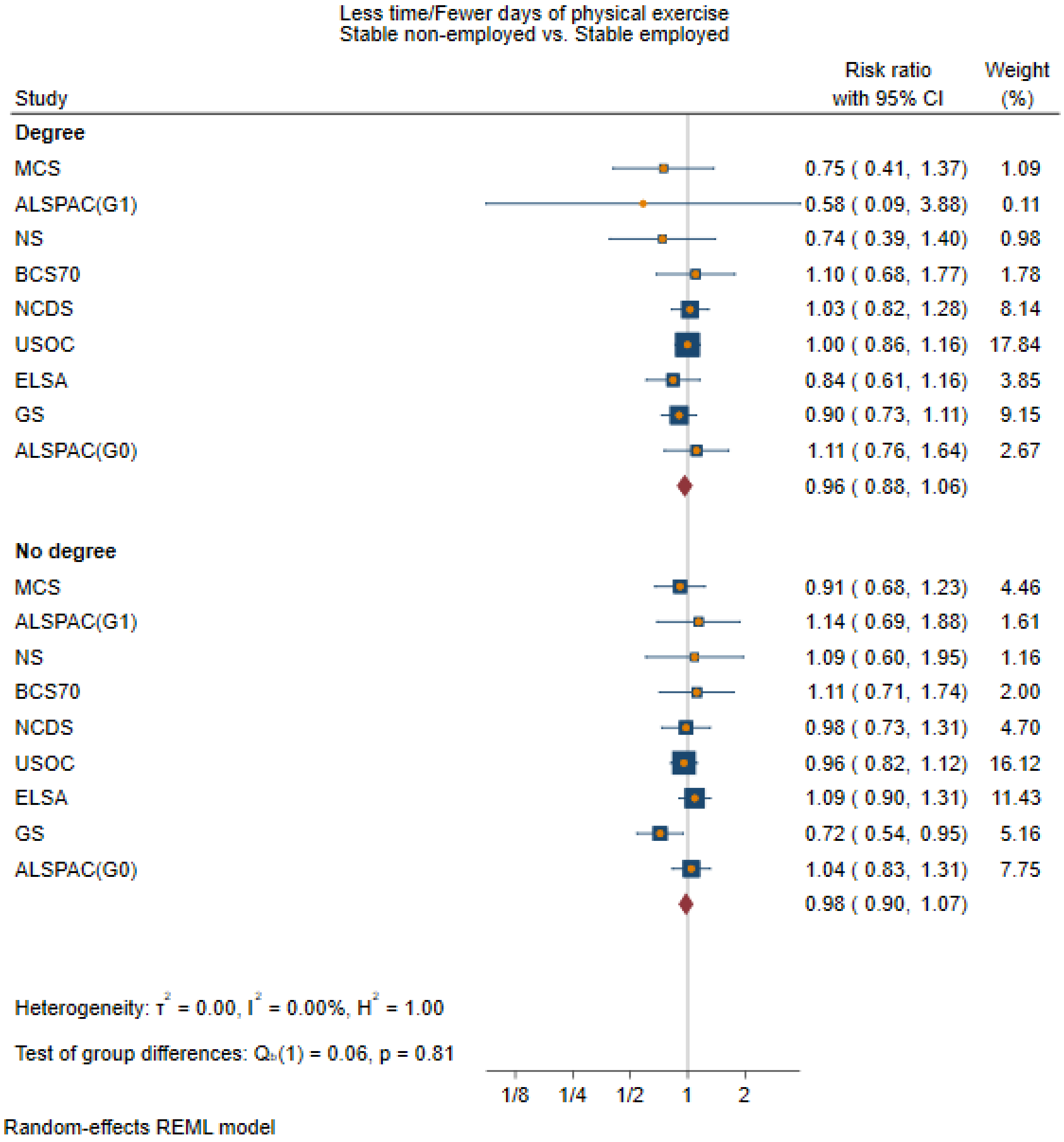
Less time/Fewer days of physical exercise
Stable non-employed vs. Stable employed
Study
Risk ratio with 95% CI
Weight (%)
Degree
MCS 0.75 (0.41, 1.37) 1.09
ALSPAC(G1) 0.58 (0.09, 3.88) 0.11
NS 0.74 (0.39, 1.40) 0.98
BCS70 1.10 (0.68, 1.77) 1.78
NCDS 1.03 (0.82, 1.28) 8.14
USOC 1.00 (0.86, 1.16) 17.84
ELSA 0.84 (0.61, 1.16) 3.85
GS 0.90 (0.73, 1.11) 9.15
ALSPAC(G0) 1.11 (0.76, 1.64) 2.67
0.96 (0.88, 1.06)
No degree
MCS 0.91 (0.68, 1.23) 4.46
ALSPAC(G1) 1.14 (0.69, 1.88) 1.61
NS 1.09 (0.60, 1.95) 1.16
BCS70 1.11 (0.71, 1.74) 2.00
NCDS 0.98 (0.73, 1.31) 4.70
USOC 0.96 (0.82, 1.12) 16.12
ELSA 1.09 (0.90, 1.31) 11.43
GS 0.72 (0.54, 0.95) 5.16
ALSPAC(G0) 1.04 (0.83, 1.31) 7.75
0.98 (0.90, 1.07)
Heterogeneity: τ² = 0.00, I² = 0.00%, H² = 1.00
Test of group differences: Qb(1) = 0.06, p = 0.81
1/8
1/4
1/2
1
2
Random-effects REML model

Figure set 17: More time/ days of physical exercise

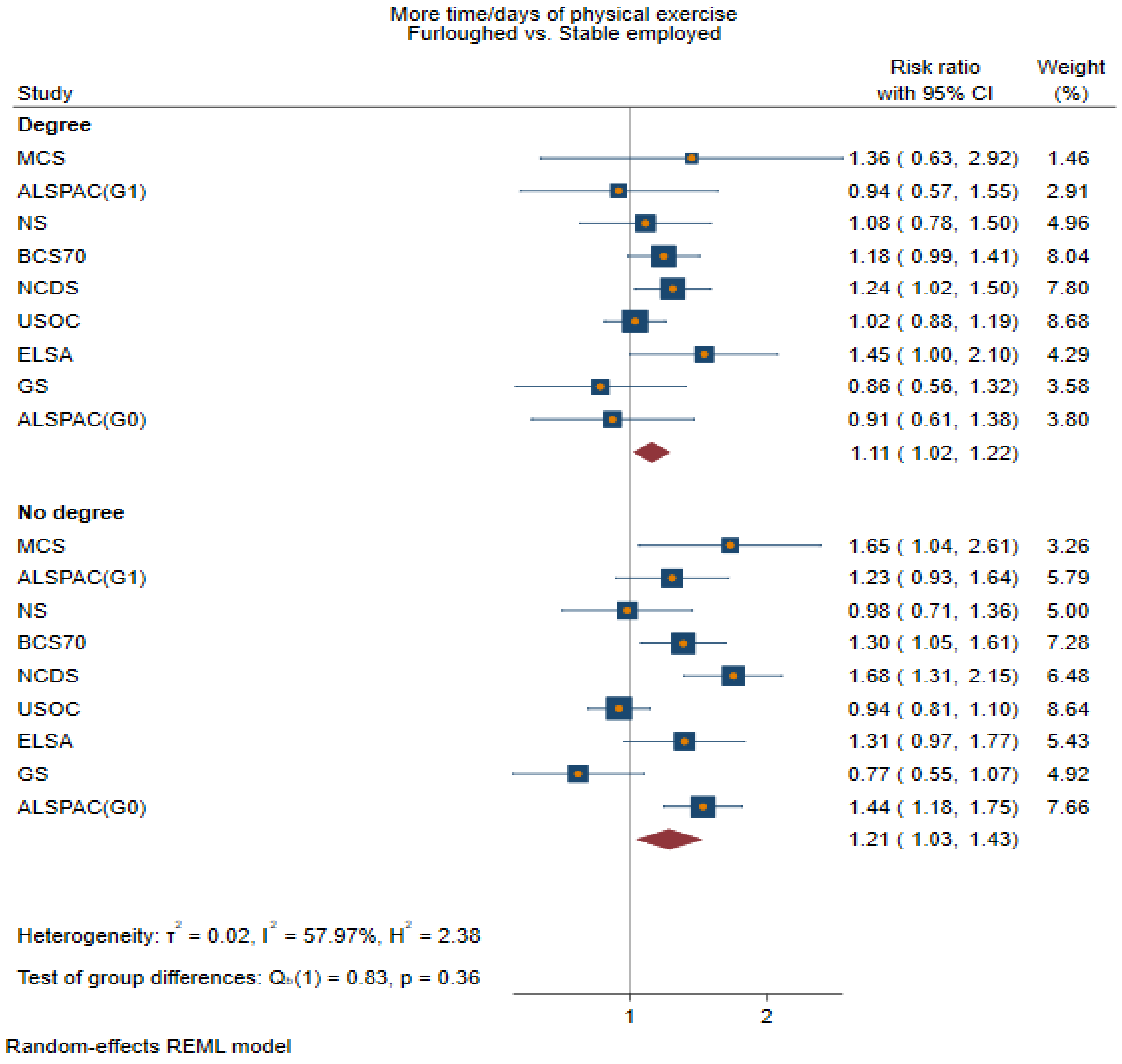

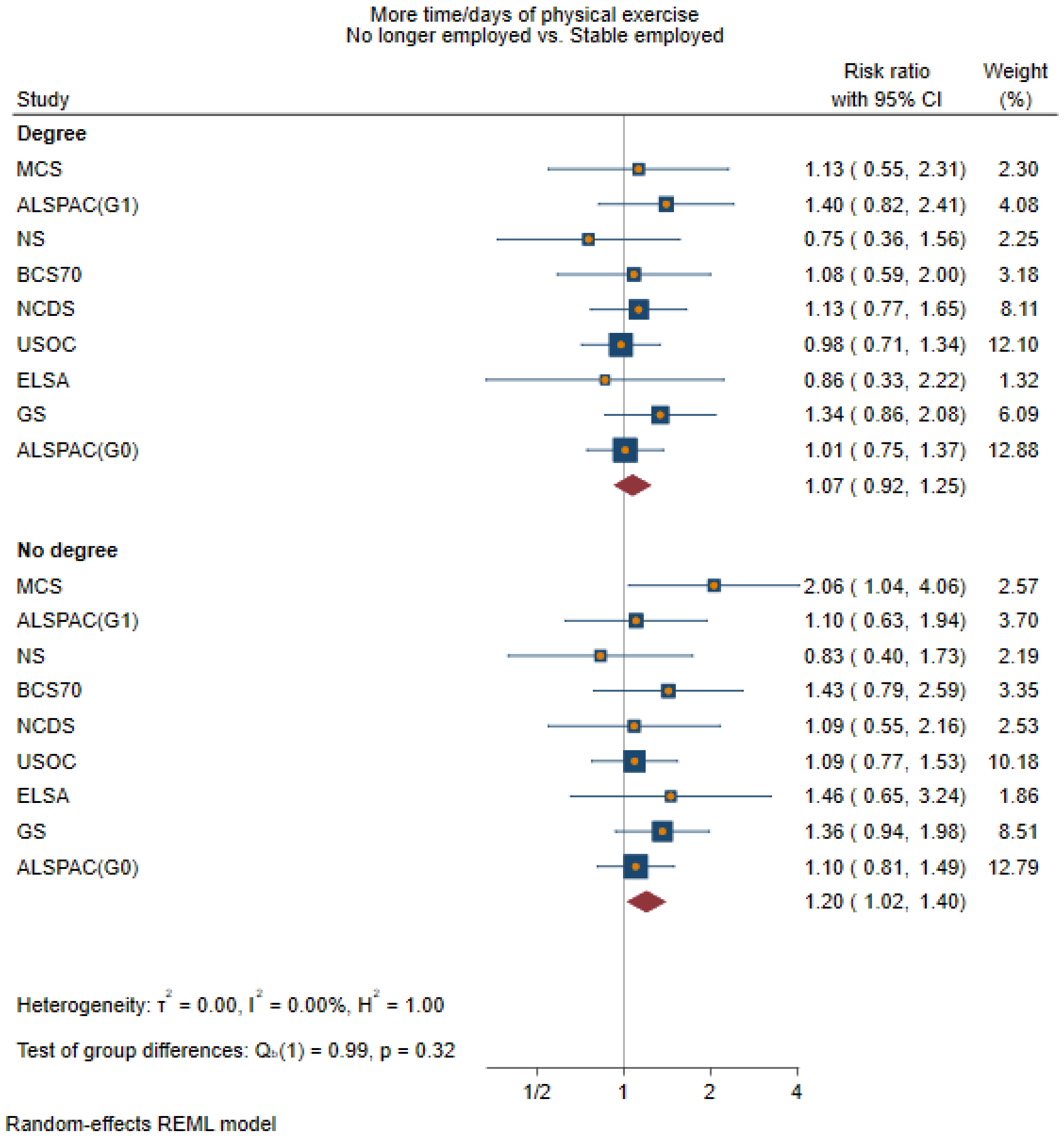

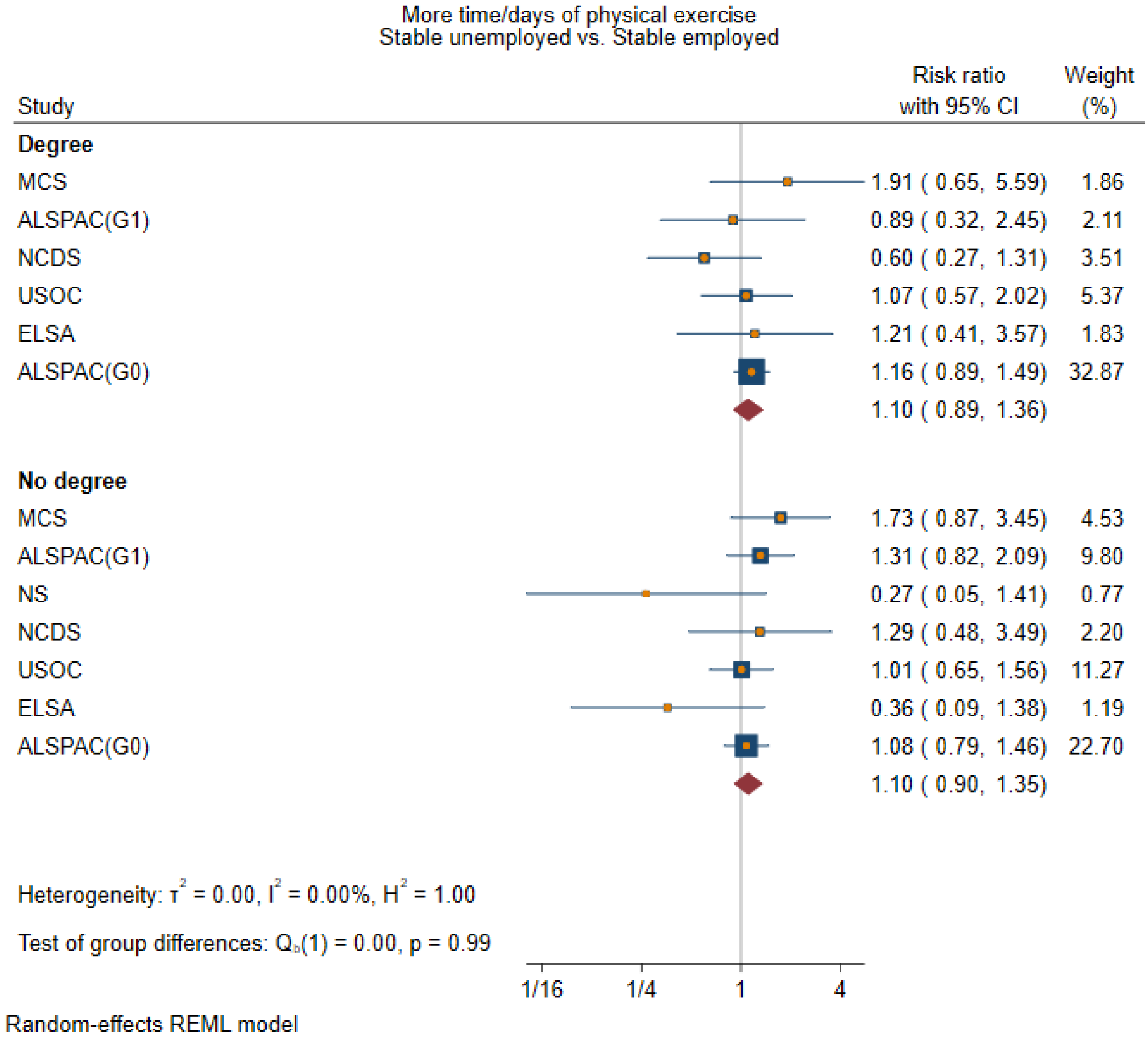
More time/days of physical exercise
Stable unemployed vs. Stable employed
Study
Risk ratio with 95% CI
Weight (%)
Degree
MCS 1.91 (0.65, 5.59) 1.86
ALSPAC(G1) 0.89 (0.32, 2.45) 2.11
NCDS 0.60 (0.27, 1.31) 3.51
USOC 1.07 (0.57, 2.02) 5.37
ELSA 1.21 (0.41, 3.57) 1.83
ALSPAC(G0) 1.16 (0.89, 1.49) 32.87
1.10 (0.89, 1.36)
No degree
MCS 1.73 (0.87, 3.45) 4.53
ALSPAC(G1) 1.31 (0.82, 2.09) 9.80
NS 0.27 (0.05, 1.41) 0.77
NCDS 1.29 (0.48, 3.49) 2.20
USOC 1.01 (0.65, 1.56) 11.27
ELSA 0.36 (0.09, 1.38) 1.19
ALSPAC(G0) 1.08 (0.79, 1.46) 22.70
1.10 (0.90, 1.35)
Heterogeneity: τ^2 = 0.00, I^2 = 0.00%, H^2 = 1.00
Test of group differences: $Q_b(1)$ = 0.00, p = 0.99
1/16
1/4
1
4
Random-effects REML model

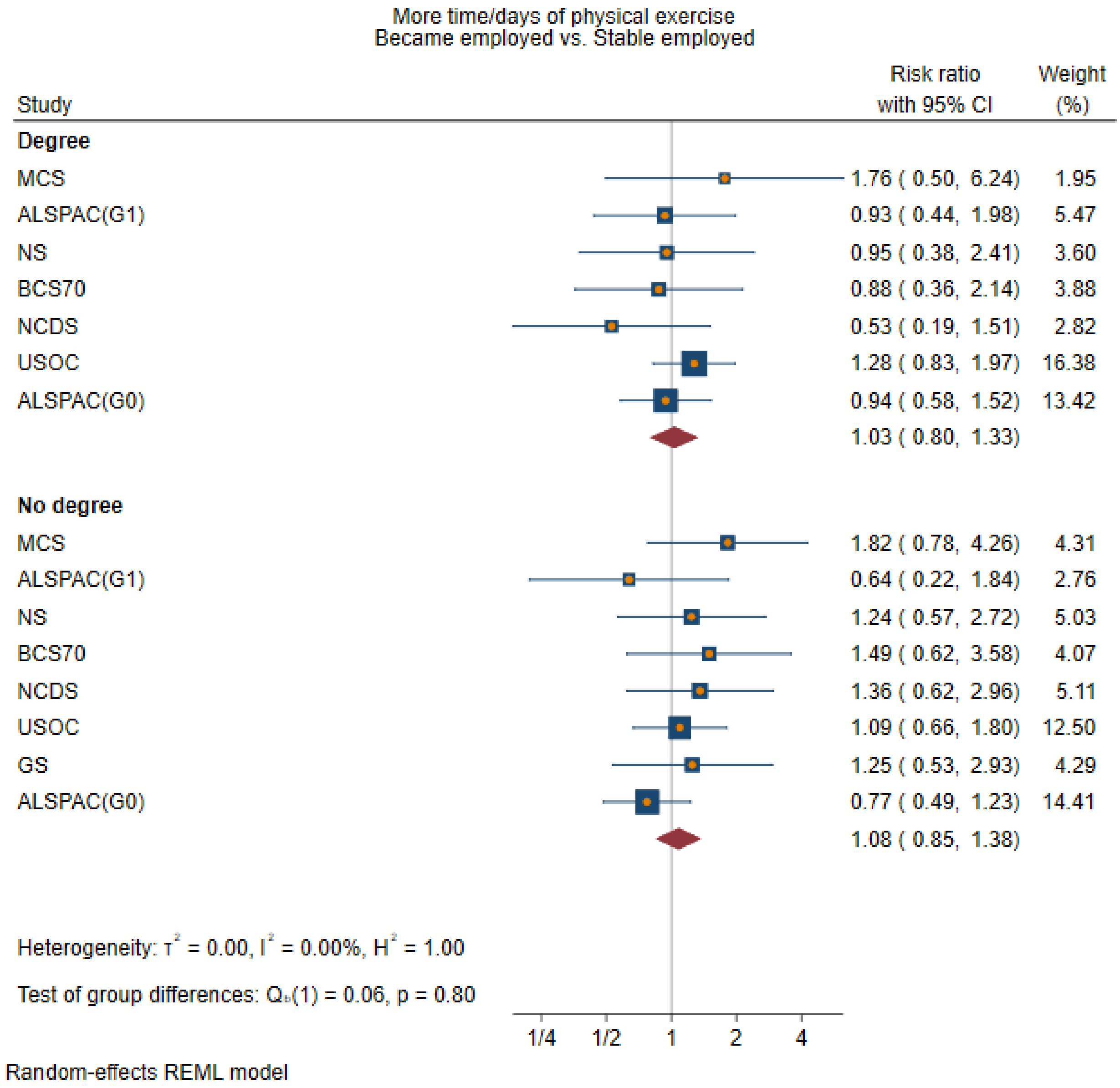
More time/days of physical exercise
Became employed vs. Stable employed
Study
Risk ratio with 95% CI
Weight (%)
Degree
MCS 1.76 (0.50, 6.24) 1.95
ALSPAC(G1) 0.93 (0.44, 1.98) 5.47
NS 0.95 (0.38, 2.41) 3.60
BCS70 0.88 (0.36, 2.14) 3.88
NCDS 0.53 (0.19, 1.51) 2.82
USOC 1.28 (0.83, 1.97) 16.38
ALSPAC(G0) 0.94 (0.58, 1.52) 13.42
1.03 (0.80, 1.33)
No degree
MCS 1.82 (0.78, 4.26) 4.31
ALSPAC(G1) 0.64 (0.22, 1.84) 2.76
NS 1.24 (0.57, 2.72) 5.03
BCS70 1.49 (0.62, 3.58) 4.07
NCDS 1.36 (0.62, 2.96) 5.11
USOC 1.09 (0.66, 1.80) 12.50
GS 1.25 (0.53, 2.93) 4.29
ALSPAC(G0) 0.77 (0.49, 1.23) 14.41
1.08 (0.85, 1.38)
Heterogeneity: τ^2 = 0.00, I^2 = 0.00%, H^2 = 1.00
Test of group differences: $Q_b(1)$ = 0.06, p = 0.80
1/4
1/2
1
2
4
Random-effects REML model

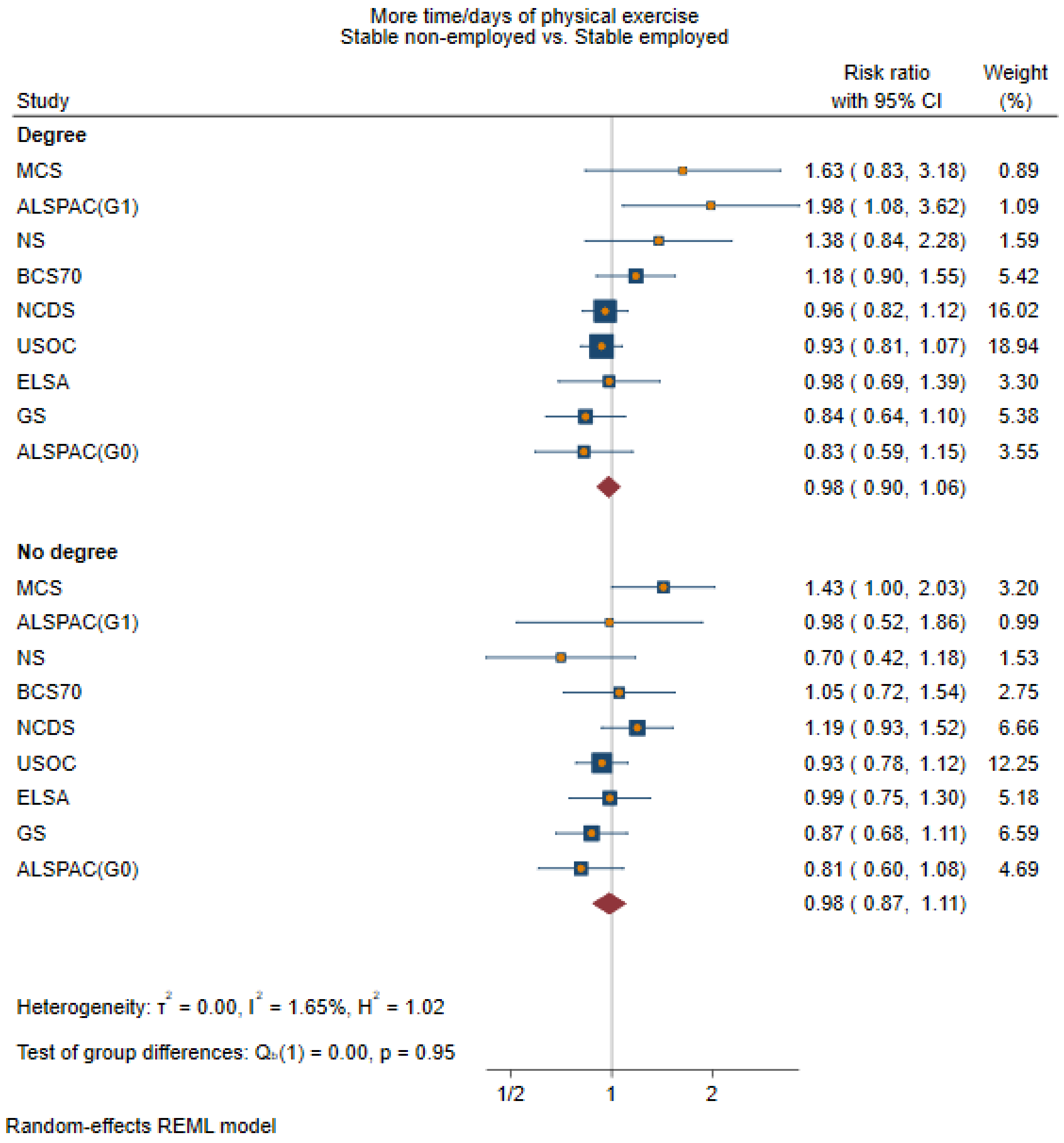

More time/days of physical exercise
Stable non-employed vs. Stable employed
Study
Risk ratio with 95% CI
Weight (%)
Degree
MCS 1.63 (0.83, 3.18) 0.89
ALSPAC(G1) 1.98 (1.08, 3.62) 1.09
NS 1.38 (0.84, 2.28) 1.59
BCS70 1.18 (0.90, 1.55) 5.42
NCDS 0.96 (0.82, 1.12) 16.02
USOC 0.93 (0.81, 1.07) 18.94
ELSA 0.98 (0.69, 1.39) 3.30
GS 0.84 (0.64, 1.10) 5.38
ALSPAC(G0) 0.83 (0.59, 1.15) 3.55
0.98 (0.90, 1.06)
No degree
MCS 1.43 (1.00, 2.03) 3.20
ALSPAC(G1) 0.98 (0.52, 1.86) 0.99
NS 0.70 (0.42, 1.18) 1.53
BCS70 1.05 (0.72, 1.54) 2.75
NCDS 1.19 (0.93, 1.52) 6.66
USOC 0.93 (0.78, 1.12) 12.25
ELSA 0.99 (0.75, 1.30) 5.18
GS 0.87 (0.68, 1.11) 6.59
ALSPAC(G0) 0.81 (0.60, 1.08) 4.69
0.98 (0.87, 1.11)
Heterogeneity: τ^2 = 0.00, I^2 = 1.65%, H^2 = 1.02
Test of group differences: $Q_b(1)$ = 0.00, p = 0.95
1/2
1
2
Random-effects REML model

Figure set 18: Sleeps outside ‘normal range’ (i.e. <6 or 9+ hours)

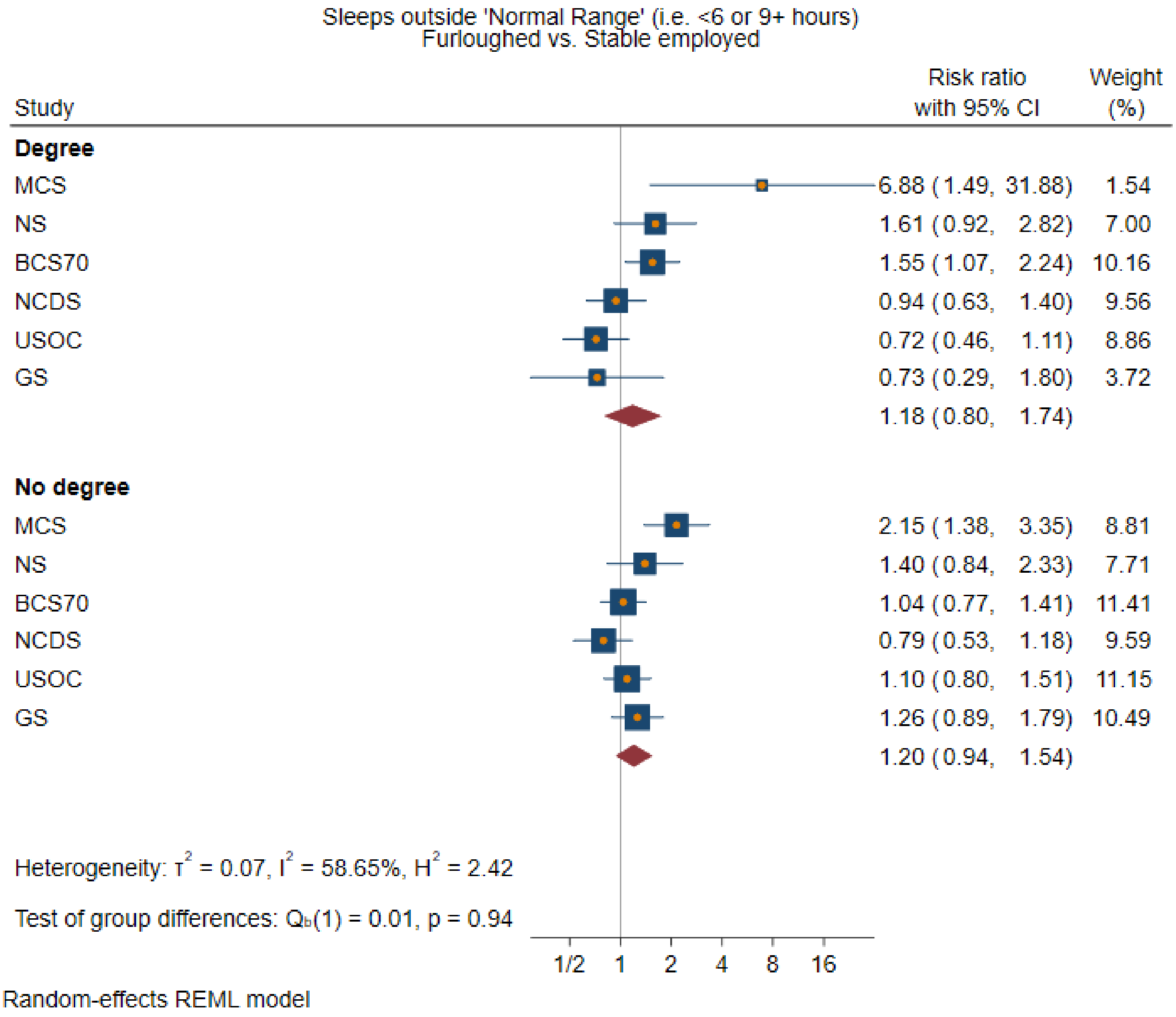

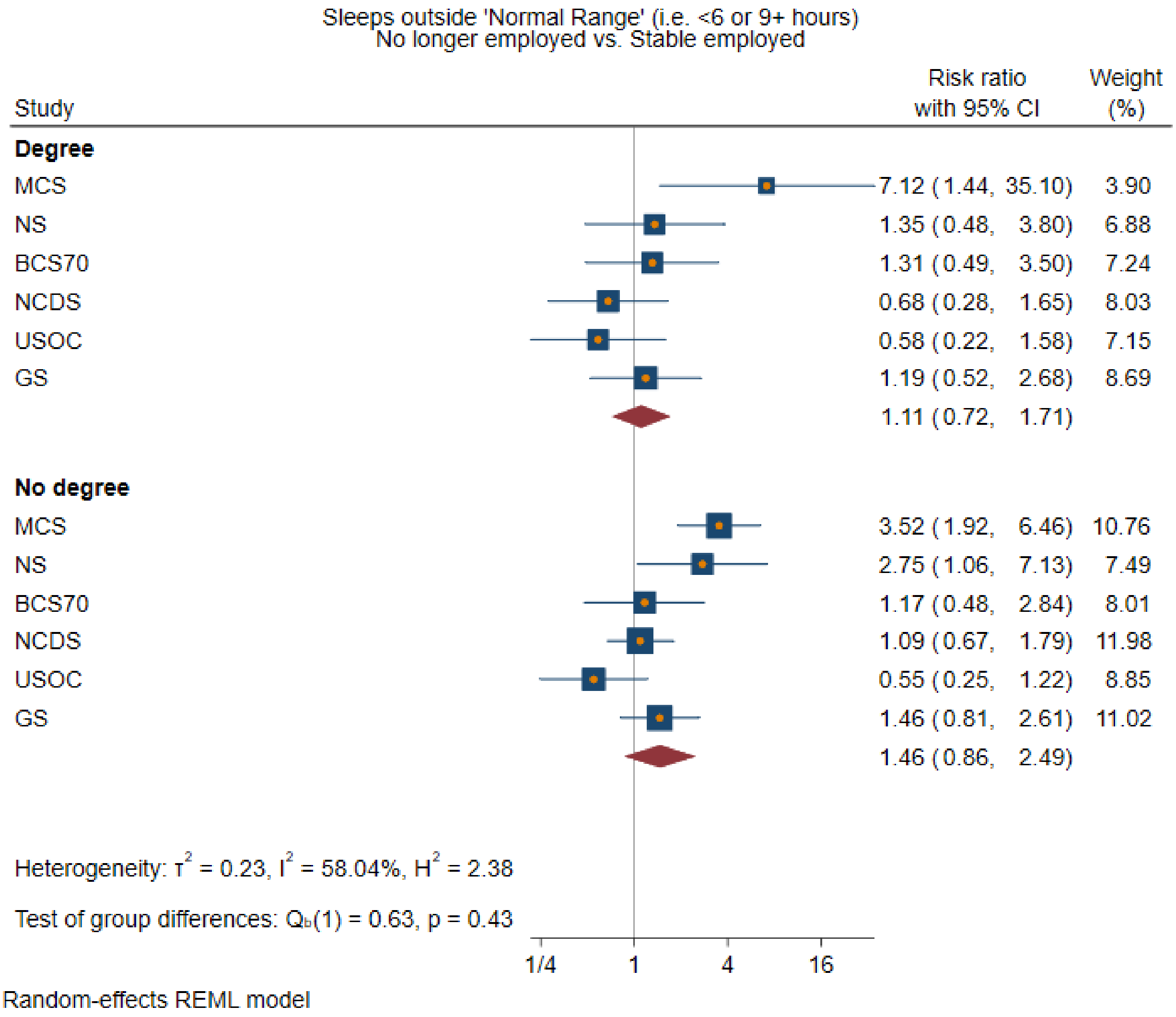

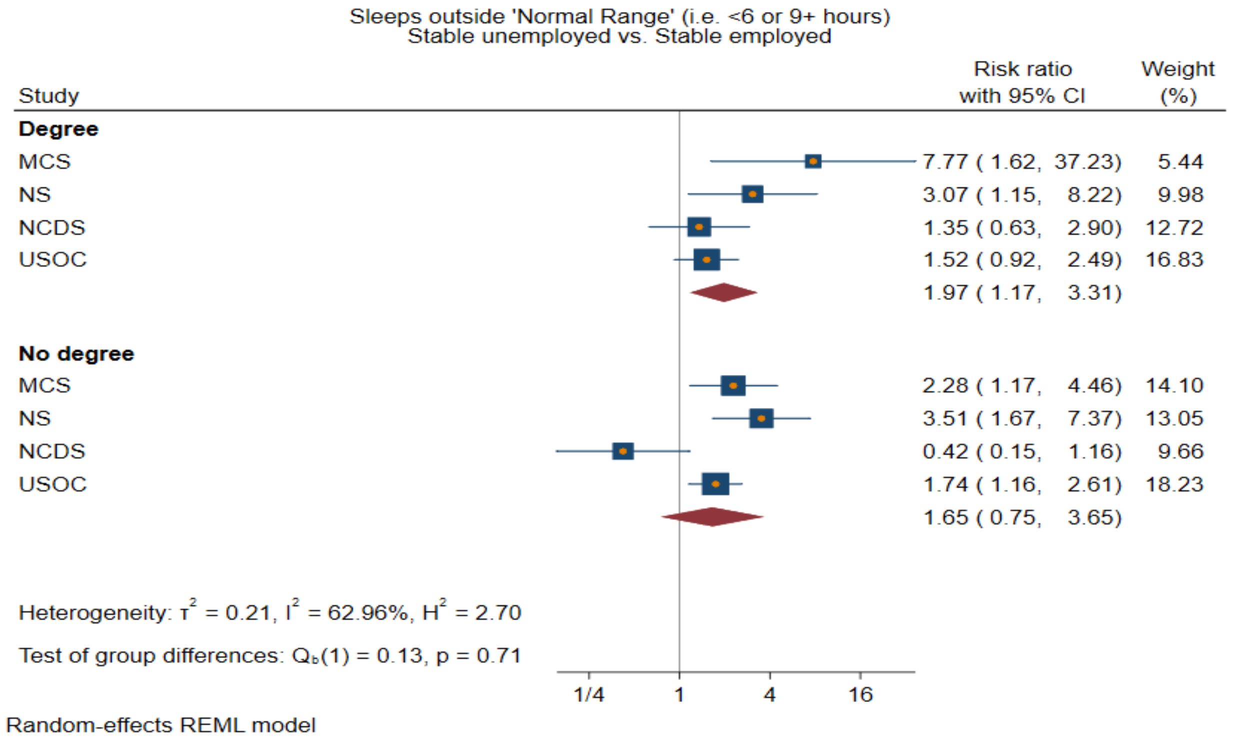
Sleeps outside 'Normal Range' (i.e. <6 or 9+ hours)
Stable unemployed vs. Stable employed
Study
Risk ratio with 95% CI
Weight (%)
Degree
MCS 7.77 (1.62, 37.23) 5.44
NS 3.07 (1.15, 8.22) 9.98
NCDS 1.35 (0.63, 2.90) 12.72
USOC 1.52 (0.92, 2.49) 16.83
1.97 (1.17, 3.31)
No degree
MCS 2.28 (1.17, 4.46) 14.10
NS 3.51 (1.67, 7.37) 13.05
NCDS 0.42 (0.15, 1.16) 9.66
USOC 1.74 (1.16, 2.61) 18.23
1.65 (0.75, 3.65)
Heterogeneity: τ² = 0.21, I² = 62.96%, H² = 2.70
Test of group differences: Qb(1) = 0.13, p = 0.71
1/4
1
4
16
Random-effects REML model

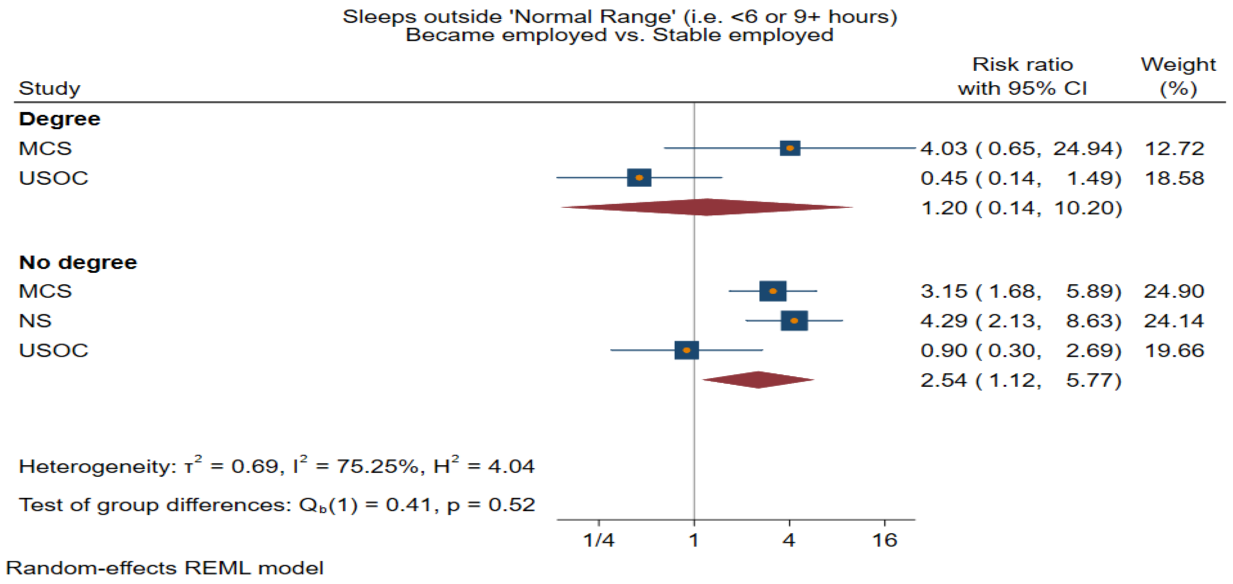
Sleeps outside 'Normal Range' (i.e. <6 or 9+ hours)
Became employed vs. Stable employed
Study
Risk ratio with 95% CI
Weight (%)
Degree
MCS 4.03 (0.65, 24.94) 12.72
USOC 0.45 (0.14, 1.49) 18.58
1.20 (0.14, 10.20)
No degree
MCS 3.15 (1.68, 5.89) 24.90
NS 4.29 (2.13, 8.63) 24.14
USOC 0.90 (0.30, 2.69) 19.66
2.54 (1.12, 5.77)
Heterogeneity: τ² = 0.69, I² = 75.25%, H² = 4.04
Test of group differences: Qb(1) = 0.41, p = 0.52
1/4
1
4
16
Random-effects REML model

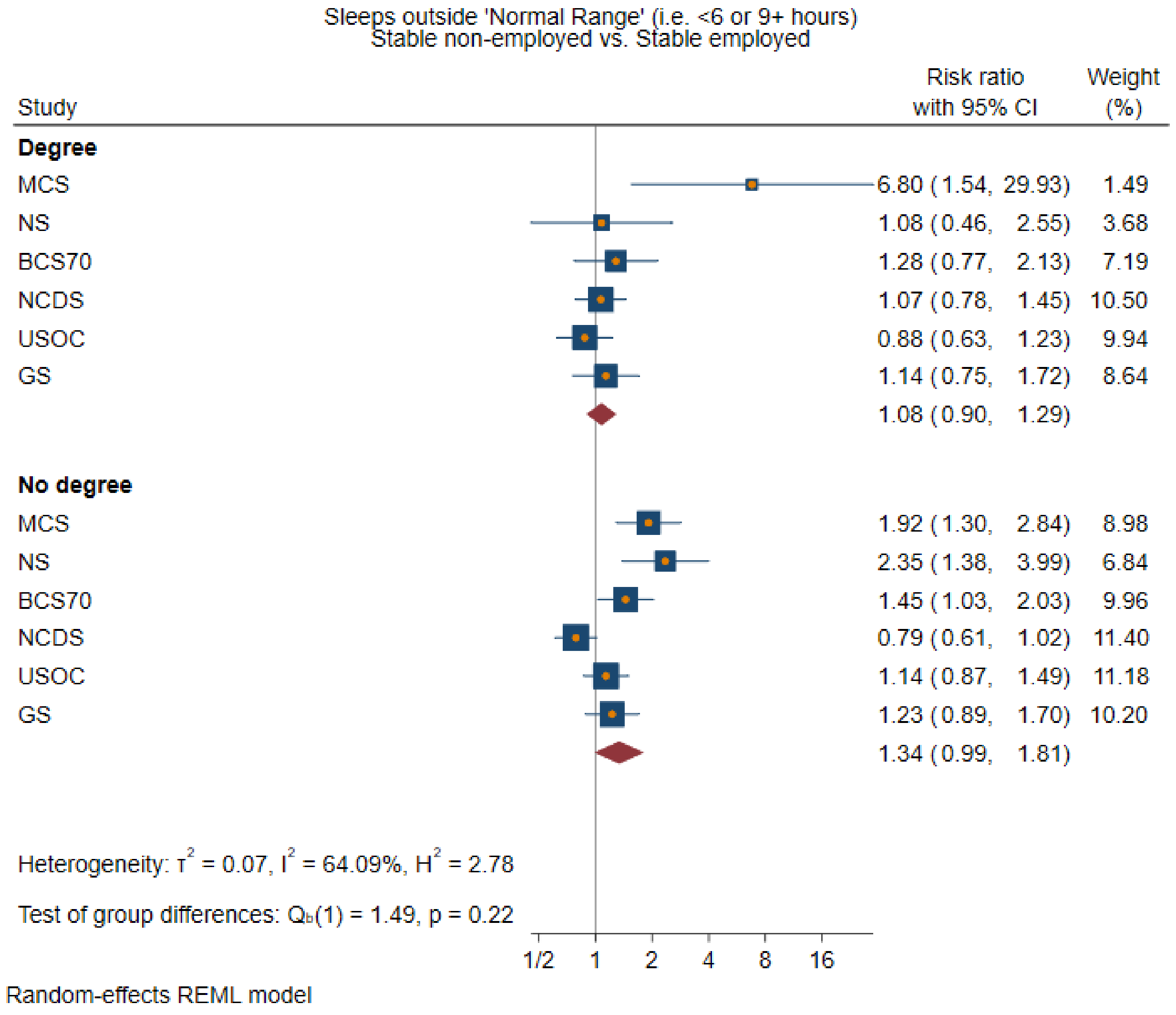
Sleeps outside 'Normal Range' (i.e. <6 or 9+ hours)
Stable non-employed vs. Stable employed
Study
Risk ratio with 95% CI
Weight (%)
Degree
MCS 6.80 (1.54, 29.93) 1.49
NS 1.08 (0.46, 2.55) 3.68
BCS70 1.28 (0.77, 2.13) 7.19
NCDS 1.07 (0.78, 1.45) 10.50
USOC 0.88 (0.63, 1.23) 9.94
GS 1.14 (0.75, 1.72) 8.64
1.08 (0.90, 1.29)
No degree
MCS 1.92 (1.30, 2.84) 8.98
NS 2.35 (1.38, 3.99) 6.84
BCS70 1.45 (1.03, 2.03) 9.96
NCDS 0.79 (0.61, 1.02) 11.40
USOC 1.14 (0.87, 1.49) 11.18
GS 1.23 (0.89, 1.70) 10.20
1.34 (0.99, 1.81)
Heterogeneity: τ² = 0.07, I² = 64.09%, H² = 2.78
Test of group differences: Qb(1) = 1.49, p = 0.22
1/2
1
2
4
8
16
Random-effects REML model

Figure set 19: Sleeps less than before

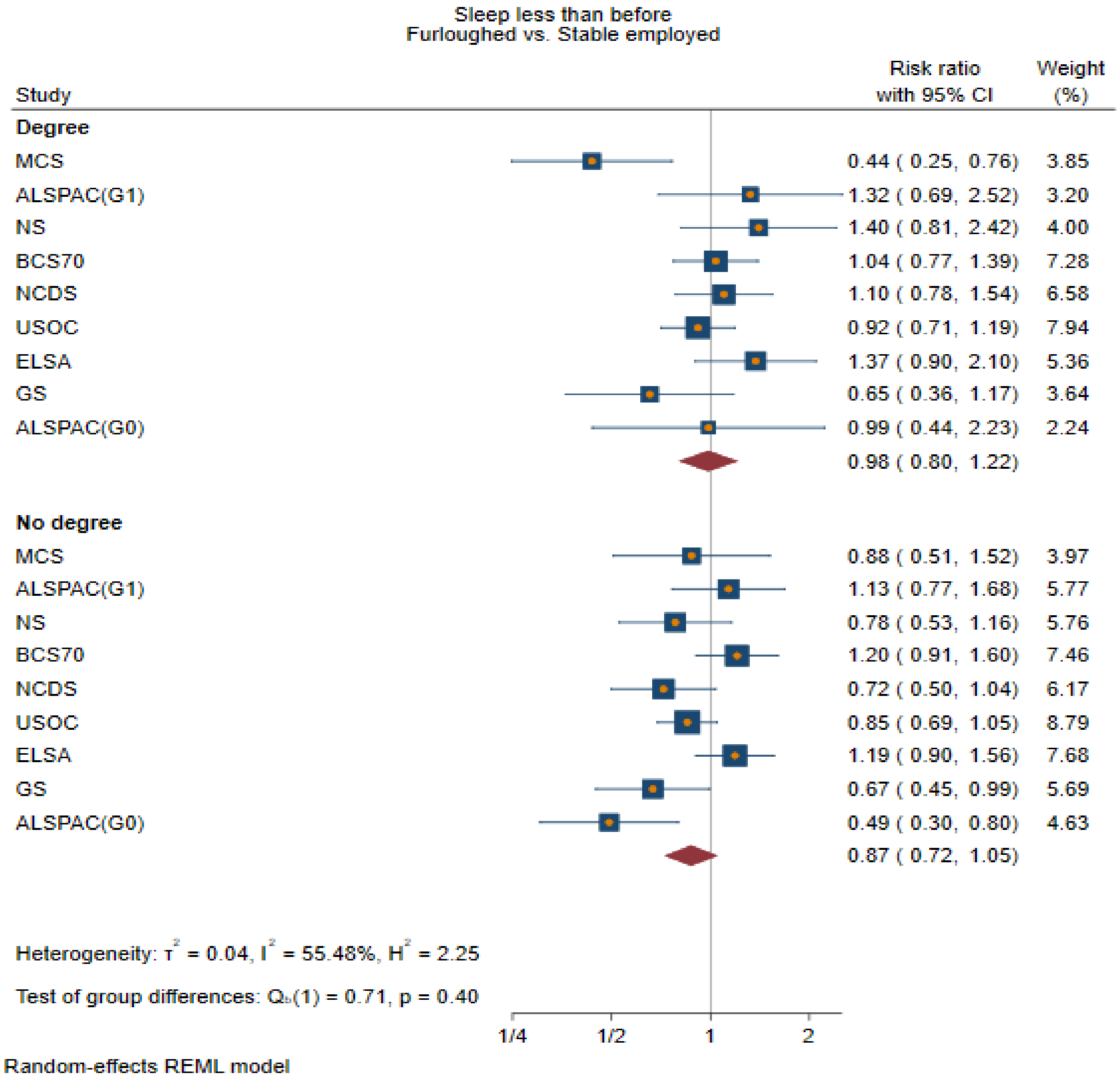

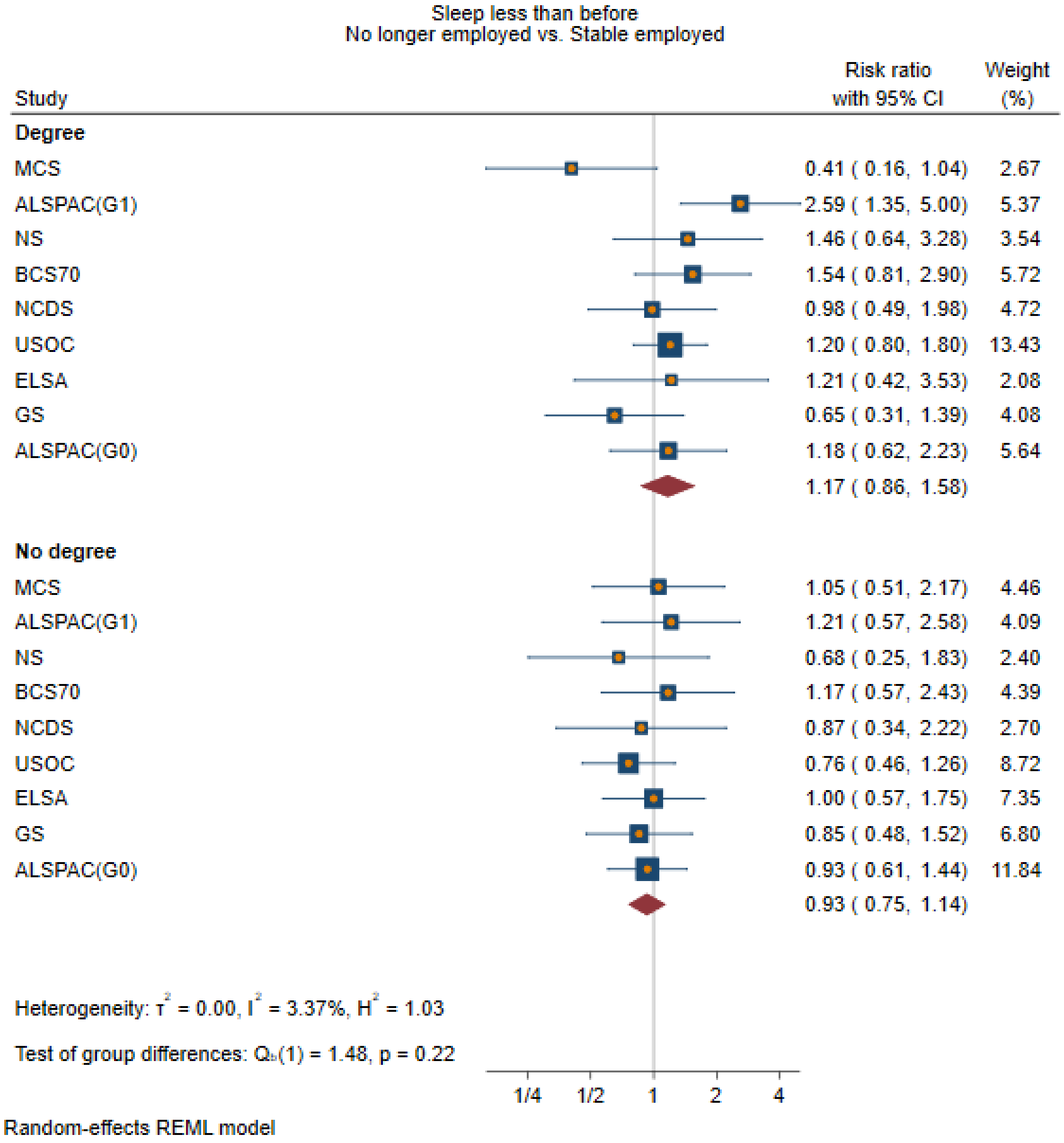

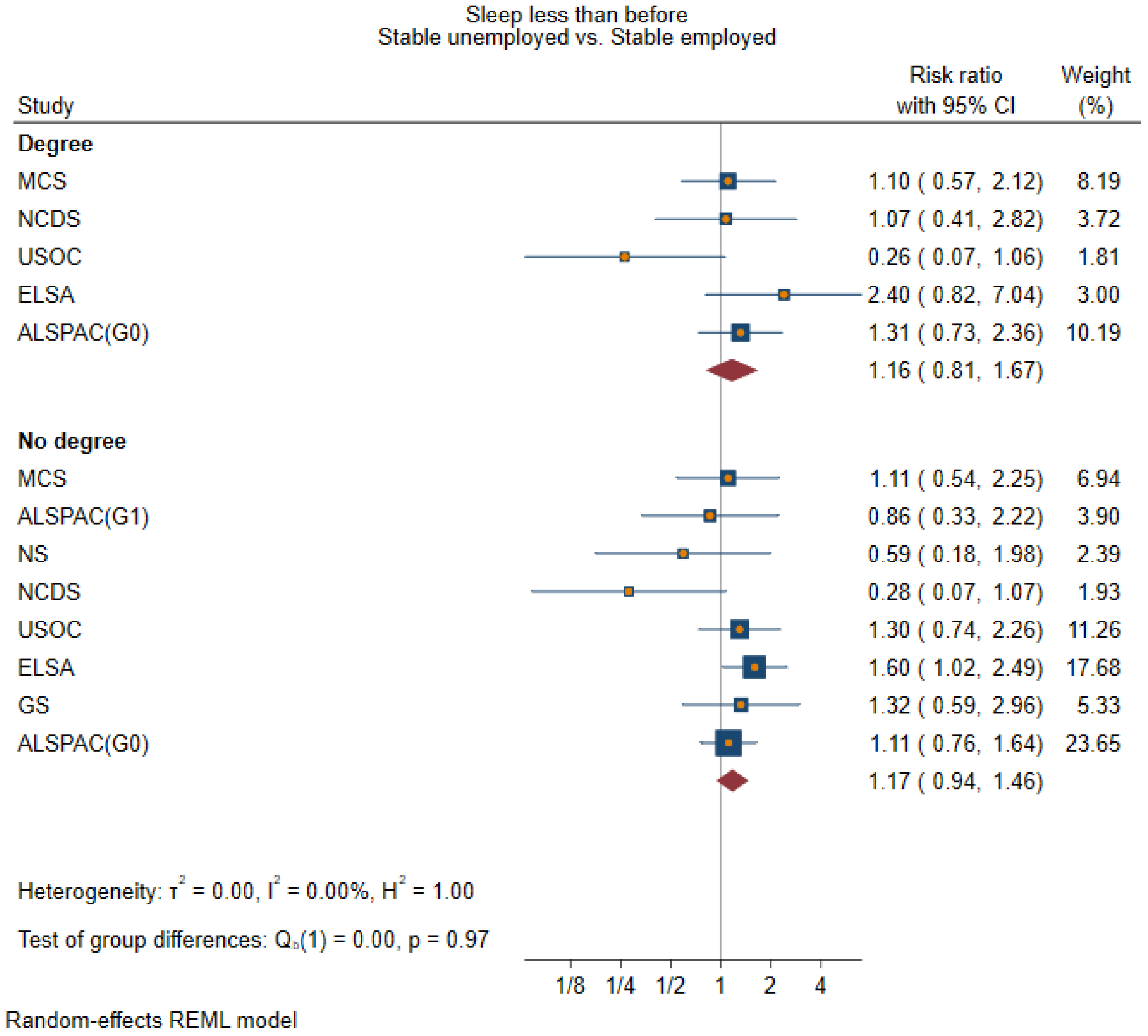
Sleep less than before
Stable unemployed vs. Stable employed
Study
Risk ratio with 95% CI
Weight (%)
Degree
MCS 1.10 (0.57, 2.12) 8.19
NCDS 1.07 (0.41, 2.82) 3.72
USOC 0.26 (0.07, 1.06) 1.81
ELSA 2.40 (0.82, 7.04) 3.00
ALSPAC(G0) 1.31 (0.73, 2.36) 10.19
1.16 (0.81, 1.67)
No degree
MCS 1.11 (0.54, 2.25) 6.94
ALSPAC(G1) 0.86 (0.33, 2.22) 3.90
NS 0.59 (0.18, 1.98) 2.39
NCDS 0.28 (0.07, 1.07) 1.93
USOC 1.30 (0.74, 2.26) 11.26
ELSA 1.60 (1.02, 2.49) 17.68
GS 1.32 (0.59, 2.96) 5.33
ALSPAC(G0) 1.11 (0.76, 1.64) 23.65
1.17 (0.94, 1.46)
Heterogeneity: τ^2 = 0.00, I^2 = 0.00%, H^2 = 1.00
Test of group differences: $Q_b(1)$ = 0.00, p = 0.97
1/8 1/4 1/2 1 2 4
Random-effects REML model

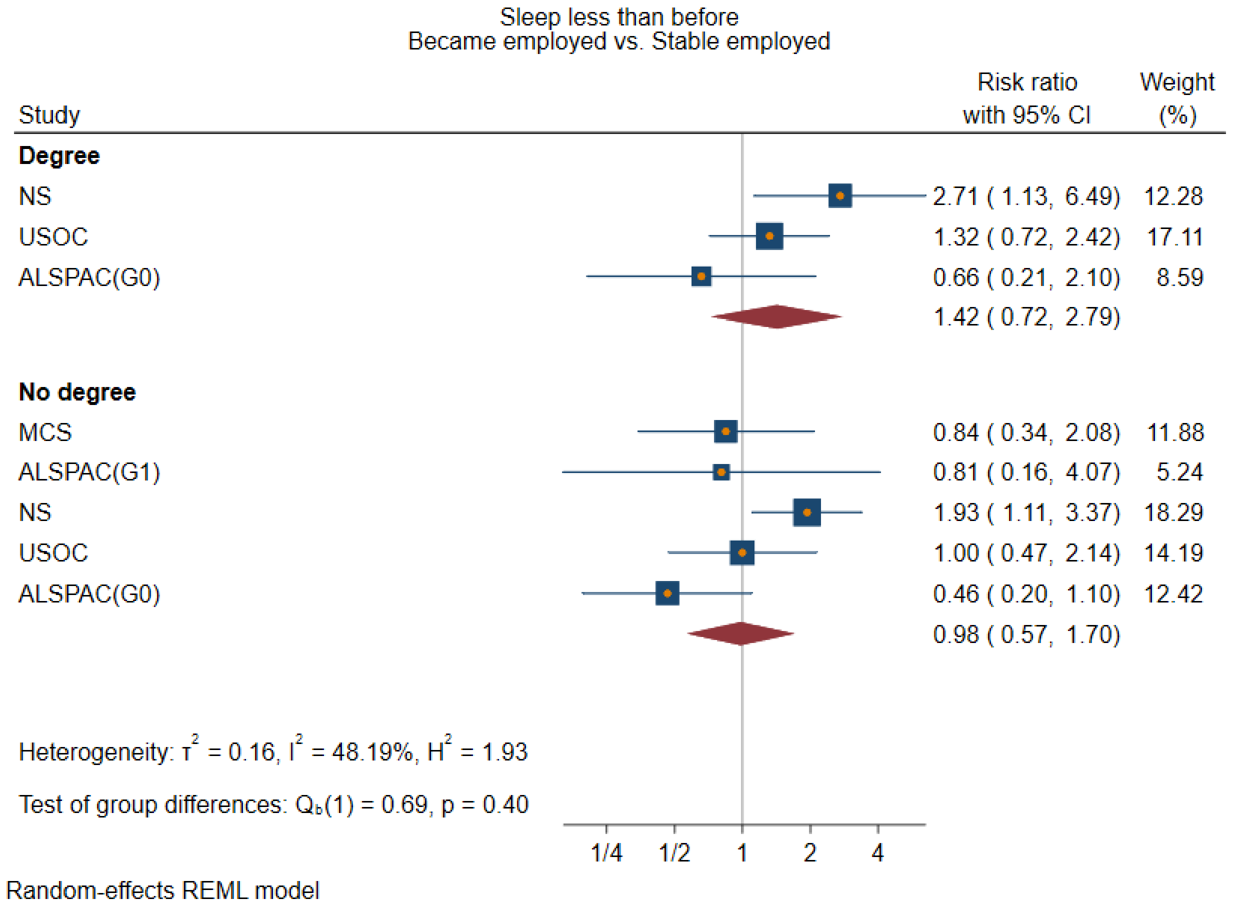
Sleep less than before
Became employed vs. Stable employed
Study
Risk ratio with 95% CI
Weight (%)
Degree
NS 2.71 (1.13, 6.49) 12.28
USOC 1.32 (0.72, 2.42) 17.11
ALSPAC(G0) 0.66 (0.21, 2.10) 8.59
1.42 (0.72, 2.79)
No degree
MCS 0.84 (0.34, 2.08) 11.88
ALSPAC(G1) 0.81 (0.16, 4.07) 5.24
NS 1.93 (1.11, 3.37) 18.29
USOC 1.00 (0.47, 2.14) 14.19
ALSPAC(G0) 0.46 (0.20, 1.10) 12.42
0.98 (0.57, 1.70)
Heterogeneity: τ^2 = 0.16, I^2 = 48.19%, H^2 = 1.93
Test of group differences: $Q_b(1)$ = 0.69, p = 0.40
1/4 1/2 1 2 4
Random-effects REML model

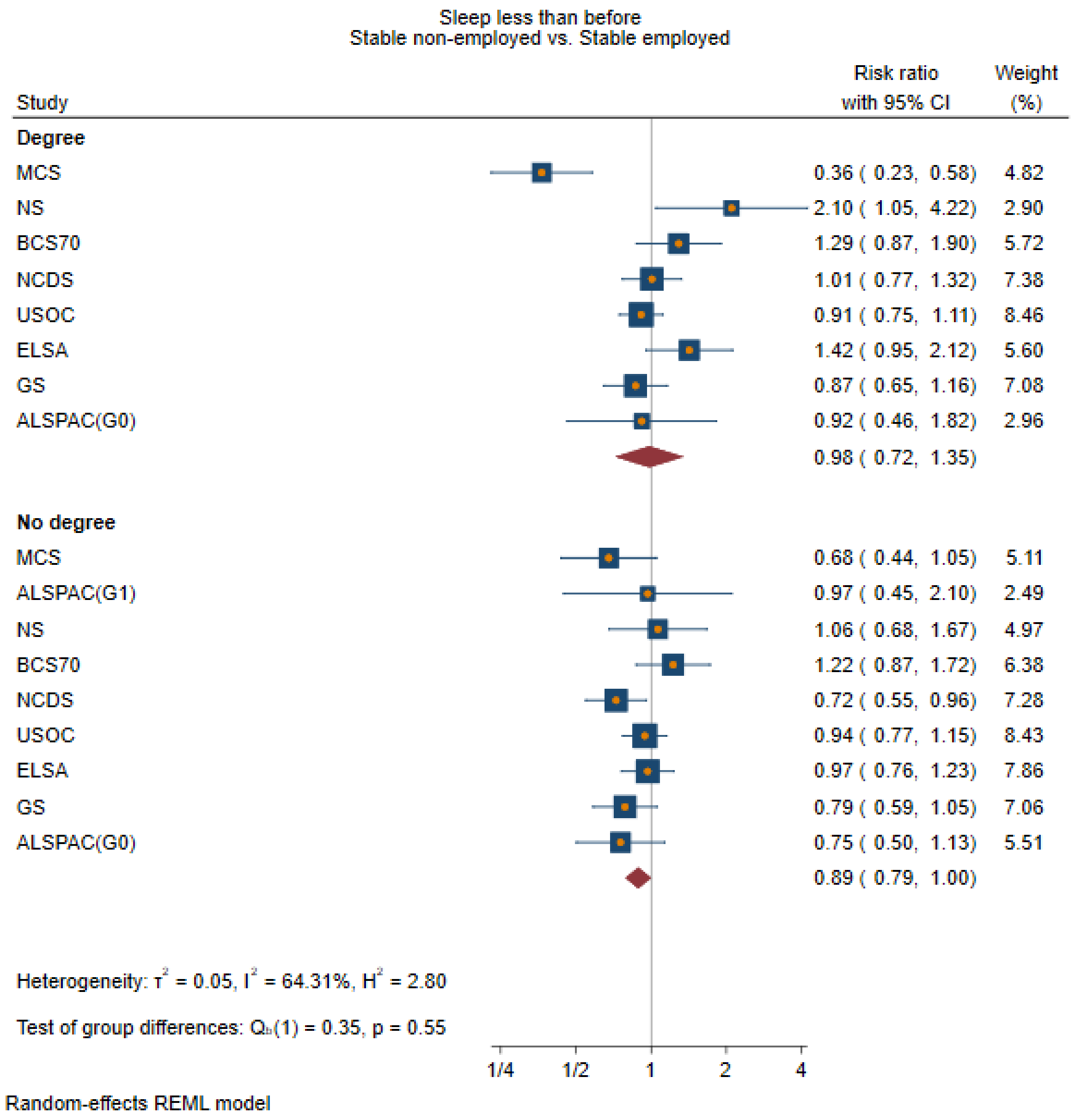
Sleep less than before
Stable non-employed vs. Stable employed
Study
Risk ratio with 95% CI
Weight (%)
Degree
MCS 0.36 (0.23, 0.58) 4.82
NS 2.10 (1.05, 4.22) 2.90
BCS70 1.29 (0.87, 1.90) 5.72
NCDS 1.01 (0.77, 1.32) 7.38
USOC 0.91 (0.75, 1.11) 8.46
ELSA 1.42 (0.95, 2.12) 5.60
GS 0.87 (0.65, 1.16) 7.08
ALSPAC(G0) 0.92 (0.46, 1.82) 2.96
0.98 (0.72, 1.35)
No degree
MCS 0.68 (0.44, 1.05) 5.11
ALSPAC(G1) 0.97 (0.45, 2.10) 2.49
NS 1.06 (0.68, 1.67) 4.97
BCS70 1.22 (0.87, 1.72) 6.38
NCDS 0.72 (0.55, 0.96) 7.28
USOC 0.94 (0.77, 1.15) 8.43
ELSA 0.97 (0.76, 1.23) 7.86
GS 0.79 (0.59, 1.05) 7.06
ALSPAC(G0) 0.75 (0.50, 1.13) 5.51
0.89 (0.79, 1.00)
Heterogeneity: τ^2 = 0.05, I^2 = 64.31%, H^2 = 2.80
Test of group differences: $Q_b(1)$ = 0.35, p = 0.55
1/4
1/2
1
2
4
Random-effects REML model

Figure set 20: Sleeps more than before

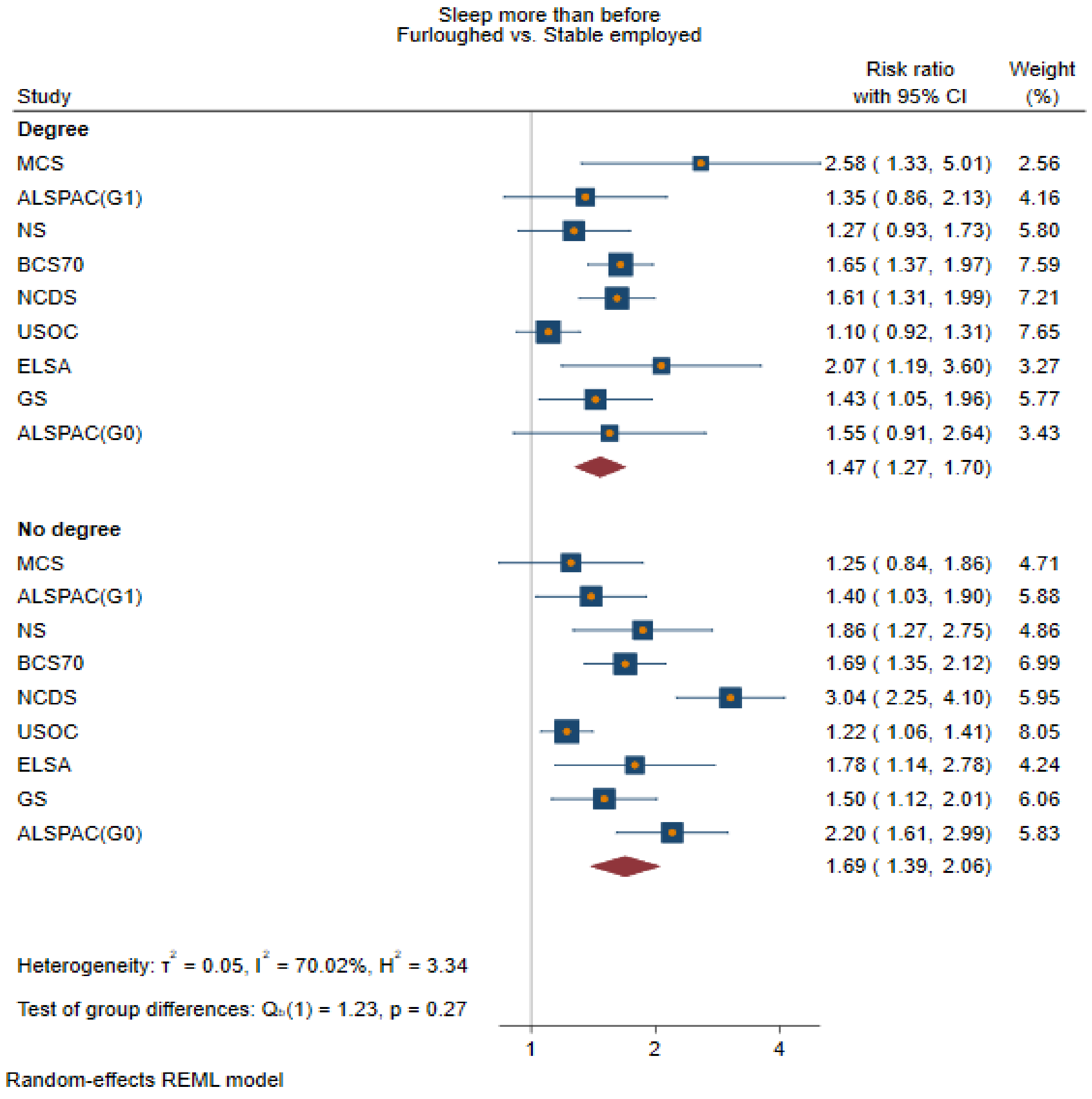

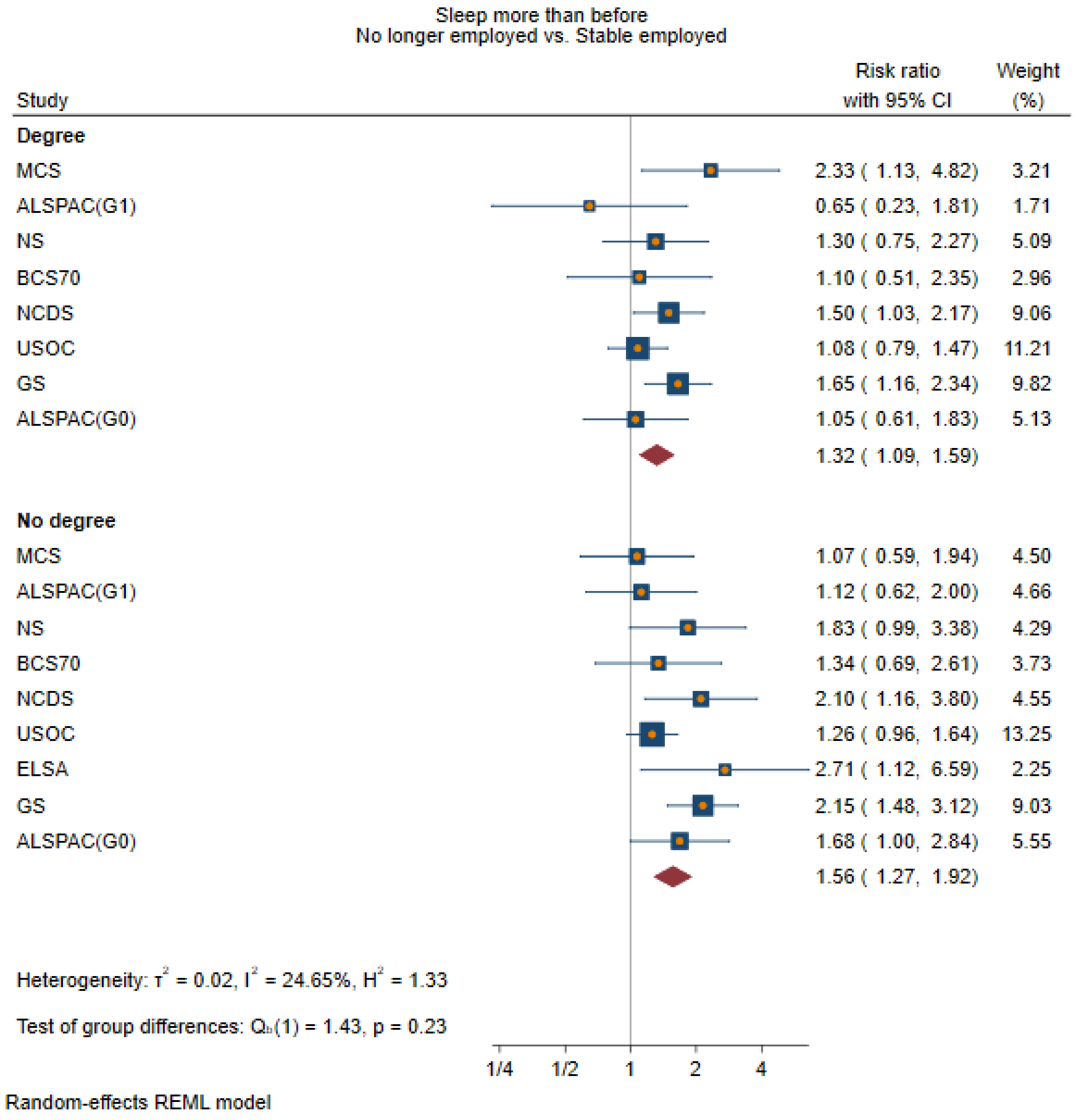

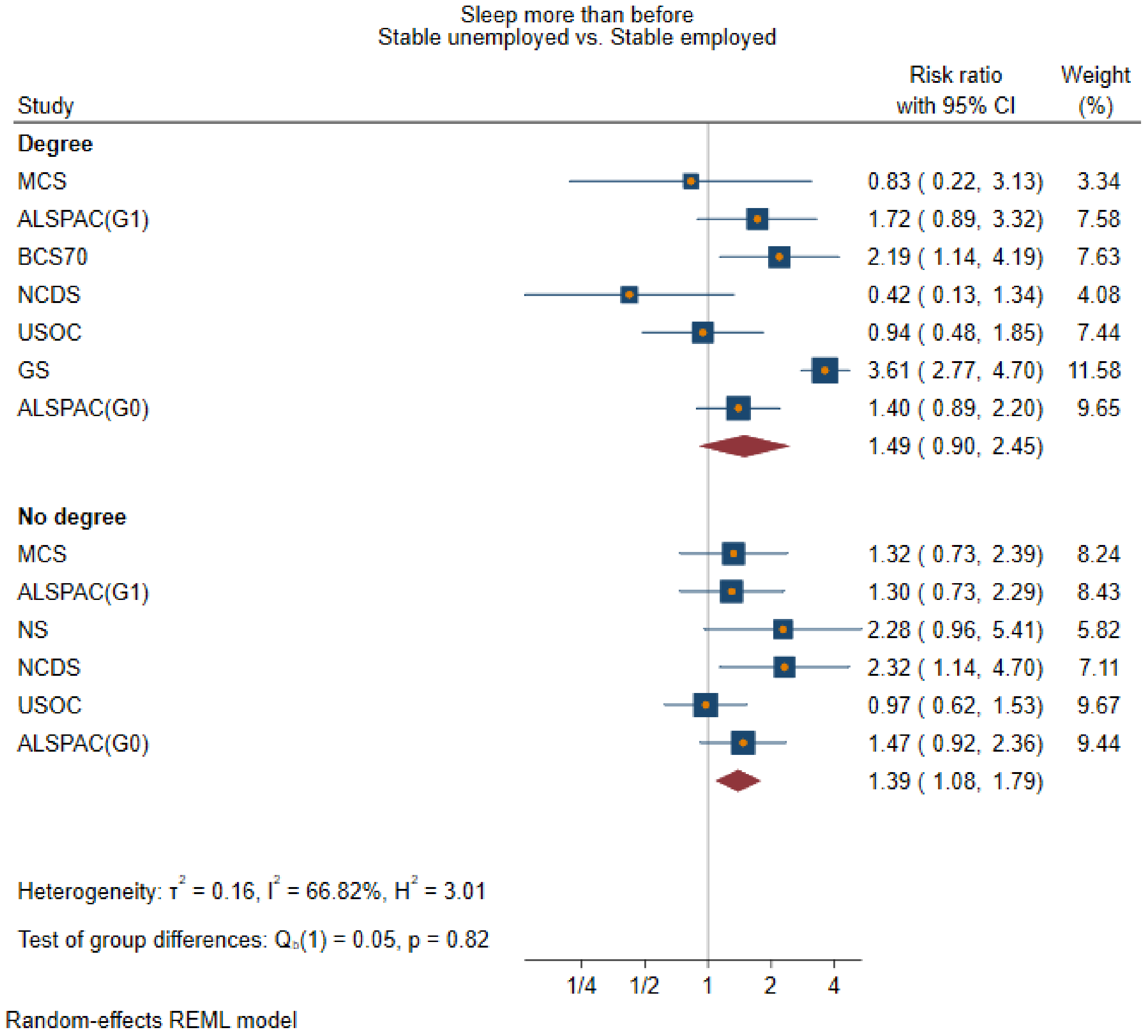
Sleep more than before
Stable unemployed vs. Stable employed
Study
Risk ratio with 95% CI
Weight (%)
Degree
MCS 0.83 (0.22, 3.13) 3.34
ALSPAC(G1) 1.72 (0.89, 3.32) 7.58
BCS70 2.19 (1.14, 4.19) 7.63
NCDS 0.42 (0.13, 1.34) 4.08
USOC 0.94 (0.48, 1.85) 7.44
GS 3.61 (2.77, 4.70) 11.58
ALSPAC(G0) 1.40 (0.89, 2.20) 9.65
1.49 (0.90, 2.45)
No degree
MCS 1.32 (0.73, 2.39) 8.24
ALSPAC(G1) 1.30 (0.73, 2.29) 8.43
NS 2.28 (0.96, 5.41) 5.82
NCDS 2.32 (1.14, 4.70) 7.11
USOC 0.97 (0.62, 1.53) 9.67
ALSPAC(G0) 1.47 (0.92, 2.36) 9.44
1.39 (1.08, 1.79)
Heterogeneity: τ^2 = 0.16, I^2 = 66.82%, H^2 = 3.01
Test of group differences: $Q_b(1)$ = 0.05, p = 0.82
1/4 1/2 1 2 4
Random-effects REML model

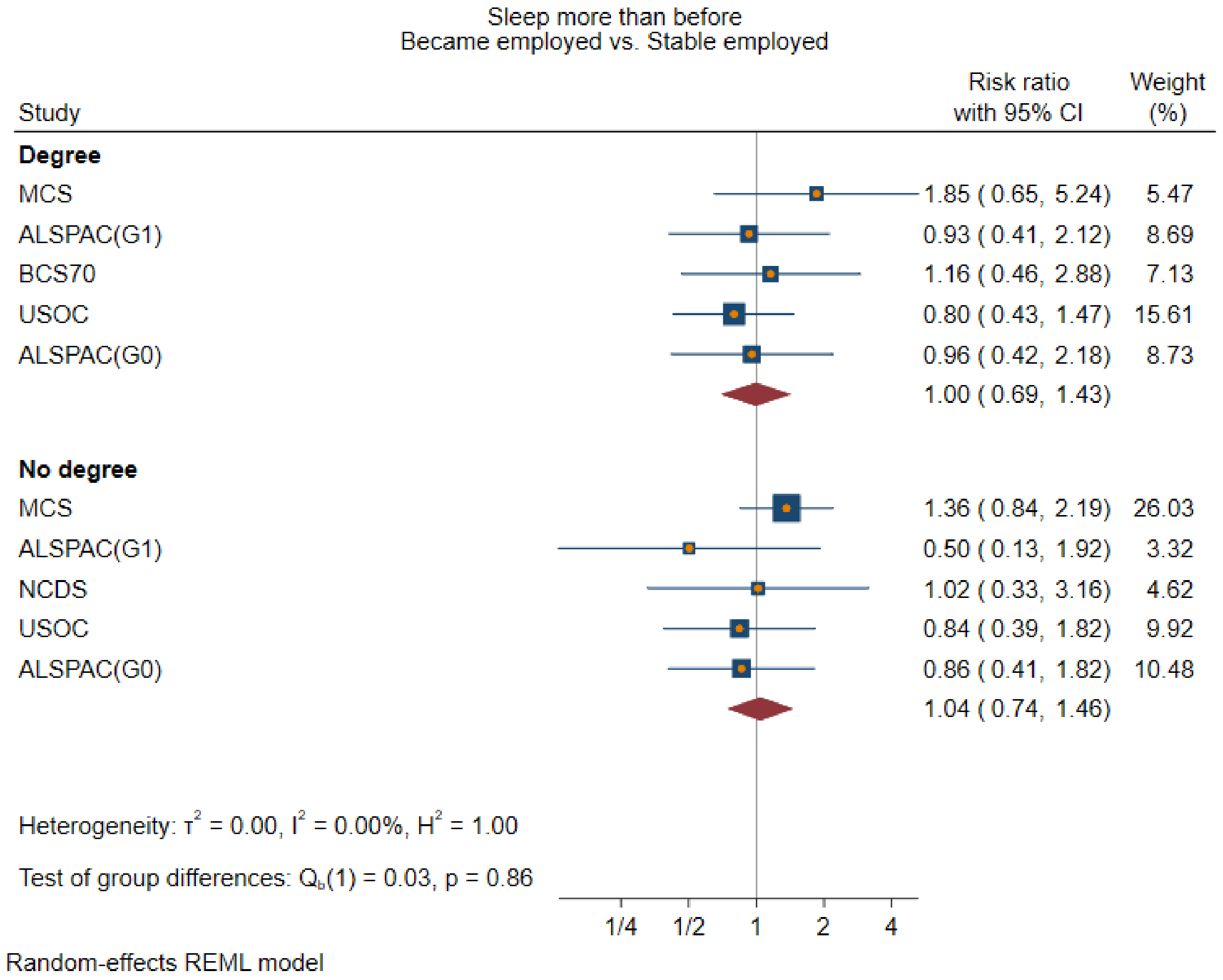
Sleep more than before
Became employed vs. Stable employed
Study
Risk ratio with 95% CI
Weight (%)
Degree
MCS 1.85 (0.65, 5.24) 5.47
ALSPAC(G1) 0.93 (0.41, 2.12) 8.69
BCS70 1.16 (0.46, 2.88) 7.13
USOC 0.80 (0.43, 1.47) 15.61
ALSPAC(G0) 0.96 (0.42, 2.18) 8.73
1.00 (0.69, 1.43)
No degree
MCS 1.36 (0.84, 2.19) 26.03
ALSPAC(G1) 0.50 (0.13, 1.92) 3.32
NCDS 1.02 (0.33, 3.16) 4.62
USOC 0.84 (0.39, 1.82) 9.92
ALSPAC(G0) 0.86 (0.41, 1.82) 10.48
1.04 (0.74, 1.46)
Heterogeneity: τ^2 = 0.00, I^2 = 0.00%, H^2 = 1.00
Test of group differences: $Q_b(1)$ = 0.03, p = 0.86
1/4 1/2 1 2 4
Random-effects REML model

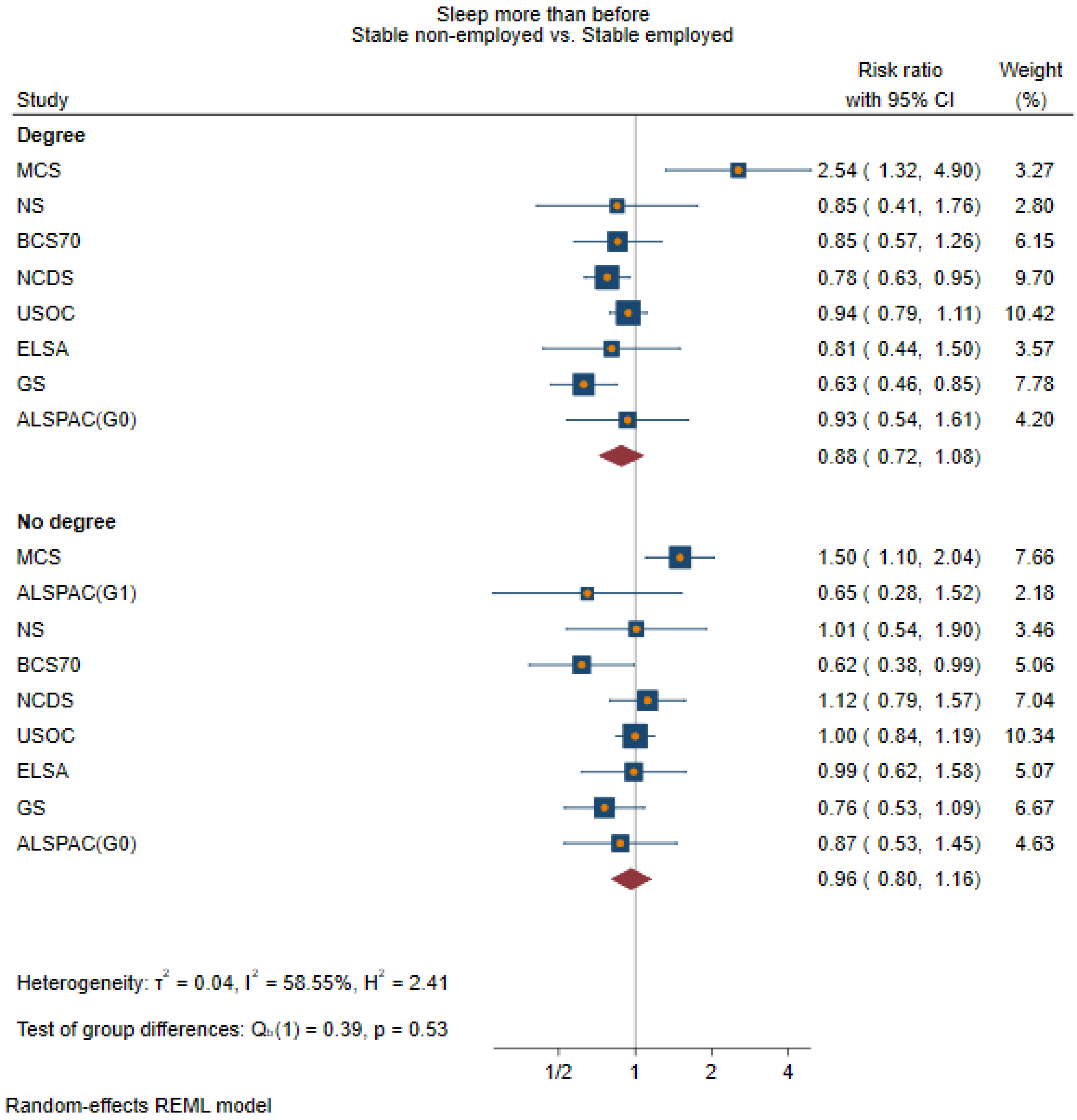
Sleep more than before
Stable non-employed vs. Stable employed
Study
Risk ratio with 95% CI
Weight (%)
Degree
MCS 2.54 (1.32, 4.90) 3.27
NS 0.85 (0.41, 1.76) 2.80
BCS70 0.85 (0.57, 1.26) 6.15
NCDS 0.78 (0.63, 0.95) 9.70
USOC 0.94 (0.79, 1.11) 10.42
ELSA 0.81 (0.44, 1.50) 3.57
GS 0.63 (0.46, 0.85) 7.78
ALSPAC(G0) 0.93 (0.54, 1.61) 4.20
0.88 (0.72, 1.08)
No degree
MCS 1.50 (1.10, 2.04) 7.66
ALSPAC(G1) 0.65 (0.28, 1.52) 2.18
NS 1.01 (0.54, 1.90) 3.46
BCS70 0.62 (0.38, 0.99) 5.06
NCDS 1.12 (0.79, 1.57) 7.04
USOC 1.00 (0.84, 1.19) 10.34
ELSA 0.99 (0.62, 1.58) 5.07
GS 0.76 (0.53, 1.09) 6.67
ALSPAC(G0) 0.87 (0.53, 1.45) 4.63
0.96 (0.80, 1.16)
Heterogeneity: τ² = 0.04, I² = 58.55%, H² = 2.41
Test of group differences: Qb(1) = 0.39, p = 0.53
1/2
1
2
4
Random-effects REML model

Figure set 21: From 6/9h a night to 'normal range'

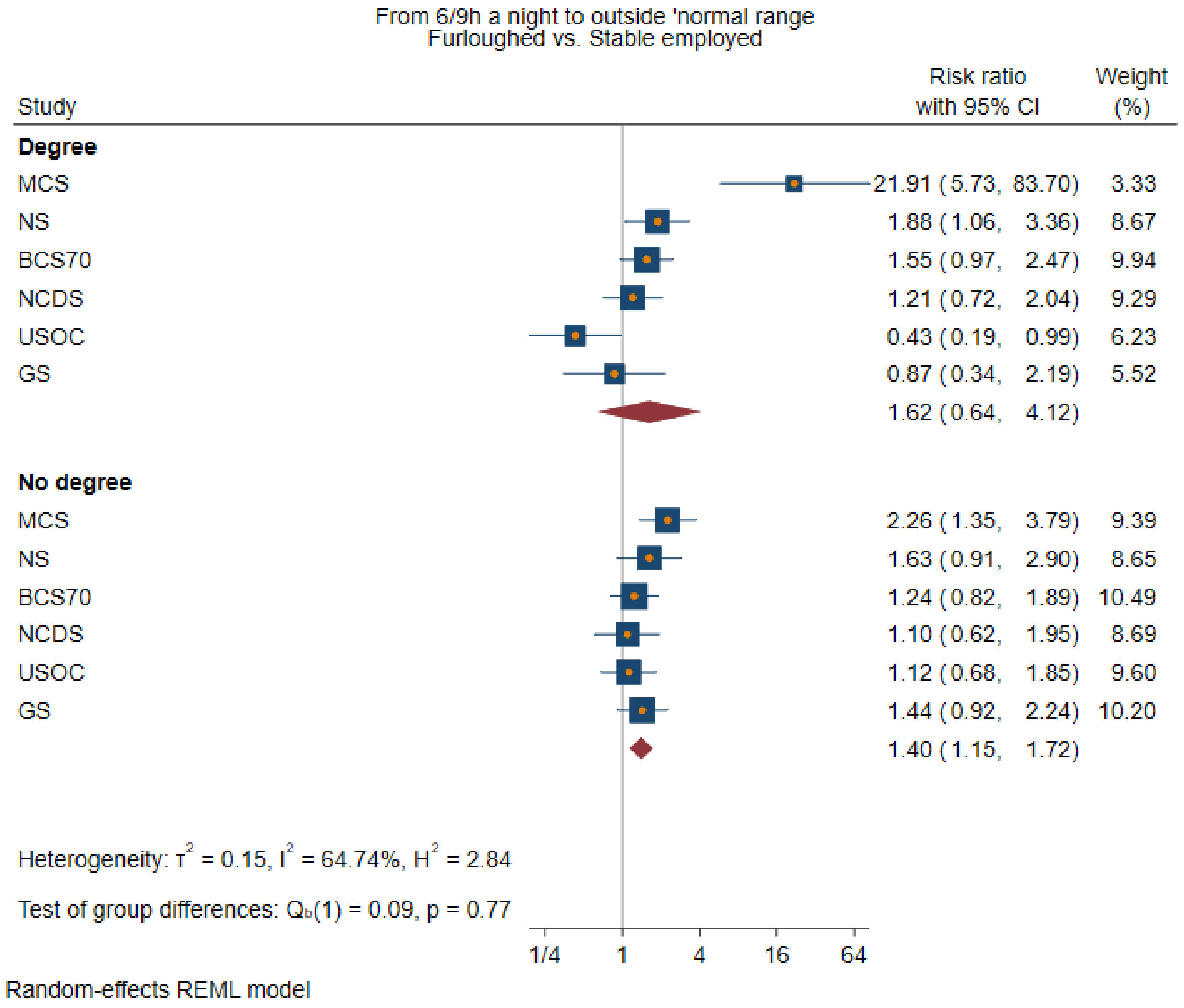

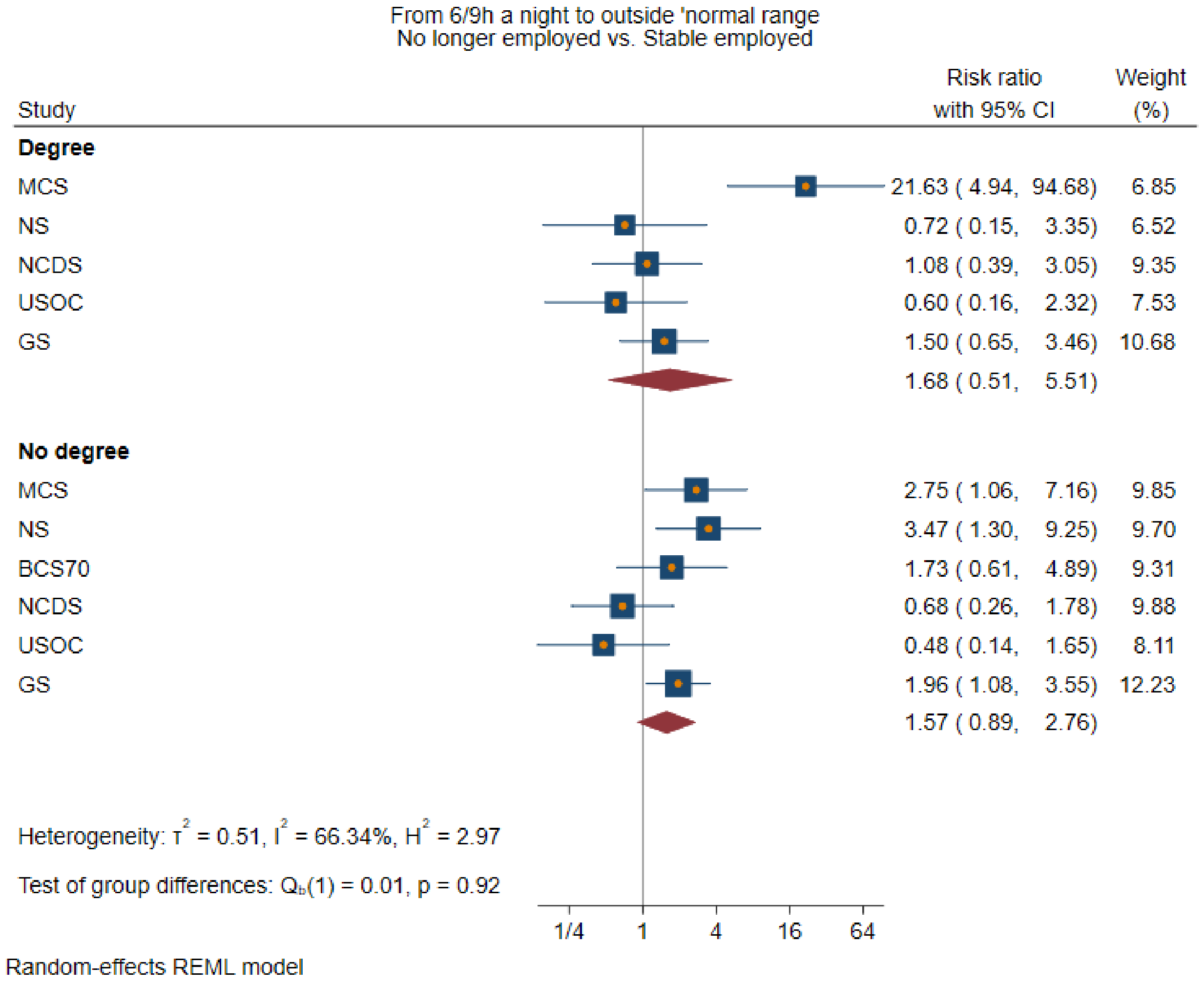

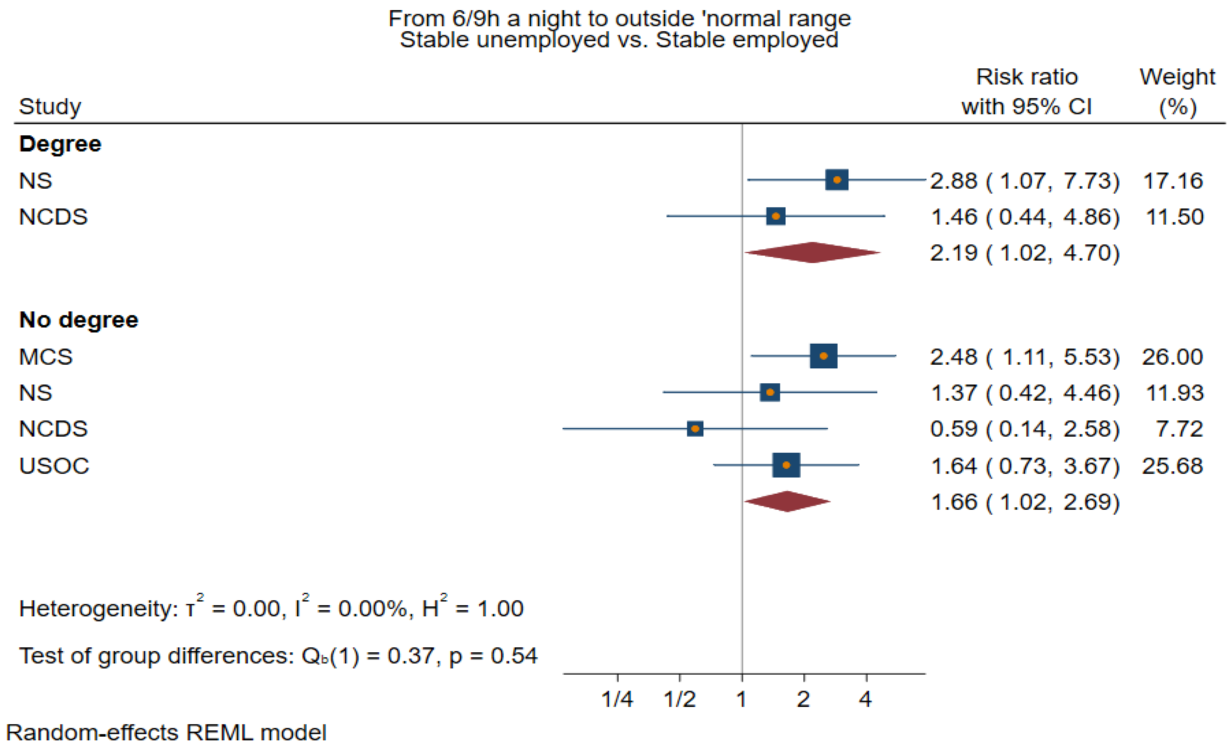
From 6/9h a night to outside 'normal range
Stable unemployed vs. Stable employed
Study
Risk ratio with 95% CI
Weight (%)
Degree
NS 2.88 (1.07, 7.73) 17.16
NCDS 1.46 (0.44, 4.86) 11.50
2.19 (1.02, 4.70)
No degree
MCS 2.48 (1.11, 5.53) 26.00
NS 1.37 (0.42, 4.46) 11.93
NCDS 0.59 (0.14, 2.58) 7.72
USOC 1.64 (0.73, 3.67) 25.68
1.66 (1.02, 2.69)
Heterogeneity: τ² = 0.00, I² = 0.00%, H² = 1.00
Test of group differences: Qb(1) = 0.37, p = 0.54
1/4
1/2
1
2
4
Random-effects REML model

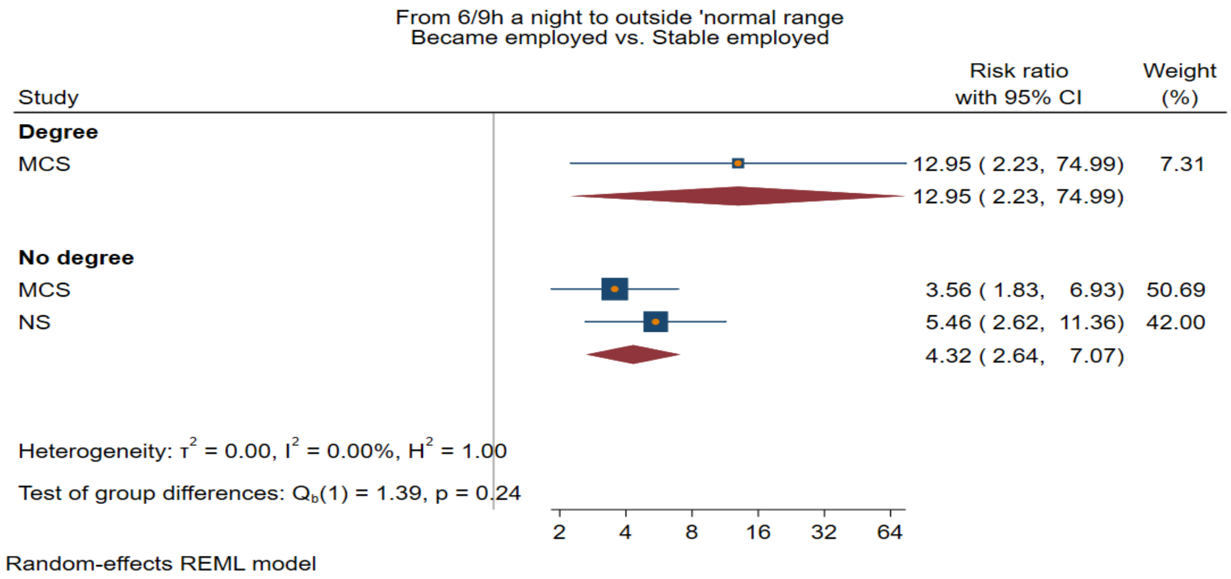
From 6/9h a night to outside 'normal range
Became employed vs. Stable employed
Study
Risk ratio with 95% CI
Weight (%)
Degree
MCS 12.95 (2.23, 74.99) 7.31
12.95 (2.23, 74.99)
No degree
MCS 3.56 (1.83, 6.93) 50.69
NS 5.46 (2.62, 11.36) 42.00
4.32 (2.64, 7.07)
Heterogeneity: τ² = 0.00, I² = 0.00%, H² = 1.00
Test of group differences: Qb(1) = 1.39, p = 0.24
2
4
8
16
32
64
Random-effects REML model

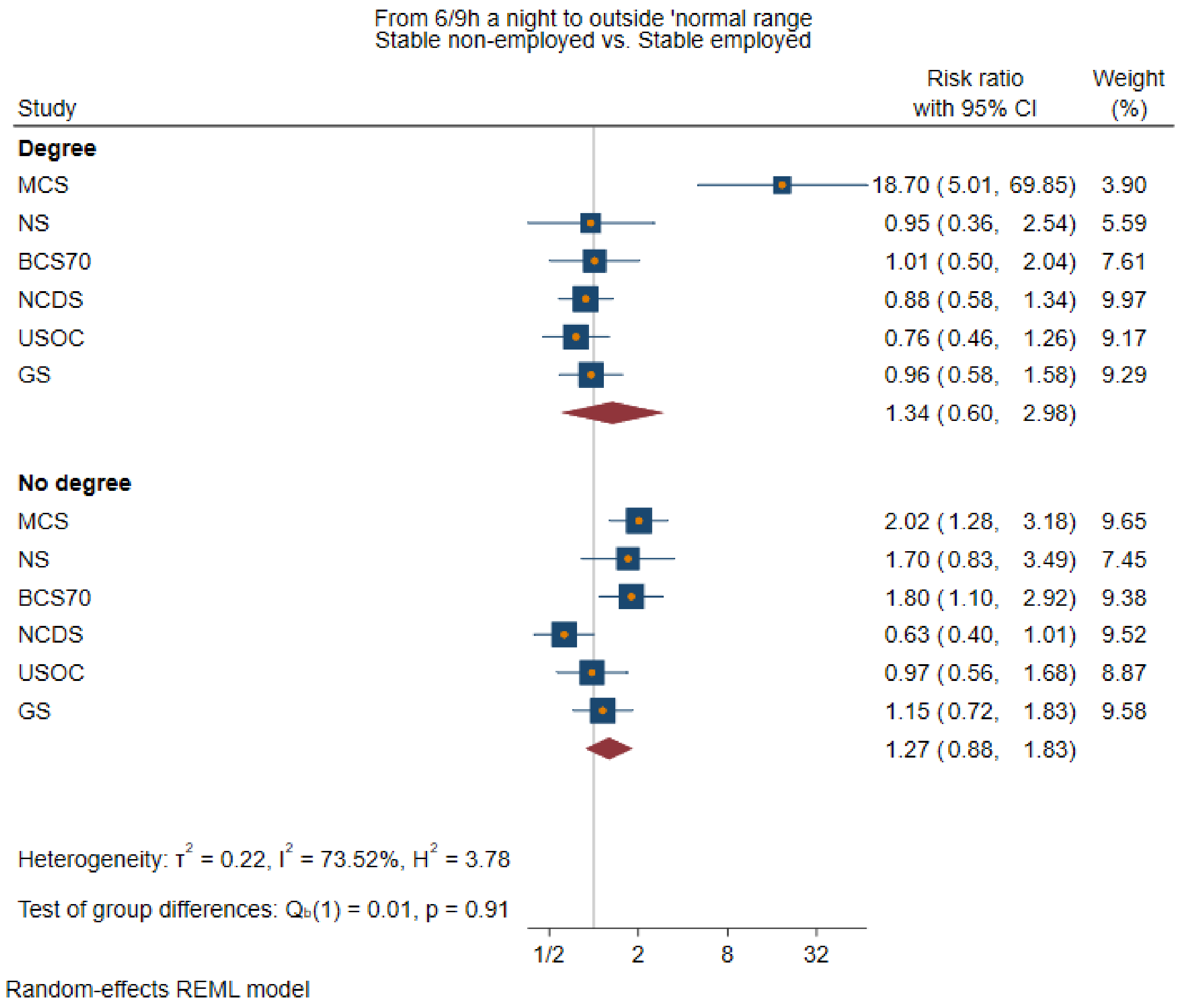
From 6/9h a night to outside 'normal range
Stable non-employed vs. Stable employed
Study
Risk ratio with 95% CI
Weight (%)
Degree
MCS 18.70 (5.01, 69.85) 3.90
NS 0.95 (0.36, 2.54) 5.59
BCS70 1.01 (0.50, 2.04) 7.61
NCDS 0.88 (0.58, 1.34) 9.97
USOC 0.76 (0.46, 1.26) 9.17
GS 0.96 (0.58, 1.58) 9.29
1.34 (0.60, 2.98)
No degree
MCS 2.02 (1.28, 3.18) 9.65
NS 1.70 (0.83, 3.49) 7.45
BCS70 1.80 (1.10, 2.92) 9.38
NCDS 0.63 (0.40, 1.01) 9.52
USOC 0.97 (0.56, 1.68) 8.87
GS 1.15 (0.72, 1.83) 9.58
1.27 (0.88, 1.83)
Heterogeneity: τ² = 0.22, I² = 73.52%, H² = 3.78
Test of group differences: Qb(1) = 0.01, p = 0.91
1/2
2
8
32
Random-effects REML model

Figure set 22: From ‘normal range’ to 6/9h a night

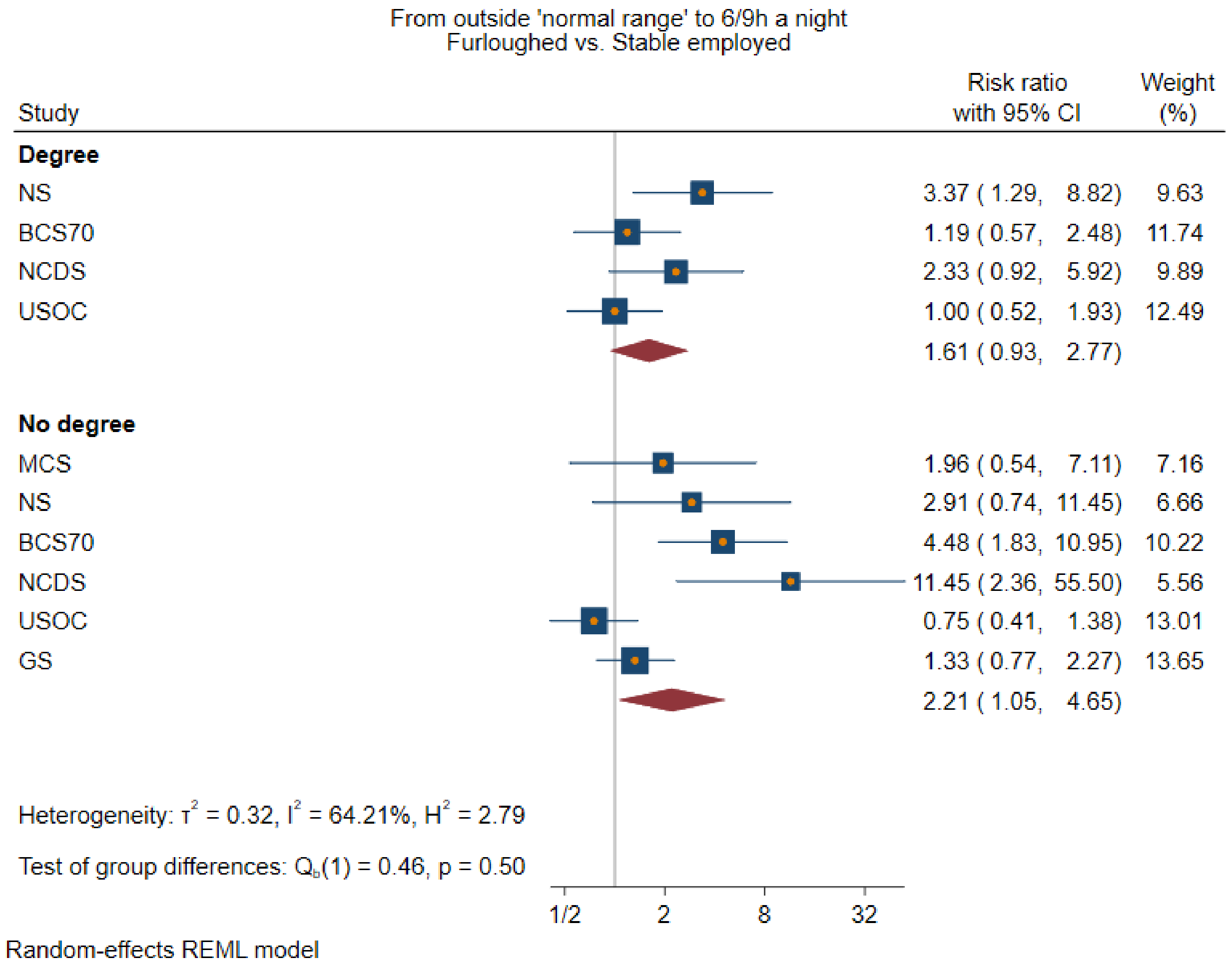

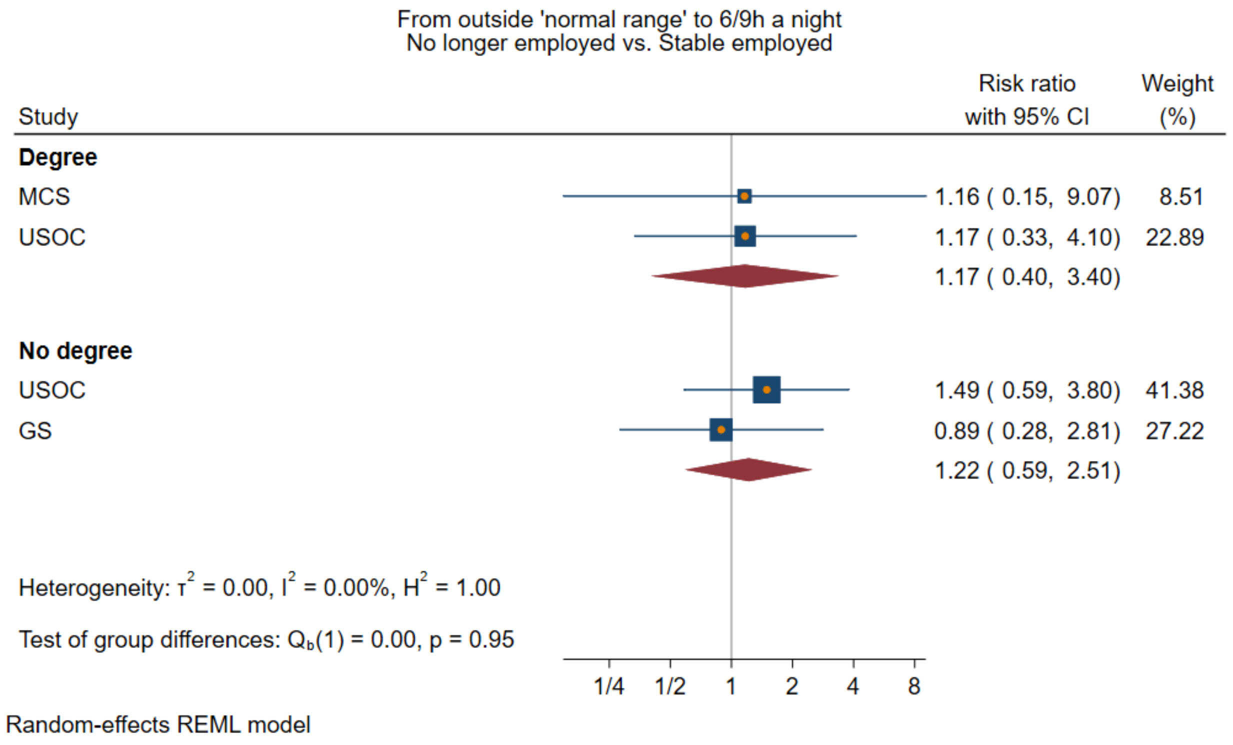

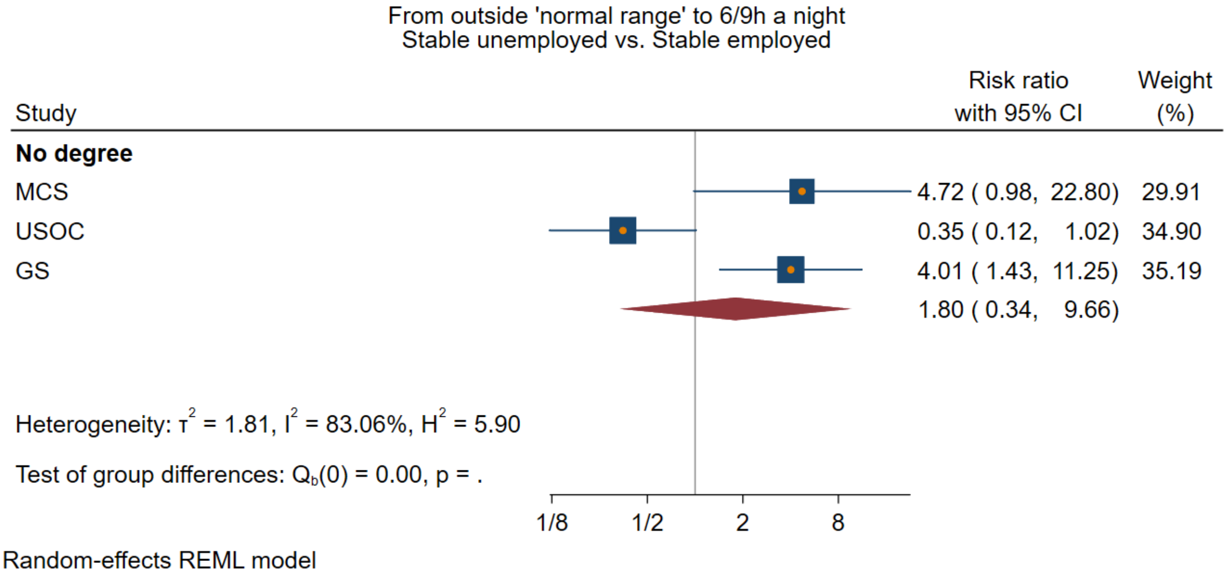
From outside 'normal range' to 6/9h a night
Stable unemployed vs. Stable employed
Study
Risk ratio with 95% CI
Weight (%)
No degree
MCS 4.72 (0.98, 22.80) 29.91
USOC 0.35 (0.12, 1.02) 34.90
GS 4.01 (1.43, 11.25) 35.19
1.80 (0.34, 9.66)
Heterogeneity: τ² = 1.81, I² = 83.06%, H² = 5.90
Test of group differences: Qb(0) = 0.00, p = .
1/8
1/2
2
8
Random-effects REML model

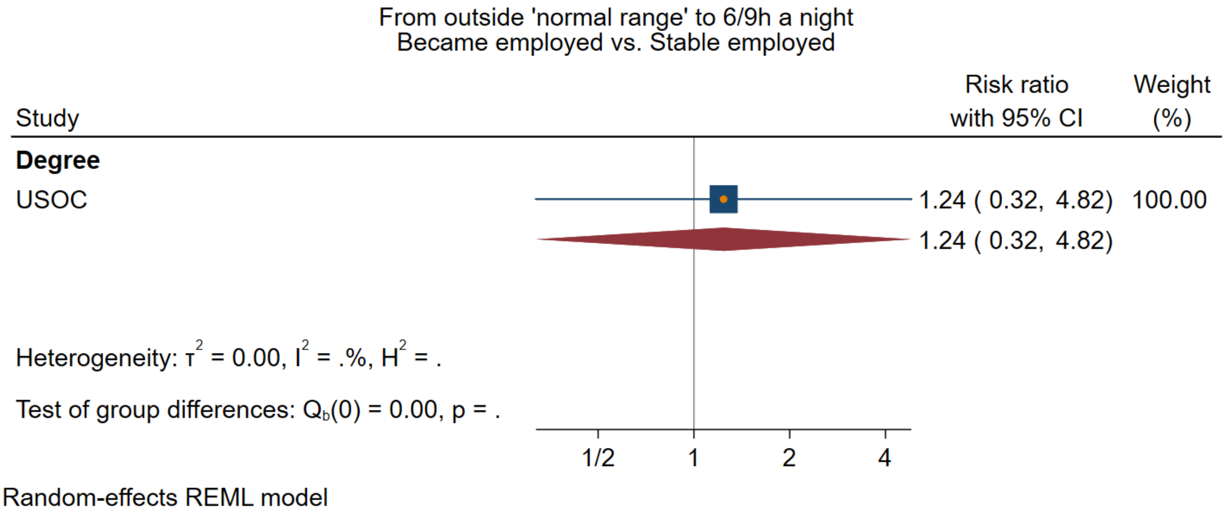
From outside 'normal range' to 6/9h a night
Became employed vs. Stable employed
Study
Risk ratio with 95% CI
Weight (%)
Degree
USOC 1.24 (0.32, 4.82) 100.00
1.24 (0.32, 4.82)
Heterogeneity: τ² = 0.00, I² = .%, H² = .
Test of group differences: Qb(0) = 0.00, p = .
1/2
1
2
4
Random-effects REML model

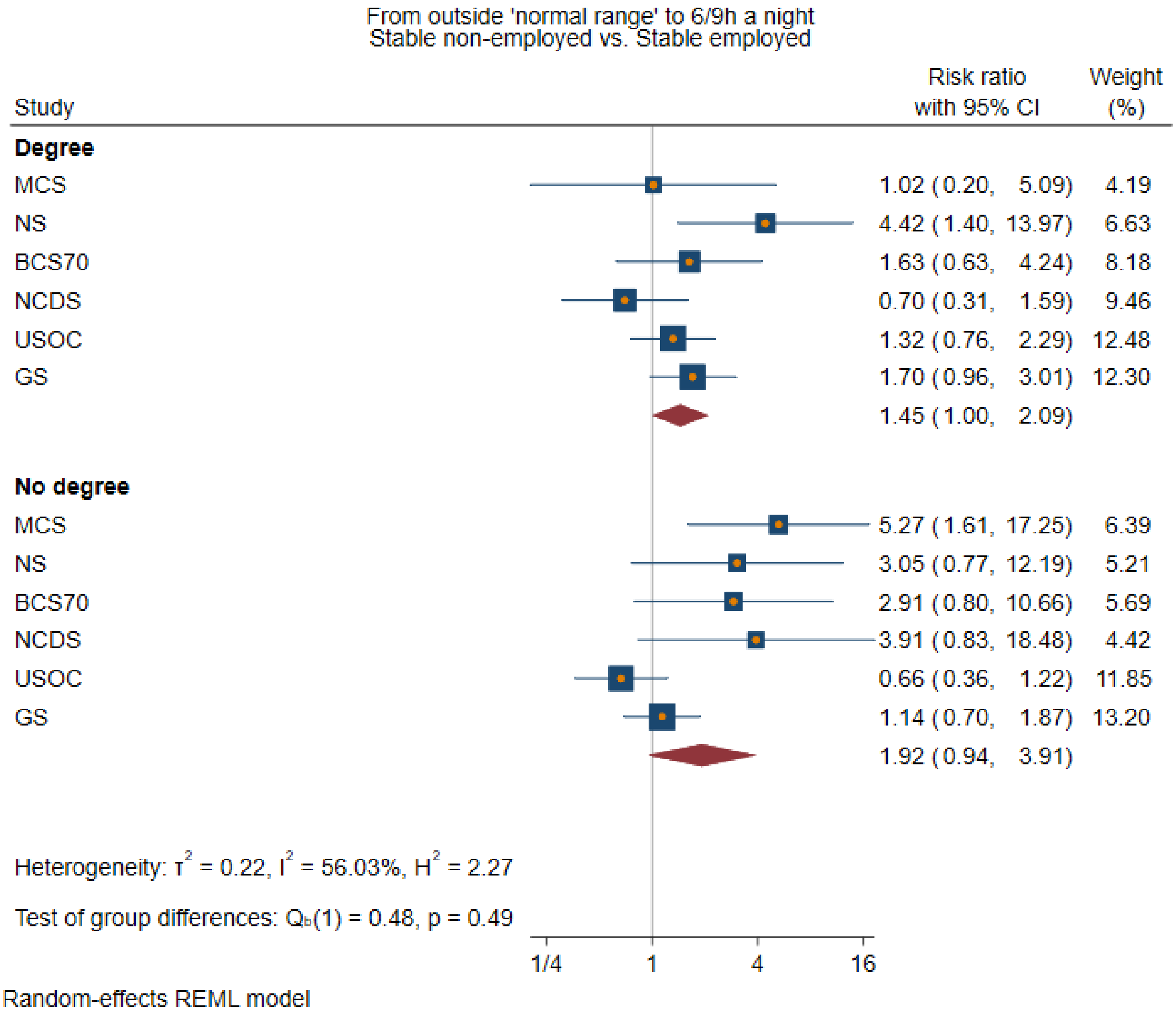
From outside 'normal range' to 6/9h a night
Stable non-employed vs. Stable employed
Study
Risk ratio with 95% CI
Weight (%)
Degree
MCS 1.02 (0.20, 5.09) 4.19
NS 4.42 (1.40, 13.97) 6.63
BCS70 1.63 (0.63, 4.24) 8.18
NCDS 0.70 (0.31, 1.59) 9.46
USOC 1.32 (0.76, 2.29) 12.48
GS 1.70 (0.96, 3.01) 12.30
1.45 (1.00, 2.09)
No degree
MCS 5.27 (1.61, 17.25) 6.39
NS 3.05 (0.77, 12.19) 5.21
BCS70 2.91 (0.80, 10.66) 5.69
NCDS 3.91 (0.83, 18.48) 4.42
USOC 0.66 (0.36, 1.22) 11.85
GS 1.14 (0.70, 1.87) 13.20
1.92 (0.94, 3.91)
Heterogeneity: τ² = 0.22, I² = 56.03%, H² = 2.27
Test of group differences: Qb(1) = 0.48, p = 0.49
1/4
1
4
16
Random-effects REML model

Figure set 23: Currently eats 2 or fewer fruit and veg

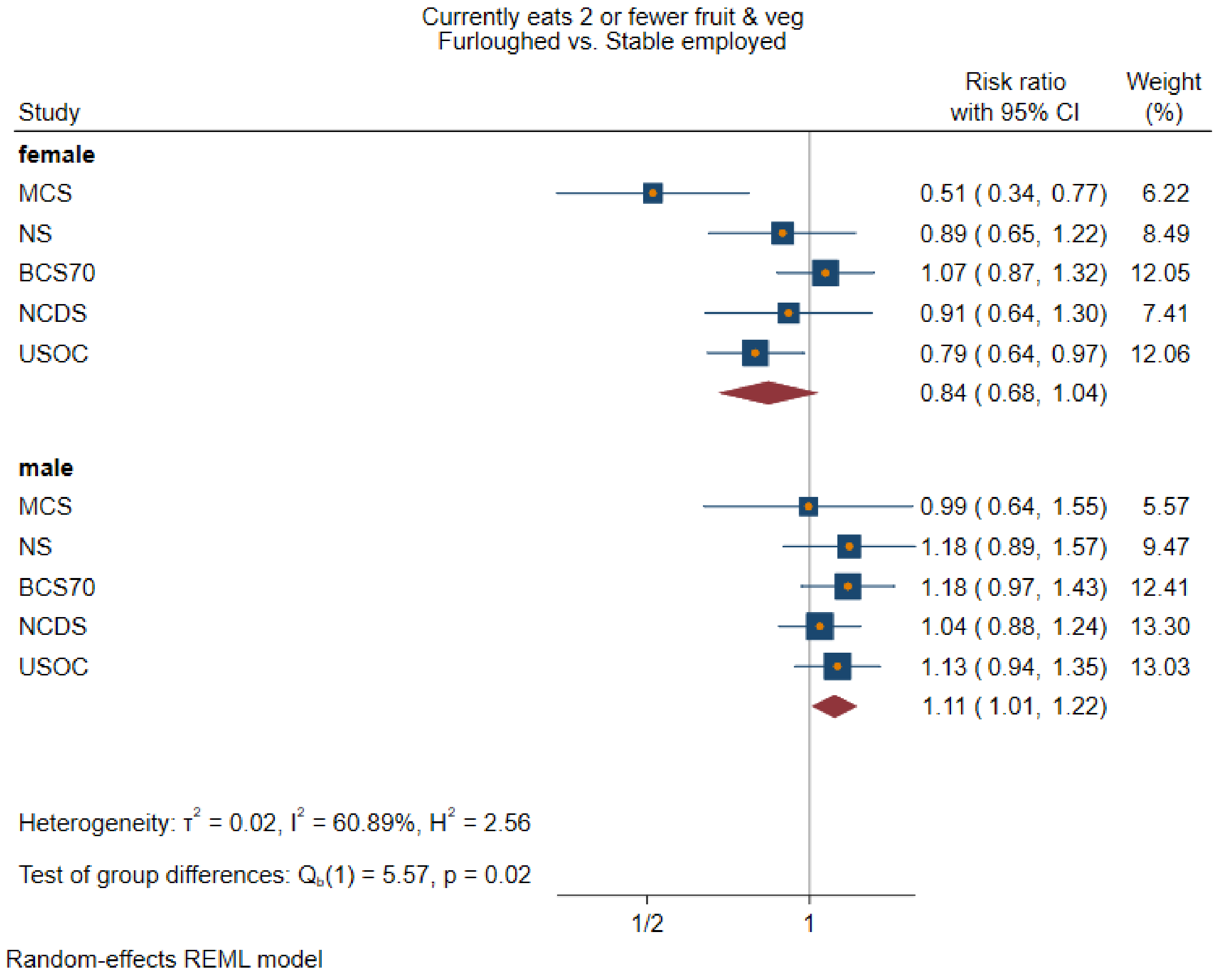

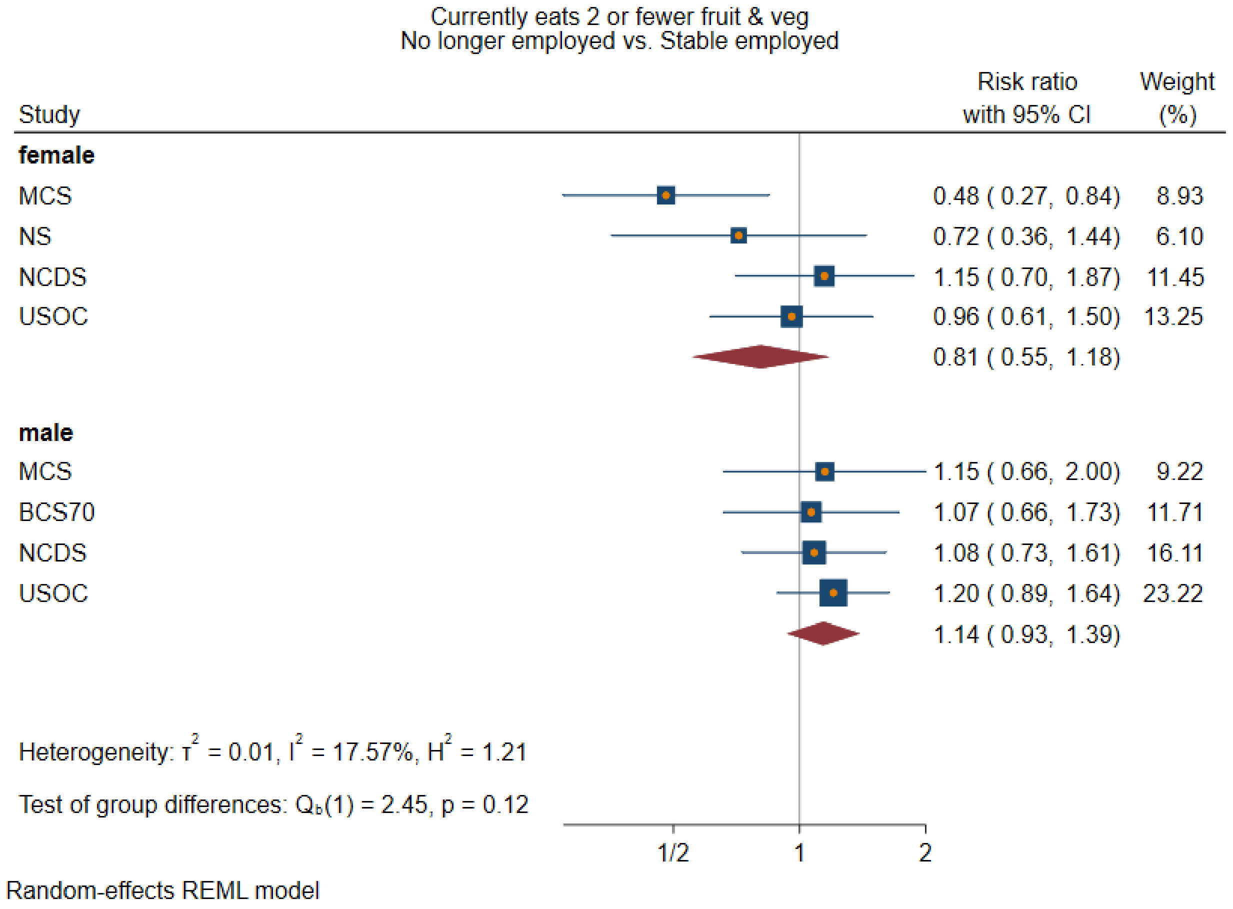

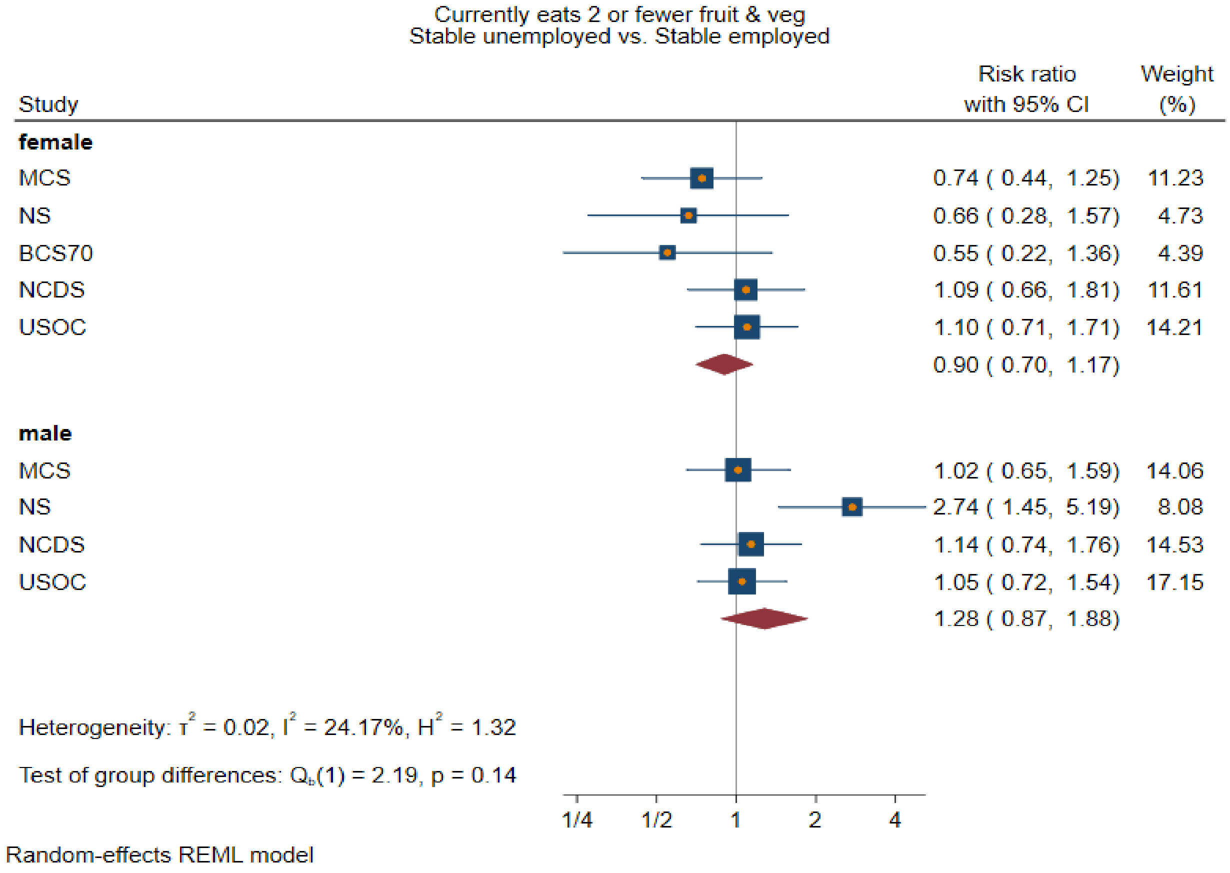
Currently eats 2 or fewer fruit & veg
Stable unemployed vs. Stable employed
Study
Risk ratio with 95% CI
Weight (%)
female
MCS 0.74 (0.44, 1.25) 11.23
NS 0.66 (0.28, 1.57) 4.73
BCS70 0.55 (0.22, 1.36) 4.39
NCDS 1.09 (0.66, 1.81) 11.61
USOC 1.10 (0.71, 1.71) 14.21
0.90 (0.70, 1.17)
male
MCS 1.02 (0.65, 1.59) 14.06
NS 2.74 (1.45, 5.19) 8.08
NCDS 1.14 (0.74, 1.76) 14.53
USOC 1.05 (0.72, 1.54) 17.15
1.28 (0.87, 1.88)
Heterogeneity: τ^2 = 0.02, I^2 = 24.17%, H^2 = 1.32
Test of group differences: $Q_b(1)$ = 2.19, p = 0.14
1/4 1/2 1 2 4
Random-effects REML model

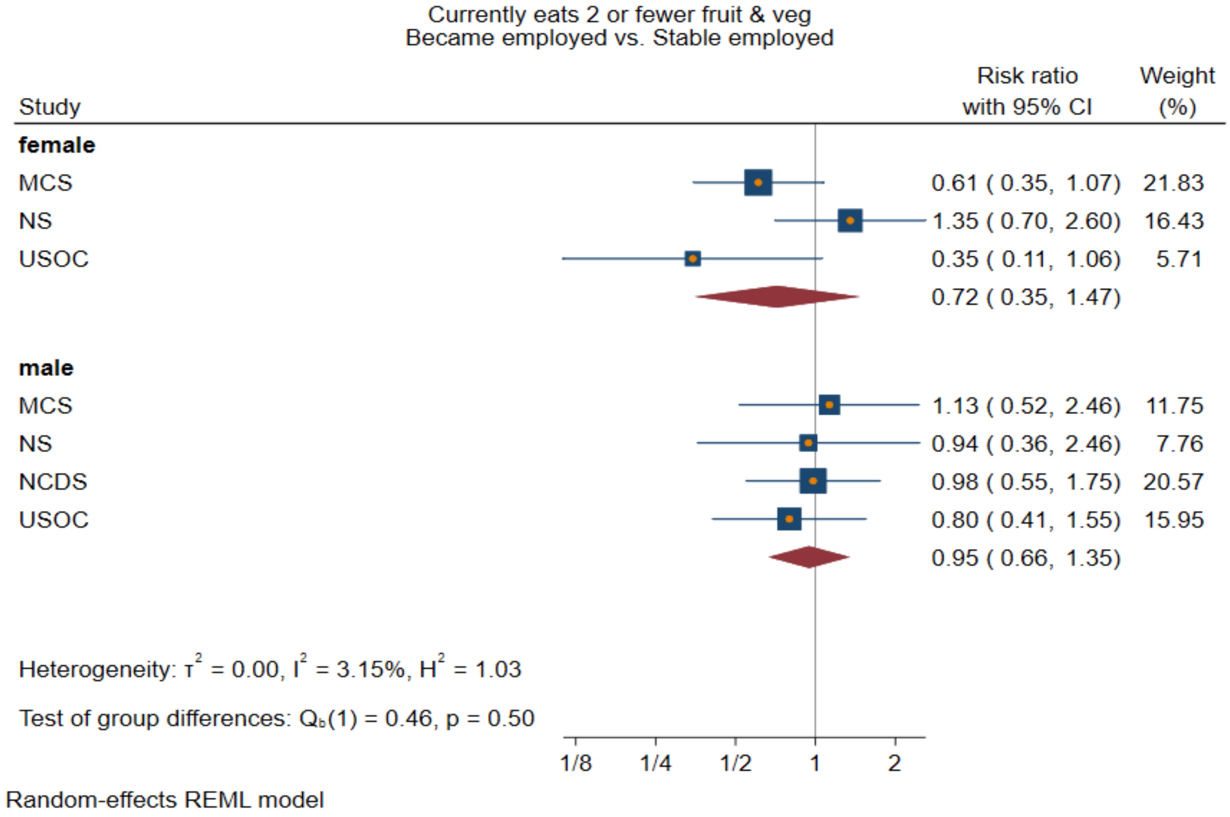
Currently eats 2 or fewer fruit & veg
Became employed vs. Stable employed
Study
Risk ratio with 95% CI
Weight (%)
female
MCS 0.61 (0.35, 1.07) 21.83
NS 1.35 (0.70, 2.60) 16.43
USOC 0.35 (0.11, 1.06) 5.71
0.72 (0.35, 1.47)
male
MCS 1.13 (0.52, 2.46) 11.75
NS 0.94 (0.36, 2.46) 7.76
NCDS 0.98 (0.55, 1.75) 20.57
USOC 0.80 (0.41, 1.55) 15.95
0.95 (0.66, 1.35)
Heterogeneity: τ^2 = 0.00, I^2 = 3.15%, H^2 = 1.03
Test of group differences: $Q_b(1)$ = 0.46, p = 0.50
1/8 1/4 1/2 1 2
Random-effects REML model

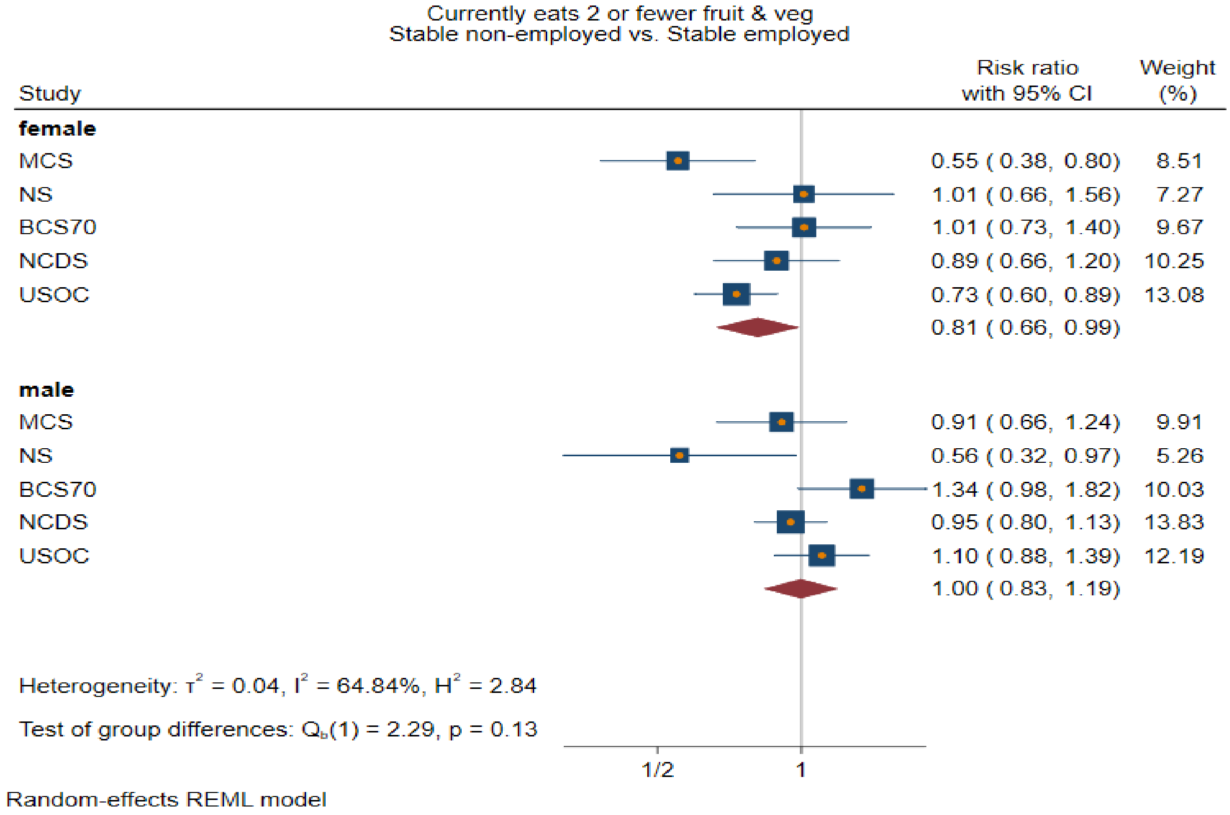
Currently eats 2 or fewer fruit & veg
Stable non-employed vs. Stable employed
Study
Risk ratio with 95% CI
Weight (%)
female
MCS 0.55 (0.38, 0.80) 8.51
NS 1.01 (0.66, 1.56) 7.27
BCS70 1.01 (0.73, 1.40) 9.67
NCDS 0.89 (0.66, 1.20) 10.25
USOC 0.73 (0.60, 0.89) 13.08
0.81 (0.66, 0.99)
male
MCS 0.91 (0.66, 1.24) 9.91
NS 0.56 (0.32, 0.97) 5.26
BCS70 1.34 (0.98, 1.82) 10.03
NCDS 0.95 (0.80, 1.13) 13.83
USOC 1.10 (0.88, 1.39) 12.19
1.00 (0.83, 1.19)
Heterogeneity: τ^2 = 0.04, I^2 = 64.84%, H^2 = 2.84
Test of group differences: $Q_b(1)$ = 2.29, p = 0.13
1/2 1
Random-effects REML model

Figure set 24: Fewer fruit and veg

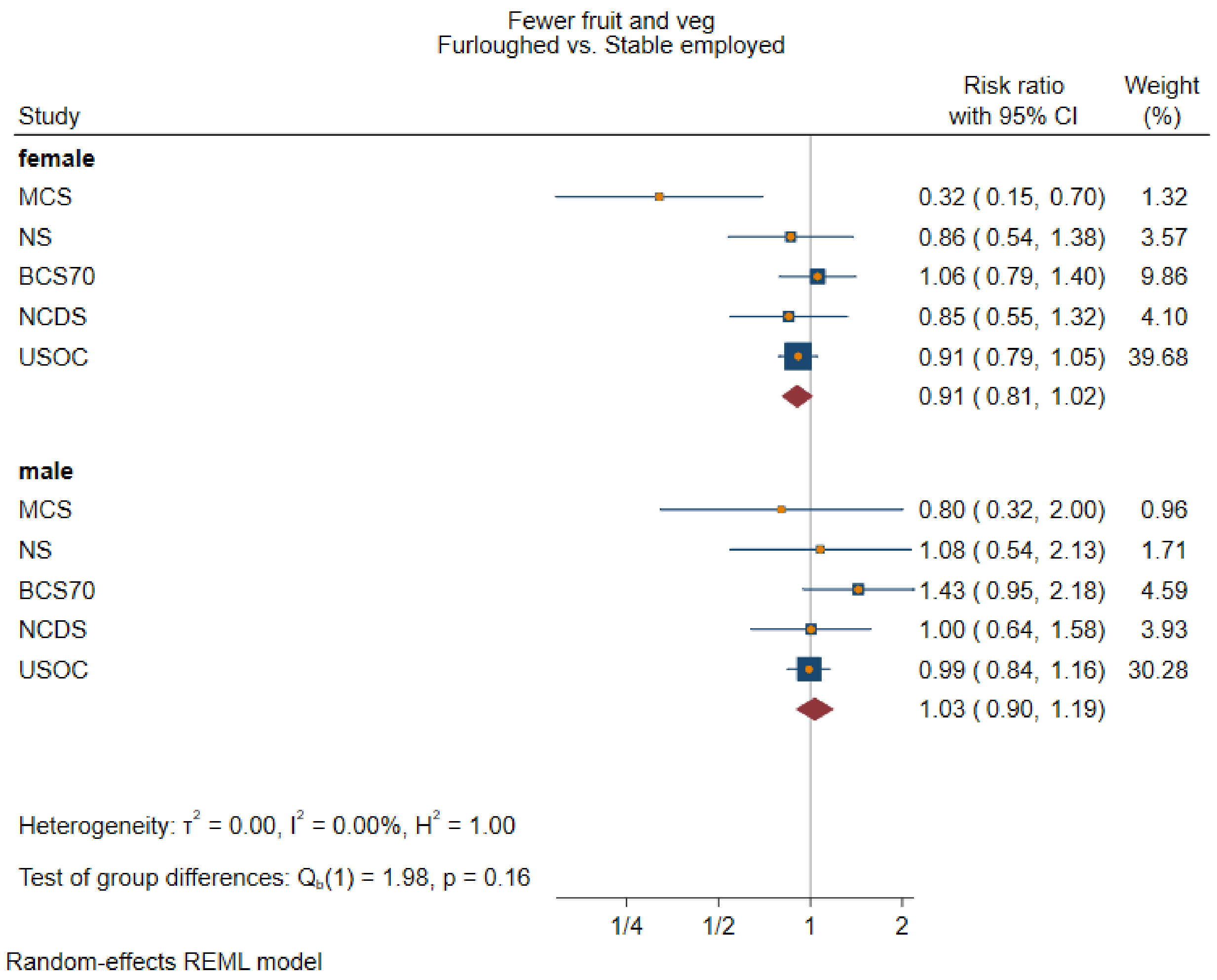

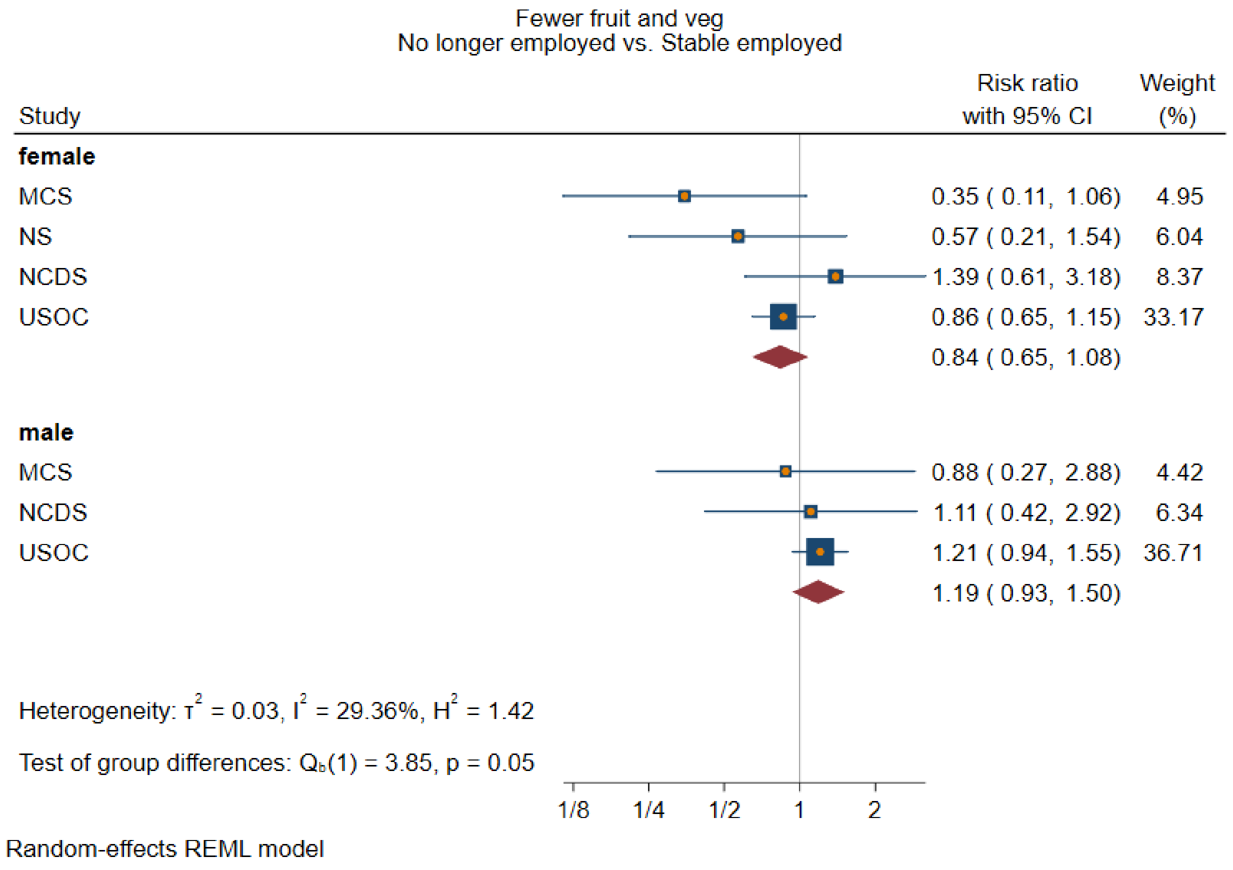

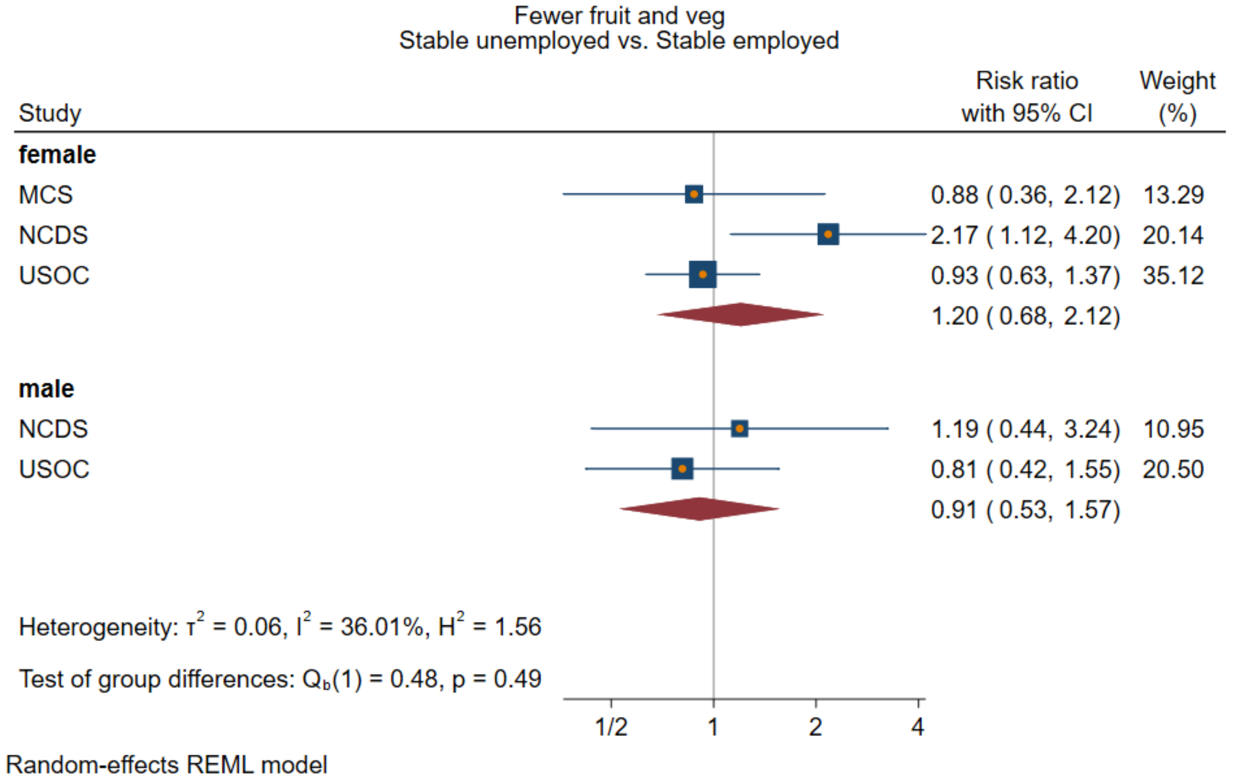
Fewer fruit and veg
Stable unemployed vs. Stable employed
Study
Risk ratio with 95% CI
Weight (%)
female
MCS 0.88 (0.36, 2.12) 13.29
NCDS 2.17 (1.12, 4.20) 20.14
USOC 0.93 (0.63, 1.37) 35.12
1.20 (0.68, 2.12)
male
NCDS 1.19 (0.44, 3.24) 10.95
USOC 0.81 (0.42, 1.55) 20.50
0.91 (0.53, 1.57)
Heterogeneity: τ² = 0.06, I² = 36.01%, H² = 1.56
Test of group differences: Qb(1) = 0.48, p = 0.49
1/2
1
2
4
Random-effects REML model

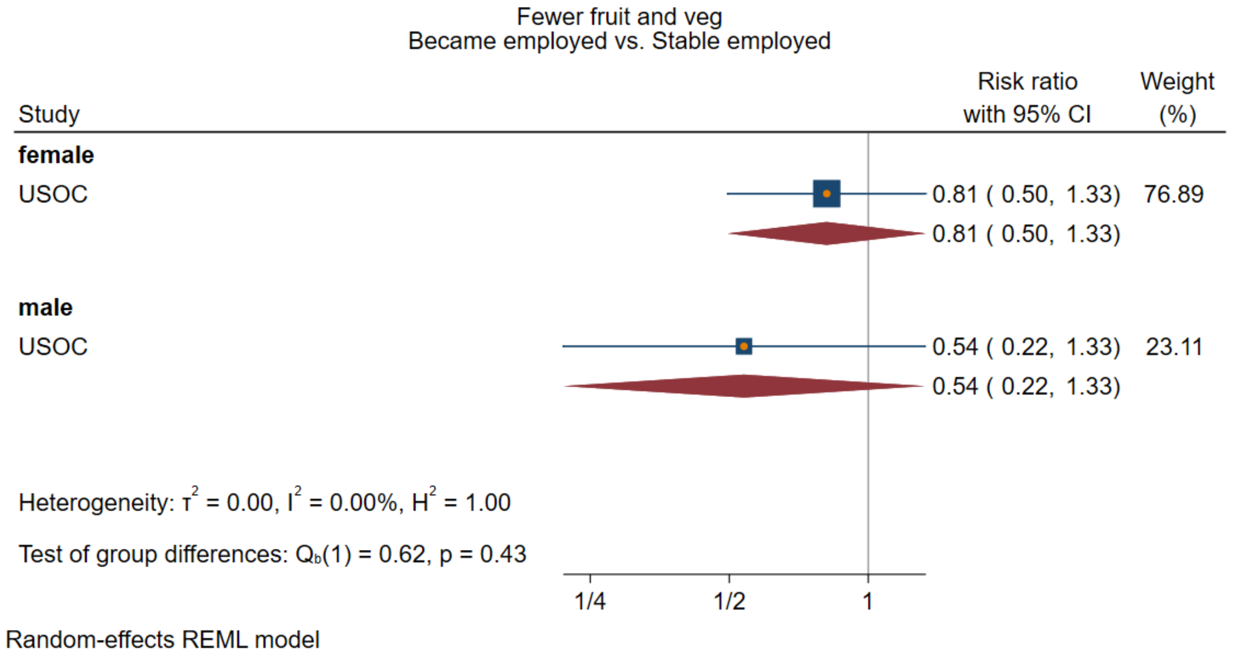
Fewer fruit and veg
Became employed vs. Stable employed
Study
Risk ratio with 95% CI
Weight (%)
female
USOC 0.81 (0.50, 1.33) 76.89
0.81 (0.50, 1.33)
male
USOC 0.54 (0.22, 1.33) 23.11
0.54 (0.22, 1.33)
Heterogeneity: τ² = 0.00, I² = 0.00%, H² = 1.00
Test of group differences: Qb(1) = 0.62, p = 0.43
1/4
1/2
1
Random-effects REML model

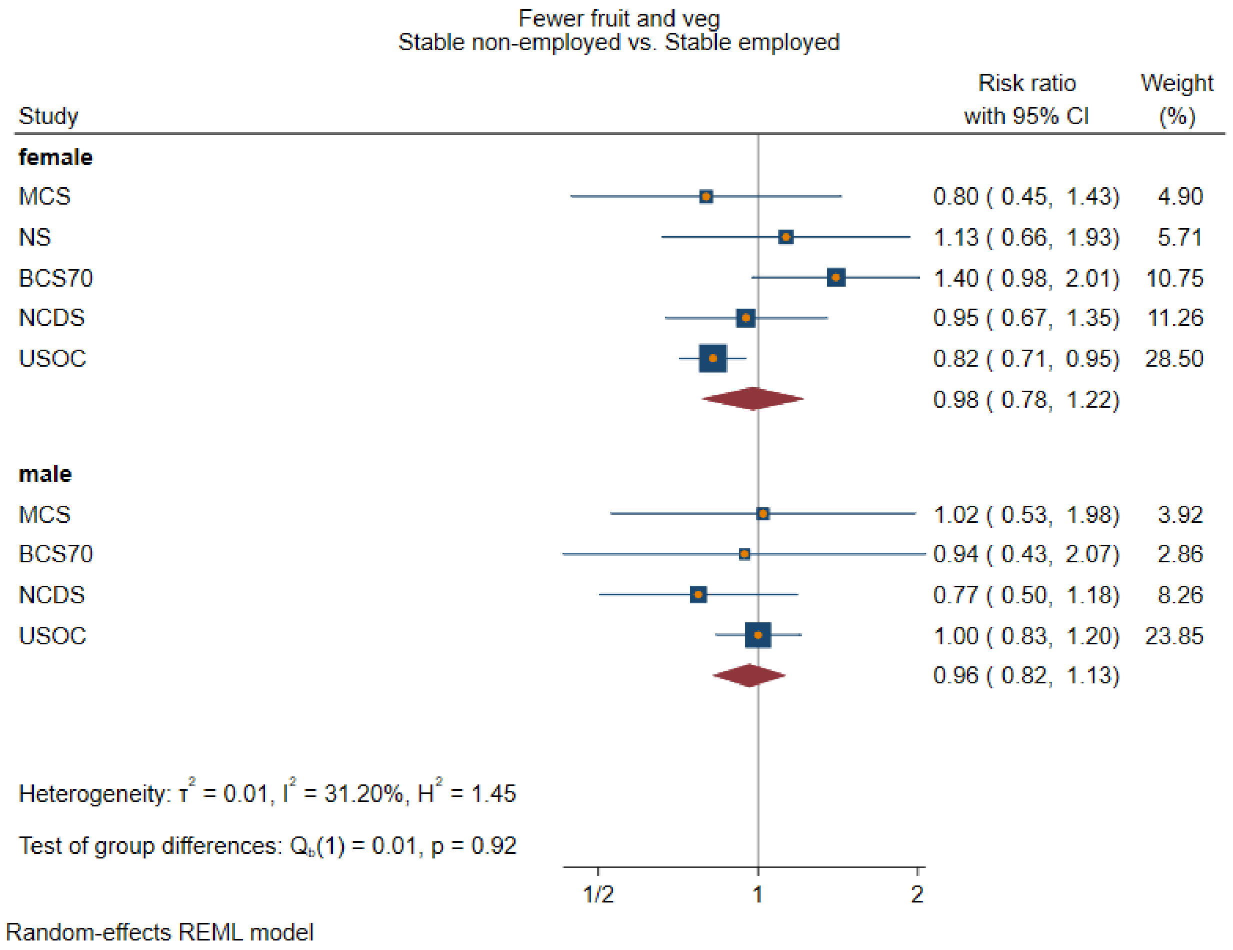
Fewer fruit and veg
Stable non-employed vs. Stable employed
Study
Risk ratio with 95% CI
Weight (%)
female
MCS 0.80 (0.45, 1.43) 4.90
NS 1.13 (0.66, 1.93) 5.71
BCS70 1.40 (0.98, 2.01) 10.75
NCDS 0.95 (0.67, 1.35) 11.26
USOC 0.82 (0.71, 0.95) 28.50
0.98 (0.78, 1.22)
male
MCS 1.02 (0.53, 1.98) 3.92
BCS70 0.94 (0.43, 2.07) 2.86
NCDS 0.77 (0.50, 1.18) 8.26
USOC 1.00 (0.83, 1.20) 23.85
0.96 (0.82, 1.13)
Heterogeneity: τ² = 0.01, I² = 31.20%, H² = 1.45
Test of group differences: Qb(1) = 0.01, p = 0.92
1/2
1
2
Random-effects REML model

Figure set 25: More fruit and veg

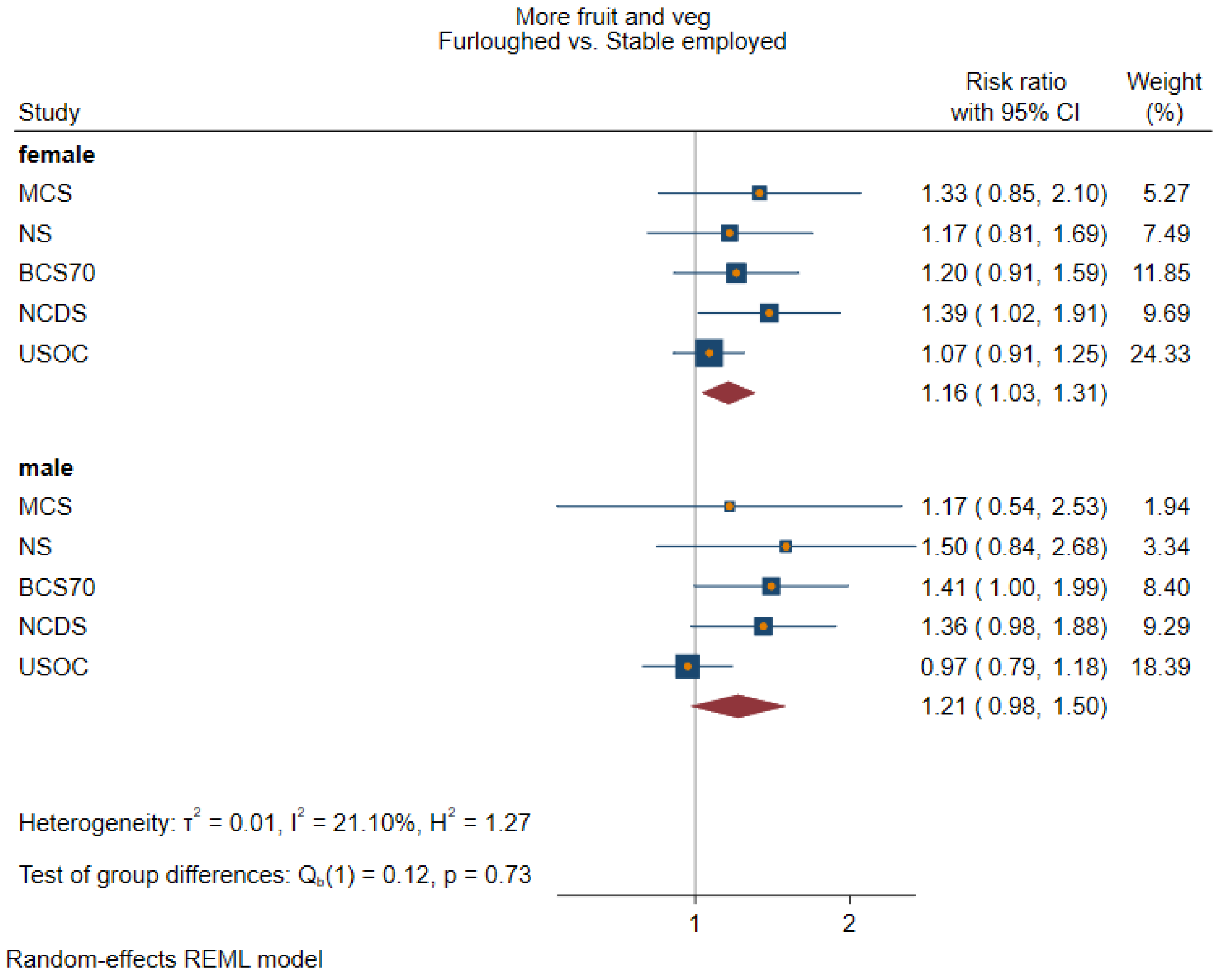

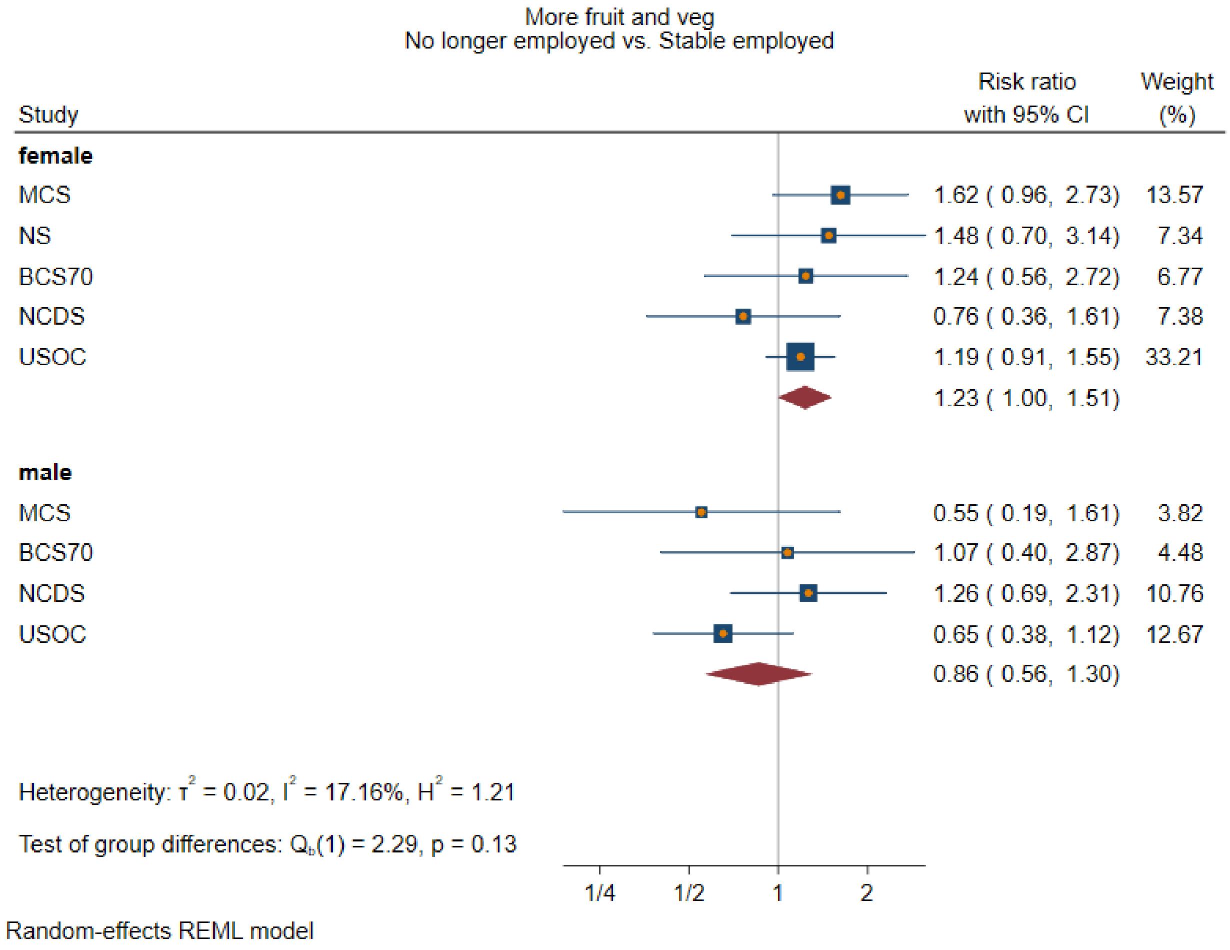

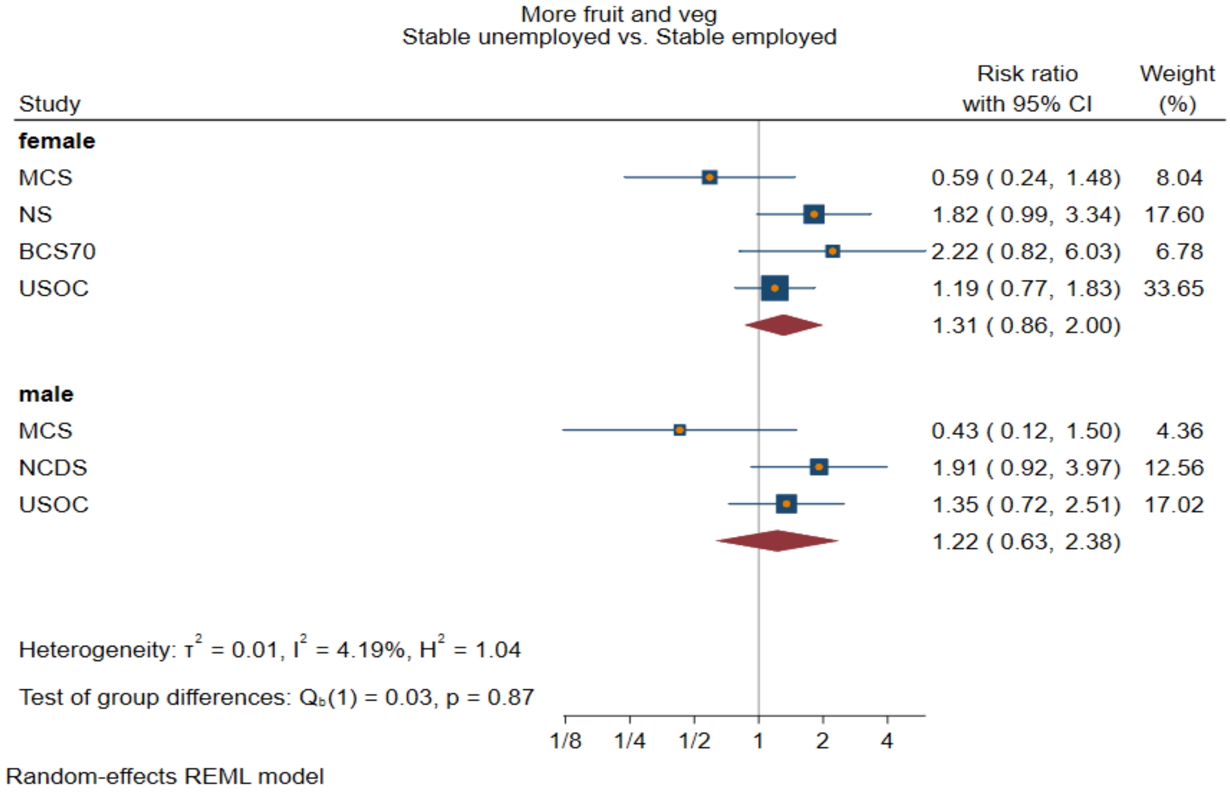
More fruit and veg
Stable unemployed vs. Stable employed
Study
Risk ratio with 95% CI
Weight (%)
female
MCS 0.59 (0.24, 1.48) 8.04
NS 1.82 (0.99, 3.34) 17.60
BCS70 2.22 (0.82, 6.03) 6.78
USOC 1.19 (0.77, 1.83) 33.65
1.31 (0.86, 2.00)
male
MCS 0.43 (0.12, 1.50) 4.36
NCDS 1.91 (0.92, 3.97) 12.56
USOC 1.35 (0.72, 2.51) 17.02
1.22 (0.63, 2.38)
Heterogeneity: τ² = 0.01, I² = 4.19%, H² = 1.04
Test of group differences: Qb(1) = 0.03, p = 0.87
1/8 1/4 1/2 1 2 4
Random-effects REML model

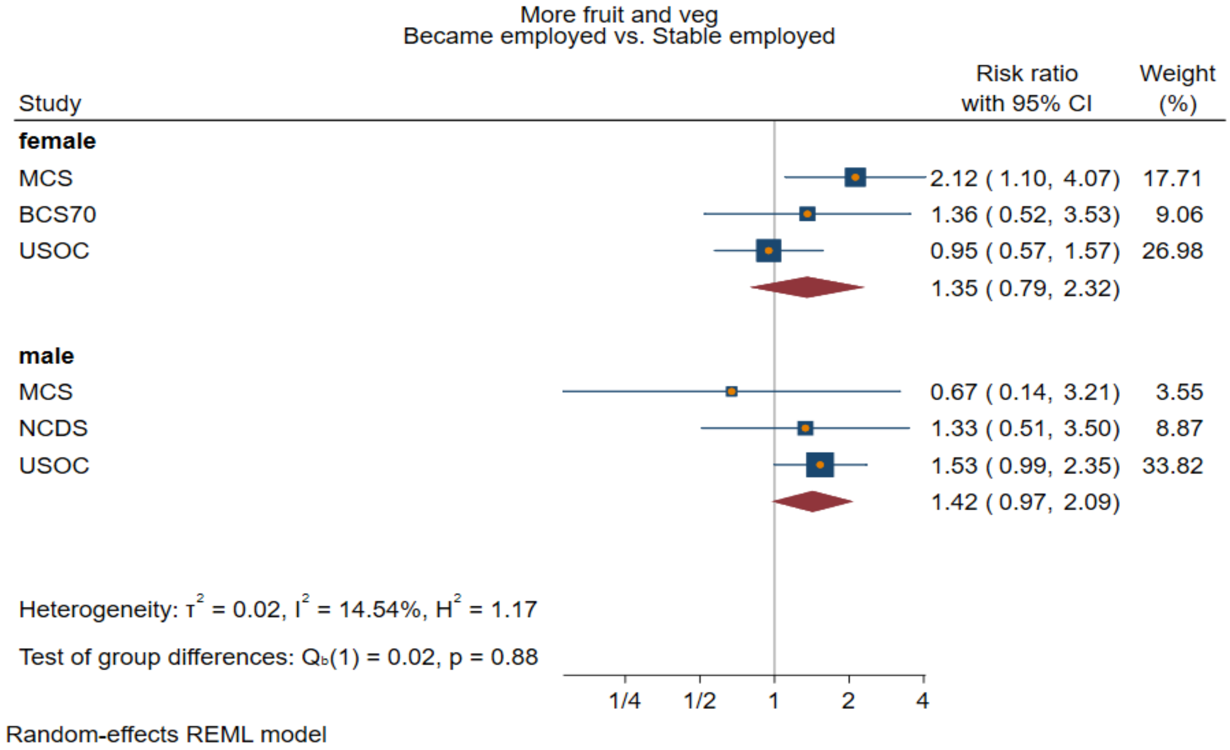
More fruit and veg
Became employed vs. Stable employed
Study
Risk ratio with 95% CI
Weight (%)
female
MCS 2.12 (1.10, 4.07) 17.71
BCS70 1.36 (0.52, 3.53) 9.06
USOC 0.95 (0.57, 1.57) 26.98
1.35 (0.79, 2.32)
male
MCS 0.67 (0.14, 3.21) 3.55
NCDS 1.33 (0.51, 3.50) 8.87
USOC 1.53 (0.99, 2.35) 33.82
1.42 (0.97, 2.09)
Heterogeneity: τ² = 0.02, I² = 14.54%, H² = 1.17
Test of group differences: Qb(1) = 0.02, p = 0.88
1/4 1/2 1 2 4
Random-effects REML model

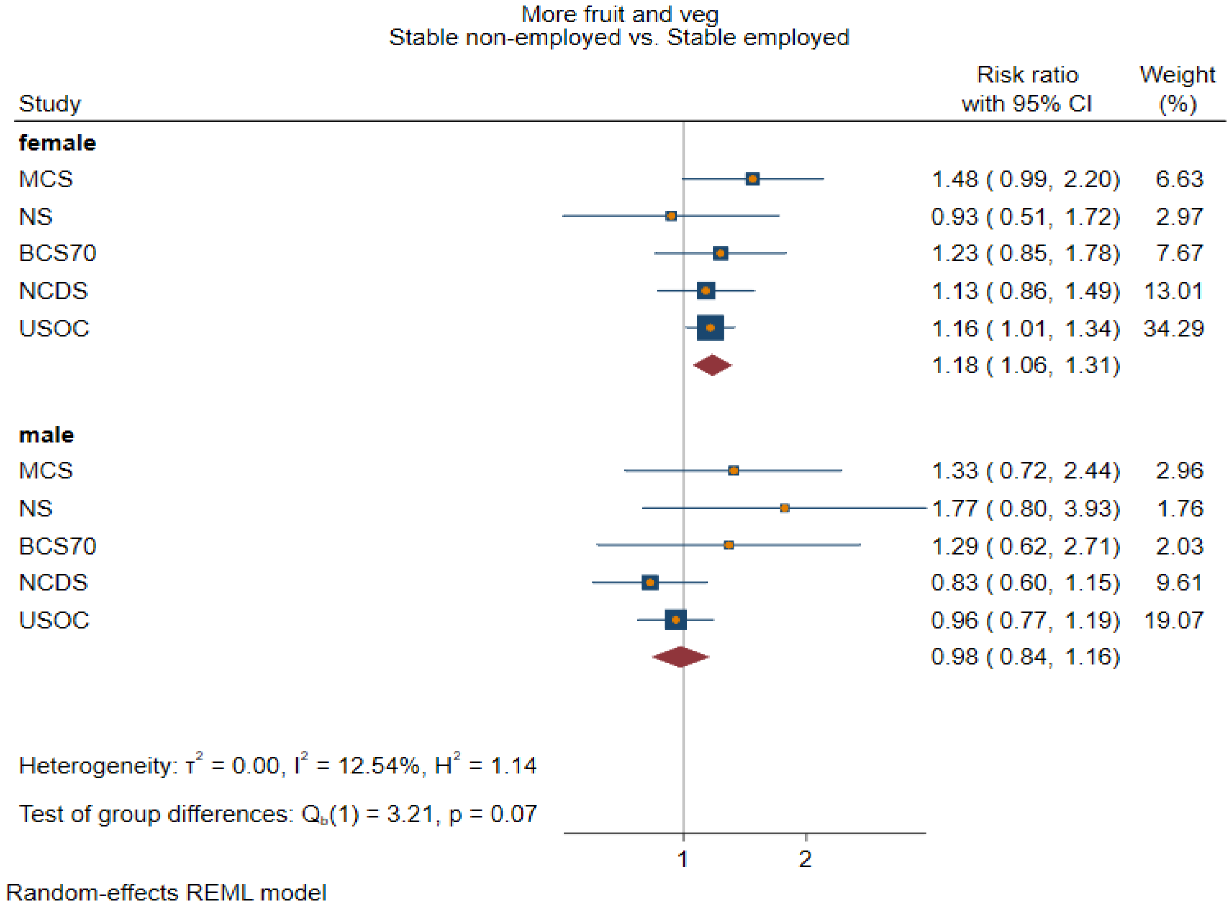
More fruit and veg
Stable non-employed vs. Stable employed
Study
Risk ratio with 95% CI
Weight (%)
female
MCS 1.48 (0.99, 2.20) 6.63
NS 0.93 (0.51, 1.72) 2.97
BCS70 1.23 (0.85, 1.78) 7.67
NCDS 1.13 (0.86, 1.49) 13.01
USOC 1.16 (1.01, 1.34) 34.29
1.18 (1.06, 1.31)
male
MCS 1.33 (0.72, 2.44) 2.96
NS 1.77 (0.80, 3.93) 1.76
BCS70 1.29 (0.62, 2.71) 2.03
NCDS 0.83 (0.60, 1.15) 9.61
USOC 0.96 (0.77, 1.19) 19.07
0.98 (0.84, 1.16)
Heterogeneity: τ² = 0.00, I² = 12.54%, H² = 1.14
Test of group differences: Qb(1) = 3.21, p = 0.07
1 2
Random-effects REML model

Figure set 26: Less than 3 days a week of at least 30min exercise

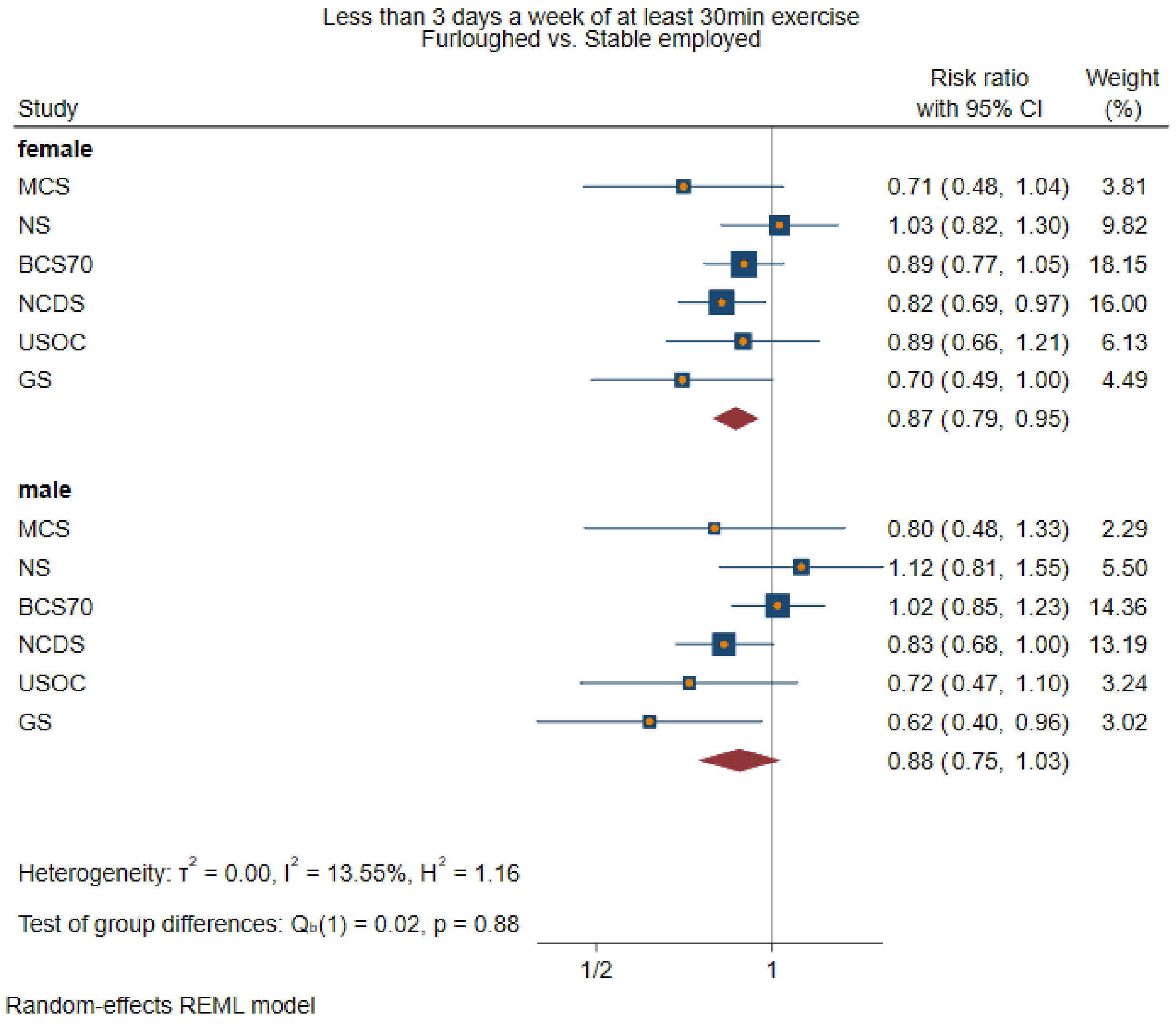

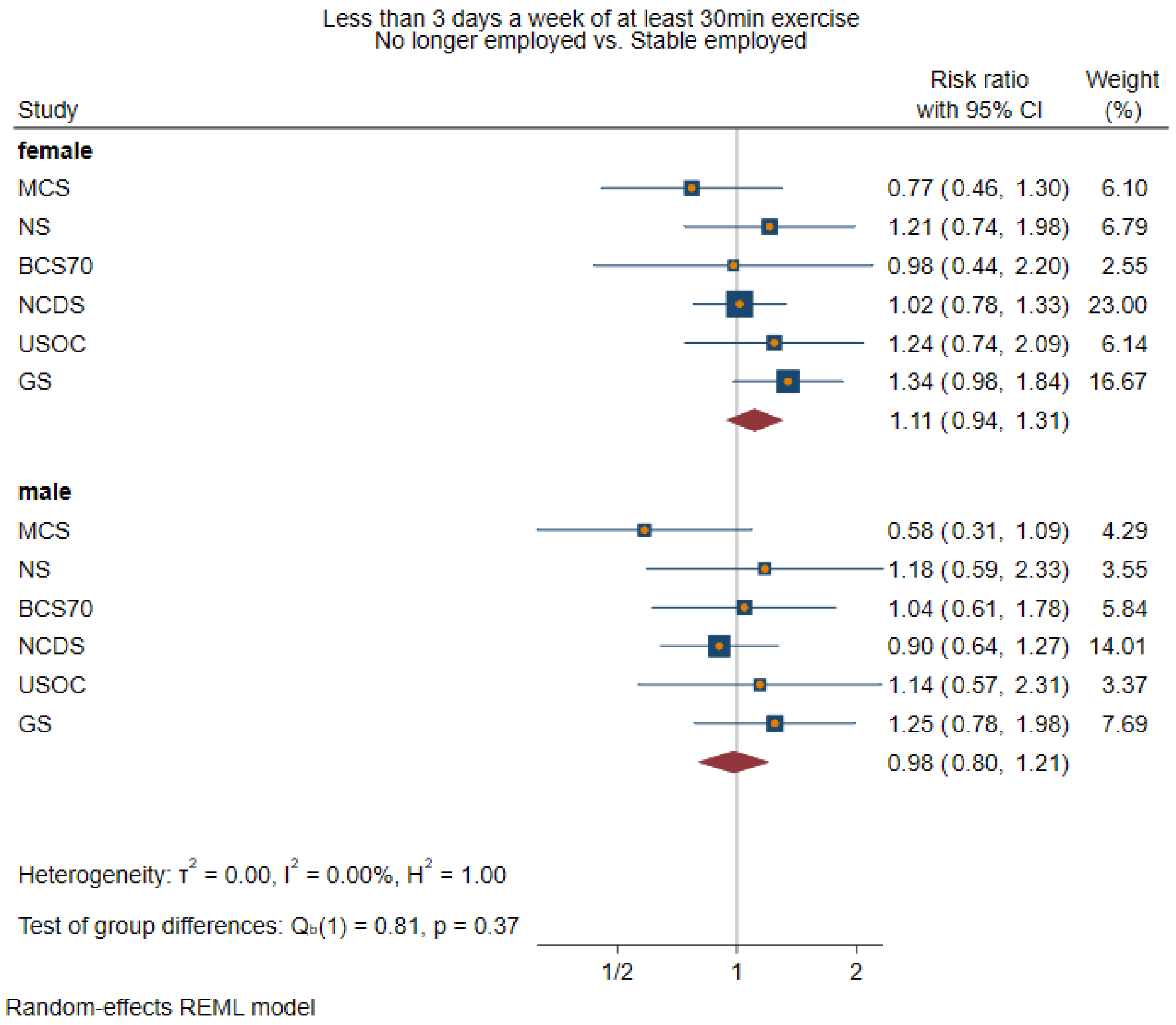

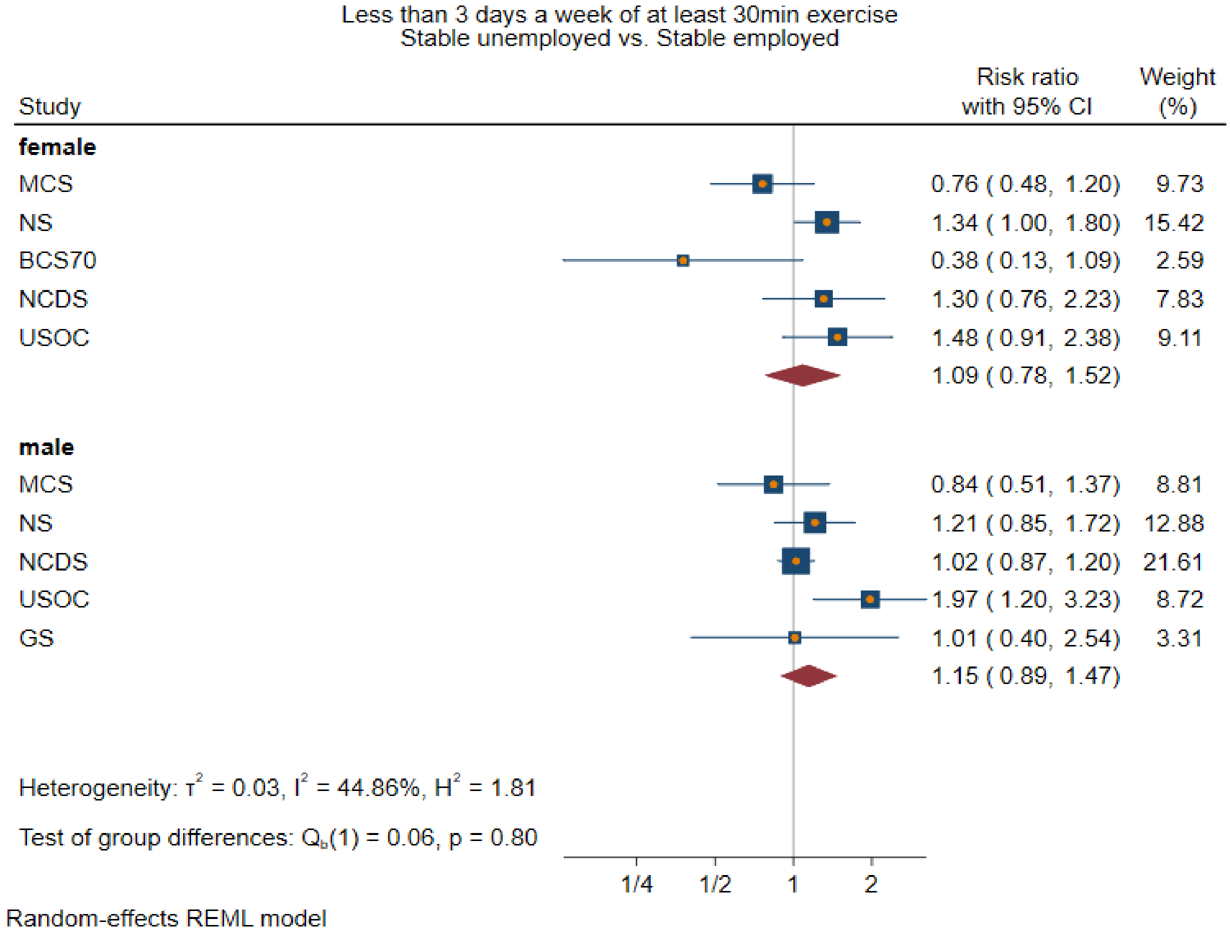
Less than 3 days a week of at least 30min exercise
Stable unemployed vs. Stable employed
Study
Risk ratio with 95% CI
Weight (%)
female
MCS 0.76 (0.48, 1.20) 9.73
NS 1.34 (1.00, 1.80) 15.42
BCS70 0.38 (0.13, 1.09) 2.59
NCDS 1.30 (0.76, 2.23) 7.83
USOC 1.48 (0.91, 2.38) 9.11
1.09 (0.78, 1.52)
male
MCS 0.84 (0.51, 1.37) 8.81
NS 1.21 (0.85, 1.72) 12.88
NCDS 1.02 (0.87, 1.20) 21.61
USOC 1.97 (1.20, 3.23) 8.72
GS 1.01 (0.40, 2.54) 3.31
1.15 (0.89, 1.47)
Heterogeneity: τ² = 0.03, I² = 44.86%, H² = 1.81
Test of group differences: Qb(1) = 0.06, p = 0.80
1/4
1/2
1
2
Random-effects REML model

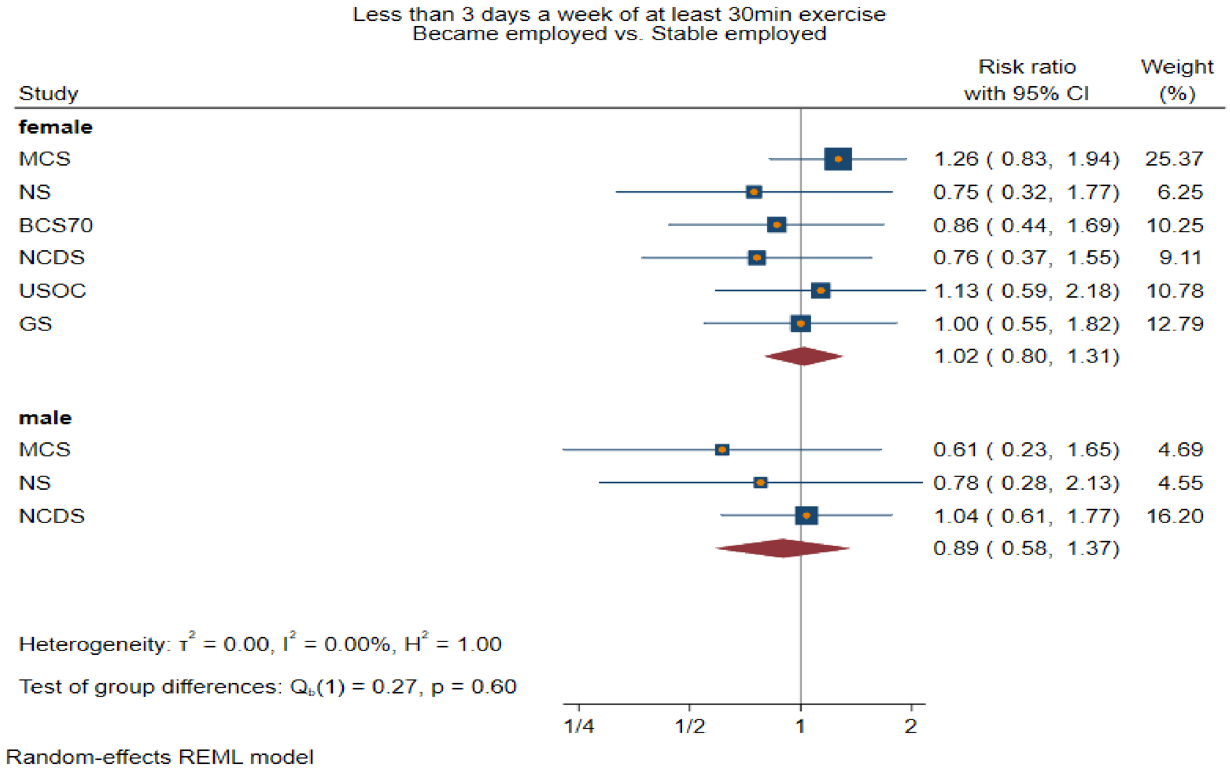
Less than 3 days a week of at least 30min exercise
Became employed vs. Stable employed
Study
Risk ratio with 95% CI
Weight (%)
female
MCS 1.26 (0.83, 1.94) 25.37
NS 0.75 (0.32, 1.77) 6.25
BCS70 0.86 (0.44, 1.69) 10.25
NCDS 0.76 (0.37, 1.55) 9.11
USOC 1.13 (0.59, 2.18) 10.78
GS 1.00 (0.55, 1.82) 12.79
1.02 (0.80, 1.31)
male
MCS 0.61 (0.23, 1.65) 4.69
NS 0.78 (0.28, 2.13) 4.55
NCDS 1.04 (0.61, 1.77) 16.20
0.89 (0.58, 1.37)
Heterogeneity: τ² = 0.00, I² = 0.00%, H² = 1.00
Test of group differences: Qb(1) = 0.27, p = 0.60
1/4
1/2
1
2
Random-effects REML model

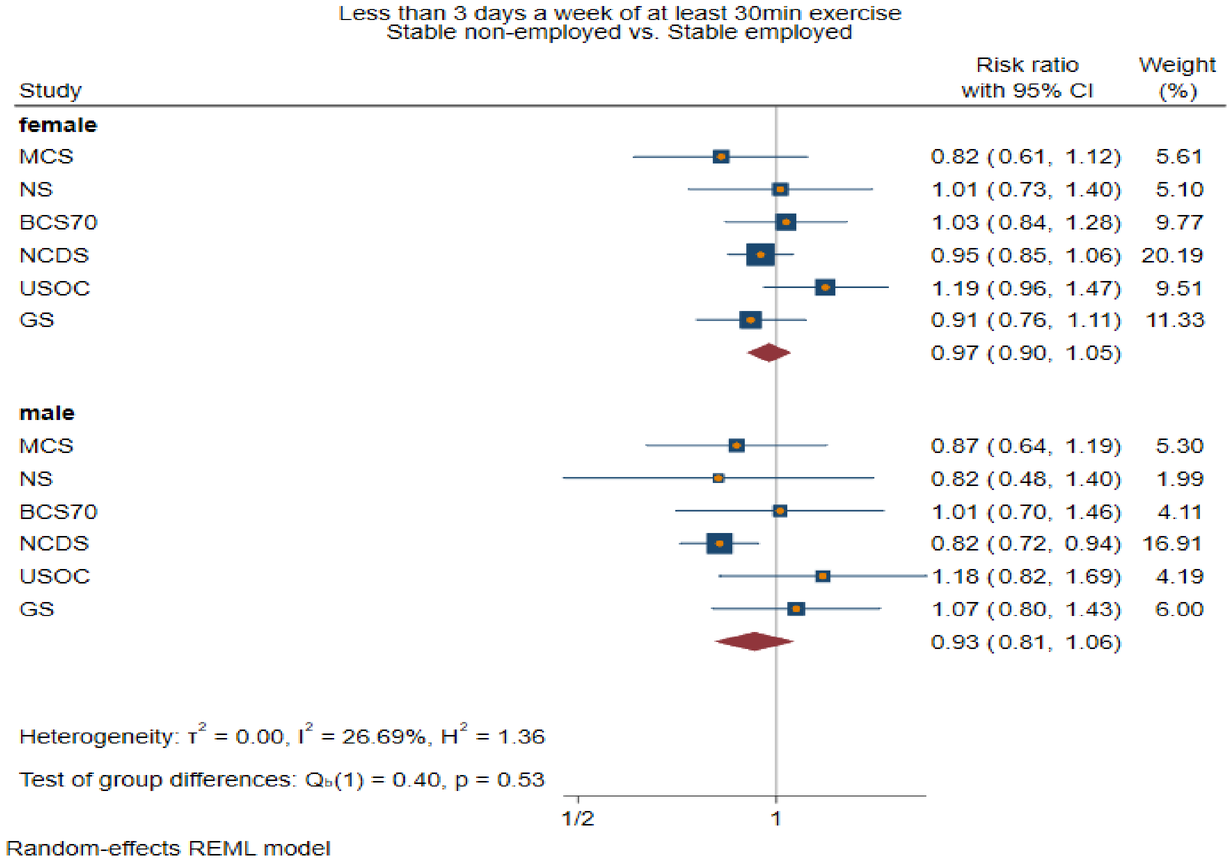
Less than 3 days a week of at least 30min exercise
Stable non-employed vs. Stable employed
Study
Risk ratio with 95% CI
Weight (%)
female
MCS 0.82 (0.61, 1.12) 5.61
NS 1.01 (0.73, 1.40) 5.10
BCS70 1.03 (0.84, 1.28) 9.77
NCDS 0.95 (0.85, 1.06) 20.19
USOC 1.19 (0.96, 1.47) 9.51
GS 0.91 (0.76, 1.11) 11.33
0.97 (0.90, 1.05)
male
MCS 0.87 (0.64, 1.19) 5.30
NS 0.82 (0.48, 1.40) 1.99
BCS70 1.01 (0.70, 1.46) 4.11
NCDS 0.82 (0.72, 0.94) 16.91
USOC 1.18 (0.82, 1.69) 4.19
GS 1.07 (0.80, 1.43) 6.00
0.93 (0.81, 1.06)
Heterogeneity: τ² = 0.00, I² = 26.69%, H² = 1.36
Test of group differences: Qb(1) = 0.40, p = 0.53
1/2
1
Random-effects REML model

Figure set 27: Less time/fewer days of physical exercise

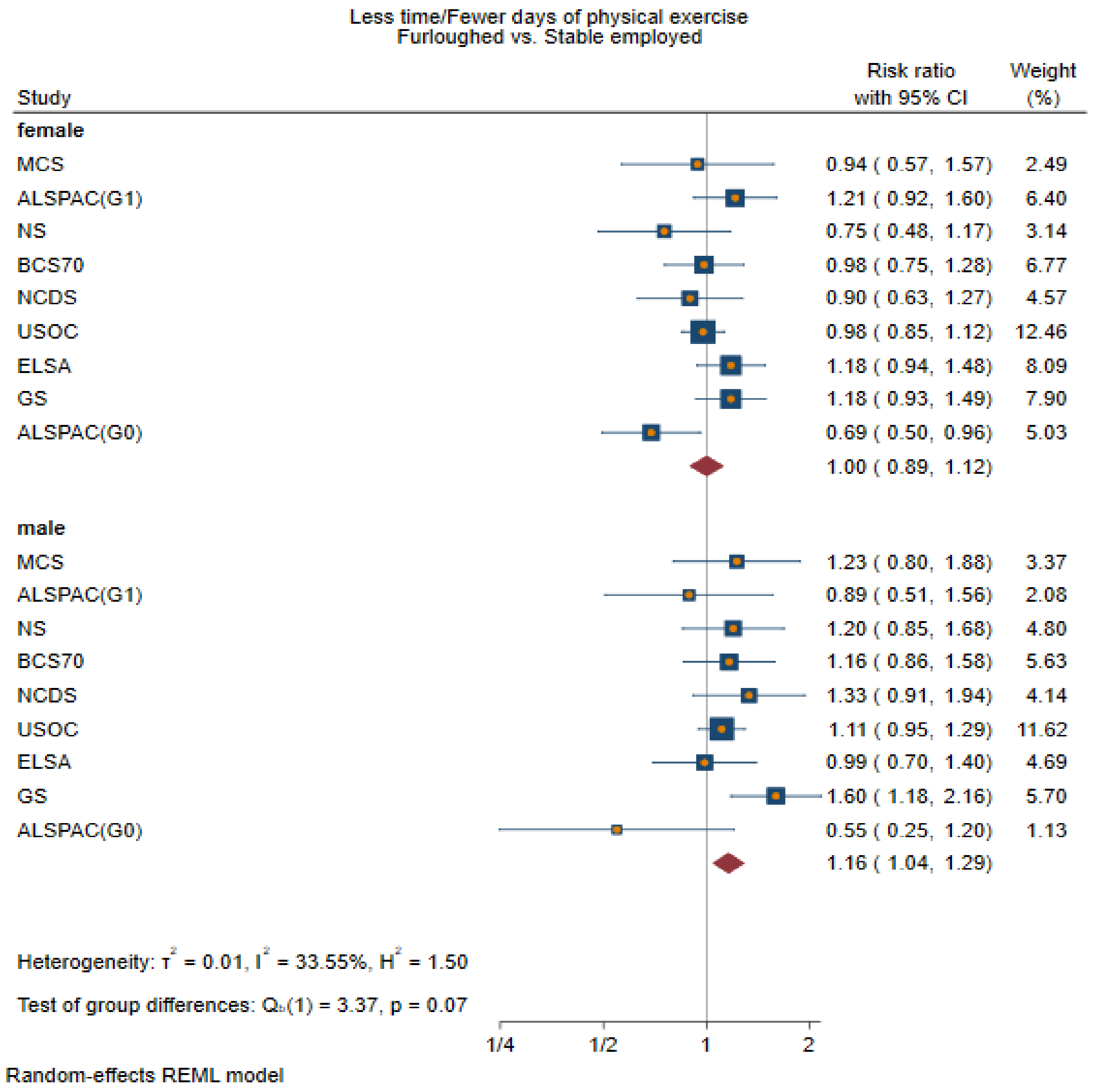

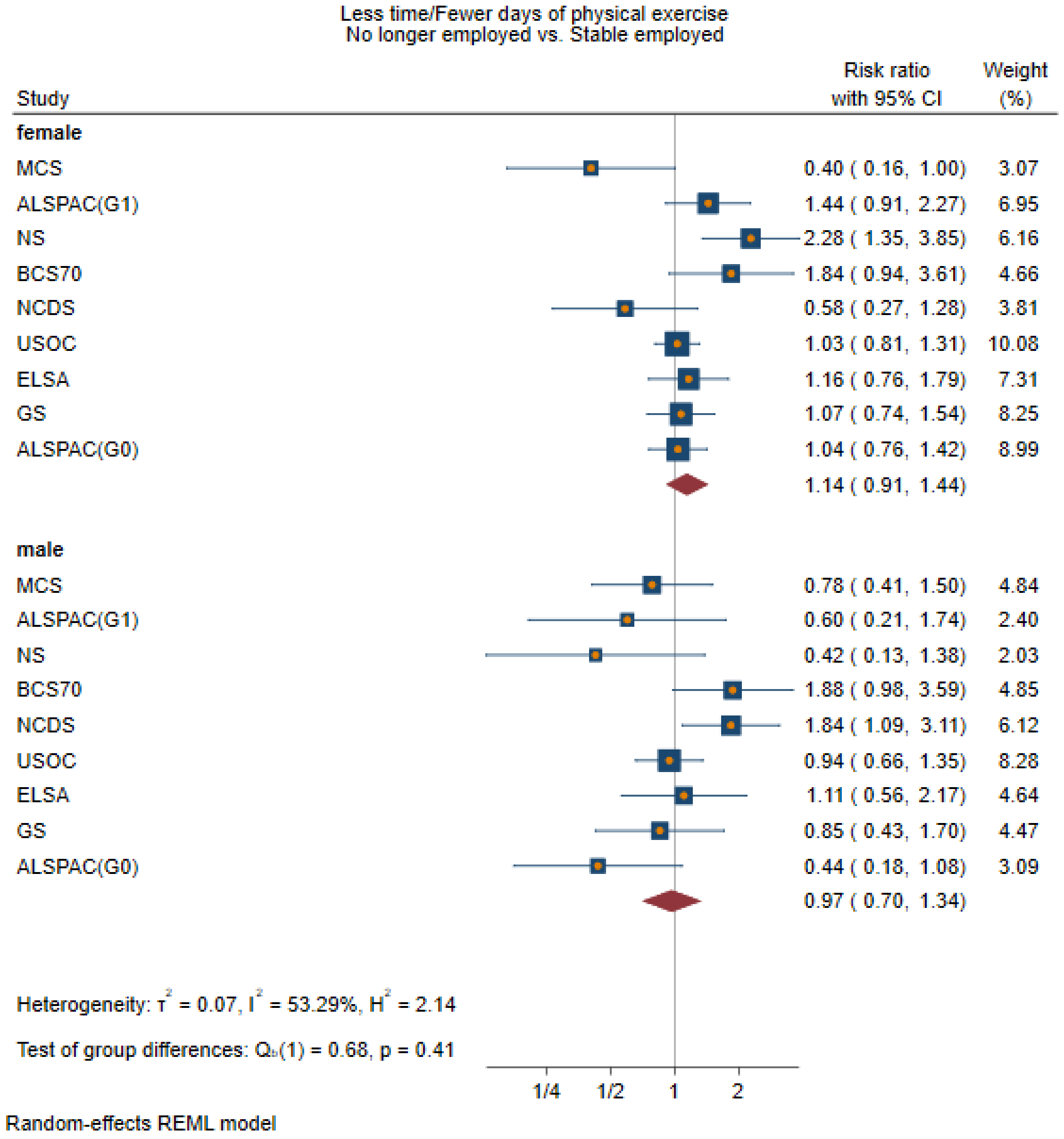

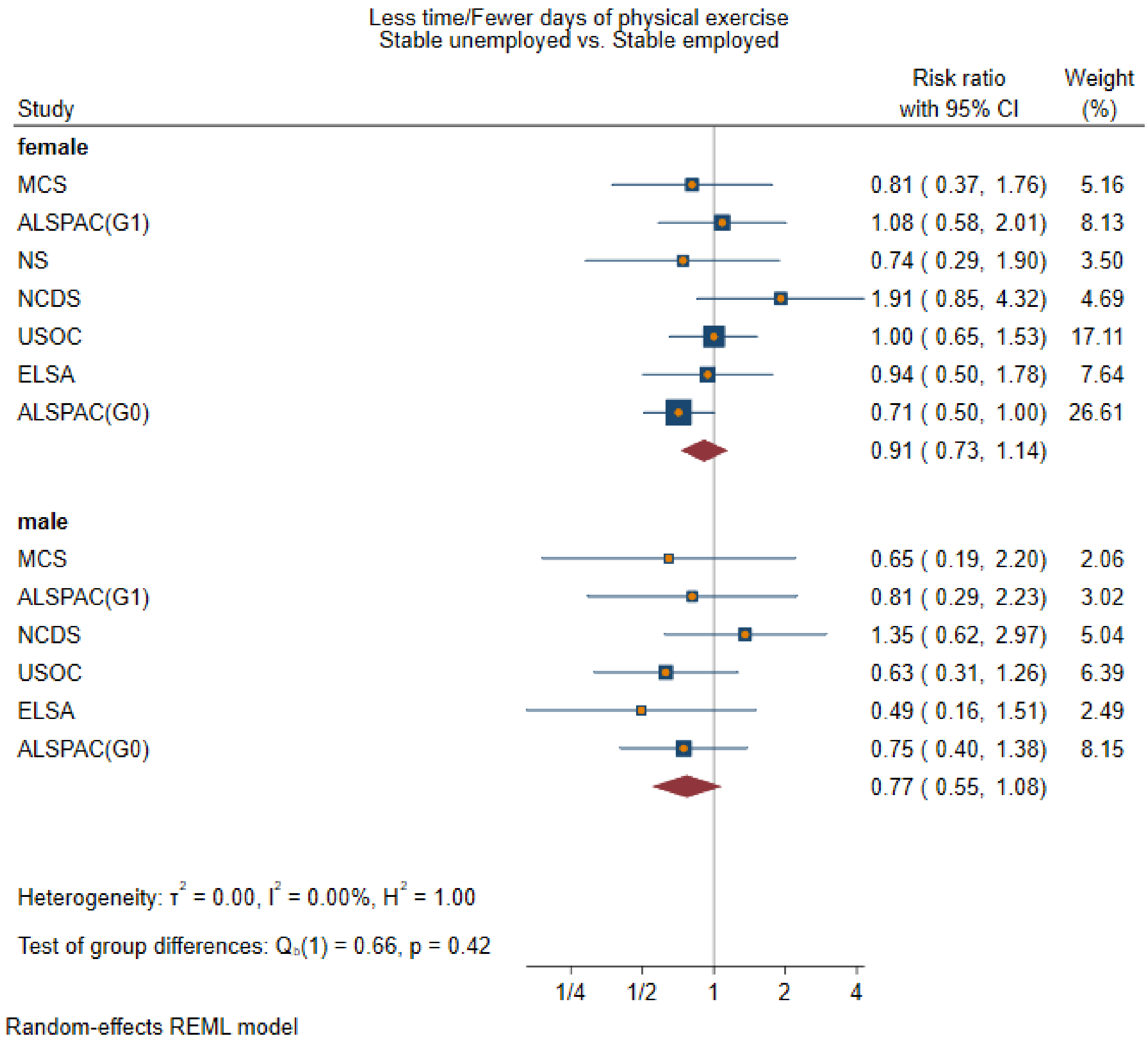
Less time/Fewer days of physical exercise
Stable unemployed vs. Stable employed
Study
Risk ratio with 95% CI
Weight (%)
female
MCS 0.81 (0.37, 1.76) 5.16
ALSPAC(G1) 1.08 (0.58, 2.01) 8.13
NS 0.74 (0.29, 1.90) 3.50
NCDS 1.91 (0.85, 4.32) 4.69
USOC 1.00 (0.65, 1.53) 17.11
ELSA 0.94 (0.50, 1.78) 7.64
ALSPAC(G0) 0.71 (0.50, 1.00) 26.61
0.91 (0.73, 1.14)
male
MCS 0.65 (0.19, 2.20) 2.06
ALSPAC(G1) 0.81 (0.29, 2.23) 3.02
NCDS 1.35 (0.62, 2.97) 5.04
USOC 0.63 (0.31, 1.26) 6.39
ELSA 0.49 (0.16, 1.51) 2.49
ALSPAC(G0) 0.75 (0.40, 1.38) 8.15
0.77 (0.55, 1.08)
Heterogeneity: τ^2 = 0.00, I^2 = 0.00%, H^2 = 1.00
Test of group differences: $Q_b(1)$ = 0.66, p = 0.42
1/4
1/2
1
2
4
Random-effects REML model

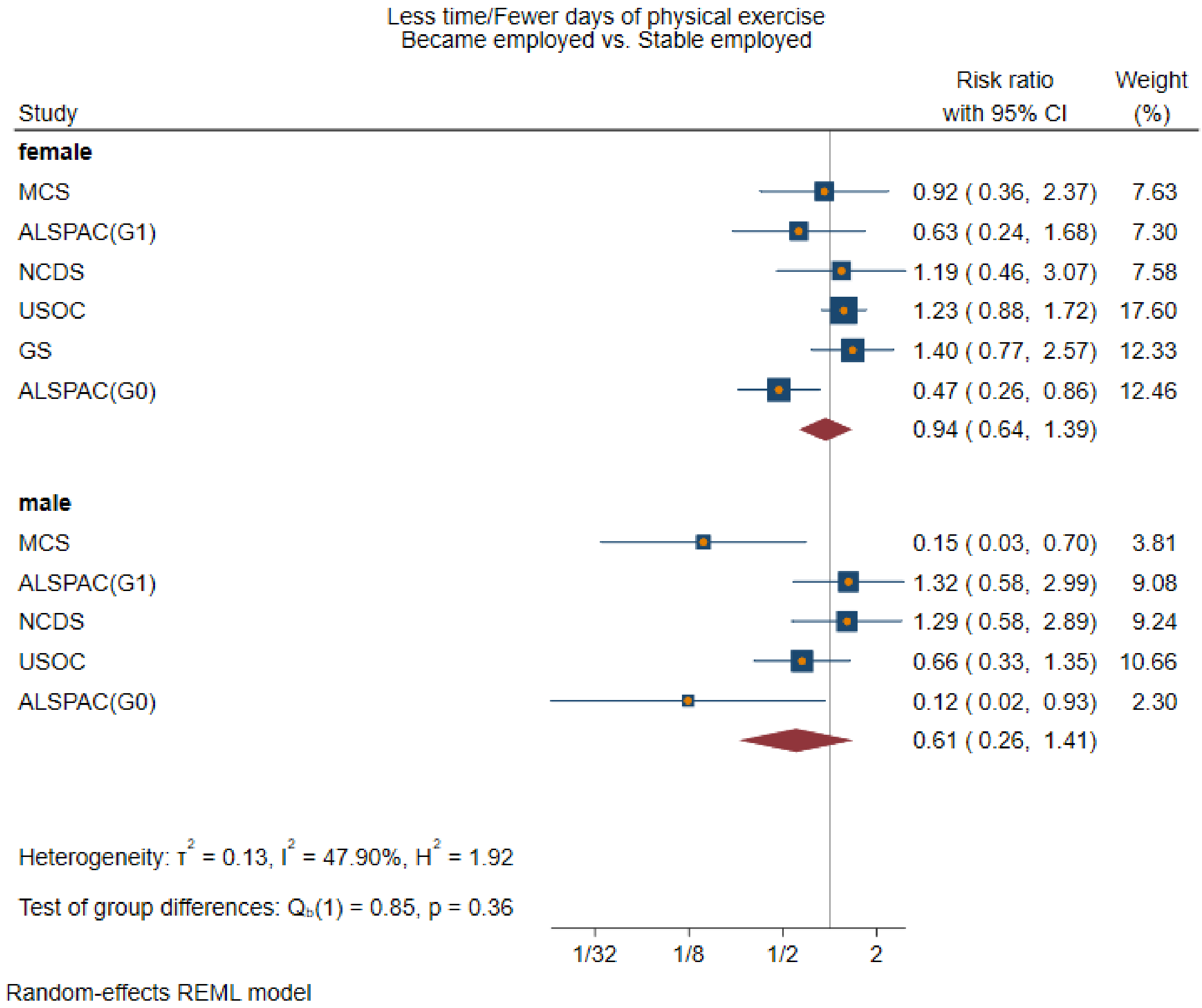
Less time/Fewer days of physical exercise
Became employed vs. Stable employed
Study
Risk ratio with 95% CI
Weight (%)
female
MCS 0.92 (0.36, 2.37) 7.63
ALSPAC(G1) 0.63 (0.24, 1.68) 7.30
NCDS 1.19 (0.46, 3.07) 7.58
USOC 1.23 (0.88, 1.72) 17.60
GS 1.40 (0.77, 2.57) 12.33
ALSPAC(G0) 0.47 (0.26, 0.86) 12.46
0.94 (0.64, 1.39)
male
MCS 0.15 (0.03, 0.70) 3.81
ALSPAC(G1) 1.32 (0.58, 2.99) 9.08
NCDS 1.29 (0.58, 2.89) 9.24
USOC 0.66 (0.33, 1.35) 10.66
ALSPAC(G0) 0.12 (0.02, 0.93) 2.30
0.61 (0.26, 1.41)
Heterogeneity: τ^2 = 0.13, I^2 = 47.90%, H^2 = 1.92
Test of group differences: $Q_b(1)$ = 0.85, p = 0.36
1/32
1/8
1/2
2
Random-effects REML model

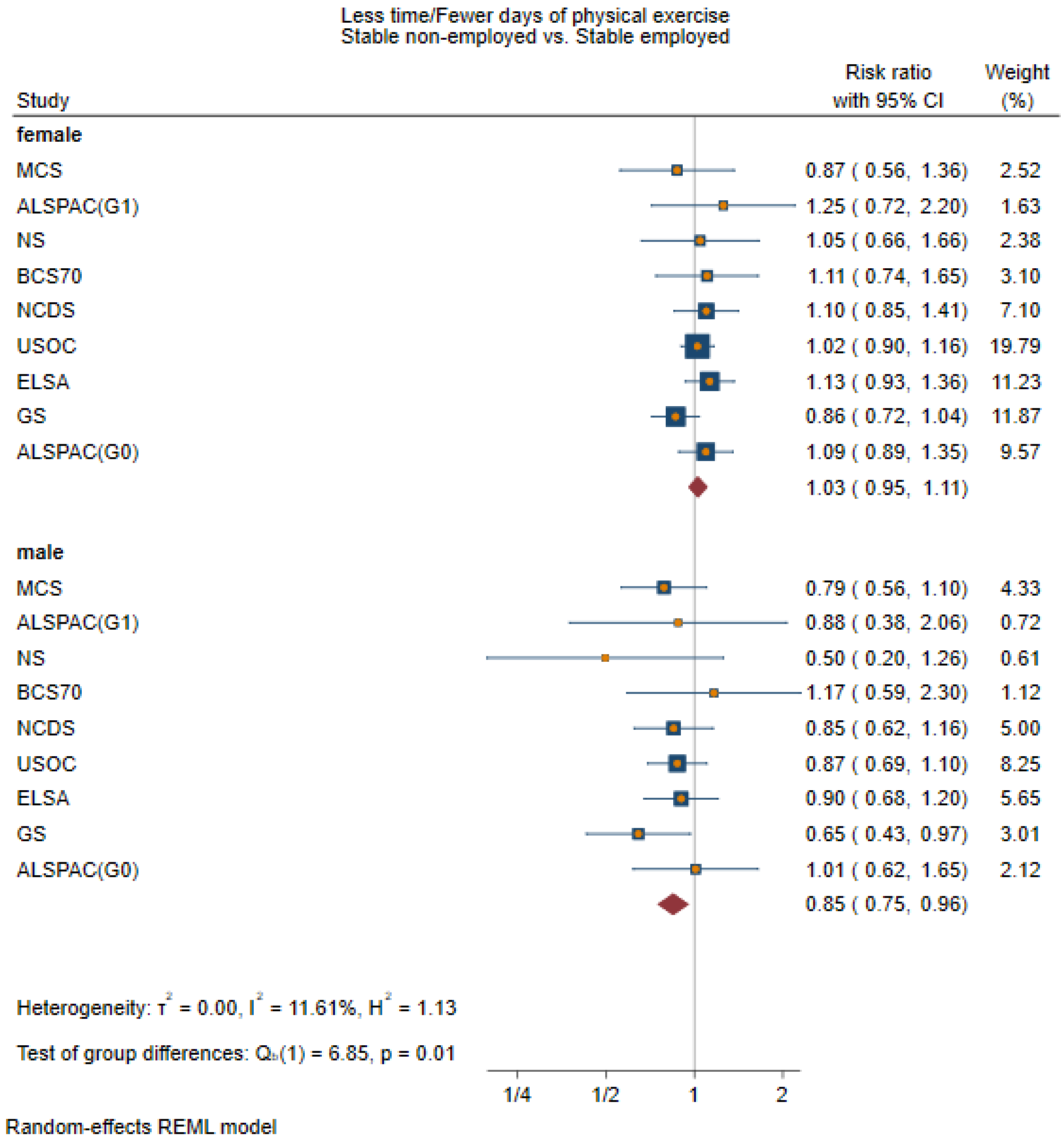
Less time/Fewer days of physical exercise
Stable non-employed vs. Stable employed
Study
Risk ratio with 95% CI
Weight (%)
female
MCS 0.87 (0.56, 1.36) 2.52
ALSPAC(G1) 1.25 (0.72, 2.20) 1.63
NS 1.05 (0.66, 1.66) 2.38
BCS70 1.11 (0.74, 1.65) 3.10
NCDS 1.10 (0.85, 1.41) 7.10
USOC 1.02 (0.90, 1.16) 19.79
ELSA 1.13 (0.93, 1.36) 11.23
GS 0.86 (0.72, 1.04) 11.87
ALSPAC(G0) 1.09 (0.89, 1.35) 9.57
1.03 (0.95, 1.11)
male
MCS 0.79 (0.56, 1.10) 4.33
ALSPAC(G1) 0.88 (0.38, 2.06) 0.72
NS 0.50 (0.20, 1.26) 0.61
BCS70 1.17 (0.59, 2.30) 1.12
NCDS 0.85 (0.62, 1.16) 5.00
USOC 0.87 (0.69, 1.10) 8.25
ELSA 0.90 (0.68, 1.20) 5.65
GS 0.65 (0.43, 0.97) 3.01
ALSPAC(G0) 1.01 (0.62, 1.65) 2.12
0.85 (0.75, 0.96)
Heterogeneity: τ² = 0.00, I² = 11.61%, H² = 1.13
Test of group differences: Qb(1) = 6.85, p = 0.01
1/4
1/2
1
2
Random-effects REML model

Figure set 28: More time/ days of physical exercise

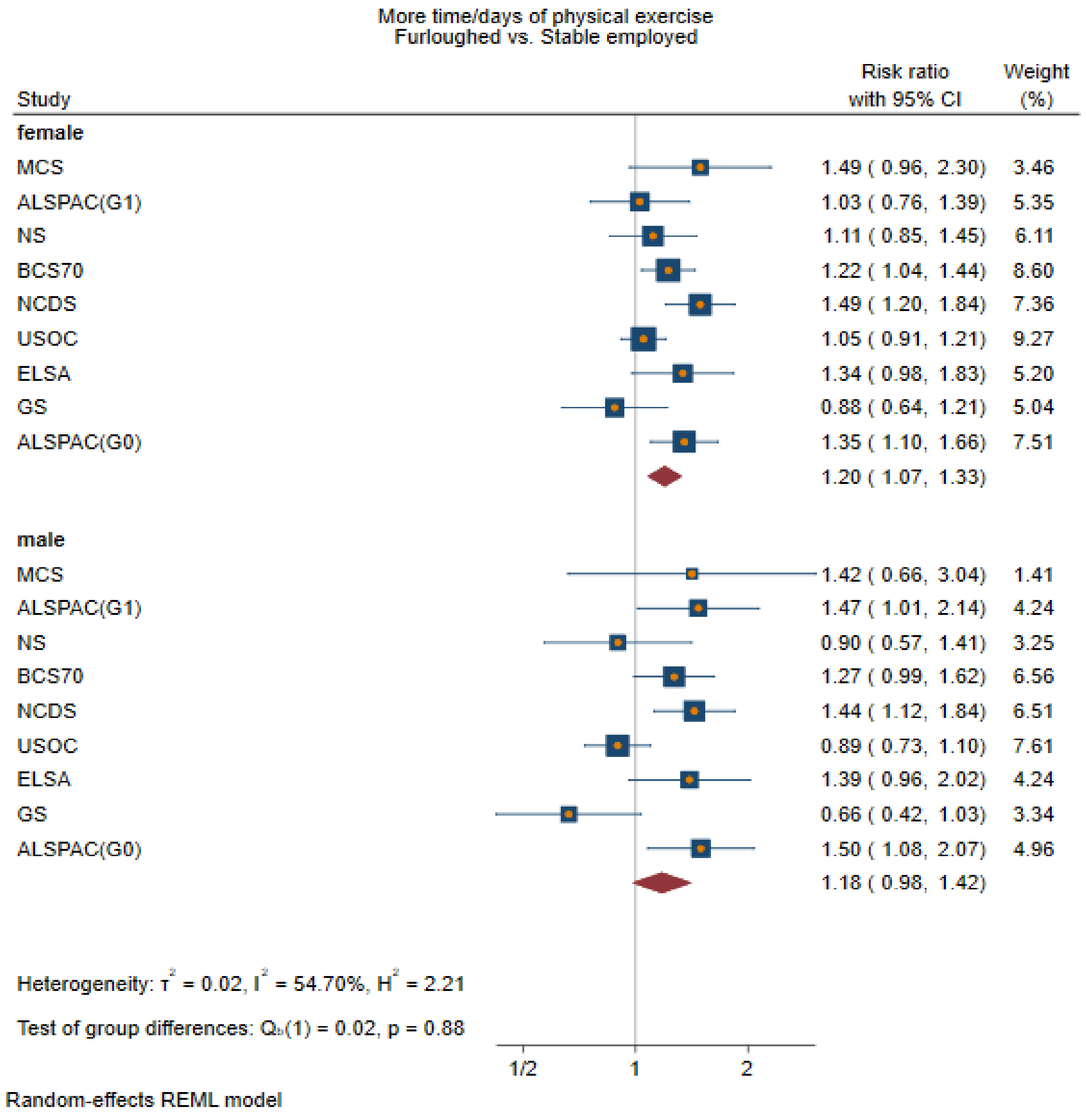

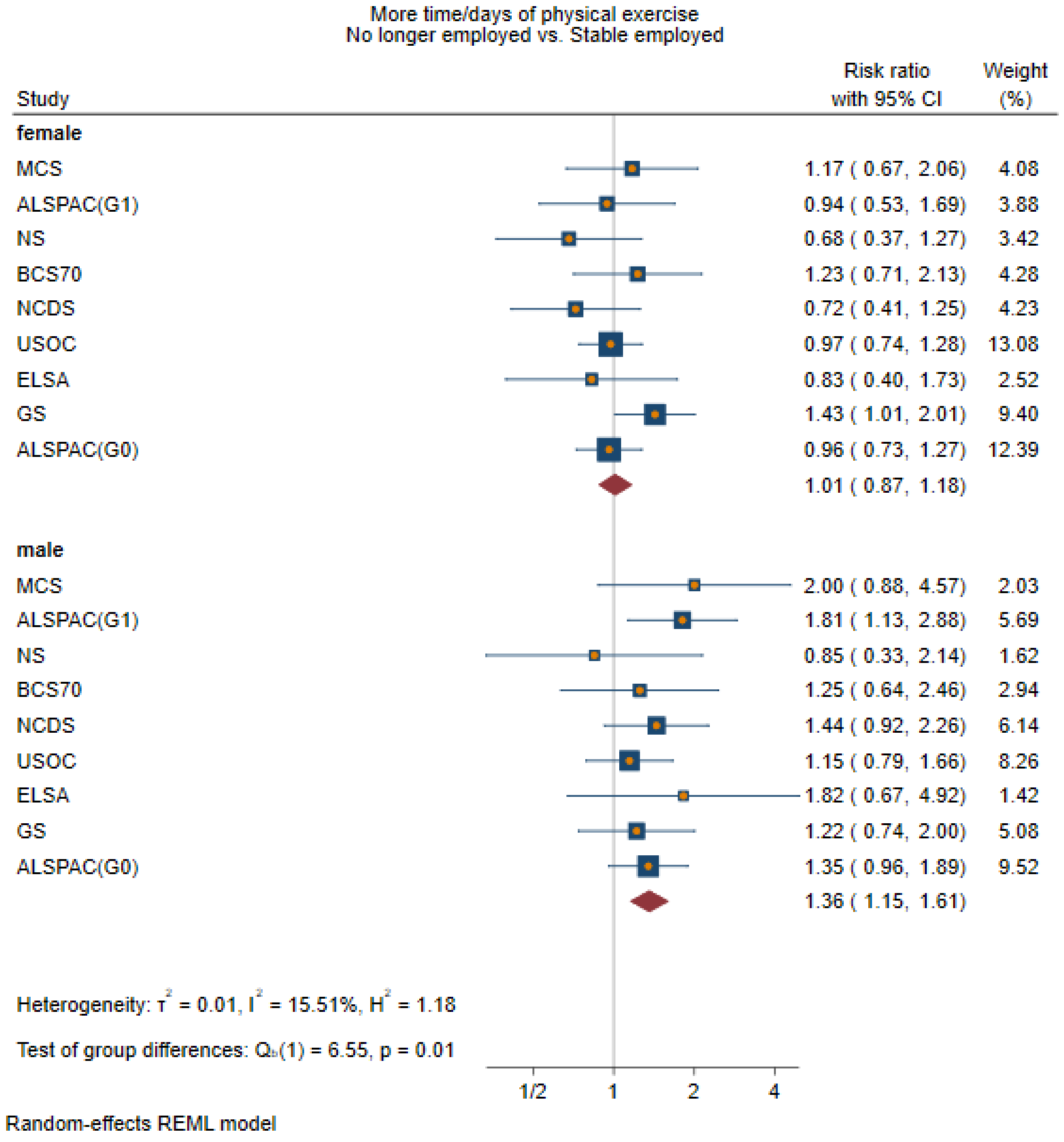

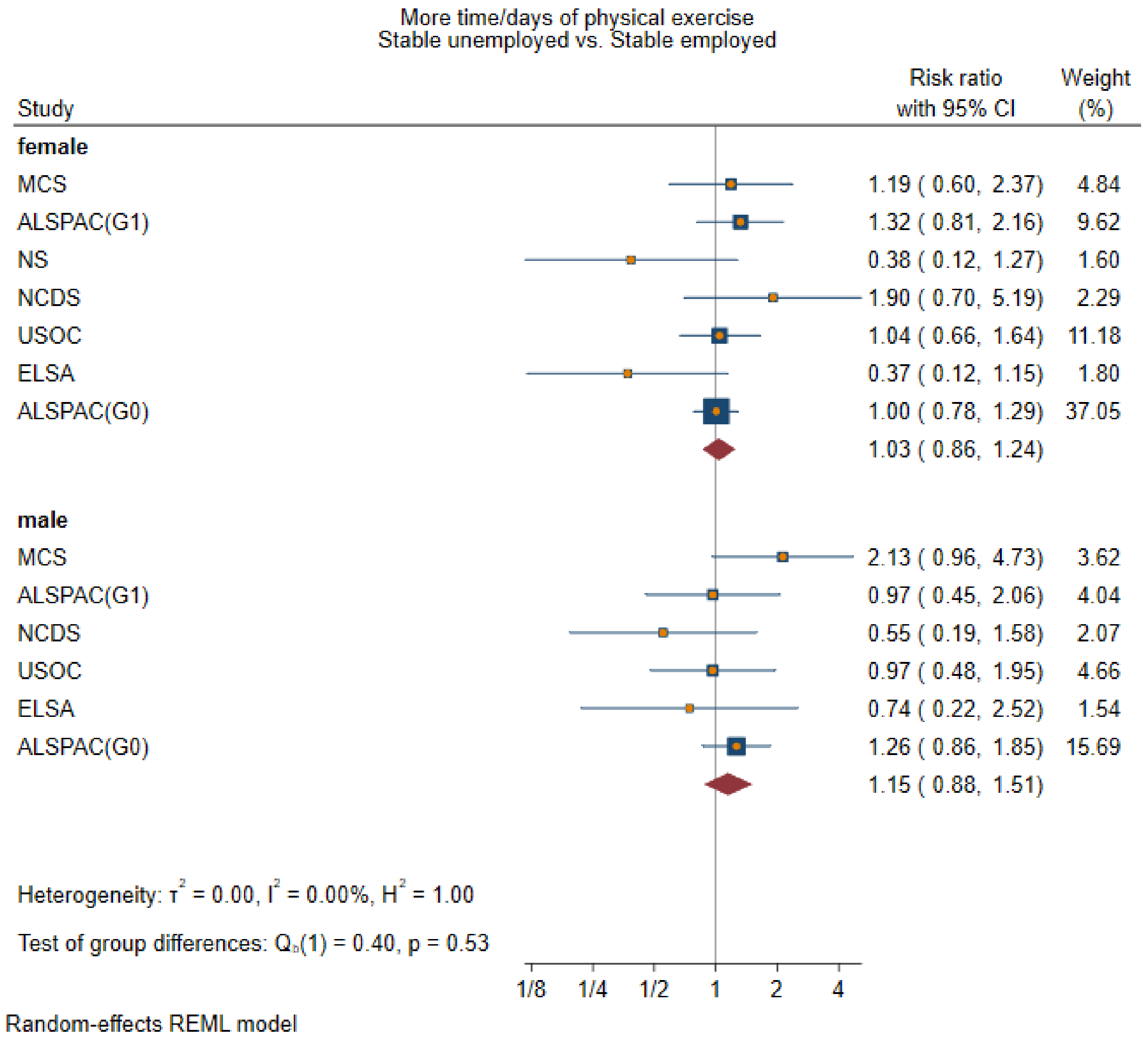
More time/days of physical exercise
Stable unemployed vs. Stable employed
Study
Risk ratio with 95% CI
Weight (%)
female
MCS 1.19 (0.60, 2.37) 4.84
ALSPAC(G1) 1.32 (0.81, 2.16) 9.62
NS 0.38 (0.12, 1.27) 1.60
NCDS 1.90 (0.70, 5.19) 2.29
USOC 1.04 (0.66, 1.64) 11.18
ELSA 0.37 (0.12, 1.15) 1.80
ALSPAC(G0) 1.00 (0.78, 1.29) 37.05
1.03 (0.86, 1.24)
male
MCS 2.13 (0.96, 4.73) 3.62
ALSPAC(G1) 0.97 (0.45, 2.06) 4.04
NCDS 0.55 (0.19, 1.58) 2.07
USOC 0.97 (0.48, 1.95) 4.66
ELSA 0.74 (0.22, 2.52) 1.54
ALSPAC(G0) 1.26 (0.86, 1.85) 15.69
1.15 (0.88, 1.51)
Heterogeneity: τ² = 0.00, I² = 0.00%, H² = 1.00
Test of group differences: Qb(1) = 0.40, p = 0.53
1/8 1/4 1/2 1 2 4
Random-effects REML model

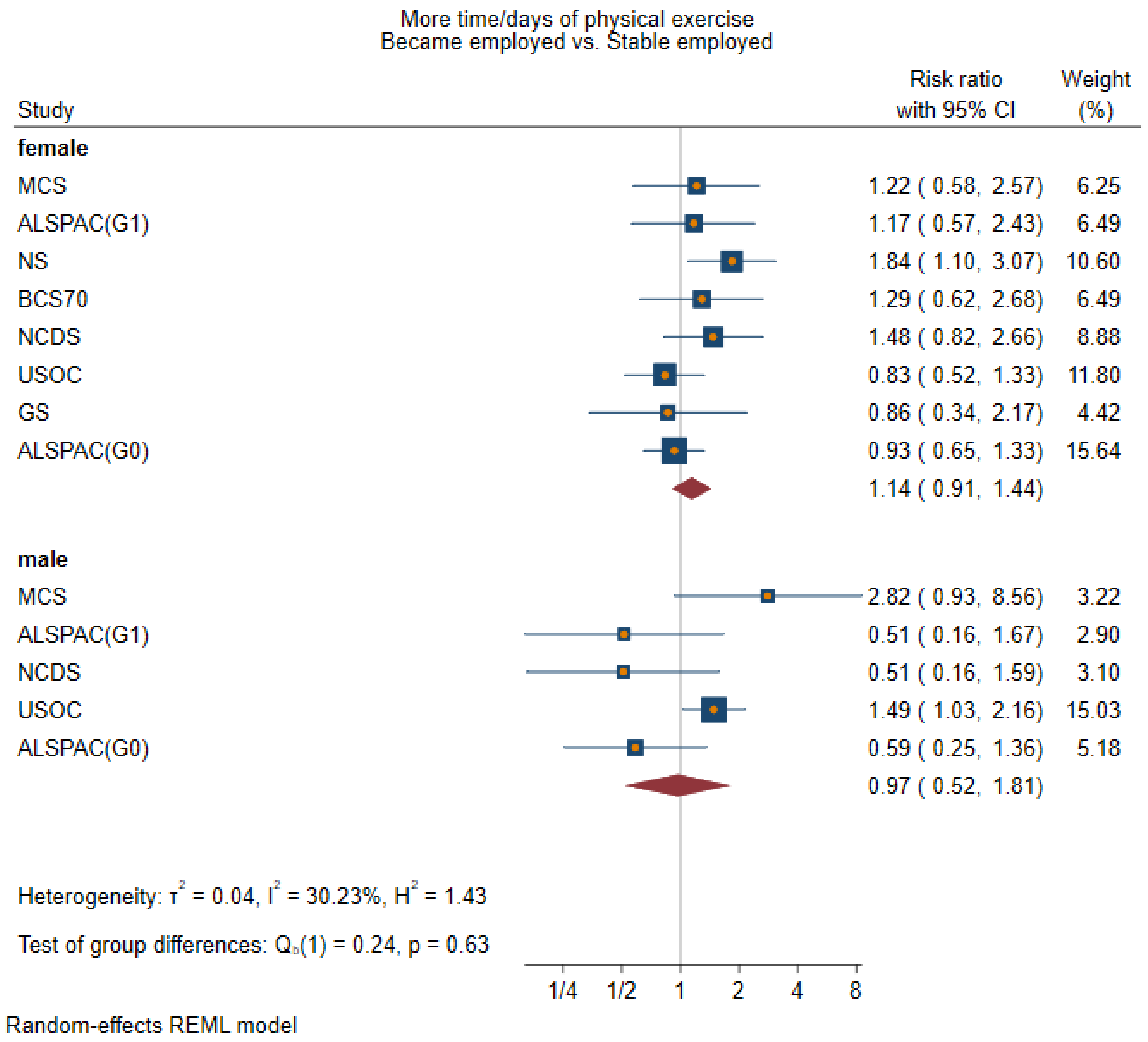
More time/days of physical exercise
Became employed vs. Stable employed
Study
Risk ratio with 95% CI
Weight (%)
female
MCS 1.22 (0.58, 2.57) 6.25
ALSPAC(G1) 1.17 (0.57, 2.43) 6.49
NS 1.84 (1.10, 3.07) 10.60
BCS70 1.29 (0.62, 2.68) 6.49
NCDS 1.48 (0.82, 2.66) 8.88
USOC 0.83 (0.52, 1.33) 11.80
GS 0.86 (0.34, 2.17) 4.42
ALSPAC(G0) 0.93 (0.65, 1.33) 15.64
1.14 (0.91, 1.44)
male
MCS 2.82 (0.93, 8.56) 3.22
ALSPAC(G1) 0.51 (0.16, 1.67) 2.90
NCDS 0.51 (0.16, 1.59) 3.10
USOC 1.49 (1.03, 2.16) 15.03
ALSPAC(G0) 0.59 (0.25, 1.36) 5.18
0.97 (0.52, 1.81)
Heterogeneity: τ² = 0.04, I² = 30.23%, H² = 1.43
Test of group differences: Qb(1) = 0.24, p = 0.63
1/4 1/2 1 2 4 8
Random-effects REML model

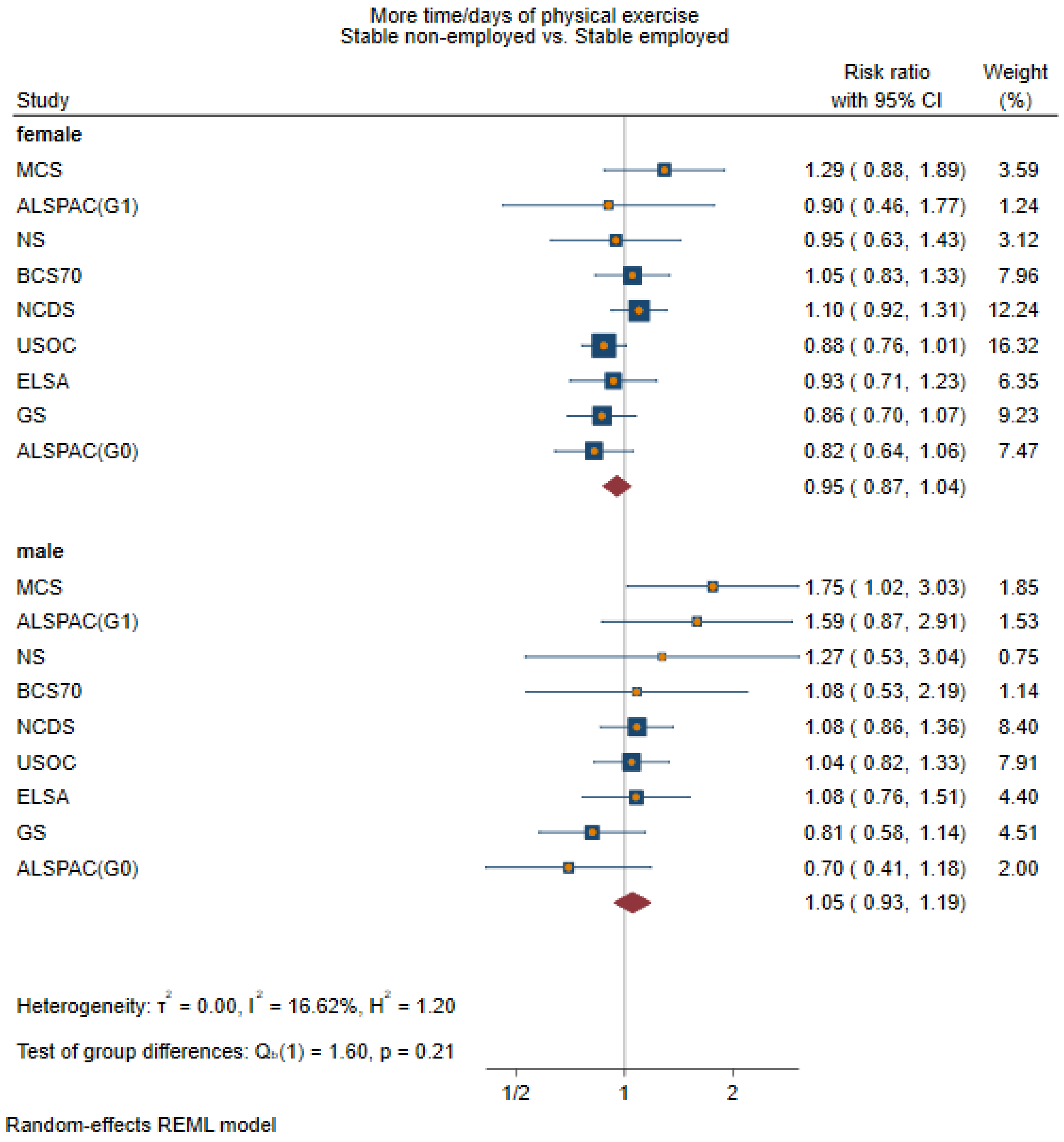

More time/days of physical exercise
Stable non-employed vs. Stable employed
Study
Risk ratio with 95% CI
Weight (%)
female
MCS 1.29 (0.88, 1.89) 3.59
ALSPAC(G1) 0.90 (0.46, 1.77) 1.24
NS 0.95 (0.63, 1.43) 3.12
BCS70 1.05 (0.83, 1.33) 7.96
NCDS 1.10 (0.92, 1.31) 12.24
USOC 0.88 (0.76, 1.01) 16.32
ELSA 0.93 (0.71, 1.23) 6.35
GS 0.86 (0.70, 1.07) 9.23
ALSPAC(G0) 0.82 (0.64, 1.06) 7.47
0.95 (0.87, 1.04)
male
MCS 1.75 (1.02, 3.03) 1.85
ALSPAC(G1) 1.59 (0.87, 2.91) 1.53
NS 1.27 (0.53, 3.04) 0.75
BCS70 1.08 (0.53, 2.19) 1.14
NCDS 1.08 (0.86, 1.36) 8.40
USOC 1.04 (0.82, 1.33) 7.91
ELSA 1.08 (0.76, 1.51) 4.40
GS 0.81 (0.58, 1.14) 4.51
ALSPAC(G0) 0.70 (0.41, 1.18) 2.00
1.05 (0.93, 1.19)
Heterogeneity: τ^2 = 0.00, I^2 = 16.62%, H^2 = 1.20
Test of group differences: $Q_b(1)$ = 1.60, p = 0.21
1/2
1
2
Random-effects REML model

Figure set 29: Sleeps outside ‘normal range’ (i.e. <6 or 9+ hours)

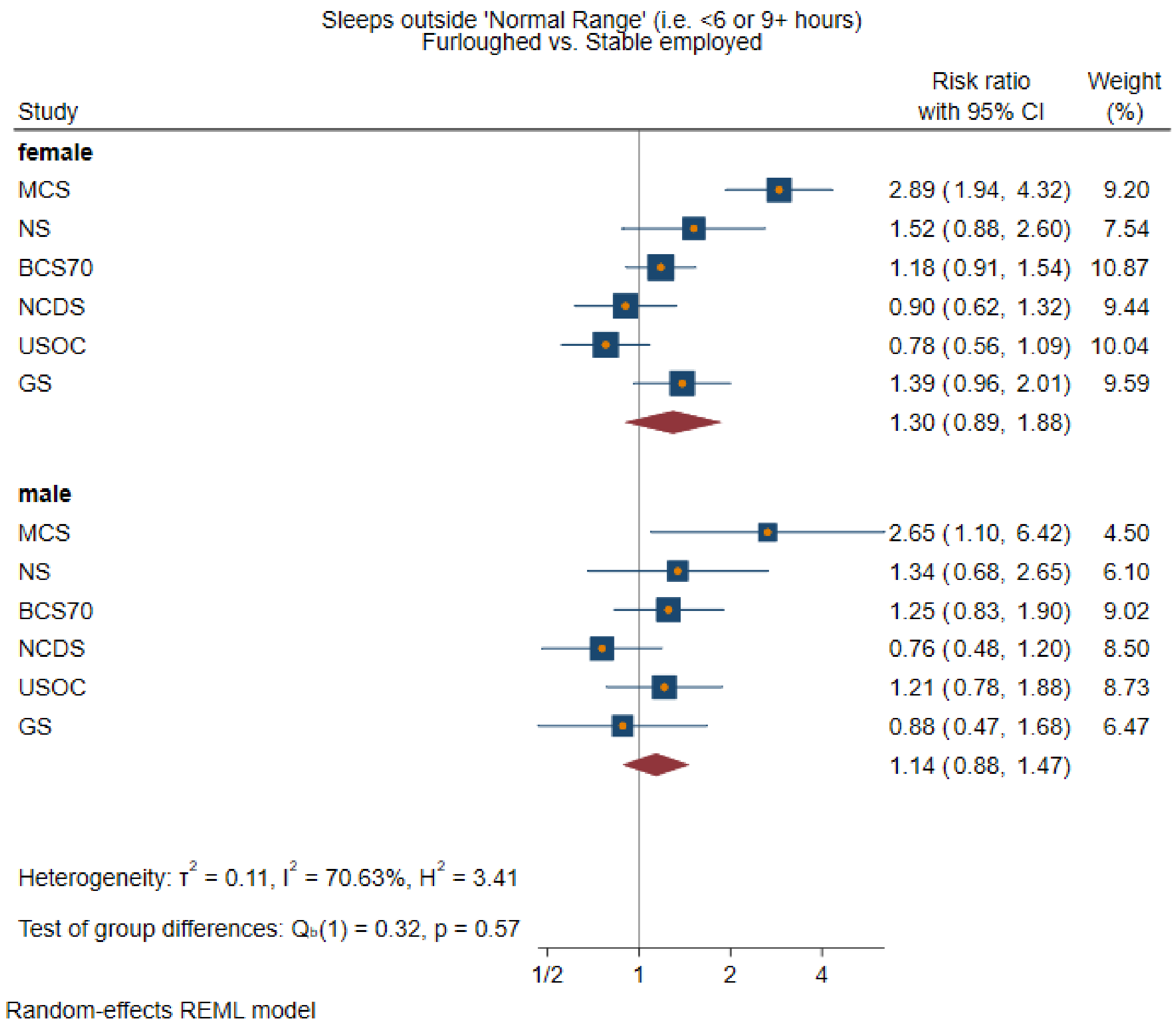

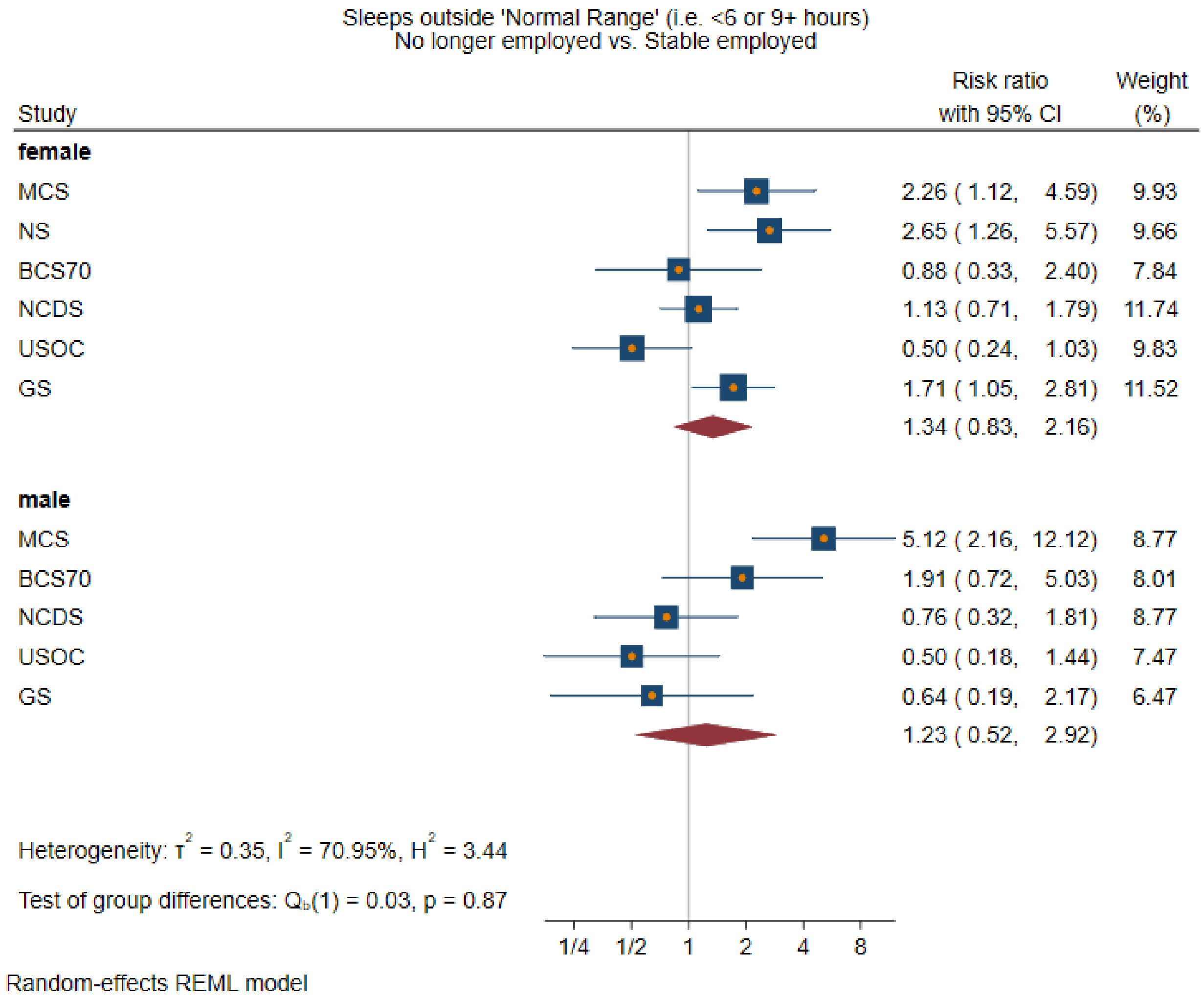

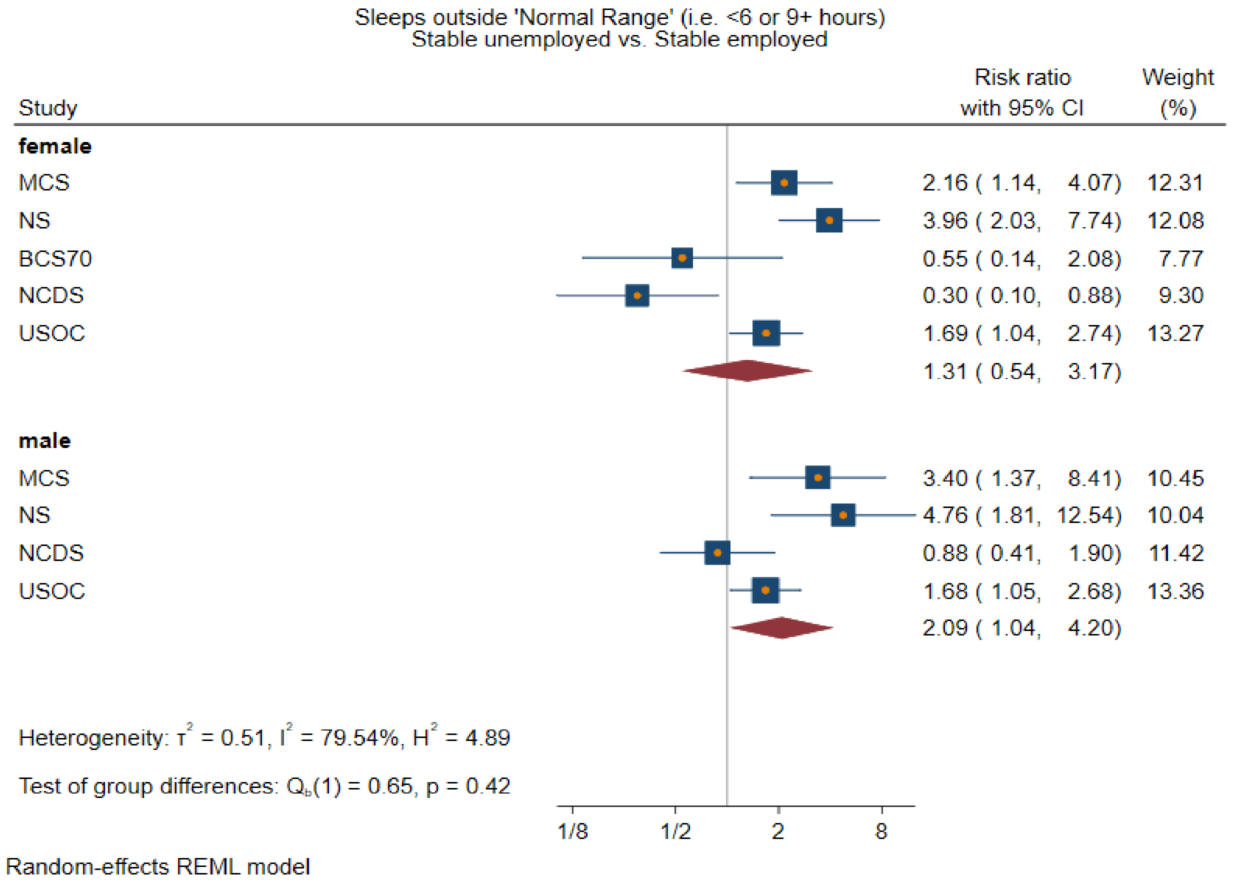
Sleeps outside 'Normal Range' (i.e. <6 or 9+ hours)
Stable unemployed vs. Stable employed
Study
Risk ratio with 95% CI
Weight (%)
female
MCS 2.16 (1.14, 4.07) 12.31
NS 3.96 (2.03, 7.74) 12.08
BCS70 0.55 (0.14, 2.08) 7.77
NCDS 0.30 (0.10, 0.88) 9.30
USOC 1.69 (1.04, 2.74) 13.27
1.31 (0.54, 3.17)
male
MCS 3.40 (1.37, 8.41) 10.45
NS 4.76 (1.81, 12.54) 10.04
NCDS 0.88 (0.41, 1.90) 11.42
USOC 1.68 (1.05, 2.68) 13.36
2.09 (1.04, 4.20)
Heterogeneity: τ² = 0.51, I² = 79.54%, H² = 4.89
Test of group differences: Qb(1) = 0.65, p = 0.42
1/8
1/2
2
8
Random-effects REML model

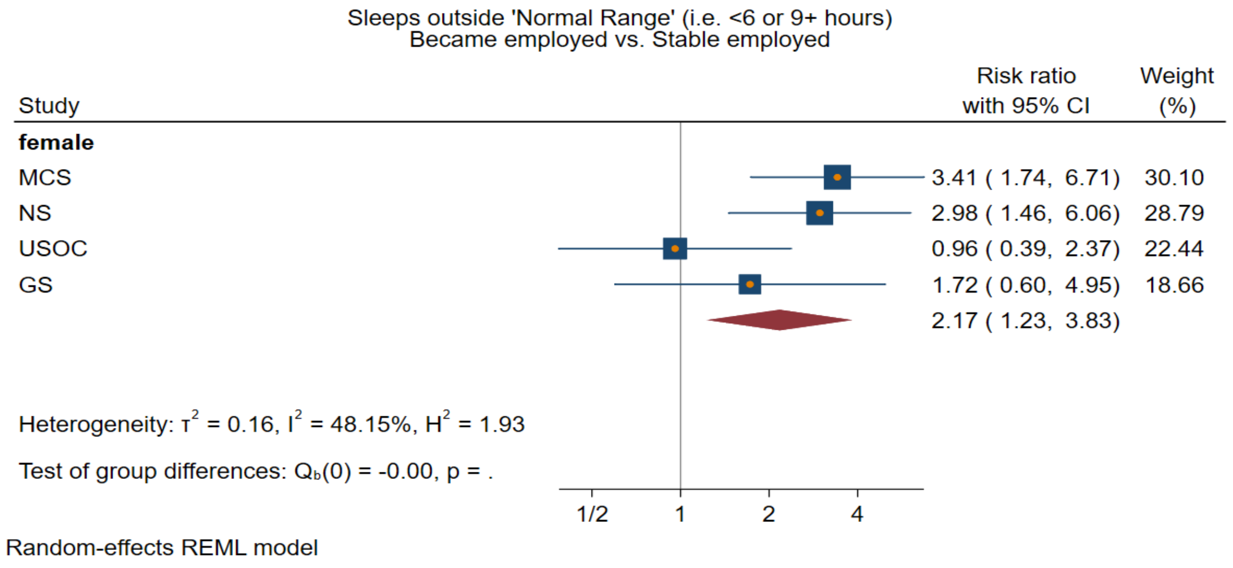
Sleeps outside 'Normal Range' (i.e. <6 or 9+ hours)
Became employed vs. Stable employed
Study
Risk ratio with 95% CI
Weight (%)
female
MCS 3.41 (1.74, 6.71) 30.10
NS 2.98 (1.46, 6.06) 28.79
USOC 0.96 (0.39, 2.37) 22.44
GS 1.72 (0.60, 4.95) 18.66
2.17 (1.23, 3.83)
Heterogeneity: τ² = 0.16, I² = 48.15%, H² = 1.93
Test of group differences: Qb(0) = -0.00, p = .
1/2
1
2
4
Random-effects REML model

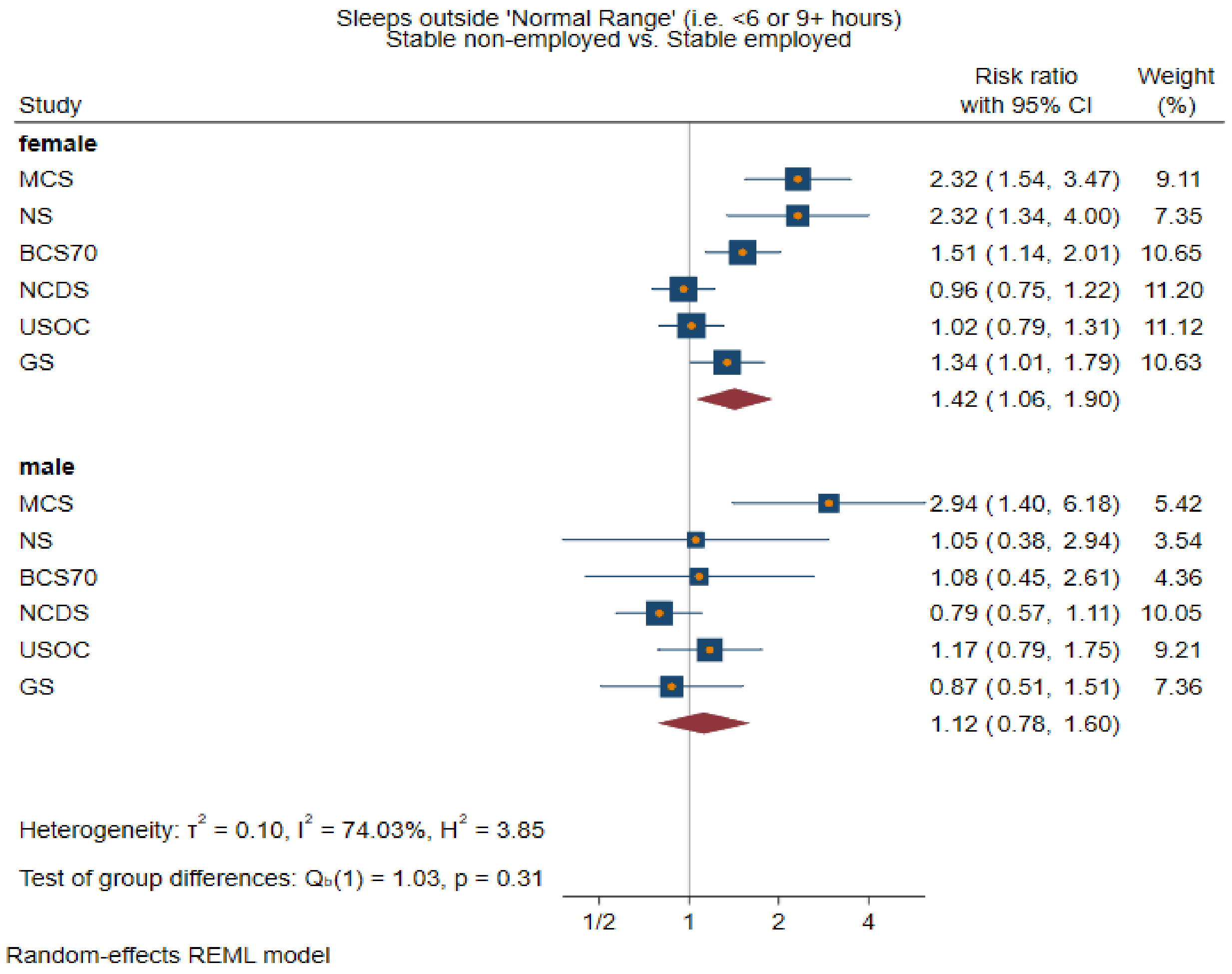
Sleeps outside 'Normal Range' (i.e. <6 or 9+ hours)
Stable non-employed vs. Stable employed
Study
Risk ratio with 95% CI
Weight (%)
female
MCS 2.32 (1.54, 3.47) 9.11
NS 2.32 (1.34, 4.00) 7.35
BCS70 1.51 (1.14, 2.01) 10.65
NCDS 0.96 (0.75, 1.22) 11.20
USOC 1.02 (0.79, 1.31) 11.12
GS 1.34 (1.01, 1.79) 10.63
1.42 (1.06, 1.90)
male
MCS 2.94 (1.40, 6.18) 5.42
NS 1.05 (0.38, 2.94) 3.54
BCS70 1.08 (0.45, 2.61) 4.36
NCDS 0.79 (0.57, 1.11) 10.05
USOC 1.17 (0.79, 1.75) 9.21
GS 0.87 (0.51, 1.51) 7.36
1.12 (0.78, 1.60)
Heterogeneity: τ² = 0.10, I² = 74.03%, H² = 3.85
Test of group differences: Qb(1) = 1.03, p = 0.31
1/2
1
2
4
Random-effects REML model

Figure set 30: Sleeps less than before

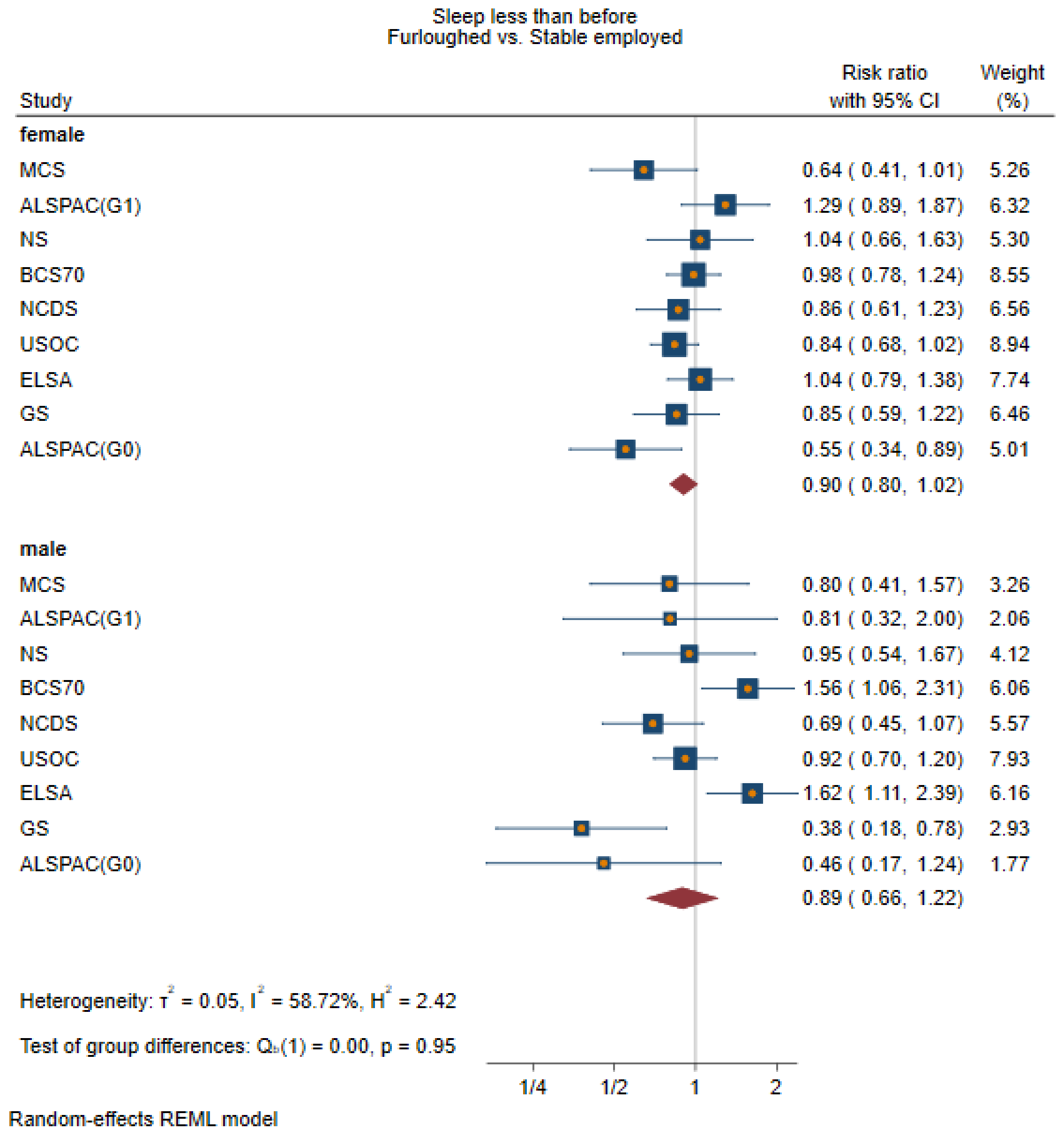

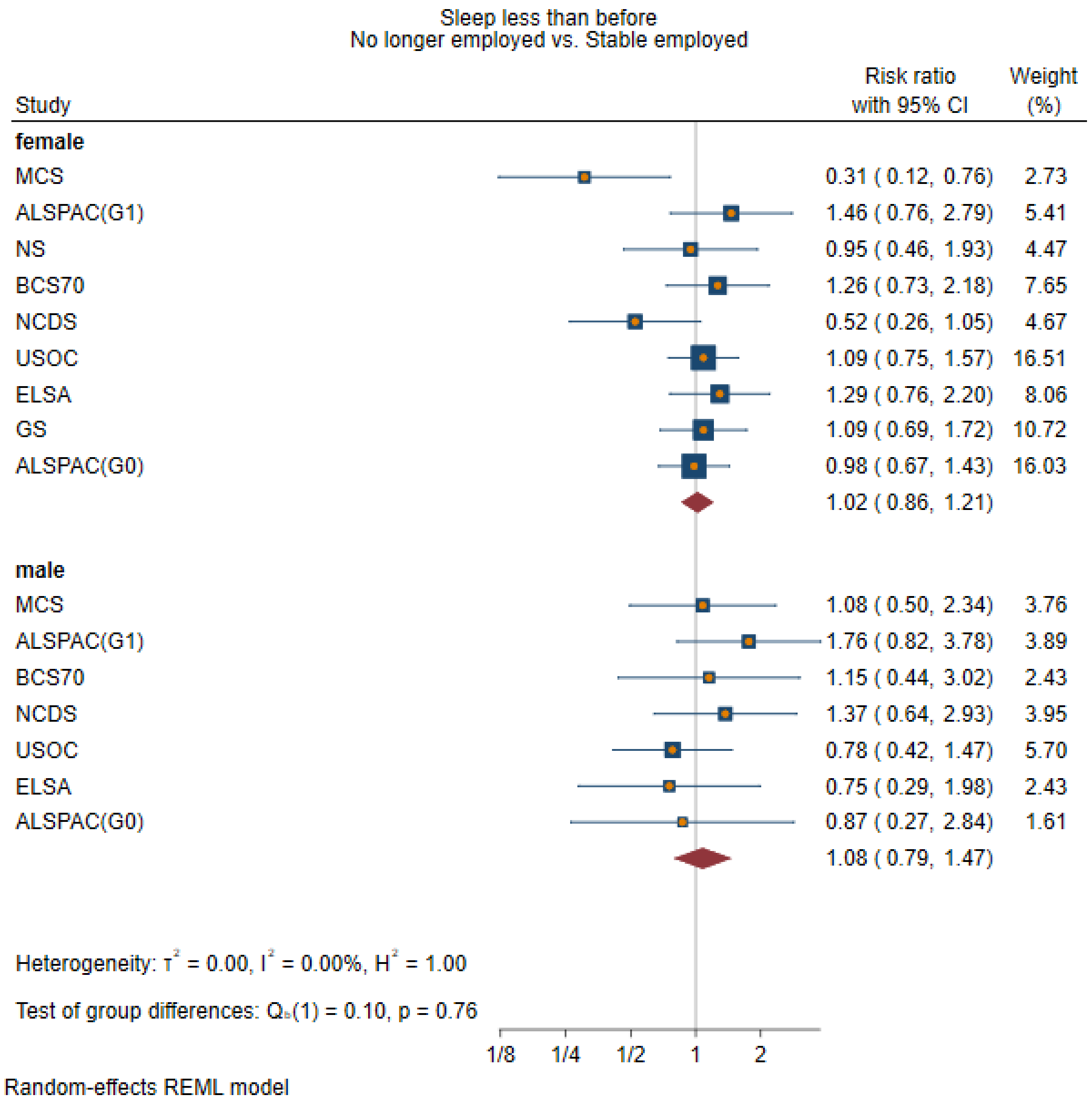

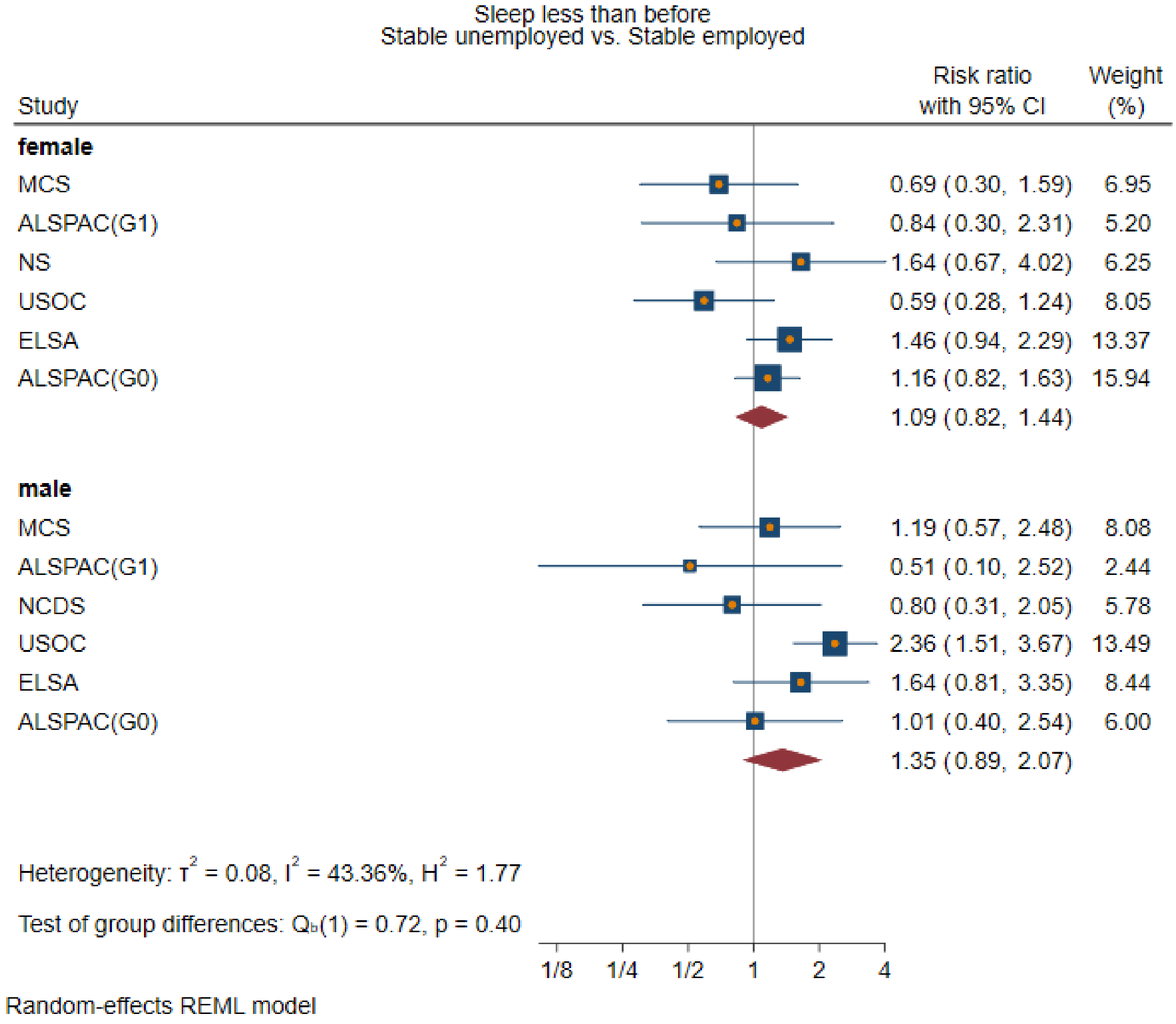
Sleep less than before
Stable unemployed vs. Stable employed
Study
Risk ratio with 95% CI
Weight (%)
female
MCS 0.69 (0.30, 1.59) 6.95
ALSPAC(G1) 0.84 (0.30, 2.31) 5.20
NS 1.64 (0.67, 4.02) 6.25
USOC 0.59 (0.28, 1.24) 8.05
ELSA 1.46 (0.94, 2.29) 13.37
ALSPAC(G0) 1.16 (0.82, 1.63) 15.94
1.09 (0.82, 1.44)
male
MCS 1.19 (0.57, 2.48) 8.08
ALSPAC(G1) 0.51 (0.10, 2.52) 2.44
NCDS 0.80 (0.31, 2.05) 5.78
USOC 2.36 (1.51, 3.67) 13.49
ELSA 1.64 (0.81, 3.35) 8.44
ALSPAC(G0) 1.01 (0.40, 2.54) 6.00
1.35 (0.89, 2.07)
Heterogeneity: τ² = 0.08, I² = 43.36%, H² = 1.77
Test of group differences: Qb(1) = 0.72, p = 0.40
1/8 1/4 1/2 1 2 4
Random-effects REML model

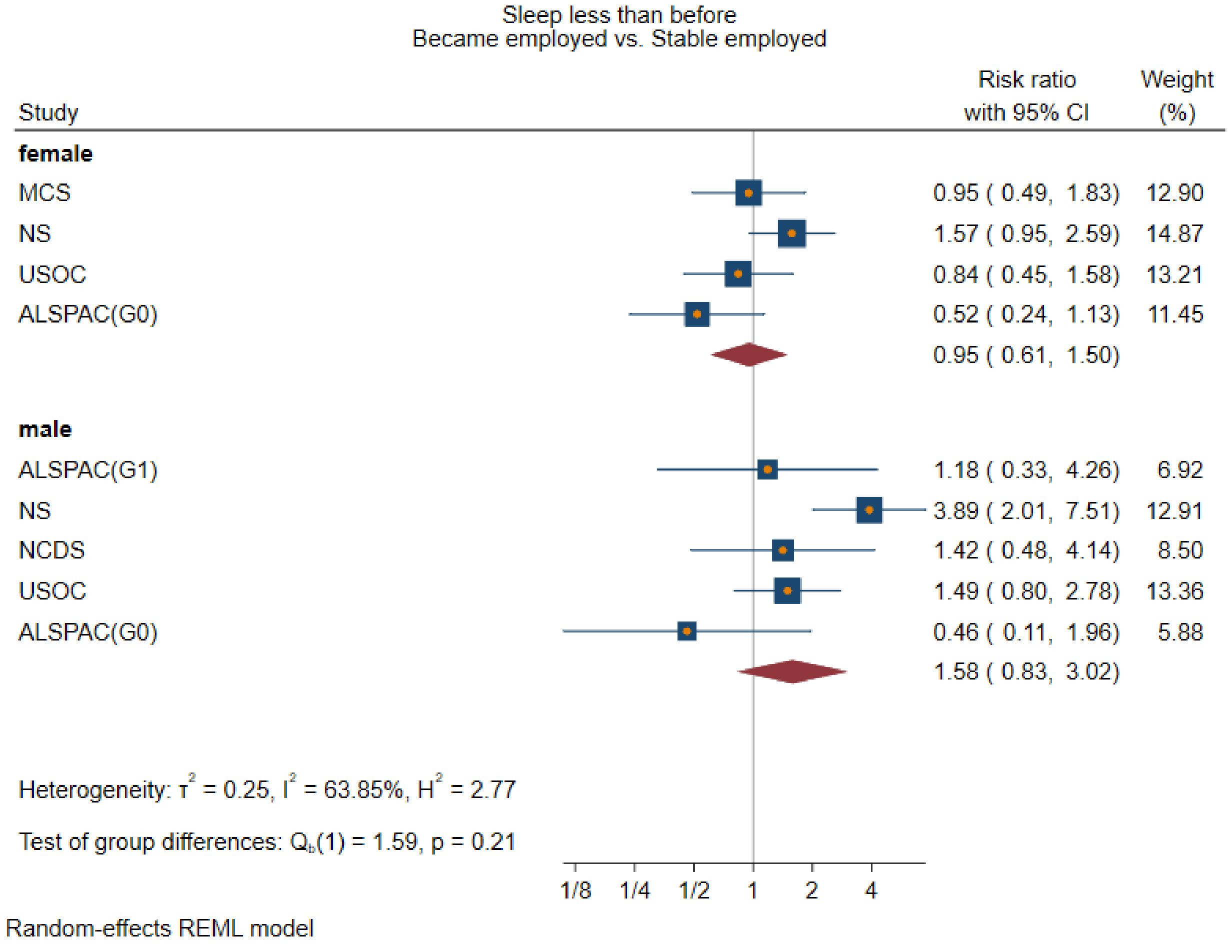
Sleep less than before
Became employed vs. Stable employed
Study
Risk ratio with 95% CI
Weight (%)
female
MCS 0.95 (0.49, 1.83) 12.90
NS 1.57 (0.95, 2.59) 14.87
USOC 0.84 (0.45, 1.58) 13.21
ALSPAC(G0) 0.52 (0.24, 1.13) 11.45
0.95 (0.61, 1.50)
male
ALSPAC(G1) 1.18 (0.33, 4.26) 6.92
NS 3.89 (2.01, 7.51) 12.91
NCDS 1.42 (0.48, 4.14) 8.50
USOC 1.49 (0.80, 2.78) 13.36
ALSPAC(G0) 0.46 (0.11, 1.96) 5.88
1.58 (0.83, 3.02)
Heterogeneity: τ² = 0.25, I² = 63.85%, H² = 2.77
Test of group differences: Qb(1) = 1.59, p = 0.21
1/8 1/4 1/2 1 2 4
Random-effects REML model

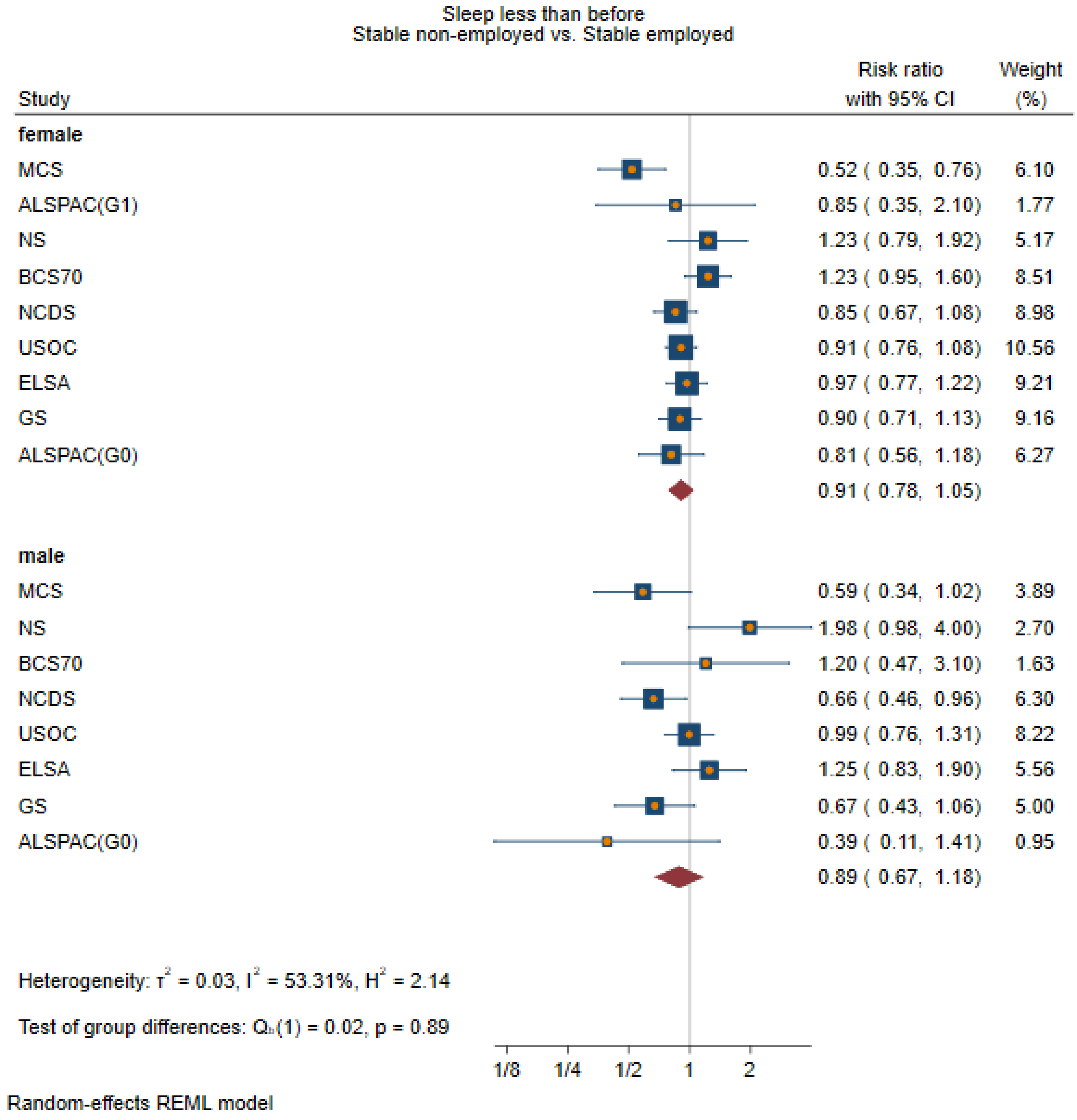
Sleep less than before
Stable non-employed vs. Stable employed
Study
Risk ratio with 95% CI
Weight (%)
female
MCS 0.52 (0.35, 0.76) 6.10
ALSPAC(G1) 0.85 (0.35, 2.10) 1.77
NS 1.23 (0.79, 1.92) 5.17
BCS70 1.23 (0.95, 1.60) 8.51
NCDS 0.85 (0.67, 1.08) 8.98
USOC 0.91 (0.76, 1.08) 10.56
ELSA 0.97 (0.77, 1.22) 9.21
GS 0.90 (0.71, 1.13) 9.16
ALSPAC(G0) 0.81 (0.56, 1.18) 6.27
0.91 (0.78, 1.05)
male
MCS 0.59 (0.34, 1.02) 3.89
NS 1.98 (0.98, 4.00) 2.70
BCS70 1.20 (0.47, 3.10) 1.63
NCDS 0.66 (0.46, 0.96) 6.30
USOC 0.99 (0.76, 1.31) 8.22
ELSA 1.25 (0.83, 1.90) 5.56
GS 0.67 (0.43, 1.06) 5.00
ALSPAC(G0) 0.39 (0.11, 1.41) 0.95
0.89 (0.67, 1.18)
Heterogeneity: $\tau^2 = 0.03$, $I^2 = 53.31\%$, $H^2 = 2.14$
Test of group differences: $Q_b(1) = 0.02$, $p = 0.89$
1/8
1/4
1/2
1
2
Random-effects REML model

Figure set 31: Sleeps more than before

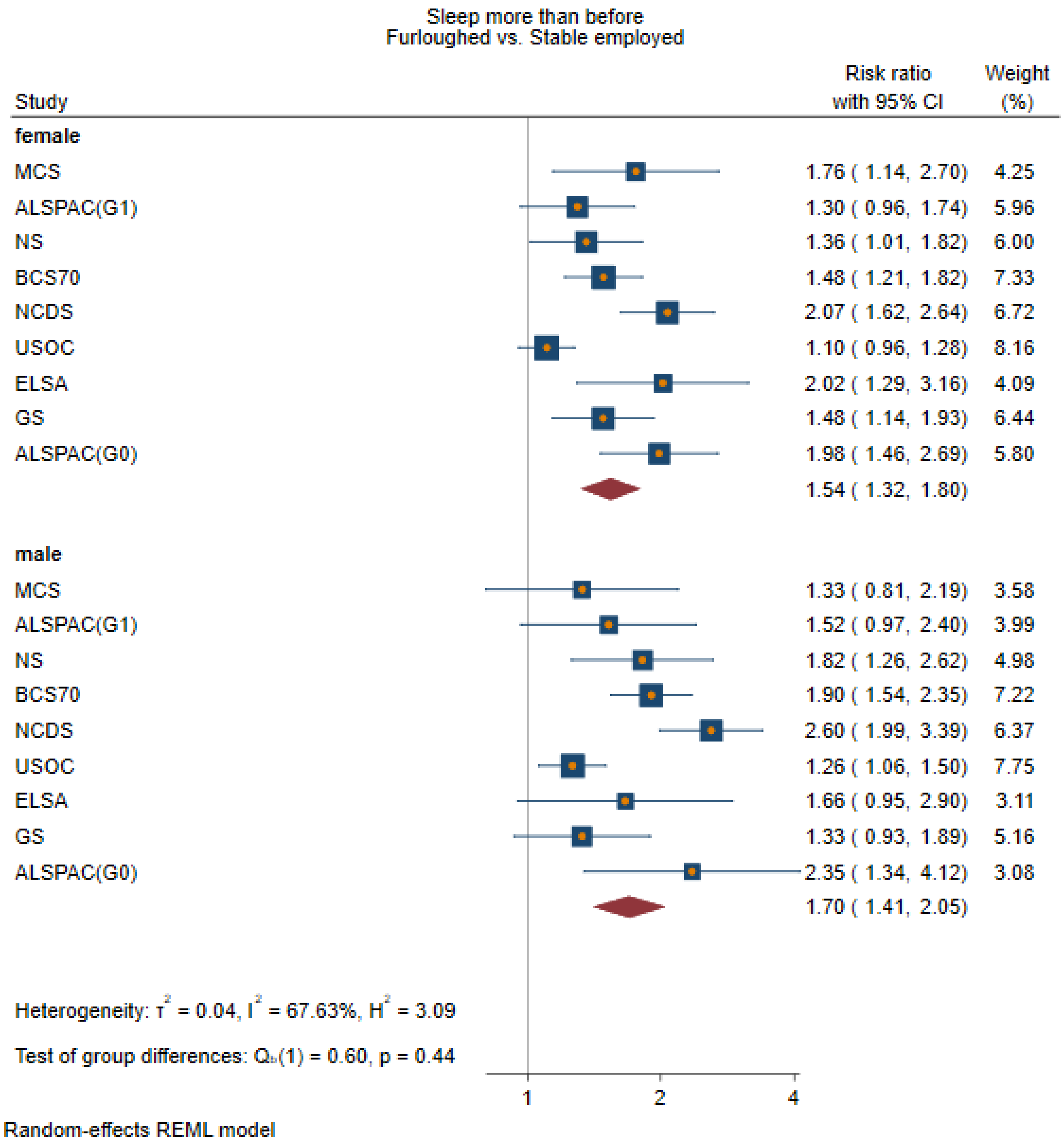

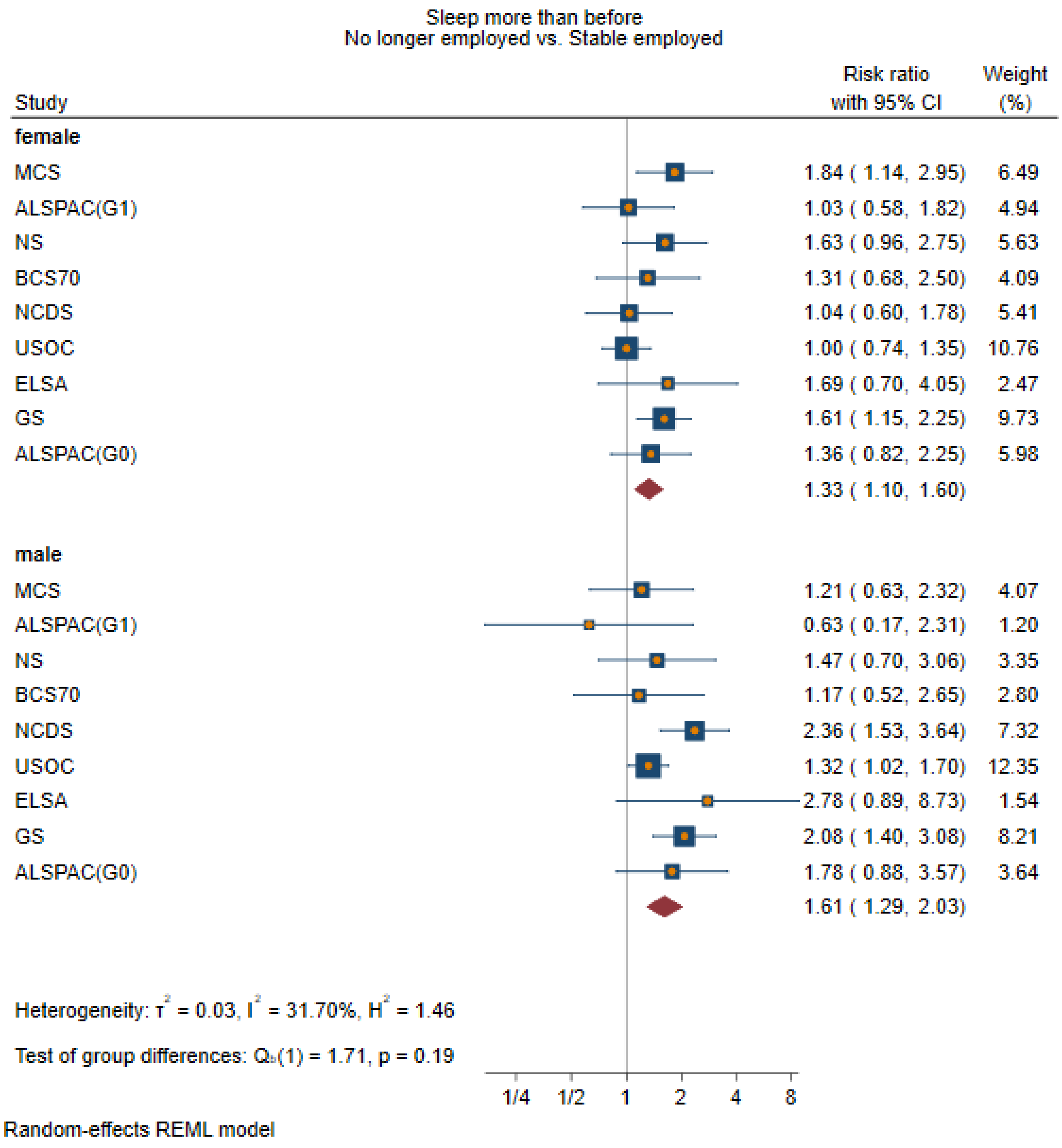

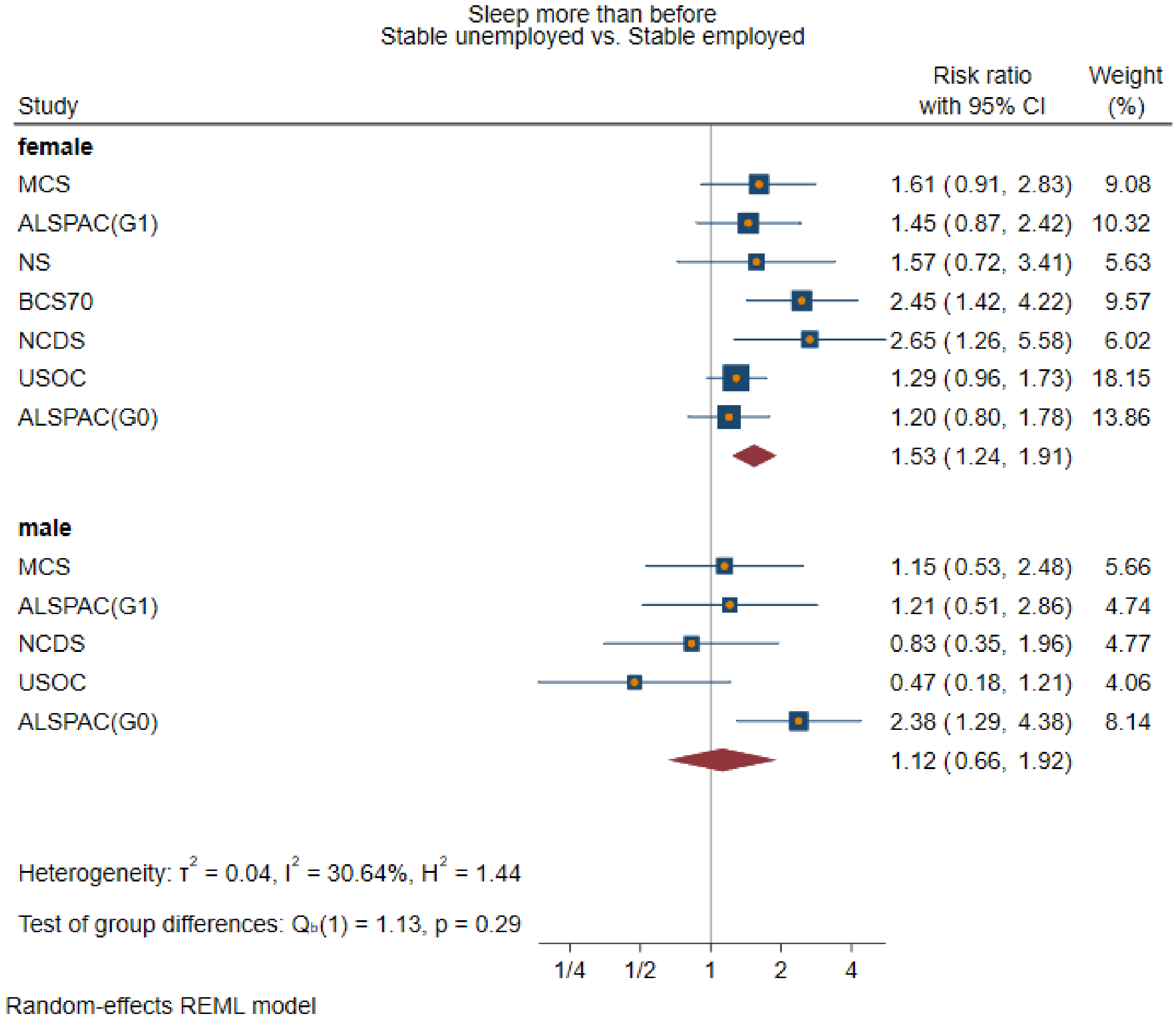
Sleep more than before
Stable unemployed vs. Stable employed
Study
Risk ratio with 95% CI
Weight (%)
female
MCS 1.61 (0.91, 2.83) 9.08
ALSPAC(G1) 1.45 (0.87, 2.42) 10.32
NS 1.57 (0.72, 3.41) 5.63
BCS70 2.45 (1.42, 4.22) 9.57
NCDS 2.65 (1.26, 5.58) 6.02
USOC 1.29 (0.96, 1.73) 18.15
ALSPAC(G0) 1.20 (0.80, 1.78) 13.86
1.53 (1.24, 1.91)
male
MCS 1.15 (0.53, 2.48) 5.66
ALSPAC(G1) 1.21 (0.51, 2.86) 4.74
NCDS 0.83 (0.35, 1.96) 4.77
USOC 0.47 (0.18, 1.21) 4.06
ALSPAC(G0) 2.38 (1.29, 4.38) 8.14
1.12 (0.66, 1.92)
Heterogeneity: τ² = 0.04, I² = 30.64%, H² = 1.44
Test of group differences: Q_b(1) = 1.13, p = 0.29
1/4 1/2 1 2 4
Random-effects REML model

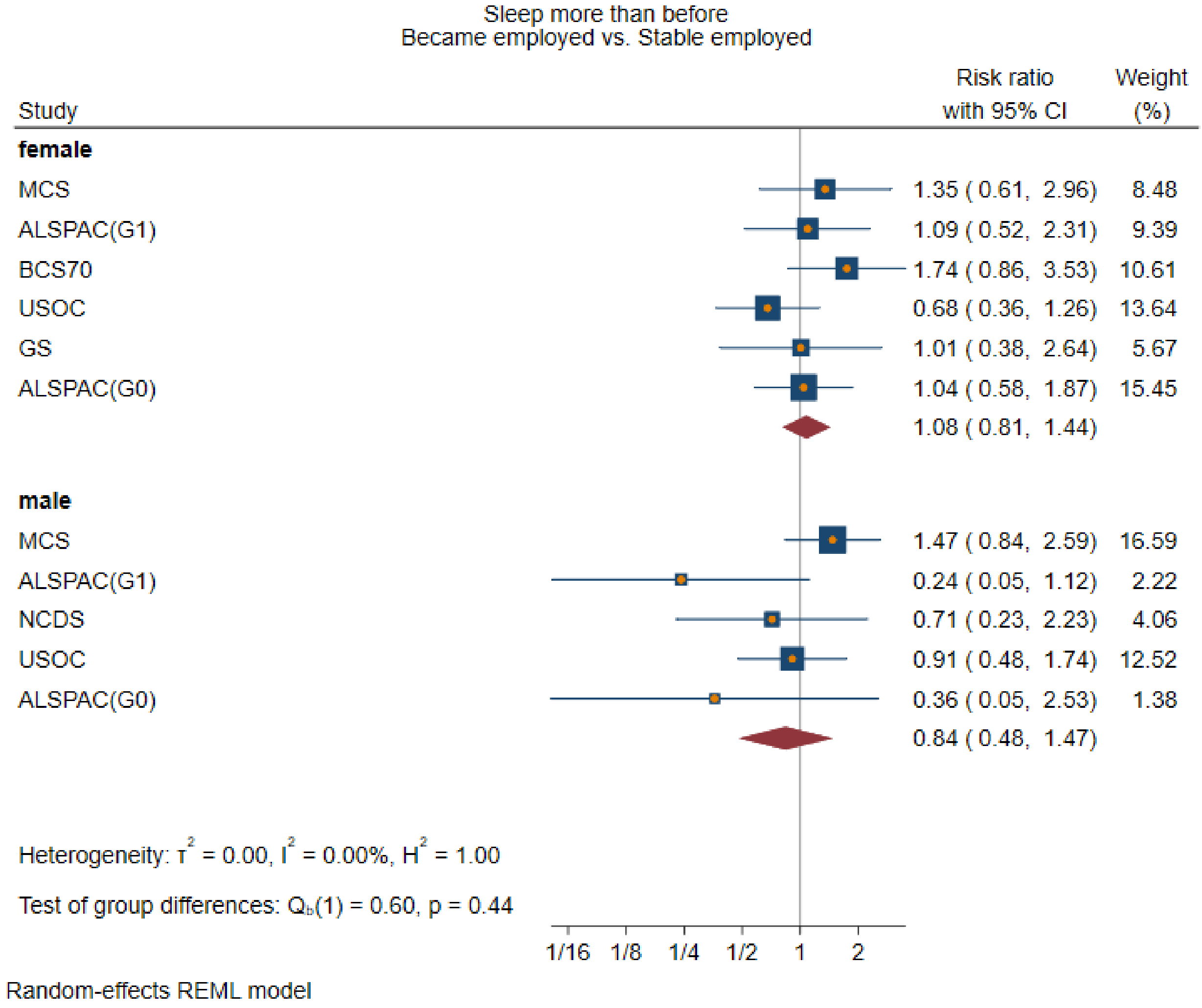
Sleep more than before
Became employed vs. Stable employed
Study
Risk ratio with 95% CI
Weight (%)
female
MCS 1.35 (0.61, 2.96) 8.48
ALSPAC(G1) 1.09 (0.52, 2.31) 9.39
BCS70 1.74 (0.86, 3.53) 10.61
USOC 0.68 (0.36, 1.26) 13.64
GS 1.01 (0.38, 2.64) 5.67
ALSPAC(G0) 1.04 (0.58, 1.87) 15.45
1.08 (0.81, 1.44)
male
MCS 1.47 (0.84, 2.59) 16.59
ALSPAC(G1) 0.24 (0.05, 1.12) 2.22
NCDS 0.71 (0.23, 2.23) 4.06
USOC 0.91 (0.48, 1.74) 12.52
ALSPAC(G0) 0.36 (0.05, 2.53) 1.38
0.84 (0.48, 1.47)
Heterogeneity: τ² = 0.00, I² = 0.00%, H² = 1.00
Test of group differences: Q_b(1) = 0.60, p = 0.44
1/16 1/8 1/4 1/2 1 2
Random-effects REML model

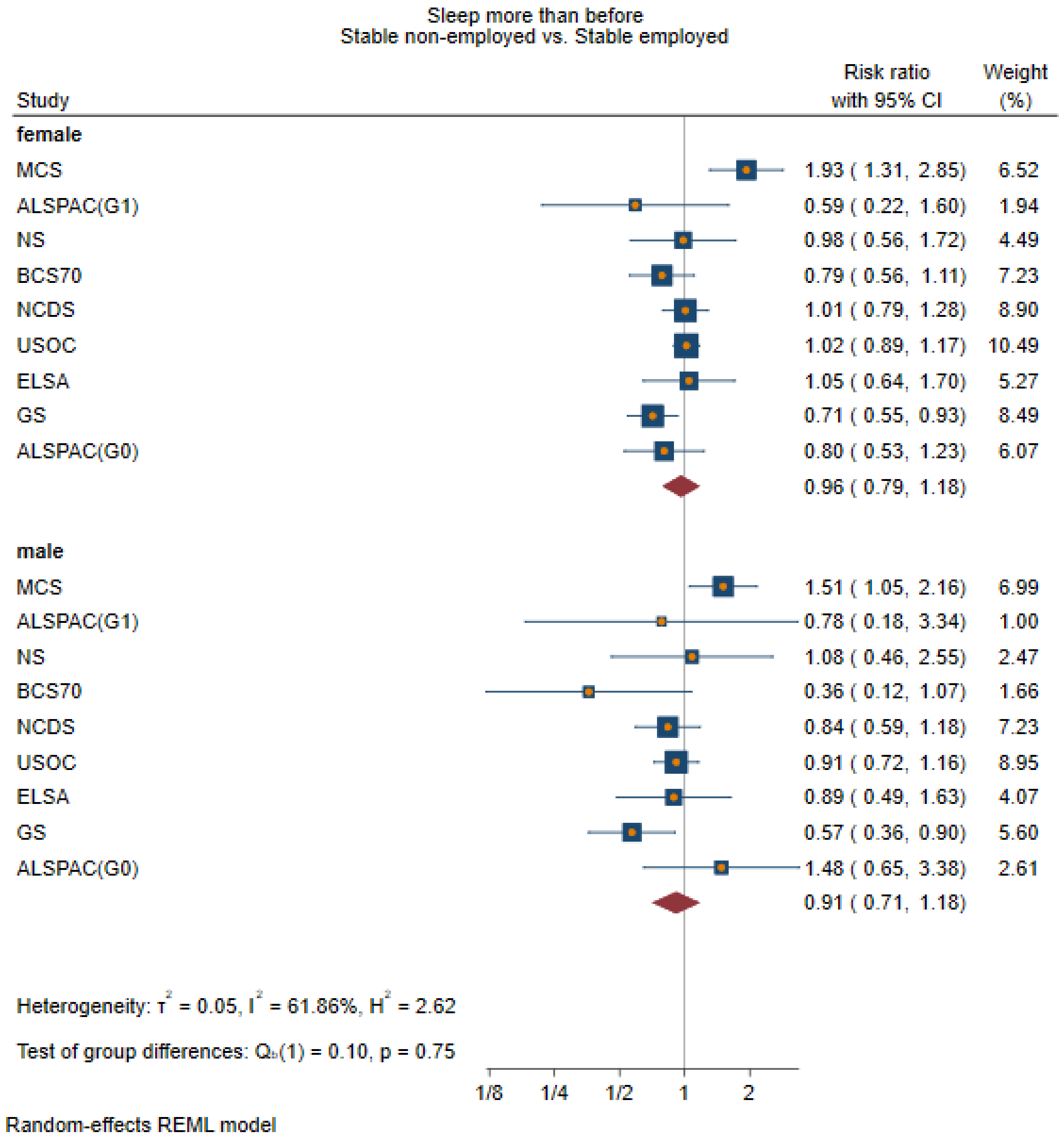
Sleep more than before
Stable non-employed vs. Stable employed
Study
Risk ratio with 95% CI
Weight (%)
female
MCS 1.93 (1.31, 2.85) 6.52
ALSPAC(G1) 0.59 (0.22, 1.60) 1.94
NS 0.98 (0.56, 1.72) 4.49
BCS70 0.79 (0.56, 1.11) 7.23
NCDS 1.01 (0.79, 1.28) 8.90
USOC 1.02 (0.89, 1.17) 10.49
ELSA 1.05 (0.64, 1.70) 5.27
GS 0.71 (0.55, 0.93) 8.49
ALSPAC(G0) 0.80 (0.53, 1.23) 6.07
0.96 (0.79, 1.18)
male
MCS 1.51 (1.05, 2.16) 6.99
ALSPAC(G1) 0.78 (0.18, 3.34) 1.00
NS 1.08 (0.46, 2.55) 2.47
BCS70 0.36 (0.12, 1.07) 1.66
NCDS 0.84 (0.59, 1.18) 7.23
USOC 0.91 (0.72, 1.16) 8.95
ELSA 0.89 (0.49, 1.63) 4.07
GS 0.57 (0.36, 0.90) 5.60
ALSPAC(G0) 1.48 (0.65, 3.38) 2.61
0.91 (0.71, 1.18)
Heterogeneity: τ^2 = 0.05, I^2 = 61.86%, H^2 = 2.62
Test of group differences: $Q_b(1)$ = 0.10, p = 0.75
1/8
1/4
1/2
1
2
Random-effects REML model

Figure set 32: From 6/9h a night to outside 'normal range'

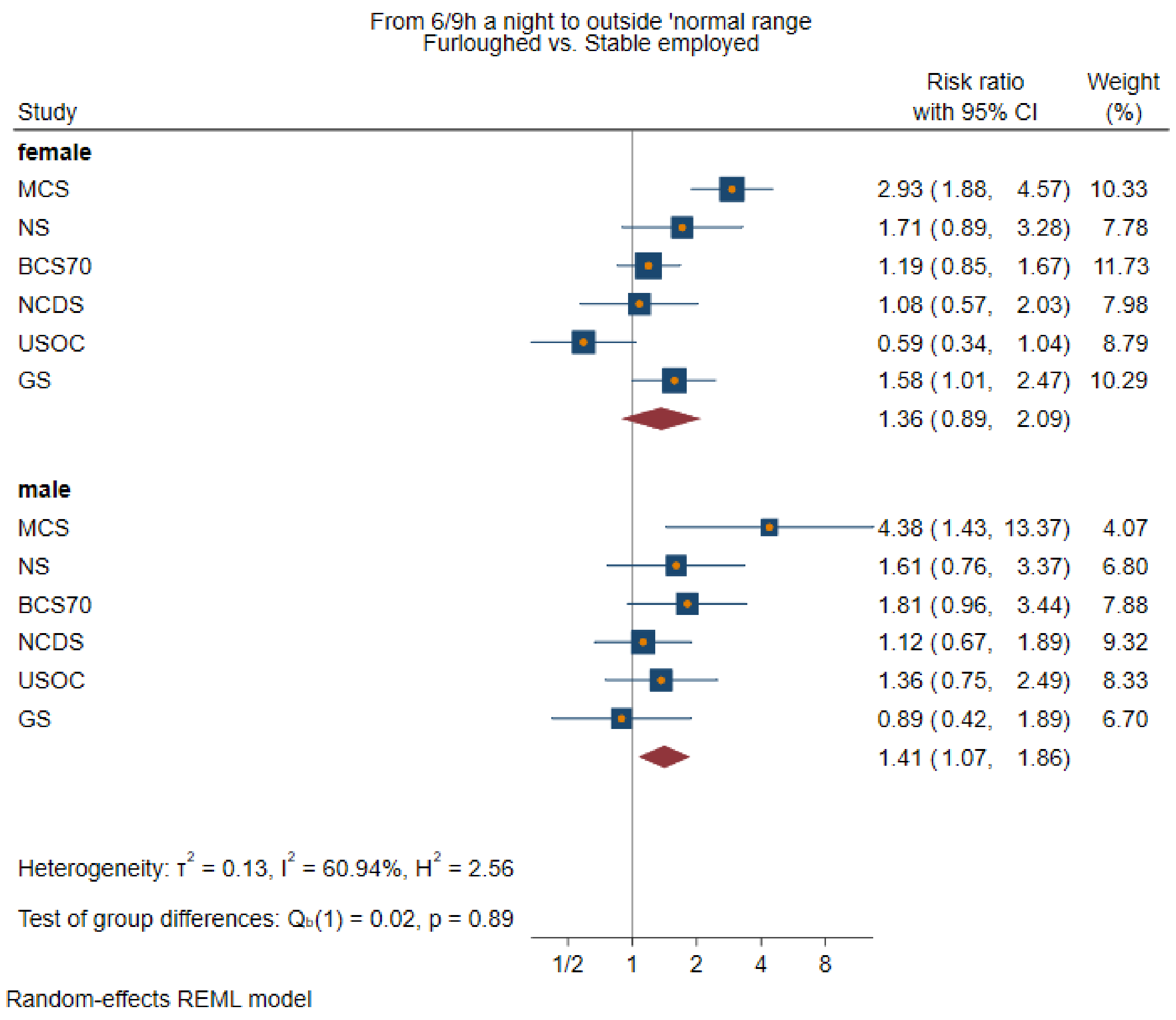

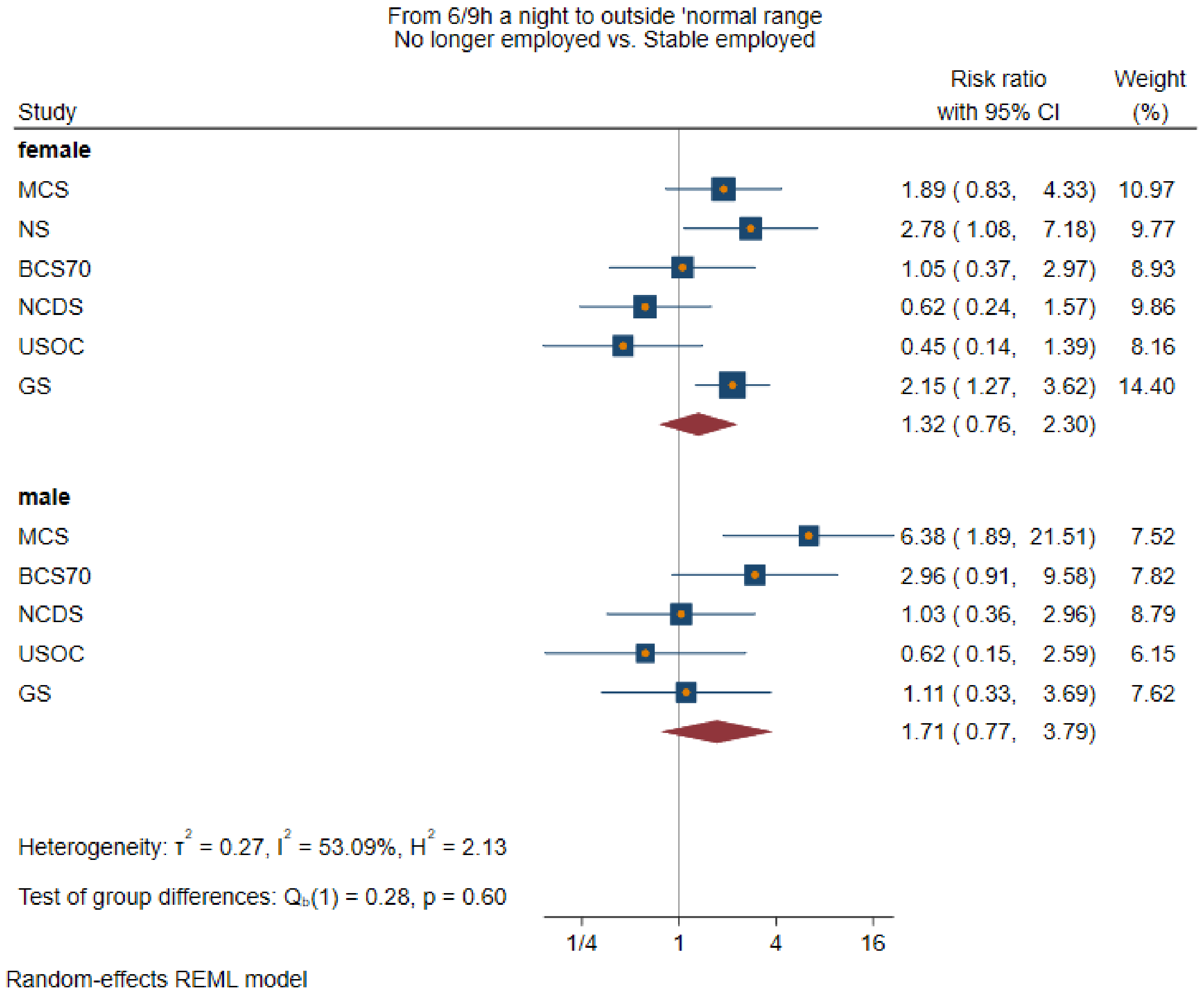

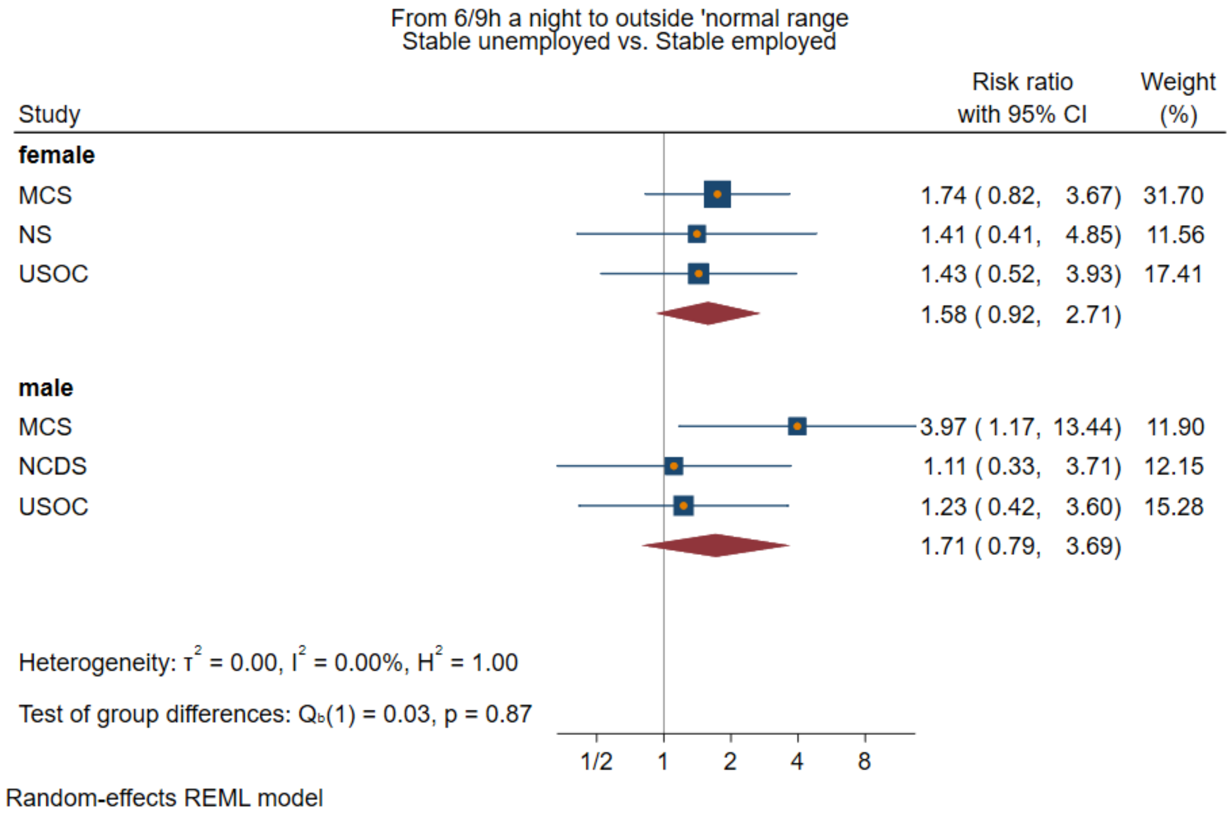
From 6/9h a night to outside 'normal range
Stable unemployed vs. Stable employed
Study
Risk ratio with 95% CI
Weight (%)
female
MCS 1.74 (0.82, 3.67) 31.70
NS 1.41 (0.41, 4.85) 11.56
USOC 1.43 (0.52, 3.93) 17.41
1.58 (0.92, 2.71)
male
MCS 3.97 (1.17, 13.44) 11.90
NCDS 1.11 (0.33, 3.71) 12.15
USOC 1.23 (0.42, 3.60) 15.28
1.71 (0.79, 3.69)
Heterogeneity: τ² = 0.00, I² = 0.00%, H² = 1.00
Test of group differences: Qb(1) = 0.03, p = 0.87
1/2
1
2
4
8
Random-effects REML model

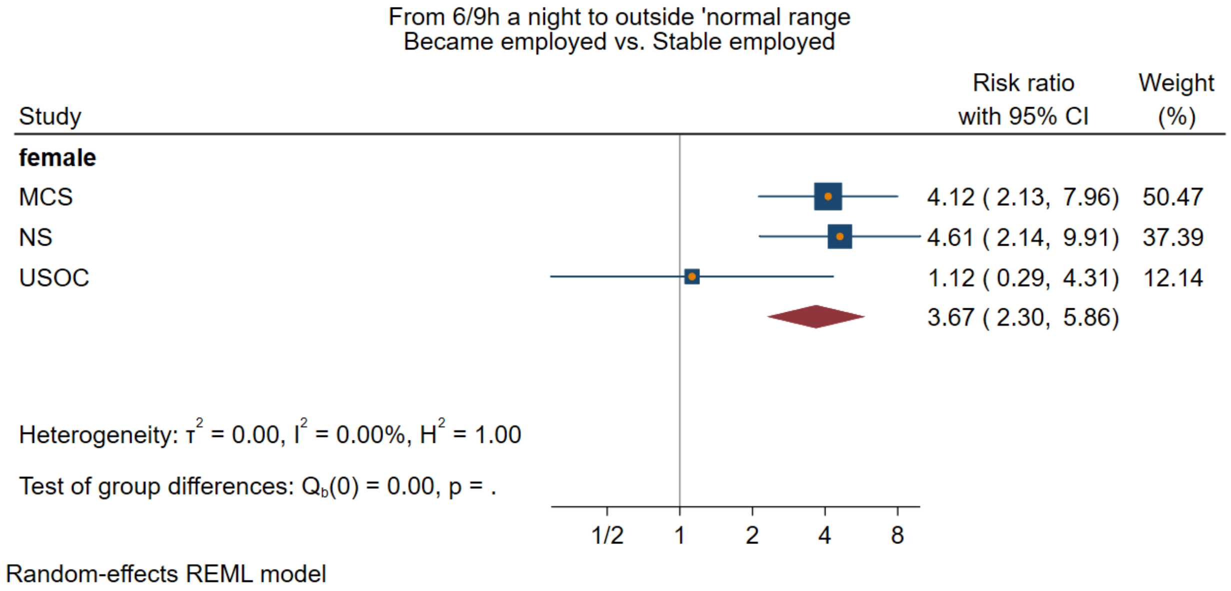
From 6/9h a night to outside 'normal range
Became employed vs. Stable employed
Study
Risk ratio with 95% CI
Weight (%)
female
MCS 4.12 (2.13, 7.96) 50.47
NS 4.61 (2.14, 9.91) 37.39
USOC 1.12 (0.29, 4.31) 12.14
3.67 (2.30, 5.86)
Heterogeneity: τ² = 0.00, I² = 0.00%, H² = 1.00
Test of group differences: Qb(0) = 0.00, p = .
1/2
1
2
4
8
Random-effects REML model

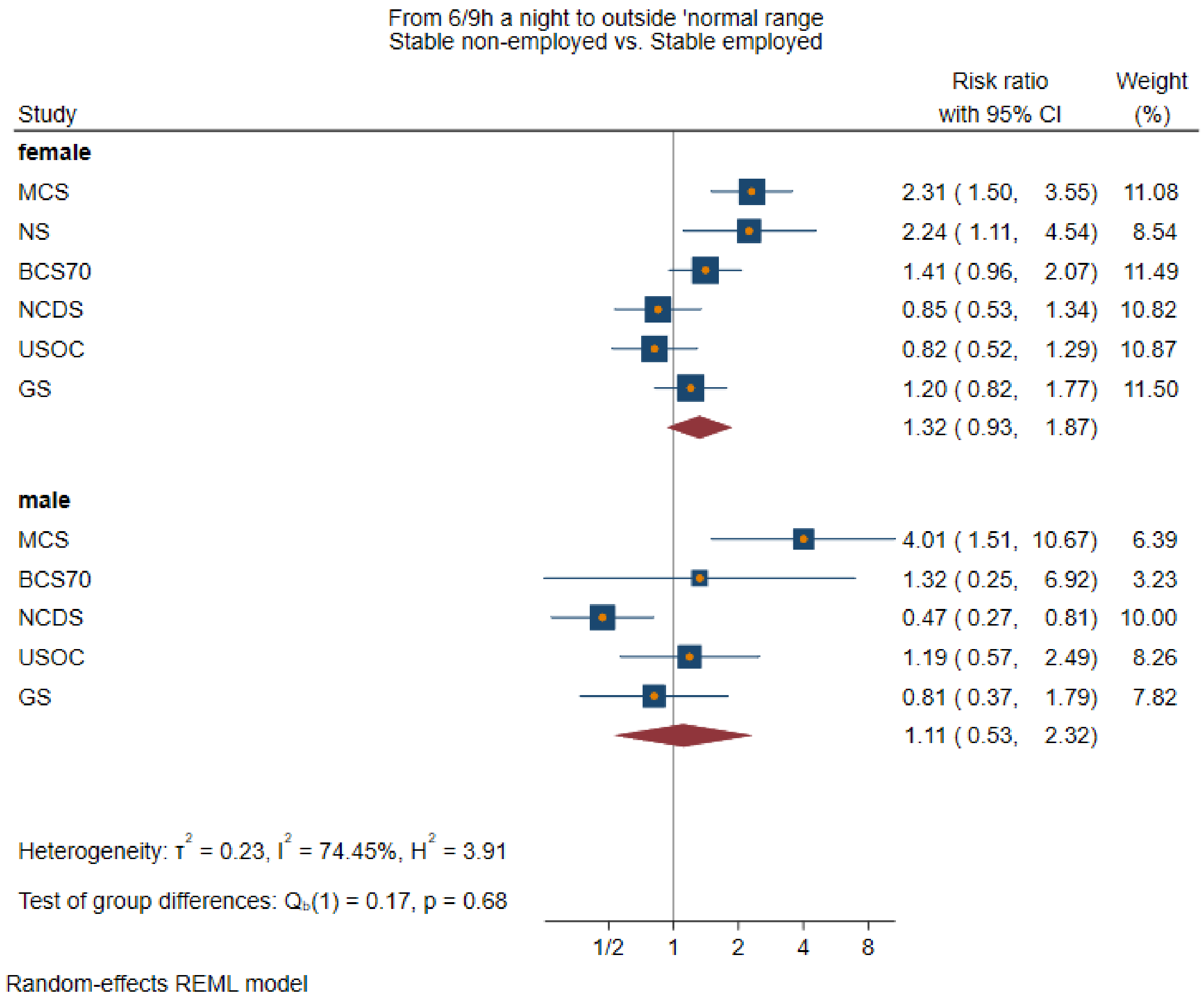
From 6/9h a night to outside 'normal range
Stable non-employed vs. Stable employed
Study
Risk ratio with 95% CI
Weight (%)
female
MCS 2.31 (1.50, 3.55) 11.08
NS 2.24 (1.11, 4.54) 8.54
BCS70 1.41 (0.96, 2.07) 11.49
NCDS 0.85 (0.53, 1.34) 10.82
USOC 0.82 (0.52, 1.29) 10.87
GS 1.20 (0.82, 1.77) 11.50
1.32 (0.93, 1.87)
male
MCS 4.01 (1.51, 10.67) 6.39
BCS70 1.32 (0.25, 6.92) 3.23
NCDS 0.47 (0.27, 0.81) 10.00
USOC 1.19 (0.57, 2.49) 8.26
GS 0.81 (0.37, 1.79) 7.82
1.11 (0.53, 2.32)
Heterogeneity: τ² = 0.23, I² = 74.45%, H² = 3.91
Test of group differences: Qb(1) = 0.17, p = 0.68
1/2
1
2
4
8
Random-effects REML model

Figure set 33: From outside ‘normal range’ to 6/9h a night

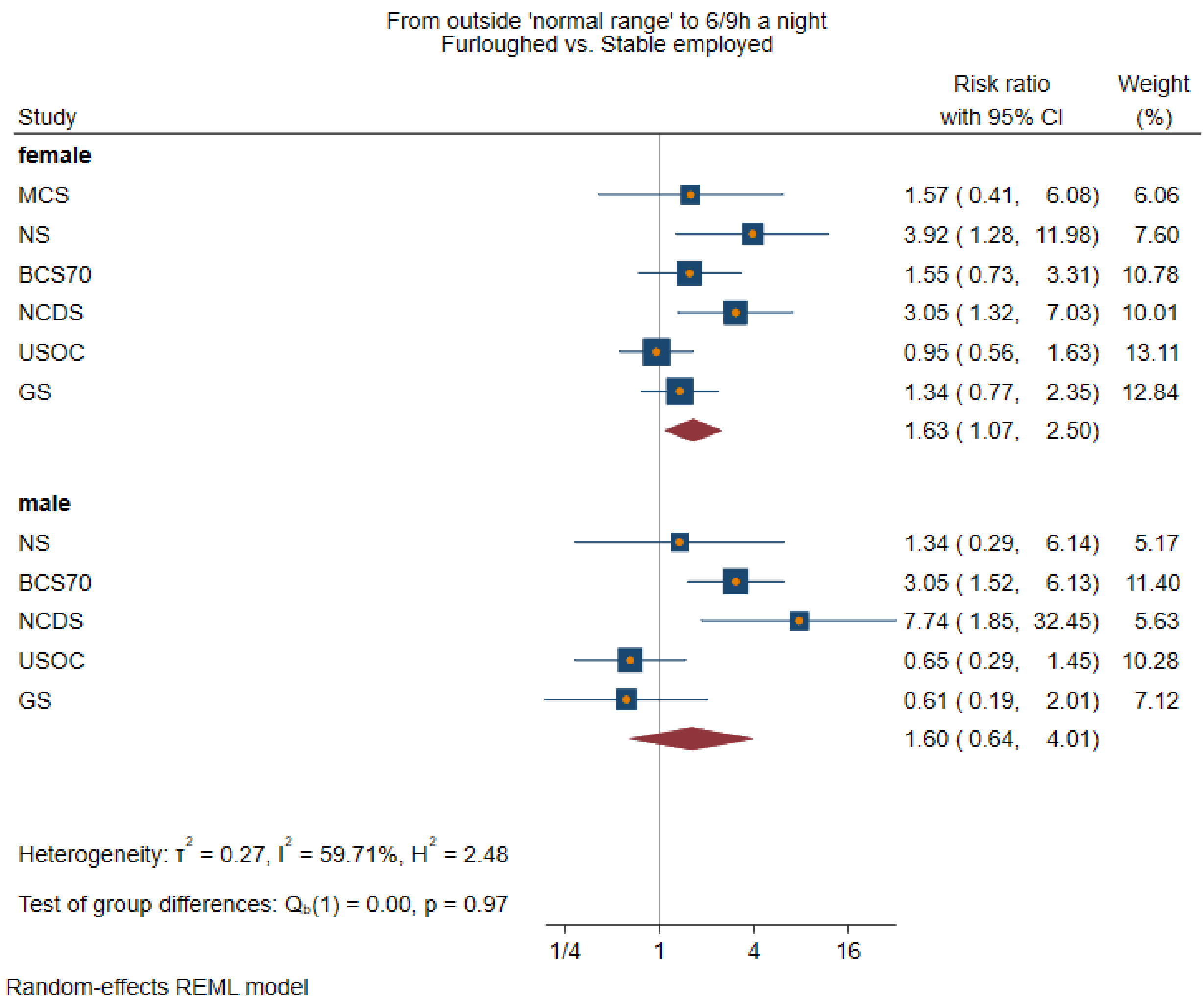

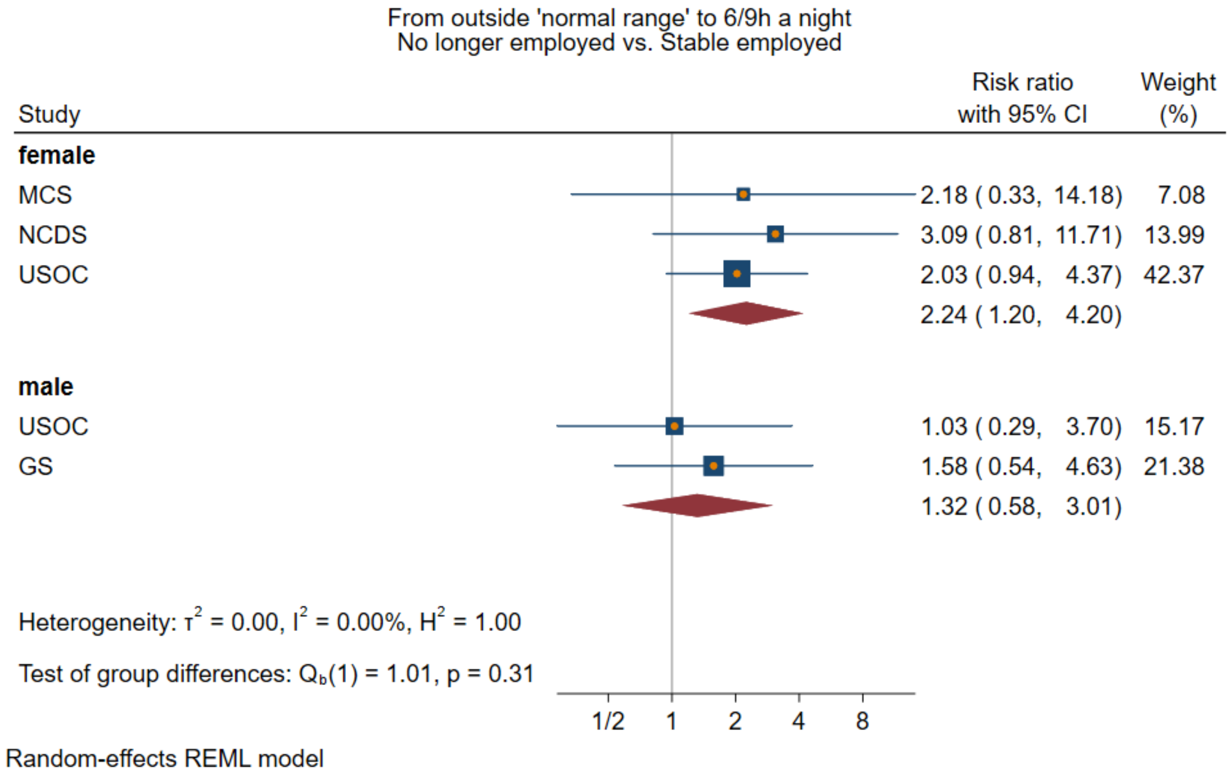

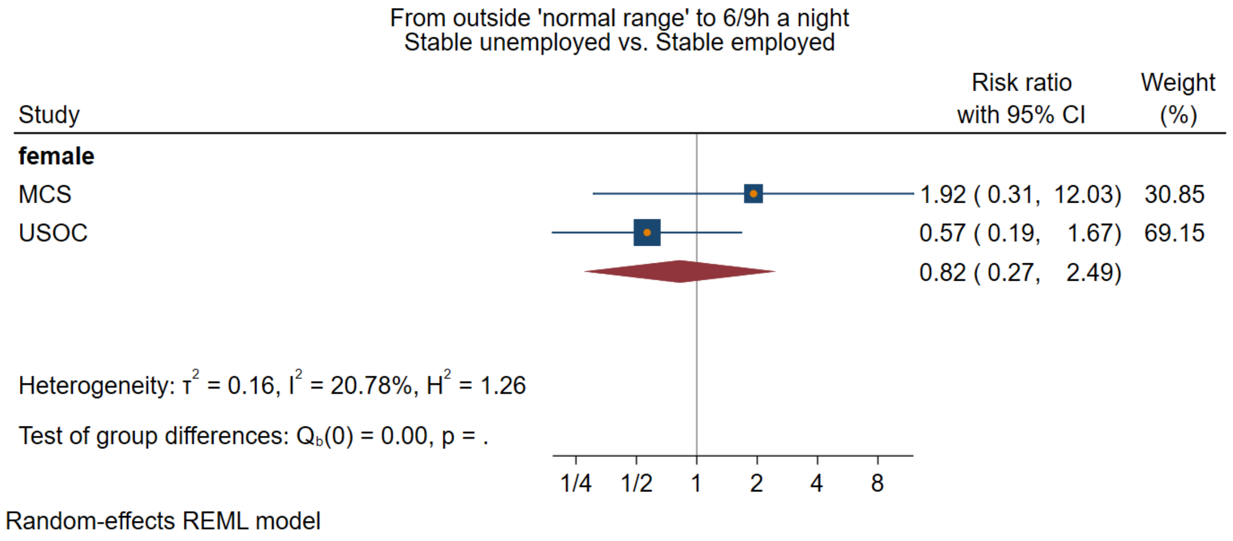
From outside 'normal range' to 6/9h a night
Stable unemployed vs. Stable employed
Study
Risk ratio with 95% CI
Weight (%)
female
MCS 1.92 (0.31, 12.03) 30.85
USOC 0.57 (0.19, 1.67) 69.15
0.82 (0.27, 2.49)
Heterogeneity: τ² = 0.16, I² = 20.78%, H² = 1.26
Test of group differences: Q_b(0) = 0.00, p = .
1/4 1/2 1 2 4 8
Random-effects REML model

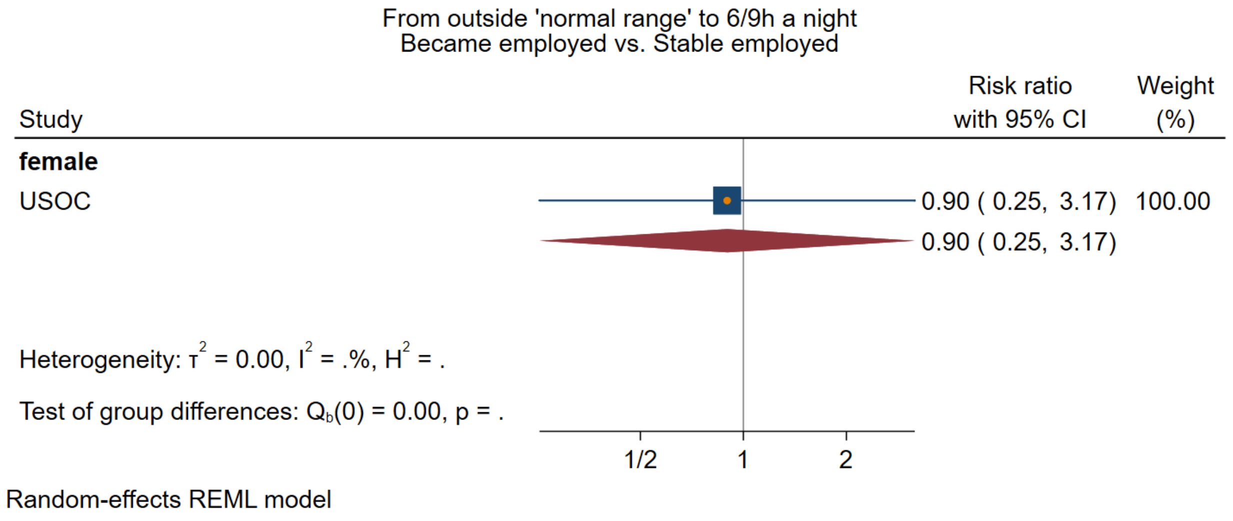
From outside 'normal range' to 6/9h a night
Became employed vs. Stable employed
Study
Risk ratio with 95% CI
Weight (%)
female
USOC 0.90 (0.25, 3.17) 100.00
0.90 (0.25, 3.17)
Heterogeneity: τ² = 0.00, I² = .%, H² = .
Test of group differences: Q_b(0) = 0.00, p = .
1/2 1 2
Random-effects REML model

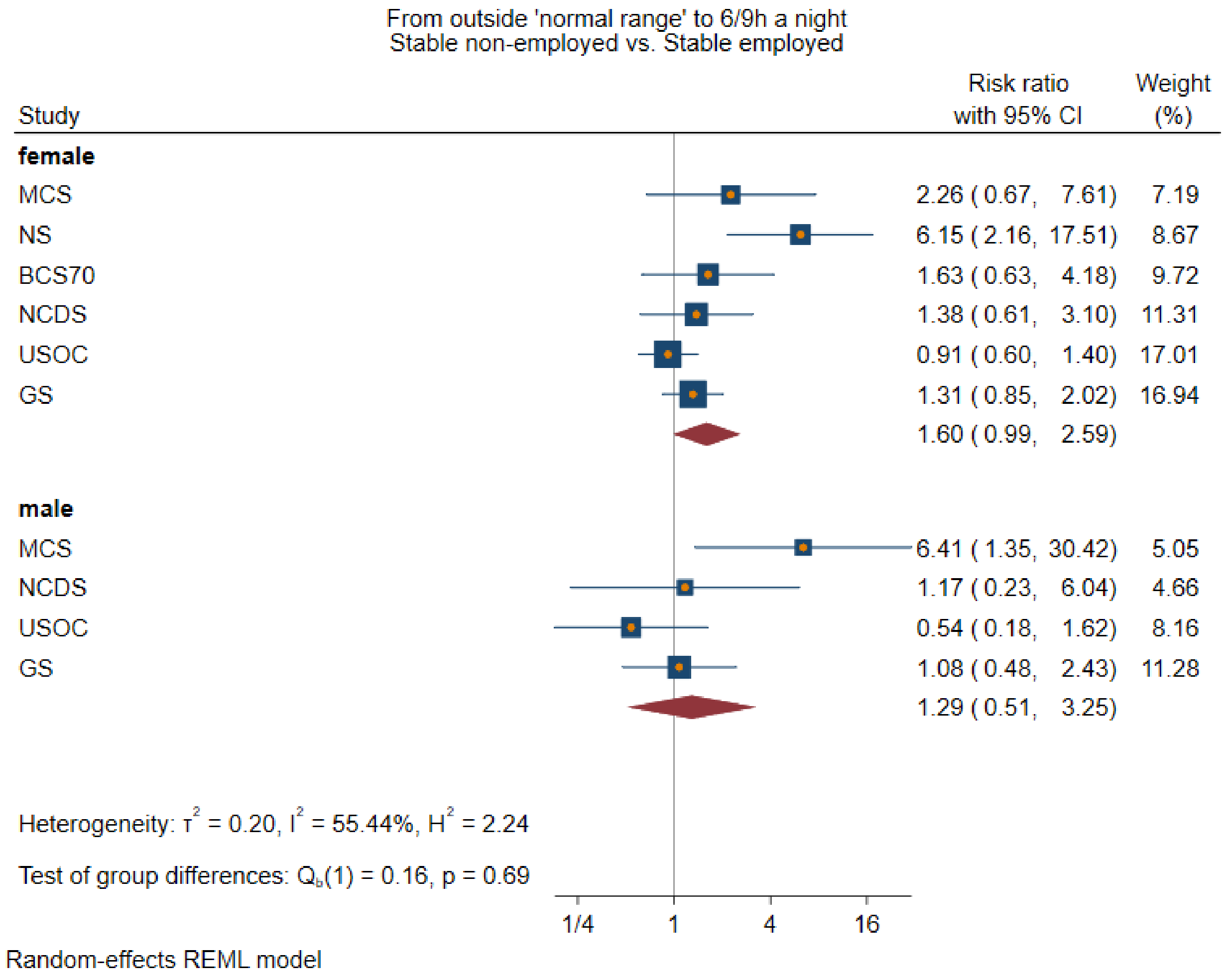
From outside 'normal range' to 6/9h a night
Stable non-employed vs. Stable employed
Study
Risk ratio with 95% CI
Weight (%)
female
MCS 2.26 (0.67, 7.61) 7.19
NS 6.15 (2.16, 17.51) 8.67
BCS70 1.63 (0.63, 4.18) 9.72
NCDS 1.38 (0.61, 3.10) 11.31
USOC 0.91 (0.60, 1.40) 17.01
GS 1.31 (0.85, 2.02) 16.94
1.60 (0.99, 2.59)
male
MCS 6.41 (1.35, 30.42) 5.05
NCDS 1.17 (0.23, 6.04) 4.66
USOC 0.54 (0.18, 1.62) 8.16
GS 1.08 (0.48, 2.43) 11.28
1.29 (0.51, 3.25)
Heterogeneity: τ² = 0.20, I² = 55.44%, H² = 2.24
Test of group differences: Q_b(1) = 0.16, p = 0.69
1/4 1 4 16
Random-effects REML model
