# Supplementary material for: Correction: The UK Coronavirus Job Retention Scheme and diet, physical activity, and sleep during the COVID-19 pandemic: evidence from eight longitudinal population surveys
Source: BMC Med. 2022 Jul 30;20:285. doi: 10.1186/s12916-022-02502-1 (PMC9338472; doi:10.1186/s12916-022-02502-1)

The UK Coronavirus Job Retention Scheme and diet, physical activity and sleep during the COVID-19 pandemic: Evidence from eight longitudinal population studies

List of additional tables:

Table S1: Description of Studies

Table S2: Ethics and data access statements for each study

Table S3: Sample characteristics by study

Table S4: Employment status change by gender, education, and age-group

Table S5: Meta-analysed risk ratios and heterogeneity estimates for associations between changes in employment status and fruit and vegetable consumption: unadjusted, basic & full adjustment results

Table S6: Meta-analysed risk ratios and heterogeneity estimates for associations between changes in employment status and physical activity: unadjusted, basic & full adjustment results

Table S7: Meta-analysed risk ratios and heterogeneity estimates for associations between changes in employment status and sleep: unadjusted, basic & full adjustment results

Figure S8: Causal pathways blocked under differing levels of adjustment

Table S1: Description of studies

Study Population	Design, Sample Frame and Weighting	2020 Age range (years)	Most recent pre-pandemic survey	Details of 2020 COVID Surveys (response rate)	Analytical N
Age Homogenous Cohorts					
MCS: Millennium Cohort Study	A nationally representative cohort of UK children born between Sept 2000 and Jan 2002 with regular follow-up surveys from birth. Weighted for sampling design, pre-pandemic attrition and non-response to COVID surveys.	18-20	2018	May (26.6%)	1924
ALSPAC (G1): Avon Longitudinal Study of Parents and Children- Generation 1 (original young people)	Cohort of children born in the South-West of England between April 1991 and Dec 1992, with regular follow-up surveys from birth. Weighted for pre-pandemic attrition and non-response to COVID surveys.	27-29	2017-2018	June (17.4%)	1273
NS: Next Steps (known as Longitudinal Study of Young People in England)	A nationally representative sample recruited via secondary schools in England at around age 13 with regular follow-up surveys thereafter. Weighted for sampling design, pre-pandemic attrition and non-response to COVID surveys.	29-31	2015	May (20.3%)	1493
BCS70: British Cohort Study 1970	A nationally representative cohort of all children born in Great Britain in one week in 1970, with regular follow-up surveys from birth. Weighted for pre-pandemic attrition and non-response to COVID surveys.	50	2016	May (40.4%)	3050
NCDS: National Child Development Study	A nationally representative cohort of all children born in Great Britain in one week in 1958, with regular follow-up surveys from birth. Weighted for pre-pandemic attrition and non-response to COVID surveys.	62	2013	May (57.9%)	4195
Age Heterogeneous Studies					
USOC: Understanding Society: the UK Household Longitudinal Survey	A nationally representative longitudinal household panel study, based on a clustered-stratified probability sample of UK households, with all adults aged 16+ in chosen households surveyed annually. Weighted for sampling design, pre-pandemic attrition, non-response to COVID surveys, and outcome non-response within COVID surveys.	17-66	2018-2019	Two surveys: April (40.3%) & July (31.2%)	6051
ELSA: English Longitudinal Study of Aging	A nationally representative longitudinal study of individuals aged 50+ living in England, with biennial surveys and periodic refreshing of the sample to maintain representativeness. Weighted for sampling design, pre-pandemic attrition, and non-response to COVID surveys.	52-66	2018-2019	Jun-July (75%)	2417
GS: Generation Scotland: the Scottish Family Health Study	A family-structured, population-based Scottish cohort, with participants aged 18-99 recruited between 2006-2011. No weights were available.	27-66	2006-2011	April-Jun (21.6%)	2618
ALSPAC(G0): Avon Longitudinal Study of Parents and Children- Generation 0 (original parents)	Parents of the ALSPAC(G1) cohort described above, treated as a separate age-heterogenous study population.	44-66	2011-2013	June (12.2%)	2071

Table S2: Ethics and data access statements for each study

The most recent sweeps of the **NCDS, BCS70**, **Next Steps** and **MCS** have all been granted ethical approval by the National Health Service (NHS) Research Ethics Committee and all participants have given informed consent. Data for NCDS (SN 6137), BCS70 (SN 8547), Next Steps (SN 5545), MCS (SN 8682) and all four COVID-19 surveys (SN 8658) are available through the UK Data Service. NSHD data are available on request to the NSHD Data Sharing Committee. Interested researchers can apply to access the NSHD data via a standard application procedure. Data requests should be submitted to mrclha.swiftinfo@ucl.ac.uk; further details can be found at http://www.nshd.mrc.ac.uk/data.aspx. doi:10.5522/NSHD/Q101; doi:10.5522/NSHD/Q10.
Ethical approval was obtained from the **ALSPAC** Ethics and Law Committee and the Local Research Ethics Committees. The study website contains details of all the data that is available through a fully searchable data dictionary and variable search tool: http://www.bristol.ac.uk/alspac/researchers/our-data. ALSPAC data is available to researchers through an online proposal system. Information regarding access can be found on the ALSPAC website (http://www.bristol.ac.uk/media-library/sites/alspac/documents/researchers/data-access/ALSPAC_Access_Policy.pdf).
All wave of **TwinsUK** have received ethical approval associated with TwinsUK Biobank (19/NW/0187), TwinsUK (EC04/015) or Healthy Ageing Twin Study (H.A.T.S) (07/H0802/84) studies from NHS Research Ethics Committees at the Department of Twin Research and Genetic Epidemiology, King's College London. The TwinsUK Resource Executive Committee (TREC) oversees management, data sharing and collaborations involving the TwinsUK registry (for further details see https://twinsuk.ac.uk/resources-for-researchers/access-our-data/).
The University of Essex Ethics Committee has approved all data collection for the **Understanding Society** main study and COVID-19 waves. No additional ethical approval was necessary for this secondary data analysis. All data are available through the UK Data Service (SN 6614 and SN 8644).
Waves 1-9 of **ELSA** were approved through the National Research Ethics Service, while the COVID-19 Sub-study was approved by the UCL Research Ethics Committee. All participants provided informed consent. All data are available through the UK Data Service (SN 8688 and 5050).
**Generation Scotland** obtained ethical approval from the East of Scotland Committee on Medical Research Ethics (on behalf of the National Health Service). Reference number 20/ES/0021. Access to data is approved by the Generation Scotland Access Committee. See https://www.ed.ac.uk/generation-scotland/for-researchers/access or email access@generationscotland.org for further details.

Table S3: Sample characteristics by study

		MCS	NS	BCS	NCDS	GS	USOC	ELSA	ALSPAC-G0	ALSPAC-G1
	**Total N**	1924	1494	3049	4195	2618	6051	2417	2071	1273
Gender	Male	49.1 (46.0-52.2)	45.5 (41.2-50.0)	48.7 (46.9-50.4)	49.4 (47.9-51)	33.8 (32.0-35.6)	48.0 (46.2-49.9)	48.0 (45.6-50.5)	22.8 (20.5-25.4)	29.0 (25.6-32.7)
	Female	50.9 (47.8-54.0)	54.4 (50.0-58.8)	51.3 (49.6-53.1)	50.6 (49-52.1)	66.2 (64.4-68.0)	52.0 (50.1-53.8)	52.0 (49.5-54.4)	77.2 (74.6-79.5)	71.0 (67.3-74.4)
Ethnicity	White	89.4 (85.9-9.2)	87.3 (84.6-89.6)	NA	NA	99.2 (98.8-99.5)	88.7 (86.7-90.5)	90.2 (88.2-91.8)	98.8 (98.0-99.2)	96.5 (94.2-97.9)
	Non-White ethnic minority	10.6 (7.8-14.1)	12.7 (10.4-15.4)	NA	NA	0.8 (0.5-1.2)	11.3 (9.5-13.3)	9.8 (8.2-11.8)	1.2 (0.7-2.0)	3.5 (2.1-5.8)
Education	Degree	41.1 (36.6-45.9)	47.0 (42.9-51.3)	40.3 (38.6-42.1)	35.9 (34.4-37.3)	52.3 (50.4 - 54.2)	39.8 (38.0-41.6)	25.0 (23.0-27.1)	20.8 (19.0-22.8)	30.6 (27.1-34.3)
	Less than degree	58.9 (54.1-63.4)	53.0 (48.7-57.1)	59.7 (58.0-61.4)	64.1 (62.7-65.6)	47.7 (45.8-49.6)	60.2 (58.4-62.0)	75.0 (73.0-77.0)	79.2 (77.2-81.0)	69.4 (65.7-72.9)
Household Composition	Single, with children	NA	NA	NA	NA	4.7 (4.0 - 5.6)	4.1 (3.3-5.1)	8.7 (7.3-10.5)	5.3 (4.0-6.8)	1.1 (0.4-2.6)
	Couple, with children	NA	NA	NA	NA	35.4 (33.6 - 37.3)	25.3 (23.7-26.9)	30.8 (28.6-33.2)	20.8 (18.7-23.1)	11.6 (9.2-14.4)
	Alone or Single (no children)	2.2 (1.1-4.3)	11.0 (8.9-13.7)	14.2 (13.0-15.5)	24.1 (22.8-25.4)	13.0 (11.8-14.4)	39.5 (37.4-41.7)	18.3 (16.3-20.4)	6.8 (5.4-8.6)	8.3 (6.3-10.9)
	Living only with partner	2.1 (1.4-3)	33.3 (29.4-37.3)	17.7 (16.4-19.1)	46.7 (45.2-48.2)	39.6 (37.7 - 41.5)	31.1 (29.5-32.7)	38.4 (36.2-40.8)	33.3 (30.8-36.0)	46.7 (42.8-50.7)
	Partner and others	4.4 (3.1-6.1)	33.0 (29.1-37.3)	55.8 (54-57.5)	22.6 (21.3-23.8)	NA	NA	NA	NA	NA
	Others	91.3 (8.9- 9.3)	22.6 (19.2-26.4)	12.3 (11.2-13.5)	6.7 (6-7.5)	NA	NA	3.7 (3.0-4.7)	33.7 (31.1-36.5)	34.3 (30.7-37.2)
UK Nation	England	83.4 (80.6-85.8)	96.6 (94.6-98)	86.4 (85.1-87.6)	85.1 (84-86.2)	0.5 (0.3-0.8)	86.9 (85.5-88.1)	100.0	NA	NA
	Wales	5.7 (4.5-7.2)	1.4 (0.4-3.8)	5.1 (4.4-5.9)	4.4 (3.8-5.0)	NA	3.9 (3.3-4.5)	NA	NA	NA
	Scotland	8.3 (6.6-10.4)	0.7 (0.2-1.7)	7.6 (6.7-8.6)	8.3 (7.5-9.2)	99.4 (99.1-99.7)	7.2 (6.2-8.5)	NA	NA	NA
	Northern Ireland	1.8 (1.4-2.4)	0.1 (0-0.2)	0.2 (0 – 0.4)	0.4 (0.2-0.6)	NA	2.0 (1.7-2.4)	NA	NA	NA
	Other (Channel Islands/Isle of Man)	0.8 (0.1-2.6)	1.2 (0.8-1.9)	0.8 (0.5-1.2)	1.8 (1.5-2.3)	NA	NA	NA	NA	NA
Pre-Pandemic Mental health - High symptoms		18.2 (15.6-21.2)	25.6 (22-30)	19.2 (17.8-20.6)	14.7 (13.7-15.8)	12.7 (11.5 - 14.1)	21.8 (20.2-23.6)	13.7 (12.0-15.6)	18.9 (16.7-21.4)	22.9 (19.7-26.5)
Pre-Pandemic Fair-Poor Self-Rated health		12.9 (10.6-15.7)	11.4 (8.9-14.7)	14.7 (13.5-16)	18.3 (17.1-19.5)	NA	19.3 (17.7-21.0)	22.9 (20.7-25.3)	NA	NA

Table S4: Employment status change by gender, education, and age-group

	MCS	NS	BCS	NCDS	GS	USOC	ELSA	ALSPAC-G0	ALSPAC-G1
Total N	1924	1494	3049	4195	2618	6051	2417	2071	1273
Age range	18-20	29-31	50	62	27-66	17-66	52-66	50-66	27-29
	%	%	%	%	%	%	%	%	%
Stable Employed	13.6 (11.2-16.3)	60.8 (56.5-64.9)	61.7 (60.0-63.4)	32.8 (31.4-37.6)	62.8 (60.9-64.6)	58.9 (56.9-60.9)	51.1 (48.6-53.6)	58.1 (55.3-60.9)	72.3 (68.5-75.7)
Male	13.9 (10.8-17.7)	69.3 (62.6-75.3)	65.6 (62.9-68.2)	35.1 (33.1-37.2)	65.4 (62.9-68.4)	58.9 (55.6-62.0)	55.3 (51.4-59.1)	57.8 (51.5-63.8)	76.2 (69.9-81.6)
Female	13.3 (10.1-17.4)	53.7 (48.5-58.7)	58.1 (55.8-60.3)	30.5 (28.6-32.4)	61.5 (59.2-63.8)	58.9 (56.7-61.2)	47.2 (44.1-50.3)	58.2 (55.0-61.3)	70.6 (66.1-74.8)
Degree	9.1 (5.7-14.1)	70.6 (65-75.6)	70.2 (67.9-72.4)	33.9 (31.9-36)	67.4 (64.8 - 69.8)	71.5 (69.3-73.6)	54.9 (50.5-59.3)	54.4 (49.7-59.0)	77.0 (70.7-82.2)
No Degree	16.7 (13.9-20)	52.1 (46-58.1)	56.0 (53.5-58.5)	32.2 (30.2-34.1)	57.8 (55.0 - 60.5)	50.6 (47.7-53.4)	49.8 (46.9-52.8)	59.1 (55.7-62.3)	70.0 (65.3-74.3)
Age 16-29	13.6 (11.2-16.3)				73.0 (57.0 - 84.6)	46.7 (41.4-52.2)			72.3 (68.5-75.7)
Age 30-49		60.8 (56.5-64.9)			78.2 (75.2 - 80.9)	70.0 (67.0-72.9)			
Age 50-66			61.7 (60.0-63.4)	32.8 (31.4-37.6)	55.5 (53.2 - 57.8)	55.2 (53.0-57.4)	51.1 (48.6-53.6)	58.1 (55.3-60.9)	
Furloughed	16.9 (14-20.1)	25.1 (21.5-29)	24.7 (23.2-26.2)	21.1 (19.9-22.4)	8.4 (7.4-9.5)	14.5 (13.0-16.0)	13.5 (11.9-15.2)	12.8 (10.9-15.0)	16.1 (13.3-19.3)
Male	13.5 (9.5-18.6)	22.6 (17.3-29)	24.4 (22- 26.8)	25 (23.2-27)	10.1 (8.3-12.2)	14.7 (12.5-17.2)	12.8 (10.4-15.5)	13.5 (9.4-19.0)	13.3 (9.2-19.1)
Female	20.2 (16.8-24.1)	27.1 (22.5-32.6)	24.9 (23.0-27.0)	17.3 (15.7-18.9)	7.5 (6.3-8.8)	14.2 (12.7-15.9)	14.1 (12.1-16.4)	12.6 (10.5-15.1)	17.1 (13.8-21.2)
Degree	10.8 (8.2-14.1)	18.5 (14.3-23.5)	20.4 (18.5-22.5)	13.5 (12-15)	5.0 (4.0-6.3)	10.0 (8.6-11.5)	11.5 (9.0-14.6)	7.8 (5.6-10.9)	14.3 (9.9-20.3)
No Degree	21.2 (17.3-25.7)	30.9 (25.5-36.9)	27.5 (25.3-29.8)	25.4 (23.6-27.6)	12.0 (10.3 - 13.9)	17.4 (15.3-19.7)	14.1 (12.2-16.2)	14.1 (11.8-16.8)	16.9 (13.6-20.9)

Age 16-29	16.9 (14-20.1)				2.7 (0.5-13.8)	17.6 (14.1-21.7)			16.1 (13.3-19.3)
Age 30-49		25.1 (21.5-29)			10.5 (8.5-12.8)	14.9 (12.8-17.2)			
Age 50-66			24.7 (23.2-26.2)	21.1 (19.9-22.4)	7.5 (6.4-8.8)	11.9 (10.5-13.3)	13.5 (11.9-15.2)	12.8 (10.9-15.0)	
No Longer Employed	3.7 (2.6-5.2)	2.5 (1.6-3.8)	1.9 (1.5-2.5)	2.8 (2.4-3.4)	3.3 (2.7- 4.1)	3.3 (2.6-4.2)	2.0 (1.4-2.7)	10.3 (8.8-12.1)	5.4 (3.8-7.5)
Male	4.6 (2.8-7.4)	1.5 (0.8-2.8)	2.0 (1.3-3.0)	3.0 (2.3-3.8)	3.4 (2.4-4.8)	3.8 (2.6-5.6)	2.2 (1.3-3.6)	10.6 (7.6-14.6)	5.3 (2.9-9.3)
Female	2.8 (2.0-4.0)	3.3 (1.9-5.6)	1.8 (1.3-2.6)	2.7 (2.1-3.5)	3.3 (2.5-4.2)	2.8 (2.2-3.7)	1.8 (1.2-2.6)	10.3 (8.6-12.2)	5.4 (3.6-8.1)
Degree	4.2 (2.8-6.4)	3.2 (1.7-5.8)	1.7 (1.2 -2.5)	2.8 (2.2-3.6)	3.3 (2.5-4.4)	3.1 (2.3-4.1)	1.5 (0.9-2.7)	12.7 (10.1-16.1)	4.9 (2.9-8.3)
No Degree	3.3 (2-5.5)	1.8 (1-3.1)	2.0 (1.4-2.9)	2.9 (2.2-3.7)	3.4 (2.5-4.5)	3.4 (2.4-4.9)	2.1 (1.5-3.1)	9.7 (8.0-11.7)	5.6 (3.6-8.5)
Age 16-29	3.7 (2.6-5.2)				--	5.1 (3.4-7.7)			5.4 (3.8-7.5)
Age 30-49		2.5 (1.6-3.8)			2.8 (1.9-4.2)	2.6 (1.5-4.6)			
Age 50-66			1.9 (1.5-2.5)	2.8 (2.4-3.4)	3.6 (2.8-4.6)	2.8 (2.2-3.5)	2.0 (1.4-2.7)	10.3 (8.8-12.1)	
Stable Unemployed	5.6 (3.4-9.0)	1.5 (0.7-2.8)	0.9 (0.6-1.3)	2.0 (1.6-2.5)	0.5 (0.3-0.8)	2.0 (1.4-2.8)	3.2 (2.2-4.4)	5.1 (4.1-6.5)	2.0 (1.3-3.1)
Male	8.7 (4.6-15.8)	1.1 (0.4-2.8)	0.2 (0.03-0.7)	2.5 (1.9-3.2)	0.8 (0.4-1.6)	1.8 (1.0-3.2)	4.0 (2.5-6.3)	7.3 (4.5-11.6)	2.4 (1.2-4.9)
Female	2.6 (1.6-4)	1.8 (0.6-4.1)	0.8 (0.4-1.3)	1.6 (1.2-2.2)	0.3 (0.2-0.8)	2.2 (1.5-3.3)	2.4 (1.5-3.9)	4.5 (3.5-5.8)	1.8 (1.0-3.2)
Degree	2.3 (1.1-4.3)	0.7 (0.2-2.1)	0.7 (0.4 - 1.3)	1.6 (1.1-2.3)	0.3 (0.1-0.7)	0.4 (0.2-0.7)	2.5 (1.1-5.3)	9.2 (6.9-12.1)	1.1 (0.4-3.1)
No Degree	7.9	2.1	1.1	2.3	0.7	3.1	3.4	4.1	2.4

	(4.5-13.4)	(0.9-4.6)	(0.6-1.7)	(1.7-3.0)	(0.4-1.4)	(2.1-4.4)	(2.3-4.9)	(2.9-5.6)	(1.5-3.9)
Age 16-29	5.6<br>(3.4-9.0)				--	3.5<br>(1.8-6.9)			2.0<br>(1.3-3.1)
Age 30-49		1.5<br>(0.7-2.8)			0.1<br>(0.0-0.7)	1.3<br>(0.9-2.1)			
Age 50-66			0.9<br>(0.6-1.3)	2.0<br>(1.6-2.5)	0.7<br>(0.4-1.2)	1.7<br>(1.1-2.5)	3.2<br>(2.2-4.4)	5.1<br>(4.1-6.5)	
Became Employed	1.2<br>(0.7-2.0)	0.9<br>(0.5-1.7	0.5<br>(0.0-1.0)	0.7<br>(0.5-1.0)	0.6<br>(0.3 - 0.9)	1.1<br>(0.8-1.6)	0.4<br>(0.2-0.7)	0.1<br>(0.0-0.4)	1.1<br>(0.6-2.0)
Male	1.3<br>(0.5-2.8)	1.0<br>(0.4-2.4)	0.2<br>(0.0-0.7)	0.8<br>(0.5-1.4)	0.2<br>(0.1-0.8)	1.3<br>(1.0-2.2)	0.2<br>(0.0-0.6)	NA	1.6<br>(0.6-4.3)
Female	1.2<br>(0.6-2.2)	0.8<br>(0.3-2.0)	0.8<br>(0.5-1.3)	0.5<br>(0.3-1.0)	1.0<br>(0.6-1.7)	1.0<br>(0.6-1.6)	0.5<br>(0.3-1.2)	0.1<br>(0.0-0.5)	0.9<br>(0.4-1.9)
Degree	1.0<br>(0.2-2.1)	0.7<br>(0.4-1.5)	0.6<br>(0.3-1.1)	0.9<br>(0.5-1.4)	0.5<br>(0.2-1.1)	1.5<br>(0.9-2.4)	0.5<br>(0.2-1.7)	NA	2.2<br>(1.0-4.5)
No Degree	1.4<br>(0.7-2.6)	1.1<br>(0.4-2.6)	0.5<br>(0.2- 0.9)	0.6<br>(0.3-1)	0.6<br>(0.3-1.3)	0.8<br>(0.5-1.5)	0.3<br>(0.1-0.7)	0.1<br>(0.0-0.5)	0.6<br>(0.2-1.8)
Age 16-29	1.2<br>(0.7-2.0)				2.7<br>(0.5-13.8)	1.4<br>(0.6-3.0)			1.1<br>(0.6-2.0)
Age 30-49		0.9<br>(0.5-1.7			0.2<br>(0.0-0.9)	1.2<br>(0.7-2.1)			
Age 50-66			0.5<br>(0.0-1.0)	0.7<br>(0.5-1.0)	0.7<br>(0.4-1.2)	0.9<br>(0.6-1.4)	0.4<br>(0.2-0.7)	0.1<br>(0.0-0.4)	
Stable Non-Employed	59.0<br>(53.9-63.9)	9.3<br>(7.1-12.0)	10.3<br>(9.2-11.4)	40.6<br>(39.1-42.1)	24.4<br>(22.8 - 26.1)	20.2<br>(18.6-22.0)	30.0<br>(27.9-32.2)	13.5<br>(11.7-15.6)	3.3<br>(2.0-5.1)
Male	58.1<br>(51.0-64.8)	4.4<br>(2.3-8.1)	6.8<br>(5.5-8.3)	33.5<br>(31.5-35.6)	20.1<br>(17.6-22.9)	19.6<br>(16.8-22.7)	25.6<br>(22.5-29.1)	10.8<br>(7.6-15.2)	1.1<br>(0.5-2.4)
Female	59.9<br>(55.5-64.2)	13.4<br>(10.0-17.5)	13.6<br>(12.1-15.2)	47.4<br>(45.3-49.5)	26.6<br>(24.6-28.8)	20.8<br>(19.0-22.7)	34.0<br>(31.2-36.9)	14.3<br>(12.2-16.7)	4.1<br>(2.5-6.7)
Degree	72.0	6.3	6.3	47.3	23.5	13.5	29.1	15.7	0.5

	(67.1-77.6)	(4.0-10.0)	(5.2-7.7)	(45.1-49.5)	(21.3-25.8)	(12.1-15.1)	(25.4-33.1)	(12.4-19.7)	(0.2-1.3)
No Degree	49.4<br>(43.7-55.2)	12.0<br>(8.6-16.3)	12.9<br>(11.3-14.7)	36.8<br>(34.8-38.8)	25.5<br>(23.1-28.0)	24.6<br>(22.1-27.3)	30.3<br>(27.8-33.0)	12.9<br>(10.8-15.4)	4.4<br>(2.7-7.1)
Age 16-29	59.0<br>(53.9-63.9)				21.6<br>(11.4-37.2)	25.6<br>(20.6-31.4)			3.3<br>(2.0-5.1)
Age 30-49		9.3<br>(7.1-12.0)			8.1<br>(6.4-10.2)	10.0<br>(8.0-12.2)			
Age 50-66			10.3<br>(9.2-11.4)	40.6<br>(39.1-42.1)	32.0<br>(29.9-34.2)	27.6<br>(25.7-29.5)	30.0<br>(27.9-32.2)	13.5<br>(11.7-15.6)	

Table S5: Meta-analysed risk ratios and heterogeneity estimates for associations between changes in employment status and fruit and vegetable consumption: unadjusted, basic & full adjustment results

Ref Cat: Stable Employed	Currently eats ≤2 fruit & veg portions			Fewer fruit and vegetable portions			More fruit and vegetable portions		
	**Unadjusted**	**Basic**	**Full**	**Unadjusted**	**Basic**	**Full**	**Unadjusted**	**Basic**	**Full**
**Furloughed**	1.22 [1.10-1.36]	1.13 [1.00-1.26]	0.97 [0.87-1.09]	1.01 [0.91-1.11]	0.97 [0.84-1.12]	0.96 [0.87-1.08]	1.19 [1.02-1.40]	1.22 [1.04-1.44]	1.22 [1.04-1.43]
I^2 %	0.0	23.59	41.83	3.65	28.85	8.96	52.93	54.04	52.46
**No longer employed**	1.05 [0.75-1.46]	1.03 [0.80-1.33]	0.95 [0.81-1.12]	0.79 [0.49-1.29]	0.75 [0.44-1.28]	0.74 [0.44-1.22]	1.01 [0.82,1.25]	1.02 [0.83-1.26]	1.02 [0.83-1.25]
I^2 %	47.44	31.02	0.0	62.10	68.10	64.10	0.00	0.83	0.00
**Stable Unemployed**	1.66 [1.32-2.08]	1.35 [1.07-1.70]	1.00 [0.83-1.20]	1.11 [0.58-2.12]	1.01 [0.49-2.11]	0.92 [0.47-1.81]	1.10 [0.65-1.85]	1.14 [0.72-1.78]	1.16 [0.82-1.63]
I^2 %	0.0	0.0	0.00	67.66	76.98	76.02	58.09	45.08	21.37

'Basic' adjustment includes age, sex, ethnicity, education, UK nation, and household composition. 'Full' adjustment additionally includes pre-pandemic measures of mental health, self-rated health, diet, exercise and sleep. Pre-pandemic diet was not included in models of dietary change (fewer or more fruit and vegetable portions).

Table S6: Meta-analysed risk ratios and heterogeneity estimates for associations between changes in employment status and physical activity: unadjusted, basic & full adjustment results

**Ref Cat: Stable Employed**	≤ 3 days a week of at least 30m exercise			Less PA/fewer days of +30m exercise			More PA/more days of +30m exercise		
	**Unadjusted**	**Basic**	**Full**	**Unadjusted**	**Basic**	**Full**	**Unadjusted**	**Basic**	**Full**
**Furloughed**	0.92 [0.81-1.05]	0.89 [0.78-1.02]	0.85 [0.75-0.97]	1.08 [1.01-1.15]	1.08 [0.98-1.18]	1.07 [0.97-1.17]	1.15 [1.01-1.31]	1.17 [1.03-1.34]	1.19 [1.04-1.36]
I^2 %	52.16	57.74	58.73	0.00	34.92	38.53	71.62	74.49	75.78
**No longer employed**	1.10 [0.88-1.36]	1.10 [0.90-1.34]	1.02 [0.84-1.24]	1.06 [0.94-1.19]	1.08 [0.94-1.23]	1.07 [0.93-1.23]	1.11 [0.99-1.24]	1.12 [0.99-1.25]	1.12 [1.00-1.26]
I^2 %	47.03	37.26	40.11	0.00	13.61	21.00	0.00	3.48	0.17
**Stable Unemployed**	1.63 [1.20-2.22]	1.52 [1.15-2.02]	1.15 [0.90-1.47]	0.83 [0.67-1.02]	0.83 [0.68-1.02]	0.85 [0.67-1.07]	0.99 [0.75-1.31]	1.10 [0.93-1.32]	1.12 [0.95-1.33]
I^2 %	68.62	59.09	62.25	6.23	0.00	17.33	46.29	1.46	0.00

'Basic' adjustment includes age, sex, ethnicity, education, UK nation, and household composition. 'Full' adjustment additionally includes pre-pandemic measures of mental health, self-rated health, diet, exercise and sleep. Pre-pandemic exercise was not included in models of exercise change (less or more PA/days of +30m exercise).

Table S7: Meta-analysed risk ratios and heterogeneity estimates for associations between changes in employment status and sleep: unadjusted, basic & full adjustment results

Ref Cat: Stable Employed	Sleep level: <6 hours/>9 hours			Sleep change: becomes abnormal (from 6-9h to outside normal hours)			Sleep change: becomes normal (from outside 6-9h to within)		
	Unadjusted	Basic	Full	Unadjusted	Basic	Full	Unadjusted	Basic	Full
**Furloughed**	1.45 [1.01-2.08]	1.33 [0.94-1.89]	1.28 [0.92-1.78]	1.61 [1.11-2.34]	1.51 [1.07-2.14]	1.46 [1.03-2.07]	1.81 [1.08-3.02]	1.81 [1.04-3.15]	1.78 [1.03-3.07]
I^2 %	85.11	84.51	84.34	77.38	74.71	75.13	70.60	75.87	75.70
**No longer employed**	1.50 [0.86-2.60]	1.51 [0.90-2.52]	1.38 [0.81-2.36]	1.54 [0.90-2.64]	1.52 [0.91-2.57]	1.44 [0.84-2.49]	1.83 [1.11-3.00]	1.75 [1.06-2.89]	1.53 [0.93-2.50]
I^2 %	82.77	80.42	82.29	70.75	68.11	70.63	18.30	13.30	4.52
**Stable Unemployed**	2.46 [1.23-4.88]	2.09 [1.13-3.87]	1.44 [0.69-2.99]	1.93 [1.08-3.46]	1.77 [1.05-2.99]	1.45 [0.84-2.51]	1.38 [0.23-8.39]	1.35 [0.16-11.4]	1.13 [0.10-12.6]
I^2 %	83.19	79.03	85.86	40.46	32.29	33.89	68.82	78.73	85.65

Ref Cat: Stable Employed	Sleep change: more than before			Sleep change: less than before		
	Unadjusted	Basic	Full	Unadjusted	Basic	Full
**Furloughed**	1.48 [1.18-1.85]	1.63 [1.39-1.91]	1.63 [1.39-1.91]	1.03 [0.85-1.24]	0.91 [0.76-1.10]	0.90 [0.75-1.07]
I^2 %	89.43	80.01	80.20	73.84	71.99	70.47
**No longer employed**	1.34 [1.13-1.59]	1.48 [1.27-1.73]	1.45 [1.24-1.70]	1.16 [0.93-1.44]	1.02 [0.88-1.20]	0.99 [0.85-1.16]
I^2 %	37.57	36.31	39.21	51.59	0.00	0.00
**Stable Unemployed**	1.31 [1.07-1.60]	1.31 [1.08-1.60]	1.30 [1.06-1.59]	1.17 [0.88-1.55]	1.15 [0.87-1.51]	1.08 [0.85-1.37]
I^2 %	3.95	2.16	5.00	44.04	39.75	21.23

'Basic' adjustment includes age, sex, ethnicity, education, UK nation, and household composition. 'Full' adjustment additionally includes pre-pandemic measures of mental health, self-rated health, diet, exercise and sleep. Pre-pandemic sleep was not included in models of sleep change (all outcomes except sleep level).

Figure S8: Causal pathways blocked under differing levels of adjustment

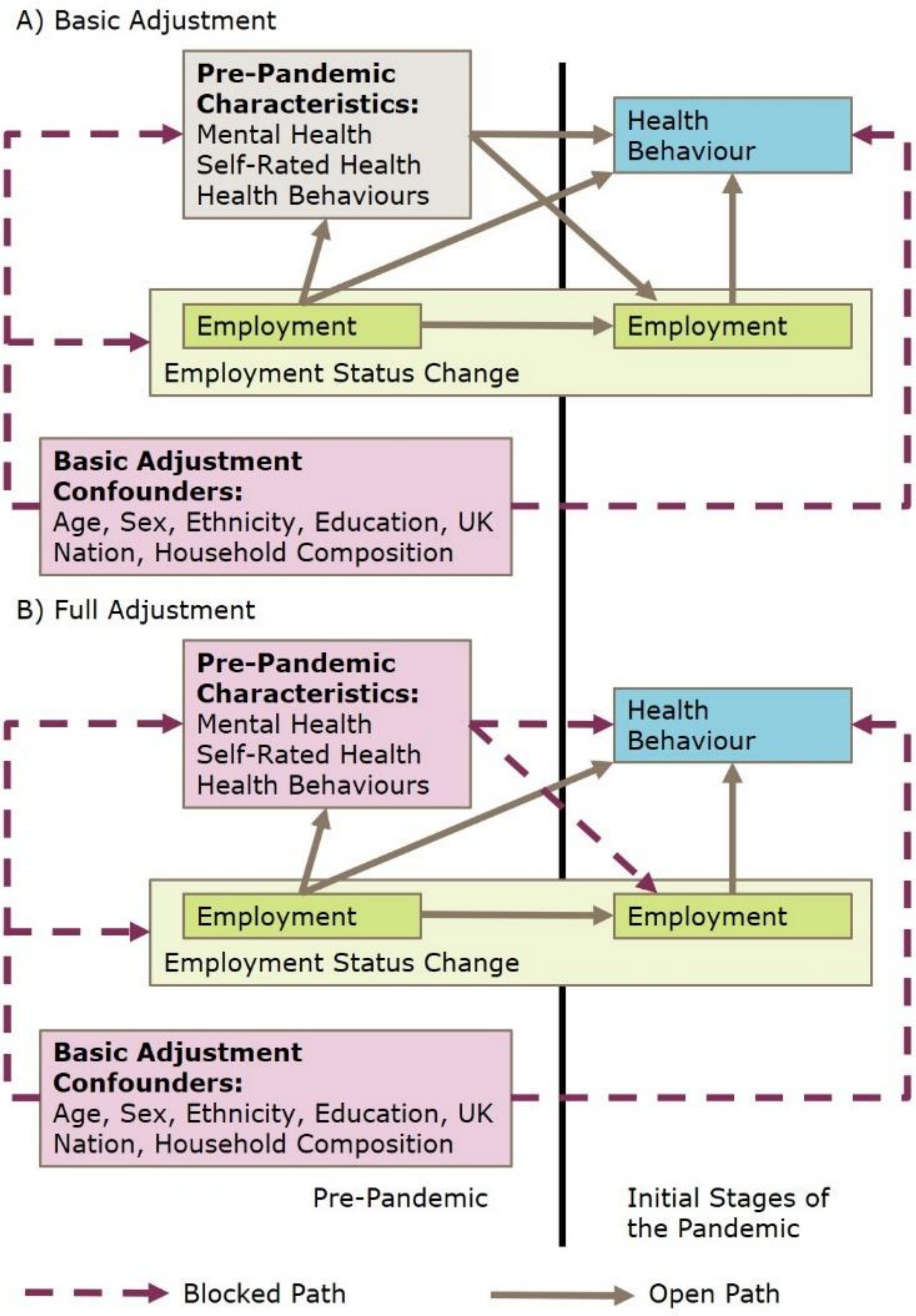

Supplement: Supplementary file 1 — Additional file 1. [file 12916_2022_2502_MOESM1_ESM.pdf]
