# Supplementary material for: Correction: The UK Coronavirus Job Retention Scheme and diet, physical activity, and sleep during the COVID-19 pandemic: evidence from eight longitudinal population surveys
Source: BMC Med. 2022 Jul 30;20:285. doi: 10.1186/s12916-022-02502-1 (PMC9338472; doi:10.1186/s12916-022-02502-1)
Supplement: Supplementary file 2 — Additional file 3. [file 12916_2022_2502_MOESM2_ESM.pdf]

Additional File 3: Meta-analysis results

Summary Tables

Table 1. Main analysis excluding studies with ≤5 cell counts for exposure-outcome 2
Table 2. Main analysis excluding studies with ≤2 cell counts for exposure-outcome 3
Table 3. Main analysis excluding studies with zero cell counts for exposure-outcome 4
Table 4. Analysis of change excluding studies with ≤ 5 cell counts 5
Table 5. Analysis of change excluding studies with ≤ 2 cell counts 8
Table 6. Analysis of change excluding studies with zero cell counts 11

Forest Plots (*Unadjusted, Basic, and Fully adjusted*)

Figure set 1: Currently eats 2 or fewer fruit & veg 14
Figure set 2: Fewer fruit and vegetables 19
Figure set 3: More fruit and vegetables 24
Figure set 4: Less than 3 days a week of at least 30 min exercise 29
Figure set 5: Less time/fewer days of physical exercise 34
Figure set 6: More time/days of physical exercise 40
Figure set 7: Sleeps outside 'Normal Range' (i.e. <6 or 9+ hours) 46
Figure set 8: Sleeps less than before 51
Figure set 9: Sleeps more than before 57
Figure set 10: From 6/9 hours a night to outside 'normal range' 63
Figure set 11: From outside 'normal range' to 6/9h a night 68

Notes: Basic adjustment includes socio-demographic characteristics: age (only in age-heterogeneous studies), sex, ethnicity (except the BCS70 and NCDS cohorts which were nearly are almost entirely white), education, UK nation (except ALSPAC, GS and ELSA which only had participants from a single country), and household composition. Full adjustment additionally includes pre-pandemic measures of psychological distress, self-rated health, and health behaviours.

Table 1. Main analysis excluding studies with ≤5 cell counts for exposure-outcome

		Currently eats 2 or fewer fruit & veg				Less than 3 days a week of at least 30min exercise				Sleeps outside 'Normal Range' (i.e. <6 or 9+ hours)			
		RR	Lower CI	Upper CI	I2%	RR	Lower CI	Upper CI	I2%	RR	Lower CI	Upper CI	I2%
Furloughed	Overall	0.97	0.87	1.09	41.83	0.85	0.75	0.97	58.73	1.28	0.92	1.78	84.34
	female	0.84	0.68	1.04	65.28	0.87	0.79	0.95	0	1.30	0.89	1.88	83.76
	male	1.11	1.01	1.22	0	0.88	0.75	1.03	41.13	1.14	0.88	1.47	24.55
	Degree	0.96	0.71	1.30	76.09	0.81	0.72	0.91	0.00	1.28	0.81	2.01	73.72
	No degree	1.00	0.91	1.09	0.00	0.88	0.75	1.03	60.15	1.20	0.94	1.54	61.57
	16-29y	0.84	0.57	1.25	66.75	0.76	0.56	1.03	0.00	1.39	0.31	6.16	90.51
	30-49y	0.89	0.77	1.04	0.00	0.83	0.59	1.17	65.57	1.10	0.78	1.54	36.92
	50+y	1.06	0.96	1.17	0.00	0.86	0.72	1.03	68.17	1.10	0.88	1.39	46.33
No longer employed	Overall	0.95	0.81	1.12	0	1.02	0.85	1.24	40.11	1.38	0.81	2.36	82.29
	female	0.81	0.55	1.18	49.84	1.11	0.94	1.31	1.06	1.34	0.83	2.16	68.97
	male	1.14	0.93	1.39	0	0.98	0.80	1.21	0	1.28	0.31	5.20	85.62
	Degree	0.92	0.62	1.36	0.00	1.08	0.80	1.47	53.38	1.58	0.41	6.00	74.04
	No degree	1.08	0.89	1.30	0.00	0.96	0.80	1.15	0.00	1.46	0.86	2.49	71.78
	16-29y	0.92	0.59	1.43	45.37	0.98	0.46	2.09	73.41	3.80	2.35	6.15	
	30-49y	0.77	0.41	1.44	59.88	1.28	0.96	1.71	0.00	1.36	0.58	3.20	57.29
	50+y	0.99	0.78	1.25	0.00	1.02	0.86	1.20	0.03	1.09	0.70	1.68	56.48
Stable unemployed	Overall	1.00	0.83	1.20	0	1.21	0.91	1.61	74.71	1.76	0.85	3.64	85.29
	female	0.94	0.72	1.23	0	1.20	0.90	1.59	42.82	2.34	1.43	3.81	51.22
	male	1.07	0.84	1.36	0	1.17	0.74	1.84	76.87	1.66	0.85	3.23	63.24
	Degree	1.04	0.63	1.73	*	0.83	0.69	0.99	*	no information			
	No degree	1.03	0.84	1.26	0.00	1.33	0.96	1.82	72.69	2.19	1.48	3.25	28.66
	16-29y	1.27	0.63	2.56	87.75	1.50	0.41	5.39	91.84	2.92	1.69	5.05	*
	30-49y	0.83	0.36	1.93	69.28	1.41	1.15	1.72	0.00	2.09	0.89	4.89	70.99
	50+y	0.71	0.38	1.33	64.94	1.17	0.90	1.53	26.48	0.96	0.39	2.39	78.41

* indicates only one study included

Table 2. Main analysis excluding studies with ≤2 cell counts for exposure-outcome

		Currently eats 2 or fewer fruit & veg				Less than 3 days a week of at least 30min exercise				Sleeps outside 'Normal Range' (i.e. <6 or 9+ hours)			
		RR	Lower CI	Upper CI	I2%	RR	Lower CI	Upper CI	I2%	RR	Lower CI	Upper CI	I2%
Furloughed	Overall	0.97	0.87	1.09	41.83	0.85	0.75	0.97	58.73	1.28	0.92	1.78	84.34
	female	0.84	0.68	1.04	65.28	0.87	0.79	0.95	0	1.30	0.89	1.88	83.76
	male	1.11	1.01	1.22	0	0.88	0.75	1.03	41.13	1.14	0.88	1.47	24.55
	Degree	0.96	0.71	1.30	76.09	0.81	0.72	0.91	0.00	1.18	0.80	1.74	65.59
	No degree	1.00	0.91	1.09	0.00	0.88	0.75	1.03	60.15	1.20	0.94	1.54	61.57
	16-29y	0.84	0.57	1.25	66.75	0.76	0.56	1.03	0.00	1.39	0.31	6.16	90.51
	30-49y	0.89	0.77	1.04	0.00	0.83	0.59	1.17	65.57	1.10	0.78	1.54	36.92
	50+y	1.06	0.96	1.17	0.00	0.86	0.72	1.03	68.17	1.10	0.88	1.39	46.33
No longer employed	Overall	0.95	0.81	1.12	0	1.02	0.85	1.24	40.11	1.38	0.81	2.36	82.29
	female	0.81	0.55	1.18	49.84	1.11	0.94	1.31	1.06	1.34	0.83	2.16	68.97
	male	1.14	0.93	1.39	0	0.98	0.80	1.21	0	1.23	0.52	2.92	74.05
	Degree	0.79	0.46	1.36	69.70	1.08	0.80	1.47	53.38	1.11	0.72	1.71	12.58
	No degree	1.00	0.84	1.19	0.00	0.96	0.80	1.15	0.00	1.46	0.86	2.49	71.78
	16-29y	0.92	0.59	1.43	45.37	0.98	0.46	2.09	73.41	3.80	2.35	6.15	*
	30-49y	0.77	0.41	1.44	59.88	1.28	0.96	1.71	0.00	1.16	0.60	2.26	45.34
	50+y	0.99	0.78	1.25	0.00	1.02	0.86	1.20	0.03	1.09	0.70	1.68	56.48
Stable unemployed	Overall	1.00	0.83	1.20	0	1.15	0.90	1.47	62.25	1.44	0.69	2.99	85.86
	female	0.90	0.70	1.17	0	1.09	0.78	1.52	56.95	1.31	0.54	3.17	85.51
	male	1.28	0.87	1.88	63.67	1.15	0.89	1.47	50.01	2.09	1.04	4.20	71.15
	Degree	1.01	0.50	2.06	82.44	1.02	0.70	1.50	66.83	1.97	1.17	3.31	35.72
	No degree	1.02	0.83	1.24	0.00	1.32	1.03	1.68	58.58	1.65	0.75	3.65	82.22
	16-29y	1.27	0.63	2.56	87.75	1.50	0.41	5.39	91.84	2.75	1.63	4.63	0.00
	30-49y	0.83	0.36	1.93	69.28	1.41	1.15	1.72	0.00	2.09	0.89	4.89	70.99
	50+y	0.71	0.38	1.33	64.94	1.12	0.93	1.34	0.00	0.98	0.53	1.80	60.57

* indicates only one study included

Table 3. Main analysis excluding studies with zero cell counts for exposure-outcome

		Currently eats 2 or fewer fruit & veg				Less than 3 days a week of at least 30min exercise				Sleeps outside 'Normal Range' (i.e. <6 or 9+ hours)			
		RR	Lower CI	Upper CI	I2%	RR	Lower CI	Upper CI	I2%	RR	Lower CI	Upper CI	I2%
Furloughed	Overall	0.97	0.87	1.09	41.83	0.85	0.75	0.97	58.73	1.28	0.92	1.78	84.34
	female	0.84	0.68	1.04	65.28	0.87	0.79	0.95	0	1.30	0.89	1.88	83.76
	male	1.11	1.01	1.22	0	0.88	0.75	1.03	41.13	1.14	0.88	1.47	24.55
	Degree	0.96	0.71	1.30	76.09	0.81	0.72	0.91	0.00	1.18	0.80	1.74	65.59
	No degree	1.00	0.91	1.09	0.00	0.88	0.75	1.03	60.15	1.20	0.94	1.54	61.57
	16-29y	0.84	0.57	1.25	66.75	0.76	0.56	1.03	0.00	1.39	0.31	6.16	90.51
	30-49y	0.89	0.77	1.04	0.00	0.83	0.59	1.17	65.57	1.10	0.78	1.54	36.92
	50+y	1.06	0.96	1.17	0.00	0.86	0.72	1.03	68.17	1.10	0.88	1.39	46.33
No longer employed	Overall	0.95	0.81	1.12	0	1.02	0.85	1.24	40.11	1.38	0.81	2.36	82.29
	female	0.80	0.56	1.13	40.08	1.11	0.94	1.31	1.06	1.34	0.83	2.16	68.97
	male	1.14	0.93	1.39	0	0.98	0.80	1.21	0	1.23	0.52	2.92	74.05
	Degree	0.79	0.46	1.36	69.70	1.08	0.80	1.47	53.38	1.11	0.72	1.71	12.58
	No degree	1.00	0.84	1.19	0.00	0.96	0.80	1.15	0.00	1.46	0.86	2.49	71.78
	16-29y	0.92	0.59	1.43	45.37	0.98	0.46	2.09	73.41	3.80	2.35	6.15	*
	30-49y	0.77	0.41	1.44	59.88	1.28	0.96	1.71	0.00	1.16	0.60	2.26	45.34
	50+y	0.99	0.78	1.25	0.00	1.02	0.86	1.20	0.03	1.09	0.70	1.68	56.48
Stable unemployed	Overall	1.00	0.83	1.20	0	1.15	0.90	1.47	62.25	1.48	0.80	2.75	80.5
	female	0.90	0.70	1.17	0	1.09	0.78	1.52	56.95	1.29	0.58	2.85	80.81
	male	1.22	0.83	1.77	57.81	1.20	0.95	1.53	51.36	1.96	1.14	3.39	58.6
	Degree	1.01	0.50	2.06	82.44	0.98	0.68	1.40	55.89	1.75	1.22	2.50	0.00
	No degree	1.02	0.83	1.24	0.00	1.32	1.03	1.68	58.58	1.37	0.70	2.70	76.04
	16-29y	1.27	0.63	2.56	87.75	1.50	0.41	5.39	91.84	2.75	1.63	4.63	0.00
	30-49y	0.83	0.36	1.93	69.28	1.39	1.14	1.70	0.00	2.09	0.89	4.89	70.99
	50+y	0.71	0.38	1.33	64.94	1.12	0.93	1.34	0.00	0.98	0.53	1.80	60.57

* indicates only one study included

Table 4. Analysis of change excluding studies with ≤ 5 cell counts

		Fewer fruit and veg				More fruit and veg			
		RR	Lower CI	Upper CI	I2%	RR	Lower CI	Upper CI	I2%
Furloughed	Overall	0.96	0.87	1.08	8.96	1.22	1.04	1.43	52.46
	female	0.91	0.81	1.02	0	1.16	1.03	1.31	5.34
	male	1.03	0.90	1.19	1.11	1.21	0.98	1.50	40.11
	Degree	0.95	0.70	1.29	63.37	1.17	1.04	1.33	0
	No degree	0.97	0.87	1.09	0	1.26	0.99	1.61	62.7
	16-29y	0.75	0.38	1.47	75.61	1.18	0.91	1.52	0
	30-49y	0.88	0.76	1.03	0	1.16	0.90	1.50	42.19
	50+y	1.05	0.93	1.18	0	1.16	0.90	1.51	78.74
No longer employed	Overall	0.86	0.54	1.35	55.22	1.02	0.83	1.25	0
	female	0.88	0.68	1.14	0	1.23	1.00	1.51	0
	male	1.21	0.94	1.55		0.90	0.47	1.71	60.88
	Degree	0.72	0.30	1.75	66.85	1.17	0.91	1.51	0
	No degree	1.11	0.87	1.41	0	1.07	0.68	1.68	48.26
	16-29y	0.82	0.50	1.36	18.7	1.12	0.76	1.66	0
	30-49y	0.80	0.28	2.25	80.55	0.94	0.38	2.34	73.5
	50+y	1.00	0.79	1.27	0	1.00	0.80	1.24	0
Stable unemployed	Overall	0.86	0.34	2.20	86.92	0.98	0.56	1.70	56.16
	female	0.92	0.65	1.31	0	0.95	0.50	1.80	45.59
	male	0.81	0.42	1.55		1.22	0.63	2.38	49.82
	Degree	0.87	0.45	1.71		1.15	0.65	2.04	
	No degree	0.93	0.33	2.59	86.33	0.68	0.17	2.82	84.44
	16-29y	0.63	0.18	2.19	77.06	0.47	0.20	1.09	
	30-49y	0.47	0.23	0.96		1.88	1.32	2.70	
	50+y	1.22	0.54	2.74	80.15	1.17	0.84	1.63	0

		Less time/Fewer days of physical exercise				More time/days of physical exercise			
		RR	Lower CI	Upper CI	I2%	RR	Lower CI	Upper CI	I2%
Furloughed	Overall	1.07	0.97	1.17	38.53	1.19	1.04	1.36	75.78
	female	1.01	0.90	1.12	31.88	1.20	1.08	1.34	46.48
	male	1.16	1.04	1.29	4.72	1.18	0.98	1.43	65.02
	Degree	1.02	0.92	1.13	0	1.11	1.02	1.22	5.98
	No degree	1.10	0.99	1.21	30.03	1.22	1.05	1.42	70.82
	16-29y	1.06	0.87	1.28	0	1.19	0.74	1.91	69.22
	30-49y	1.11	0.89	1.38	59.5	1.03	0.91	1.16	0
	50+y	1.04	0.86	1.26	73.46	1.11	0.84	1.46	91.24
No longer employed	Overall	1.07	0.93	1.23	21	1.12	1.00	1.26	0.17
	female	1.14	0.91	1.43	57.54	1.00	0.85	1.16	16.71
	male	1.03	0.77	1.39	45.18	1.33	1.12	1.59	0
	Degree	0.93	0.77	1.12	0	1.06	0.92	1.24	0
	No degree	1.31	1.08	1.58	43.69	1.15	1.00	1.33	0
	16-29y	0.86	0.54	1.36	49.18	1.27	0.80	2.00	31.73
	30-49y	1.12	0.77	1.64	18.69	1.12	0.79	1.59	35.14
	50+y	1.10	0.90	1.35	39.83	1.05	0.91	1.21	0
Stable unemployed	Overall	0.84	0.65	1.09	27.66	1.14	0.96	1.35	0
	female	0.86	0.65	1.12	10.6	1.14	0.92	1.40	0
	male	0.83	0.57	1.21	0	1.35	1.00	1.83	0
	Degree	0.63	0.35	1.12	33.8	1.12	0.89	1.42	0
	No degree	0.89	0.62	1.27	57.27	1.24	1.02	1.52	0
	16-29y	0.60	0.30	1.19	0	1.30	0.72	2.37	31.48
	30-49y	1.27	0.87	1.86	*	0.60	0.34	1.08	4.48
	50+y	0.97	0.62	1.52	72.02	1.07	0.89	1.29	0

		Sleep less than before RR	Lower CI	Upper CI	I2%	Sleep more than before RR	Lower CI	Upper CI	I2%	From 6/9h a night to outside 'normal range RR	Lower CI	Upper CI	I2%	From outside 'normal range' to 6/9h a night RR	Lower CI	Upper CI	I2%
Furloughed	Overall	0.90	0.75	1.07	70.74	1.63	1.39	1.91	80.2	1.46	1.04	2.07	75.13	1.78	1.03	3.07	75.7
	female	0.91	0.80	1.02	19.62	1.54	1.32	1.80	68.21	1.36	0.89	2.09	77.96	1.63	1.07	2.50	43.73
	male	0.90	0.66	1.22	69.53	1.71	1.41	2.06	65.36	1.41	1.07	1.86	0	2.29	0.58	9.09	84.99
	Degree	0.99	0.80	1.22	51.11	1.47	1.27	1.70	50.22	1.86	0.60	5.77	93.42	1.28	0.83	1.97	0
	No degree	0.90	0.76	1.07	57.27	1.66	1.39	1.99	74.77	1.40	1.15	1.72	1.2	2.21	1.05	4.65	74.68
	16-29y	0.76	0.56	1.03	0	1.41	1.15	1.72	0	3.35	2.13	5.27	*	1.46	0.48	4.43	*
	30-49y	0.80	0.66	0.96	0	1.41	1.15	1.72	58.96	1.20	0.78	1.84	33.3	1.60	0.65	3.93	60.62
	50+y	0.85	0.65	1.12	77.53	1.61	1.17	2.23	91.74	1.31	1.05	1.63	0	1.62	0.68	3.87	87.1
No longer employed	Overall	0.99	0.85	1.16	0	1.45	1.24	1.70	39.21	1.35	0.68	2.66	72.61	1.14	0.63	2.05	0
	female	1.05	0.89	1.24	0	1.32	1.11	1.58	22.6	2.18	1.46	3.26	0	2.03	0.94	4.37	*
	male	1.04	0.74	1.48	0	1.62	1.28	2.05	36	6.38	1.89	21.51	*	no information			
	Degree	1.16	0.90	1.49	15.71	1.32	1.32	1.10	1.58	21.63	4.94	94.68	*	no information			
	No degree	0.98	0.80	1.20	0	1.56	1.50	1.25	1.80	1.90	1.13	3.18	0	1.49	0.59	3.80	
	16-29y	0.93	0.56	1.53	26.47	1.25	0.92	1.69	0	3.64	1.92	6.92	*	no information			
	30-49y	0.81	0.51	1.30	0	1.46	1.17	1.81	0	1.83	0.73	4.56	*	no information			
	50+y	0.97	0.79	1.18	0	1.49	1.21	1.83	45.91	1.44	0.73	2.84	67.03	1.59	0.79	3.22	
Stable unemployed	Overall	1.09	0.84	1.40	26.48	1.32	1.04	1.68	21.88	1.54	0.87	2.73	39.19	0.35	0.13	0.96	*
	female	1.03	0.75	1.42	26.25	1.37	1.12	1.68	0	1.74	0.82	3.67	*	0.57	0.19	1.67	*
	male	1.45	0.97	2.17	34.27	1.11	0.57	2.17	68.03	3.97	1.17	13.44	*	no information			
	Degree	1.15	0.73	1.82	0	1.34	1.25	0.84	1.84	no information				no information			
	No degree	1.20	0.93	1.55	0	1.39	1.44	1.10	1.88	2.02	1.14	3.56	0	0.35	0.12	1.02	
	16-29y	1.07	0.66	1.76	*	1.21	0.78	1.86	0	2.91	1.40	6.06	*	no information			
	30-49y	1.12	0.70	1.82	0	1.17	0.78	1.74	0	1.41	0.49	4.07	*	no information			
	50+y	0.84	0.51	1.38	51.96	1.25	0.90	1.74	26.11	1.00	0.51	1.94	0	no information			

* indicates only one study included

Table 5. Analysis of change excluding studies with ≤ 2 cell counts

		Fewer fruit and veg				More fruit and veg			
		RR	Lower CI	Upper CI	I2%	RR	Lower CI	Upper CI	I2%
Furloughed	Overall	0.96	0.87	1.08	8.96	1.22	1.04	1.43	52.46
	female	0.91	0.81	1.02	0	1.16	1.03	1.31	5.34
	male	1.03	0.90	1.19	1.11	1.21	0.98	1.50	40.11
	Degree	0.95	0.70	1.29	63.37	1.17	1.04	1.33	0
	No degree	0.97	0.87	1.09	0	1.26	0.99	1.61	62.7
	16-29y	0.75	0.38	1.47	75.61	1.18	0.91	1.52	0
	30-49y	0.88	0.76	1.03	0	1.16	0.90	1.50	42.19
	50+y	1.05	0.93	1.18	0	1.16	0.90	1.51	78.74
No longer employed	Overall	0.74	0.44	1.22	64.1	1.02	0.83	1.25	0
	female	0.84	0.65	1.08	0	1.23	1.00	1.51	0
	male	1.19	0.93	1.50	0	0.86	0.56	1.30	23.15
	Degree	0.78	0.52	1.18	25.58	1.14	0.89	1.45	0
	No degree	1.11	0.87	1.41	0	1.07	0.68	1.68	48.26
	16-29y	0.82	0.50	1.36	18.7	1.12	0.76	1.66	0
	30-49y	0.80	0.28	2.25	80.55	0.94	0.38	2.34	73.5
	50+y	0.96	0.76	1.21	0	1.00	0.80	1.24	0
Stable unemployed	Overall	0.92	0.47	1.81	76.02	1.16	0.82	1.63	21.37
	female	1.20	0.68	2.12	60.19	1.31	0.86	2.00	35.61
	male	0.91	0.53	1.57	0	1.22	0.63	2.38	49.82
	Degree	0.63	0.26	1.50	43.9	1.78	0.93	3.40	63.88
	No degree	0.93	0.33	2.59	86.33	1.07	0.53	2.16	70.2
	16-29y	0.63	0.18	2.19	77.06	0.62	0.28	1.36	14.56
	30-49y	0.65	0.29	1.44	40.92	1.84	1.34	2.54	0
	50+y	1.22	0.54	2.74	80.15	1.18	0.86	1.63	0

		Less time/Fewer days of physical exercise				More time/days of physical exercise			
		RR	Lower CI	Upper CI	I2%	RR	Lower CI	Upper CI	I2%
Furloughed	Overall	1.07	0.97	1.17	38.53	1.19	1.04	1.36	75.78
	female	1.01	0.90	1.12	31.88	1.20	1.08	1.34	46.48
	male	1.16	1.04	1.29	4.72	1.18	0.98	1.43	65.02
	Degree	1.02	0.92	1.13	0	1.11	1.02	1.22	5.98
	No degree	1.10	0.99	1.21	30.03	1.22	1.05	1.42	70.82
	16-29y	1.06	0.87	1.28	0	1.19	0.74	1.91	69.22
	30-49y	1.11	0.89	1.38	59.5	1.03	0.91	1.16	0
	50+y	1.04	0.86	1.26	73.46	1.11	0.84	1.46	91.24
No longer employed	Overall	1.07	0.93	1.23	21.00	1.12	1.00	1.26	0.17
	female	1.14	0.91	1.43	57.54	1.00	0.85	1.16	16.71
	male	0.99	0.73	1.33	46.36	1.31	1.10	1.56	0
	Degree	0.94	0.78	1.12	0	1.06	0.91	1.23	0
	No degree	1.27	1.05	1.54	43.98	1.15	1.00	1.33	0
	16-29y	0.86	0.54	1.36	49.18	1.27	0.80	2.00	31.73
	30-49y	1.12	0.77	1.64	18.69	1.12	0.79	1.59	35.14
	50+y	1.10	0.90	1.35	39.83	1.05	0.91	1.21	0
Stable unemployed	Overall	0.85	0.67	1.07	17.33	1.12	0.95	1.33	0
	female	0.91	0.70	1.19	20.28	1.09	0.89	1.33	0
	male	0.79	0.55	1.13	0	1.23	0.93	1.63	0
	Degree	0.88	0.51	1.52	59.52	1.13	0.90	1.41	0
	No degree	0.91	0.66	1.24	47.2	1.19	0.98	1.44	0
	16-29y	0.60	0.30	1.19	0	1.30	0.72	2.37	31.48
	30-49y	1.13	0.70	1.81	20.72	0.60	0.34	1.08	4.48
	50+y	0.99	0.68	1.44	59.68	1.07	0.89	1.29	0

		Sleep less than before				Sleep more than before				From 6/9h a night to outside 'normal range				From outside 'normal range' to 6/9h a night			
		RR	Lower CI	Upper CI	I2%	RR	Lower CI	Upper CI	I2%	RR	Lower CI	Upper CI	I2%	RR	Lower CI	Upper CI	I2%
Furloughed	Overall	0.90	0.75	1.07	70.74	1.63	1.39	1.91	80.2	1.46	1.04	2.07	75.13	1.78	1.03	3.07	75.7
	female	0.91	0.80	1.02	19.62	1.54	1.32	1.80	68.21	1.36	0.89	2.09	77.96	1.63	1.07	2.50	43.73
	male	0.90	0.66	1.22	69.53	1.71	1.41	2.06	65.36	1.41	1.07	1.86	0	1.60	0.64	4.01	73.78
	Degree	0.99	0.80	1.22	51.11	1.47	1.27	1.70	50.22	1.62	0.64	4.12	91.03	1.61	0.93	2.77	45.16
	No degree	0.90	0.76	1.07	57.27	1.66	1.39	1.99	74.77	1.40	1.15	1.72	1.2	2.21	1.05	4.65	74.68
	16-29y	0.76	0.56	1.03	0	1.41	1.15	1.72	0	1.67	0.36	7.75	84.08	1.02	0.45	2.32	0
	30-49y	0.80	0.66	0.96	0	1.41	1.15	1.72	58.96	1.20	0.78	1.84	33.3	1.35	0.79	2.29	22.42
	50+y	0.85	0.65	1.12	77.53	1.61	1.17	2.23	91.74	1.31	1.05	1.63	0	1.62	0.68	3.87	87.1
No longer employed	Overall	0.99	0.85	1.16	0	1.45	1.24	1.70	39.21	1.44	0.84	2.49	70.63	1.53	0.93	2.50	4.52
	female	1.05	0.89	1.24	0	1.32	1.11	1.58	22.6	1.32	0.76	2.30	59.54	2.24	1.20	4.20	0
	male	1.02	0.75	1.39	0	1.61	1.30	2.01	29.27	1.71	0.77	3.79	53.91	1.32	0.58	3.01	0
	Degree	1.17	0.94	1.46	0	1.32	1.10	1.58	15.24	1.68	0.51	5.51	79.65	1.17	0.40	3.40	0
	No degree	0.98	0.81	1.18	0	1.50	1.25	1.80	25.09	1.57	0.89	2.76	54.68	1.22	0.59	2.51	0
	16-29y	0.93	0.56	1.53	26.47	1.25	0.92	1.69	0	3.64	1.92	6.92	*	2.25	0.52	9.75	*
	30-49y	0.75	0.49	1.15	0	1.46	1.17	1.81	0	1.40	0.67	2.95	0	1.23	0.38	3.95	0
	50+y	0.97	0.79	1.18	0	1.49	1.21	1.83	45.91	1.28	0.69	2.39	61.19	1.56	0.70	3.43	47.9
Stable unemployed	Overall	1.08	0.85	1.37	21.23	1.30	1.06	1.59	5.00	1.45	0.84	2.51	33.89	1.13	0.10	12.58	85.65
	female	1.03	0.75	1.42	26.25	1.57	1.26	1.94	18.35	1.58	0.92	2.71	0	0.82	0.27	2.49	20.78
	male	1.45	0.97	2.17	34.27	1.11	0.57	2.17	68.03	1.71	0.79	3.69	23.67	no information			
	Degree	1.12	0.77	1.62	0	1.55	0.93	2.57	79.22	2.19	1.02	4.70	0	no information			
	No degree	1.14	0.90	1.44	2.16	1.44	1.10	1.88	27.39	1.66	1.02	2.69	0	1.80	0.34	9.66	83.06
	16-29y	1.25	0.78	2.01	12.63	1.21	0.78	1.86	0	2.91	1.40	6.06	*	4.15	0.90	19.23	
	30-49y	1.12	0.70	1.82	0	1.17	0.78	1.74	0	1.41	0.49	4.07	*	1.06	0.36	3.13	
	50+y	0.84	0.51	1.38	51.96	1.29	1.01	1.66	0.8	1.00	0.51	1.94	0	no information			

* indicates only one study included

Table 6. Analysis of change excluding studies with zero cell counts

		Fewer fruit and veg				More fruit and veg			
		RR	Lower CI	Upper CI	I2%	RR	Lower CI	Upper CI	I2%
Furloughed	Overall	0.96	0.87	1.08	8.96	1.22	1.04	1.43	52.46
	female	0.91	0.81	1.02	0	1.16	1.03	1.31	5.34
	male	1.03	0.90	1.19	1.11	1.21	0.98	1.50	40.11
	Degree	0.95	0.70	1.29	63.37	1.17	1.04	1.33	0
	No degree	0.97	0.87	1.09	0	1.26	0.99	1.61	62.7
	16-29y	0.75	0.38	1.47	75.61	1.18	0.91	1.52	0
	30-49y	0.88	0.76	1.03	0	1.16	0.90	1.50	42.19
	50+y	1.05	0.93	1.18	0	1.16	0.90	1.51	78.74
No longer employed	Overall	0.74	0.44	1.22	64.1	1.02	0.83	1.25	0
	female	0.78	0.56	1.10	14.94	1.23	1.00	1.51	0
	male	1.16	0.91	1.47	0	0.85	0.58	1.24	13.66
	Degree	0.78	0.52	1.18	25.58	1.14	0.89	1.45	0
	No degree	1.05	0.83	1.34	0	1.07	0.68	1.68	48.26
	16-29y	0.82	0.50	1.36	18.7	1.12	0.76	1.66	0
	30-49y	0.80	0.28	2.25	80.55	0.94	0.38	2.34	73.5
	50+y	0.96	0.76	1.21	0	1.00	0.80	1.24	0
Stable unemployed	Overall	0.85	0.45	1.59	71.11	1.16	0.82	1.63	21.37
	female	1.06	0.66	1.70	42.23	1.31	0.86	2.00	35.61
	male	0.87	0.38	1.99	57.38	1.19	0.69	2.05	24.75
	Degree	0.72	0.44	1.19	0.08	1.78	0.93	3.40	63.88
	No degree	0.87	0.42	1.82	70.36	1.07	0.53	2.16	70.2
	16-29y	0.63	0.18	2.19	77.06	0.62	0.28	1.36	14.56
	30-49y	0.65	0.29	1.44	40.92	1.84	1.34	2.54	0
	50+y	1.02	0.48	2.19	71.06	1.18	0.86	1.63	0

		**Less time/Fewer days of physical exercise**				**More time/days of physical exercise**			
		RR	Lower CI	Upper CI	I2%	RR	Lower CI	Upper CI	I2%
Furloughed	Overall	1.07	0.97	1.17	38.53	1.19	1.04	1.36	75.78
	female	1.01	0.90	1.12	31.88	1.20	1.08	1.34	46.48
	male	1.16	1.04	1.29	4.72	1.18	0.98	1.43	65.02
	Degree	1.02	0.92	1.13	0	1.11	1.02	1.22	5.98
	No degree	1.10	0.99	1.21	30.03	1.22	1.05	1.42	70.82
	16-29y	1.06	0.87	1.28	0	1.19	0.74	1.91	69.22
	30-49y	1.11	0.89	1.38	59.5	1.03	0.91	1.16	0
	50+y	1.04	0.86	1.26	73.46	1.11	0.84	1.46	91.24
No longer employed	Overall	1.07	0.93	1.23	21.00	1.12	1.00	1.26	0.17
	female	1.14	0.91	1.43	57.54	1.00	0.85	1.16	16.71
	male	0.99	0.73	1.33	46.36	1.31	1.10	1.56	0
	Degree	0.93	0.78	1.11	0	1.06	0.91	1.23	0
	No degree	1.27	1.05	1.54	43.98	1.15	1.00	1.33	0
	16-29y	0.86	0.54	1.36	49.18	1.27	0.80	2.00	31.73
	30-49y	1.12	0.77	1.64	18.69	1.12	0.79	1.59	35.14
	50+y	1.10	0.90	1.35	39.83	1.09	0.95	1.24	0
Stable unemployed	Overall	0.83	0.66	1.05	15.14	1.11	0.94	1.32	0.47
	female	0.92	0.71	1.19	16.21	1.09	0.89	1.32	0
	male	0.81	0.58	1.13	0	1.18	0.90	1.55	0
	Degree	0.88	0.58	1.33	34.49	1.12	0.91	1.39	0
	No degree	0.91	0.66	1.24	47.2	1.17	0.97	1.43	0
	16-29y	0.60	0.30	1.19	0	1.30	0.72	2.37	31.48
	30-49y	1.13	0.70	1.81	20.72	0.60	0.34	1.08	4.48
	50+y	0.94	0.65	1.37	52.73	1.06	0.88	1.28	0

		Sleep less than before				Sleep more than before				From 6/9h a night to outside 'normal range				From outside 'normal range' to 6/9h a night			
		RR	Lower CI	Upper CI	I2%	RR	Lower CI	Upper CI	I2%	RR	Lower CI	Upper CI	I2%	RR	Lower CI	Upper CI	I2%
Furloughed	Overall	0.90	0.75	1.07	70.74	1.63	1.39	1.91	80.2	1.46	1.04	2.07	75.13	1.78	1.03	3.07	75.7
	female	0.91	0.80	1.02	19.62	1.54	1.32	1.80	68.21	1.36	0.89	2.09	77.96	1.63	1.07	2.50	43.73
	male	0.90	0.66	1.22	69.53	1.71	1.41	2.06	65.36	1.41	1.07	1.86	0	1.57	0.70	3.55	66.38
	Degree	0.99	0.80	1.22	51.11	1.47	1.27	1.70	50.22	1.62	0.64	4.12	91.03	1.36	0.79	2.33	39.8
	No degree	0.90	0.76	1.07	57.27	1.66	1.39	1.99	74.77	1.40	1.15	1.72	1.2	2.21	1.05	4.65	74.68
	16-29y	0.76	0.56	1.03	0	1.41	1.15	1.72	0	1.67	0.36	7.75	84.08	1.02	0.45	2.32	0
	30-49y	0.80	0.66	0.96	0	1.41	1.15	1.72	58.96	1.20	0.78	1.84	33.3	1.35	0.79	2.29	22.42
	50+y	0.85	0.65	1.12	77.53	1.61	1.17	2.23	91.74	1.31	1.05	1.63	0	1.62	0.68	3.87	87.1
No longer employed	Overall	0.99	0.85	1.16	0	1.45	1.24	1.70	39.21	1.44	0.84	2.49	70.63	1.49	0.93	2.37	0
	female	1.05	0.89	1.24	0	1.32	1.11	1.58	22.6	1.32	0.76	2.30	59.54	1.93	1.14	3.26	0
	male	0.96	0.71	1.30	0	1.61	1.30	2.01	29.27	1.71	0.77	3.79	53.91	1.51	0.74	3.07	0
	Degree	1.17	0.94	1.46	0	1.30	1.08	1.56	14.39	1.54	0.59	4.00	74.01	1.25	0.61	2.58	0
	No degree	0.98	0.81	1.18	0	1.50	1.25	1.80	25.09	1.57	0.89	2.76	54.68	2.45	1.08	5.57	41.89
	16-29y	0.93	0.56	1.53	26.47	1.25	0.92	1.69	0	3.64	1.92	6.92	*	1.78	0.59	5.39	0
	30-49y	0.75	0.49	1.15	0	1.46	1.17	1.81	0	1.16	0.58	2.30	8.35	1.58	0.65	3.82	0
	50+y	0.97	0.79	1.18	0	1.49	1.21	1.83	45.91	1.28	0.69	2.39	61.19	1.50	0.78	2.89	27.27
Stable unemployed	Overall	1.03	0.79	1.33	28.94	1.30	1.06	1.59	5.00	1.23	0.68	2.25	43.72	2.58	0.82	8.11	77.94
	female	0.90	0.64	1.27	36.82	1.54	1.26	1.88	9.86	1.26	0.77	2.09	0	2.85	0.87	9.37	72.45
	male	1.37	0.91	2.05	35	1.04	0.64	1.69	47.94	1.65	0.99	2.76	0	4.03	1.27	12.83	0
	Degree	1.05	0.74	1.51	0	1.52	0.96	2.40	72.88	1.69	0.70	4.08	48.4	2.18	0.26	18.02	*
	No degree	1.14	0.90	1.44	2.16	1.36	1.03	1.78	27.99	1.66	1.02	2.69	0	3.41	0.84	13.83	78.46
	16-29y	1.25	0.78	2.01	12.63	1.21	0.78	1.86	0	2.84	1.42	5.67	0	4.15	0.90	19.23	*
	30-49y	1.53	0.80	2.90	68.07	1.17	0.78	1.74	0	1.34	0.60	2.98	0	3.77	0.81	17.48	74.69
	50+y	0.72	0.43	1.19	50.84	1.29	1.01	1.66	0.8	0.87	0.48	1.58	0	1.79	0.29	11.03	82.46

* indicates only one study included

Figure set 1: Currently eats 2 or fewer fruit & veg

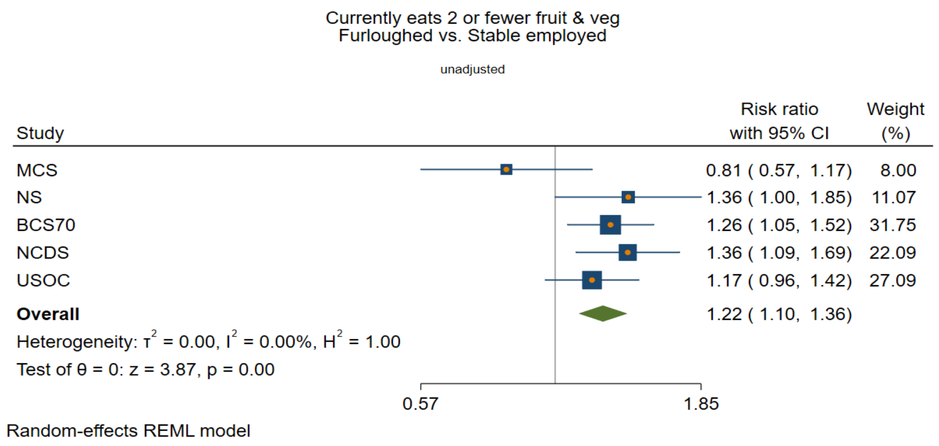

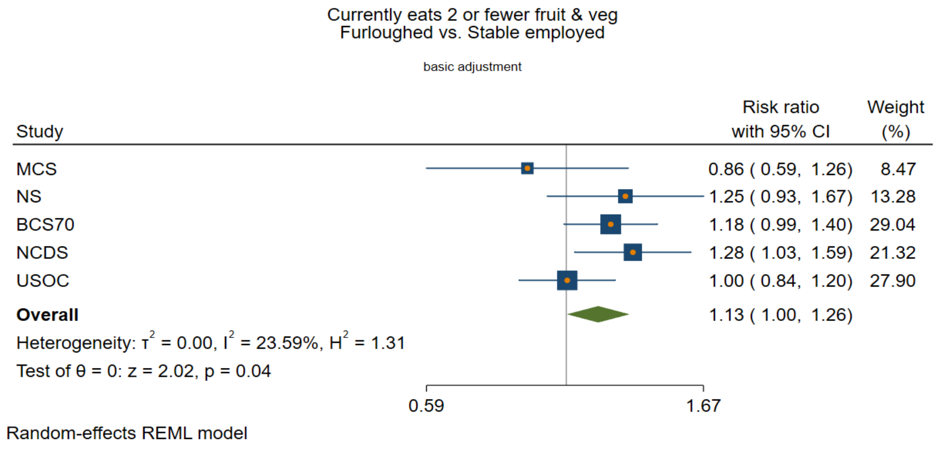

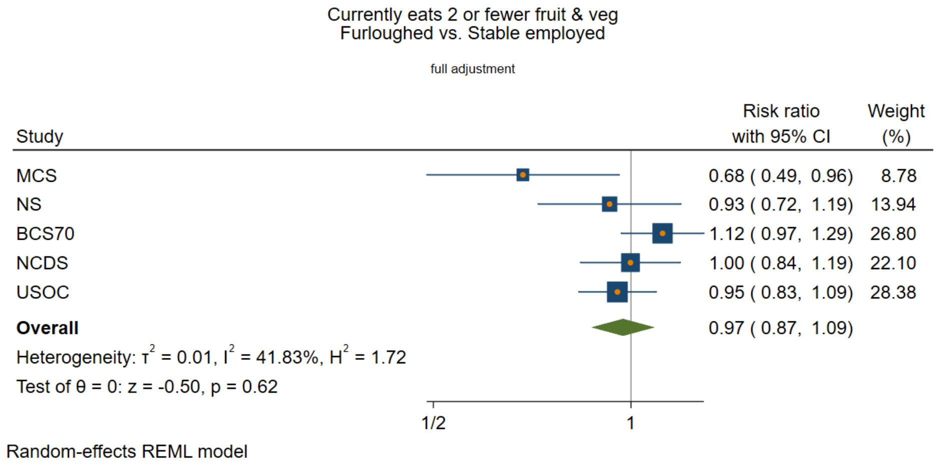

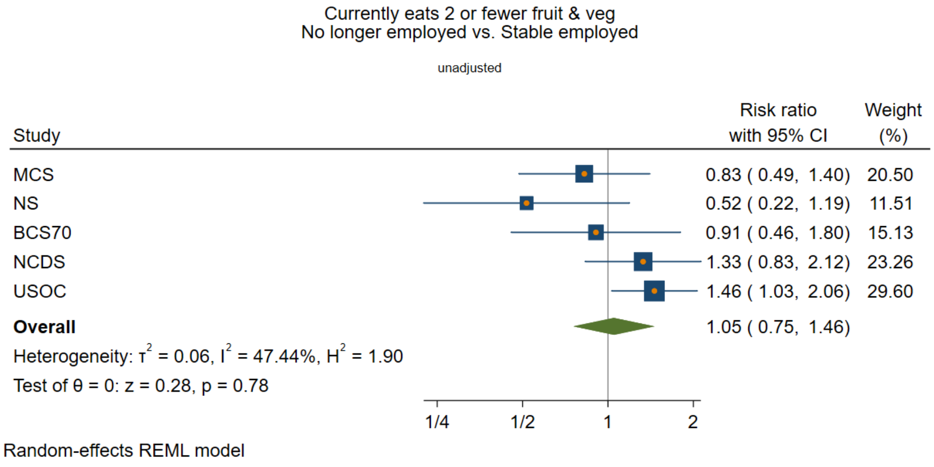
Currently eats 2 or fewer fruit & veg
No longer employed vs. Stable employed
unadjusted
Study
Risk ratio with 95% CI
Weight (%)
MCS 0.83 (0.49, 1.40) 20.50
NS 0.52 (0.22, 1.19) 11.51
BCS70 0.91 (0.46, 1.80) 15.13
NCDS 1.33 (0.83, 2.12) 23.26
USOC 1.46 (1.03, 2.06) 29.60
Overall 1.05 (0.75, 1.46)
Heterogeneity: τ² = 0.06, I² = 47.44%, H² = 1.90
Test of θ = 0: z = 0.28, p = 0.78
1/4
1/2
1
2
Random-effects REML model

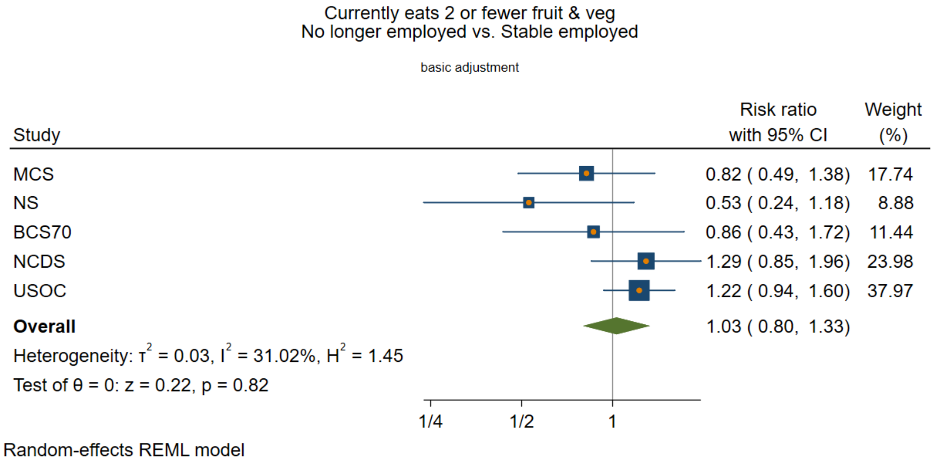
Currently eats 2 or fewer fruit & veg
No longer employed vs. Stable employed
basic adjustment
Study
Risk ratio with 95% CI
Weight (%)
MCS 0.82 (0.49, 1.38) 17.74
NS 0.53 (0.24, 1.18) 8.88
BCS70 0.86 (0.43, 1.72) 11.44
NCDS 1.29 (0.85, 1.96) 23.98
USOC 1.22 (0.94, 1.60) 37.97
Overall 1.03 (0.80, 1.33)
Heterogeneity: τ² = 0.03, I² = 31.02%, H² = 1.45
Test of θ = 0: z = 0.22, p = 0.82
1/4
1/2
1
Random-effects REML model

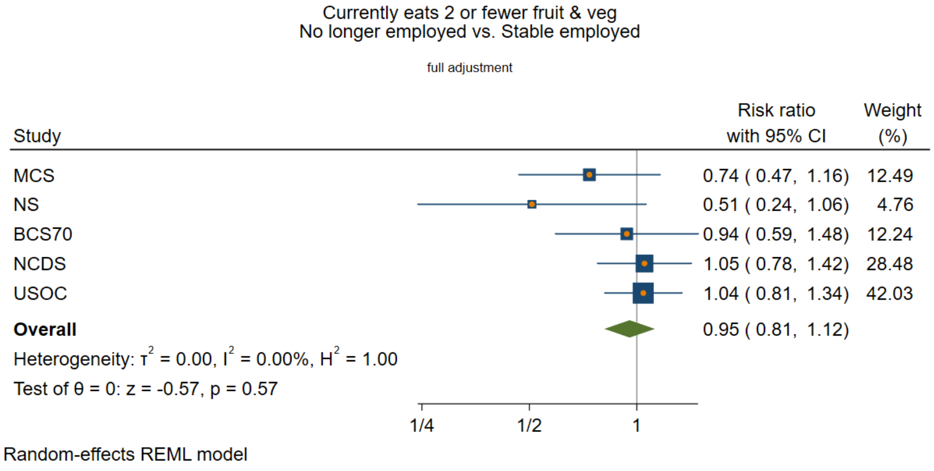
Currently eats 2 or fewer fruit & veg
No longer employed vs. Stable employed
full adjustment
Study
Risk ratio with 95% CI
Weight (%)
MCS 0.74 (0.47, 1.16) 12.49
NS 0.51 (0.24, 1.06) 4.76
BCS70 0.94 (0.59, 1.48) 12.24
NCDS 1.05 (0.78, 1.42) 28.48
USOC 1.04 (0.81, 1.34) 42.03
Overall 0.95 (0.81, 1.12)
Heterogeneity: τ² = 0.00, I² = 0.00%, H² = 1.00
Test of θ = 0: z = -0.57, p = 0.57
1/4
1/2
1
Random-effects REML model

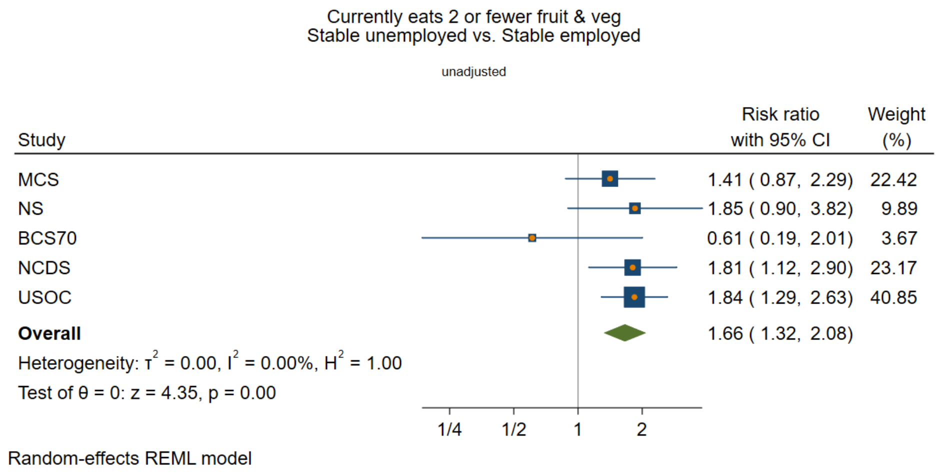

Currently eats 2 or fewer fruit & veg
Stable unemployed vs. Stable employed
unadjusted
Study
Risk ratio with 95% CI
Weight (%)
MCS 1.41 (0.87, 2.29) 22.42
NS 1.85 (0.90, 3.82) 9.89
BCS70 0.61 (0.19, 2.01) 3.67
NCDS 1.81 (1.12, 2.90) 23.17
USOC 1.84 (1.29, 2.63) 40.85
Overall 1.66 (1.32, 2.08)
Heterogeneity: τ^2 = 0.00, I^2 = 0.00%, H^2 = 1.00
Test of θ = 0: z = 4.35, p = 0.00
1/4
1/2
1
2
Random-effects REML model

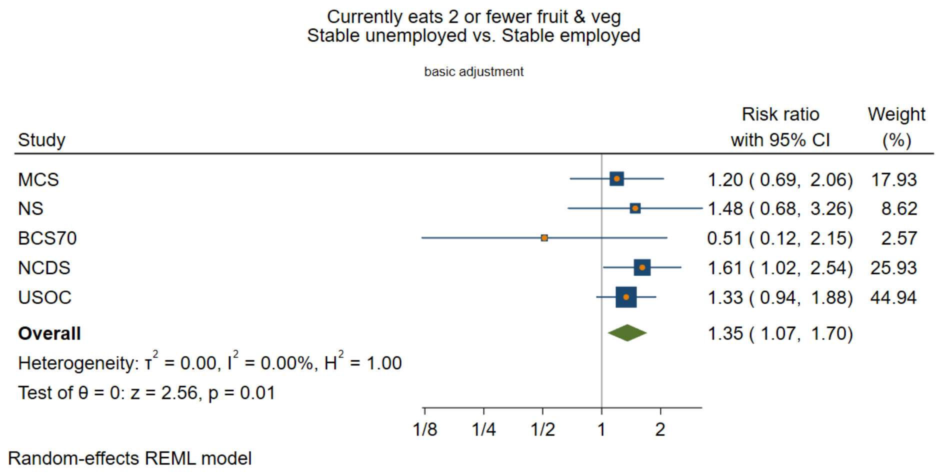

Currently eats 2 or fewer fruit & veg
Stable unemployed vs. Stable employed
basic adjustment
Study
Risk ratio with 95% CI
Weight (%)
MCS 1.20 (0.69, 2.06) 17.93
NS 1.48 (0.68, 3.26) 8.62
BCS70 0.51 (0.12, 2.15) 2.57
NCDS 1.61 (1.02, 2.54) 25.93
USOC 1.33 (0.94, 1.88) 44.94
Overall 1.35 (1.07, 1.70)
Heterogeneity: τ^2 = 0.00, I^2 = 0.00%, H^2 = 1.00
Test of θ = 0: z = 2.56, p = 0.01
1/8
1/4
1/2
1
2
Random-effects REML model

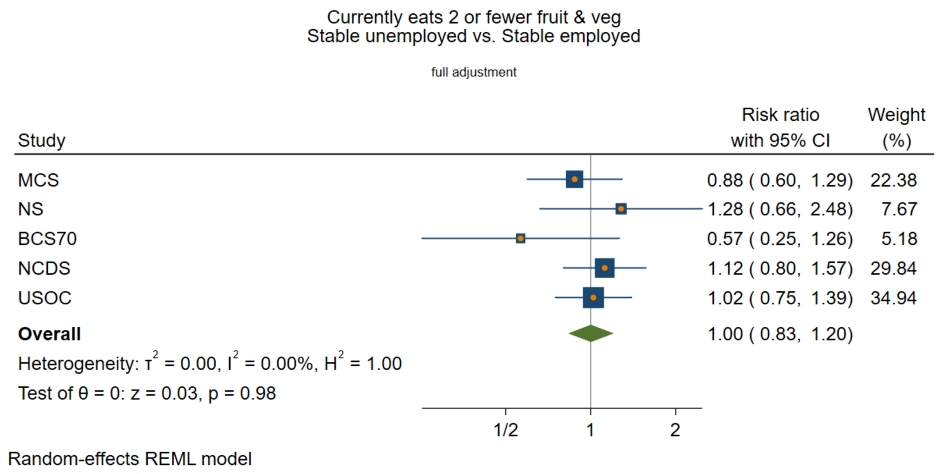

Currently eats 2 or fewer fruit & veg
Stable unemployed vs. Stable employed
full adjustment
Study
Risk ratio with 95% CI
Weight (%)
MCS 0.88 (0.60, 1.29) 22.38
NS 1.28 (0.66, 2.48) 7.67
BCS70 0.57 (0.25, 1.26) 5.18
NCDS 1.12 (0.80, 1.57) 29.84
USOC 1.02 (0.75, 1.39) 34.94
Overall 1.00 (0.83, 1.20)
Heterogeneity: τ^2 = 0.00, I^2 = 0.00%, H^2 = 1.00
Test of θ = 0: z = 0.03, p = 0.98
1/2
1
2
Random-effects REML model

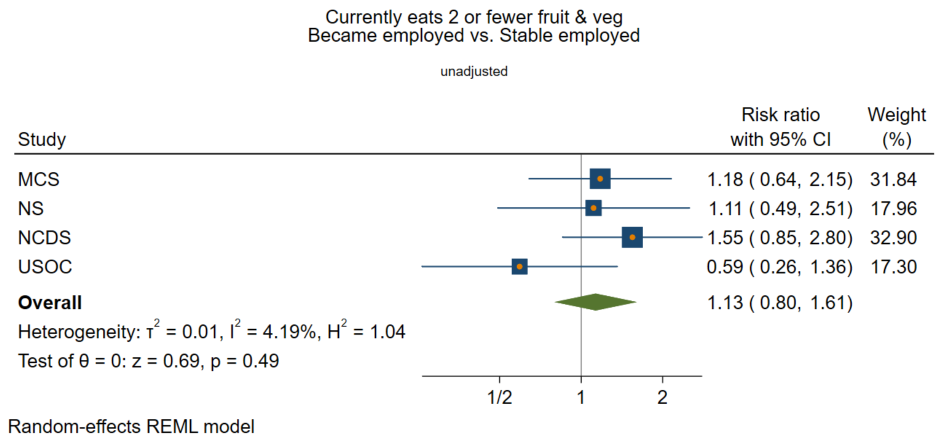
Currently eats 2 or fewer fruit & veg
Became employed vs. Stable employed
unadjusted
Study
Risk ratio with 95% CI
Weight (%)
MCS 1.18 (0.64, 2.15) 31.84
NS 1.11 (0.49, 2.51) 17.96
NCDS 1.55 (0.85, 2.80) 32.90
USOC 0.59 (0.26, 1.36) 17.30
Overall 1.13 (0.80, 1.61)
Heterogeneity: τ² = 0.01, I² = 4.19%, H² = 1.04
Test of θ = 0: z = 0.69, p = 0.49
1/2
1
2
Random-effects REML model

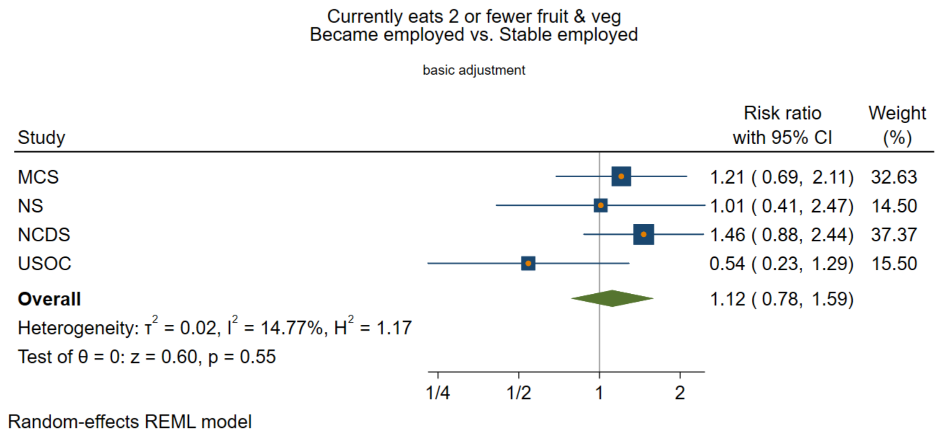
Currently eats 2 or fewer fruit & veg
Became employed vs. Stable employed
basic adjustment
Study
Risk ratio with 95% CI
Weight (%)
MCS 1.21 (0.69, 2.11) 32.63
NS 1.01 (0.41, 2.47) 14.50
NCDS 1.46 (0.88, 2.44) 37.37
USOC 0.54 (0.23, 1.29) 15.50
Overall 1.12 (0.78, 1.59)
Heterogeneity: τ² = 0.02, I² = 14.77%, H² = 1.17
Test of θ = 0: z = 0.60, p = 0.55
1/4
1/2
1
2
Random-effects REML model

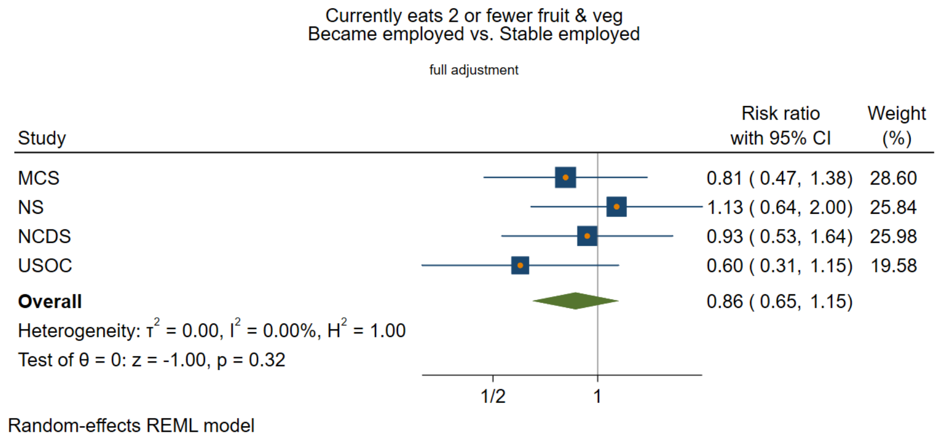
Currently eats 2 or fewer fruit & veg
Became employed vs. Stable employed
full adjustment
Study
Risk ratio with 95% CI
Weight (%)
MCS 0.81 (0.47, 1.38) 28.60
NS 1.13 (0.64, 2.00) 25.84
NCDS 0.93 (0.53, 1.64) 25.98
USOC 0.60 (0.31, 1.15) 19.58
Overall 0.86 (0.65, 1.15)
Heterogeneity: τ² = 0.00, I² = 0.00%, H² = 1.00
Test of θ = 0: z = -1.00, p = 0.32
1/2
1
Random-effects REML model

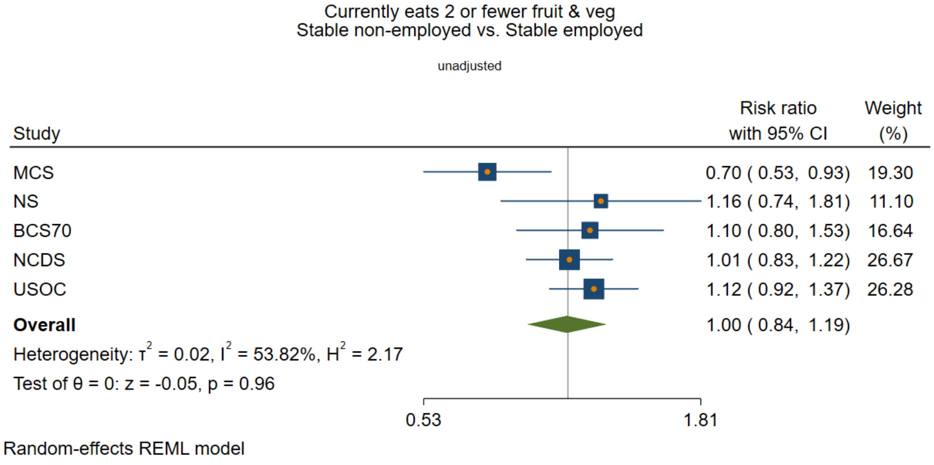
Currently eats 2 or fewer fruit & veg
Stable non-employed vs. Stable employed
unadjusted
Study
Risk ratio with 95% CI
Weight (%)
MCS 0.70 (0.53, 0.93) 19.30
NS 1.16 (0.74, 1.81) 11.10
BCS70 1.10 (0.80, 1.53) 16.64
NCDS 1.01 (0.83, 1.22) 26.67
USOC 1.12 (0.92, 1.37) 26.28
Overall 1.00 (0.84, 1.19)
Heterogeneity: τ² = 0.02, I² = 53.82%, H² = 2.17
Test of θ = 0: z = -0.05, p = 0.96
0.53
1.81
Random-effects REML model

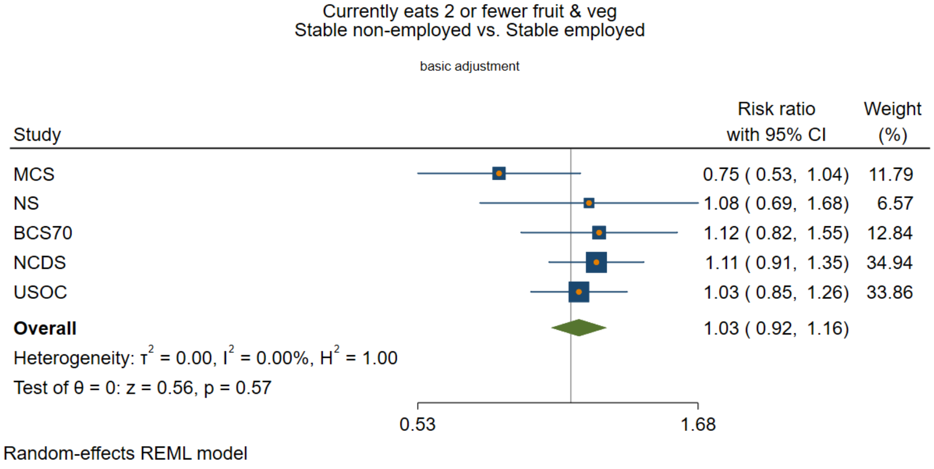
Currently eats 2 or fewer fruit & veg
Stable non-employed vs. Stable employed
basic adjustment
Study
Risk ratio with 95% CI
Weight (%)
MCS 0.75 (0.53, 1.04) 11.79
NS 1.08 (0.69, 1.68) 6.57
BCS70 1.12 (0.82, 1.55) 12.84
NCDS 1.11 (0.91, 1.35) 34.94
USOC 1.03 (0.85, 1.26) 33.86
Overall 1.03 (0.92, 1.16)
Heterogeneity: τ² = 0.00, I² = 0.00%, H² = 1.00
Test of θ = 0: z = 0.56, p = 0.57
0.53
1.68
Random-effects REML model

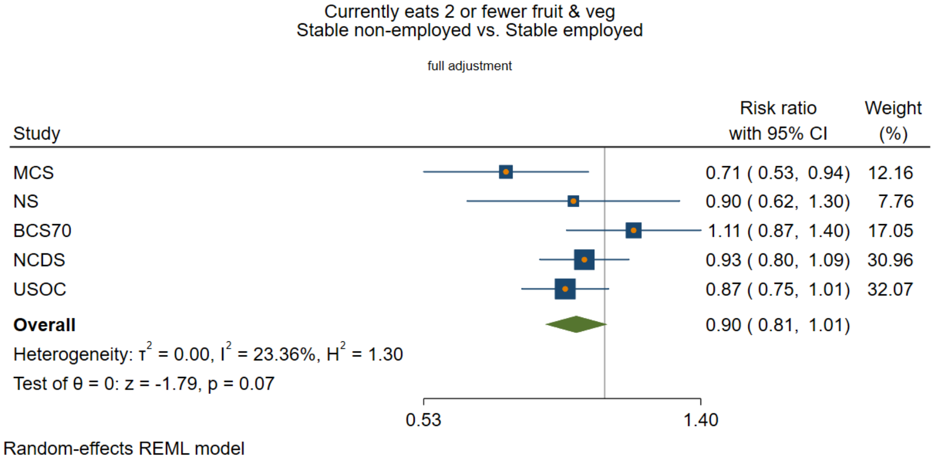
Currently eats 2 or fewer fruit & veg
Stable non-employed vs. Stable employed
full adjustment
Study
Risk ratio with 95% CI
Weight (%)
MCS 0.71 (0.53, 0.94) 12.16
NS 0.90 (0.62, 1.30) 7.76
BCS70 1.11 (0.87, 1.40) 17.05
NCDS 0.93 (0.80, 1.09) 30.96
USOC 0.87 (0.75, 1.01) 32.07
Overall 0.90 (0.81, 1.01)
Heterogeneity: τ² = 0.00, I² = 23.36%, H² = 1.30
Test of θ = 0: z = -1.79, p = 0.07
0.53
1.40
Random-effects REML model

Figure set 2: Fewer fruit and vegetables

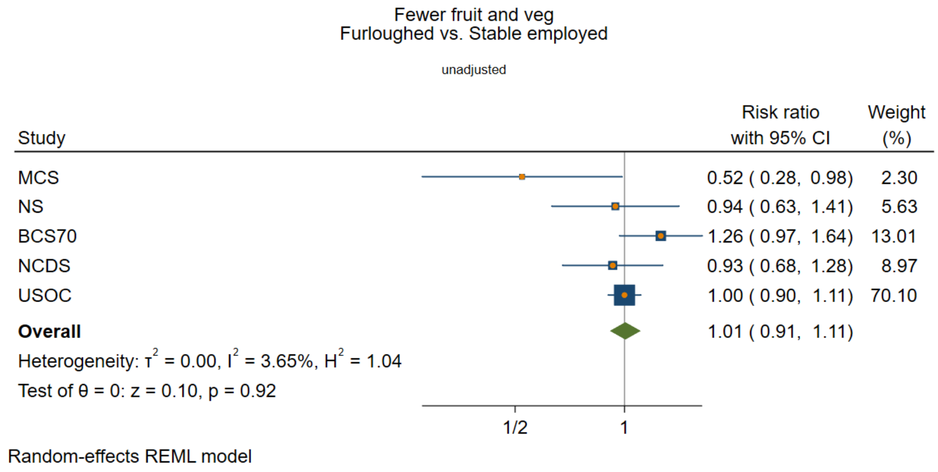

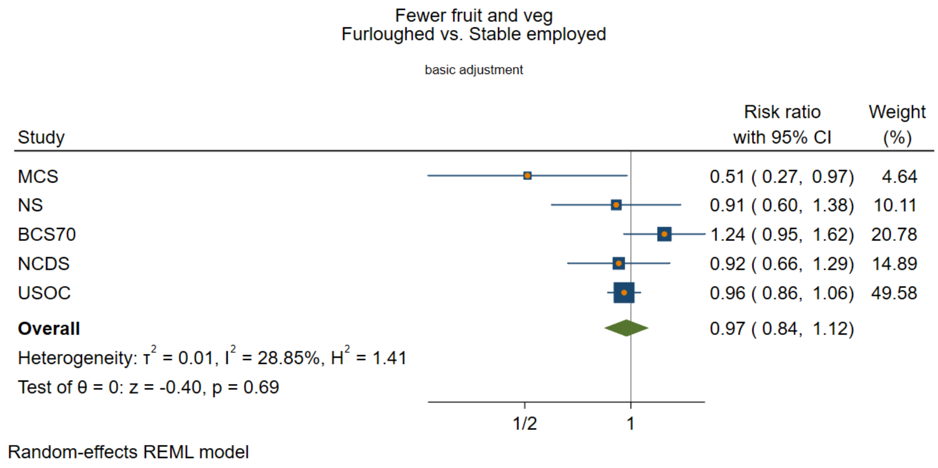

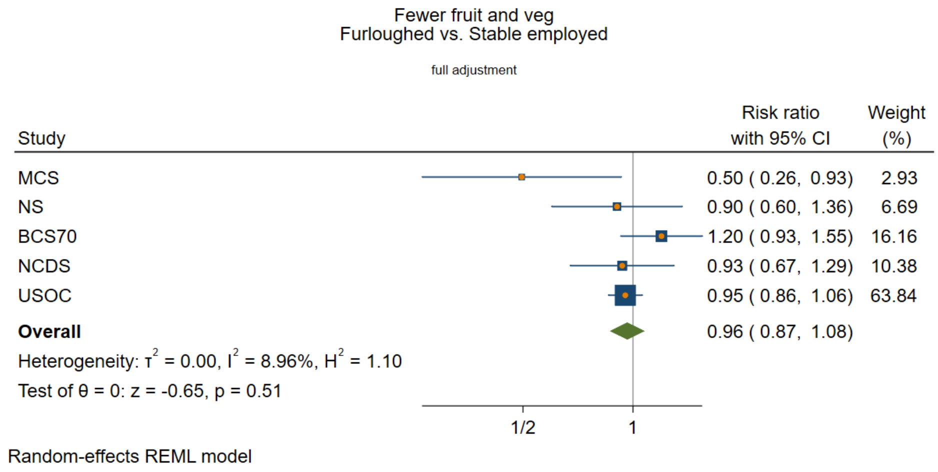

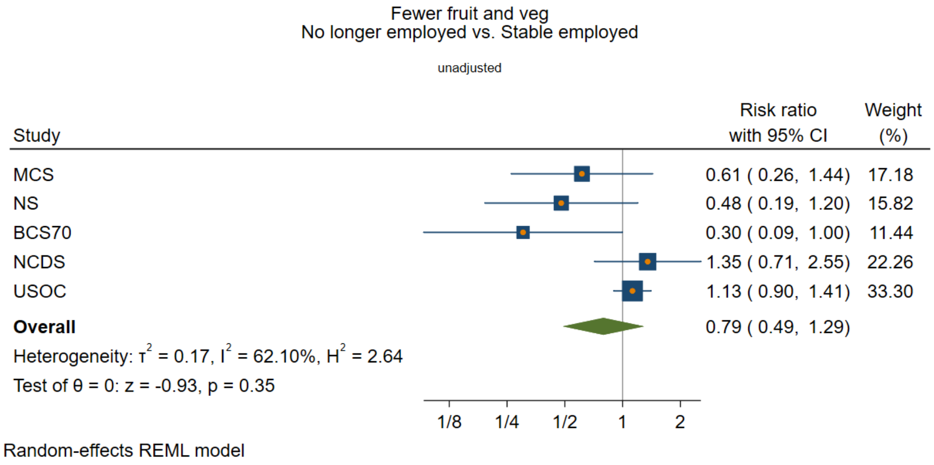
Fewer fruit and veg
No longer employed vs. Stable employed
unadjusted
Study
Risk ratio with 95% CI
Weight (%)
MCS 0.61 (0.26, 1.44) 17.18
NS 0.48 (0.19, 1.20) 15.82
BCS70 0.30 (0.09, 1.00) 11.44
NCDS 1.35 (0.71, 2.55) 22.26
USOC 1.13 (0.90, 1.41) 33.30
Overall 0.79 (0.49, 1.29)
Heterogeneity: τ² = 0.17, I² = 62.10%, H² = 2.64
Test of θ = 0: z = -0.93, p = 0.35
1/8 1/4 1/2 1 2
Random-effects REML model

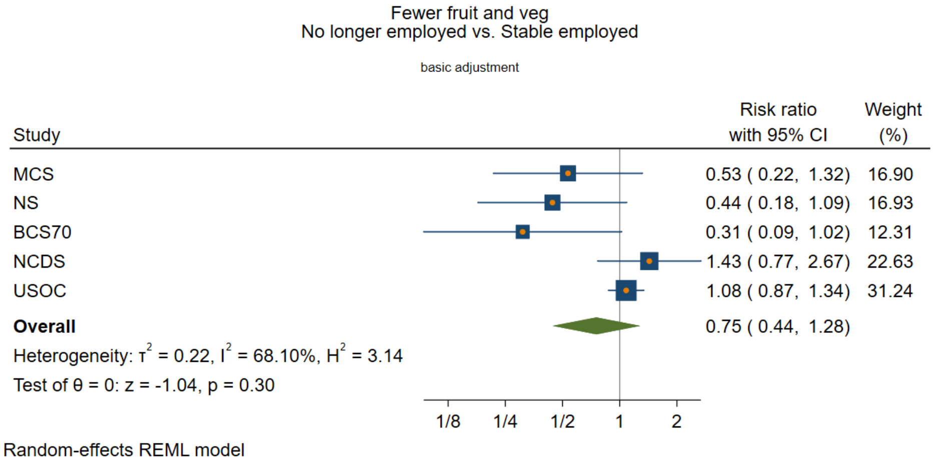
Fewer fruit and veg
No longer employed vs. Stable employed
basic adjustment
Study
Risk ratio with 95% CI
Weight (%)
MCS 0.53 (0.22, 1.32) 16.90
NS 0.44 (0.18, 1.09) 16.93
BCS70 0.31 (0.09, 1.02) 12.31
NCDS 1.43 (0.77, 2.67) 22.63
USOC 1.08 (0.87, 1.34) 31.24
Overall 0.75 (0.44, 1.28)
Heterogeneity: τ² = 0.22, I² = 68.10%, H² = 3.14
Test of θ = 0: z = -1.04, p = 0.30
1/8 1/4 1/2 1 2
Random-effects REML model

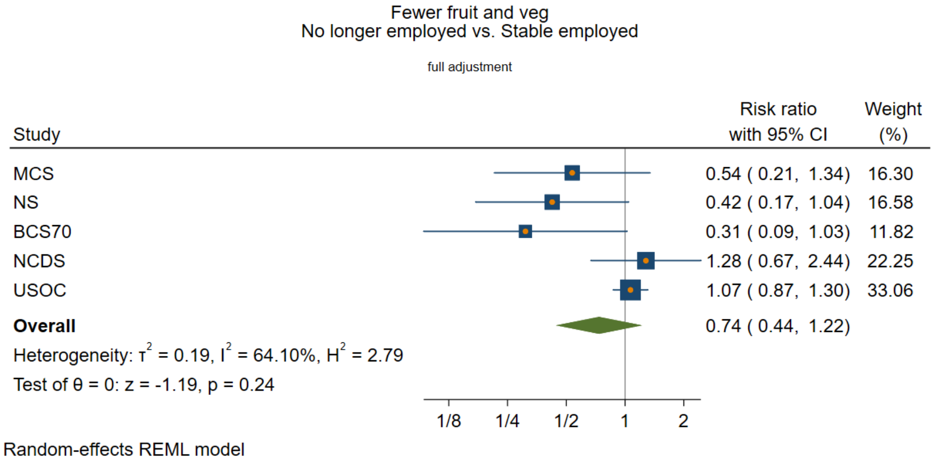
Fewer fruit and veg
No longer employed vs. Stable employed
full adjustment
Study
Risk ratio with 95% CI
Weight (%)
MCS 0.54 (0.21, 1.34) 16.30
NS 0.42 (0.17, 1.04) 16.58
BCS70 0.31 (0.09, 1.03) 11.82
NCDS 1.28 (0.67, 2.44) 22.25
USOC 1.07 (0.87, 1.30) 33.06
Overall 0.74 (0.44, 1.22)
Heterogeneity: τ² = 0.19, I² = 64.10%, H² = 2.79
Test of θ = 0: z = -1.19, p = 0.24
1/8 1/4 1/2 1 2
Random-effects REML model

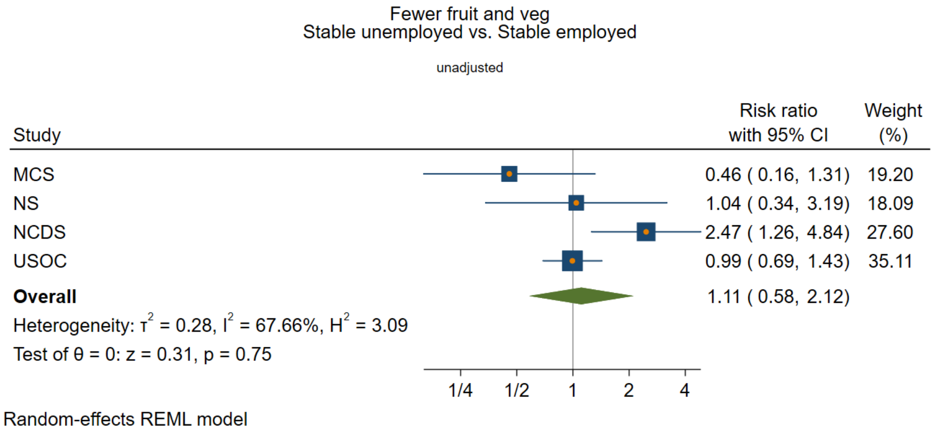
Fewer fruit and veg
Stable unemployed vs. Stable employed
unadjusted
Study
Risk ratio with 95% CI
Weight (%)
MCS 0.46 (0.16, 1.31) 19.20
NS 1.04 (0.34, 3.19) 18.09
NCDS 2.47 (1.26, 4.84) 27.60
USOC 0.99 (0.69, 1.43) 35.11
Overall 1.11 (0.58, 2.12)
Heterogeneity: τ^2 = 0.28, I^2 = 67.66%, H^2 = 3.09
Test of θ = 0: z = 0.31, p = 0.75
1/4
1/2
1
2
4
Random-effects REML model

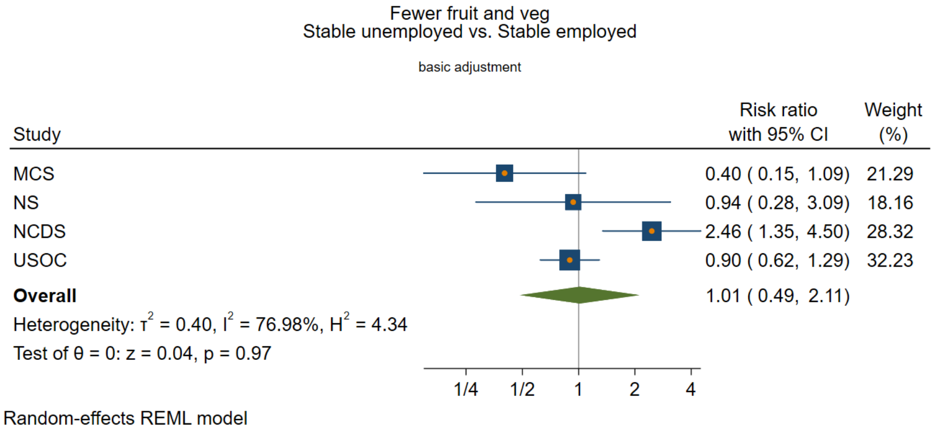
Fewer fruit and veg
Stable unemployed vs. Stable employed
basic adjustment
Study
Risk ratio with 95% CI
Weight (%)
MCS 0.40 (0.15, 1.09) 21.29
NS 0.94 (0.28, 3.09) 18.16
NCDS 2.46 (1.35, 4.50) 28.32
USOC 0.90 (0.62, 1.29) 32.23
Overall 1.01 (0.49, 2.11)
Heterogeneity: τ^2 = 0.40, I^2 = 76.98%, H^2 = 4.34
Test of θ = 0: z = 0.04, p = 0.97
1/4
1/2
1
2
4
Random-effects REML model

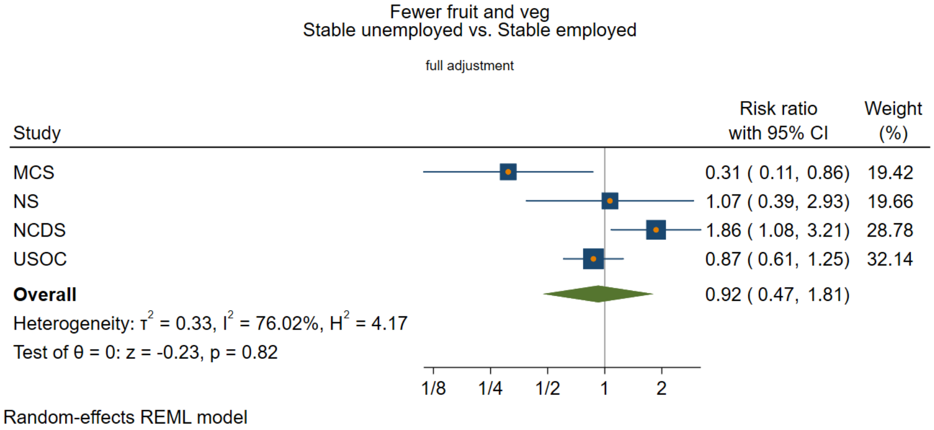
Fewer fruit and veg
Stable unemployed vs. Stable employed
full adjustment
Study
Risk ratio with 95% CI
Weight (%)
MCS 0.31 (0.11, 0.86) 19.42
NS 1.07 (0.39, 2.93) 19.66
NCDS 1.86 (1.08, 3.21) 28.78
USOC 0.87 (0.61, 1.25) 32.14
Overall 0.92 (0.47, 1.81)
Heterogeneity: τ^2 = 0.33, I^2 = 76.02%, H^2 = 4.17
Test of θ = 0: z = -0.23, p = 0.82
1/8
1/4
1/2
1
2
Random-effects REML model

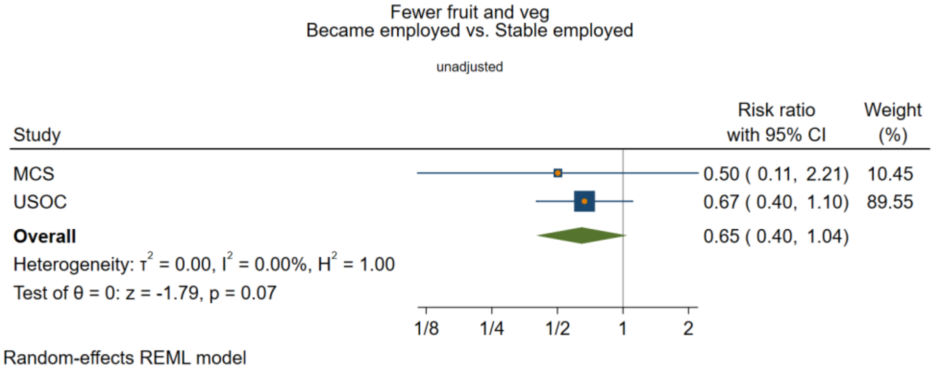
Fewer fruit and veg
Became employed vs. Stable employed
unadjusted
Study
Risk ratio with 95% CI
Weight (%)
MCS
0.50 (0.11, 2.21)
10.45
USOC
0.67 (0.40, 1.10)
89.55
Overall
0.65 (0.40, 1.04)
Heterogeneity: τ² = 0.00, I² = 0.00%, H² = 1.00
Test of θ = 0: z = -1.79, p = 0.07
1/8
1/4
1/2
1
2
Random-effects REML model

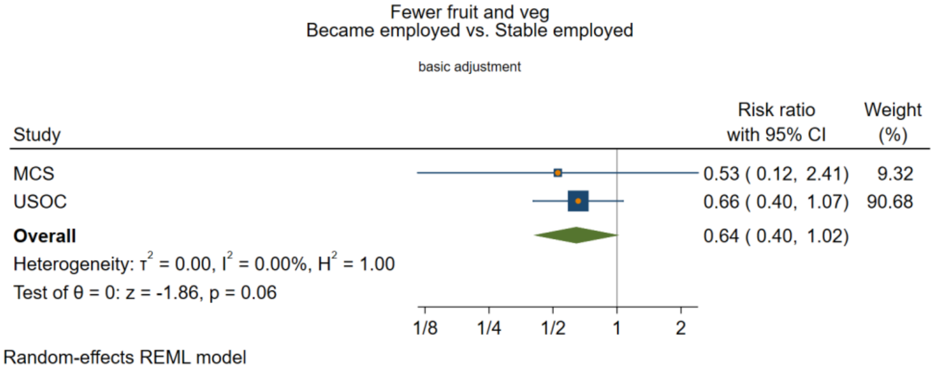
Fewer fruit and veg
Became employed vs. Stable employed
basic adjustment
Study
Risk ratio with 95% CI
Weight (%)
MCS
0.53 (0.12, 2.41)
9.32
USOC
0.66 (0.40, 1.07)
90.68
Overall
0.64 (0.40, 1.02)
Heterogeneity: τ² = 0.00, I² = 0.00%, H² = 1.00
Test of θ = 0: z = -1.86, p = 0.06
1/8
1/4
1/2
1
2
Random-effects REML model

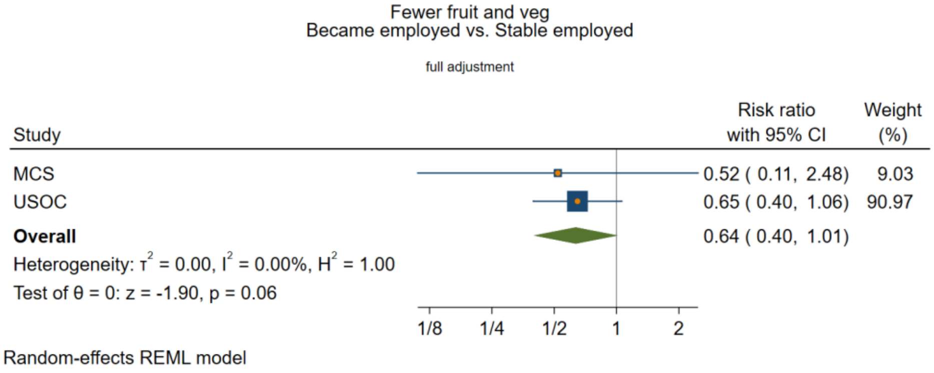
Fewer fruit and veg
Became employed vs. Stable employed
full adjustment
Study
Risk ratio with 95% CI
Weight (%)
MCS
0.52 (0.11, 2.48)
9.03
USOC
0.65 (0.40, 1.06)
90.97
Overall
0.64 (0.40, 1.01)
Heterogeneity: τ² = 0.00, I² = 0.00%, H² = 1.00
Test of θ = 0: z = -1.90, p = 0.06
1/8
1/4
1/2
1
2
Random-effects REML model

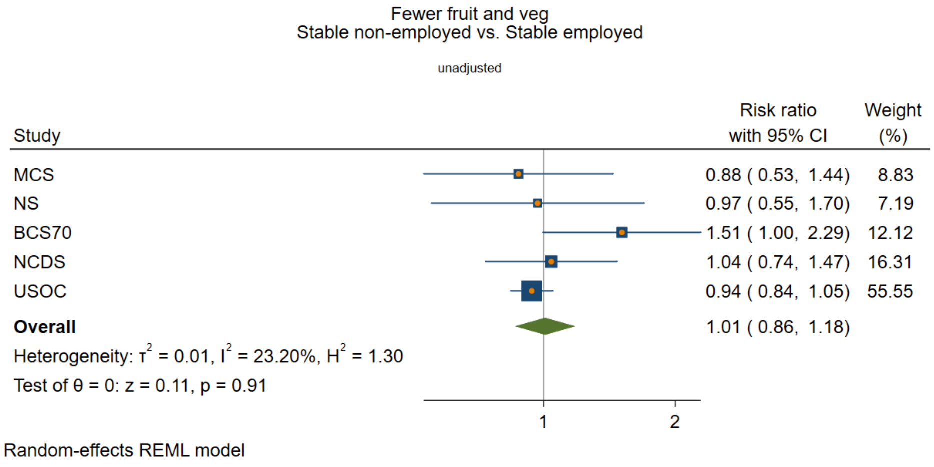
Fewer fruit and veg
Stable non-employed vs. Stable employed
unadjusted
Study
Risk ratio with 95% CI
Weight (%)
MCS 0.88 (0.53, 1.44) 8.83
NS 0.97 (0.55, 1.70) 7.19
BCS70 1.51 (1.00, 2.29) 12.12
NCDS 1.04 (0.74, 1.47) 16.31
USOC 0.94 (0.84, 1.05) 55.55
Overall 1.01 (0.86, 1.18)
Heterogeneity: τ² = 0.01, I² = 23.20%, H² = 1.30
Test of θ = 0: z = 0.11, p = 0.91
1
2
Random-effects REML model

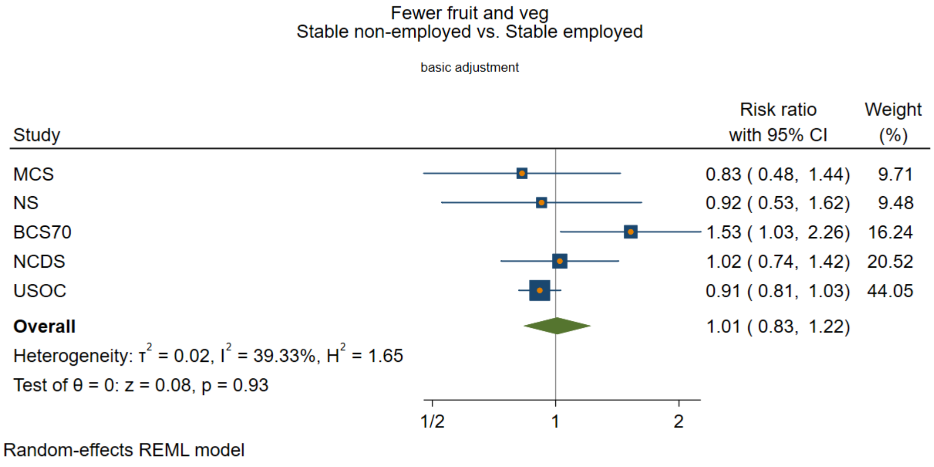
Fewer fruit and veg
Stable non-employed vs. Stable employed
basic adjustment
Study
Risk ratio with 95% CI
Weight (%)
MCS 0.83 (0.48, 1.44) 9.71
NS 0.92 (0.53, 1.62) 9.48
BCS70 1.53 (1.03, 2.26) 16.24
NCDS 1.02 (0.74, 1.42) 20.52
USOC 0.91 (0.81, 1.03) 44.05
Overall 1.01 (0.83, 1.22)
Heterogeneity: τ² = 0.02, I² = 39.33%, H² = 1.65
Test of θ = 0: z = 0.08, p = 0.93
1/2
1
2
Random-effects REML model

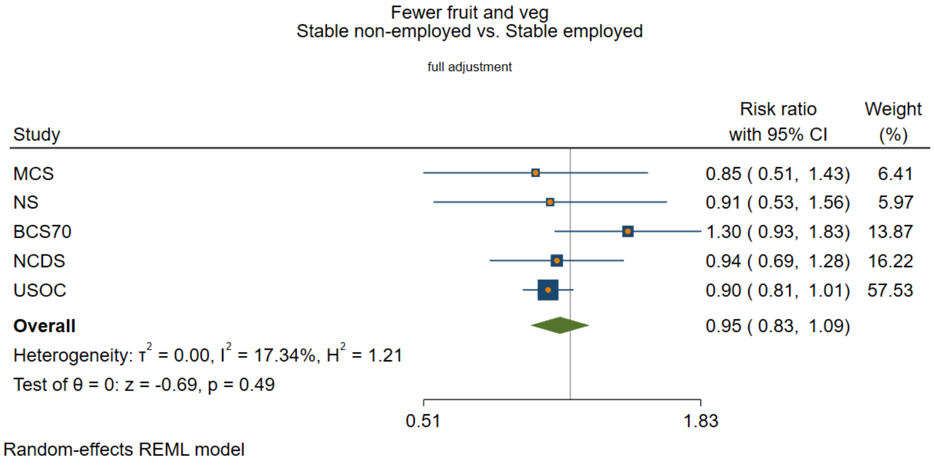
Fewer fruit and veg
Stable non-employed vs. Stable employed
full adjustment
Study
Risk ratio with 95% CI
Weight (%)
MCS 0.85 (0.51, 1.43) 6.41
NS 0.91 (0.53, 1.56) 5.97
BCS70 1.30 (0.93, 1.83) 13.87
NCDS 0.94 (0.69, 1.28) 16.22
USOC 0.90 (0.81, 1.01) 57.53
Overall 0.95 (0.83, 1.09)
Heterogeneity: τ² = 0.00, I² = 17.34%, H² = 1.21
Test of θ = 0: z = -0.69, p = 0.49
0.51
1.83
Random-effects REML model

Figure set 3: More fruit and vegetables

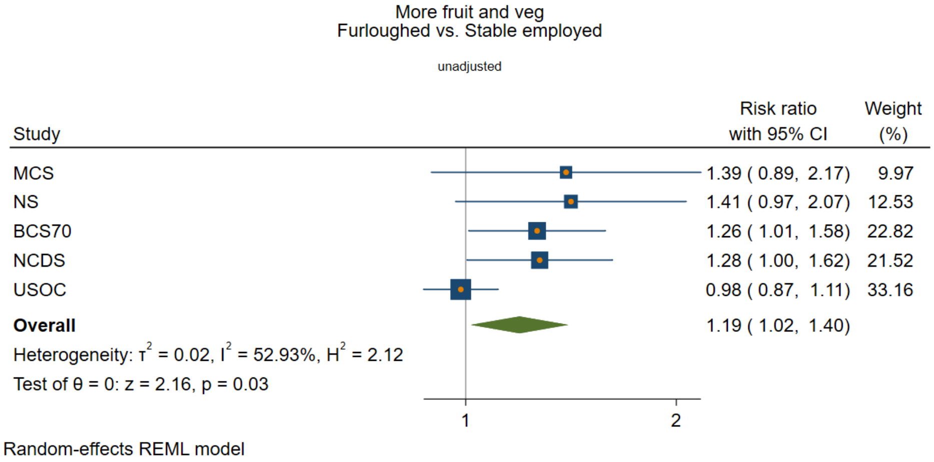

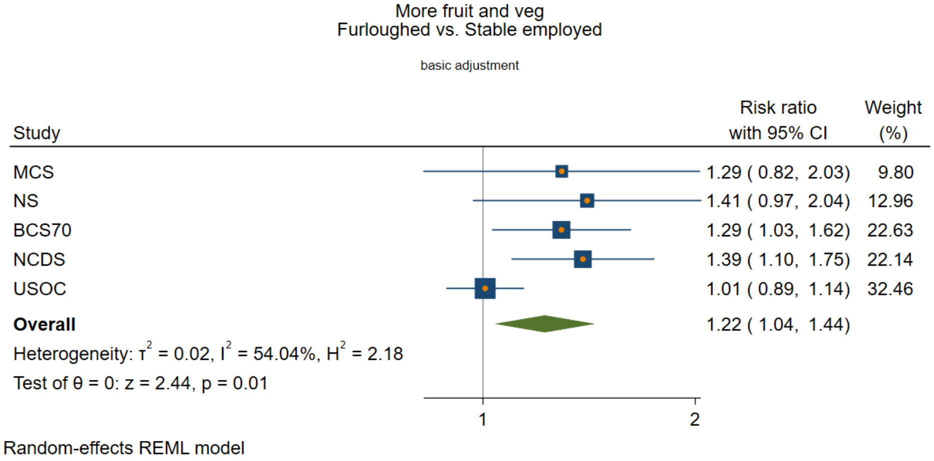

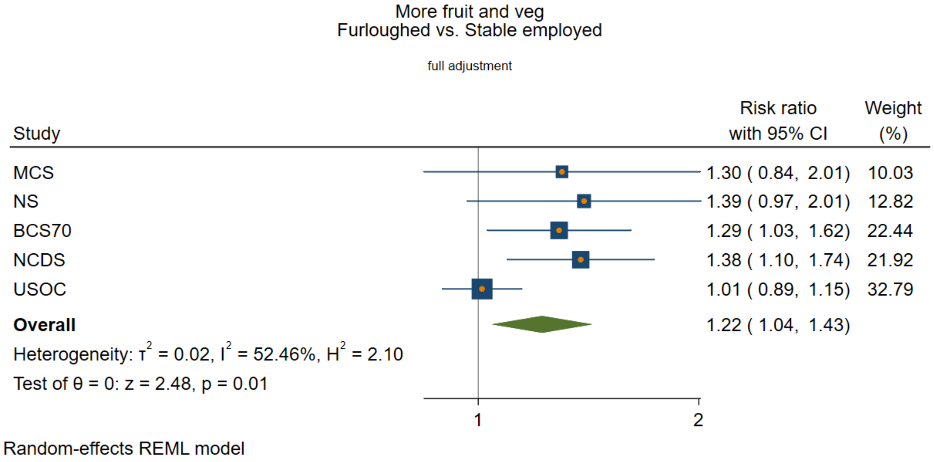

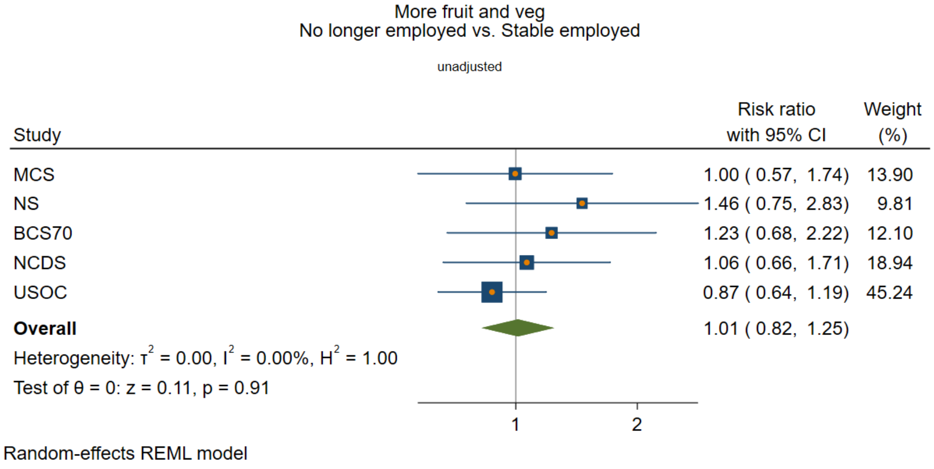
More fruit and veg
No longer employed vs. Stable employed
unadjusted
Study
Risk ratio with 95% CI
Weight (%)
MCS 1.00 (0.57, 1.74) 13.90
NS 1.46 (0.75, 2.83) 9.81
BCS70 1.23 (0.68, 2.22) 12.10
NCDS 1.06 (0.66, 1.71) 18.94
USOC 0.87 (0.64, 1.19) 45.24
Overall 1.01 (0.82, 1.25)
Heterogeneity: τ^2 = 0.00, I^2 = 0.00%, H^2 = 1.00
Test of θ = 0: z = 0.11, p = 0.91
1
2
Random-effects REML model

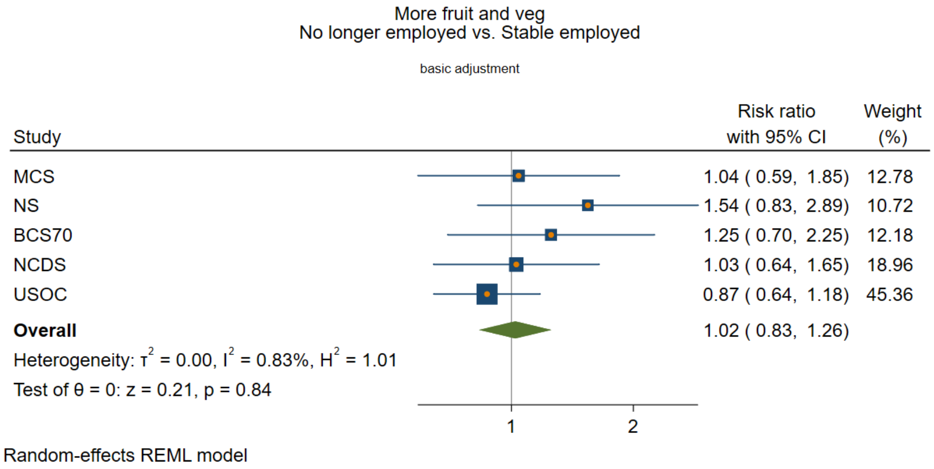
More fruit and veg
No longer employed vs. Stable employed
basic adjustment
Study
Risk ratio with 95% CI
Weight (%)
MCS 1.04 (0.59, 1.85) 12.78
NS 1.54 (0.83, 2.89) 10.72
BCS70 1.25 (0.70, 2.25) 12.18
NCDS 1.03 (0.64, 1.65) 18.96
USOC 0.87 (0.64, 1.18) 45.36
Overall 1.02 (0.83, 1.26)
Heterogeneity: τ^2 = 0.00, I^2 = 0.83%, H^2 = 1.01
Test of θ = 0: z = 0.21, p = 0.84
1
2
Random-effects REML model

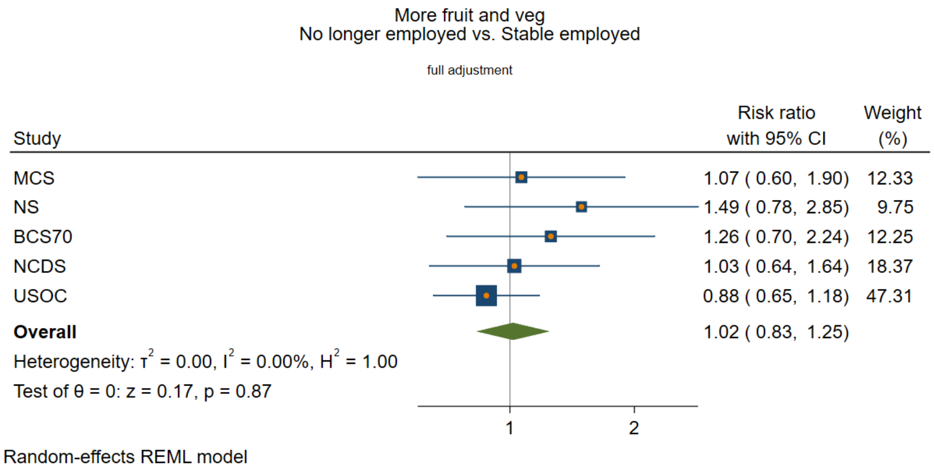
More fruit and veg
No longer employed vs. Stable employed
full adjustment
Study
Risk ratio with 95% CI
Weight (%)
MCS 1.07 (0.60, 1.90) 12.33
NS 1.49 (0.78, 2.85) 9.75
BCS70 1.26 (0.70, 2.24) 12.25
NCDS 1.03 (0.64, 1.64) 18.37
USOC 0.88 (0.65, 1.18) 47.31
Overall 1.02 (0.83, 1.25)
Heterogeneity: τ^2 = 0.00, I^2 = 0.00%, H^2 = 1.00
Test of θ = 0: z = 0.17, p = 0.87
1
2
Random-effects REML model

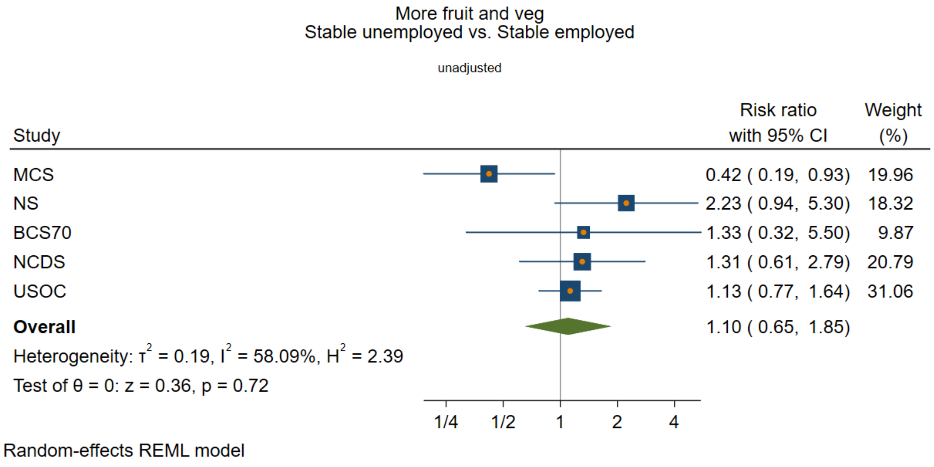
More fruit and veg
Stable unemployed vs. Stable employed
unadjusted
Study
Risk ratio with 95% CI
Weight (%)
MCS 0.42 (0.19, 0.93) 19.96
NS 2.23 (0.94, 5.30) 18.32
BCS70 1.33 (0.32, 5.50) 9.87
NCDS 1.31 (0.61, 2.79) 20.79
USOC 1.13 (0.77, 1.64) 31.06
Overall 1.10 (0.65, 1.85)
Heterogeneity: τ^2 = 0.19, I^2 = 58.09%, H^2 = 2.39
Test of θ = 0: z = 0.36, p = 0.72
1/4
1/2
1
2
4
Random-effects REML model

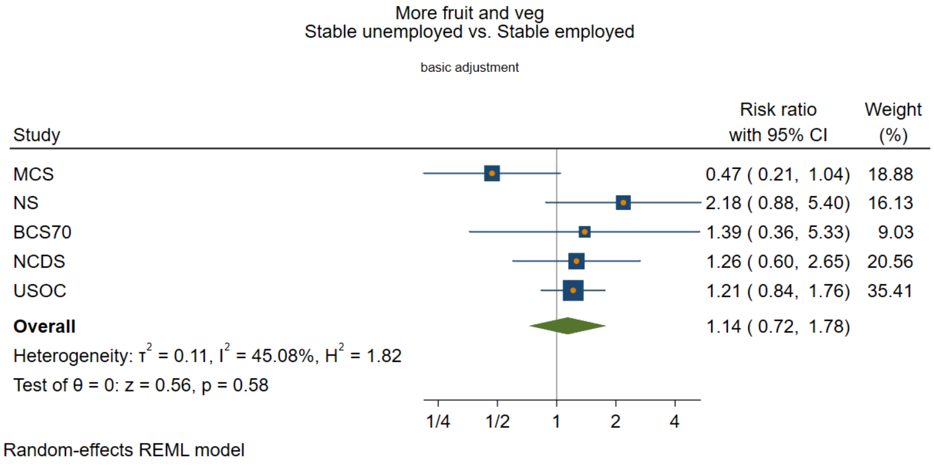
More fruit and veg
Stable unemployed vs. Stable employed
basic adjustment
Study
Risk ratio with 95% CI
Weight (%)
MCS 0.47 (0.21, 1.04) 18.88
NS 2.18 (0.88, 5.40) 16.13
BCS70 1.39 (0.36, 5.33) 9.03
NCDS 1.26 (0.60, 2.65) 20.56
USOC 1.21 (0.84, 1.76) 35.41
Overall 1.14 (0.72, 1.78)
Heterogeneity: τ^2 = 0.11, I^2 = 45.08%, H^2 = 1.82
Test of θ = 0: z = 0.56, p = 0.58
1/4
1/2
1
2
4
Random-effects REML model

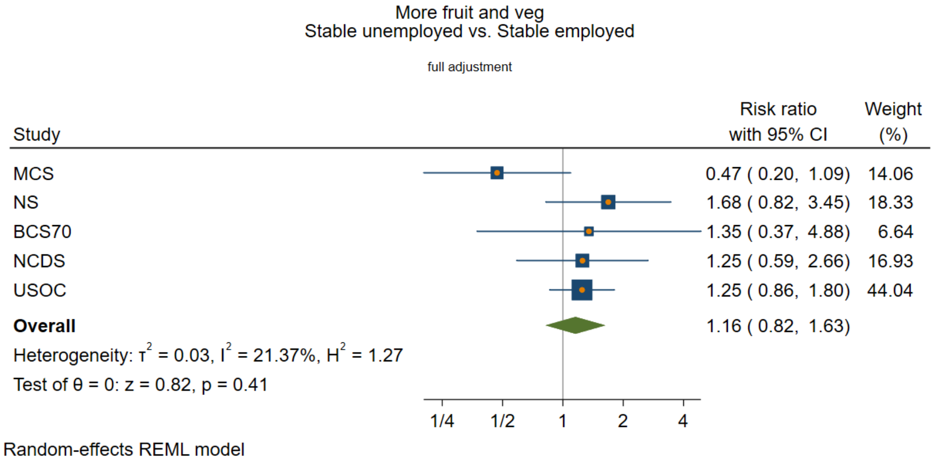
More fruit and veg
Stable unemployed vs. Stable employed
full adjustment
Study
Risk ratio with 95% CI
Weight (%)
MCS 0.47 (0.20, 1.09) 14.06
NS 1.68 (0.82, 3.45) 18.33
BCS70 1.35 (0.37, 4.88) 6.64
NCDS 1.25 (0.59, 2.66) 16.93
USOC 1.25 (0.86, 1.80) 44.04
Overall 1.16 (0.82, 1.63)
Heterogeneity: τ^2 = 0.03, I^2 = 21.37%, H^2 = 1.27
Test of θ = 0: z = 0.82, p = 0.41
1/4
1/2
1
2
4
Random-effects REML model

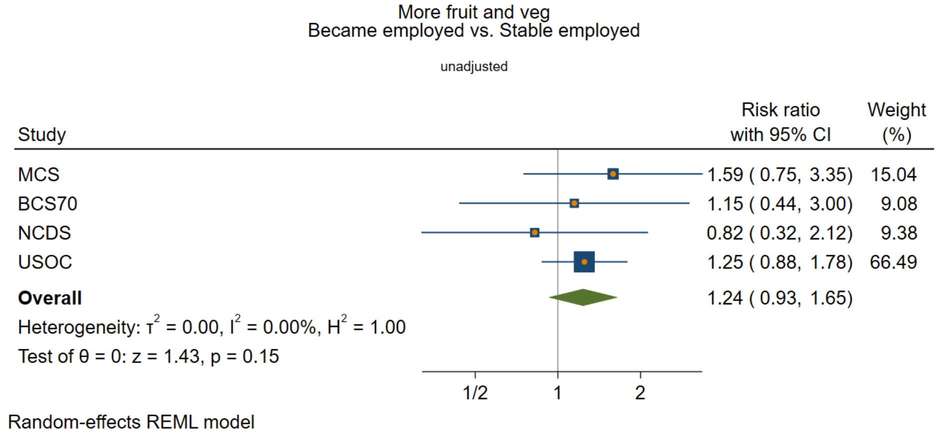
More fruit and veg
Became employed vs. Stable employed
unadjusted
Study
Risk ratio with 95% CI
Weight (%)
MCS 1.59 (0.75, 3.35) 15.04
BCS70 1.15 (0.44, 3.00) 9.08
NCDS 0.82 (0.32, 2.12) 9.38
USOC 1.25 (0.88, 1.78) 66.49
Overall 1.24 (0.93, 1.65)
Heterogeneity: τ² = 0.00, I² = 0.00%, H² = 1.00
Test of θ = 0: z = 1.43, p = 0.15
1/2
1
2
Random-effects REML model

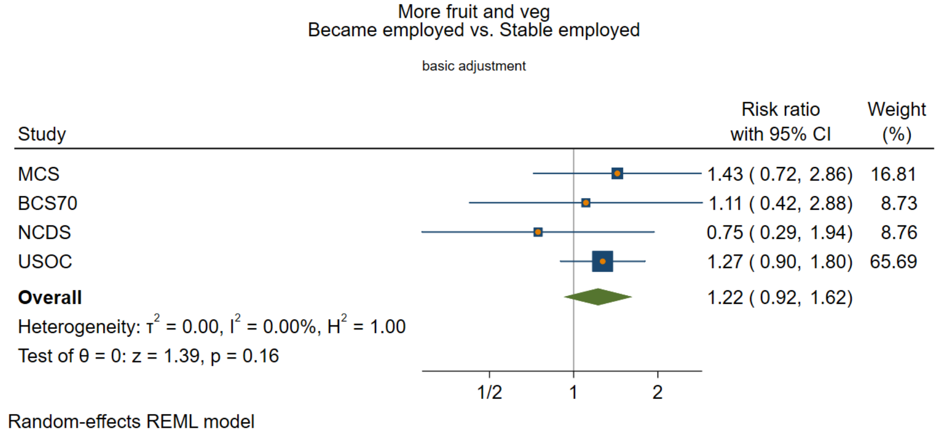
More fruit and veg
Became employed vs. Stable employed
basic adjustment
Study
Risk ratio with 95% CI
Weight (%)
MCS 1.43 (0.72, 2.86) 16.81
BCS70 1.11 (0.42, 2.88) 8.73
NCDS 0.75 (0.29, 1.94) 8.76
USOC 1.27 (0.90, 1.80) 65.69
Overall 1.22 (0.92, 1.62)
Heterogeneity: τ² = 0.00, I² = 0.00%, H² = 1.00
Test of θ = 0: z = 1.39, p = 0.16
1/2
1
2
Random-effects REML model

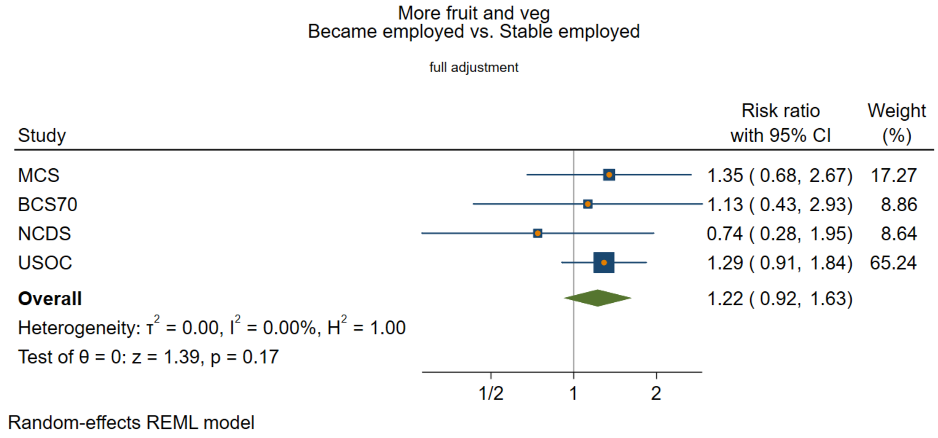
More fruit and veg
Became employed vs. Stable employed
full adjustment
Study
Risk ratio with 95% CI
Weight (%)
MCS 1.35 (0.68, 2.67) 17.27
BCS70 1.13 (0.43, 2.93) 8.86
NCDS 0.74 (0.28, 1.95) 8.64
USOC 1.29 (0.91, 1.84) 65.24
Overall 1.22 (0.92, 1.63)
Heterogeneity: τ² = 0.00, I² = 0.00%, H² = 1.00
Test of θ = 0: z = 1.39, p = 0.17
1/2
1
2
Random-effects REML model

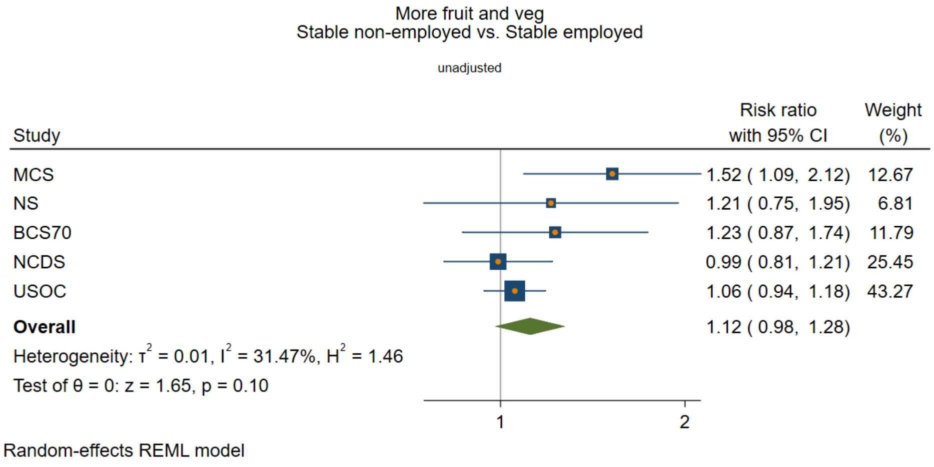
More fruit and veg
Stable non-employed vs. Stable employed
unadjusted
Study
Risk ratio with 95% CI
Weight (%)
MCS 1.52 (1.09, 2.12) 12.67
NS 1.21 (0.75, 1.95) 6.81
BCS70 1.23 (0.87, 1.74) 11.79
NCDS 0.99 (0.81, 1.21) 25.45
USOC 1.06 (0.94, 1.18) 43.27
Overall 1.12 (0.98, 1.28)
Heterogeneity: τ² = 0.01, I² = 31.47%, H² = 1.46
Test of θ = 0: z = 1.65, p = 0.10
1
2
Random-effects REML model

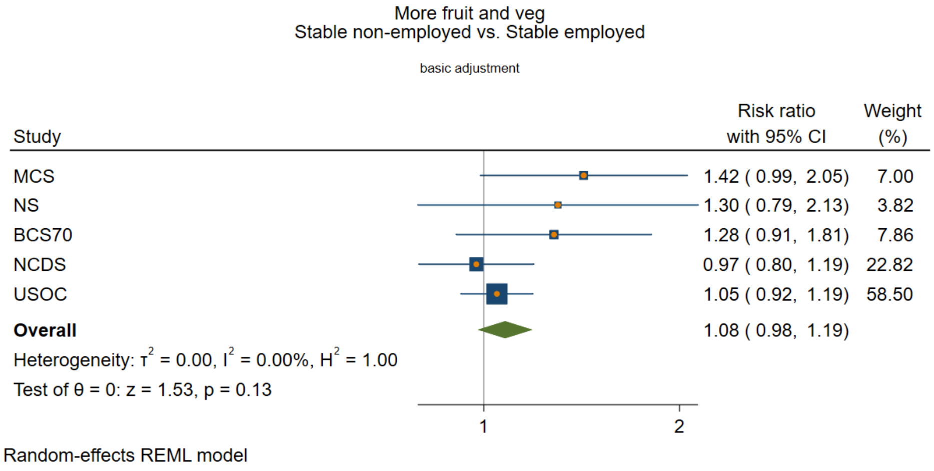
More fruit and veg
Stable non-employed vs. Stable employed
basic adjustment
Study
Risk ratio with 95% CI
Weight (%)
MCS 1.42 (0.99, 2.05) 7.00
NS 1.30 (0.79, 2.13) 3.82
BCS70 1.28 (0.91, 1.81) 7.86
NCDS 0.97 (0.80, 1.19) 22.82
USOC 1.05 (0.92, 1.19) 58.50
Overall 1.08 (0.98, 1.19)
Heterogeneity: τ² = 0.00, I² = 0.00%, H² = 1.00
Test of θ = 0: z = 1.53, p = 0.13
1
2
Random-effects REML model

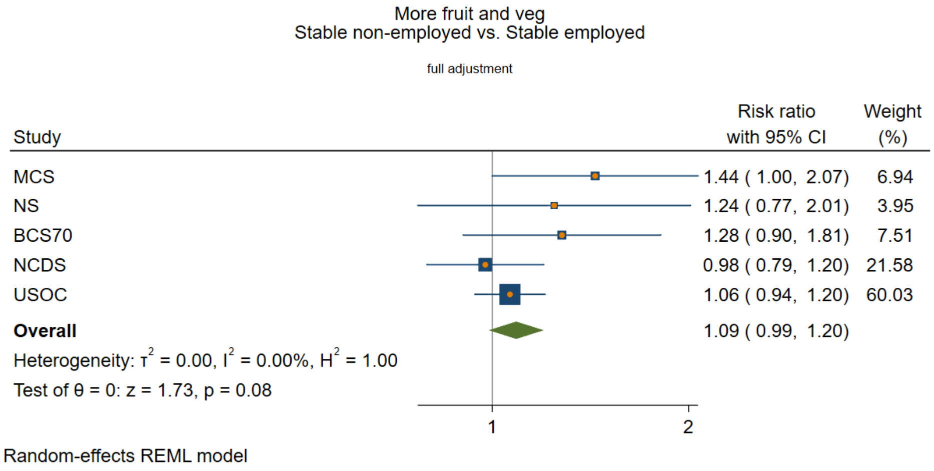
More fruit and veg
Stable non-employed vs. Stable employed
full adjustment
Study
Risk ratio with 95% CI
Weight (%)
MCS 1.44 (1.00, 2.07) 6.94
NS 1.24 (0.77, 2.01) 3.95
BCS70 1.28 (0.90, 1.81) 7.51
NCDS 0.98 (0.79, 1.20) 21.58
USOC 1.06 (0.94, 1.20) 60.03
Overall 1.09 (0.99, 1.20)
Heterogeneity: τ² = 0.00, I² = 0.00%, H² = 1.00
Test of θ = 0: z = 1.73, p = 0.08
1
2
Random-effects REML model

Figure set 4: Less than 3 days a week of at least 30 min exercise

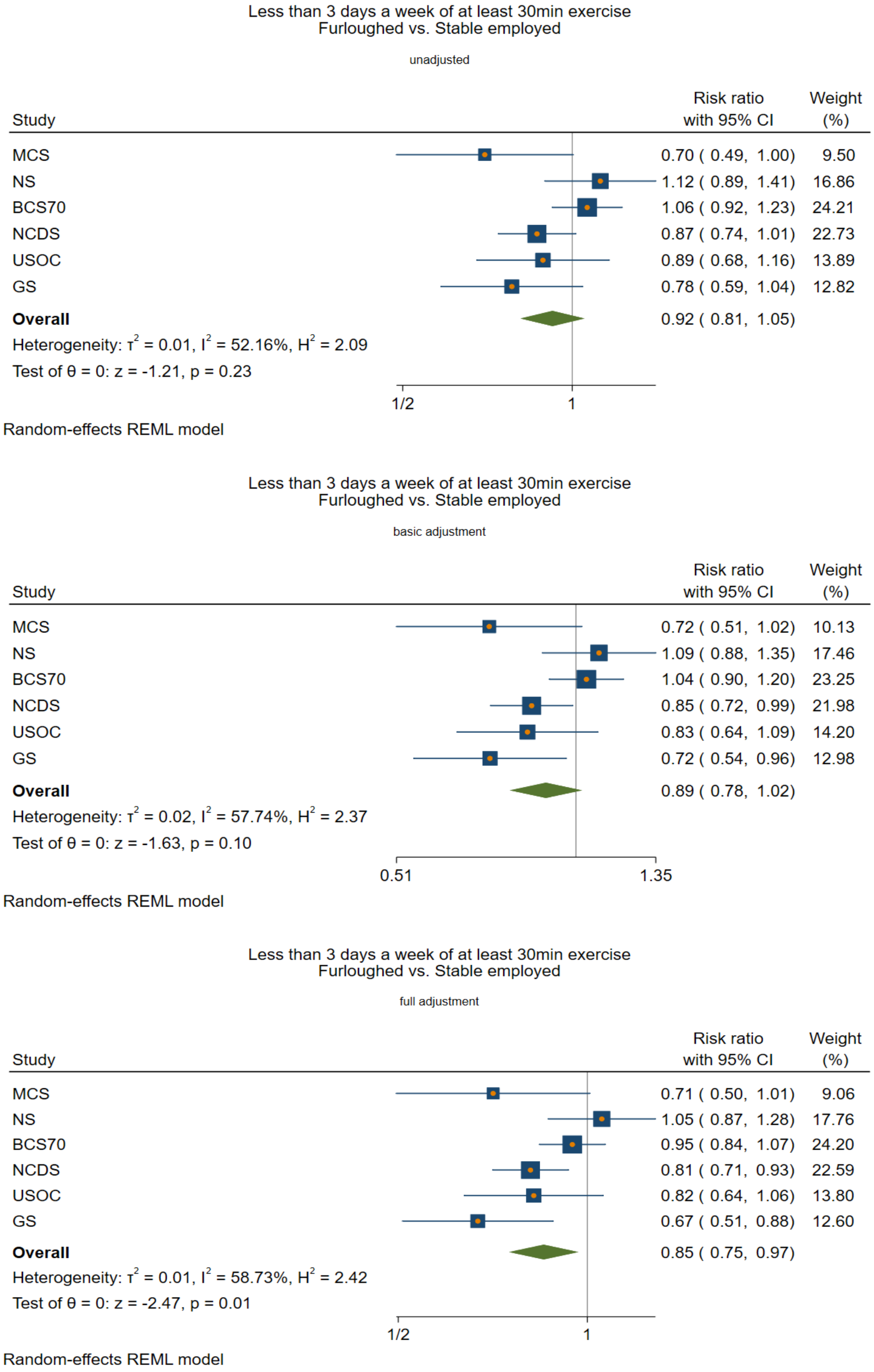

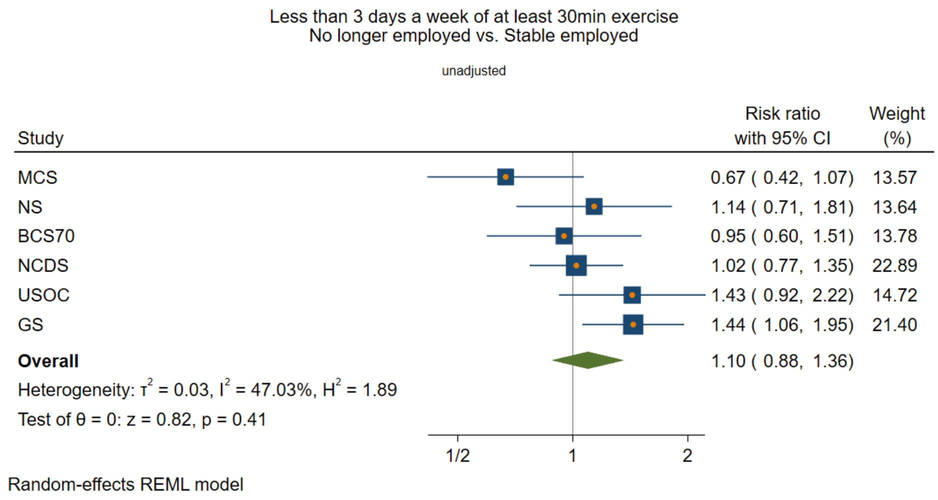
Less than 3 days a week of at least 30min exercise
No longer employed vs. Stable employed
unadjusted
Study
Risk ratio with 95% CI
Weight (%)
MCS 0.67 (0.42, 1.07) 13.57
NS 1.14 (0.71, 1.81) 13.64
BCS70 0.95 (0.60, 1.51) 13.78
NCDS 1.02 (0.77, 1.35) 22.89
USOC 1.43 (0.92, 2.22) 14.72
GS 1.44 (1.06, 1.95) 21.40
Overall 1.10 (0.88, 1.36)
Heterogeneity: τ² = 0.03, I² = 47.03%, H² = 1.89
Test of θ = 0: z = 0.82, p = 0.41
1/2
1
2
Random-effects REML model

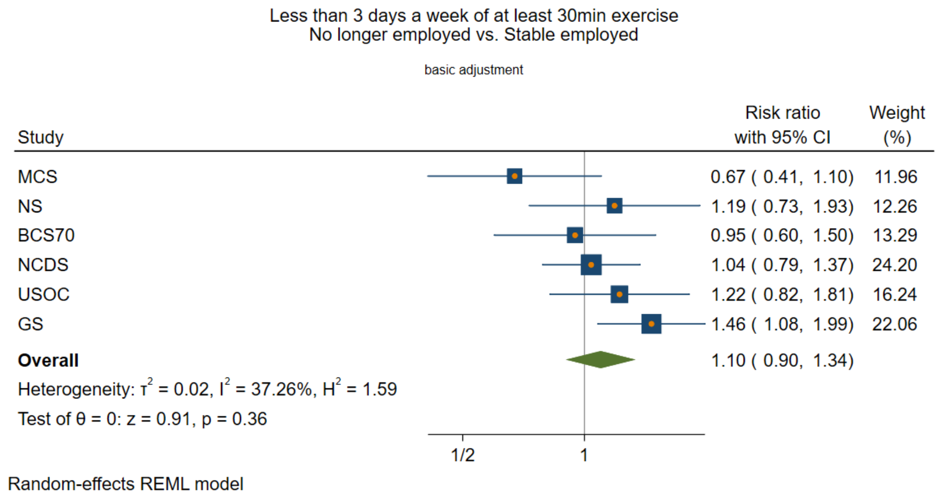
Less than 3 days a week of at least 30min exercise
No longer employed vs. Stable employed
basic adjustment
Study
Risk ratio with 95% CI
Weight (%)
MCS 0.67 (0.41, 1.10) 11.96
NS 1.19 (0.73, 1.93) 12.26
BCS70 0.95 (0.60, 1.50) 13.29
NCDS 1.04 (0.79, 1.37) 24.20
USOC 1.22 (0.82, 1.81) 16.24
GS 1.46 (1.08, 1.99) 22.06
Overall 1.10 (0.90, 1.34)
Heterogeneity: τ² = 0.02, I² = 37.26%, H² = 1.59
Test of θ = 0: z = 0.91, p = 0.36
1/2
1
Random-effects REML model

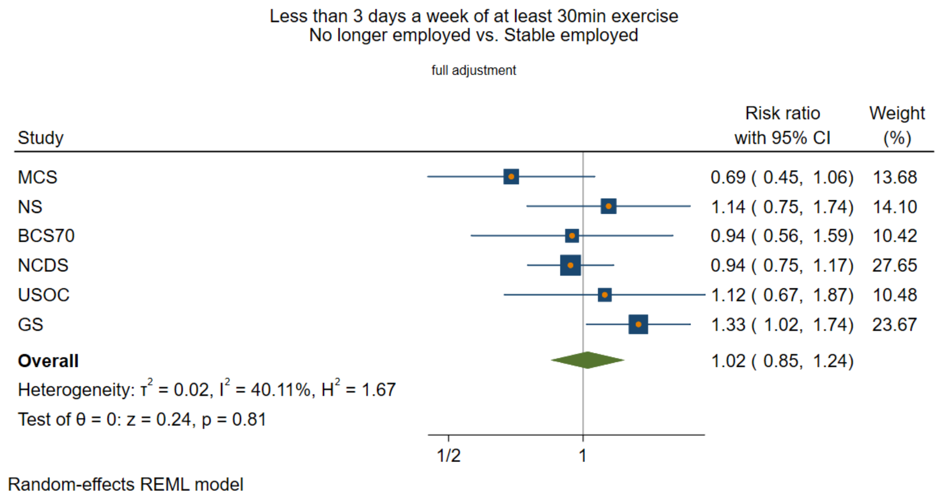
Less than 3 days a week of at least 30min exercise
No longer employed vs. Stable employed
full adjustment
Study
Risk ratio with 95% CI
Weight (%)
MCS 0.69 (0.45, 1.06) 13.68
NS 1.14 (0.75, 1.74) 14.10
BCS70 0.94 (0.56, 1.59) 10.42
NCDS 0.94 (0.75, 1.17) 27.65
USOC 1.12 (0.67, 1.87) 10.48
GS 1.33 (1.02, 1.74) 23.67
Overall 1.02 (0.85, 1.24)
Heterogeneity: τ² = 0.02, I² = 40.11%, H² = 1.67
Test of θ = 0: z = 0.24, p = 0.81
1/2
1
Random-effects REML model

Less than 3 days a week of at least 30min exercise
Stable unemployed vs. Stable employed

unadjusted

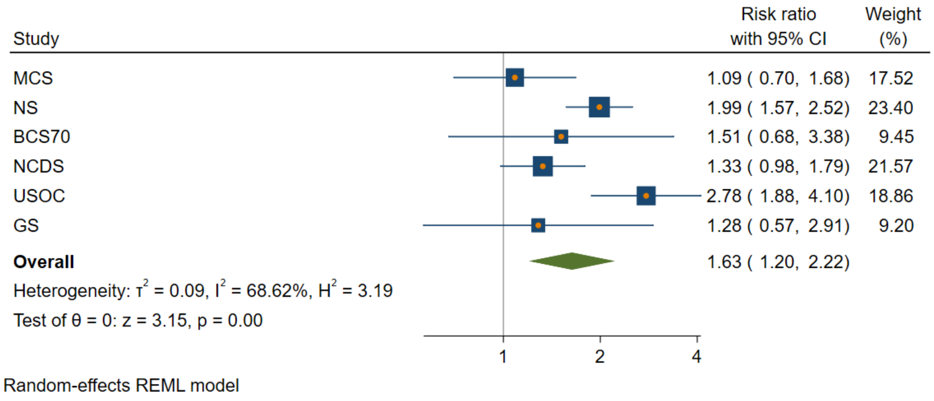

Less than 3 days a week of at least 30min exercise
Stable unemployed vs. Stable employed

basic adjustment

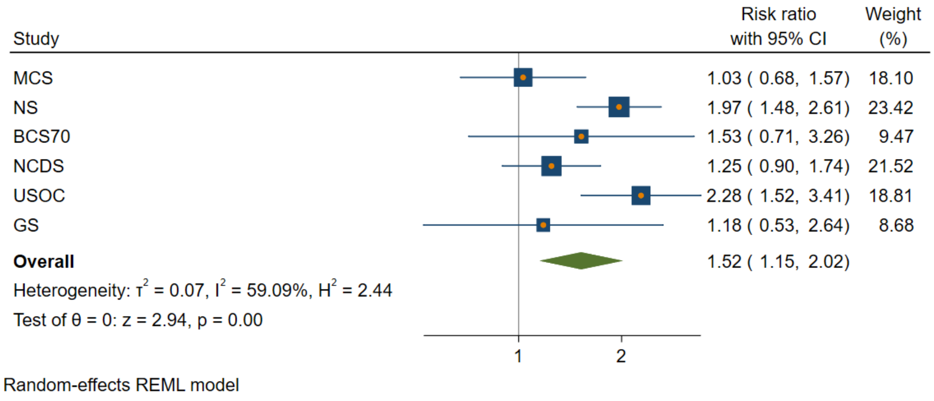

Less than 3 days a week of at least 30min exercise
Stable unemployed vs. Stable employed

full adjustment

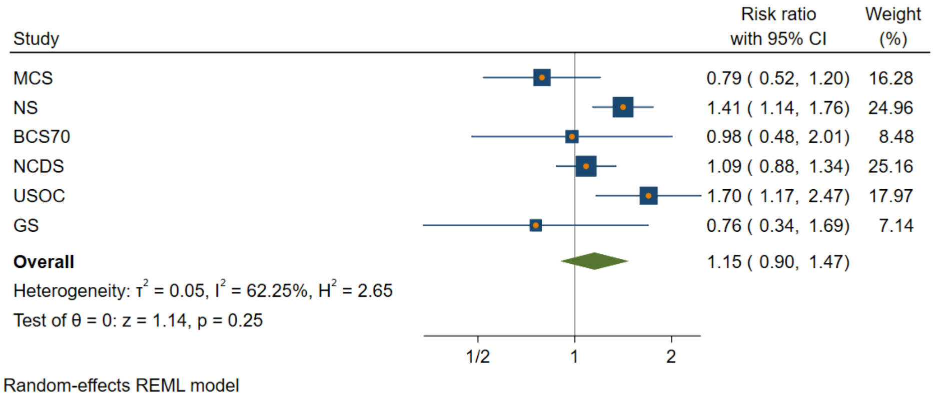

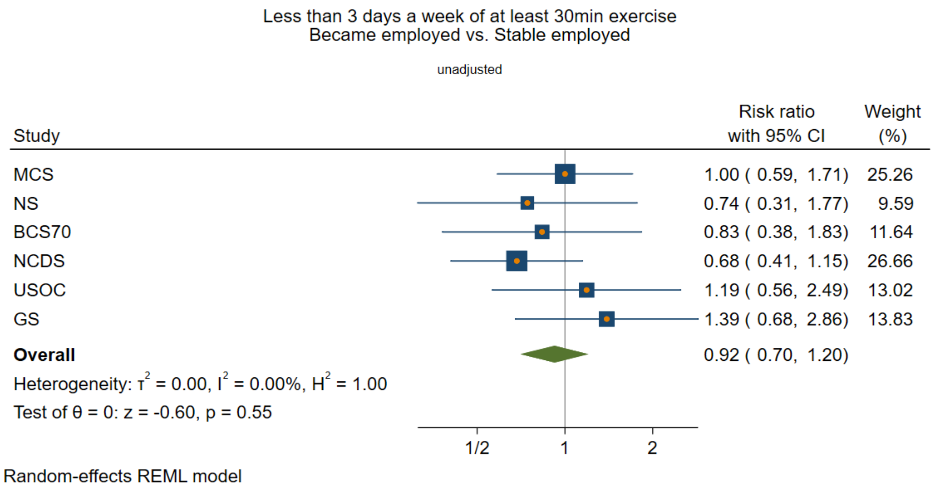
Less than 3 days a week of at least 30min exercise
Became employed vs. Stable employed
unadjusted
Study
Risk ratio with 95% CI
Weight (%)
MCS 1.00 (0.59, 1.71) 25.26
NS 0.74 (0.31, 1.77) 9.59
BCS70 0.83 (0.38, 1.83) 11.64
NCDS 0.68 (0.41, 1.15) 26.66
USOC 1.19 (0.56, 2.49) 13.02
GS 1.39 (0.68, 2.86) 13.83
Overall 0.92 (0.70, 1.20)
Heterogeneity: τ² = 0.00, I² = 0.00%, H² = 1.00
Test of θ = 0: z = -0.60, p = 0.55
1/2
1
2
Random-effects REML model

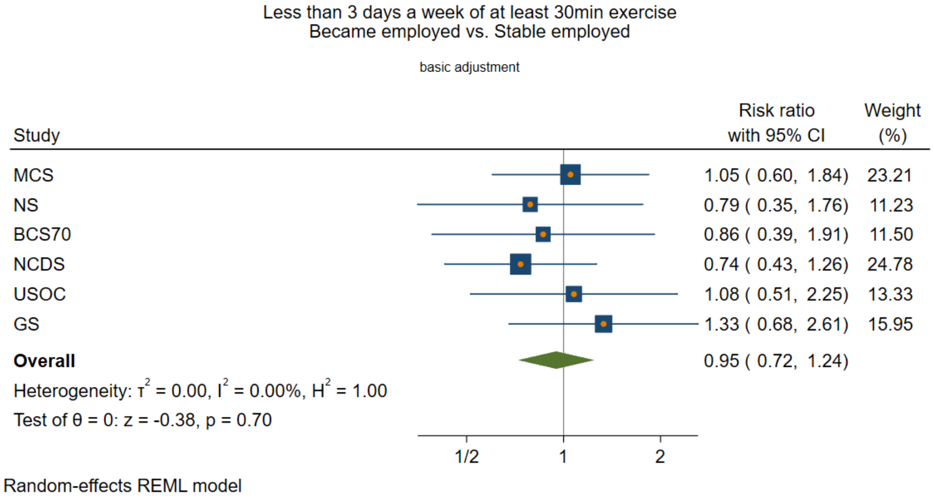
Less than 3 days a week of at least 30min exercise
Became employed vs. Stable employed
basic adjustment
Study
Risk ratio with 95% CI
Weight (%)
MCS 1.05 (0.60, 1.84) 23.21
NS 0.79 (0.35, 1.76) 11.23
BCS70 0.86 (0.39, 1.91) 11.50
NCDS 0.74 (0.43, 1.26) 24.78
USOC 1.08 (0.51, 2.25) 13.33
GS 1.33 (0.68, 2.61) 15.95
Overall 0.95 (0.72, 1.24)
Heterogeneity: τ² = 0.00, I² = 0.00%, H² = 1.00
Test of θ = 0: z = -0.38, p = 0.70
1/2
1
2
Random-effects REML model

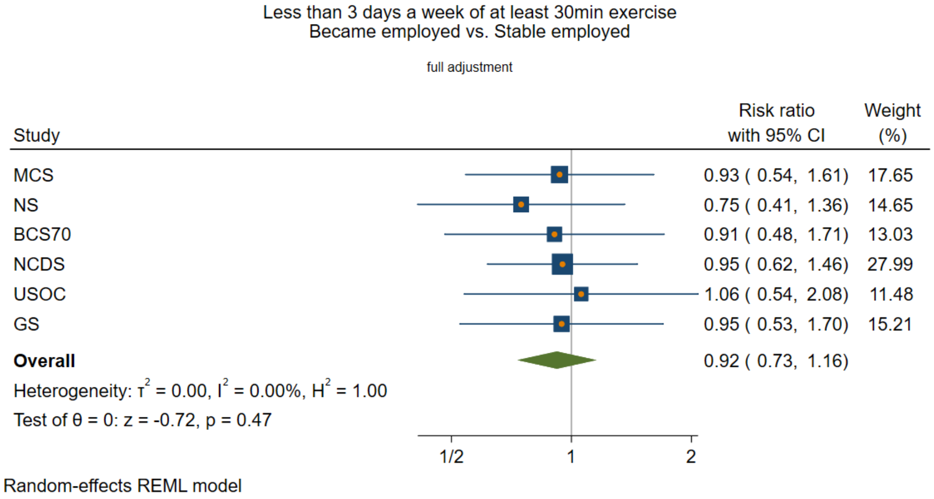
Less than 3 days a week of at least 30min exercise
Became employed vs. Stable employed
full adjustment
Study
Risk ratio with 95% CI
Weight (%)
MCS 0.93 (0.54, 1.61) 17.65
NS 0.75 (0.41, 1.36) 14.65
BCS70 0.91 (0.48, 1.71) 13.03
NCDS 0.95 (0.62, 1.46) 27.99
USOC 1.06 (0.54, 2.08) 11.48
GS 0.95 (0.53, 1.70) 15.21
Overall 0.92 (0.73, 1.16)
Heterogeneity: τ² = 0.00, I² = 0.00%, H² = 1.00
Test of θ = 0: z = -0.72, p = 0.47
1/2
1
2
Random-effects REML model

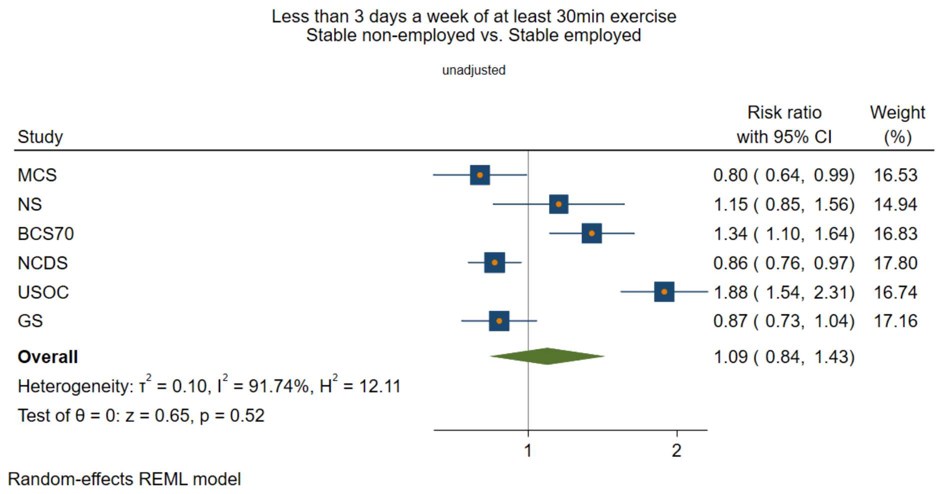
Less than 3 days a week of at least 30min exercise
Stable non-employed vs. Stable employed
unadjusted
Study
Risk ratio with 95% CI
Weight (%)
MCS 0.80 (0.64, 0.99) 16.53
NS 1.15 (0.85, 1.56) 14.94
BCS70 1.34 (1.10, 1.64) 16.83
NCDS 0.86 (0.76, 0.97) 17.80
USOC 1.88 (1.54, 2.31) 16.74
GS 0.87 (0.73, 1.04) 17.16
Overall 1.09 (0.84, 1.43)
Heterogeneity: τ^2 = 0.10, I^2 = 91.74%, H^2 = 12.11
Test of θ = 0: z = 0.65, p = 0.52
1
2
Random-effects REML model

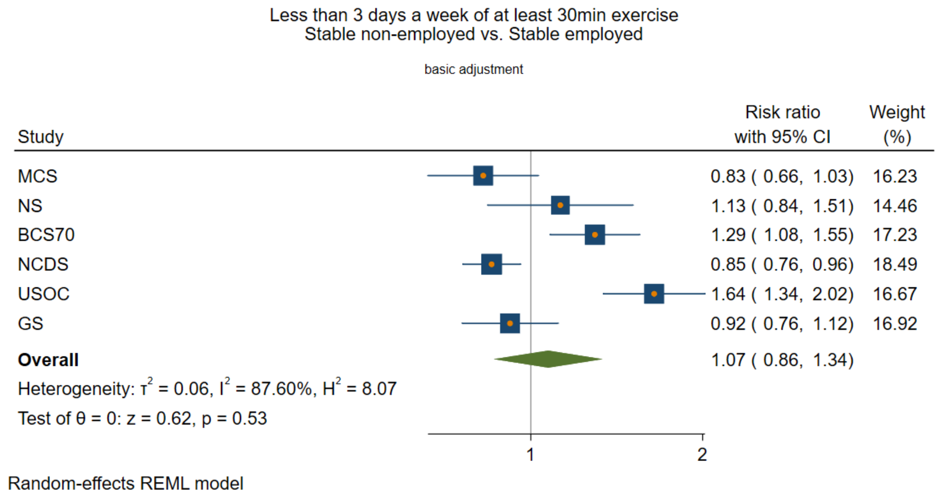
Less than 3 days a week of at least 30min exercise
Stable non-employed vs. Stable employed
basic adjustment
Study
Risk ratio with 95% CI
Weight (%)
MCS 0.83 (0.66, 1.03) 16.23
NS 1.13 (0.84, 1.51) 14.46
BCS70 1.29 (1.08, 1.55) 17.23
NCDS 0.85 (0.76, 0.96) 18.49
USOC 1.64 (1.34, 2.02) 16.67
GS 0.92 (0.76, 1.12) 16.92
Overall 1.07 (0.86, 1.34)
Heterogeneity: τ^2 = 0.06, I^2 = 87.60%, H^2 = 8.07
Test of θ = 0: z = 0.62, p = 0.53
1
2
Random-effects REML model

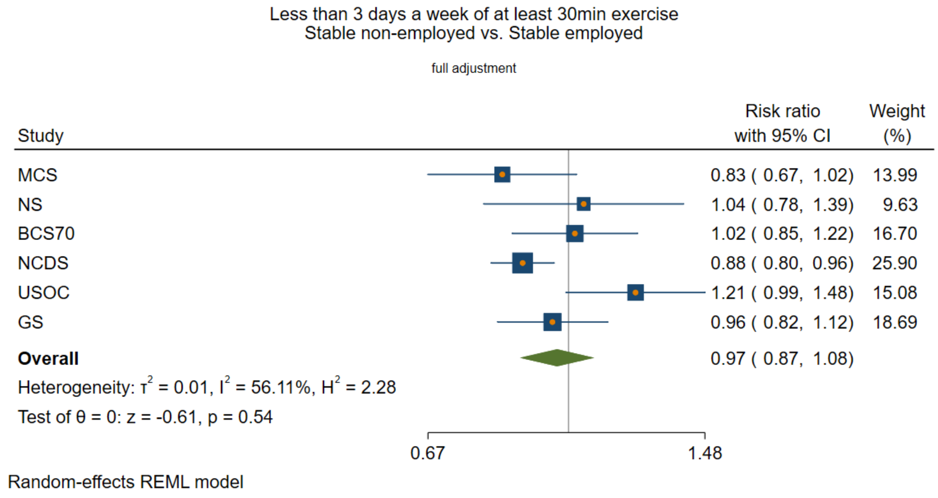
Less than 3 days a week of at least 30min exercise
Stable non-employed vs. Stable employed
full adjustment
Study
Risk ratio with 95% CI
Weight (%)
MCS 0.83 (0.67, 1.02) 13.99
NS 1.04 (0.78, 1.39) 9.63
BCS70 1.02 (0.85, 1.22) 16.70
NCDS 0.88 (0.80, 0.96) 25.90
USOC 1.21 (0.99, 1.48) 15.08
GS 0.96 (0.82, 1.12) 18.69
Overall 0.97 (0.87, 1.08)
Heterogeneity: τ^2 = 0.01, I^2 = 56.11%, H^2 = 2.28
Test of θ = 0: z = -0.61, p = 0.54
0.67
1.48
Random-effects REML model

Figure set 5: Less time/fewer days of physical exercise

Commented [MJ1]:

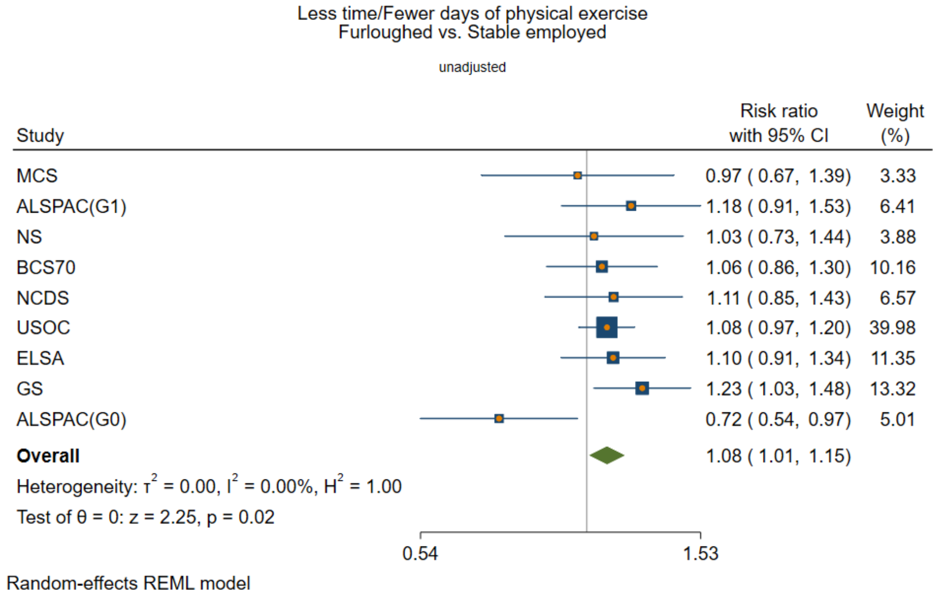

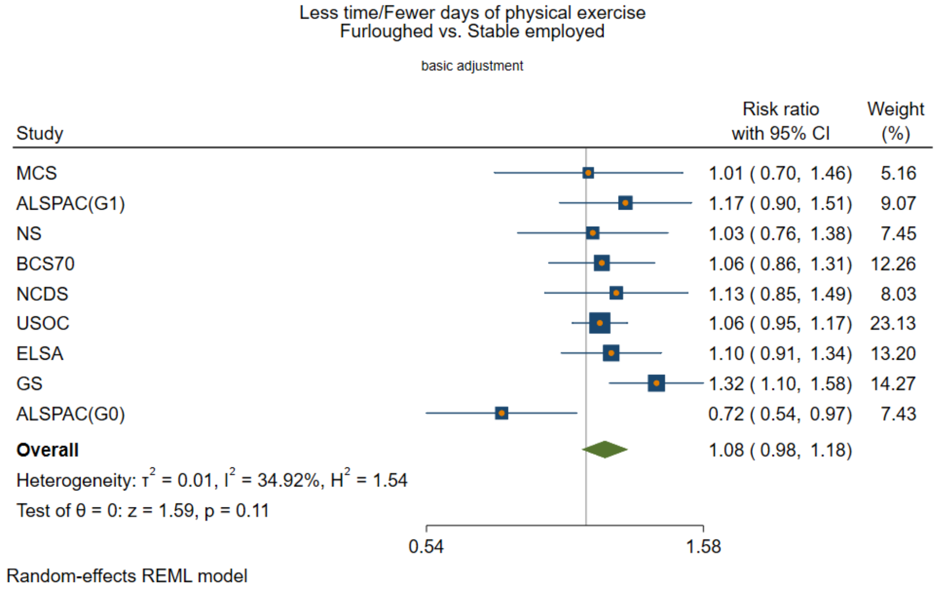

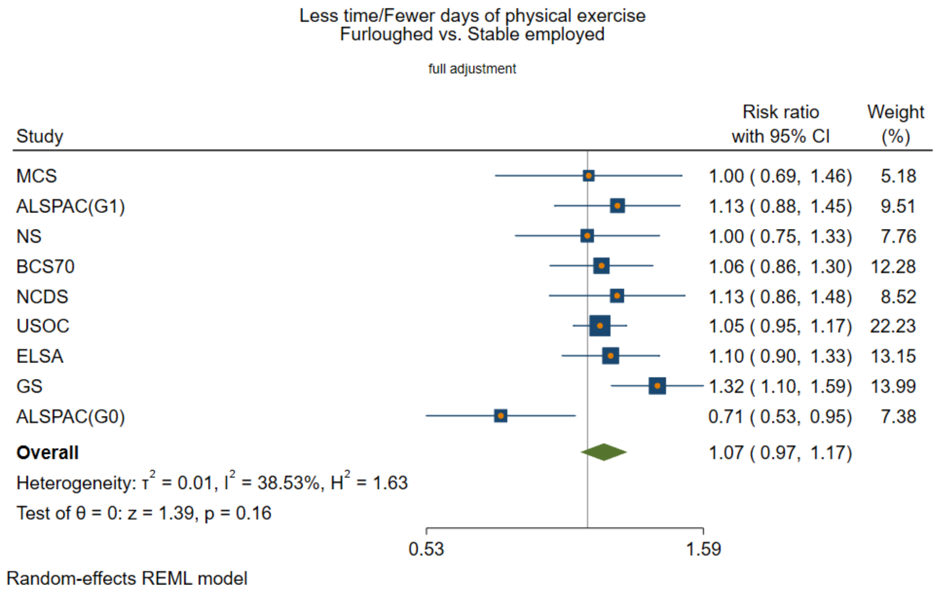
Less time/Fewer days of physical exercise
Furloughed vs. Stable employed
full adjustment
Study
Risk ratio with 95% CI
Weight (%)
MCS 1.00 (0.69, 1.46) 5.18
ALSPAC(G1) 1.13 (0.88, 1.45) 9.51
NS 1.00 (0.75, 1.33) 7.76
BCS70 1.06 (0.86, 1.30) 12.28
NCDS 1.13 (0.86, 1.48) 8.52
USOC 1.05 (0.95, 1.17) 22.23
ELSA 1.10 (0.90, 1.33) 13.15
GS 1.32 (1.10, 1.59) 13.99
ALSPAC(G0) 0.71 (0.53, 0.95) 7.38
Overall 1.07 (0.97, 1.17)
Heterogeneity: τ² = 0.01, I² = 38.53%, H² = 1.63
Test of θ = 0: z = 1.39, p = 0.16
0.53
1.59
Random-effects REML model

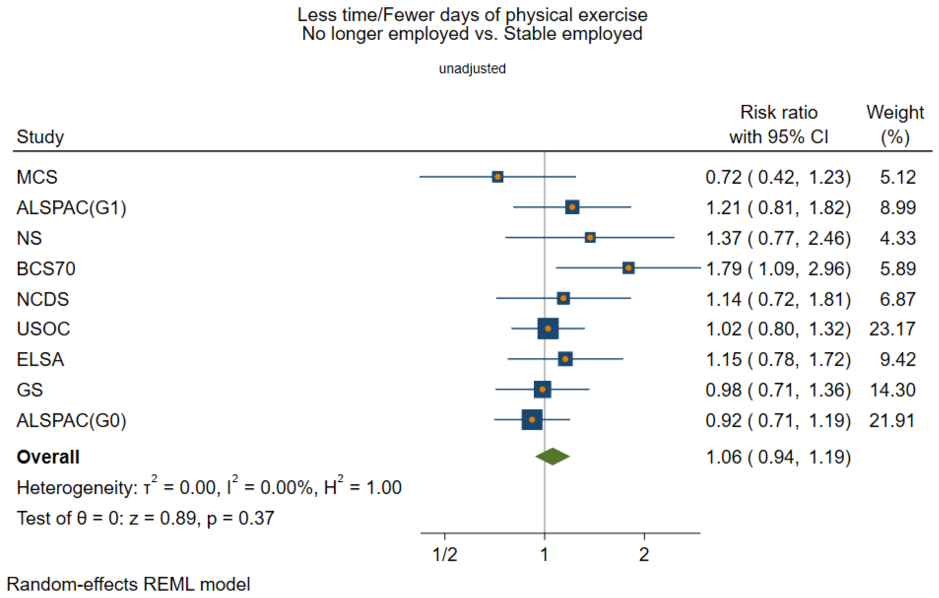
Less time/Fewer days of physical exercise
No longer employed vs. Stable employed
unadjusted
Study
Risk ratio with 95% CI
Weight (%)
MCS 0.72 (0.42, 1.23) 5.12
ALSPAC(G1) 1.21 (0.81, 1.82) 8.99
NS 1.37 (0.77, 2.46) 4.33
BCS70 1.79 (1.09, 2.96) 5.89
NCDS 1.14 (0.72, 1.81) 6.87
USOC 1.02 (0.80, 1.32) 23.17
ELSA 1.15 (0.78, 1.72) 9.42
GS 0.98 (0.71, 1.36) 14.30
ALSPAC(G0) 0.92 (0.71, 1.19) 21.91
Overall 1.06 (0.94, 1.19)
Heterogeneity: τ² = 0.00, I² = 0.00%, H² = 1.00
Test of θ = 0: z = 0.89, p = 0.37
1/2
1
2
Random-effects REML model

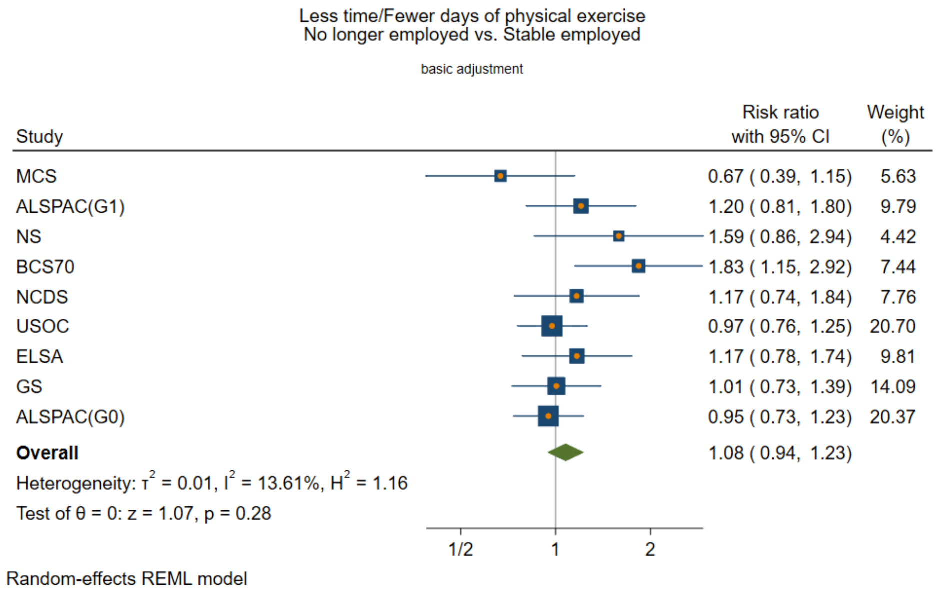
Less time/Fewer days of physical exercise
No longer employed vs. Stable employed
basic adjustment
Study
Risk ratio with 95% CI
Weight (%)
MCS 0.67 (0.39, 1.15) 5.63
ALSPAC(G1) 1.20 (0.81, 1.80) 9.79
NS 1.59 (0.86, 2.94) 4.42
BCS70 1.83 (1.15, 2.92) 7.44
NCDS 1.17 (0.74, 1.84) 7.76
USOC 0.97 (0.76, 1.25) 20.70
ELSA 1.17 (0.78, 1.74) 9.81
GS 1.01 (0.73, 1.39) 14.09
ALSPAC(G0) 0.95 (0.73, 1.23) 20.37
Overall 1.08 (0.94, 1.23)
Heterogeneity: τ² = 0.01, I² = 13.61%, H² = 1.16
Test of θ = 0: z = 1.07, p = 0.28
1/2
1
2
Random-effects REML model

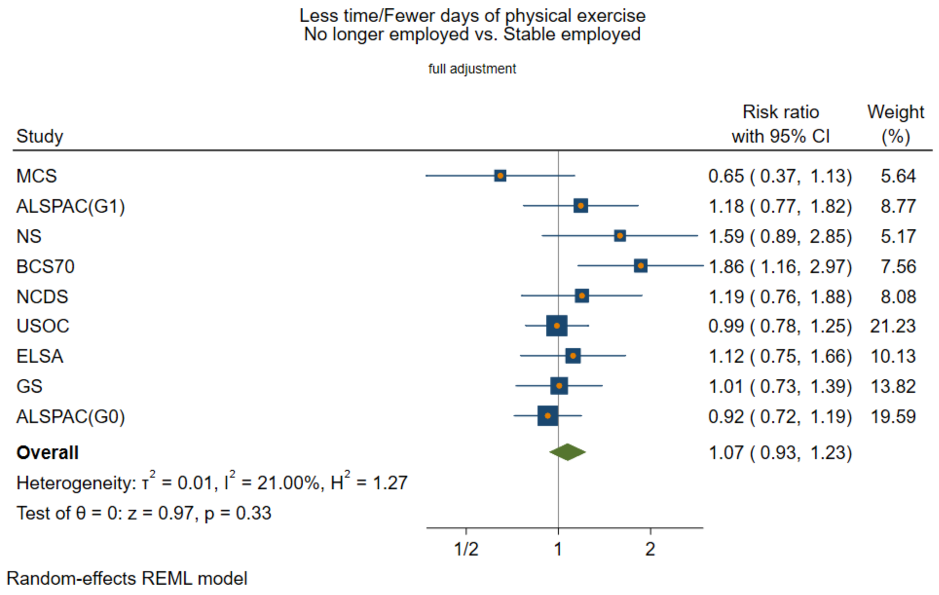
Less time/Fewer days of physical exercise
No longer employed vs. Stable employed
full adjustment
Study
Risk ratio with 95% CI
Weight (%)
MCS 0.65 (0.37, 1.13) 5.64
ALSPAC(G1) 1.18 (0.77, 1.82) 8.77
NS 1.59 (0.89, 2.85) 5.17
BCS70 1.86 (1.16, 2.97) 7.56
NCDS 1.19 (0.76, 1.88) 8.08
USOC 0.99 (0.78, 1.25) 21.23
ELSA 1.12 (0.75, 1.66) 10.13
GS 1.01 (0.73, 1.39) 13.82
ALSPAC(G0) 0.92 (0.72, 1.19) 19.59
Overall 1.07 (0.93, 1.23)
Heterogeneity: τ^2 = 0.01, I^2 = 21.00%, H^2 = 1.27
Test of θ = 0: z = 0.97, p = 0.33
1/2
1
2
Random-effects REML model

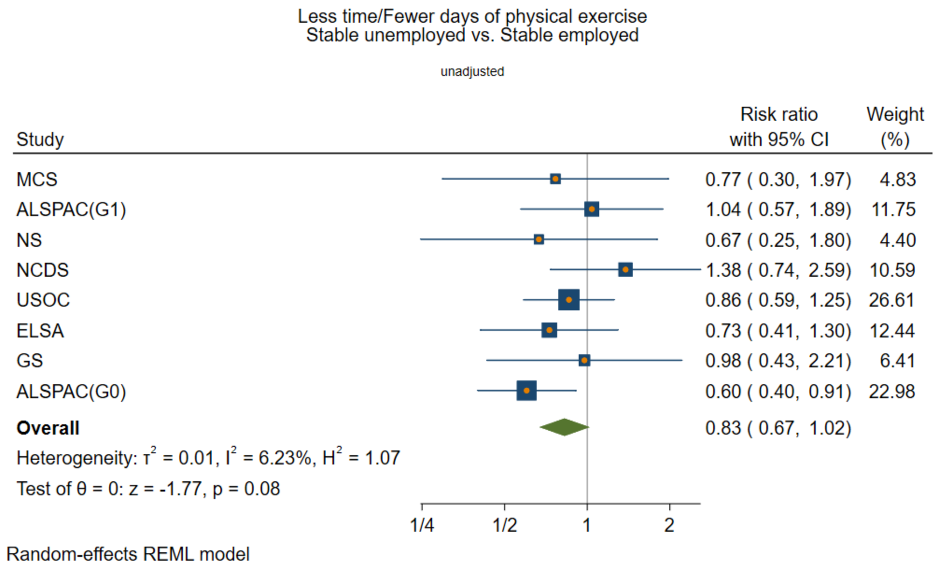
Less time/Fewer days of physical exercise
Stable unemployed vs. Stable employed
unadjusted
Study
Risk ratio with 95% CI
Weight (%)
MCS 0.77 (0.30, 1.97) 4.83
ALSPAC(G1) 1.04 (0.57, 1.89) 11.75
NS 0.67 (0.25, 1.80) 4.40
NCDS 1.38 (0.74, 2.59) 10.59
USOC 0.86 (0.59, 1.25) 26.61
ELSA 0.73 (0.41, 1.30) 12.44
GS 0.98 (0.43, 2.21) 6.41
ALSPAC(G0) 0.60 (0.40, 0.91) 22.98
Overall 0.83 (0.67, 1.02)
Heterogeneity: τ^2 = 0.01, I^2 = 6.23%, H^2 = 1.07
Test of θ = 0: z = -1.77, p = 0.08
1/4
1/2
1
2
Random-effects REML model

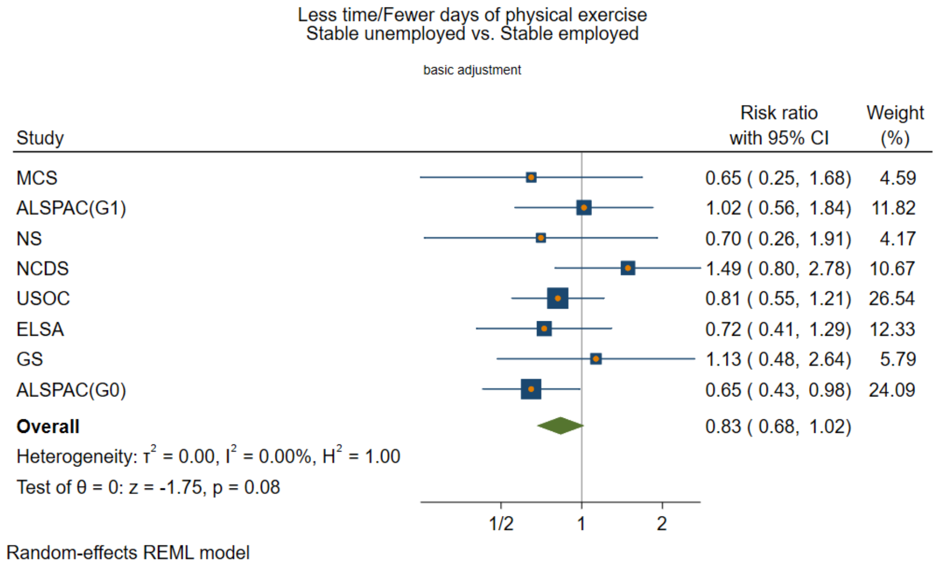
Less time/Fewer days of physical exercise
Stable unemployed vs. Stable employed
basic adjustment
Study
Risk ratio with 95% CI
Weight (%)
MCS 0.65 (0.25, 1.68) 4.59
ALSPAC(G1) 1.02 (0.56, 1.84) 11.82
NS 0.70 (0.26, 1.91) 4.17
NCDS 1.49 (0.80, 2.78) 10.67
USOC 0.81 (0.55, 1.21) 26.54
ELSA 0.72 (0.41, 1.29) 12.33
GS 1.13 (0.48, 2.64) 5.79
ALSPAC(G0) 0.65 (0.43, 0.98) 24.09
Overall 0.83 (0.68, 1.02)
Heterogeneity: τ^2 = 0.00, I^2 = 0.00%, H^2 = 1.00
Test of θ = 0: z = -1.75, p = 0.08
1/2
1
2
Random-effects REML model

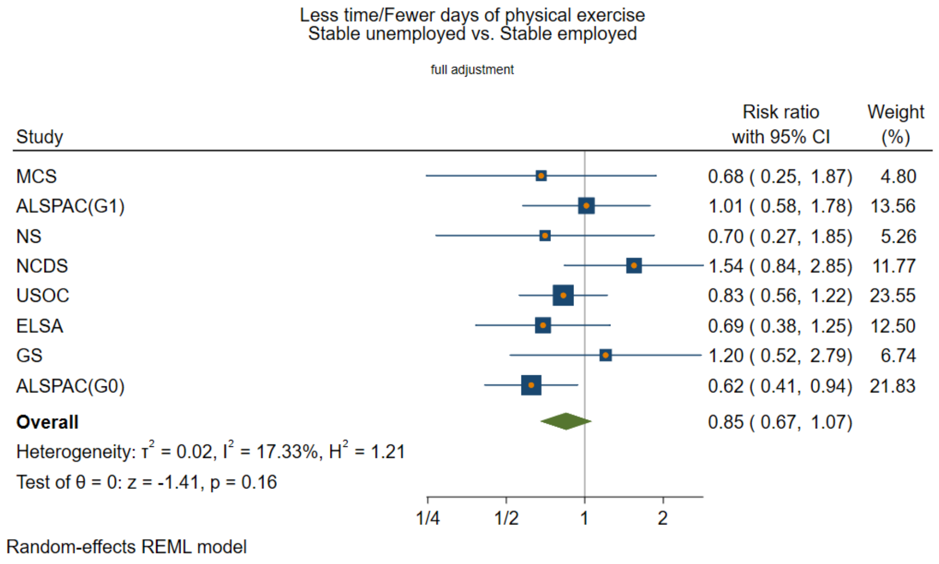
Less time/Fewer days of physical exercise
Stable unemployed vs. Stable employed
full adjustment
Study
Risk ratio with 95% CI
Weight (%)
MCS 0.68 (0.25, 1.87) 4.80
ALSPAC(G1) 1.01 (0.58, 1.78) 13.56
NS 0.70 (0.27, 1.85) 5.26
NCDS 1.54 (0.84, 2.85) 11.77
USOC 0.83 (0.56, 1.22) 23.55
ELSA 0.69 (0.38, 1.25) 12.50
GS 1.20 (0.52, 2.79) 6.74
ALSPAC(G0) 0.62 (0.41, 0.94) 21.83
Overall 0.85 (0.67, 1.07)
Heterogeneity: τ² = 0.02, I² = 17.33%, H² = 1.21
Test of θ = 0: z = -1.41, p = 0.16
1/4
1/2
1
2
Random-effects REML model

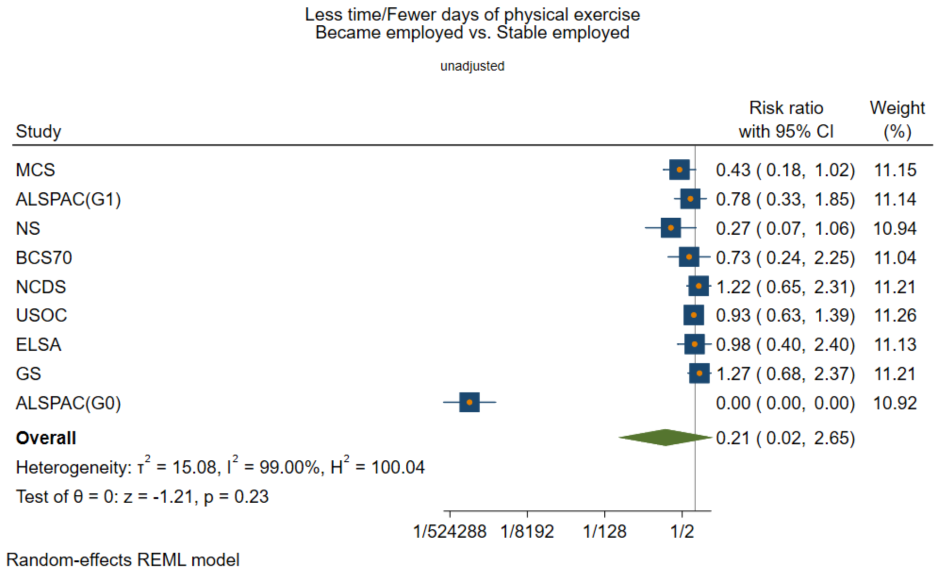
Less time/Fewer days of physical exercise
Became employed vs. Stable employed
unadjusted
Study
Risk ratio with 95% CI
Weight (%)
MCS 0.43 (0.18, 1.02) 11.15
ALSPAC(G1) 0.78 (0.33, 1.85) 11.14
NS 0.27 (0.07, 1.06) 10.94
BCS70 0.73 (0.24, 2.25) 11.04
NCDS 1.22 (0.65, 2.31) 11.21
USOC 0.93 (0.63, 1.39) 11.26
ELSA 0.98 (0.40, 2.40) 11.13
GS 1.27 (0.68, 2.37) 11.21
ALSPAC(G0) 0.00 (0.00, 0.00) 10.92
Overall 0.21 (0.02, 2.65)
Heterogeneity: τ² = 15.08, I² = 99.00%, H² = 100.04
Test of θ = 0: z = -1.21, p = 0.23
1/524288
1/8192
1/128
1/2
Random-effects REML model

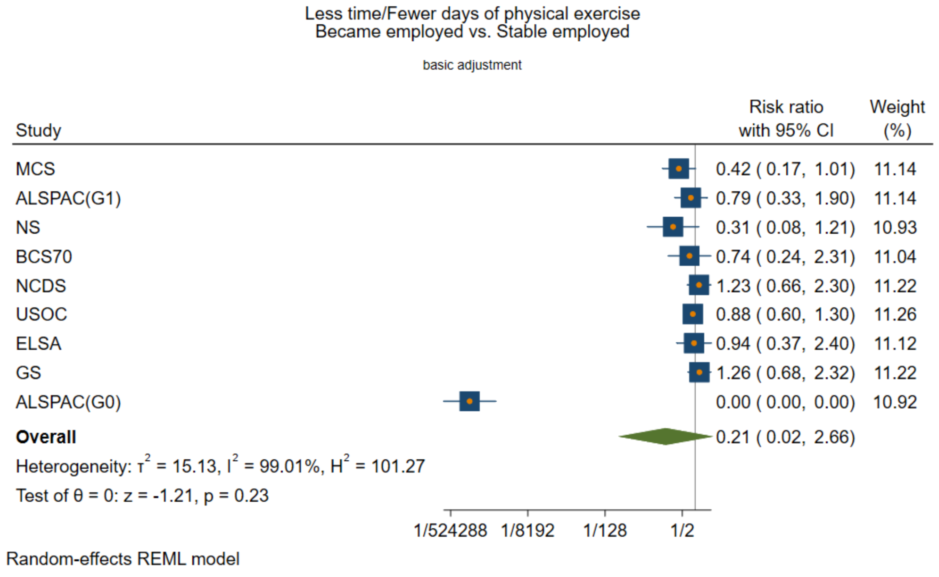
Less time/Fewer days of physical exercise
Became employed vs. Stable employed
basic adjustment
Study
Risk ratio with 95% CI
Weight (%)
MCS 0.42 (0.17, 1.01) 11.14
ALSPAC(G1) 0.79 (0.33, 1.90) 11.14
NS 0.31 (0.08, 1.21) 10.93
BCS70 0.74 (0.24, 2.31) 11.04
NCDS 1.23 (0.66, 2.30) 11.22
USOC 0.88 (0.60, 1.30) 11.26
ELSA 0.94 (0.37, 2.40) 11.12
GS 1.26 (0.68, 2.32) 11.22
ALSPAC(G0) 0.00 (0.00, 0.00) 10.92
Overall 0.21 (0.02, 2.66)
Heterogeneity: τ² = 15.13, I² = 99.01%, H² = 101.27
Test of θ = 0: z = -1.21, p = 0.23
1/524288
1/8192
1/128
1/2
Random-effects REML model

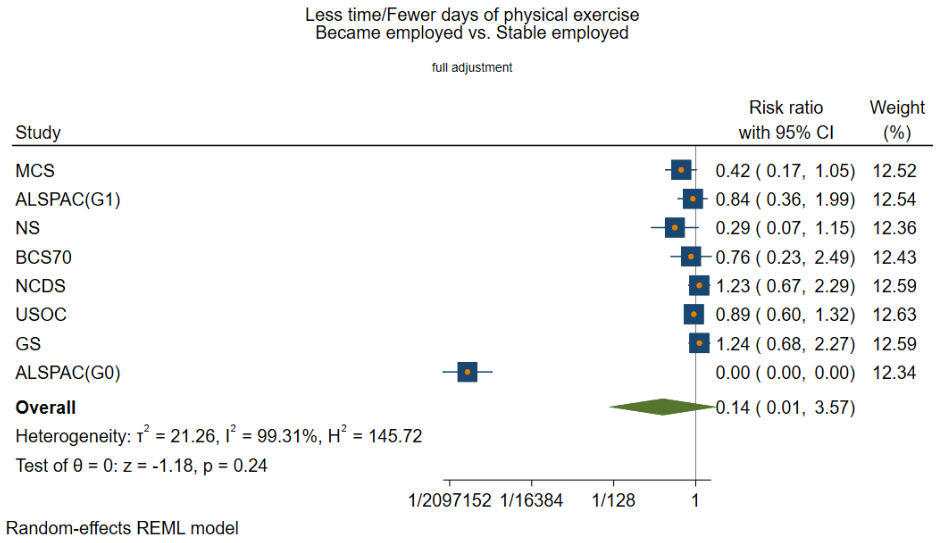
Less time/Fewer days of physical exercise
Became employed vs. Stable employed
full adjustment
Study
Risk ratio with 95% CI
Weight (%)
MCS 0.42 (0.17, 1.05) 12.52
ALSPAC(G1) 0.84 (0.36, 1.99) 12.54
NS 0.29 (0.07, 1.15) 12.36
BCS70 0.76 (0.23, 2.49) 12.43
NCDS 1.23 (0.67, 2.29) 12.59
USOC 0.89 (0.60, 1.32) 12.63
GS 1.24 (0.68, 2.27) 12.59
ALSPAC(G0) 0.00 (0.00, 0.00) 12.34
Overall 0.14 (0.01, 3.57)
Heterogeneity: τ² = 21.26, I² = 99.31%, H² = 145.72
Test of θ = 0: z = -1.18, p = 0.24
1/2097152
1/16384
1/128
1
Random-effects REML model

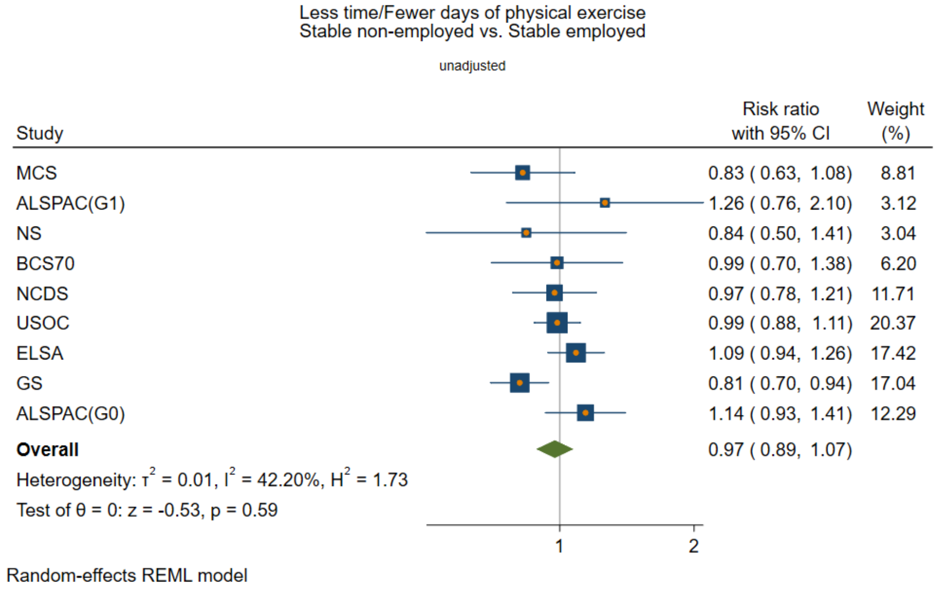
Less time/Fewer days of physical exercise
Stable non-employed vs. Stable employed
unadjusted
Study
Risk ratio with 95% CI
Weight (%)
MCS 0.83 (0.63, 1.08) 8.81
ALSPAC(G1) 1.26 (0.76, 2.10) 3.12
NS 0.84 (0.50, 1.41) 3.04
BCS70 0.99 (0.70, 1.38) 6.20
NCDS 0.97 (0.78, 1.21) 11.71
USOC 0.99 (0.88, 1.11) 20.37
ELSA 1.09 (0.94, 1.26) 17.42
GS 0.81 (0.70, 0.94) 17.04
ALSPAC(G0) 1.14 (0.93, 1.41) 12.29
Overall 0.97 (0.89, 1.07)
Heterogeneity: τ² = 0.01, I² = 42.20%, H² = 1.73
Test of θ = 0: z = -0.53, p = 0.59
1
2
Random-effects REML model

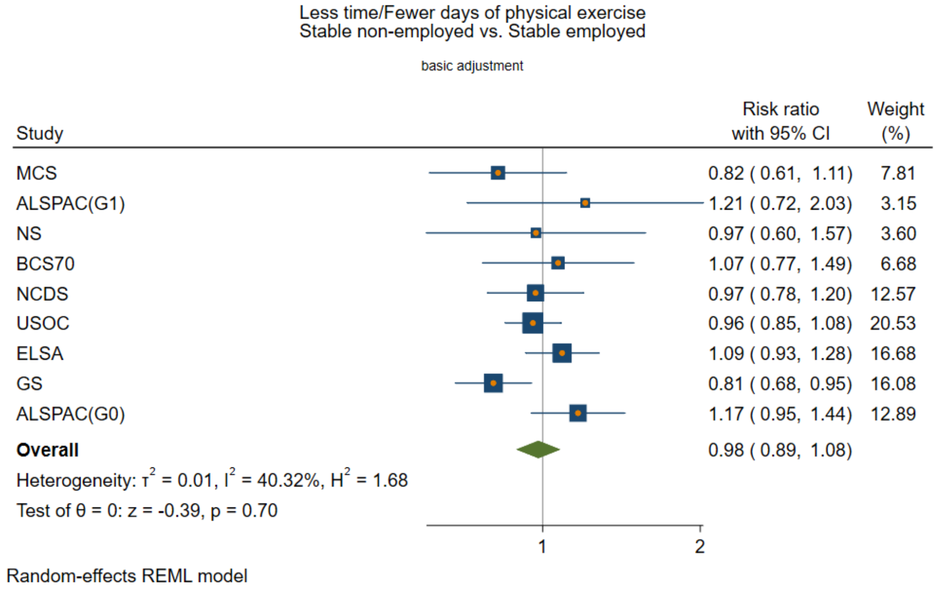
Less time/Fewer days of physical exercise
Stable non-employed vs. Stable employed
basic adjustment
Study
Risk ratio with 95% CI
Weight (%)
MCS 0.82 (0.61, 1.11) 7.81
ALSPAC(G1) 1.21 (0.72, 2.03) 3.15
NS 0.97 (0.60, 1.57) 3.60
BCS70 1.07 (0.77, 1.49) 6.68
NCDS 0.97 (0.78, 1.20) 12.57
USOC 0.96 (0.85, 1.08) 20.53
ELSA 1.09 (0.93, 1.28) 16.68
GS 0.81 (0.68, 0.95) 16.08
ALSPAC(G0) 1.17 (0.95, 1.44) 12.89
Overall 0.98 (0.89, 1.08)
Heterogeneity: τ² = 0.01, I² = 40.32%, H² = 1.68
Test of θ = 0: z = -0.39, p = 0.70
1
2
Random-effects REML model

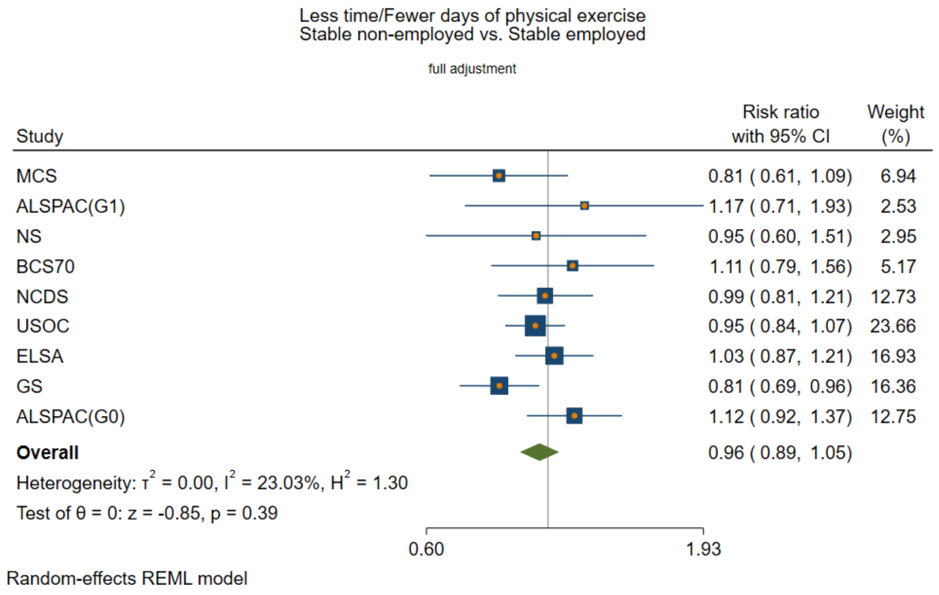
Less time/Fewer days of physical exercise
Stable non-employed vs. Stable employed
full adjustment
Study
Risk ratio with 95% CI
Weight (%)
MCS 0.81 (0.61, 1.09) 6.94
ALSPAC(G1) 1.17 (0.71, 1.93) 2.53
NS 0.95 (0.60, 1.51) 2.95
BCS70 1.11 (0.79, 1.56) 5.17
NCDS 0.99 (0.81, 1.21) 12.73
USOC 0.95 (0.84, 1.07) 23.66
ELSA 1.03 (0.87, 1.21) 16.93
GS 0.81 (0.69, 0.96) 16.36
ALSPAC(G0) 1.12 (0.92, 1.37) 12.75
Overall 0.96 (0.89, 1.05)
Heterogeneity: τ² = 0.00, I² = 23.03%, H² = 1.30
Test of θ = 0: z = -0.85, p = 0.39
0.60
1.93
Random-effects REML model

Figure set 6: More time/days of physical exercise

Commented [MJ2]:

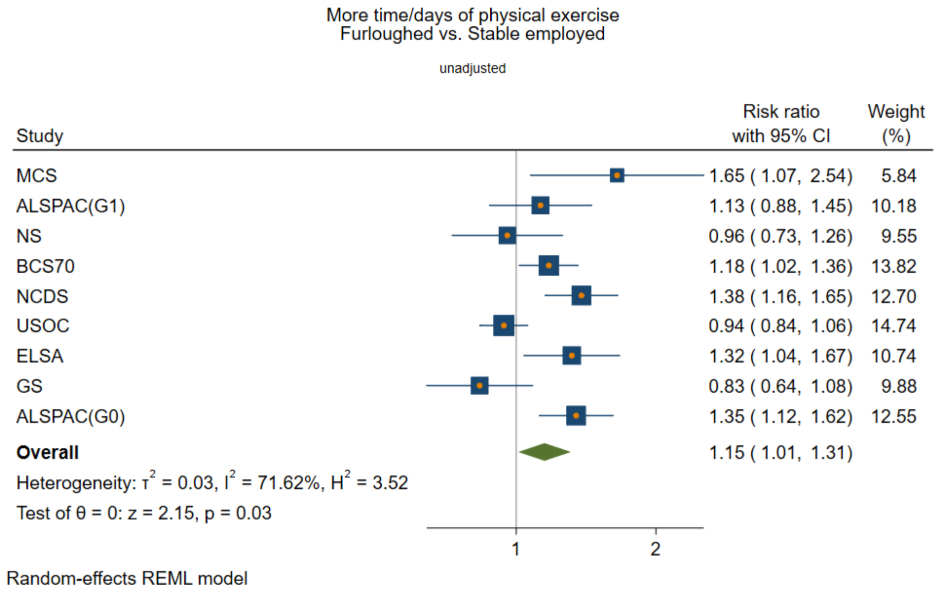

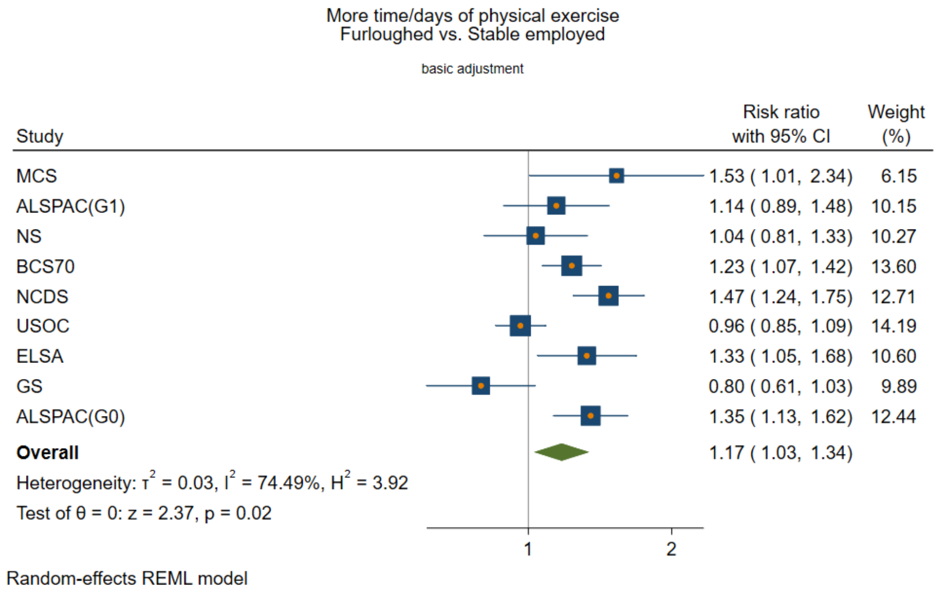

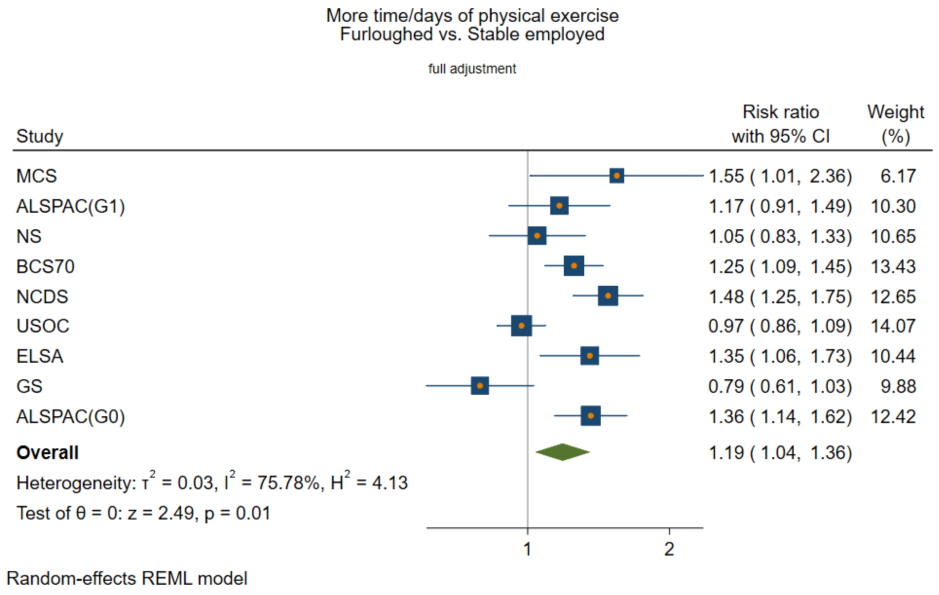
More time/days of physical exercise
Furloughed vs. Stable employed
full adjustment
Study
Risk ratio with 95% CI
Weight (%)
MCS 1.55 (1.01, 2.36) 6.17
ALSPAC(G1) 1.17 (0.91, 1.49) 10.30
NS 1.05 (0.83, 1.33) 10.65
BCS70 1.25 (1.09, 1.45) 13.43
NCDS 1.48 (1.25, 1.75) 12.65
USOC 0.97 (0.86, 1.09) 14.07
ELSA 1.35 (1.06, 1.73) 10.44
GS 0.79 (0.61, 1.03) 9.88
ALSPAC(G0) 1.36 (1.14, 1.62) 12.42
Overall 1.19 (1.04, 1.36)
Heterogeneity: τ² = 0.03, I² = 75.78%, H² = 4.13
Test of θ = 0: z = 2.49, p = 0.01
1
2
Random-effects REML model

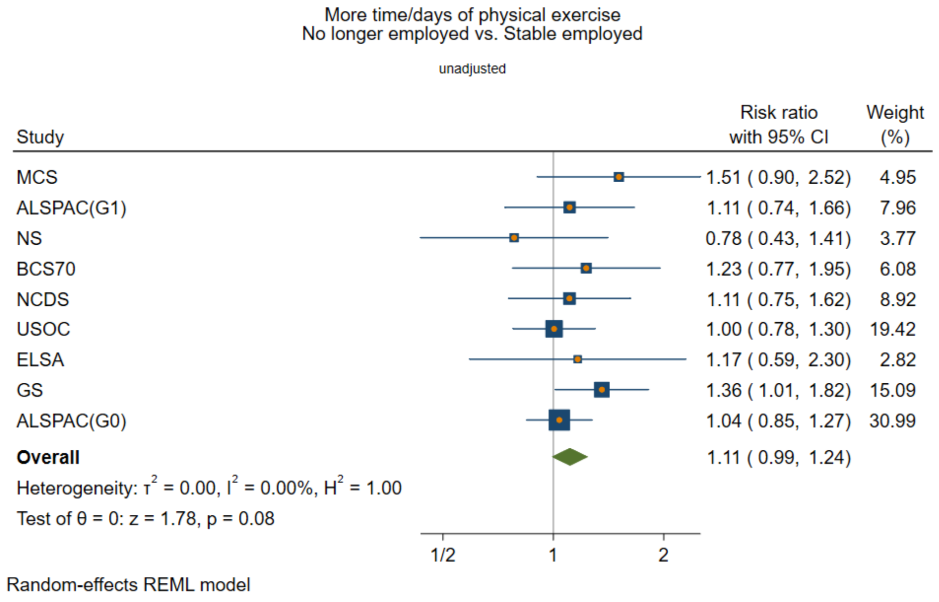
More time/days of physical exercise
No longer employed vs. Stable employed
unadjusted
Study
Risk ratio with 95% CI
Weight (%)
MCS 1.51 (0.90, 2.52) 4.95
ALSPAC(G1) 1.11 (0.74, 1.66) 7.96
NS 0.78 (0.43, 1.41) 3.77
BCS70 1.23 (0.77, 1.95) 6.08
NCDS 1.11 (0.75, 1.62) 8.92
USOC 1.00 (0.78, 1.30) 19.42
ELSA 1.17 (0.59, 2.30) 2.82
GS 1.36 (1.01, 1.82) 15.09
ALSPAC(G0) 1.04 (0.85, 1.27) 30.99
Overall 1.11 (0.99, 1.24)
Heterogeneity: τ² = 0.00, I² = 0.00%, H² = 1.00
Test of θ = 0: z = 1.78, p = 0.08
1/2
1
2
Random-effects REML model

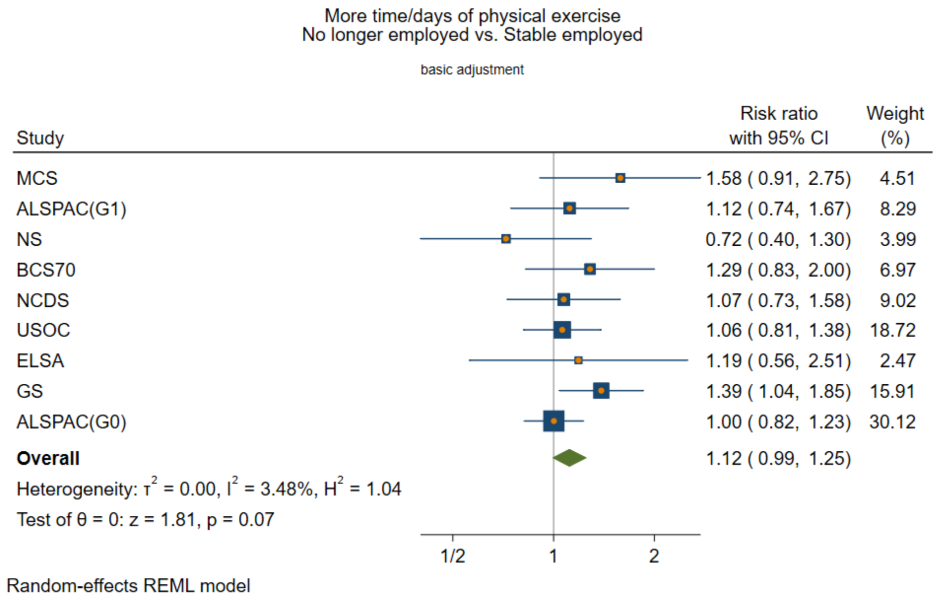

More time/days of physical exercise
No longer employed vs. Stable employed
basic adjustment
Study
Risk ratio with 95% CI
Weight (%)
MCS 1.58 (0.91, 2.75) 4.51
ALSPAC(G1) 1.12 (0.74, 1.67) 8.29
NS 0.72 (0.40, 1.30) 3.99
BCS70 1.29 (0.83, 2.00) 6.97
NCDS 1.07 (0.73, 1.58) 9.02
USOC 1.06 (0.81, 1.38) 18.72
ELSA 1.19 (0.56, 2.51) 2.47
GS 1.39 (1.04, 1.85) 15.91
ALSPAC(G0) 1.00 (0.82, 1.23) 30.12
Overall 1.12 (0.99, 1.25)
Heterogeneity: τ² = 0.00, I² = 3.48%, H² = 1.04
Test of θ = 0: z = 1.81, p = 0.07
1/2
1
2
Random-effects REML model

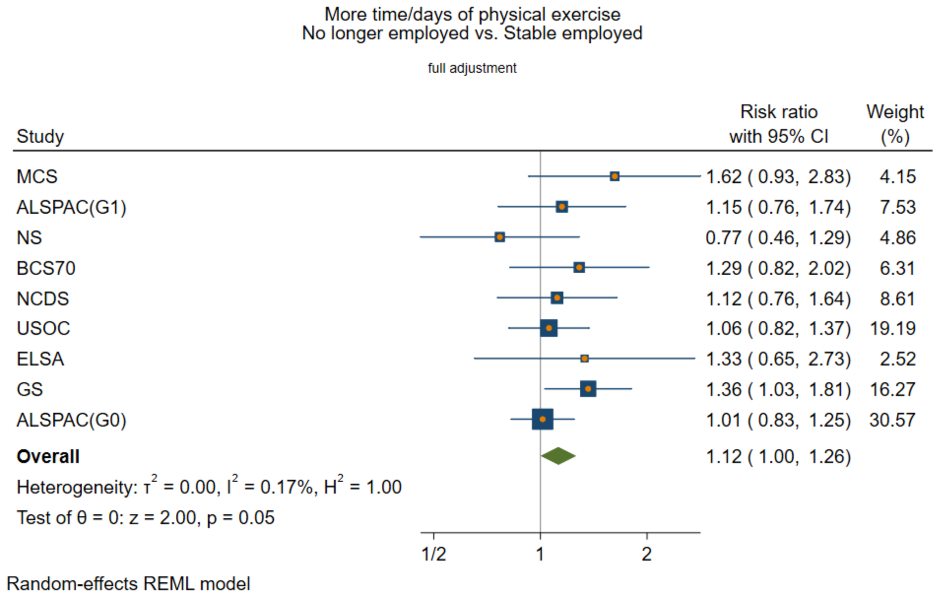

More time/days of physical exercise
No longer employed vs. Stable employed
full adjustment
Study
Risk ratio with 95% CI
Weight (%)
MCS 1.62 (0.93, 2.83) 4.15
ALSPAC(G1) 1.15 (0.76, 1.74) 7.53
NS 0.77 (0.46, 1.29) 4.86
BCS70 1.29 (0.82, 2.02) 6.31
NCDS 1.12 (0.76, 1.64) 8.61
USOC 1.06 (0.82, 1.37) 19.19
ELSA 1.33 (0.65, 2.73) 2.52
GS 1.36 (1.03, 1.81) 16.27
ALSPAC(G0) 1.01 (0.83, 1.25) 30.57
Overall 1.12 (1.00, 1.26)
Heterogeneity: τ² = 0.00, I² = 0.17%, H² = 1.00
Test of θ = 0: z = 2.00, p = 0.05
1/2
1
2
Random-effects REML model

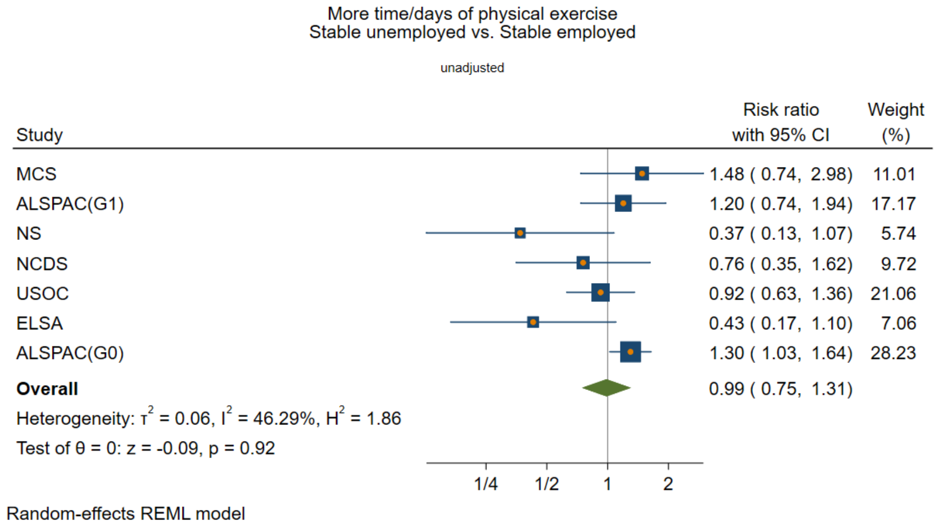

More time/days of physical exercise
Stable unemployed vs. Stable employed
unadjusted
Study
Risk ratio with 95% CI
Weight (%)
MCS 1.48 (0.74, 2.98) 11.01
ALSPAC(G1) 1.20 (0.74, 1.94) 17.17
NS 0.37 (0.13, 1.07) 5.74
NCDS 0.76 (0.35, 1.62) 9.72
USOC 0.92 (0.63, 1.36) 21.06
ELSA 0.43 (0.17, 1.10) 7.06
ALSPAC(G0) 1.30 (1.03, 1.64) 28.23
Overall 0.99 (0.75, 1.31)
Heterogeneity: τ² = 0.06, I² = 46.29%, H² = 1.86
Test of θ = 0: z = -0.09, p = 0.92
1/4
1/2
1
2
Random-effects REML model

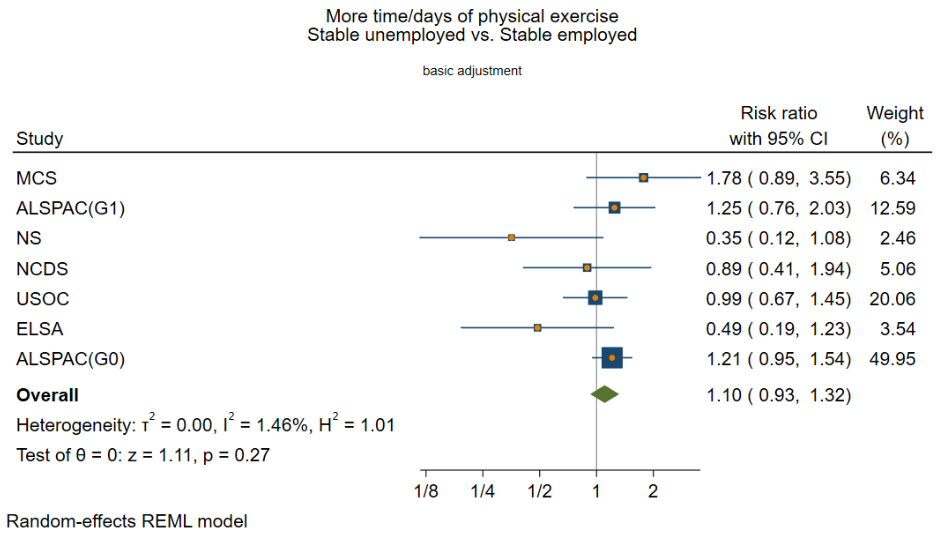
More time/days of physical exercise
Stable unemployed vs. Stable employed
basic adjustment
Study
Risk ratio with 95% CI
Weight (%)
MCS 1.78 (0.89, 3.55) 6.34
ALSPAC(G1) 1.25 (0.76, 2.03) 12.59
NS 0.35 (0.12, 1.08) 2.46
NCDS 0.89 (0.41, 1.94) 5.06
USOC 0.99 (0.67, 1.45) 20.06
ELSA 0.49 (0.19, 1.23) 3.54
ALSPAC(G0) 1.21 (0.95, 1.54) 49.95
Overall 1.10 (0.93, 1.32)
Heterogeneity: τ² = 0.00, I² = 1.46%, H² = 1.01
Test of θ = 0: z = 1.11, p = 0.27
1/8
1/4
1/2
1
2
Random-effects REML model

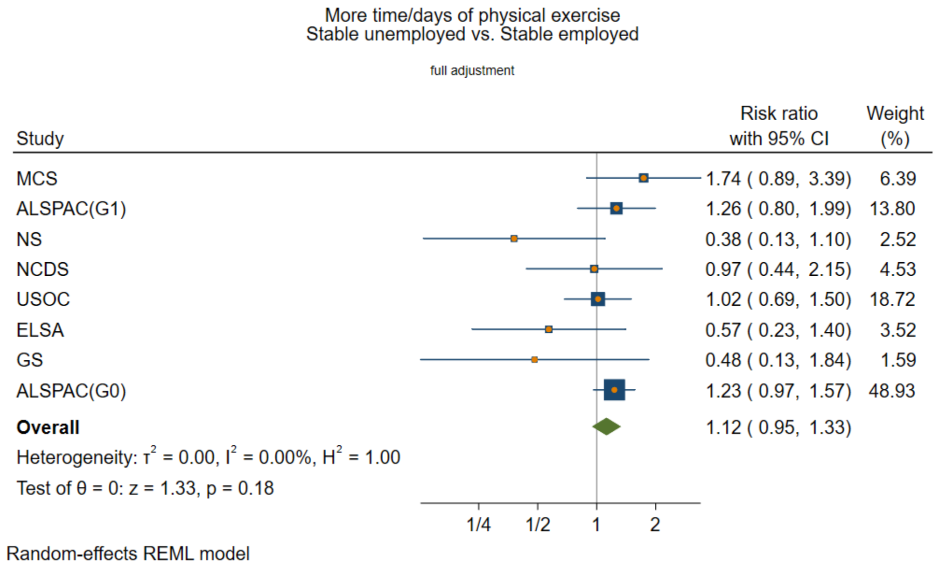
More time/days of physical exercise
Stable unemployed vs. Stable employed
full adjustment
Study
Risk ratio with 95% CI
Weight (%)
MCS 1.74 (0.89, 3.39) 6.39
ALSPAC(G1) 1.26 (0.80, 1.99) 13.80
NS 0.38 (0.13, 1.10) 2.52
NCDS 0.97 (0.44, 2.15) 4.53
USOC 1.02 (0.69, 1.50) 18.72
ELSA 0.57 (0.23, 1.40) 3.52
GS 0.48 (0.13, 1.84) 1.59
ALSPAC(G0) 1.23 (0.97, 1.57) 48.93
Overall 1.12 (0.95, 1.33)
Heterogeneity: τ² = 0.00, I² = 0.00%, H² = 1.00
Test of θ = 0: z = 1.33, p = 0.18
1/4
1/2
1
2
Random-effects REML model

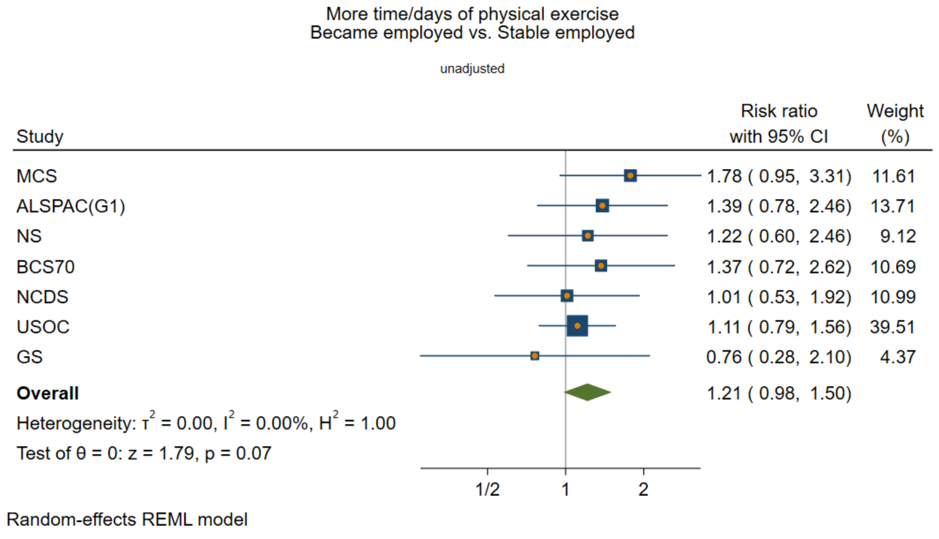
More time/days of physical exercise
Became employed vs. Stable employed
unadjusted
Study
Risk ratio with 95% CI
Weight (%)
MCS 1.78 (0.95, 3.31) 11.61
ALSPAC(G1) 1.39 (0.78, 2.46) 13.71
NS 1.22 (0.60, 2.46) 9.12
BCS70 1.37 (0.72, 2.62) 10.69
NCDS 1.01 (0.53, 1.92) 10.99
USOC 1.11 (0.79, 1.56) 39.51
GS 0.76 (0.28, 2.10) 4.37
Overall 1.21 (0.98, 1.50)
Heterogeneity: τ² = 0.00, I² = 0.00%, H² = 1.00
Test of θ = 0: z = 1.79, p = 0.07
1/2
1
2
Random-effects REML model

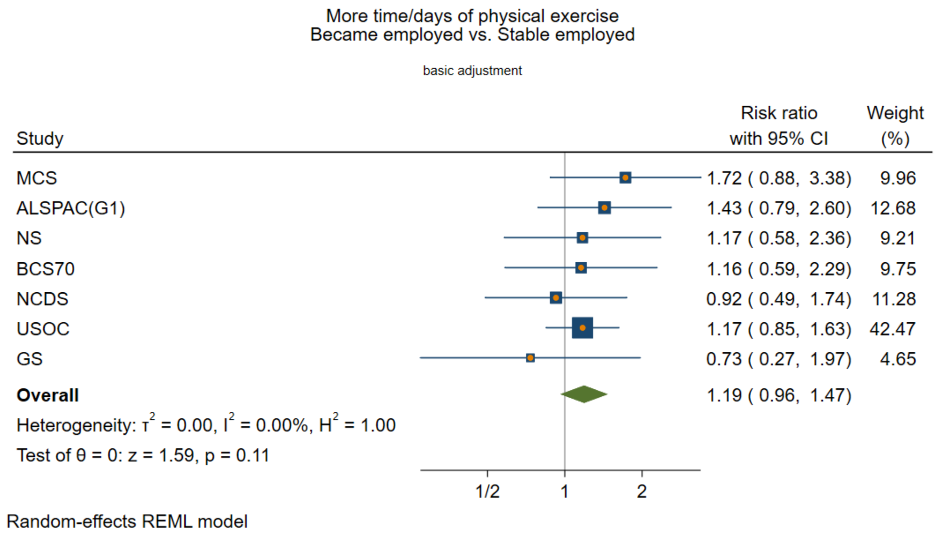
More time/days of physical exercise
Became employed vs. Stable employed
basic adjustment
Study
Risk ratio with 95% CI
Weight (%)
MCS 1.72 (0.88, 3.38) 9.96
ALSPAC(G1) 1.43 (0.79, 2.60) 12.68
NS 1.17 (0.58, 2.36) 9.21
BCS70 1.16 (0.59, 2.29) 9.75
NCDS 0.92 (0.49, 1.74) 11.28
USOC 1.17 (0.85, 1.63) 42.47
GS 0.73 (0.27, 1.97) 4.65
Overall 1.19 (0.96, 1.47)
Heterogeneity: τ² = 0.00, I² = 0.00%, H² = 1.00
Test of θ = 0: z = 1.59, p = 0.11
1/2
1
2
Random-effects REML model

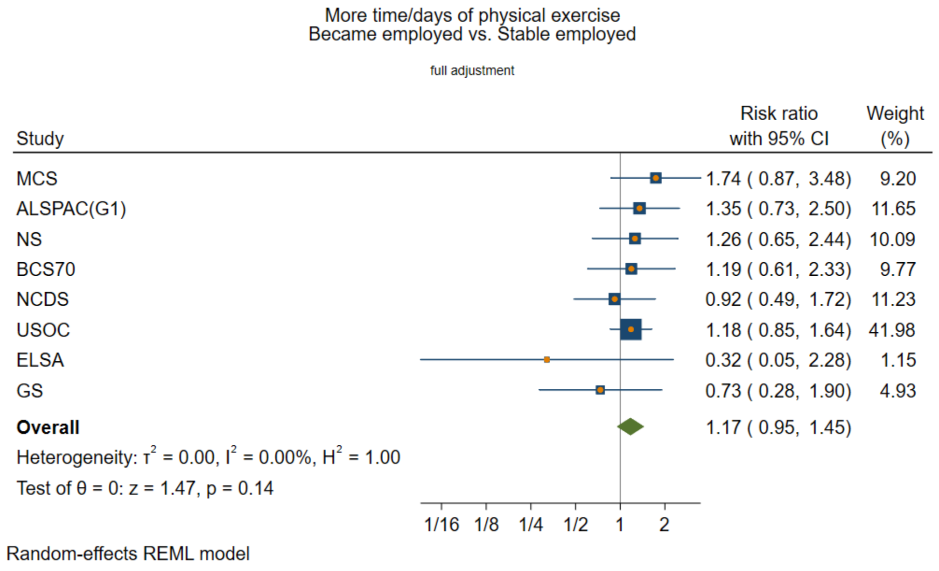
More time/days of physical exercise
Became employed vs. Stable employed
full adjustment
Study
Risk ratio with 95% CI
Weight (%)
MCS 1.74 (0.87, 3.48) 9.20
ALSPAC(G1) 1.35 (0.73, 2.50) 11.65
NS 1.26 (0.65, 2.44) 10.09
BCS70 1.19 (0.61, 2.33) 9.77
NCDS 0.92 (0.49, 1.72) 11.23
USOC 1.18 (0.85, 1.64) 41.98
ELSA 0.32 (0.05, 2.28) 1.15
GS 0.73 (0.28, 1.90) 4.93
Overall 1.17 (0.95, 1.45)
Heterogeneity: τ² = 0.00, I² = 0.00%, H² = 1.00
Test of θ = 0: z = 1.47, p = 0.14
1/16
1/8
1/4
1/2
1
2
Random-effects REML model

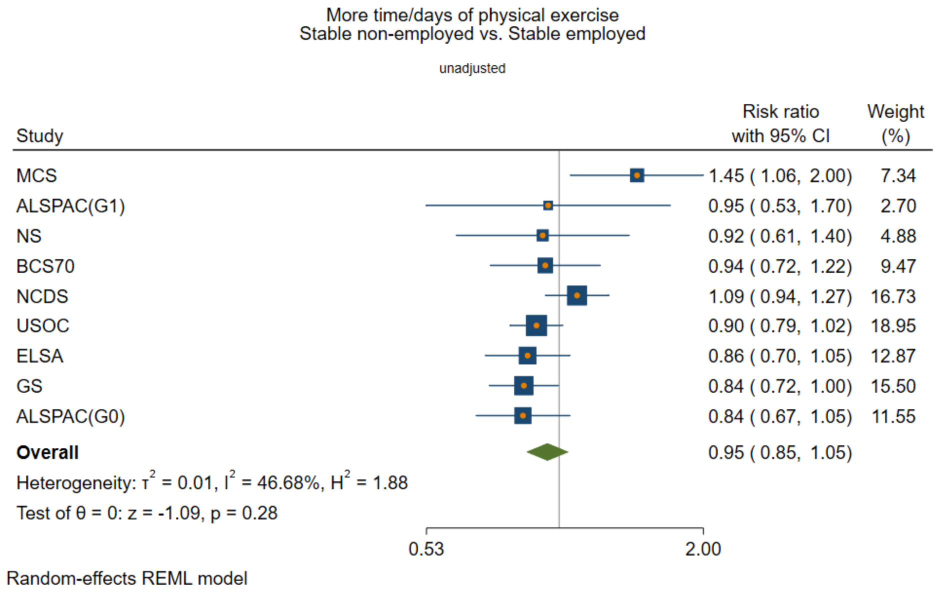
More time/days of physical exercise
Stable non-employed vs. Stable employed
unadjusted
Study
Risk ratio with 95% CI
Weight (%)
MCS 1.45 (1.06, 2.00) 7.34
ALSPAC(G1) 0.95 (0.53, 1.70) 2.70
NS 0.92 (0.61, 1.40) 4.88
BCS70 0.94 (0.72, 1.22) 9.47
NCDS 1.09 (0.94, 1.27) 16.73
USOC 0.90 (0.79, 1.02) 18.95
ELSA 0.86 (0.70, 1.05) 12.87
GS 0.84 (0.72, 1.00) 15.50
ALSPAC(G0) 0.84 (0.67, 1.05) 11.55
Overall 0.95 (0.85, 1.05)
Heterogeneity: τ² = 0.01, I² = 46.68%, H² = 1.88
Test of θ = 0: z = -1.09, p = 0.28
0.53
2.00
Random-effects REML model

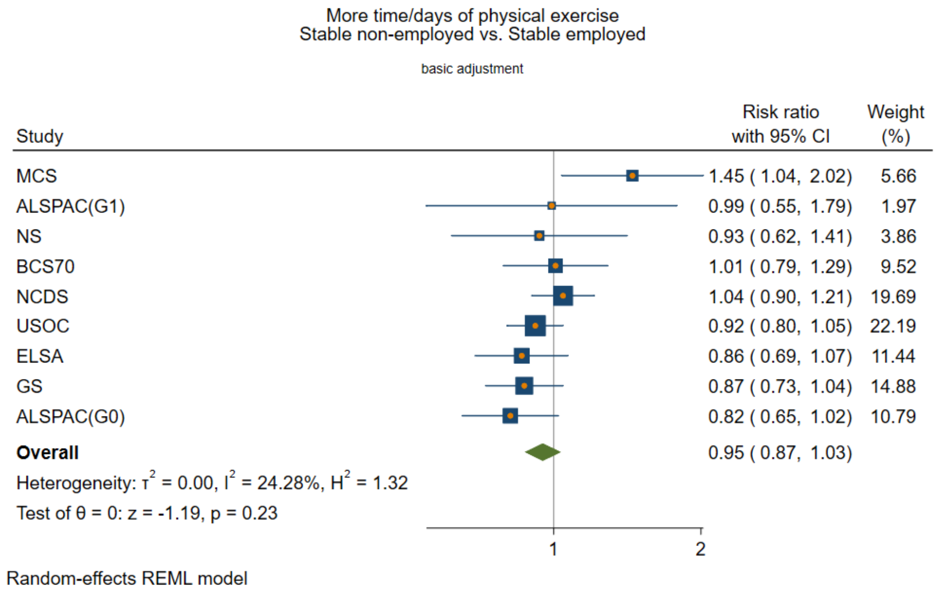
More time/days of physical exercise
Stable non-employed vs. Stable employed
basic adjustment
Study
Risk ratio with 95% CI
Weight (%)
MCS 1.45 (1.04, 2.02) 5.66
ALSPAC(G1) 0.99 (0.55, 1.79) 1.97
NS 0.93 (0.62, 1.41) 3.86
BCS70 1.01 (0.79, 1.29) 9.52
NCDS 1.04 (0.90, 1.21) 19.69
USOC 0.92 (0.80, 1.05) 22.19
ELSA 0.86 (0.69, 1.07) 11.44
GS 0.87 (0.73, 1.04) 14.88
ALSPAC(G0) 0.82 (0.65, 1.02) 10.79
Overall 0.95 (0.87, 1.03)
Heterogeneity: τ^2 = 0.00, I^2 = 24.28%, H^2 = 1.32
Test of θ = 0: z = -1.19, p = 0.23
1
2
Random-effects REML model

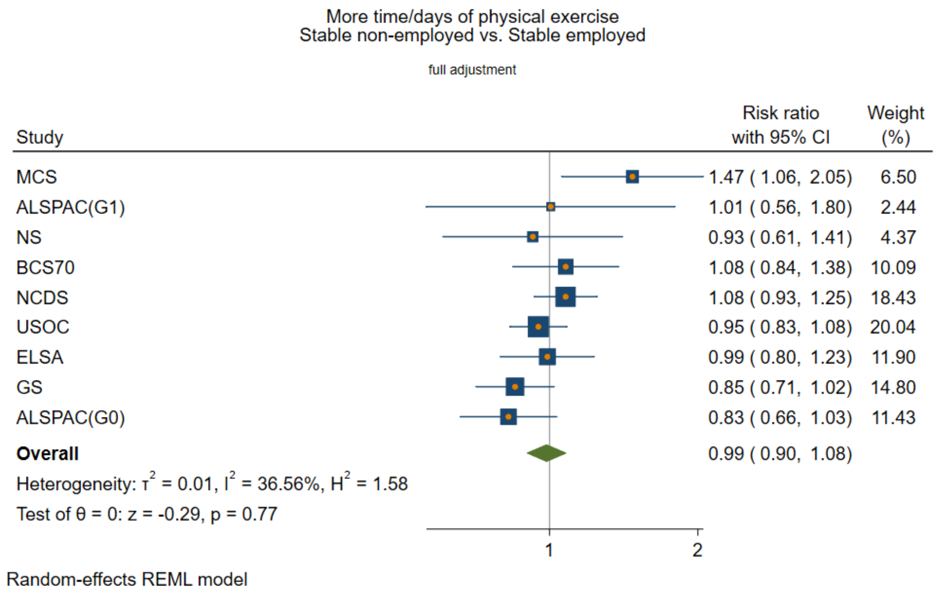
More time/days of physical exercise
Stable non-employed vs. Stable employed
full adjustment
Study
Risk ratio with 95% CI
Weight (%)
MCS 1.47 (1.06, 2.05) 6.50
ALSPAC(G1) 1.01 (0.56, 1.80) 2.44
NS 0.93 (0.61, 1.41) 4.37
BCS70 1.08 (0.84, 1.38) 10.09
NCDS 1.08 (0.93, 1.25) 18.43
USOC 0.95 (0.83, 1.08) 20.04
ELSA 0.99 (0.80, 1.23) 11.90
GS 0.85 (0.71, 1.02) 14.80
ALSPAC(G0) 0.83 (0.66, 1.03) 11.43
Overall 0.99 (0.90, 1.08)
Heterogeneity: τ^2 = 0.01, I^2 = 36.56%, H^2 = 1.58
Test of θ = 0: z = -0.29, p = 0.77
1
2
Random-effects REML model

Figure set 7: Sleeps outside 'Normal Range' (i.e. <6 or 9+ hours)

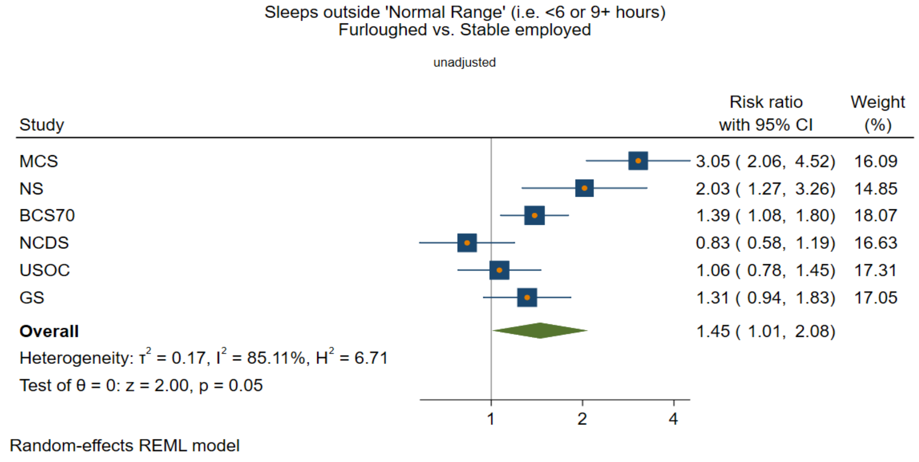

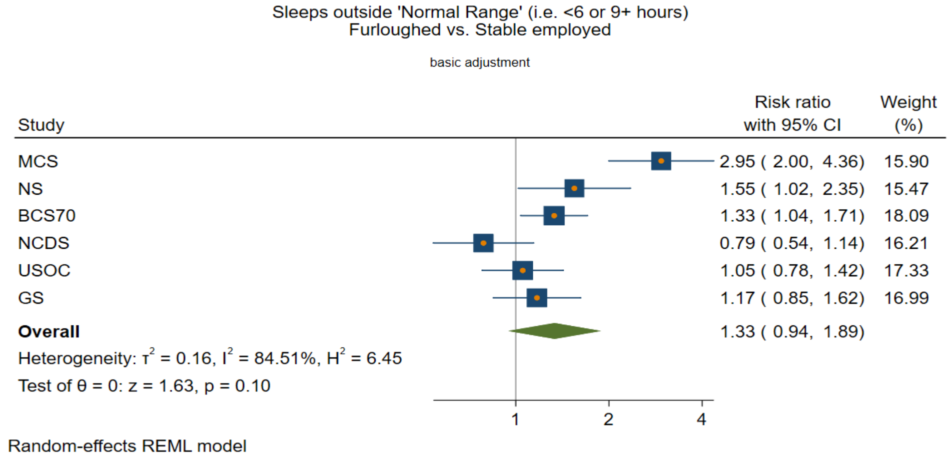

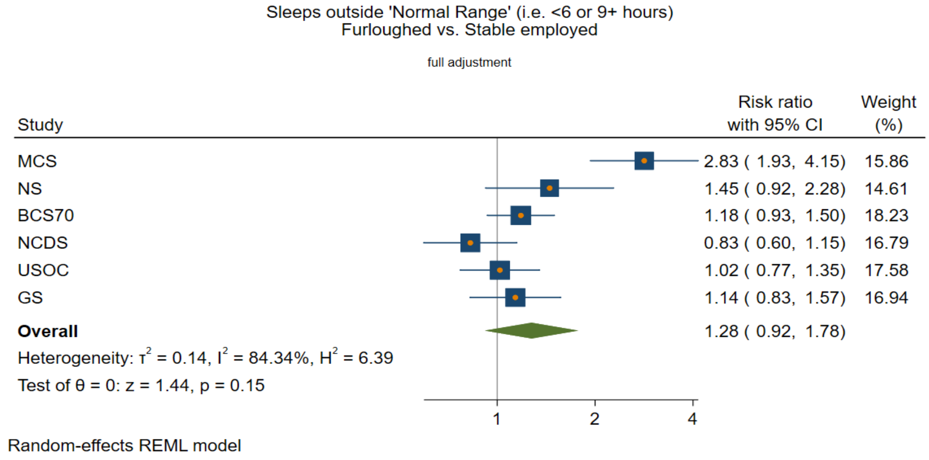

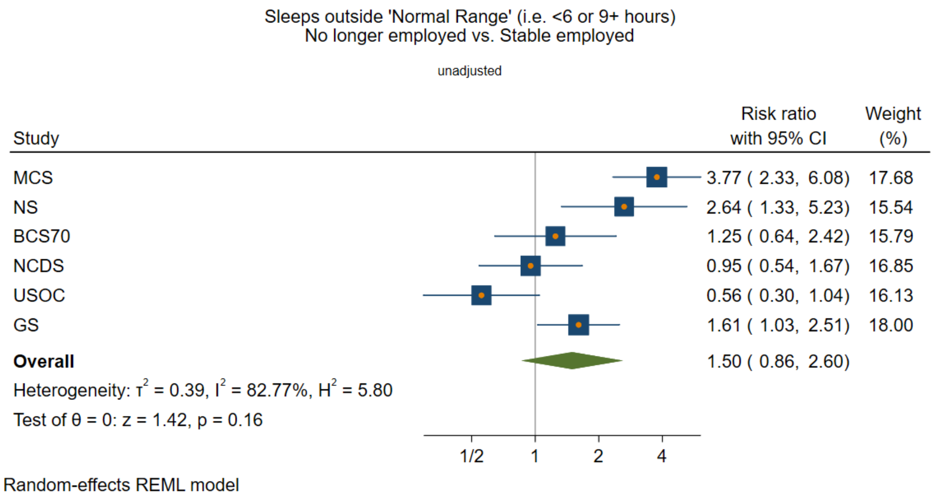
Sleeps outside 'Normal Range' (i.e. <6 or 9+ hours)
No longer employed vs. Stable employed
unadjusted
Study
Risk ratio with 95% CI
Weight (%)
MCS 3.77 (2.33, 6.08) 17.68
NS 2.64 (1.33, 5.23) 15.54
BCS70 1.25 (0.64, 2.42) 15.79
NCDS 0.95 (0.54, 1.67) 16.85
USOC 0.56 (0.30, 1.04) 16.13
GS 1.61 (1.03, 2.51) 18.00
Overall 1.50 (0.86, 2.60)
Heterogeneity: τ² = 0.39, I² = 82.77%, H² = 5.80
Test of θ = 0: z = 1.42, p = 0.16
1/2
1
2
4
Random-effects REML model

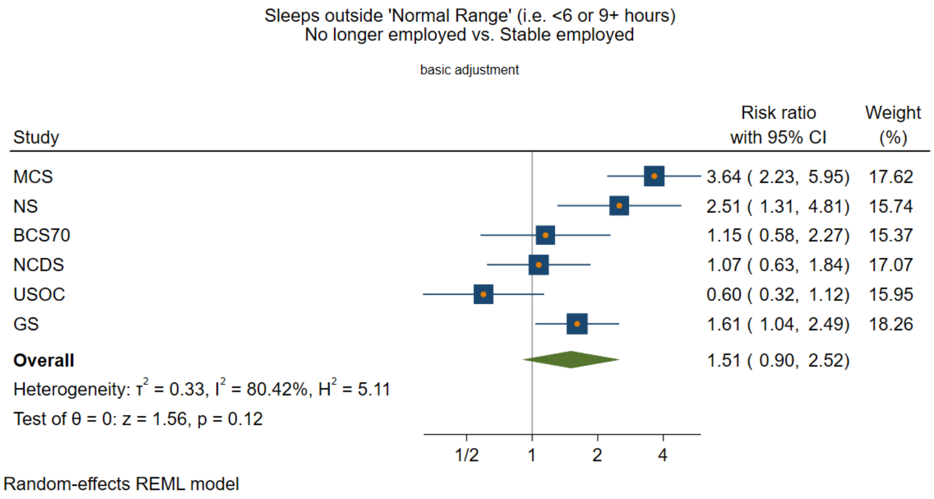
Sleeps outside 'Normal Range' (i.e. <6 or 9+ hours)
No longer employed vs. Stable employed
basic adjustment
Study
Risk ratio with 95% CI
Weight (%)
MCS 3.64 (2.23, 5.95) 17.62
NS 2.51 (1.31, 4.81) 15.74
BCS70 1.15 (0.58, 2.27) 15.37
NCDS 1.07 (0.63, 1.84) 17.07
USOC 0.60 (0.32, 1.12) 15.95
GS 1.61 (1.04, 2.49) 18.26
Overall 1.51 (0.90, 2.52)
Heterogeneity: τ² = 0.33, I² = 80.42%, H² = 5.11
Test of θ = 0: z = 1.56, p = 0.12
1/2
1
2
4
Random-effects REML model

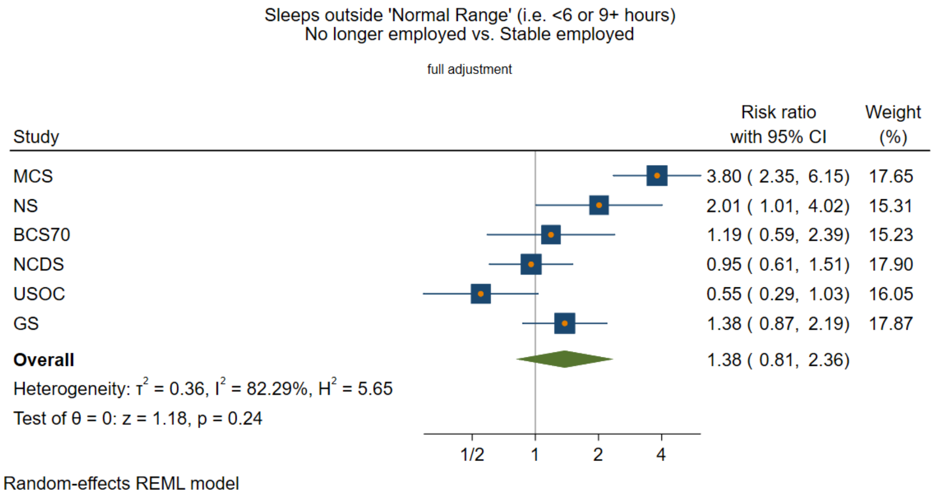
Sleeps outside 'Normal Range' (i.e. <6 or 9+ hours)
No longer employed vs. Stable employed
full adjustment
Study
Risk ratio with 95% CI
Weight (%)
MCS 3.80 (2.35, 6.15) 17.65
NS 2.01 (1.01, 4.02) 15.31
BCS70 1.19 (0.59, 2.39) 15.23
NCDS 0.95 (0.61, 1.51) 17.90
USOC 0.55 (0.29, 1.03) 16.05
GS 1.38 (0.87, 2.19) 17.87
Overall 1.38 (0.81, 2.36)
Heterogeneity: τ² = 0.36, I² = 82.29%, H² = 5.65
Test of θ = 0: z = 1.18, p = 0.24
1/2
1
2
4
Random-effects REML model

Sleeps outside 'Normal Range' (i.e. <6 or 9+ hours)
Stable unemployed vs. Stable employed

unadjusted

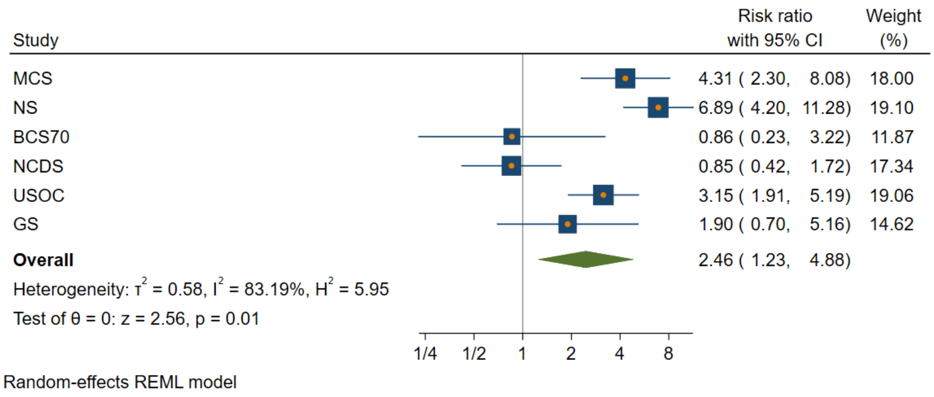

Sleeps outside 'Normal Range' (i.e. <6 or 9+ hours)
Stable unemployed vs. Stable employed

basic adjustment

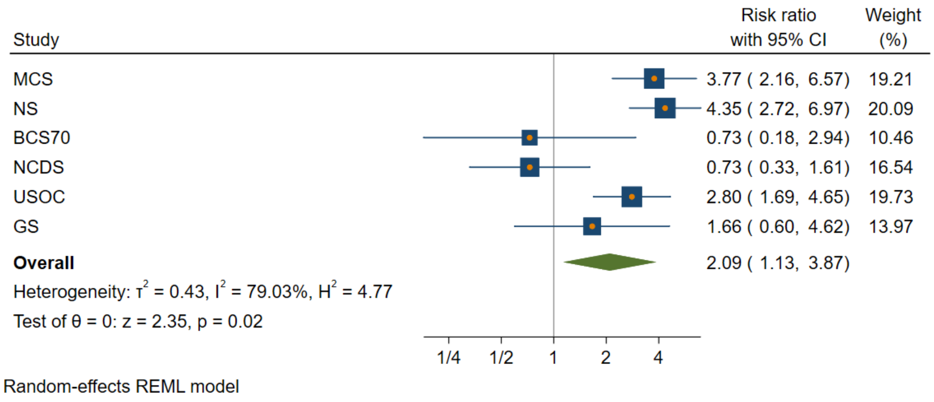

Sleeps outside 'Normal Range' (i.e. <6 or 9+ hours)
Stable unemployed vs. Stable employed

full adjustment

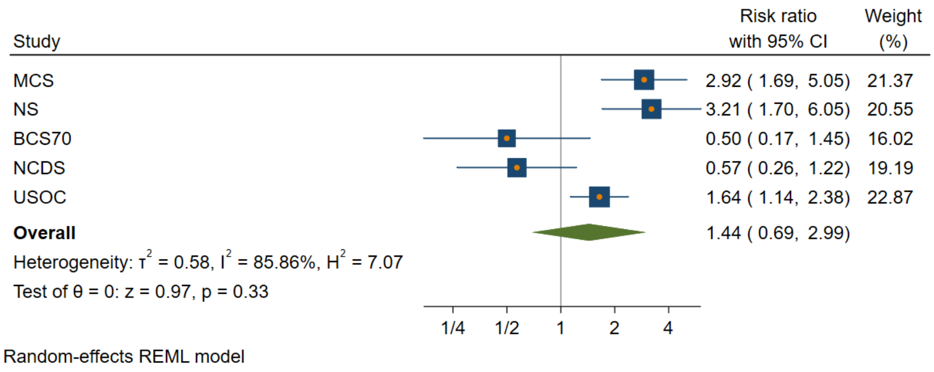

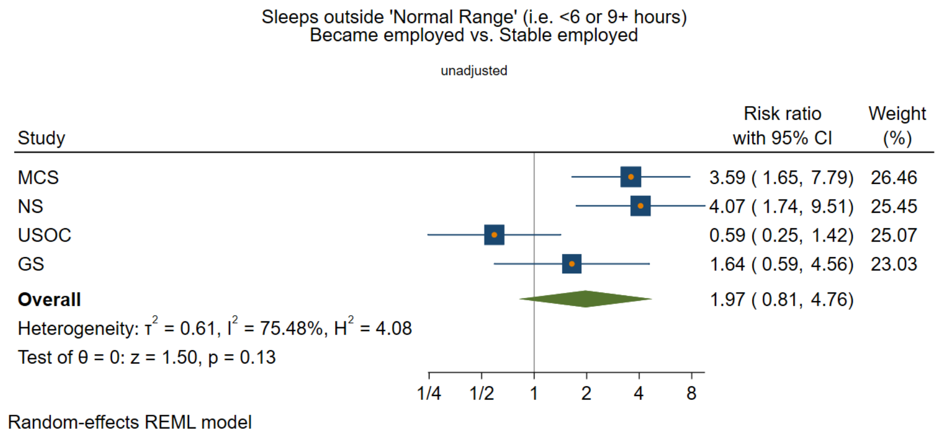

Sleeps outside 'Normal Range' (i.e. <6 or 9+ hours)
Became employed vs. Stable employed
unadjusted
Study
Risk ratio with 95% CI
Weight (%)
MCS 3.59 (1.65, 7.79) 26.46
NS 4.07 (1.74, 9.51) 25.45
USOC 0.59 (0.25, 1.42) 25.07
GS 1.64 (0.59, 4.56) 23.03
Overall 1.97 (0.81, 4.76)
Heterogeneity: τ² = 0.61, I² = 75.48%, H² = 4.08
Test of θ = 0: z = 1.50, p = 0.13
1/4 1/2 1 2 4 8
Random-effects REML model

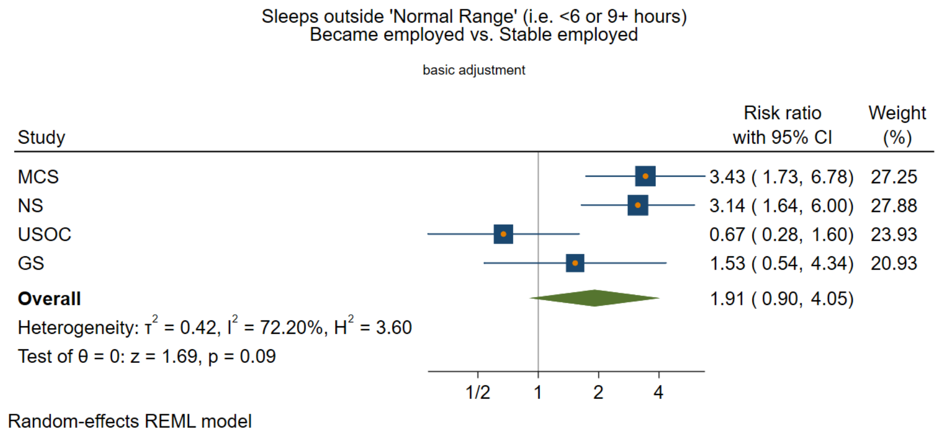

Sleeps outside 'Normal Range' (i.e. <6 or 9+ hours)
Became employed vs. Stable employed
basic adjustment
Study
Risk ratio with 95% CI
Weight (%)
MCS 3.43 (1.73, 6.78) 27.25
NS 3.14 (1.64, 6.00) 27.88
USOC 0.67 (0.28, 1.60) 23.93
GS 1.53 (0.54, 4.34) 20.93
Overall 1.91 (0.90, 4.05)
Heterogeneity: τ² = 0.42, I² = 72.20%, H² = 3.60
Test of θ = 0: z = 1.69, p = 0.09
1/2 1 2 4
Random-effects REML model

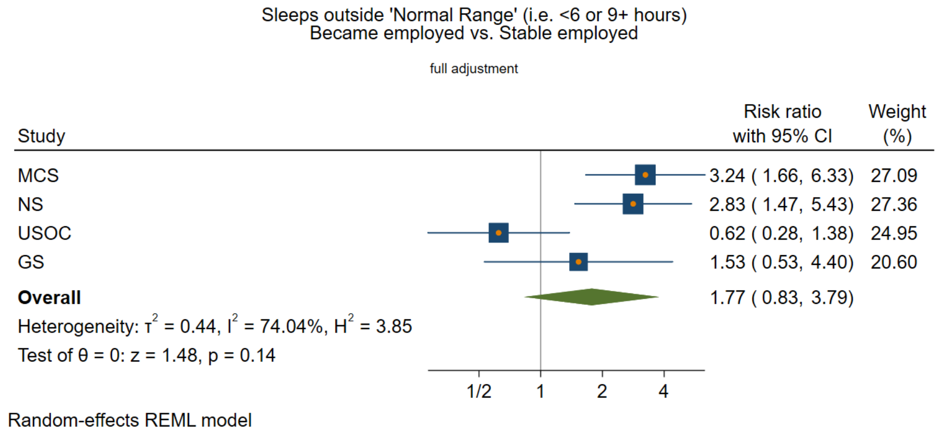

Sleeps outside 'Normal Range' (i.e. <6 or 9+ hours)
Became employed vs. Stable employed
full adjustment
Study
Risk ratio with 95% CI
Weight (%)
MCS 3.24 (1.66, 6.33) 27.09
NS 2.83 (1.47, 5.43) 27.36
USOC 0.62 (0.28, 1.38) 24.95
GS 1.53 (0.53, 4.40) 20.60
Overall 1.77 (0.83, 3.79)
Heterogeneity: τ² = 0.44, I² = 74.04%, H² = 3.85
Test of θ = 0: z = 1.48, p = 0.14
1/2 1 2 4
Random-effects REML model

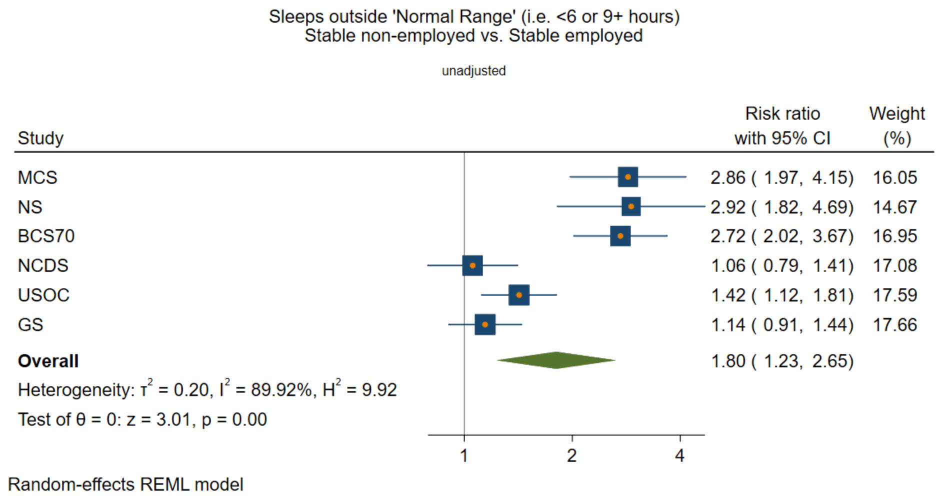
Sleeps outside 'Normal Range' (i.e. <6 or 9+ hours)
Stable non-employed vs. Stable employed
unadjusted
Study
Risk ratio with 95% CI
Weight (%)
MCS 2.86 (1.97, 4.15) 16.05
NS 2.92 (1.82, 4.69) 14.67
BCS70 2.72 (2.02, 3.67) 16.95
NCDS 1.06 (0.79, 1.41) 17.08
USOC 1.42 (1.12, 1.81) 17.59
GS 1.14 (0.91, 1.44) 17.66
Overall 1.80 (1.23, 2.65)
Heterogeneity: τ^2 = 0.20, I^2 = 89.92%, H^2 = 9.92
Test of θ = 0: z = 3.01, p = 0.00
1
2
4
Random-effects REML model

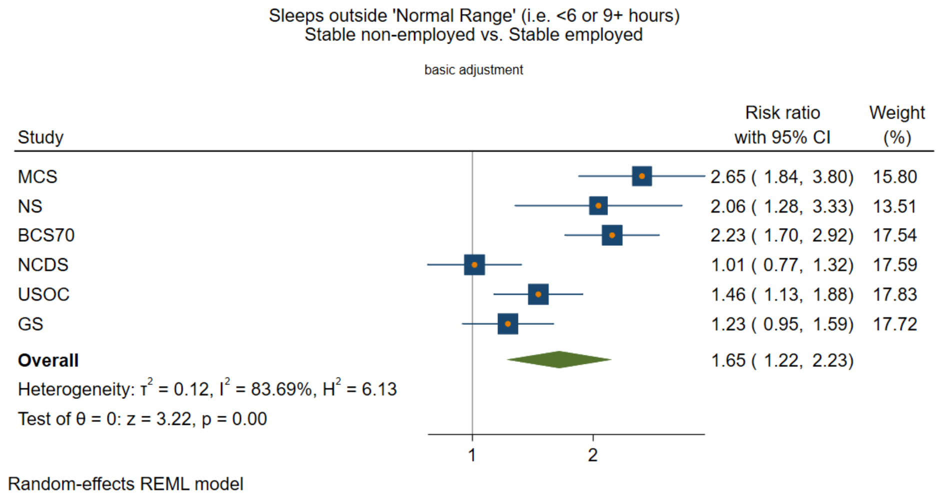
Sleeps outside 'Normal Range' (i.e. <6 or 9+ hours)
Stable non-employed vs. Stable employed
basic adjustment
Study
Risk ratio with 95% CI
Weight (%)
MCS 2.65 (1.84, 3.80) 15.80
NS 2.06 (1.28, 3.33) 13.51
BCS70 2.23 (1.70, 2.92) 17.54
NCDS 1.01 (0.77, 1.32) 17.59
USOC 1.46 (1.13, 1.88) 17.83
GS 1.23 (0.95, 1.59) 17.72
Overall 1.65 (1.22, 2.23)
Heterogeneity: τ^2 = 0.12, I^2 = 83.69%, H^2 = 6.13
Test of θ = 0: z = 3.22, p = 0.00
1
2
Random-effects REML model

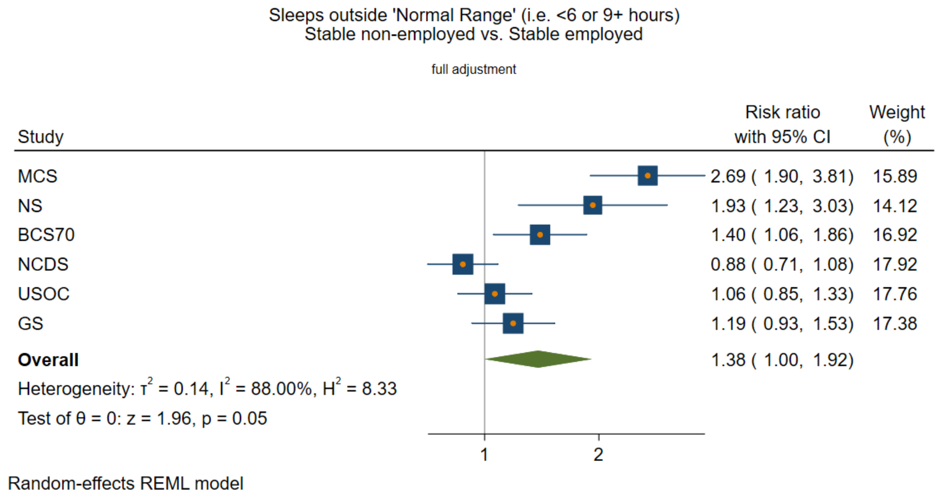
Sleeps outside 'Normal Range' (i.e. <6 or 9+ hours)
Stable non-employed vs. Stable employed
full adjustment
Study
Risk ratio with 95% CI
Weight (%)
MCS 2.69 (1.90, 3.81) 15.89
NS 1.93 (1.23, 3.03) 14.12
BCS70 1.40 (1.06, 1.86) 16.92
NCDS 0.88 (0.71, 1.08) 17.92
USOC 1.06 (0.85, 1.33) 17.76
GS 1.19 (0.93, 1.53) 17.38
Overall 1.38 (1.00, 1.92)
Heterogeneity: τ^2 = 0.14, I^2 = 88.00%, H^2 = 8.33
Test of θ = 0: z = 1.96, p = 0.05
1
2
Random-effects REML model

Figure set 8: Sleeps less than before

Commented [MJ3]:

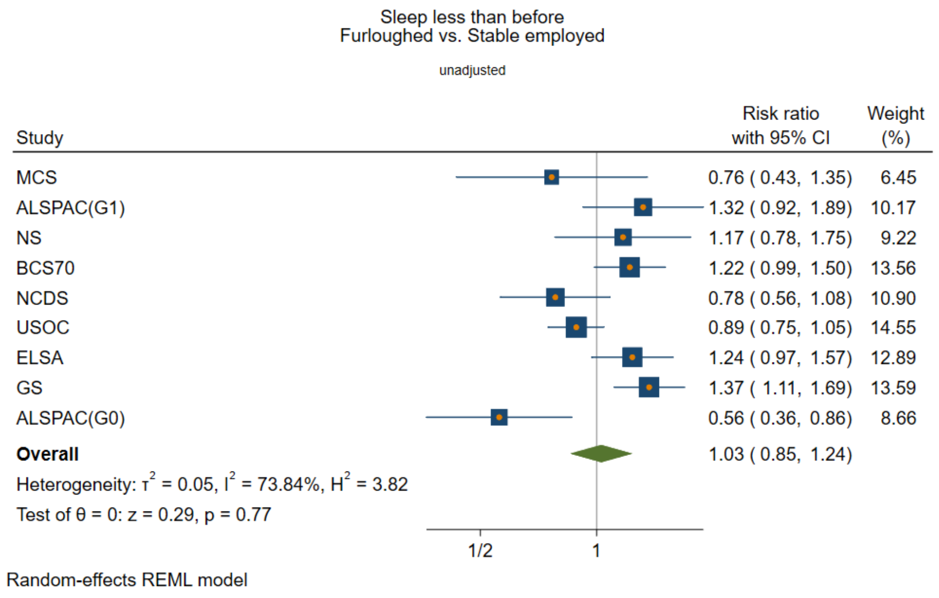

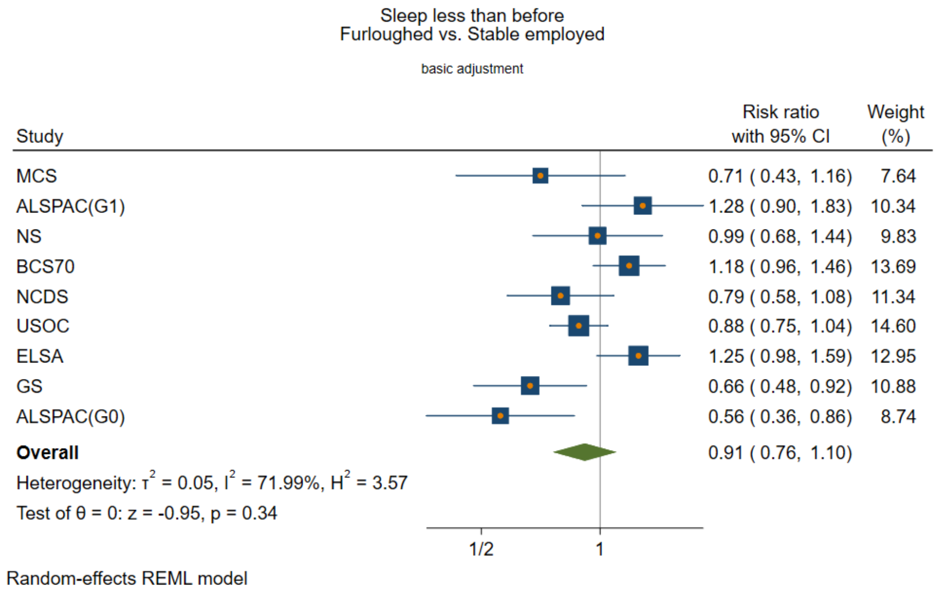

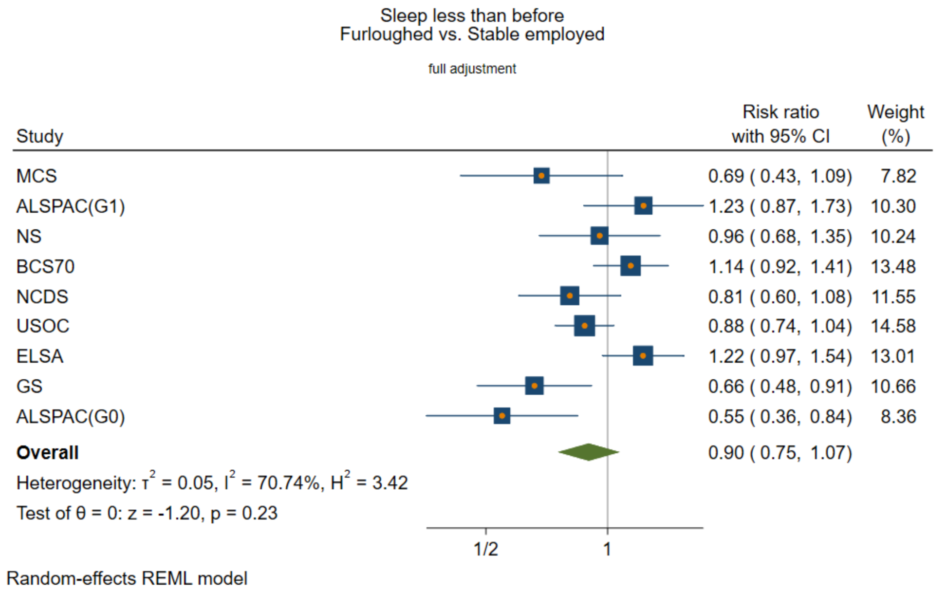
Sleep less than before
Furloughed vs. Stable employed
full adjustment
Study
Risk ratio with 95% CI
Weight (%)
MCS 0.69 (0.43, 1.09) 7.82
ALSPAC(G1) 1.23 (0.87, 1.73) 10.30
NS 0.96 (0.68, 1.35) 10.24
BCS70 1.14 (0.92, 1.41) 13.48
NCDS 0.81 (0.60, 1.08) 11.55
USOC 0.88 (0.74, 1.04) 14.58
ELSA 1.22 (0.97, 1.54) 13.01
GS 0.66 (0.48, 0.91) 10.66
ALSPAC(G0) 0.55 (0.36, 0.84) 8.36
Overall 0.90 (0.75, 1.07)
Heterogeneity: τ^2 = 0.05, I^2 = 70.74%, H^2 = 3.42
Test of θ = 0: z = -1.20, p = 0.23
1/2
1
Random-effects REML model

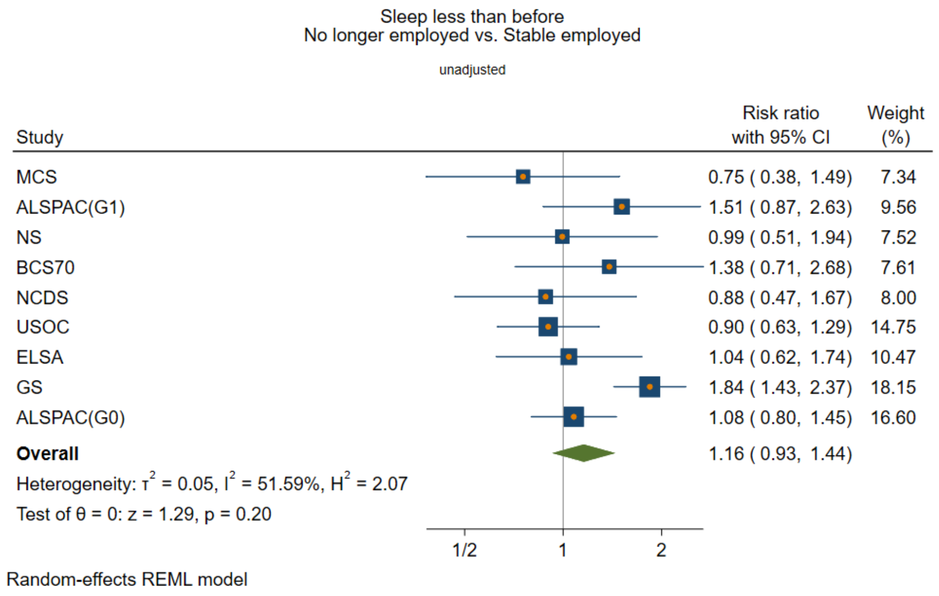
Sleep less than before
No longer employed vs. Stable employed
unadjusted
Study
Risk ratio with 95% CI
Weight (%)
MCS 0.75 (0.38, 1.49) 7.34
ALSPAC(G1) 1.51 (0.87, 2.63) 9.56
NS 0.99 (0.51, 1.94) 7.52
BCS70 1.38 (0.71, 2.68) 7.61
NCDS 0.88 (0.47, 1.67) 8.00
USOC 0.90 (0.63, 1.29) 14.75
ELSA 1.04 (0.62, 1.74) 10.47
GS 1.84 (1.43, 2.37) 18.15
ALSPAC(G0) 1.08 (0.80, 1.45) 16.60
Overall 1.16 (0.93, 1.44)
Heterogeneity: τ^2 = 0.05, I^2 = 51.59%, H^2 = 2.07
Test of θ = 0: z = 1.29, p = 0.20
1/2
1
2
Random-effects REML model

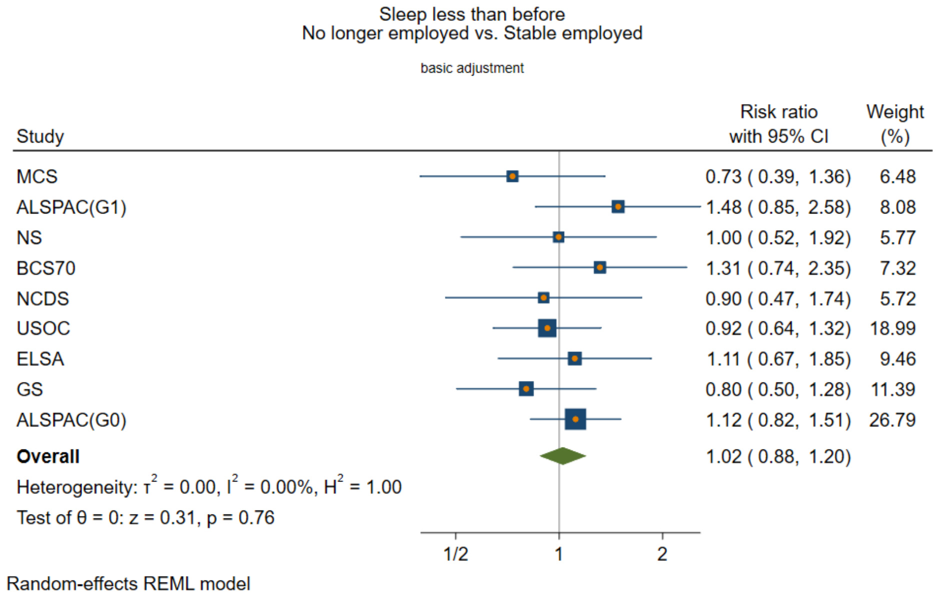
Sleep less than before
No longer employed vs. Stable employed
basic adjustment
Study
Risk ratio with 95% CI
Weight (%)
MCS 0.73 (0.39, 1.36) 6.48
ALSPAC(G1) 1.48 (0.85, 2.58) 8.08
NS 1.00 (0.52, 1.92) 5.77
BCS70 1.31 (0.74, 2.35) 7.32
NCDS 0.90 (0.47, 1.74) 5.72
USOC 0.92 (0.64, 1.32) 18.99
ELSA 1.11 (0.67, 1.85) 9.46
GS 0.80 (0.50, 1.28) 11.39
ALSPAC(G0) 1.12 (0.82, 1.51) 26.79
Overall 1.02 (0.88, 1.20)
Heterogeneity: τ^2 = 0.00, I^2 = 0.00%, H^2 = 1.00
Test of θ = 0: z = 0.31, p = 0.76
1/2
1
2
Random-effects REML model

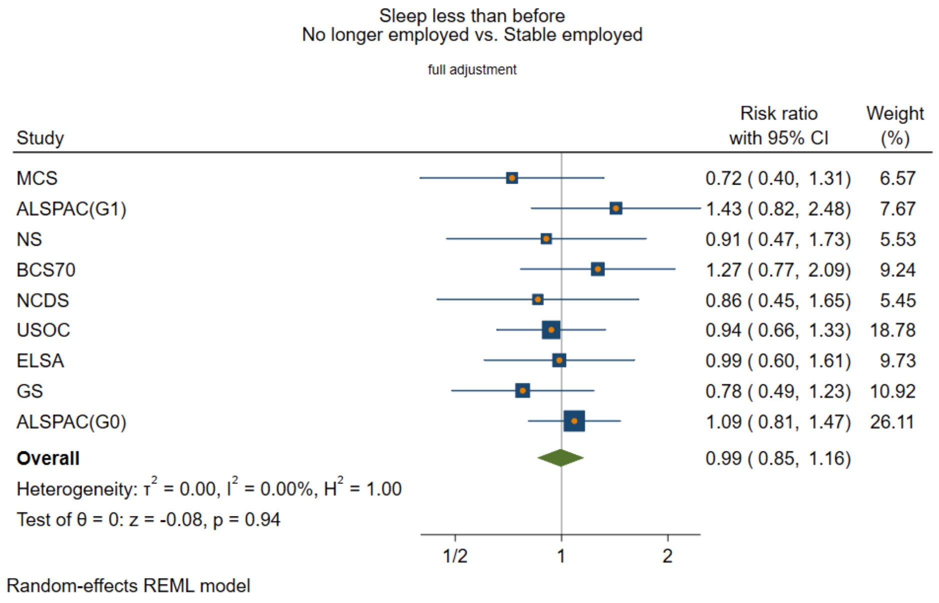
Sleep less than before
No longer employed vs. Stable employed
full adjustment
Study
Risk ratio with 95% CI
Weight (%)
MCS 0.72 (0.40, 1.31) 6.57
ALSPAC(G1) 1.43 (0.82, 2.48) 7.67
NS 0.91 (0.47, 1.73) 5.53
BCS70 1.27 (0.77, 2.09) 9.24
NCDS 0.86 (0.45, 1.65) 5.45
USOC 0.94 (0.66, 1.33) 18.78
ELSA 0.99 (0.60, 1.61) 9.73
GS 0.78 (0.49, 1.23) 10.92
ALSPAC(G0) 1.09 (0.81, 1.47) 26.11
Overall 0.99 (0.85, 1.16)
Heterogeneity: τ^2 = 0.00, I^2 = 0.00%, H^2 = 1.00
Test of θ = 0: z = -0.08, p = 0.94
1/2
1
2
Random-effects REML model

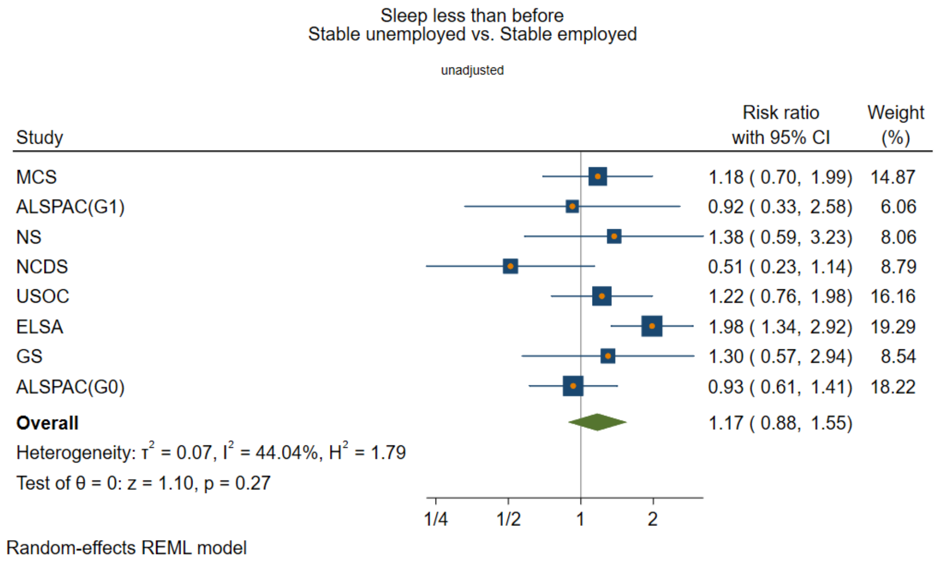
Sleep less than before
Stable unemployed vs. Stable employed
unadjusted
Study
Risk ratio with 95% CI
Weight (%)
MCS 1.18 (0.70, 1.99) 14.87
ALSPAC(G1) 0.92 (0.33, 2.58) 6.06
NS 1.38 (0.59, 3.23) 8.06
NCDS 0.51 (0.23, 1.14) 8.79
USOC 1.22 (0.76, 1.98) 16.16
ELSA 1.98 (1.34, 2.92) 19.29
GS 1.30 (0.57, 2.94) 8.54
ALSPAC(G0) 0.93 (0.61, 1.41) 18.22
Overall 1.17 (0.88, 1.55)
Heterogeneity: τ^2 = 0.07, I^2 = 44.04%, H^2 = 1.79
Test of θ = 0: z = 1.10, p = 0.27
1/4
1/2
1
2
Random-effects REML model

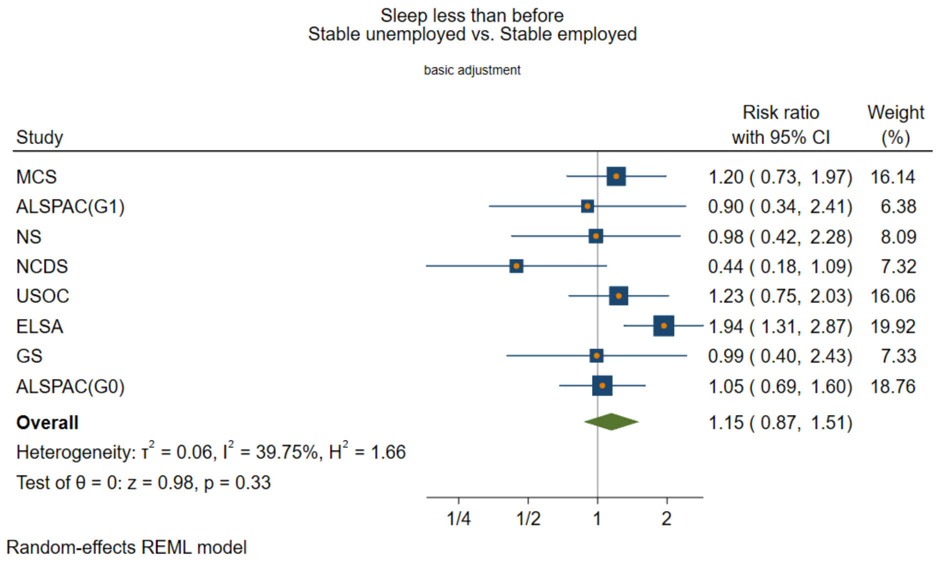
Sleep less than before
Stable unemployed vs. Stable employed
basic adjustment
Study
Risk ratio with 95% CI
Weight (%)
MCS 1.20 (0.73, 1.97) 16.14
ALSPAC(G1) 0.90 (0.34, 2.41) 6.38
NS 0.98 (0.42, 2.28) 8.09
NCDS 0.44 (0.18, 1.09) 7.32
USOC 1.23 (0.75, 2.03) 16.06
ELSA 1.94 (1.31, 2.87) 19.92
GS 0.99 (0.40, 2.43) 7.33
ALSPAC(G0) 1.05 (0.69, 1.60) 18.76
Overall 1.15 (0.87, 1.51)
Heterogeneity: τ^2 = 0.06, I^2 = 39.75%, H^2 = 1.66
Test of θ = 0: z = 0.98, p = 0.33
1/4
1/2
1
2
Random-effects REML model

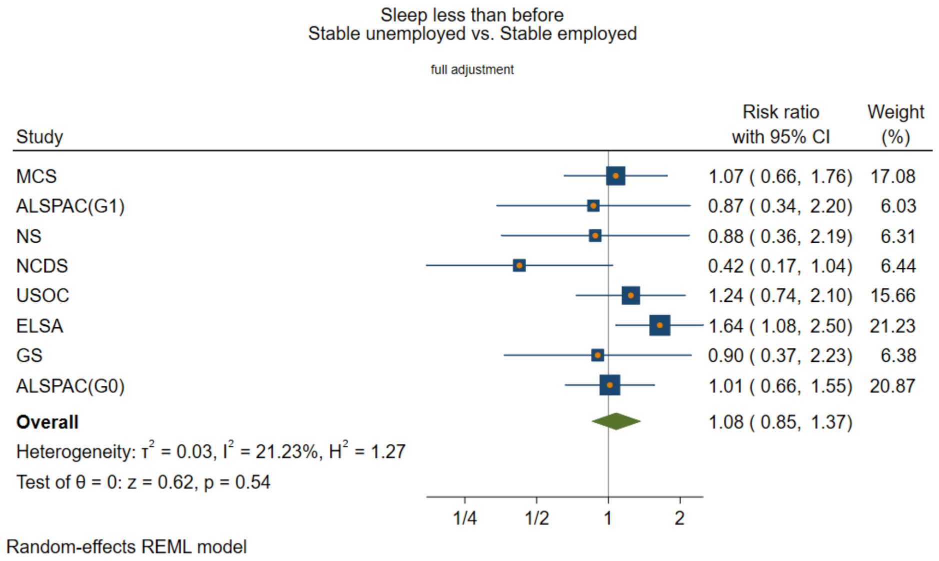
Sleep less than before
Stable unemployed vs. Stable employed
full adjustment
Study
Risk ratio with 95% CI
Weight (%)
MCS 1.07 (0.66, 1.76) 17.08
ALSPAC(G1) 0.87 (0.34, 2.20) 6.03
NS 0.88 (0.36, 2.19) 6.31
NCDS 0.42 (0.17, 1.04) 6.44
USOC 1.24 (0.74, 2.10) 15.66
ELSA 1.64 (1.08, 2.50) 21.23
GS 0.90 (0.37, 2.23) 6.38
ALSPAC(G0) 1.01 (0.66, 1.55) 20.87
Overall 1.08 (0.85, 1.37)
Heterogeneity: τ² = 0.03, I² = 21.23%, H² = 1.27
Test of θ = 0: z = 0.62, p = 0.54
1/4
1/2
1
2
Random-effects REML model

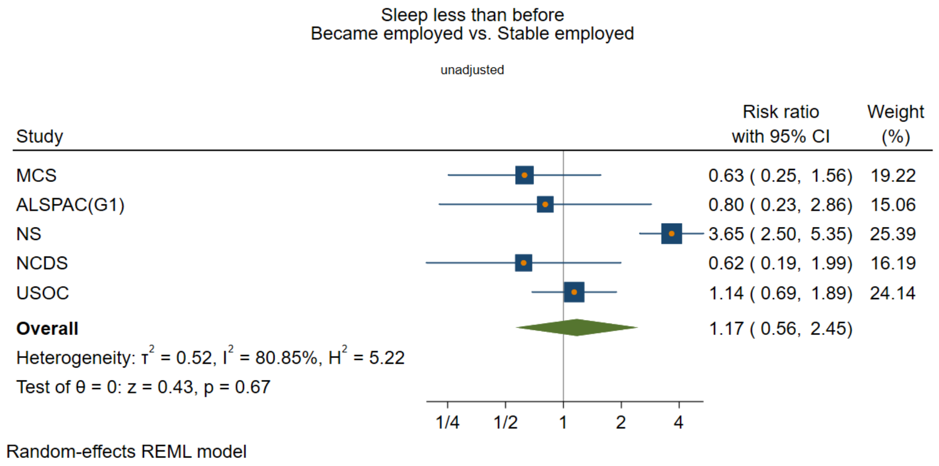
Sleep less than before
Became employed vs. Stable employed
unadjusted
Study
Risk ratio with 95% CI
Weight (%)
MCS 0.63 (0.25, 1.56) 19.22
ALSPAC(G1) 0.80 (0.23, 2.86) 15.06
NS 3.65 (2.50, 5.35) 25.39
NCDS 0.62 (0.19, 1.99) 16.19
USOC 1.14 (0.69, 1.89) 24.14
Overall 1.17 (0.56, 2.45)
Heterogeneity: τ² = 0.52, I² = 80.85%, H² = 5.22
Test of θ = 0: z = 0.43, p = 0.67
1/4
1/2
1
2
4
Random-effects REML model

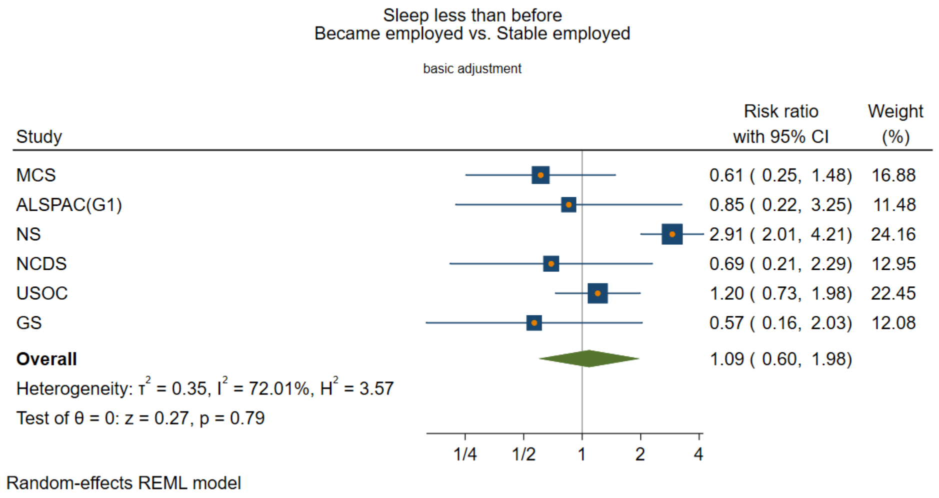
Sleep less than before
Became employed vs. Stable employed
basic adjustment
Study
Risk ratio with 95% CI
Weight (%)
MCS 0.61 (0.25, 1.48) 16.88
ALSPAC(G1) 0.85 (0.22, 3.25) 11.48
NS 2.91 (2.01, 4.21) 24.16
NCDS 0.69 (0.21, 2.29) 12.95
USOC 1.20 (0.73, 1.98) 22.45
GS 0.57 (0.16, 2.03) 12.08
Overall 1.09 (0.60, 1.98)
Heterogeneity: τ² = 0.35, I² = 72.01%, H² = 3.57
Test of θ = 0: z = 0.27, p = 0.79
1/4
1/2
1
2
4
Random-effects REML model

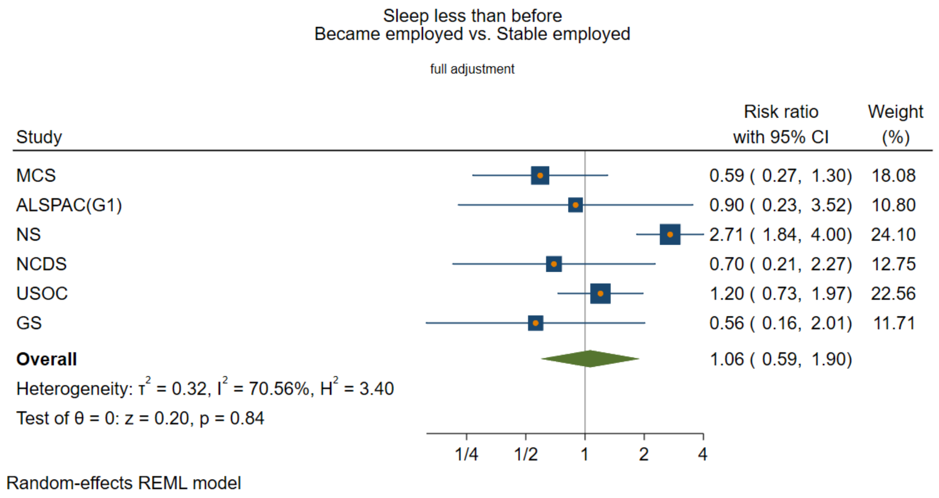

Sleep less than before
Became employed vs. Stable employed
full adjustment
Study
Risk ratio with 95% CI
Weight (%)
MCS 0.59 (0.27, 1.30) 18.08
ALSPAC(G1) 0.90 (0.23, 3.52) 10.80
NS 2.71 (1.84, 4.00) 24.10
NCDS 0.70 (0.21, 2.27) 12.75
USOC 1.20 (0.73, 1.97) 22.56
GS 0.56 (0.16, 2.01) 11.71
Overall 1.06 (0.59, 1.90)
Heterogeneity: τ^2 = 0.32, I^2 = 70.56%, H^2 = 3.40
Test of θ = 0: z = 0.20, p = 0.84
1/4
1/2
1
2
4
Random-effects REML model

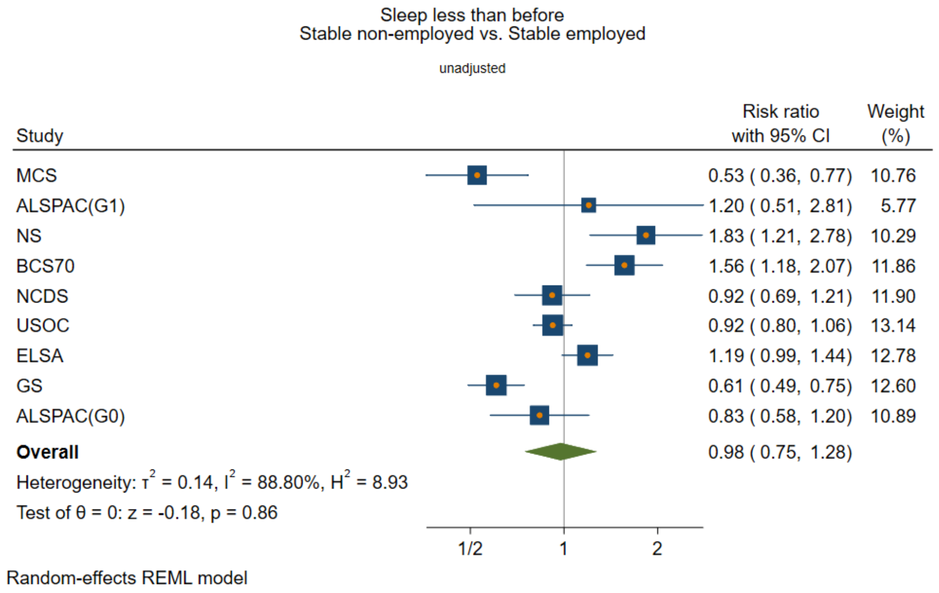

Sleep less than before
Stable non-employed vs. Stable employed
unadjusted
Study
Risk ratio with 95% CI
Weight (%)
MCS 0.53 (0.36, 0.77) 10.76
ALSPAC(G1) 1.20 (0.51, 2.81) 5.77
NS 1.83 (1.21, 2.78) 10.29
BCS70 1.56 (1.18, 2.07) 11.86
NCDS 0.92 (0.69, 1.21) 11.90
USOC 0.92 (0.80, 1.06) 13.14
ELSA 1.19 (0.99, 1.44) 12.78
GS 0.61 (0.49, 0.75) 12.60
ALSPAC(G0) 0.83 (0.58, 1.20) 10.89
Overall 0.98 (0.75, 1.28)
Heterogeneity: τ^2 = 0.14, I^2 = 88.80%, H^2 = 8.93
Test of θ = 0: z = -0.18, p = 0.86
1/2
1
2
Random-effects REML model

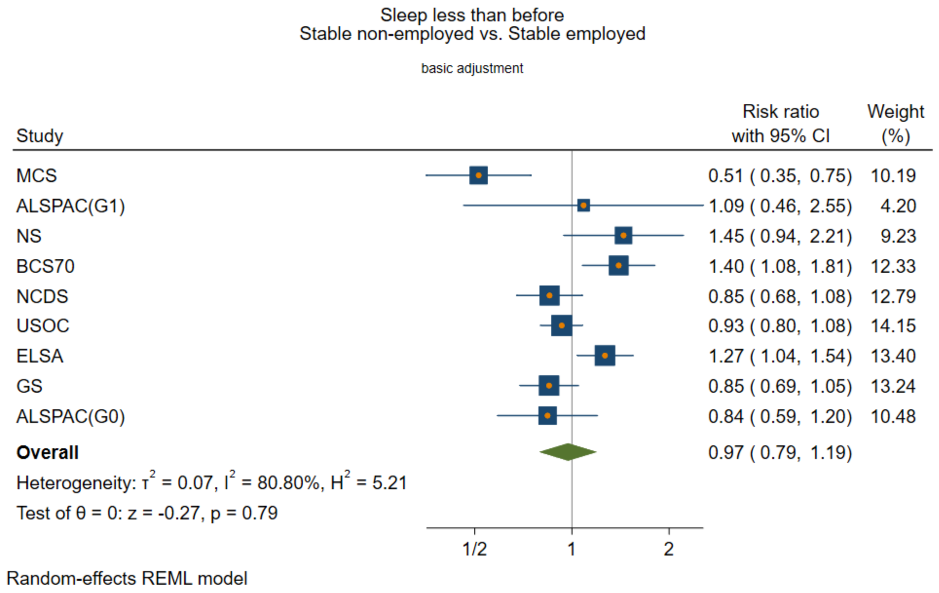

Sleep less than before
Stable non-employed vs. Stable employed
basic adjustment
Study
Risk ratio with 95% CI
Weight (%)
MCS 0.51 (0.35, 0.75) 10.19
ALSPAC(G1) 1.09 (0.46, 2.55) 4.20
NS 1.45 (0.94, 2.21) 9.23
BCS70 1.40 (1.08, 1.81) 12.33
NCDS 0.85 (0.68, 1.08) 12.79
USOC 0.93 (0.80, 1.08) 14.15
ELSA 1.27 (1.04, 1.54) 13.40
GS 0.85 (0.69, 1.05) 13.24
ALSPAC(G0) 0.84 (0.59, 1.20) 10.48
Overall 0.97 (0.79, 1.19)
Heterogeneity: τ^2 = 0.07, I^2 = 80.80%, H^2 = 5.21
Test of θ = 0: z = -0.27, p = 0.79
1/2
1
2
Random-effects REML model

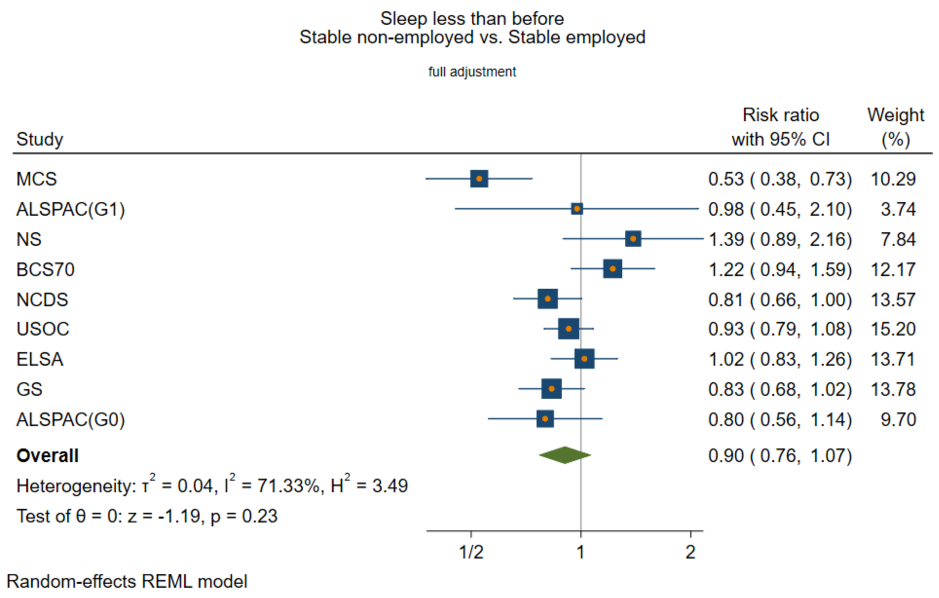
Sleep less than before
Stable non-employed vs. Stable employed
full adjustment
Study
Risk ratio with 95% CI
Weight (%)
MCS 0.53 (0.38, 0.73) 10.29
ALSPAC(G1) 0.98 (0.45, 2.10) 3.74
NS 1.39 (0.89, 2.16) 7.84
BCS70 1.22 (0.94, 1.59) 12.17
NCDS 0.81 (0.66, 1.00) 13.57
USOC 0.93 (0.79, 1.08) 15.20
ELSA 1.02 (0.83, 1.26) 13.71
GS 0.83 (0.68, 1.02) 13.78
ALSPAC(G0) 0.80 (0.56, 1.14) 9.70
Overall 0.90 (0.76, 1.07)
Heterogeneity: τ² = 0.04, I² = 71.33%, H² = 3.49
Test of θ = 0: z = -1.19, p = 0.23
1/2
1
2
Random-effects REML model

Figure set 9: Sleeps more than before

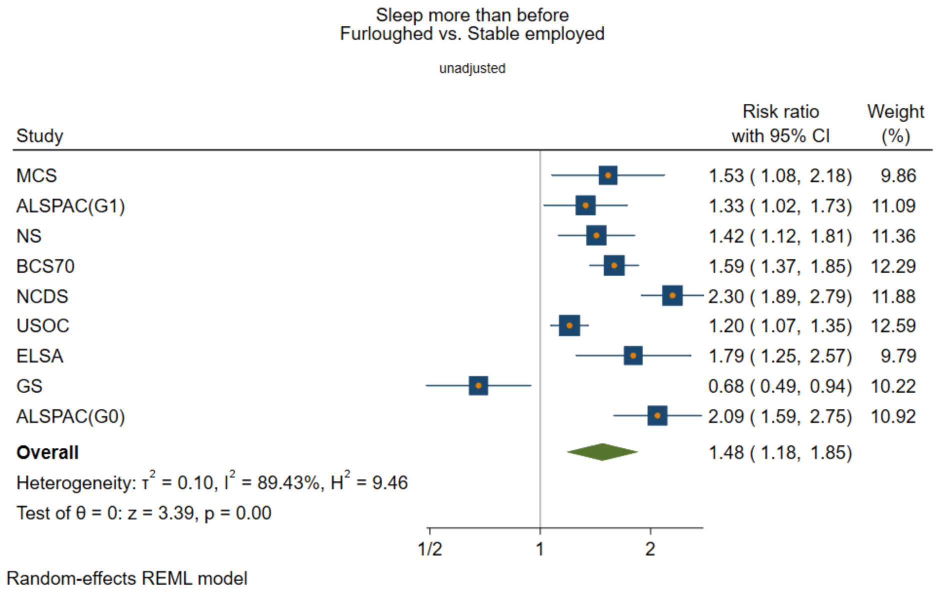

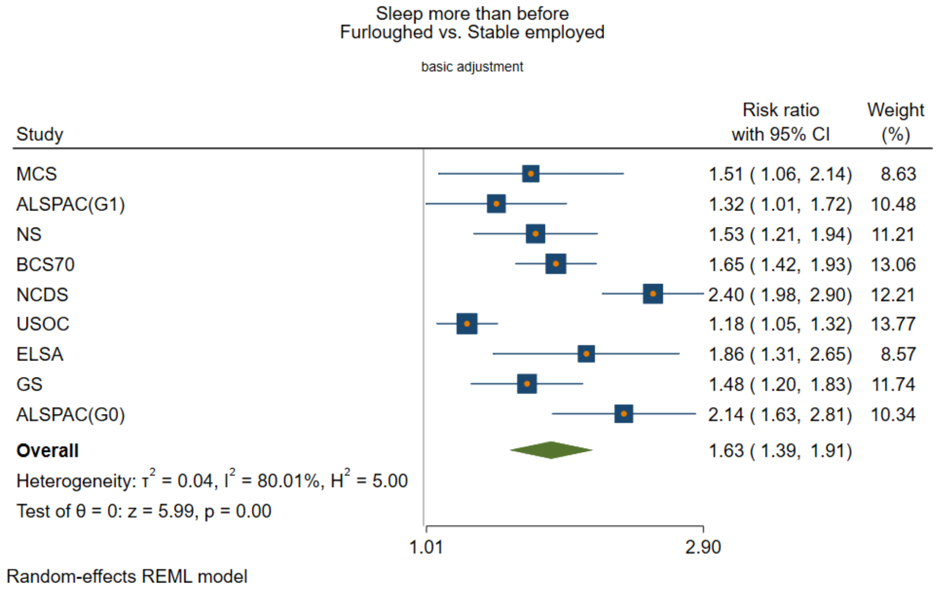

Commented [MJ4]:

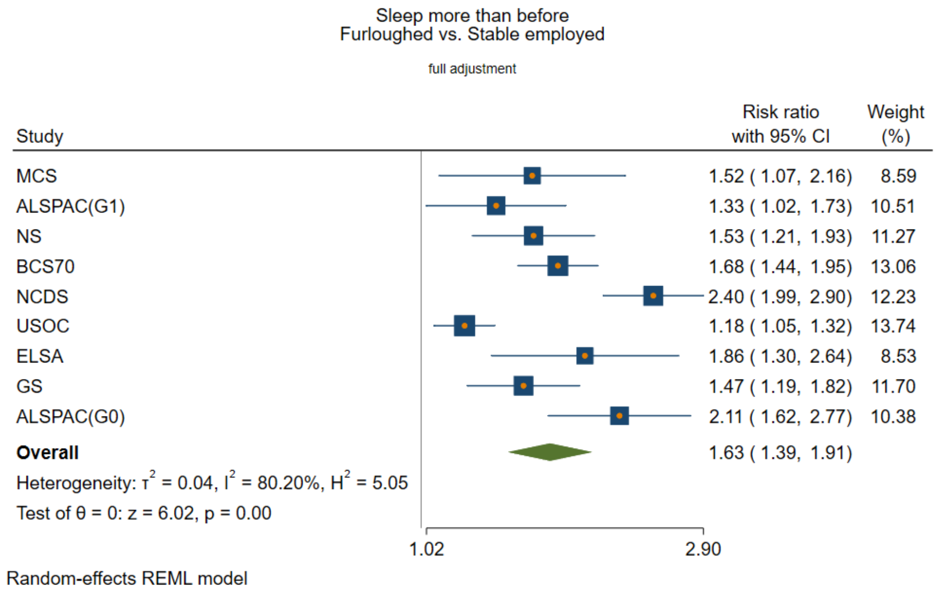
Sleep more than before
Furloughed vs. Stable employed
full adjustment
Study
Risk ratio with 95% CI
Weight (%)
MCS 1.52 (1.07, 2.16) 8.59
ALSPAC(G1) 1.33 (1.02, 1.73) 10.51
NS 1.53 (1.21, 1.93) 11.27
BCS70 1.68 (1.44, 1.95) 13.06
NCDS 2.40 (1.99, 2.90) 12.23
USOC 1.18 (1.05, 1.32) 13.74
ELSA 1.86 (1.30, 2.64) 8.53
GS 1.47 (1.19, 1.82) 11.70
ALSPAC(G0) 2.11 (1.62, 2.77) 10.38
Overall 1.63 (1.39, 1.91)
Heterogeneity: τ² = 0.04, I² = 80.20%, H² = 5.05
Test of θ = 0: z = 6.02, p = 0.00
1.02
2.90
Random-effects REML model

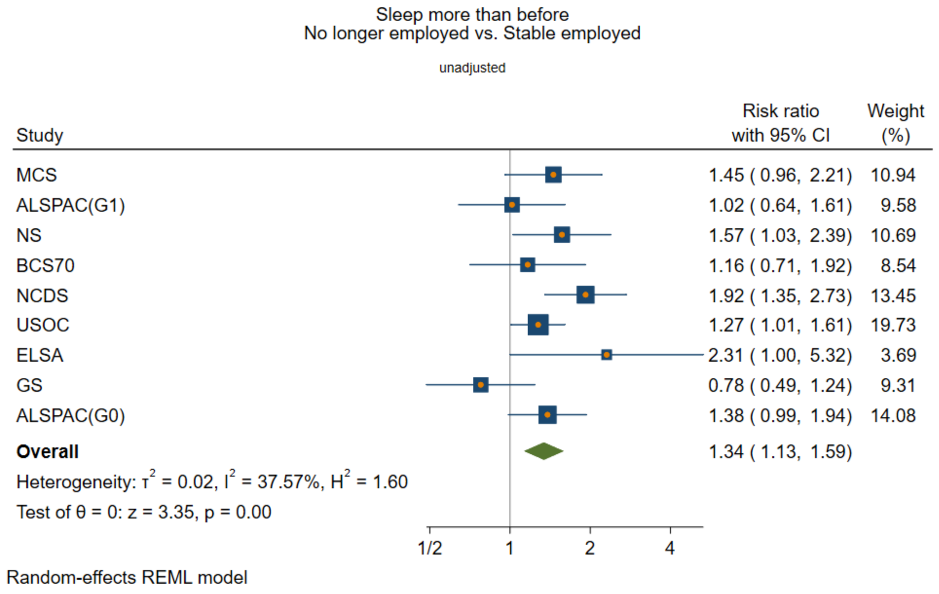
Sleep more than before
No longer employed vs. Stable employed
unadjusted
Study
Risk ratio with 95% CI
Weight (%)
MCS 1.45 (0.96, 2.21) 10.94
ALSPAC(G1) 1.02 (0.64, 1.61) 9.58
NS 1.57 (1.03, 2.39) 10.69
BCS70 1.16 (0.71, 1.92) 8.54
NCDS 1.92 (1.35, 2.73) 13.45
USOC 1.27 (1.01, 1.61) 19.73
ELSA 2.31 (1.00, 5.32) 3.69
GS 0.78 (0.49, 1.24) 9.31
ALSPAC(G0) 1.38 (0.99, 1.94) 14.08
Overall 1.34 (1.13, 1.59)
Heterogeneity: τ² = 0.02, I² = 37.57%, H² = 1.60
Test of θ = 0: z = 3.35, p = 0.00
1/2
1
2
4
Random-effects REML model

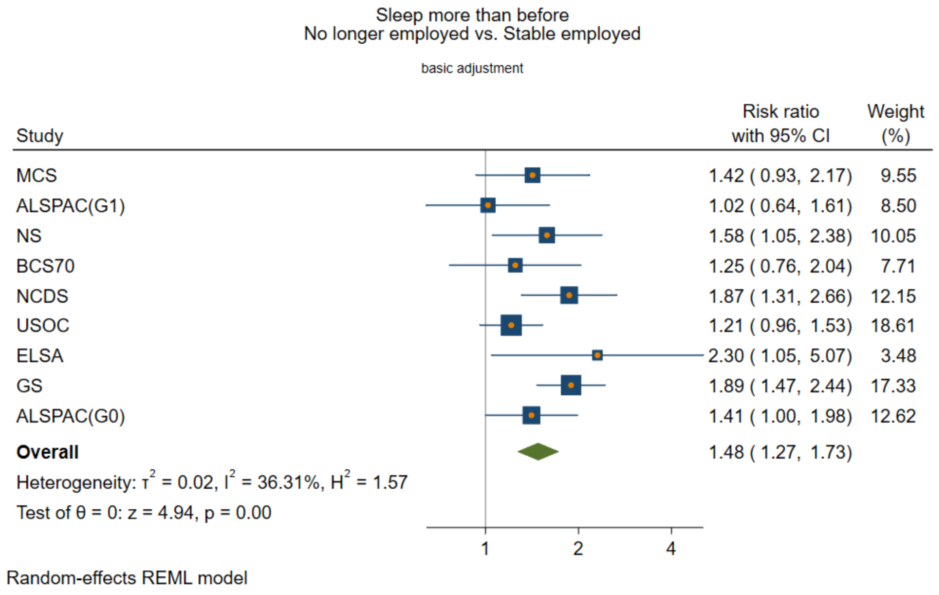
Sleep more than before
No longer employed vs. Stable employed
basic adjustment
Study
Risk ratio with 95% CI
Weight (%)
MCS 1.42 (0.93, 2.17) 9.55
ALSPAC(G1) 1.02 (0.64, 1.61) 8.50
NS 1.58 (1.05, 2.38) 10.05
BCS70 1.25 (0.76, 2.04) 7.71
NCDS 1.87 (1.31, 2.66) 12.15
USOC 1.21 (0.96, 1.53) 18.61
ELSA 2.30 (1.05, 5.07) 3.48
GS 1.89 (1.47, 2.44) 17.33
ALSPAC(G0) 1.41 (1.00, 1.98) 12.62
Overall 1.48 (1.27, 1.73)
Heterogeneity: τ² = 0.02, I² = 36.31%, H² = 1.57
Test of θ = 0: z = 4.94, p = 0.00
1
2
4
Random-effects REML model

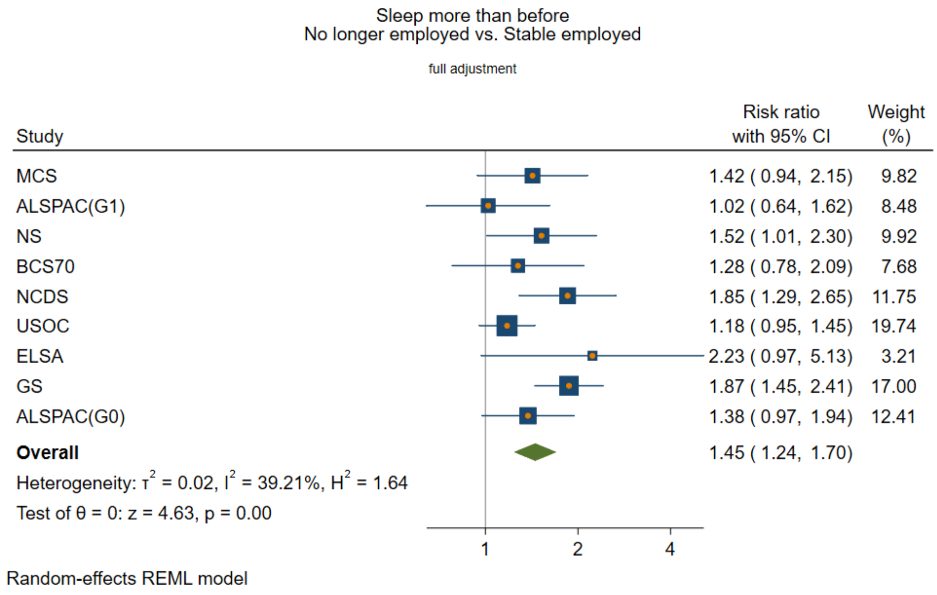
Sleep more than before
No longer employed vs. Stable employed
full adjustment
Study
Risk ratio with 95% CI
Weight (%)
MCS 1.42 (0.94, 2.15) 9.82
ALSPAC(G1) 1.02 (0.64, 1.62) 8.48
NS 1.52 (1.01, 2.30) 9.92
BCS70 1.28 (0.78, 2.09) 7.68
NCDS 1.85 (1.29, 2.65) 11.75
USOC 1.18 (0.95, 1.45) 19.74
ELSA 2.23 (0.97, 5.13) 3.21
GS 1.87 (1.45, 2.41) 17.00
ALSPAC(G0) 1.38 (0.97, 1.94) 12.41
Overall 1.45 (1.24, 1.70)
Heterogeneity: τ² = 0.02, I² = 39.21%, H² = 1.64
Test of θ = 0: z = 4.63, p = 0.00
1
2
4
Random-effects REML model

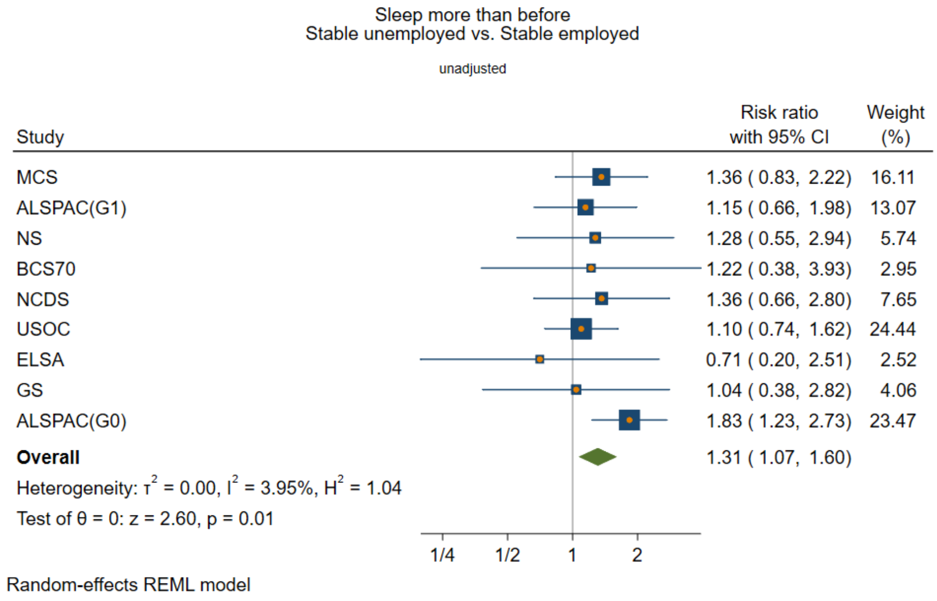
Sleep more than before
Stable unemployed vs. Stable employed
unadjusted
Study
Risk ratio with 95% CI
Weight (%)
MCS 1.36 (0.83, 2.22) 16.11
ALSPAC(G1) 1.15 (0.66, 1.98) 13.07
NS 1.28 (0.55, 2.94) 5.74
BCS70 1.22 (0.38, 3.93) 2.95
NCDS 1.36 (0.66, 2.80) 7.65
USOC 1.10 (0.74, 1.62) 24.44
ELSA 0.71 (0.20, 2.51) 2.52
GS 1.04 (0.38, 2.82) 4.06
ALSPAC(G0) 1.83 (1.23, 2.73) 23.47
Overall 1.31 (1.07, 1.60)
Heterogeneity: τ² = 0.00, I² = 3.95%, H² = 1.04
Test of θ = 0: z = 2.60, p = 0.01
1/4
1/2
1
2
Random-effects REML model

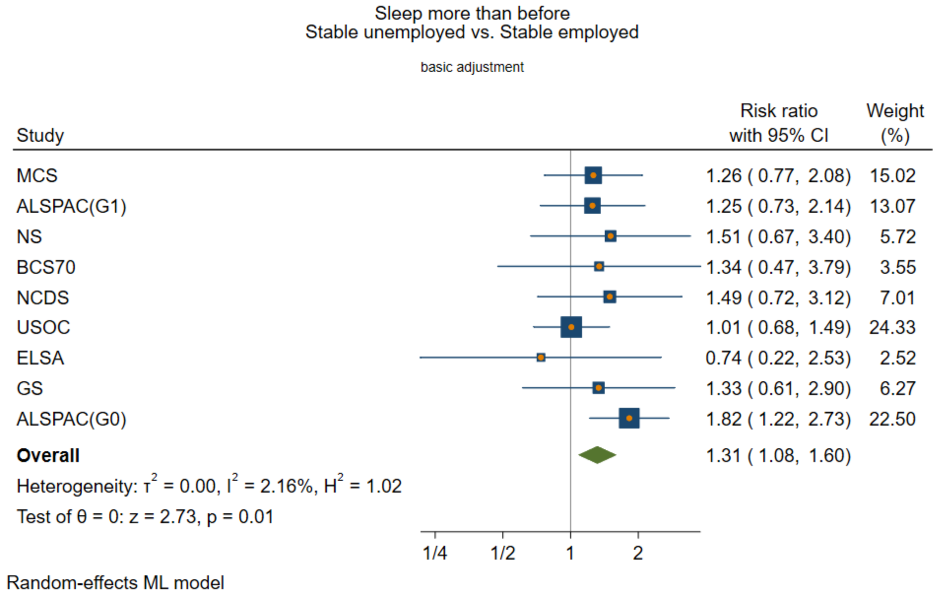
Sleep more than before
Stable unemployed vs. Stable employed
basic adjustment
Study
Risk ratio with 95% CI
Weight (%)
MCS 1.26 (0.77, 2.08) 15.02
ALSPAC(G1) 1.25 (0.73, 2.14) 13.07
NS 1.51 (0.67, 3.40) 5.72
BCS70 1.34 (0.47, 3.79) 3.55
NCDS 1.49 (0.72, 3.12) 7.01
USOC 1.01 (0.68, 1.49) 24.33
ELSA 0.74 (0.22, 2.53) 2.52
GS 1.33 (0.61, 2.90) 6.27
ALSPAC(G0) 1.82 (1.22, 2.73) 22.50
Overall 1.31 (1.08, 1.60)
Heterogeneity: τ² = 0.00, I² = 2.16%, H² = 1.02
Test of θ = 0: z = 2.73, p = 0.01
1/4
1/2
1
2
Random-effects ML model

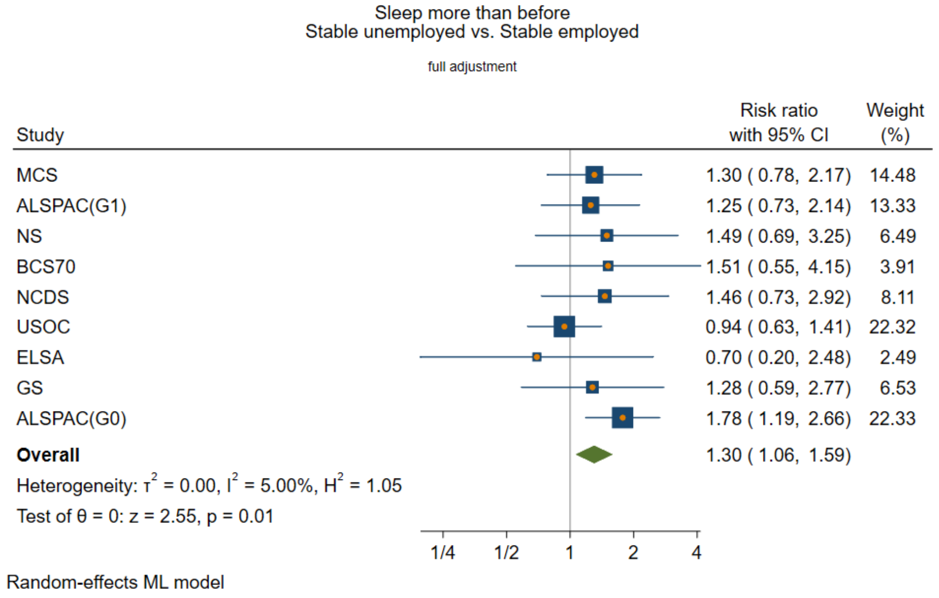
Sleep more than before
Stable unemployed vs. Stable employed
full adjustment
Study
Risk ratio with 95% CI
Weight (%)
MCS 1.30 (0.78, 2.17) 14.48
ALSPAC(G1) 1.25 (0.73, 2.14) 13.33
NS 1.49 (0.69, 3.25) 6.49
BCS70 1.51 (0.55, 4.15) 3.91
NCDS 1.46 (0.73, 2.92) 8.11
USOC 0.94 (0.63, 1.41) 22.32
ELSA 0.70 (0.20, 2.48) 2.49
GS 1.28 (0.59, 2.77) 6.53
ALSPAC(G0) 1.78 (1.19, 2.66) 22.33
Overall 1.30 (1.06, 1.59)
Heterogeneity: τ² = 0.00, I² = 5.00%, H² = 1.05
Test of θ = 0: z = 2.55, p = 0.01
1/4
1/2
1
2
4
Random-effects ML model

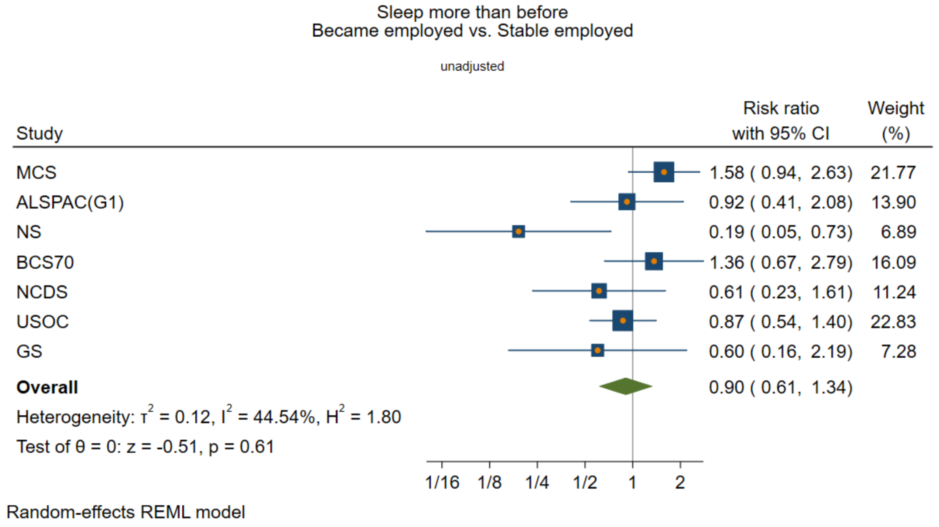
Sleep more than before
Became employed vs. Stable employed
unadjusted
Study
Risk ratio with 95% CI
Weight (%)
MCS 1.58 (0.94, 2.63) 21.77
ALSPAC(G1) 0.92 (0.41, 2.08) 13.90
NS 0.19 (0.05, 0.73) 6.89
BCS70 1.36 (0.67, 2.79) 16.09
NCDS 0.61 (0.23, 1.61) 11.24
USOC 0.87 (0.54, 1.40) 22.83
GS 0.60 (0.16, 2.19) 7.28
Overall 0.90 (0.61, 1.34)
Heterogeneity: τ² = 0.12, I² = 44.54%, H² = 1.80
Test of θ = 0: z = -0.51, p = 0.61
1/16
1/8
1/4
1/2
1
2
Random-effects REML model

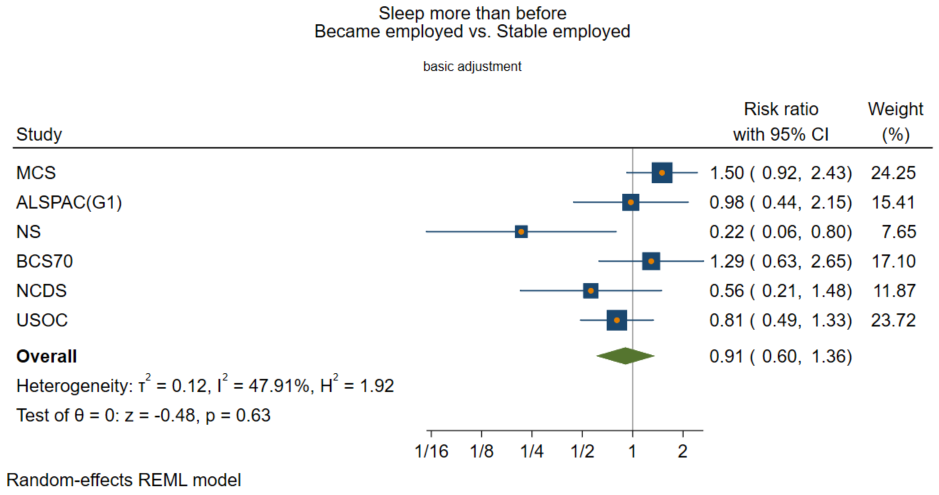
Sleep more than before
Became employed vs. Stable employed
basic adjustment
Study
Risk ratio with 95% CI
Weight (%)
MCS 1.50 (0.92, 2.43) 24.25
ALSPAC(G1) 0.98 (0.44, 2.15) 15.41
NS 0.22 (0.06, 0.80) 7.65
BCS70 1.29 (0.63, 2.65) 17.10
NCDS 0.56 (0.21, 1.48) 11.87
USOC 0.81 (0.49, 1.33) 23.72
Overall 0.91 (0.60, 1.36)
Heterogeneity: τ² = 0.12, I² = 47.91%, H² = 1.92
Test of θ = 0: z = -0.48, p = 0.63
1/16 1/8 1/4 1/2 1 2
Random-effects REML model

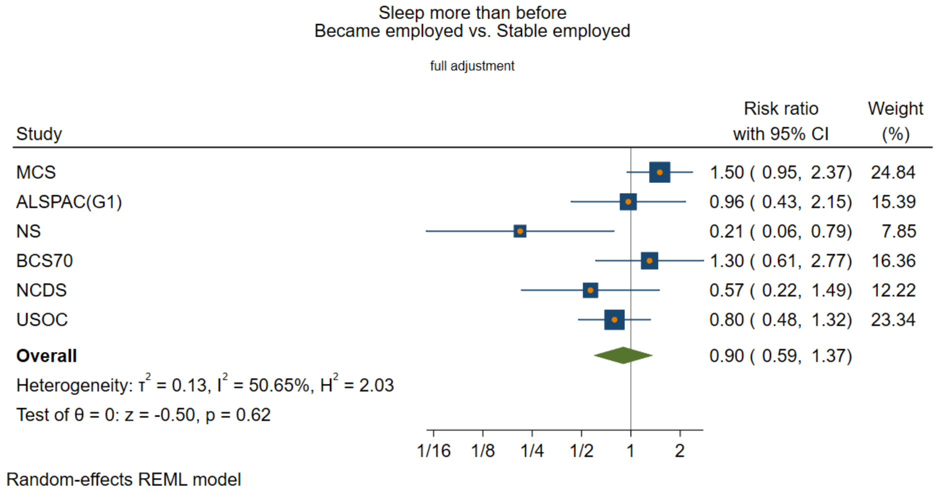
Sleep more than before
Became employed vs. Stable employed
full adjustment
Study
Risk ratio with 95% CI
Weight (%)
MCS 1.50 (0.95, 2.37) 24.84
ALSPAC(G1) 0.96 (0.43, 2.15) 15.39
NS 0.21 (0.06, 0.79) 7.85
BCS70 1.30 (0.61, 2.77) 16.36
NCDS 0.57 (0.22, 1.49) 12.22
USOC 0.80 (0.48, 1.32) 23.34
Overall 0.90 (0.59, 1.37)
Heterogeneity: τ² = 0.13, I² = 50.65%, H² = 2.03
Test of θ = 0: z = -0.50, p = 0.62
1/16 1/8 1/4 1/2 1 2
Random-effects REML model

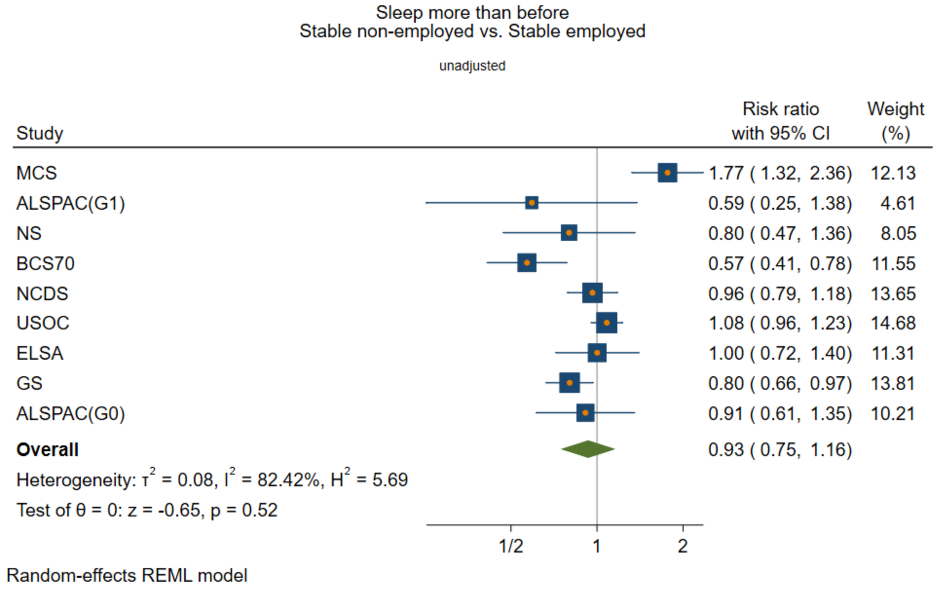
Sleep more than before
Stable non-employed vs. Stable employed
unadjusted
Study
Risk ratio with 95% CI
Weight (%)
MCS 1.77 (1.32, 2.36) 12.13
ALSPAC(G1) 0.59 (0.25, 1.38) 4.61
NS 0.80 (0.47, 1.36) 8.05
BCS70 0.57 (0.41, 0.78) 11.55
NCDS 0.96 (0.79, 1.18) 13.65
USOC 1.08 (0.96, 1.23) 14.68
ELSA 1.00 (0.72, 1.40) 11.31
GS 0.80 (0.66, 0.97) 13.81
ALSPAC(G0) 0.91 (0.61, 1.35) 10.21
Overall 0.93 (0.75, 1.16)
Heterogeneity: τ² = 0.08, I² = 82.42%, H² = 5.69
Test of θ = 0: z = -0.65, p = 0.52
1/2 1 2
Random-effects REML model

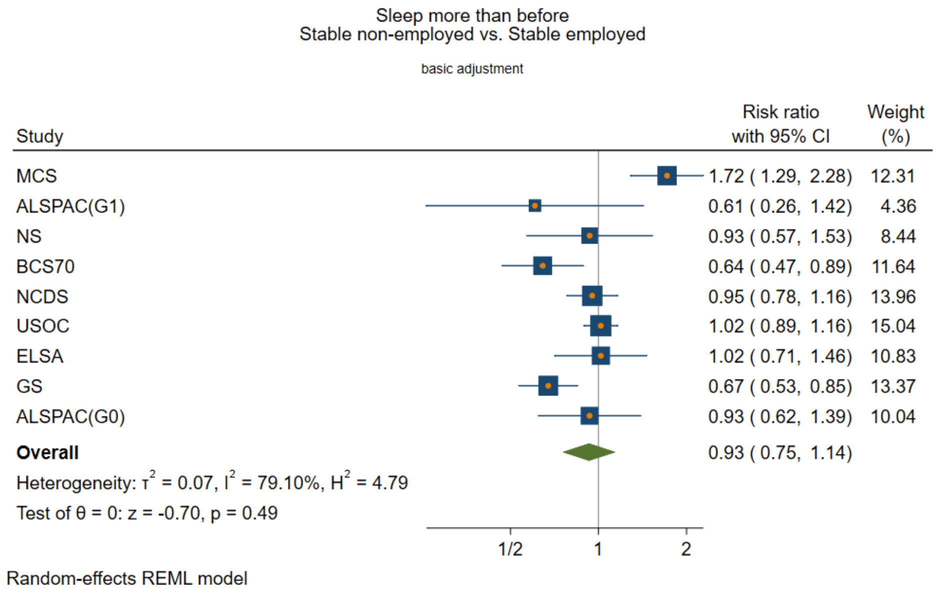
Sleep more than before
Stable non-employed vs. Stable employed
basic adjustment
Study
Risk ratio with 95% CI
Weight (%)
MCS 1.72 (1.29, 2.28) 12.31
ALSPAC(G1) 0.61 (0.26, 1.42) 4.36
NS 0.93 (0.57, 1.53) 8.44
BCS70 0.64 (0.47, 0.89) 11.64
NCDS 0.95 (0.78, 1.16) 13.96
USOC 1.02 (0.89, 1.16) 15.04
ELSA 1.02 (0.71, 1.46) 10.83
GS 0.67 (0.53, 0.85) 13.37
ALSPAC(G0) 0.93 (0.62, 1.39) 10.04
Overall 0.93 (0.75, 1.14)
Heterogeneity: τ^2 = 0.07, I^2 = 79.10%, H^2 = 4.79
Test of θ = 0: z = -0.70, p = 0.49
1/2
1
2
Random-effects REML model

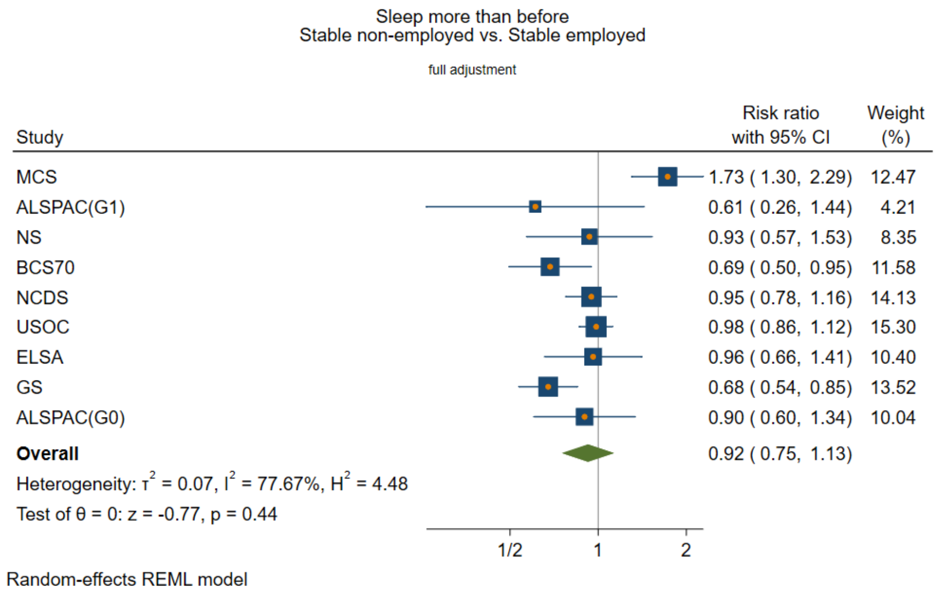
Sleep more than before
Stable non-employed vs. Stable employed
full adjustment
Study
Risk ratio with 95% CI
Weight (%)
MCS 1.73 (1.30, 2.29) 12.47
ALSPAC(G1) 0.61 (0.26, 1.44) 4.21
NS 0.93 (0.57, 1.53) 8.35
BCS70 0.69 (0.50, 0.95) 11.58
NCDS 0.95 (0.78, 1.16) 14.13
USOC 0.98 (0.86, 1.12) 15.30
ELSA 0.96 (0.66, 1.41) 10.40
GS 0.68 (0.54, 0.85) 13.52
ALSPAC(G0) 0.90 (0.60, 1.34) 10.04
Overall 0.92 (0.75, 1.13)
Heterogeneity: τ^2 = 0.07, I^2 = 77.67%, H^2 = 4.48
Test of θ = 0: z = -0.77, p = 0.44
1/2
1
2
Random-effects REML model

Figure set 10: From 6/9 hours a night to outside ‘normal range’

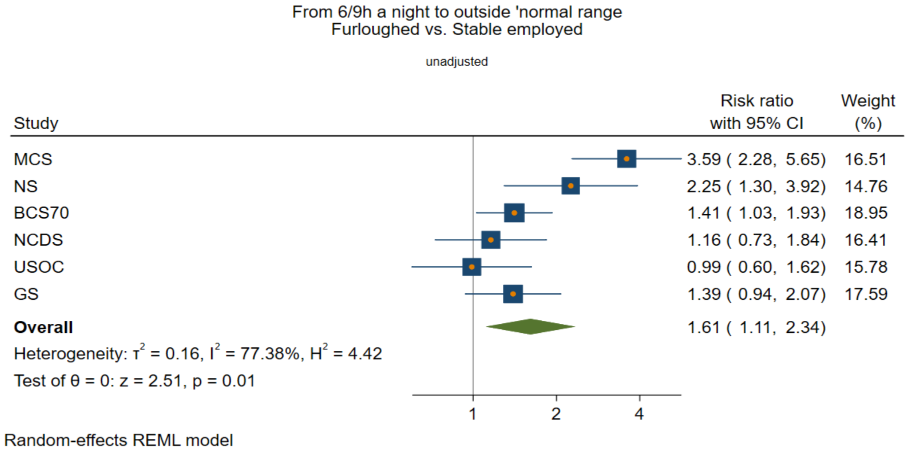

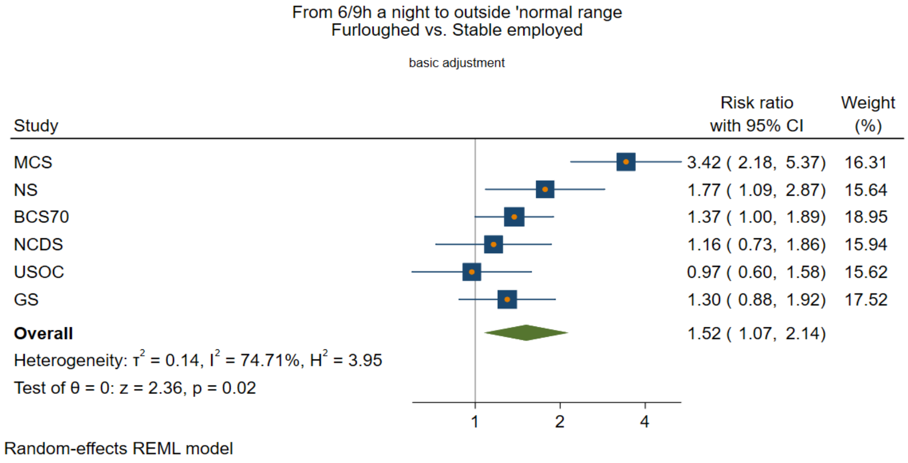

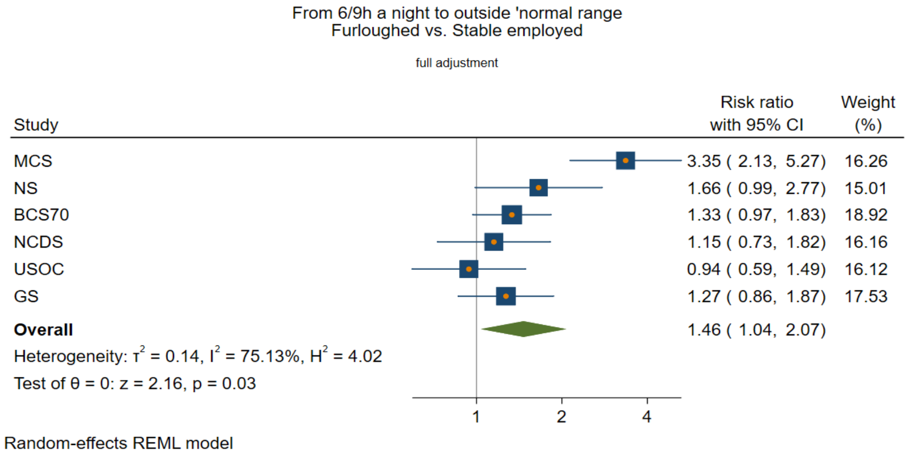

From 6/9h a night to outside 'normal range
No longer employed vs. Stable employed

unadjusted

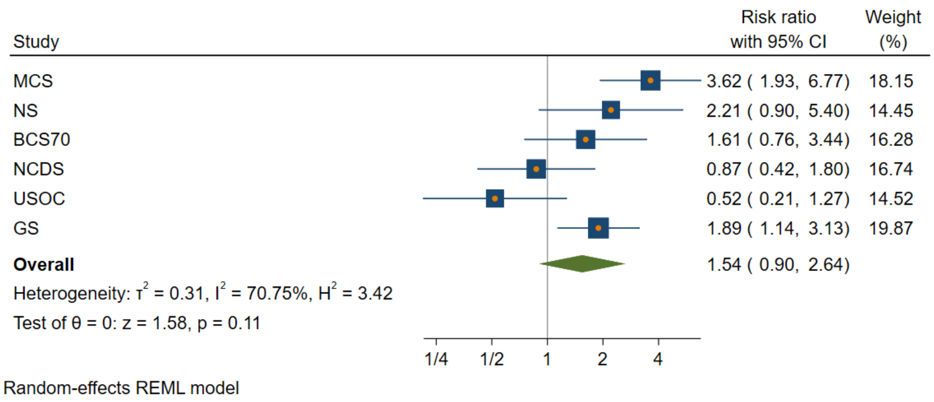

From 6/9h a night to outside 'normal range
No longer employed vs. Stable employed

basic adjustment

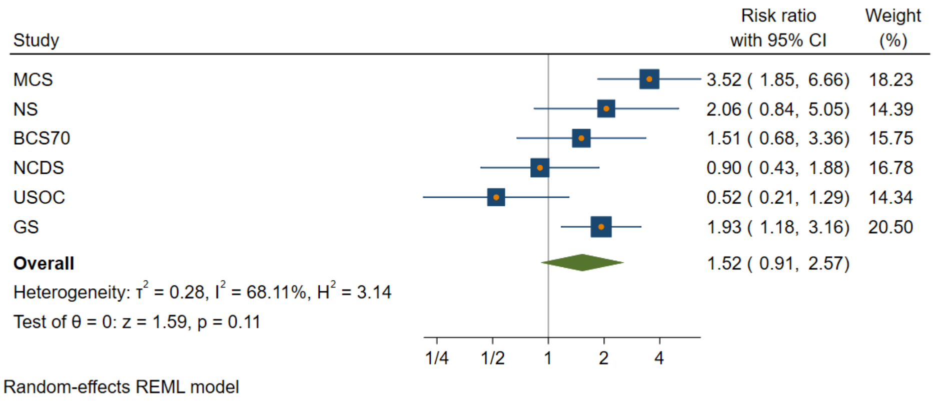

From 6/9h a night to outside 'normal range
No longer employed vs. Stable employed

full adjustment

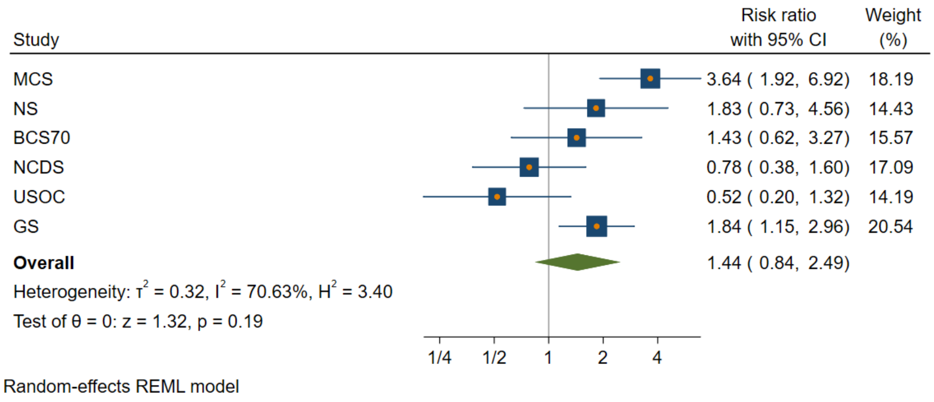

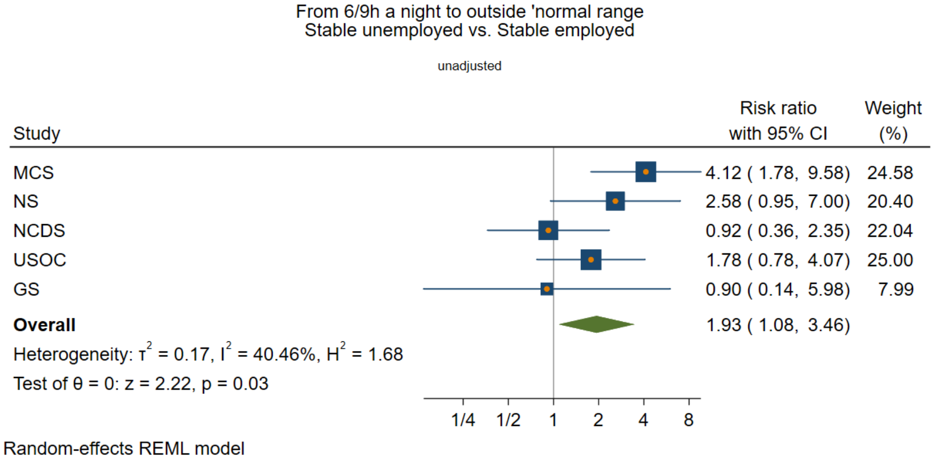
From 6/9h a night to outside 'normal range
Stable unemployed vs. Stable employed
unadjusted
Study
Risk ratio with 95% CI
Weight (%)
MCS 4.12 (1.78, 9.58) 24.58
NS 2.58 (0.95, 7.00) 20.40
NCDS 0.92 (0.36, 2.35) 22.04
USOC 1.78 (0.78, 4.07) 25.00
GS 0.90 (0.14, 5.98) 7.99
Overall 1.93 (1.08, 3.46)
Heterogeneity: τ² = 0.17, I² = 40.46%, H² = 1.68
Test of θ = 0: z = 2.22, p = 0.03
1/4
1/2
1
2
4
8
Random-effects REML model

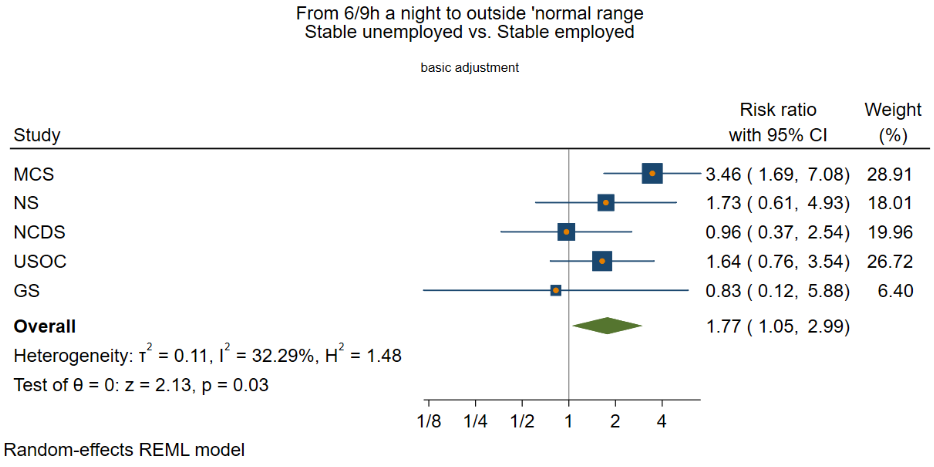
From 6/9h a night to outside 'normal range
Stable unemployed vs. Stable employed
basic adjustment
Study
Risk ratio with 95% CI
Weight (%)
MCS 3.46 (1.69, 7.08) 28.91
NS 1.73 (0.61, 4.93) 18.01
NCDS 0.96 (0.37, 2.54) 19.96
USOC 1.64 (0.76, 3.54) 26.72
GS 0.83 (0.12, 5.88) 6.40
Overall 1.77 (1.05, 2.99)
Heterogeneity: τ² = 0.11, I² = 32.29%, H² = 1.48
Test of θ = 0: z = 2.13, p = 0.03
1/8
1/4
1/2
1
2
4
Random-effects REML model

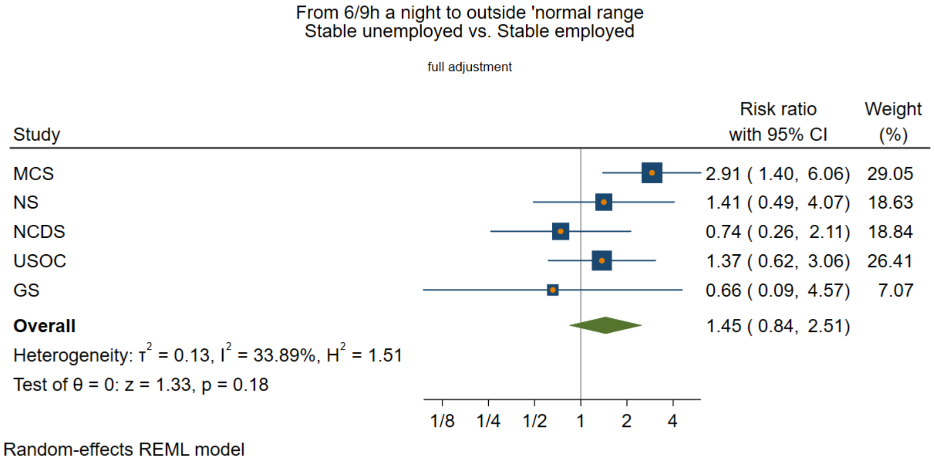
From 6/9h a night to outside 'normal range
Stable unemployed vs. Stable employed
full adjustment
Study
Risk ratio with 95% CI
Weight (%)
MCS 2.91 (1.40, 6.06) 29.05
NS 1.41 (0.49, 4.07) 18.63
NCDS 0.74 (0.26, 2.11) 18.84
USOC 1.37 (0.62, 3.06) 26.41
GS 0.66 (0.09, 4.57) 7.07
Overall 1.45 (0.84, 2.51)
Heterogeneity: τ² = 0.13, I² = 33.89%, H² = 1.51
Test of θ = 0: z = 1.33, p = 0.18
1/8
1/4
1/2
1
2
4
Random-effects REML model

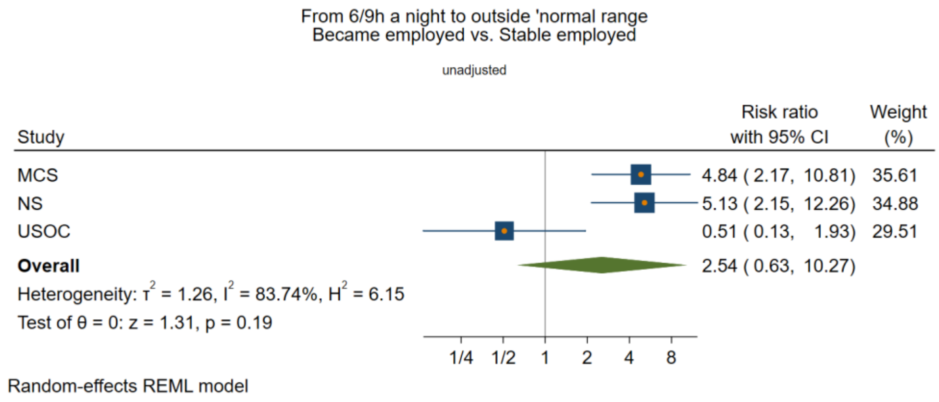
From 6/9h a night to outside 'normal range
Became employed vs. Stable employed
unadjusted
Study
Risk ratio with 95% CI
Weight (%)
MCS 4.84 (2.17, 10.81) 35.61
NS 5.13 (2.15, 12.26) 34.88
USOC 0.51 (0.13, 1.93) 29.51
Overall 2.54 (0.63, 10.27)
Heterogeneity: τ² = 1.26, I² = 83.74%, H² = 6.15
Test of θ = 0: z = 1.31, p = 0.19
1/4 1/2 1 2 4 8
Random-effects REML model

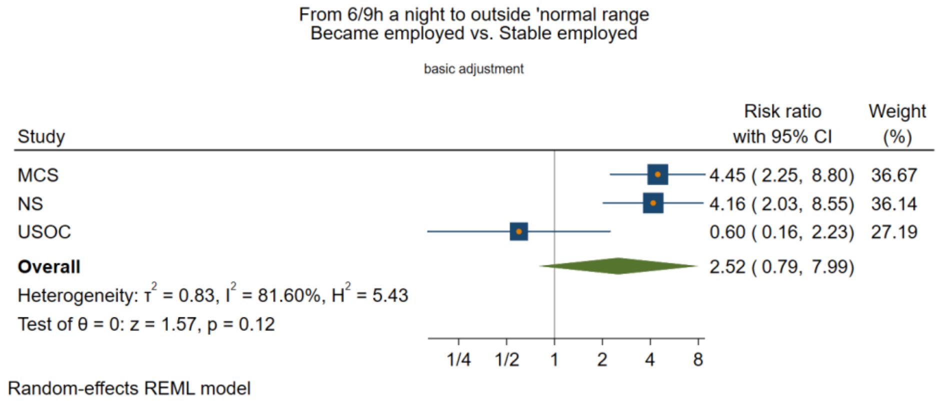
From 6/9h a night to outside 'normal range
Became employed vs. Stable employed
basic adjustment
Study
Risk ratio with 95% CI
Weight (%)
MCS 4.45 (2.25, 8.80) 36.67
NS 4.16 (2.03, 8.55) 36.14
USOC 0.60 (0.16, 2.23) 27.19
Overall 2.52 (0.79, 7.99)
Heterogeneity: τ² = 0.83, I² = 81.60%, H² = 5.43
Test of θ = 0: z = 1.57, p = 0.12
1/4 1/2 1 2 4 8
Random-effects REML model

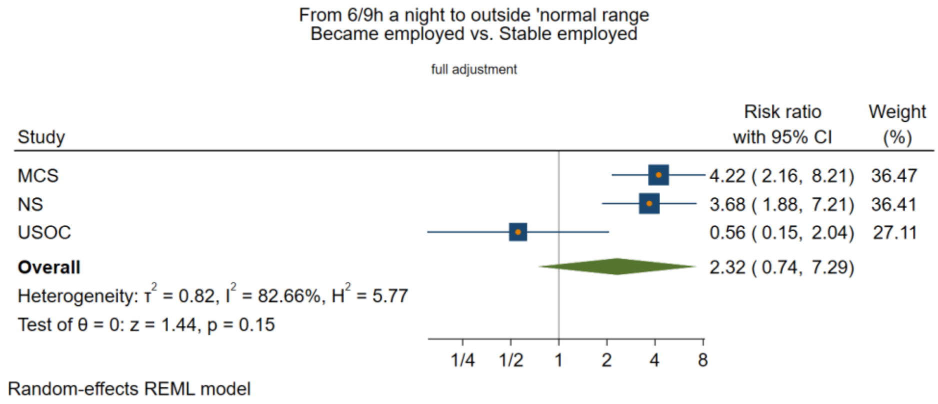
From 6/9h a night to outside 'normal range
Became employed vs. Stable employed
full adjustment
Study
Risk ratio with 95% CI
Weight (%)
MCS 4.22 (2.16, 8.21) 36.47
NS 3.68 (1.88, 7.21) 36.41
USOC 0.56 (0.15, 2.04) 27.11
Overall 2.32 (0.74, 7.29)
Heterogeneity: τ² = 0.82, I² = 82.66%, H² = 5.77
Test of θ = 0: z = 1.44, p = 0.15
1/4 1/2 1 2 4 8
Random-effects REML model

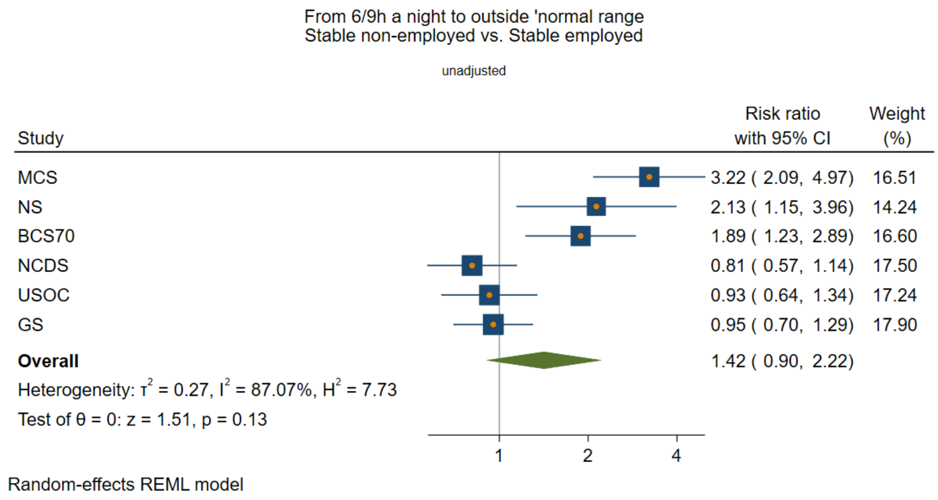
From 6/9h a night to outside 'normal range
Stable non-employed vs. Stable employed
unadjusted
Study
Risk ratio with 95% CI
Weight (%)
MCS 3.22 (2.09, 4.97) 16.51
NS 2.13 (1.15, 3.96) 14.24
BCS70 1.89 (1.23, 2.89) 16.60
NCDS 0.81 (0.57, 1.14) 17.50
USOC 0.93 (0.64, 1.34) 17.24
GS 0.95 (0.70, 1.29) 17.90
Overall 1.42 (0.90, 2.22)
Heterogeneity: τ^2 = 0.27, I^2 = 87.07%, H^2 = 7.73
Test of θ = 0: z = 1.51, p = 0.13
1
2
4
Random-effects REML model

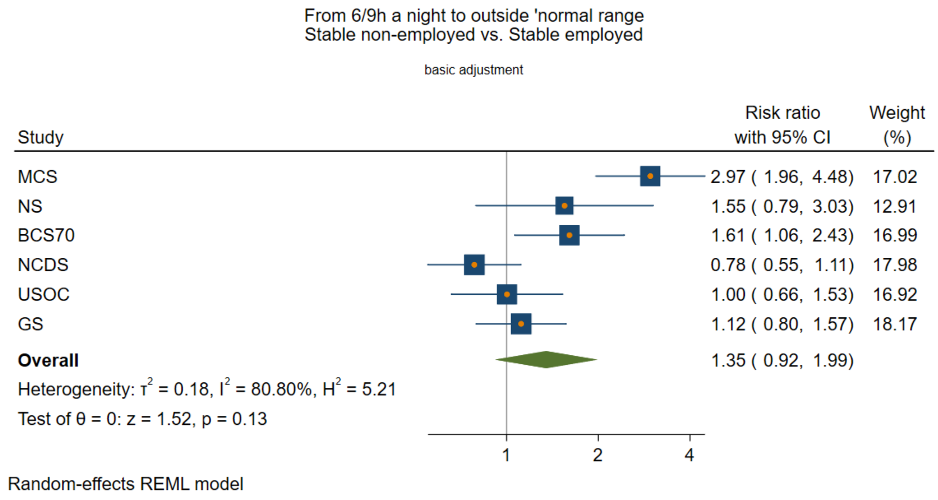
From 6/9h a night to outside 'normal range
Stable non-employed vs. Stable employed
basic adjustment
Study
Risk ratio with 95% CI
Weight (%)
MCS 2.97 (1.96, 4.48) 17.02
NS 1.55 (0.79, 3.03) 12.91
BCS70 1.61 (1.06, 2.43) 16.99
NCDS 0.78 (0.55, 1.11) 17.98
USOC 1.00 (0.66, 1.53) 16.92
GS 1.12 (0.80, 1.57) 18.17
Overall 1.35 (0.92, 1.99)
Heterogeneity: τ^2 = 0.18, I^2 = 80.80%, H^2 = 5.21
Test of θ = 0: z = 1.52, p = 0.13
1
2
4
Random-effects REML model

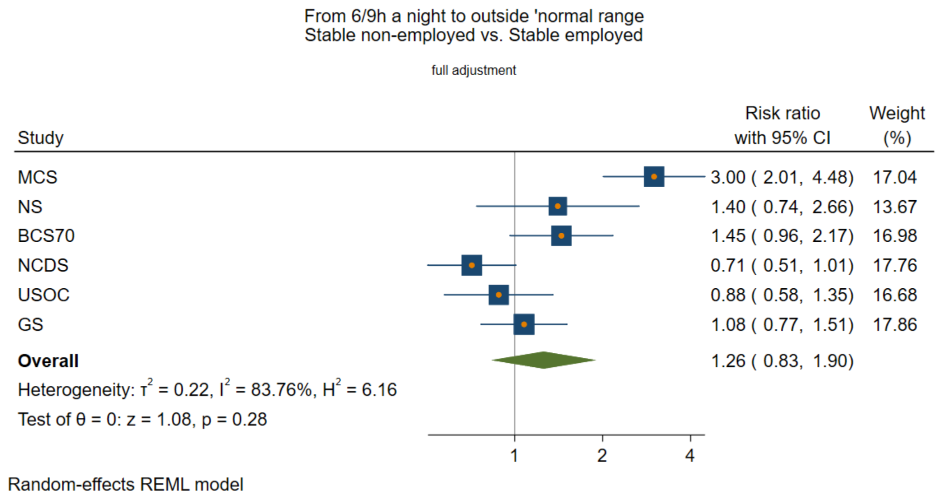
From 6/9h a night to outside 'normal range
Stable non-employed vs. Stable employed
full adjustment
Study
Risk ratio with 95% CI
Weight (%)
MCS 3.00 (2.01, 4.48) 17.04
NS 1.40 (0.74, 2.66) 13.67
BCS70 1.45 (0.96, 2.17) 16.98
NCDS 0.71 (0.51, 1.01) 17.76
USOC 0.88 (0.58, 1.35) 16.68
GS 1.08 (0.77, 1.51) 17.86
Overall 1.26 (0.83, 1.90)
Heterogeneity: τ^2 = 0.22, I^2 = 83.76%, H^2 = 6.16
Test of θ = 0: z = 1.08, p = 0.28
1
2
4
Random-effects REML model

Figure set 11: From outside ‘normal range’ to 6/9h a night

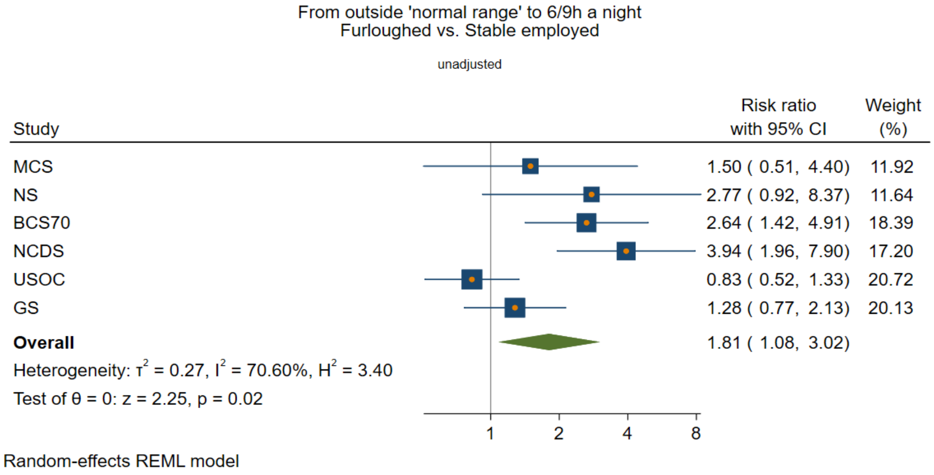

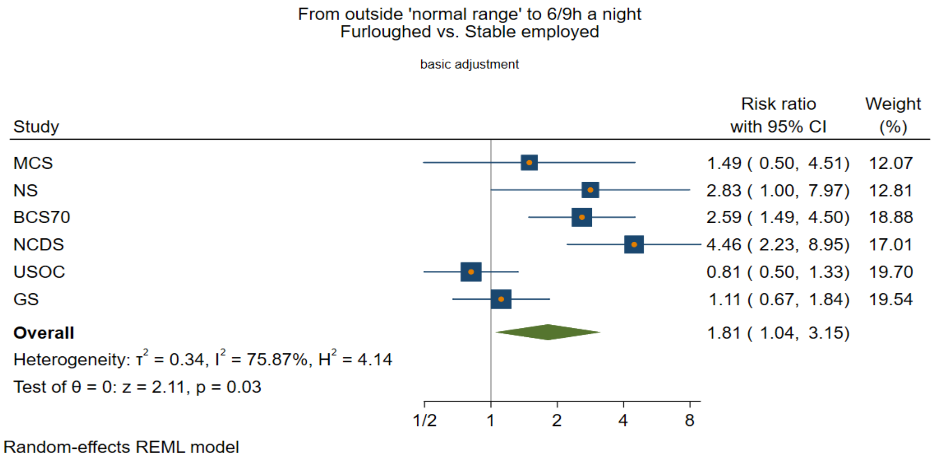

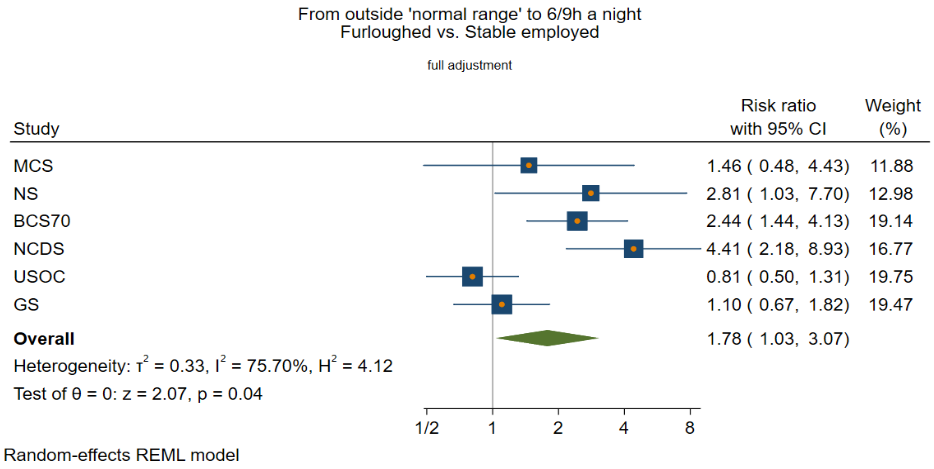

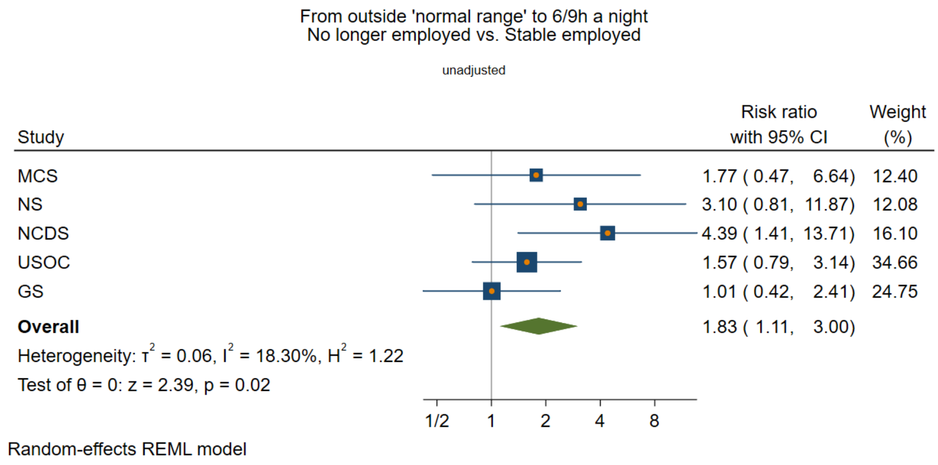
From outside 'normal range' to 6/9h a night
No longer employed vs. Stable employed
unadjusted
Study
Risk ratio with 95% CI
Weight (%)
MCS 1.77 (0.47, 6.64) 12.40
NS 3.10 (0.81, 11.87) 12.08
NCDS 4.39 (1.41, 13.71) 16.10
USOC 1.57 (0.79, 3.14) 34.66
GS 1.01 (0.42, 2.41) 24.75
Overall 1.83 (1.11, 3.00)
Heterogeneity: τ² = 0.06, I² = 18.30%, H² = 1.22
Test of θ = 0: z = 2.39, p = 0.02
1/2 1 2 4 8
Random-effects REML model

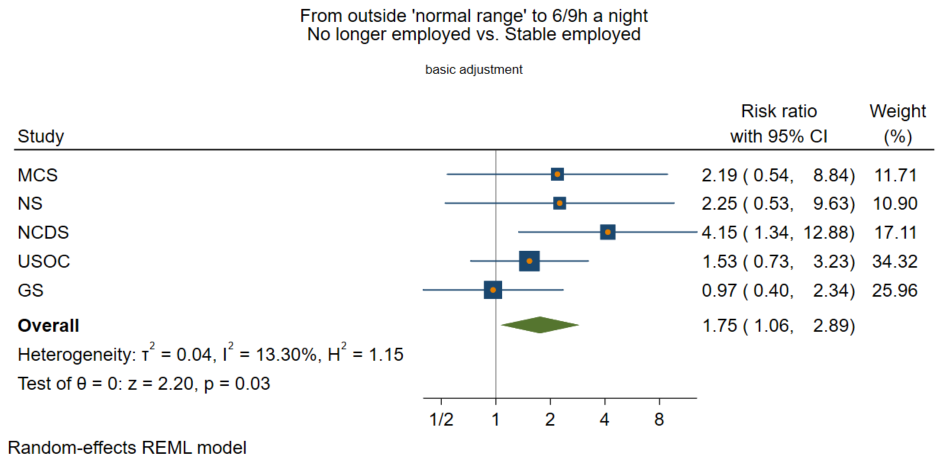
From outside 'normal range' to 6/9h a night
No longer employed vs. Stable employed
basic adjustment
Study
Risk ratio with 95% CI
Weight (%)
MCS 2.19 (0.54, 8.84) 11.71
NS 2.25 (0.53, 9.63) 10.90
NCDS 4.15 (1.34, 12.88) 17.11
USOC 1.53 (0.73, 3.23) 34.32
GS 0.97 (0.40, 2.34) 25.96
Overall 1.75 (1.06, 2.89)
Heterogeneity: τ² = 0.04, I² = 13.30%, H² = 1.15
Test of θ = 0: z = 2.20, p = 0.03
1/2 1 2 4 8
Random-effects REML model

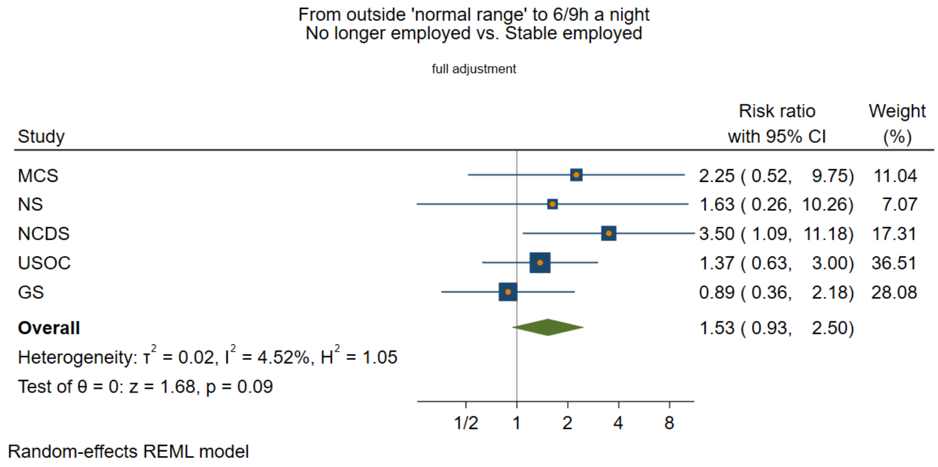
From outside 'normal range' to 6/9h a night
No longer employed vs. Stable employed
full adjustment
Study
Risk ratio with 95% CI
Weight (%)
MCS 2.25 (0.52, 9.75) 11.04
NS 1.63 (0.26, 10.26) 7.07
NCDS 3.50 (1.09, 11.18) 17.31
USOC 1.37 (0.63, 3.00) 36.51
GS 0.89 (0.36, 2.18) 28.08
Overall 1.53 (0.93, 2.50)
Heterogeneity: τ² = 0.02, I² = 4.52%, H² = 1.05
Test of θ = 0: z = 1.68, p = 0.09
1/2 1 2 4 8
Random-effects REML model

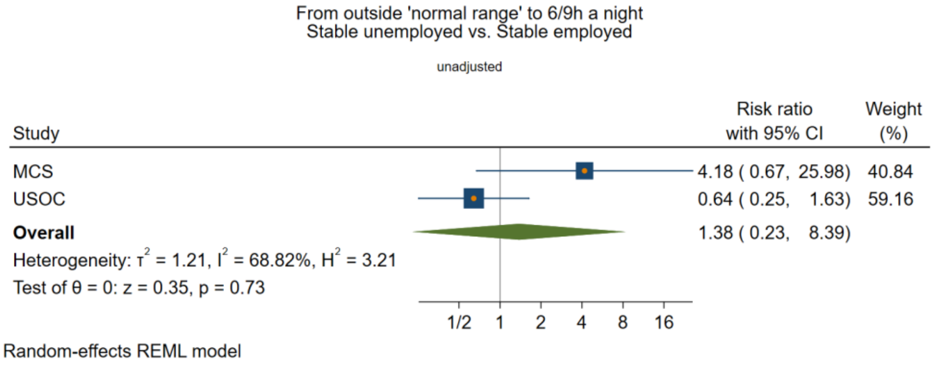
From outside 'normal range' to 6/9h a night
Stable unemployed vs. Stable employed
unadjusted
Study
Risk ratio with 95% CI
Weight (%)
MCS 4.18 (0.67, 25.98) 40.84
USOC 0.64 (0.25, 1.63) 59.16
Overall 1.38 (0.23, 8.39)
Heterogeneity: τ² = 1.21, I² = 68.82%, H² = 3.21
Test of θ = 0: z = 0.35, p = 0.73
1/2 1 2 4 8 16
Random-effects REML model

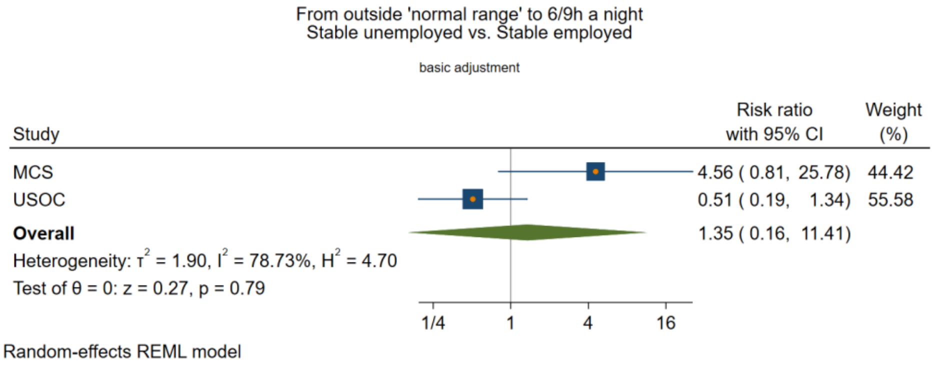
From outside 'normal range' to 6/9h a night
Stable unemployed vs. Stable employed
basic adjustment
Study
Risk ratio with 95% CI
Weight (%)
MCS 4.56 (0.81, 25.78) 44.42
USOC 0.51 (0.19, 1.34) 55.58
Overall 1.35 (0.16, 11.41)
Heterogeneity: τ² = 1.90, I² = 78.73%, H² = 4.70
Test of θ = 0: z = 0.27, p = 0.79
1/4 1 4 16
Random-effects REML model

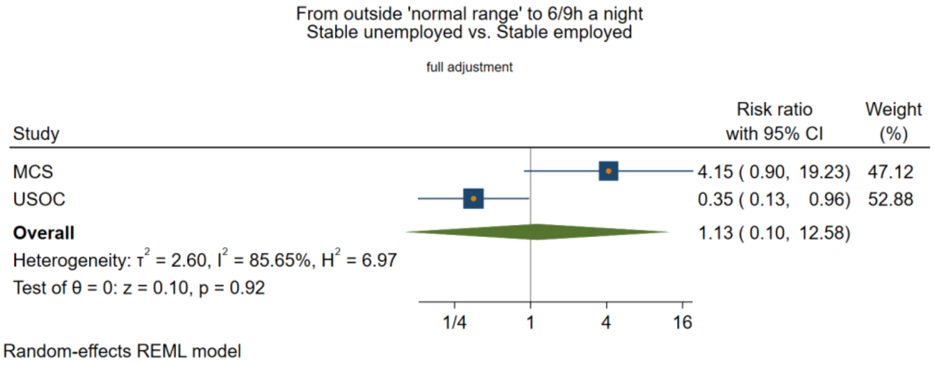
From outside 'normal range' to 6/9h a night
Stable unemployed vs. Stable employed
full adjustment
Study
Risk ratio with 95% CI
Weight (%)
MCS 4.15 (0.90, 19.23) 47.12
USOC 0.35 (0.13, 0.96) 52.88
Overall 1.13 (0.10, 12.58)
Heterogeneity: τ² = 2.60, I² = 85.65%, H² = 6.97
Test of θ = 0: z = 0.10, p = 0.92
1/4 1 4 16
Random-effects REML model

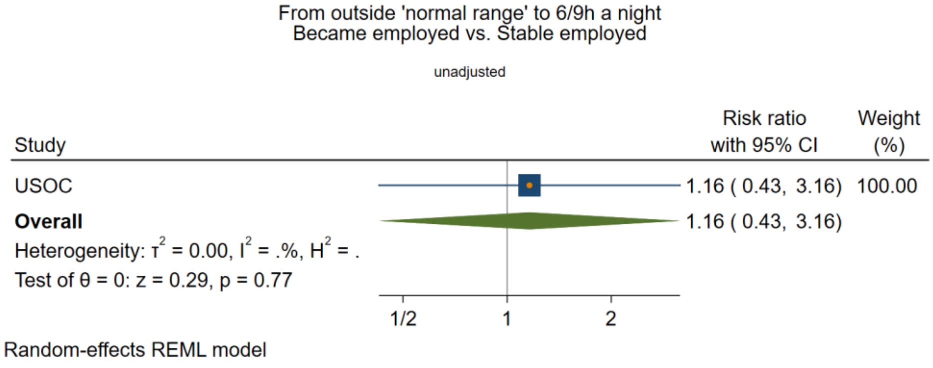
From outside 'normal range' to 6/9h a night
Became employed vs. Stable employed
unadjusted
Study
Risk ratio with 95% CI
Weight (%)
USOC
1.16 (0.43, 3.16)
100.00
Overall
1.16 (0.43, 3.16)
Heterogeneity: τ² = 0.00, I² = .%, H² = .
Test of θ = 0: z = 0.29, p = 0.77
1/2
1
2
Random-effects REML model

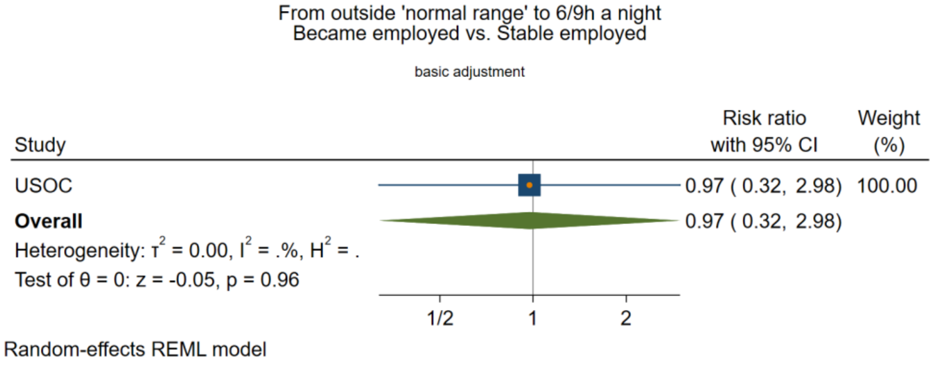
From outside 'normal range' to 6/9h a night
Became employed vs. Stable employed
basic adjustment
Study
Risk ratio with 95% CI
Weight (%)
USOC
0.97 (0.32, 2.98)
100.00
Overall
0.97 (0.32, 2.98)
Heterogeneity: τ² = 0.00, I² = .%, H² = .
Test of θ = 0: z = -0.05, p = 0.96
1/2
1
2
Random-effects REML model

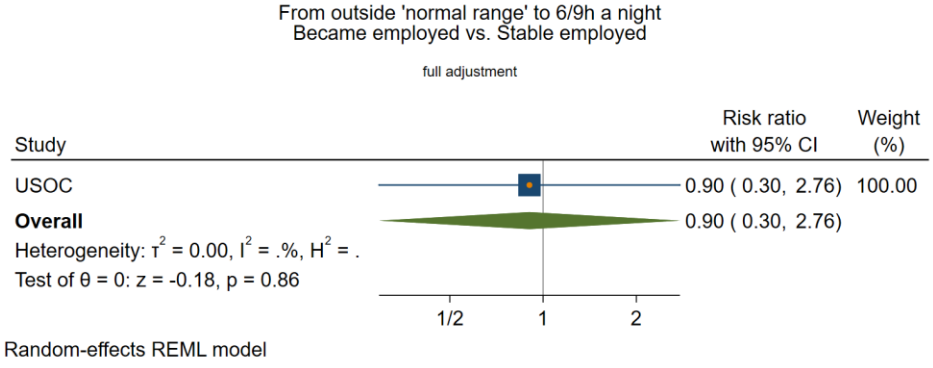
From outside 'normal range' to 6/9h a night
Became employed vs. Stable employed
full adjustment
Study
Risk ratio with 95% CI
Weight (%)
USOC
0.90 (0.30, 2.76)
100.00
Overall
0.90 (0.30, 2.76)
Heterogeneity: τ² = 0.00, I² = .%, H² = .
Test of θ = 0: z = -0.18, p = 0.86
1/2
1
2
Random-effects REML model

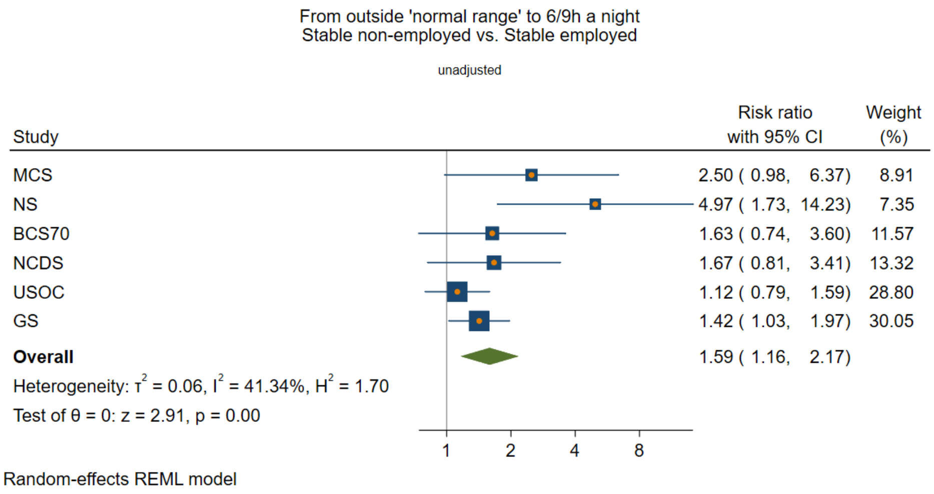
From outside 'normal range' to 6/9h a night
Stable non-employed vs. Stable employed
unadjusted
Study
Risk ratio with 95% CI
Weight (%)
MCS 2.50 (0.98, 6.37) 8.91
NS 4.97 (1.73, 14.23) 7.35
BCS70 1.63 (0.74, 3.60) 11.57
NCDS 1.67 (0.81, 3.41) 13.32
USOC 1.12 (0.79, 1.59) 28.80
GS 1.42 (1.03, 1.97) 30.05
Overall 1.59 (1.16, 2.17)
Heterogeneity: τ^2 = 0.06, I^2 = 41.34%, H^2 = 1.70
Test of θ = 0: z = 2.91, p = 0.00
1 2 4 8
Random-effects REML model

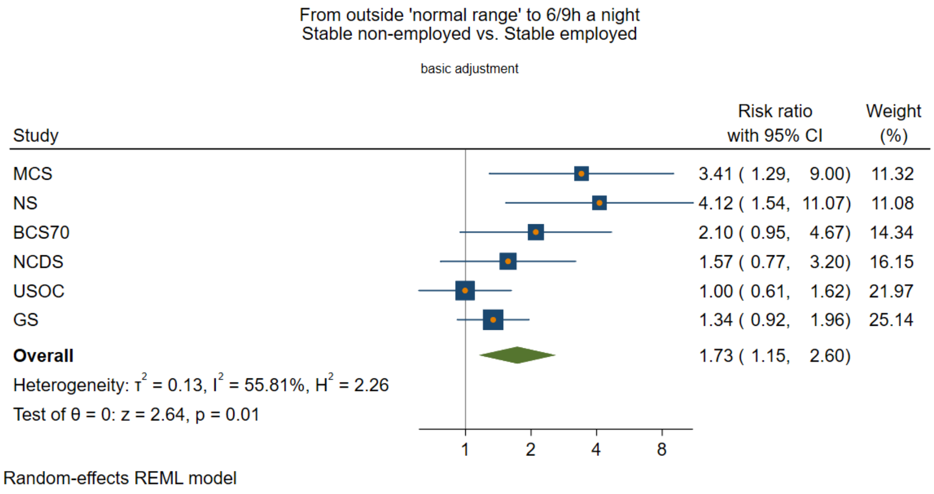
From outside 'normal range' to 6/9h a night
Stable non-employed vs. Stable employed
basic adjustment
Study
Risk ratio with 95% CI
Weight (%)
MCS 3.41 (1.29, 9.00) 11.32
NS 4.12 (1.54, 11.07) 11.08
BCS70 2.10 (0.95, 4.67) 14.34
NCDS 1.57 (0.77, 3.20) 16.15
USOC 1.00 (0.61, 1.62) 21.97
GS 1.34 (0.92, 1.96) 25.14
Overall 1.73 (1.15, 2.60)
Heterogeneity: τ^2 = 0.13, I^2 = 55.81%, H^2 = 2.26
Test of θ = 0: z = 2.64, p = 0.01
1 2 4 8
Random-effects REML model

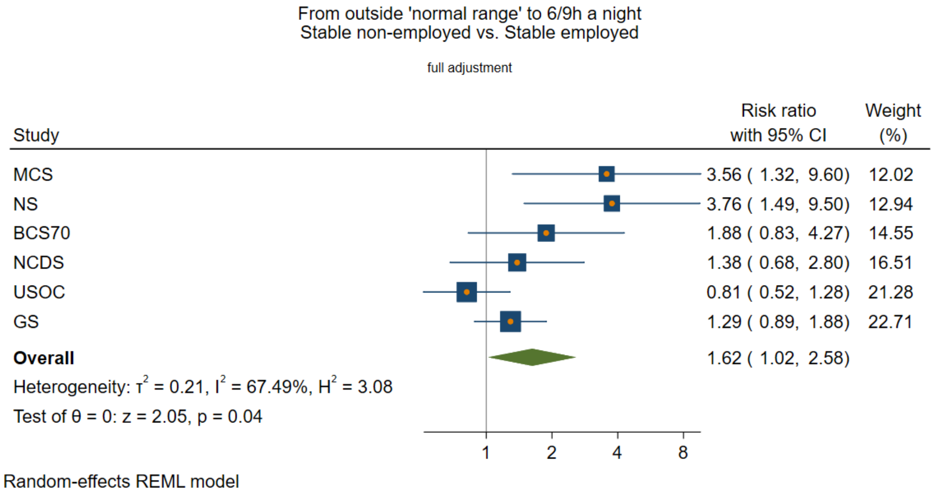
From outside 'normal range' to 6/9h a night
Stable non-employed vs. Stable employed
full adjustment
Study
Risk ratio with 95% CI
Weight (%)
MCS 3.56 (1.32, 9.60) 12.02
NS 3.76 (1.49, 9.50) 12.94
BCS70 1.88 (0.83, 4.27) 14.55
NCDS 1.38 (0.68, 2.80) 16.51
USOC 0.81 (0.52, 1.28) 21.28
GS 1.29 (0.89, 1.88) 22.71
Overall 1.62 (1.02, 2.58)
Heterogeneity: τ^2 = 0.21, I^2 = 67.49%, H^2 = 3.08
Test of θ = 0: z = 2.05, p = 0.04
1 2 4 8
Random-effects REML model
